# Post-infarction KLHL40-mediated regulation of cardiac sarcomeric integrity and function

**DOI:** 10.7717/peerj.21375

**Published:** 2026-06-05

**Authors:** Xiao Yu, Xuexue Liu, Jian Zhao, Xiaopeng Zhang, Wei Song, Xin Kong, Rui Zhang, Jianrong Bao, Mingxiu Dai, Hazrat Bilal, Chang Shi, Jing Li, Lei Sun

**Affiliations:** 1 Department of Pathology and Forensic Medicine, College of Basic Medical Sciences, Dalian Medical University, Dalian, China; 2 Department of Pathology, The First Affiliated Hospital of Dalian Medical University, Dalian, China

**Keywords:** Cardiac sarcomere, KLHL40, Z-disc, Calcium accumulation, Inflammation and apoptosis

## Abstract

**Background:**

Cardiac sarcomeric remodeling after myocardial infarction (MI) plays a pivotal role in post-injury cardiac dysfunction, yet the molecular mechanisms governing this process remain incompletely understood.

**Objective:**

We aimed to elucidate the role of Kelch-like protein 40 (KLHL40) in sarcomeric protein remodeling and calcium signaling regulation after MI.

**Methods:**

By integrating transcriptomic and proteomic datasets with experimental validation, we characterized dynamic changes in KLHL40 expression and its effects on sarcomeric components and myocyte state following hypoxia. KLHL40 expression was evaluated in human post-MI heart tissues and hypoxia-induced H9C2 cells. Functional assays revealed its regulation of key Z-disc-associated proteins (MYOT, CAPZA, and TCAP). Mechanistic candidates were identified *via* His-tag affinity capture (OCTET NTA) and MS-based enrichment analysis. Intracellular calcium levels, calpain activity, inflammasome activation, and apoptosis were assessed, and pathways governing KLHL40 degradation were examined using specific inhibitors.

**Results:**

KLHL40 expression was markedly upregulated within 6 hours (95% CI [−72.53 to −0.9783]; *P* = 0.04) post-MI and selectively reduced the expression of MYOT (95% CI [0.321–1.08]; *P* = 0.003), CAPZA (95% CI [0.449–0.902]; *P* < 0.001), and TCAP (95% CI [0.130–0.689]; *P* = 0.01) without affecting ACTN2 (95% CI [−1.07 to 1.16]; *P* > 0.99) or FLNC (95% CI [−0.0673–0.280]; *P* = 0.22). Mechanistically, KLHL40 was associated with altered handling and calpain activation, including regulation of ATP2A2 and intracellular calcium levels. KLHL40 markedly reduced NLRP3 (95% CI [0.6576–1.145]; *P* < 0.001) and Cleaved Caspase-1 (95% CI [0.6276–0.8550]; *P* < 0.001) expression. The BCL2/BAX ratio decreased (95% CI [0.1108–0.8928]; *P* = 0.02) following KLHL40 knockdown and increased (95% CI [−1.641 to −0.3629]; *P* = 0.007) with overexpression. Additionally, KLHL40 turnover was regulated via the proteasomal, calpain, and autophagic pathways.

**Conclusion:**

KLHL40 maintains sarcomeric integrity and cardiomyocyte viability after myocardial infarction by modulating calpain signaling and limiting inflammasome activation and apoptosis. KLHL40 expression and stability are tightly regulated through proteasomal, autophagic, and calpain-dependent pathways.

## Introduction

Myocardial infarction (MI), often caused by coronary artery occlusion, triggers a cascade of cellular damage that extends beyond the initial area of myocyte necrosis, impacting the surrounding surviving myocytes (Lodrini & Goumans, 2021). For these viable myocytes, the preservation of sarcomeric structure and function is pivotal to maintaining residual cardiac contractility (Mendoza & Rassier, 2020; Prill & Dawson, 2020). Sarcomeres, the fundamental contractile units of cardiomyocytes, consist of myosin thick filaments and actin thin filaments anchored at the Z-disc. The Z-disc itself is a structurally complex and functionally diverse hub composed of key proteins such as ACTN, FLNC, DES, CAPZA, MYOT, and TCAP (Frank et al., 2007; Gregorio et al., 1998; Knöll et al., 2002; Pyle et al., 2002; Valle et al., 1997). Recent evidence indicates that sarcomeric gene expression undergoes marked temporal changes post-MI (Kuppe et al., 2022; Liu et al., 2021). However, the mechanisms underlying sarcomere degradation remain largely unclear (Ahuja et al., 2004; Engel, Schebesta & Keating, 2006), emphasizing the clinical value of elucidating how sarcomeric proteins are regulated after MI.

KLHL40, a BTB-Kelch repeat containing protein, is essential for myogenesis and structural integrity, primarily through its localization to the thin filaments (Kawase et al., 2015; Stogios & Privé, 2004). Extensively studied in skeletal muscle myogenesis, KLHL40 regulates E2F1-DP1 activity (Gong et al., 2015) and stabilizes sarcomeric proteins nebulin (NEB) and leiomodin 3 (LMOD3), both of which are skeletal muscle-specific sarcomeric proteins (Garg et al., 2014). Consistent with this, pathogenic KLHL40 mutations cause severe skeletal muscle disorders such as nemaline myopathy with severe sarcomeric disruption (Stogios & Privé 2004; Turgut et al., 2024). *Klhl40* deficiency further decreases skeletal muscle-specific ACTN3 and CAPZ and causes abnormal DES ubiquitination (Mansur et al., 2023).

In cardiac muscle, however, KLHL40’s role remains largely unexplored. Current evidence indicated that KLHL40 has a less pronounced effect on cardiac sarcomeres assembly compared with skeletal muscle (Garg et al., 2014). Preliminary analyses of publicly available transcriptomic and proteomic datasets revealed time-dependent alterations in the expression of several key sarcomeric proteins, such as MYOT, TCAP, CAPZA, and ACTN2, indicating that the early phase following MI may represent a critical regulatory period for sarcomeric remodeling. In addition, KLHL40, a sarcomere-associated protein that is highly conserved across species and known to stabilize thin filament components in skeletal muscle displayed a transient increase in expression during the early stage of MI, followed by a gradual decline. These findings prompted us to propose that KLHL40 may have an unrecognized function in preserving sarcomeric organization and cardiomyocyte viability after MI, possibly through mechanisms involving calcium-dependent proteolysis and post-infarction structural remodeling.

In this study, we analyzed the molecular alterations of sarcomeric proteins following MI, explored the role of KLHL40 in sarcomere stability and cell state, and investigated its degradation pathways.

## Materials & Methods

### Human MI specimen tissues

The myocardial tissue samples from 18 autopsy cases were collected from the Department of Pathology and Forensic Medicine, Dalian Medical University. All specimens were obtained from the anterior wall of the left ventricle near the apex. To ensure RNA and protein stability, all samples were collected within 48 h postmortem, immersed in RNA later within 1 h after dissection, and stored at −80 °C until analysis. RNA quality was confirmed by spectrophotometric analysis and RNA Integrity Number (RIN > 7.0). Protein integrity was verified by uniform GAPDH levels and total protein quantification.

The cohort included three groups (*n* = 6 each).

(1) Control group: individuals who died of non-cardiac causes, with no history of congenital or acquired heart disease; four males and two females, aged 27–56 years.

(2) Early-phase MI group: patients with coronary artery disease who experienced sudden cardiac death within three days of acute MI onset; four males and two females, aged 30–63 years.

(3) Late-phase MI group: individuals with a documented history of coronary artery disease, in whom the last MI episode had occurred more than seven days prior to death; three males and three females, aged 50–71 years.

All control subjects underwent gross and histological evaluation to confirm the absence of significant coronary atherosclerosis or myocardial injury. Microscopic morphological diagnosis was verified independently by two pathologists, who additionally confirmed and demarcated the border zone and peri-scar regions based on established histological criteria. The case information is provided in Table S1. This study was approved by the Medical Ethics Committee of Dalian Medical University (Approval No. 2024-039). Written informed consent was obtained from family members before inclusion, with documentation archived at the Department of Pathology and Forensic Medicine, Dalian Medical University. The investigation conformed to the principles outlined in the Declaration of Helsinki.

### Histological analysis

Tissue samples were fixed in 4% paraformaldehyde, dehydrated, paraffin-embedded, and sectioned into 3–4 µm sections for histological analysis. For immunohistochemistry (IHC), a Universal Kit (Mouse/Rabbit Polymer Assay System, Cat: PV-6000, ZSGB-BIO, China) was used according to the manufacturer’s instructions. Sections were incubated overnight at 4 °C with KLHL40 primary antibody (1:25, Catalog ID: LS-C165404/228957 LifeSpan BioSciences, Seattle, WA, USA) after blocking the endogenous peroxidase activity. After incubation, DAB (Cat: ZLI-9018, ZSGB-BIO, China) was used for visualization and then counterstained with hematoxylin. The IHC Positive Area (%) was measured by ImageJ (version 1.53c). Masson’s trichrome staining was conducted using a commercial kit (Solarbio, Cat: G1340, China) following the manufacturer’s protocol with minor adjustments: deparaffinized sections were rehydrated to distilled water, stained with Ponceau-Acid Fuchsin Solution for 5–10 min, rinsed for 30 s in Weak Acid Working Solution (distilled water: Weak Acid Solution = 2:1, v/v), differentiated in Phosphomolybic Acid Solution for 1–2 min, and rinsed again with the same working solution for 30 s; sections were then stained with Aniline Blue Solution for 1–2 min, rinsed once more with the working solution, dehydrated rapidly in 95% ethanol (2–3 s) followed by two 5–10 s incubations in absolute ethanol, cleared twice in xylene (1–2 min each), and mounted with resinene.

### RNA-seq data analysis

Raw read quality was assessed, and datasets were retrieved from the Gene Expression Omnibus (GEO). The RNA-seq dataset provides time-dependent mRNA expression profiles in the left ventricles of mice following MI induced by permanent ligation of the left anterior descending (LAD) coronary artery: GSE114695 (1 day, 1 week, and 8 weeks after MI); GSE153494 (10 min, 1 h, 6 h, 1 day, and 3 days after MI); GSE236374 (7 days and 28 days after MI); GSE158415 (1 day after MI). FPKM or RPKM values were used to compare sarcomeric gene expression profiles. Our re-analysis followed the original experimental protocol without modifications.

The GSE114695 dataset was generated under ethical approval from the Animal Care and Use Committee of the Gwangju Institute of Science and Technology (IACUC GIST-2017-006) and Chonnam National University (CNU IACUC-H-2016-36) (Kim et al., 2018). Animal experiments in GSE153494 and GSE236374 were approved by the Institutional Animal Care and Use Committee of Tongji University (Shanghai, China) (Liu et al., 2021) and the Animal Care Committee of the Ethics of Animal Experiments of Fudan University (Yu et al., 2023). Animal experiments in GSE158415 were approved by the Institutional Animal Care and Use Committee of Peking University Health Science Center (LA2019138) (Wang et al., 2021).

### DIA data processing and analysis

Raw data from the PXD020193 dataset (https://www.iprox.cn/page/project.html?id=IPX0002295000), matched to GSE153494 MI time points (10 min, 1 h, 6 h, 1 day, and 3 days), were downloaded and subjected to Data-Independent Acquisition (DIA) analysis. Data processing was performed using Spectronaut™ 14.4.200727.47784, using the same reference database for spectral libray construction. The following parameters were configured in the software: retention time prediction was set to dynamic iRT; MS2-level interference correction was enabled; and cross-run normalization was applied. All results were filtered with a Q Value cutoff of 0.01, corresponding to a false discovery rate (FDR) < 1% (Liu et al., 2021).

### Primer design and validation

All primers were designed to span exon–exon junctions where applicable to prevent amplification of genomic DNA. Specifically, for rat *Klhl40* (NM_001108195.2), the forward primer is located in exon 1 and the reverse primer in exon 2, generating an amplicon of 163 bp. This primer pair was verified by BLAST to uniquely match the intended rat *Klhl40* transcript. For rat *Actb* (NM_031144.3), both forward and reverse primers are located in exon 5, generating an amplicon of 150 bp. For human *KLHL40* (NM_152393.4), the forward primer is located in exon 3 and the reverse primer in exon 4, generating an amplicon of 150 bp. For human *GAPDH* (NM_002046.7), the forward primer is located in exon 6 and the reverse primer at the exon 7–8 junction, generating an amplicon of 150 bp. All primer pairs were validated by BLAST against the respective reference genomes to confirm unique target specificity. Following qRT-PCR, melt curve analysis was performed for each reaction, and a single specific peak was observed for each gene, confirming a single amplicon with no nonspecific products or primer-dimers (Supplemental Information 54).

### qRT-PCR

Total RNA was extracted from cells using RNAkey reagent (Cat: SM139-02A, SEVEN, China). RNA quality and concentration were assessed using a NanoDrop ND-100 spectrophotometer (Thermo Fisher Scientific, Waltham, MA, USA). cDNA synthesis was performed using a reverse transcription kit, and mRNA expression was quantified using SYBR TransStart Top Green qPCR SuperMix (SRQ-01 SEVEN, China). ACTB was used as the internal control for cell experiments, whereas GAPDH served as the internal control for myocardial tissue samples. All qPCR reactions were run in technical duplicates. Gene expression changes were calculated by the 2^−ΔΔCt^ method, with normalization to the respective internal control gene. Primer sequences are listed in Table 1.

**Table 1:** Primer sequences list.

**Genes**	**Primers sequence**
KLHL40-Rat	Forward:CCGGTGGCCTCTTCTACAAC Reverse:CCGACCACGTAGATGGCATTA
ACTB-Rat	Forward:CCTGTGGCATCCATGAAACTAC Reverse:CCAGGGCAGTAATCTCCTTCTG
KLHL40-human	Forward:TGCTCTCCCACATGGACCTT Reverse:CAGTGGCCCCAAAGAGTGA
GAPDH-human	Forward:CATGTTCGTCATGGGTGTGAA Reverse:GGCATGGACTGTGGTCATGAG

### Western blotting

The cells/tissues were lysed using RIPA buffer containing Halt Protease Inhibitor Cocktails (Thermo Fisher Scientific, Waltham, MA, USA, USA). Lysates were cleared by centrifugation. Protein concentrations were determined using a bicinchoninic acid (BCA) assay (Beyotime, China). Equal amounts of protein were separated by SDS-PAGE and transferred onto PVDF membranes. Membranes were incubated overnight at 4 °C with primary antibodies targeting to beta-actin (ACTB) (1:3000, servicebio, Cat: ZB15001-HRP, China), GAPDH (1:10000, selleck, Cat: F0003, China), KLHL40 (1:1000, LifeSpan BioSciences, Catalog ID: LS-C165404/228957, USA), CAPZA1/2 (1:2000, Santa Cruz/Biotechnology, sc-17829, USA), ACTN2 (1:500, Santa Cruz Biotechnology, sc-17829, USA), DES (1:5000, Proteintech, Cat No. 16520-1-AP, China), ATP2A2 (1:3000, Proteintech, Cat No. 67248-1-lg, China), TCAP (1:100, Santa Cruz Biotechnology, sc-25327, USA), FLNC (1:3000, Novusbio, NBP1-89300, USA), MYOT (1:1000, Novusbio, NBP2-94022, USA), CAST (1:2000, Proteintech, Cat No. 12250-1-AP, China), CAPN2 (1:2500, Proteintech, Cat No. 11472-1-AP, China), CAPN1 (1:5000, Proteintech, Cat No. 10538-1-AP, China), BAX (1:20000, HUABIO, Catalog# ET1603-34, China), BCL2 (1:2000, Abways, Catalog No. CY6717, China), NLRP3 (1:1000, Bio-Swamp, Catalog Number: RMAB48867, China), Cleaved Caspase-1 (1:1000, Bio-Swamp, Catalog Number: PAB55588, China) were incubated with at 4 °C overnight. After washing, membranes were incubated with HRP-conjugated secondary antibodies (1:15000, Proteintech, Cat No. SA00001-2/SA00001-1, China). Protein bands were visualized using enhanced chemiluminescence (ECL, Advansta) and captured with a ChemiDoc MP imaging system (Bio-Rad, Hercules, CA, USA). Western blotting quantification was performed using ImageLab 5.1 (Bio-Rad) with normalization to GAPDH/ACTB. Full-length uncropped blots are provided in Supplemental Information.

### Cell culture and treatment

The H9C2 cell line was purchased from Wuhan Procell Life Science & Technology Co., Ltd. (Wuhan, China) and cultured in DMEM (#10313021, Thermo Fisher Scientific, Waltham, MA, USA) supplemented with 10% fetal bovine serum (FBS) and 1% penicillin-streptomycin. Cells were maintained in a humidified incubator at 37 °C with 5% CO_2_. A hypoxic environment was established using an oxygen chamber (Embrient, San Diego, CA, USA) connected to a SMF3001 Model controller (Embrient, San Diego, CA, USA). The chamber was infused with a gas mixture containing 94% N_2_, 5% CO_2_, and 1% O_2_ at a flow rate of 30–35 L/min for 4 min. Following gas infusion, cells were exposed to hypoxic conditions for 0, 3, 6, 12, 18, or 24 h. To ensure adequate humidity, distilled water was added to the chamber, which was then placed in a 37 °C incubator for the duration of the hypoxic exposure. MG132 (Selleck, S2619, China) and chloroquine (Selleck, S6999, China) were diluted with DMSO to the concentration required for the experimental protocols.

### Cell transduction

Lentiviral vectors were used for KLHL40 knockdown and overexpression. KLHL40-specific shRNAs (Target 1: CTGGACTGCGTAGTGCGTGT; Target 2: GGAAACGCGCACGGAAGTAG; Target 3: ATCGGAGATCGCGCTGGACG) and full-length KLHL40 overexpression constructs with a C-terminal 6 ×His tag were transduced into H9C2 cells. Stable cell lines were established by selecting transfected cells with puromycin (2 µg/mL) at least 48 h before use for the described experiments.

### Fluo-4 assay

The transfected cells were incubated with 4 µM Fluo-4 AM working solution containing 0.04–0.05% Pluronic F-127 at 37 °C for 30 min. After Hank’s Balanced Salt Solution (HBSS) washing, cells were incubated in HBSS at 37 °C for an additional 30 min. No additional pharmacological agents were applied to induce cardiomyocyte contraction or relaxation during the assay. Fluorescence was detected using a microscope with excitation at 494 nm and emission at 516 nm.

### MTT assay

Cells were seeded at a density of 5 × 10^3^ cells/well in 96-well plates. After treatment, cells were incubated with MTT reagent for 4 h at 37 °C in the dark. Then DMSO was added into the wells to terminate the reaction and absorbance was measured at 490 nm using a microplate reader.

### Protein fishing *via* octet biolayer interferometry and mass spectrometry analysis

Clarified supernatants were diluted to one mg/mL total protein (BCA assay). His-tagged protein fishing was performed on Octet RED96e system (Sartorius) using NTA biosensors: Baseline: 60 s in kinetics buffer (PBS + 0.01% Tween-20); Loading: 300 s with 25 μg/mL cell lysates; Association: 900 s with lysates; Dissociation: 600 s in kinetics buffer. Reference subtraction was performed using mock-transfected lysates. Data were analyzed using Octet Data Analysis HT v9.0. To remove interference and non-specific binding: proteins caught by sensors with response values < 2 were considered background and assigned to the Mock group, whereas proteins with response values > 2 were retained for analysis in the His-tagged group.

Samples were denatured with 1% SDS (final concentration) at 95 °C for 5 min. Proteins were precipitated using TCA, then resuspended in 8 M urea/100 mM Tris-HCl (pH 8.5). Reduction (10 mM TCEP) and alkylation (40 mM CAA) were performed at 37 °C for 1 h, followed by urea dilution to <2 M. Trypsin digestion (1:50 w/w) proceeded overnight at 37 °C, terminated by acidification to pH 6.0 with TFA. Peptides were desalted using SDB-RPS columns and vacuum-dried. Peptides were analyzed on a Q Exactive HF-X coupled to an UltiMate 3000 RSLCnano system (Thermo Fisher Scientific, Waltham, MA, USA). Column: In-house packed C18 (75 µm × 25 cm, 1.9 µm, 100 A). Gradient: 3–35% B (0.1% FA/3% DMSO/97% ACN) over 120 min at 300 nL/min. MS: DDA top20 mode; 350–1,500 m/z (60K resolution); HCD at 28% NCE. Raw files were processed in MaxQuant (v1.6.6.0) against Uniprot Rat database (2022-12-16) as follows. Modifications: Oxidation (M), Acetyl (N-term), Deamidation (NQ) (variable); Carbamidomethyl (C) (fixed). Search parameters: 20 ppm mass tolerance (MS1/MS2); 1% FDR (peptide/protein). Protein groups were filtered to remove contaminants/reverses. Protein mass spectrometry data were analyzed using the online platform available at https://www.bioinformatics.com.cn/. After data submission, the organism (*Rattus norvegicus*) was specified for function enrichment analysis.

### Analysis of enrichment

The Mock group was set to 0 value, and the His-oe-KLHL40 group was assigned non-zero values. Data analysis was performed using the online platform (https://www.bioinformatics.com.cn/). GO and KEGG enrichment analyses were performed. According to the steps on the website, the corresponding data were uploaded and the species was selected as Rattus norvegicus.

### Statistical analysis

Data are presented as the mean ± standard error of the mean (SEM). Statistical analyses were performed using *t*-tests or analysis of variance (ANOVA) to assess the significance of differences. Data normality was verified using the Shapiro–Wilk test (α = 0.05). For inter-group comparisons involving results from at least three independent experiments, one-way ANOVA was utilized. For ANOVA, *post-hoc* comparisons used Tukey’s correction with adjusted *p*-values reported. Spearman analysis was used for correlation analysis. Statistical significance was determined *via* two-tailed tests, with a *p*-value <0.05 considered statistically significant. All analyses were conducted using GraphPad Prism version 10.0. All analyses included complete biological replicates, and no missing data were identified. For publicly available datasets, only entries with complete quantitative values were used for downstream analysis, and data integrity was verified before statistical testing. To ensure reproducibility, all assays were performed in at least three independent replicates, and signal detection and normalization parameters were optimized prior to final quantitative analysis.

## Results

### Dynamic molecular changes in cardiac sarcomeres following MI

**Figure 1:**
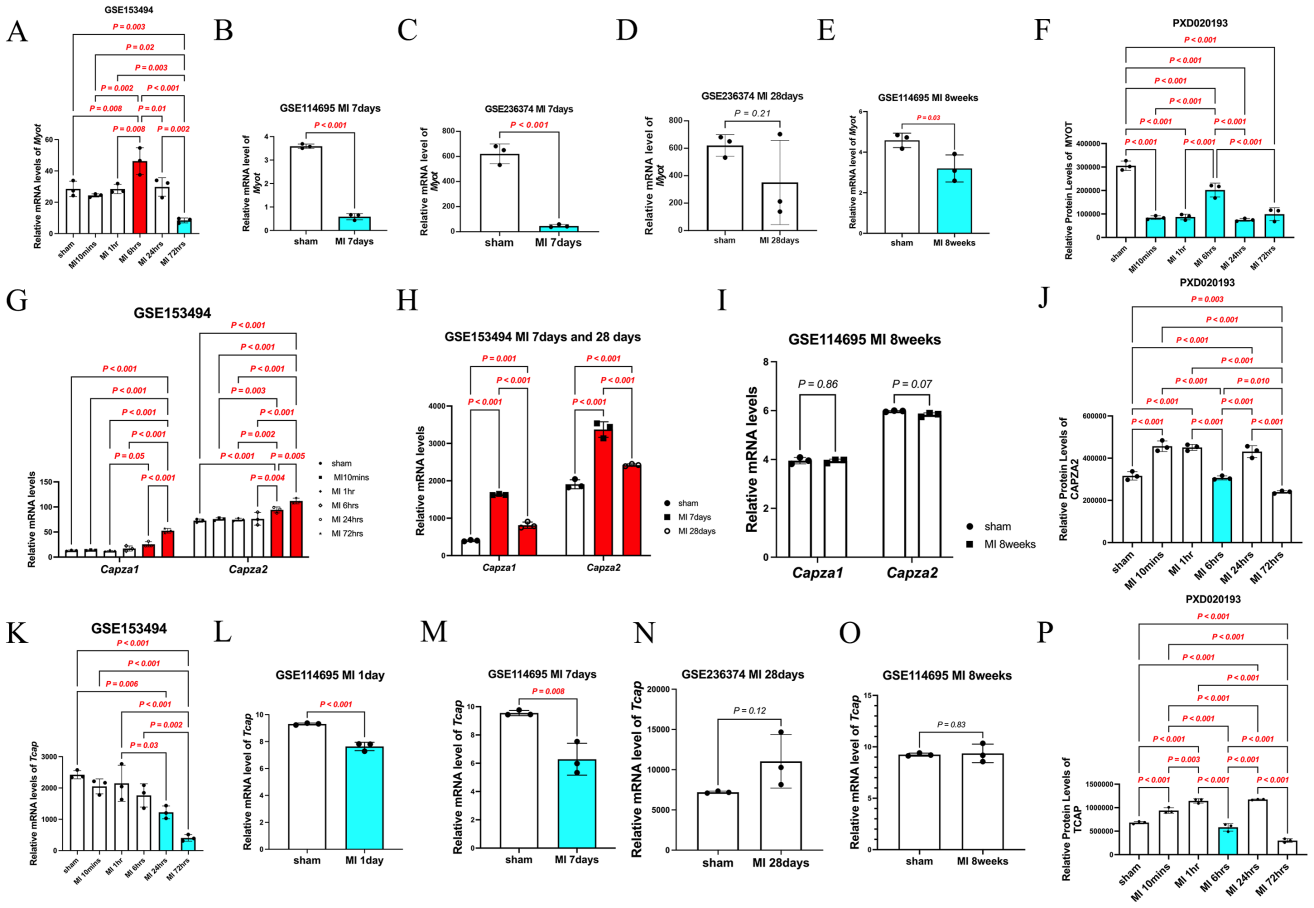
Sarcomeric molecular dynamic changes after MI. (A–E) GEO dataset analysis showed the mRNA expression levels of *Myot* at sham, 10 min, 1 h, 6 h,1 day, 3 days, 7 days, 28 days, 8 weeks after MI. (F) PXD020193 dataset analysis showed that the protein expression levels of MYOT at sham, 10 min, 1 h, 6 h, 1 day, and 3 days after MI. (G–I) GEO datasets analysis showed that the mRNA expression levels of *Capza1* and *Capza2* at sham, 10 min, 1 h, 6 h, 1 day, 3 days, 7 days, 28 days, and 8 weeks after MI. (J) PXD020193 dataset analysis showed that the protein expression levels of CAPZA2 at sham, 10 min, 1 h, 6 h, 1 day, and 3 days after MI. (K–O) GEO dataset analysis showed that the mRNA expression levels of *Tcap* at sham, 10 min, 1 h, 6 h, 1 day, 3 days, 7 days, 28 days, 8 weeks after MI. (P) PXD020193 dataset analysis showed that the protein expression levels of TCAP at sham, 10 min, 1 h, 6 h, 1 day, and 3 days after MI. ns indicates no significant difference . *P < 0.05, **P < 0.01, ***P < 0.001.

To investigate the dynamic molecular changes in cardiac sarcomeres following MI, we analyzed multiple publicly available datasets (GSE114695, GSE153494, GSE236374, and PXD020193). *Myot* mRNA expression (*n* = 3) exhibited a significant peak at 6 h (95% CI [−31.2 to −4.26]; *P* = 0.008) post-MI, followed by a decline during the subsequent days (Figs. 1A–1E). In contrast, MYOT protein levels showed a significant reduction as early as 10 min (95% CI [168,104–274,209]; *P* < 0.001) post-MI and remained consistently lower throughout the acute phase (Fig. 1F). *Capza1*, *Capza2,* and *Tcap* mRNA levels exhibited dynamic regulation during early MI, whereas CAPZA2 (95% CI [92,520–196,215]; *P* < 0.001) and TCAP (95% CI [246,733–519,135]; *P* < 0.001) protein levels demonstrated statistically significant reductions at 6 h post-MI compared to adjacent time points (Figs. 1J and 1P). Additionally, *Flnc* mRNA (95% CI [−1545 to −157.9]; *P* = 0.01) expression was significantly upregulated at this time point (Fig. S1K). Collectively, these findings highlight 6 h post-MI as a critical window of sarcomeric molecular remodeling.

### Dynamic expression profile of KLHL40 in the heart under physiological conditions and following MI

**Figure 2:**
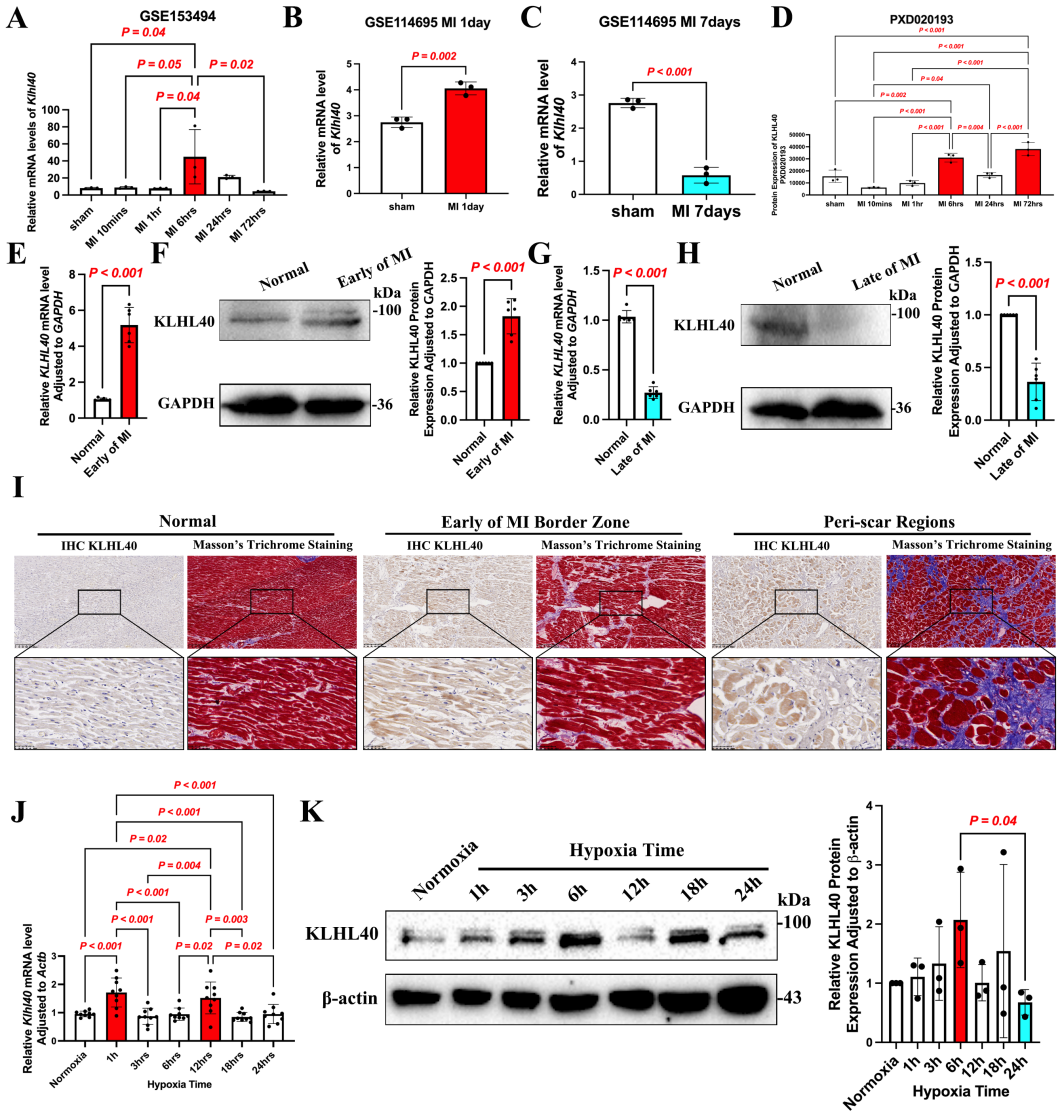
Dynamic expression of KLHL40 after MI. (A–C) GEO dataset analysis showed that the mRNA expression levels of *KLHL40* at sham, 10 min, 1 h, 6 h, 1 day, 3 days, and 7 days after MI. (D) PXD020193 dataset analysis showed that the protein expression levels of KLHL40 at sham, 10 min, 1 h, 6 h, 1 day, and 3 days after MI. (E and F) qRT-PCR and Western blotting semiquantitative analysis of KLHL40 expression in the early stage of human MI. (G and H) qRT-PCR and Western blotting semiquantitative analysis of KLHL40 expression in the late stage of human MI. (I) IHC staining of KLHL40 expression and Masson’s trichrome staining. (J and K) qRT-PCR and Western blotting semiquantitative analyses for KLHL40 in H9C2 cells after 1 h, 3 h, 6 h, 12 h, 18 h, and 24 h hypoxia. ns indicates no significant difference. *P < 0.05, **P < 0.01, ***P < 0.001.

*KLHL40* is highly expressed in human embryonic hearts but minimally in normal adult human hearts, shows >90% amino acid homology across humans, mice, and rats, and may regulate sarcomere assembly under physiological conditions (Figs. S2A–S2E; Supplemental Information 1). Following MI, *Klhl40* mRNA levels peaked at 6 h (95% CI [−72.53 to −0.9783]; *P* = 0.04), accompanied by a corresponding rise in protein levels (95% CI [−25,492 to −5,607]; *P* = 0.002), before declining at later stages (Figs. 2A–2D, Figs. S2F–S2H). Human myocardial samples confirmed early upregulation followed by subsequent downregulation (*n* = 6 per group) (Figs. 2E–2H). IHC analysis revealed robust KLHL40 expression in cardiomyocytes located in the infarct border zone (95% CI [−38.72 to −18.54]; *P* < 0.001) during the early phase of MI (Figs. 2I and S2I). In late MI, the viable myocardium adjacent to the scar consistently (95% CI [−44.50 to −24.32]; *P* < 0.001) exhibited high KLHL40 expression (Figs. 2I and S2I). H9C2 cells, suitable for studies on cardiac sarcomeres (Vejandla et al., 2021; Wang et al., 2024), were cultured under hypoxic conditions (*n* = 3). Hypoxia-treated H9C2 cells recapitulated this dynamic expression pattern, supporting a stress-responsive role for KLHL40 in cardiomyocytes (Figs. 2J and 2K).

### The potential role of KLHL40 in regulating sarcomeric proteins

Analysis of the GSE56570 dataset revealed that deletion of *Klhl40* led to increased mRNA levels of *Myot* and *Tcap* (Figs. S3A–S3C). Proteomic data from PXD020193 demonstrated a negative protein correlations between KLHL40 and CAPZA2 and TCAP post-MI (Figs. S3D and S3E). STRING protein-protein interaction analysis suggested potential functional associations between KLHL40, ACTN2, and MYOT (Fig. S3F). Analysis of datasets GSE158415, GSE114695, GSE153494, and GSE236374 revealed a significant correlation between KLHL40 expression and sarcomere components, further supporting its potential role in sarcomere regulation (Fig. S4).

### KLHL40 expression regulation in H9C2 cells

**Figure 3:**
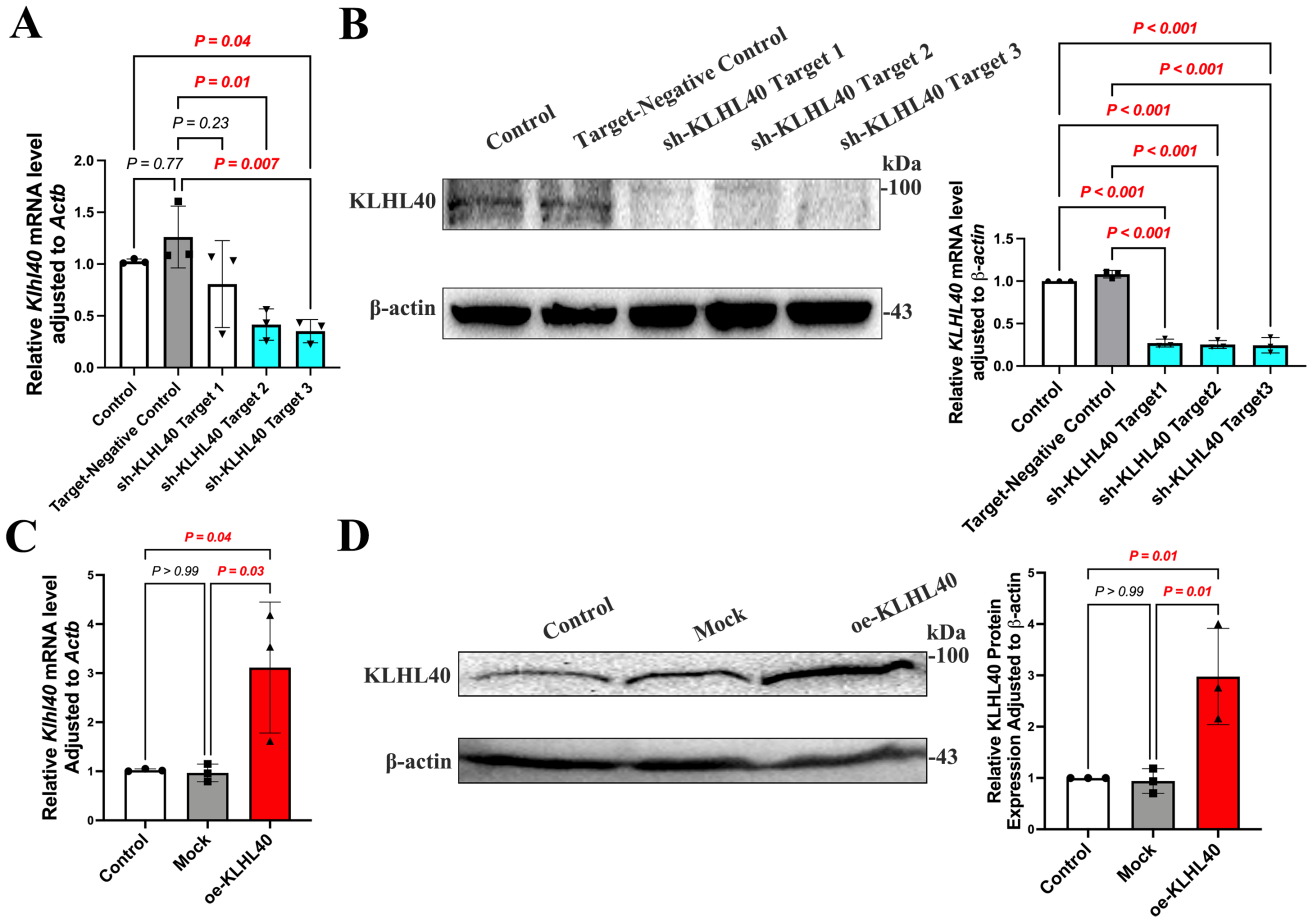
qRT-PCR and Western blotting verified the knockdown and overexpression of KLHL40. (A and B) The efficiency of KLHL40 knockdown was evaluated by qRT-PCR and Western blotting. (C and D) The efficiency of KLHL40 overexpression was evaluated by qRT-PCR and Western blotting. ns indicates no significant difference. *P < 0.05, **P < 0.01, ***P < 0. 001.

To investigate the role of KLHL40 in regulating sarcomeric proteins, we modulated KLHL40 expression (*n* = 3) in H9C2 cells using lentiviral transduction. Sequencing confirmed the effective knockdown of *Klhl40* across all three target sequences (Fig. S5). Notably, Target 3 (95% CI [0.2497–1.569]; *P* = 0.007) demonstrated the most significant reduction in *Klhl40* mRNA levels, and Western blotting analysis further validated the efficacy of Target 3 (95% CI [0.6923–0.9817]; *P* < 0.001) in suppressing KLHL40 protein expression in H9C2 cells (Figs. 3A and 3B). Additionally, qRT-PCR (95% CI [−4.093 to −0.1991]; *P* = 0.03) and Western blotting (95% CI [−3.436 to −0.6349]; *P* = 0.01) confirmed successful KLHL40 overexpression following lentiviral transduction (*n* = 3) (Figs. 3C and 3D).

### KLHL40 promotes the degradation of MYOT, CAPZA, and TCAP while preserving the integrity of Z-disc scaffold proteins

**Figure 4:**
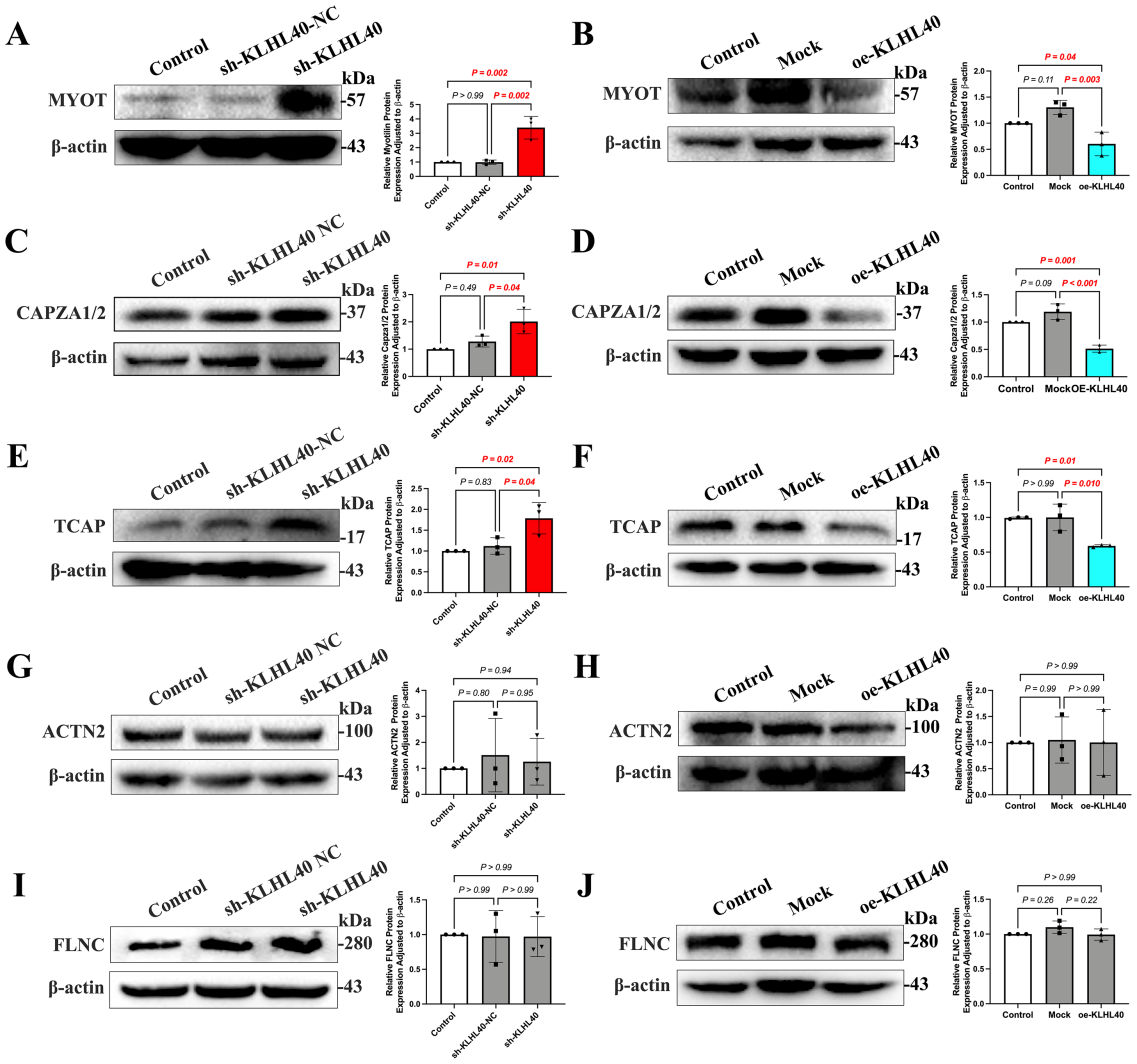
Selective regulation of Z- disc proteins by KLHL40. (A, C, E) Western blotting of MYOT, CAPZA1/2, TCAP expression in sh-KLHL40 cells. (B, D, F) Western blotting of MYOT, CAPZA1/2, TCAP expression in oe -KLHL40 cells. (G and I) Western blotting of ACTN2 and FLNC expression in sh-KLHL40 cells. (H and J) Western blotting of ACTN2 and FLNC expression in oe -KLHL40 cells. ns indicates no significant difference. *P < 0.05, **P < 0.01, ***P < 0.001.

KLHL40 knockdown resulted in increased MYOT (95% CI [−3.57 to −1.24]; *P* = 0.002) and CAPZA1/2 protein (95% CI [−1.926 to −0.002192]; *P* = 0.04) levels (*n* = 3), while KLHL40 overexpression (*n* = 3) led to their reduced expression (95% CI [0.321–1.08]; *P* = 0.003) (95% CI [0.449–0.902]; *P* < 0.001) (Figs. 4A–4D). TCAP, a key protein that links titin to the Z-disc (Gregorio et al., 1998), exhibited a similar trend: its expression increased (95% CI [−1.28 to −0.0528]; *P* = 0.04) with KLHL40 knockdown and decreased (95% CI [0.130–0.689]; *P* = 0.01) with KLHL40 overexpression (Figs. 4E and 4F). Interestingly, KLHL40 did not influence the expression of ACTN2 (95% CI [−2.17 to 2.68]; *P* = 0.95) and FLNC (95% CI [−0.679 to 0.685]; *P >* 0.99) (*n* = 3) (Figs. 4G–4J). These findings suggest that KLHL40 selectively modulates the expression of Z-disc thin myofilament-associated proteins, while leaving the primary Z-disc scaffold proteins unaffected.

### KLHL40 modulates calcium-calpain signaling and intracellular calcium homeostasis

**Figure 5:**
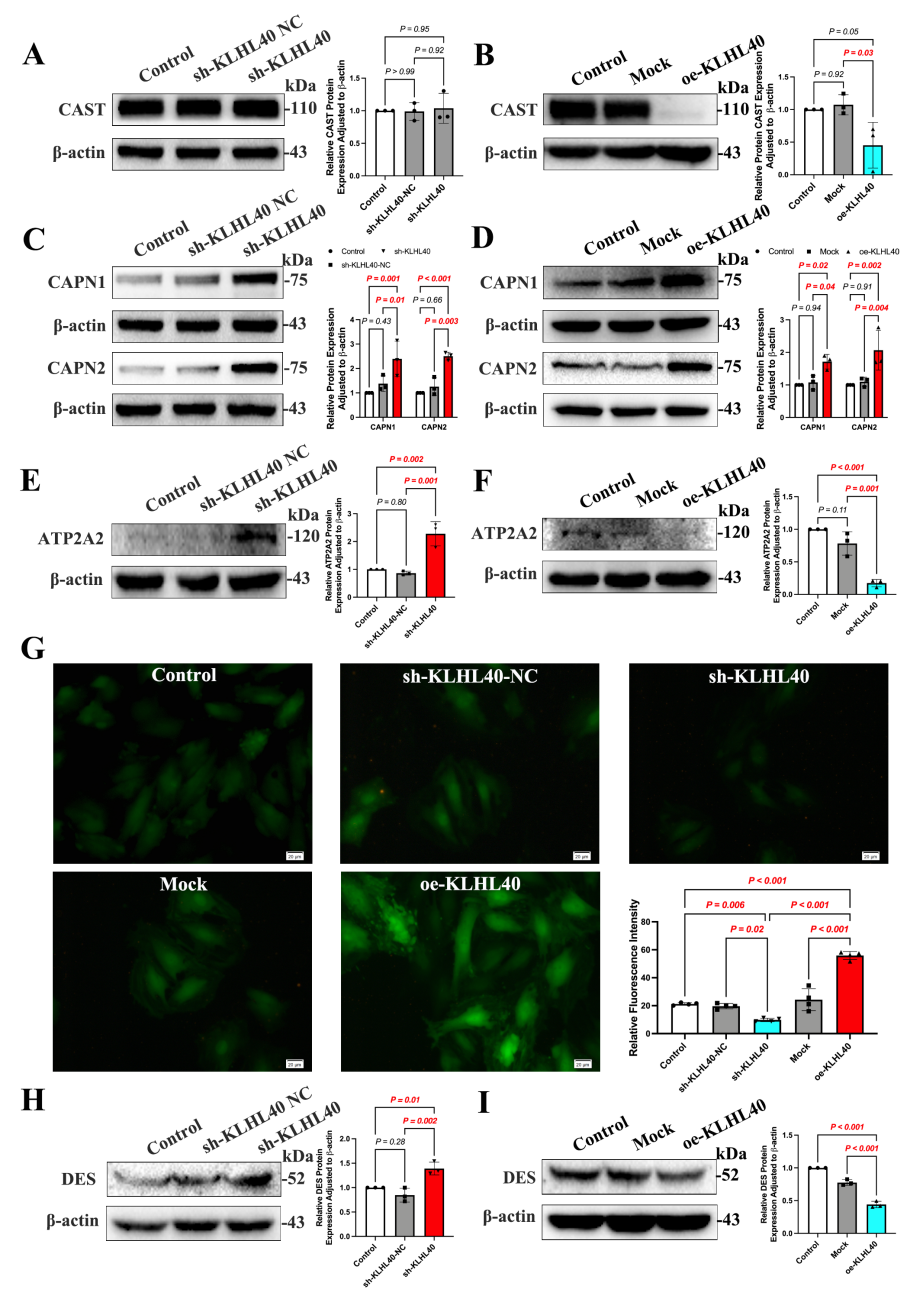
KLHL40 regulates selective sarcomeric protein turnover through intracellular calcium and calpain signaling. (A and B) Western blotting for CAST expression in sh-KLHL40 cells and oe-KLHL40 cells. (C and D) Western blotting for CAPN1 and CAPN2 expression in sh-KLHL40 cells and oe-KLHL40 cells. (E and F) Western blotting for ATP2A2 expression in sh-KLHL40 cells and oe-KLHL40 cells. (G) Representative calcium fluorescence images and related quantification of sh-KLHL40 cells and oe-KLHL40 cells by Fluo-4 Assay. (H and I) Western blotting for DES in sh-KLHL40 cells and oe-KLHL40 cells. ns indicates no significant difference . *P < 0.05, **P < 0.01, ***P < 0.001.

Correlation analysis linked KLHL40 expression with calpain pathway components (Fig. S6). Both KLHL40 knockdown and overexpression markedly increased the expression of CAPN1 (95% CI [−1.801 to −0.2301]; *P* = 0.01) (95% CI [−1.259 to −0.01700]; *P* = 0.04) and CAPN2 (95% CI [−2.034 to −0.4636]; *P* = 0.003) (95% CI [−1.588 to −0.3464]; *P* = 0.004) (Figs. 5C and 5D), while overexpression reduced CAST protein levels (95% CI [0.06625–1.175]; *P* = 0.03) (Fig. 5B). His-tag affinity capture using the OCTET NTA sensor followed by mass spectrometry and enrichment analysis identified calcium-related pathways associated with KLHL40 (Figs. S7A–S7C). KLHL40 downregulation increased ATP2A2 protein levels (95% CI [−2.055 to −0.7749]; *P* = 0.001) (*n* = 3) while overexpression of KLHL40 (*n* = 3), reduced ATP2A2 expression (95% CI [0.3361–0.8803]; *P* = 0.001) (Figs. 5E and 5F), a trend consistent with negative correlations observed for ATP2A1 and ATP2A3 (Figs. S7D and S7E). Consistently, Fluo-4 fluorescence measurements showed reduced intracellular calcium upon KLHL40 knockdown (95% CI [1.339–18.32]; *P* = 0.02) and increased calcium levels following overexpression (95% CI [−40.11 to −23.12]; *P* < 0.001) (Fig. 5G). Furthermore, our results demonstrate that DES protein levels (*n* = 3), a marker of calpains activation, increased (95% CI [−0.8249 to −0.2651]; *P* = 0.002) with KLHL40 knockdown and decreased (95% CI [0.2400–0.4303]; *P* < 0.001) with KLHL40 overexpression (Figs. 5H and 5I) (Huang & Forsberg, 1998; Kent, Spencer & Koohmaraie, 2004; McClung et al., 2009). Together, these findings support that KLHL40 modulates sarcomere protein stability at least partly through calcium-calpain-dependent mechanisms.

### KLHL40 attenuates inflammasome activation and apoptosis in cardiomyocytes

**Figure 6:**
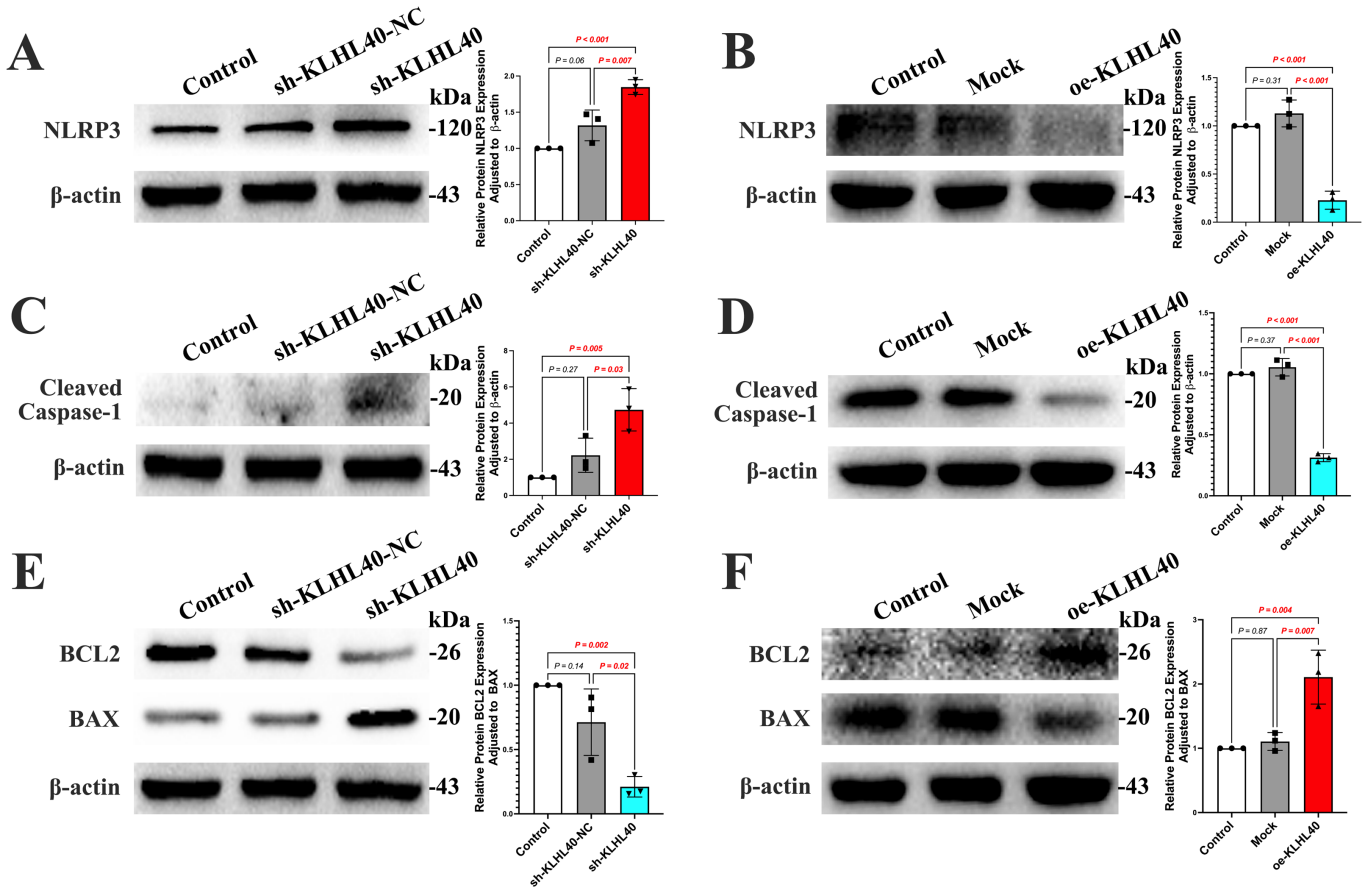
KLHL40 attenuates inflammasome activation and apoptosis in cardiomyocytes. (A and B) Western blotting for NLRP3 expression in sh-KLHL40 cells and oe-KLHL40 cells. (C and D) Western blotting for Cleaved Caspase-1 expression in sh-KLHL40 cells and oe-KLHL40 cells. (E and F) Western blotting for BAX and BCL2 expression in sh-KLHL40 cells and oe-KLHL40 cells. ns indicates no significant difference . *P < 0.05, **P < 0.01, ***P < 0.001.

To further investigate the role of KLHL40 in cell state, we assessed markers of inflammasome and apoptosis activation by Western blotting. KLHL40 knockdown led to an increase in NLRP3 (95% CI [−0.8704 to −0.1897]; *P* = 0.007) and Cleaved Caspase-1 (95% CI [−4.688 to −0.3392]; *P* = 0.03) expression (Figs. 6A and 6C). Conversely, KLHL40 overexpression markedly reduced NLRP3 (95% CI [0.6576–1.145]; *P* < 0.001) and Cleaved Caspase-1 (95% CI [0.6276–0.8550]; *P* < 0.001) expression (Figs. 6B and 6D). In parallel, the BCL2/BAX ratio decreased (95% CI [0.1108–0.8928]; *P* = 0.02) following KLHL40 knockdown and increased (95% CI [−1.641 to −0.3629]; *P* = 0.007) with overexpression (Figs. 6E and 6F). These findings indicate that KLHL40 mitigates cardiomyocyte inflammasome activation and apoptosis, thereby contributing to cellular protection in the post-MI period.

### Mechanisms of KLHL40 degradation

**Figure 7:**
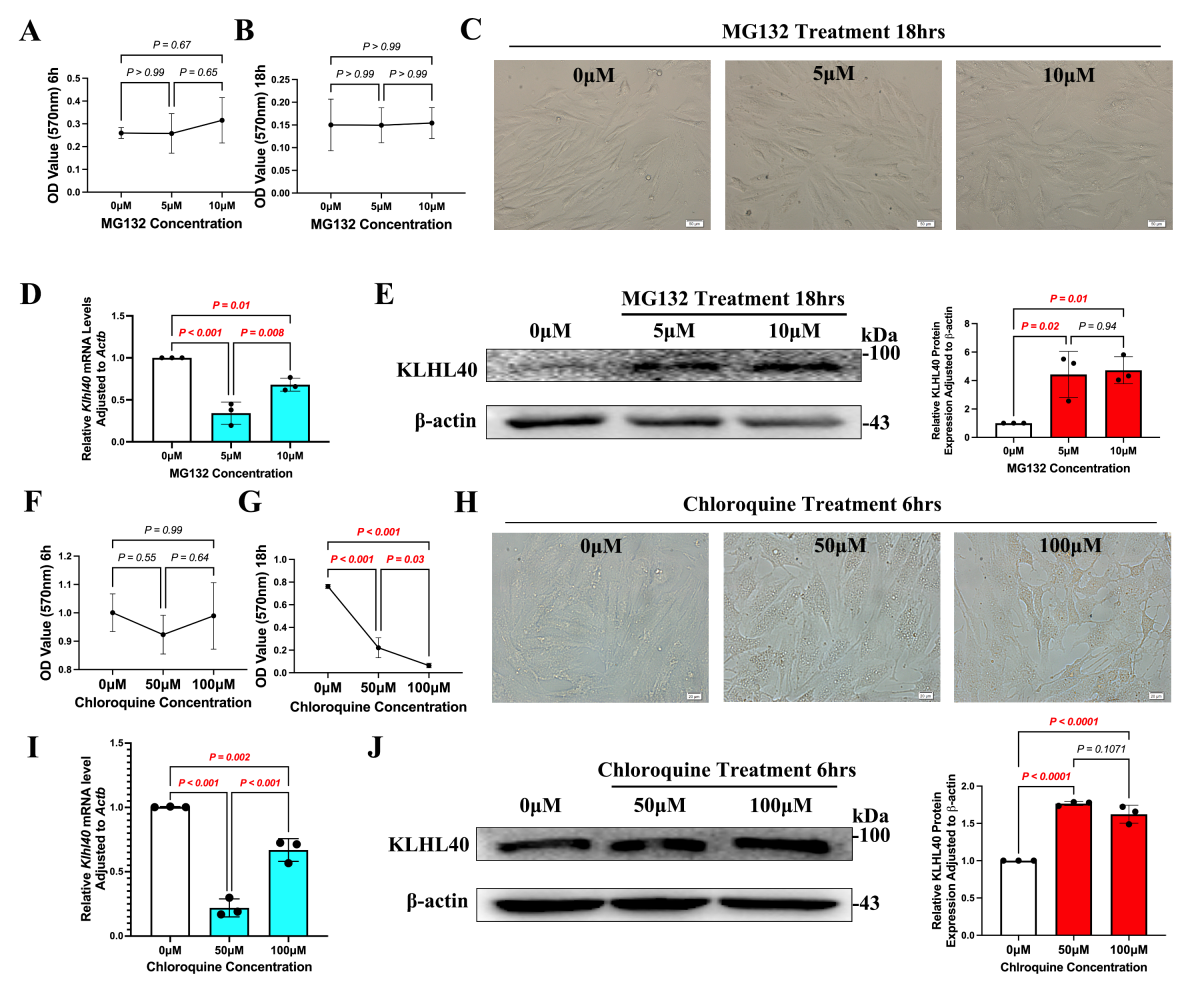
Mechanisms of KLHL40 degradation and stability. (A and B) MTT assay was used to determine the optimal MG132 treatment conditions. (C) Representive light microscopy images of cells treated with MG132 (0 μM, 5 μM, 10 μM). (D and E) qRT-PCR and Western blotting for KLHL40 expression of the cells treated with MG132 (0 μM, 5 μM, 10 μM). (F and G) MTT assay was used to determine the optimal chloroquine treatment conditions. (H) Representative light microscopy images of cells treated with chloroquine (0 μM, 50 μM, 100 μM) for 6 h. (I and J) qRT-PCR and Western blotting for KLHL40 expression of the cells treated with chloroquine (0 μM, 50 μM, 100 μM) for 6 h. ns indicates no significant difference . *P < 0.05, **P <0.01, ***P < 0.001.

Based on MTT results, both the proteasome and calpain inhibitor MG132 (Holecek et al., 2006; Lee & Goldberg, 1998), treated H9C2 at concentrations of 5 μM and 10 μM for 18 h (Figs. 7A and 7B). Morphological observations showed that 5 μM MG132 had minimal effect on cell morphology, while 10 μM MG132 resulted in more pronounced changes, including irregular cell shape and uneven cytoplasm (Fig. 7C). qRT-PCR analysis demonstrated that MG132 treatment decreased *Klhl40* mRNA (95% CI [0.4376–0.8803]; *P* < 0.001) levels, while Western blotting revealed a significant increase in KLHL40 protein (95% CI [−6.148 to −0.7049]; *P* = 0.02) levels (Figs. 7D and 7E).

Chloroquine, an autophagy inhibitor that blocks autophagosome-lysosome fusion (Kimura et al., 2007), was applied at concentrations of 50 μM and 100 μM for 6 h, as determined by MTT assays (Figs. 7F and 7G). Chloroquine induced dose-dependent morphological disruption, mild at 50 μM and pronounced at 100 μM, characterized by cell shape distortion and increased vacuolization (Fig. 7H). qRT-PCR analysis revealed decreased *Klhl40* mRNA (95% CI [0.6224–0.9478]; *P* < 0.001) levels following chloroquine treatment, while Western blotting showed increased KLHL40 protein (95% CI [−0.9447–−0.5877]; *P* < 0.0001) levels (Figs. 7I and 7J). KLHL40 degradation is mediated by the proteasomal, calpain-dependent, and autophagy-lysosome pathways.

## Discussion

### Early sarcomeric instability and protein alterations in myocardial infarction: a critical window for intervention

The early stages of MI represent a narrow therapeutic window that is strongly associated with favorable patient outcomes (Kimura et al., 2007; Lambert et al., 2016). Our study provides compelling evidence that disruption of sarcomeric integrity is one of the earliest molecular events during this period.

MYOT, a critical protein involved in anchoring thin filaments to the Z-disc (Salmikangas et al., 1999), declined within 10 min after MI. Although *Myot* mRNA showed a transient rise at 6 h, protein levels remained below baseline, supporting the presence of early structural imbalance. CAPZA2 and TCAP, both essential for anchoring thin and thick filaments to the Z-disc (Gregorio et al., 1998; Vejandla et al., 2021), showed markedly reduced levels at 6 h post-MI. Additionally, *Flnc* and *Des* transcripts showed an increasing trend at this time point, possibly reflecting a compensatory response. These findings collectively indicate that molecular changes in sarcomeric proteins begin almost immediately after ischemic injury and become pronounced within 6 h. This early phase is marked by significant sarcomeric instability and structural disruption, emphasizing the urgent need for timely intervention to mitigate myocardial damage.

### Dynamic changes in KLHL40 expression post-MI and its potential roles

KLHL40 is highly conserved across species, underscoring its functional importance and evolutionary significance. Previous studies have focused largely on skeletal muscle, where KLHL40 stabilizes sarcomeric proteins and prevents structural collapse (Kawase et al., 2015). Mutations in *KLHL40* disrupt the Z-disc organization and cause severe myopathy characterized by “Z line streaming” (Avasthi, Agarwal & Panigrahi, 2019; Buchignani et al., 2024). Consistent with its critical role in sarcomere regulation, we found that the *KLHL40* gene is highly expressed in the human heart during embryonic development.

Following MI, KLHL40 expression was significantly upregulated, with mRNA and protein levels peaking at 6 h post-MI and declining thereafter. IHC analysis revealed that KLHL40 was highly expressed in the infarct border zone during early myocardial infarction but decreased at later stages. Notably, strong KLHL40 expression persisted in cardiomyocytes surrounding the fibrotic scar, suggesting its complex role in cell remodeling. These findings indicate that KLHL40 may serve not only as a structural regulator but also as a modulator of cardiomyocyte adaptation under stress.

### The complex role of KLHL40 in regulating sarcomere

MYOT cross-links actin filaments and regulates sarcomere assembly, while CAPZA contributes to the dynamic regulation of thin filaments during myofibril elongation (Berger et al., 2022; Salmikangas et al., 2003). KLHL40 knockdown elevated MYOT and CAPZA protein levels, whereas KLHL40 overexpression reduced them. Although MYOT deficiency has limited structural impact in mice (Moza et al., 2007), excessive MYOT can aggravate muscule pathology (Garvey et al., 2008) and impaired MYOT degradation contributes to myopathy progression (Von Nandelstadh et al., 2011). Therefore, the negative regulation of MYOT by KLHL40 plays a protective role in maintaining cardiac function following myocardial injury.

The role of CAPZA in cardiac injury is complex. Reduced CAPZA levels have been associated with improved contractile function and decreased the infarct size after ischemia-reperfusion (Yang & Pyle, 2012). A moderate reduction in CAPZ protein levels can diminish the protein kinase C-dependent dysregulation of myofilament function (Pyle et al., 2002; Pyle et al., 2006). Furthermore, CAPZ-deficient hearts show enhanced contractility (Gaikis et al., 2013). In our study, CAPZA protein levels were significantly elevated in the early stage of MI, and KLHL40 overexpression reduced abundance.

TCAP anchors titin to the Z-disc (Gregorio et al., 1998), and loss of TCAP is associated with elevated cardiac reactive oxygen species levels (Zhao et al., 2024). Consistent with the negative correlation identified in GEO, we found that KLHL40 negatively regulates TCAP expression. This KLHL40-mediated TCAP titration likely functions as an adaptive regulatory mechanism to balance the metabolic stress and structural stability of the myocardium following injury. By promoting DES degradation, KLHL40 may facilitate the clearance of damaged filaments, ensuring necessary sarcomeric turnover (Goll et al., 2008).

ACTN2 and FLNC are key structural proteins of the sarcomeric Z-disc (Mao & Nakamura, 2020; Sjöblom, Salmazo & Djinović-Carugo, 2008; Squire, 1997), and their mutations or abnormalities are known to disrupt Z-disc-mediated signaling pathways, leading to sarcomeric dysfunction (Dalkilic et al., 2006; Rodriguez Garcia et al., 2023). Our findings indicate that neither KLHL40 knockdown nor overexpression affects ACTN2 or FLNC expression, suggesting that KLHL40 does not play a role in stabilizing the primary scaffold structures of the Z-disc.

### KLHL40 promotes intracellular calcium accumulation and calpain activation

Sarcomeric protein turnover is regulated by CAPN1/2 and their endogenous inhibitor CAST (Galvez et al., 2007; Mani et al., 2009; Oglakci-Ilhan et al., 2022). Unexpectedly, both upregulation and downregulation of KLHL40 increased expression of CAPN1 and CAPN2, and KLHL40 overexpression concomitantly reduced CAST, consistent with non-linear calpain regulation. KLHL40 also influenced intracellular calcium handling through ATP2A2, a key mediator that maintains calcium homeostasis in cardiomyocytes by pumping excess calcium into the sarcoplasmic reticulum (Dally et al., 2006). Altered calcium dynamics can modulate both contractility and calpain activation (Siri-Angkul et al., 2021; Wang et al., 2018; Yoder, Wright & Borzok, 2023). Reduced DES levels in the context of KLHL40 overexpression further support activation of calpain-dependent turnover (Huang & Forsberg, 1998; Kent, Spencer & Koohmaraie, 2004; McClung et al., 2009), Collectively, these findings indicate that KLHL40 affects sarcomeric stability not only structurally but also through calcium-sensitive protolytic pathway.

### KLHL40 mitigates inflammasome activation and apoptosis to protect cardiomyocytes

The activation of the NLRP3 inflammasome and downstream Caspase-1 cleavage are critical events that amplify myocardial inflammation and cell death (Sharma & Kanneganti, 2021; Wang et al., 2024). KLHL40 knockdown markedly increased NLRP3 and Cleaved Caspase-1 expression, whereas KLHL40 overexpression reduced them, indicating suppression of the NLRP3/Caspase-1 signaling axis. Similarly, KLHL40 promoted a high BCL2/BAX ratio, consistent with anti-apoptotic signaling (Ali et al., 2023). These observations, combined with the strong expression of KLHL40 detected in myocytes surrounding infarction scars, further supports the cardioprotective effect of KLHL40 in post-infarction cardiac tissue.

### KLHL40 degradation mediated by proteasomal and autophagic pathways

KLHL40 protein is barely detectable in normal myocardium despite measurable mRNA, indicating tight post-transcriptional control. After MI, dissociation between KLHL40 mRNA and protein expression suggests additional regulatory layers.

Both MG132 and chloroquine decreased *Klhl40* mRNA levels, whereas KLHL40 protein abundance was increased. These results implicate proteasome/calpain and autophagy-lysosome pathways in KLHL40 degradation (Holecek et al., 2006; Ueda et al., 2022). MG132 treatment can induce apoptosis in H9C2 cells (Turkieh et al., 2019), while chloroquine has been reported to cause mitochondrial dysfunction, disrupt vesicular transport, and result in pathological changes in cardiac cells (Ashok et al., 2024; Khosa et al., 2018). Despite these potential cytotoxic effects, neither MG132 nor chloroquine induced an increase in *Klhl40* mRNA, ruling out the possibility that the observed elevation in KLHL40 protein levels was caused by cellular stress. These findings further confirm that the proteasome-calpain and autophagy-lysosome pathways are responsible for KLHL40 degradation.

## Limitations

Several limitations warrant consideration. Mechanistic studies were primarily performed in H9C2 cells, which do not fully recapitulate adult cardiomyocyte biology; validation in primary cardiomyocytes and cardiac-specific models will be required. Calpain activation was inferred from downstream markers rather than direct enzymatic assays, and future studies incorporating direct activity measurements would further strengthen these conclusions. Although consistent correlations between KLHL40 and sarcomeric proteins were observed, causal relationships in the intact heart remain to be established. Finally, direct molecular interactions between KLHL40 and its candidate substrates were not defined and will need to be addressed in future work.

## Conclusions

1. KLHL40 maintains sarcomeric integrity and cardiomyocyte viability after myocardial infarction by modulating calpain signaling and limiting inflammasome activation and apoptosis.
2. KLHL40 expression and stability are tightly regulated through proteasomal, autophagic, and calpain-dependent pathways.

## Supplemental Information

10.7717/peerj.21375/supp-1 Supplemental Information 1 Comparison of KLHL40 amino acid sequences in Homo sapiens, Mice, and Rats Homo sapiens: NP_689606; Mice: NP_082478 is mouse; Rats: NP_001101665.
Space non →retention mutations. → Semi-retained mutation: → Preserved mutation * → No mutated amino acids

10.7717/peerj.21375/supp-2 Supplemental Information 2 Autopsy Case information

10.7717/peerj.21375/supp-3 Supplemental Information 3 GEO database analysis after myocardial infarction. (A) GSE153494: *Des* mRNA expression at 0 h, 10 min, 1 h, 6 h, 1 day, and 3 days after MI. (B) GSE236374: *Des* mRNA expression at 7 days after MI. (C) GSE236374: *Des* mRNA expression at 28 days after MI. (D) GSE114695: *Des* mRNA expression at 8 weeks after MI. (E) PXD020193: DES protein expression at 0 h, 10 min, 1 h, 6 h, 1 day, and 3 days after MI. (F) GSE153494: *Actn2* mRNA expression at 0 h, 10 min, 1 h, 6 h, 1 day, and 3 days after MI. (G) GSE114695: *Actn2* mRNA expression at 7 days after MI. (H) GSE236374: *Actn2* mRNA expression at 28 days after MI. (I) GSE114695: *Actn2* mRNA expression at 8 weeks after MI. (J) PXD020193: ACTN2 protein expression at 0 h, 10 min, 1 h, 6 h, 1 day, and 3 days after MI. (K) GSE153494: *Flnc* mRNA expression at 0 h, 10 min, 1 h, 6 h, 1 day, and 3 days after MI. (L) GSE114695: *Flnc* mRNA expression at 7 days after MI. (M) GSE236374: *Flnc* mRNA expression at 28 days after MI. (N) GSE114695: *Flnc* mRNA expression at 8 weeks after MI. (O) PXD020193: FLNC protein expression at 0 h, 10 min, 1 h, 6 h, 1 day, and 3 days after MI. ns indicates no significant difference. *P <0.05, **P <0.01, ***P <0.001.

10.7717/peerj.21375/supp-4 Supplemental Information 4 The expression of KLHL40 in the heart (A) *KLHL40* expression status during human embryonic development ( https://www.ncbi.nlm.nih.gov/gene/131377, The red arrows indicate KLHL40 expression at 11, 17, 18 , and 20 weeks of embryonic development). (B) *KLHL40* expression in adult tissues ( https://www.ncbi.nlm.nih.gov/gene/131377, The expression of KLHL40 in the adult heart is shown in the red arrow). (C) The Human Protein Atlas database IHC shows KLHL40 expression in human myocardial tissue ( https://www.proteinatlas.org/ENSG00000157119-KLHL40/tissue/heart+muscle#img). (D) Expression of *Klhl40* in mouse myocardial tissue ( https://www.ncbi.nlm.nih.gov/gene/72330, The red arrow shows *Klhl40* expression). (E) *Klhl40* expression in rat myocardial tissue (https://www.ncbi.nlm.nih.gov/gene/316088/, the red arrow shows *Klhl40* expression). (F–H) G EO dataset analysis showed that the mRNA expression levels of *KLHL40* at 7 days, 28 days, and 8 weeks after MI. (I) Quantitative analysis of IHC staining intensity in myocardial tissues. ns indicates no significant difference . * *P* < 0.05, ** *P* < 0.01, *** *P* < 0.001 .

10.7717/peerj.21375/supp-5 Supplemental Information 5 The potential role of KLHL40 in regulating sarcomeric proteins (A–C) GSE56570 dataset showing expression of *Klhl40*, *Myot* and *Tcap* in *Klhl40* WT and KO mice. (D) PXD020193 dataset analysis showed a scatter plot analysis of the protein correlation between TCAP and KLHL40 protein levels (*R*^2^ = 0.6051, *P* = 0.0001). (E) PXD020193 dataset analysis showed a scatter plot analysis of the protein correlation between CAPZA2 and KLHL40 (R^2^ = 0.6924, *P* < 0.0001). (F) Protein interacting diagram (https://cn.string-db.org/cgi/network?taskId=bfq6iHMekZSe&sessionId=bjIq9l0N74xX).

10.7717/peerj.21375/supp-6 Supplemental Information 6 Correlation of KLHL40 expression with Z-disc associated sarcomeric genes across multiple GEO myocardial infarction datasets (A) GSE236374: Correlation analyses between *Klhl40* and *Actn2*, *Capza1, Capza2,* and *Myot*. (B) GSE153494: Correlation analyses between *Klhl40* and *Flnc, Des,* and *Myot*. (C) GSE114695: Correlation analyses between *Klhl40* and *Flnc, Des, Myot,* and *Actn2*. (D) GSE158415: Correlation analyses between *Klhl40* and *Des, Flnc, Tcap,* and *Myot*.

10.7717/peerj.21375/supp-7 Supplemental Information 7 Sanger Sequencing (A) Genetic identification of mouse genotype. (B) Three sh-KLHL40 sequences.

10.7717/peerj.21375/supp-8 Supplemental Information 8 Correlation analysis of KLHL40 with inflammasomes and apoptosis-related proteins (A) PXD020193: Correlation analyses between KLHL40 and CAST, CAPN1, and CAPN2. (B) GSE158415: Correlation analyses between *Klhl40* and *Cast, Capn1*, and *Capn2*. (C) GSE236374: Correlation analyses between *Klhl40* and *Capn1* and *Capn2*. (D) PXD020193: Correlation analyses between KLHL40 and BAX. (E) GSE158415: Correlation analyses between *Klhl40* and *Nlrp3, Bax, Bcl2,* and *Casp1.* (F) GSE114695: Correlation analyses between *Klhl40* and *Nlrp3*.

10.7717/peerj.21375/supp-9 Supplemental Information 9 Identification of KLHL40-associated calcium-regulatory proteins by OCTET NTA pull-down and proteomic analysis (A) Protein captured by the NTA sensor in the Mock group. (B) Protein captured by the NTA sensor in the His-tagged-oe-KLHL40 group. (C) Proteomic enrichment analysis performed using https://www.bioinformatics.com.cn/. (D) PXD020193 dataset analysis showed a s catter plot analysis of the c orrelation between ATP2A1 and KLHL40 (*R*^2^ = 0.2984, *P* = 0.0190). (E) PXD020193 dataset analysis showed a s catter plot analysis of the c orrelation between ATP2A3 and KLHL40 (*R*^2^ = 0.444, *P* = 0.0025).

10.7717/peerj.21375/supp-10 Supplemental Information 10 IHC in figure

10.7717/peerj.21375/supp-11 Supplemental Information 11 IHC+IF+Morphology raw data

10.7717/peerj.21375/supp-12 Supplemental Information 12 Sanger Sequencing data

10.7717/peerj.21375/supp-13 Supplemental Information 13 All MTT raw data

10.7717/peerj.21375/supp-14 Supplemental Information 14 GSE56570 raw data

10.7717/peerj.21375/supp-15 Supplemental Information 15 GEO+PXD raw data

10.7717/peerj.21375/supp-16 Supplemental Information 16 QRT-PCR raw data

10.7717/peerj.21375/supp-17 Supplemental Information 17 Figs. 2 and 3 Labeled Western blot

10.7717/peerj.21375/supp-18 Supplemental Information 18 Figs. 2I raw data

10.7717/peerj.21375/supp-19 Supplemental Information 19 Figs. 2J raw data

10.7717/peerj.21375/supp-20 Supplemental Information 20 Figs. 2M raw data

10.7717/peerj.21375/supp-21 Supplemental Information 21 Figs. 3H raw data

10.7717/peerj.21375/supp-22 Supplemental Information 22 Figs. 3J raw data

10.7717/peerj.21375/supp-23 Supplemental Information 23 Fig. 4 Labeled Western blot

10.7717/peerj.21375/supp-24 Supplemental Information 24 Fig. 4A raw data

10.7717/peerj.21375/supp-25 Supplemental Information 25 Fig. 4B raw data

10.7717/peerj.21375/supp-26 Supplemental Information 26 Fig. 4C raw data

10.7717/peerj.21375/supp-27 Supplemental Information 27 Fig. 4D raw data

10.7717/peerj.21375/supp-28 Supplemental Information 28 Fig. 4E raw data

10.7717/peerj.21375/supp-29 Supplemental Information 29 Fig. 4F raw data

10.7717/peerj.21375/supp-30 Supplemental Information 30 Fig. 4G raw data

10.7717/peerj.21375/supp-31 Supplemental Information 31 Fig. 4H raw data

10.7717/peerj.21375/supp-32 Supplemental Information 32 Fig. 4I raw data

10.7717/peerj.21375/supp-33 Supplemental Information 33 Fig. 4J raw data

10.7717/peerj.21375/supp-34 Supplemental Information 34 Fig. 5 Labeled Western blot

10.7717/peerj.21375/supp-35 Supplemental Information 35 Fig. 5A raw data

10.7717/peerj.21375/supp-36 Supplemental Information 36 Fig. 5B raw data

10.7717/peerj.21375/supp-37 Supplemental Information 37 Fig. 5C raw data

10.7717/peerj.21375/supp-38 Supplemental Information 38 Raw Western Blot images from three independent replicate experiments for Figure 5D, showing CAPN1 (Calpain 1) protein expression upon OE-KLHL40 CAPN1 is involved in [brief function, *e.g.*, cytoskeletal remodeling/signaling pathways].

10.7717/peerj.21375/supp-39 Supplemental Information 39 Raw Western Blot images from three independent replicate experiments for Figure 5D, showing CAPN2 (Calpain 2) protein expression upon OE-KLHL40 CAPN2 is involved in [brief function, *e.g.*, cell migration/distinct proteolytic regulation compared to CAPN1]

10.7717/peerj.21375/supp-40 Supplemental Information 40 Fig. 5E raw data

10.7717/peerj.21375/supp-41 Supplemental Information 41 Fig. 5F raw data

10.7717/peerj.21375/supp-42 Supplemental Information 42 Fig. 5H raw data

10.7717/peerj.21375/supp-43 Supplemental Information 43 Fig. 5I raw data

10.7717/peerj.21375/supp-44 Supplemental Information 44 Fig. 6 Labeled Western blot

10.7717/peerj.21375/supp-45 Supplemental Information 45 Fig. 6A raw data

10.7717/peerj.21375/supp-46 Supplemental Information 46 Fig. 6B raw data

10.7717/peerj.21375/supp-47 Supplemental Information 47 Fig. 6C raw data

10.7717/peerj.21375/supp-48 Supplemental Information 48 Fig. 6D raw data

10.7717/peerj.21375/supp-49 Supplemental Information 49 Fig. 6E raw data

10.7717/peerj.21375/supp-50 Supplemental Information 50 Fig. 6F raw data

10.7717/peerj.21375/supp-51 Supplemental Information 51 Fig. 7 Labeled Western blot

10.7717/peerj.21375/supp-52 Supplemental Information 52 Fig. 7E raw data

10.7717/peerj.21375/supp-53 Supplemental Information 53 Fig. 7J raw data

10.7717/peerj.21375/supp-54 Supplemental Information 54 MIQE Checklist and Related Experimental Data

## Additional Information and Declarations

### Data Availability

The following information was supplied regarding data availability: The raw data is available in the Supplemental Files. The sequences are available at NCBI GEO: - GSE56570
- GSE158415
- GSE114695
- GSE153494
- GSE236374
The data is available at iProX: - PXD020193
