# Supplementary material for: Post-infarction KLHL40-mediated regulation of cardiac sarcomeric integrity and function
Source: PeerJ. 2026 Jun 5;14:e21375. doi: 10.7717/peerj.21375 (PMC13245431; doi:10.7717/peerj.21375)
Supplement: Supplemental Information 1 — Homo sapiens: NP_689606; Mice: NP_082478 is mouse; Rats: NP_001101665. Space non →retention mutations. → Semi-retained mutation: → Preserved mutation * → No mutated amino acids [file peerj-14-21375-s001.pdf]

Comparison of KLHL40 amino acid sequences in Homo sapiens, Mice, and Rats

```
NP_689606.2         MALGLEQAEEQRLYQQTLLQDGLKDMLDHGKFLDCVVRAGEREFPCHRLVLAACSPYFRA      60
NP_082478.1         MTLGLEQAEEQRLYQQTLLQDGLKDMLDHGKFLDCVVRVGEREFPCHRLVLAACSPYFRA      60
NP_001101665.2      MTLGLEQAEEQRLYQQTLLQDGLKDMLDHGKFLDCVVRVGEREFPCHRLVLAACSPYFRA      60
                    *:************************************.*********************

NP_689606.2         RFLAEPERAGELHLEEVSPDVVAQVLHYLYTSEIALDEASVQDLFAAAHRFQIPSIFTIC      120
NP_082478.1         RFLAEPDSAGEVRLEEVSPDVVSQVLHYLYTSEIALDEASVQDLFAAAHRFQIPSIFTIC      120
NP_001101665.2      RFLAEPDGAGELRLEEVSPDVVSQVLHYLYTSEIALDEASVQDLFAAAHRFQIPSIFTIC      120
                    ******: ***::*********:*************************************

NP_689606.2         VSFLQKRLCLSNCLAVFRLGLLLDCARLAVAARDFICAHFTLVARDADFLGLSADELIAI      180
NP_082478.1         VSFLQKRLCLANCLAVFRLGLLLDCARLAVAARDFICARFPLVARDNDFLGLSADELIAI      180
NP_001101665.2      VSFLQKRLCLANCLAVFRLGLLLDCARLAVAARDFICARFPLVARDNDFLGLSADELIAI      180
                    **********:***************************:* ***** *************

NP_689606.2         ISSDGLNVEKEEAVFEAVMRWAGSGDAEAQAERQRALPTVFESVRCRLLPRAFLESRVER      240
NP_082478.1         ISSDGLNVEKEEAVFEAVMRWASSGDAEAQAERQRALPTVFESVRCRLLPRAFLETRVER      240
NP_001101665.2      ISSDGLNVEKEEAVFEAVMRWASSGDADVQAEHQRALPTVFESIRCRLLPRAFLESRVER      240
                    **********************.****:.***:**********:***********:****

NP_689606.2         HPLVRAQPELLRKVQMVKDAHEGRITTLRKKKKGKD--GAGAKEADKGTSKAKAEEDEEA      298
NP_082478.1         HPLVRSQPELLRKVQMVKDAHEGRLTTLRKKKKEKGEQTARAKEANQGTEDTKAE--DDE      298
NP_001101665.2      HPLVRSQPELLRKVQMVKDAHEGRLTTLRKKKKEKGEQTARTKEANQGTEDTKAE--DDE      298
                    *****:******************:******** *.   * :***::**..:***  ::

NP_689606.2         ERILPGILNDTLRFGMFLQDLIFMISEEGAVAYDPAANECYCASLSNQVPKNHVSLVTKE      358
NP_082478.1         ERVLPGILNDTLRFGMFLQDLIFMISEEGAVAYDPAANECYCASLSTQIPKNHVSLVTKE      358
NP_001101665.2      ERVLPGILNDTLRFGMFLQDLIFMISEEGAVAYDPAANECYCASLSTQIPKNHVSLVTKE      358
                    **:*******************************************.*:***********

NP_689606.2         NQVFVAGGLFYNEDNKEDPMSAYFLQFDHLDSEWLGMPPLPSPRCLFGLGEALNSIYVVG      418
NP_082478.1         NQVFVAGGLFYNEDNKEDPMSAYFLQFDHLDSEWLGMPPLPSPRCLFGLGEALNAIYVVG      418
NP_001101665.2      NQVFVAGGLFYNEDNKEDPMSAYFLQFDHLDSEWLGMPPLPSPRCLFGLGEALNAIYVVG      418
                    ******************************************************:*****

NP_689606.2         GREIKDGERCLDSVMCYDRLSFKWGESDPLPYVVYGHTVLSHMDLVYVIGGKGSDRKCLN      478
NP_082478.1         GRELKDSEDSLDSVLCYDRLSFKWGESDPLPYAVYGHTVLSHMDLVYVIGGKGKDRKCLN      478
NP_001101665.2      GRELKDSEDSLDSVLCYDRLSFKWGESDPLPYAVYGHSVLSHMDLVYVIGGKGKDRKCLN      478
                    ***:**.* .****:*****************.****:***************.******

NP_689606.2         KMCVYDPKKFEWKELAPMQTARSLFGATVHDGRIIVAAGVTDTGLTSSAEVYSITDNKWA      538
NP_082478.1         KMCVYDPKKFEWKELAPMQTARSLFGATVHDGRIFVAAGVTDTGLTSSSEVYSIADNKWT      538
NP_001101665.2      KMCVYDPKKFEWKELAPMKTARSLFGATVHDGRIFVAAGVTDTGLTSSTEVYSIADNKWT      538
                    ******************:***************:*************:*****:****:

NP_689606.2         PFEAFPQERSSLSLVSLVGTLYAIGGFATLETESGELVPTELNDIWRYNEEEKKWEGVLR      598
NP_082478.1         SFEAFPQERSSLSLVSLAGTLYALGGFATLETESGELVPTELNDIWRYNEDEKKWEGVLR      598
NP_001101665.2      PFEAFPQERSSLSLVSLAGTLYALGGFATLETESGELVPTELNDIWRFNEDEKKWEGVLR      598
                     ****************.*****:***********************:**:*********

NP_689606.2         EIAYAAGATFLPVRLNVLCLTKM     621
NP_082478.1         EIAYAAGATFLPVRLNVLRLTKM     621
NP_001101665.2      EIAYAAGATFLPVRLNVLRLTKM     621
                    ****************** ****
```

Homo sapiens: NP_689606
Mice: NP_082478
Rats: NP_001101665

Space→Non reserved mutation
·→Semi conservative mutation
:→Reserved mutation
*→No mutations
