# Supplementary material for: Post-infarction KLHL40-mediated regulation of cardiac sarcomeric integrity and function
Source: PeerJ. 2026 Jun 5;14:e21375. doi: 10.7717/peerj.21375 (PMC13245431; doi:10.7717/peerj.21375)
Supplement: Supplemental Information 2 [file peerj-14-21375-s002.docx]

Supplementary form Autopsy Case information

Normal

| Serial number | Group | Age | Gender | Cause of Death | Pathological diagnosis | Coronary Artery AS |
| --- | --- | --- | --- | --- | --- | --- |
| 1 | Normal | 27 | Male | Committing trafhi coffences, respiratory and circulatory | No MI | No |
| 2 | Normal | 48 | Male | Death from acute respiratory failure due to pulmonary tuberculosis | No MI | No |
| 3 | Normal | 56 | Male | Committing trafhi coffences, respiratory and circulatory | No MI | No |
| 4 | Normal | 28 | Female | Acute pericardial tamponade death caused by pulmonary artery dissecting aneurysm with rupture and hemorrhage | No MI | No |
| 5 | Normal | 33 | Male | Committing trafhi coffences, respiratory and circulatory | No MI | No |
| 6 | Normal | 45 | Female | Allergic death due to the Penicillin allergies | No MI | No |

Early of MI

| Serial number | Group | Age | Gender | Cause of Death | Pathological diagnosis | Coronary Artery AS |
| --- | --- | --- | --- | --- | --- | --- |
| 1 | Early of MI | 30 | Male | Sudden cardiac death caused by acute myocardial infarction. | Acute MI | All three branches of the coronary artery atherosclerosis, grade Ⅲ stenosis. |
| 2 | Early of MI | 47 | Male | Sudden cardiac death caused by acute myocardial infarction | Acute MI | Left anterior descending artery atherosclerosis with thrombosis, grade Ⅳ stenosis; atherosclerosis thrombosis with stenosis of grade; left circumflex artery atherosclerosis, grade Ⅲ stenosis; right coronary artery atherosclerosis, grade Ⅲ stenosis. |
| 3 | Early of MI | 49 | Male | Sudden cardiac death caused by acute myocardial infarction | Acute MI | All three branches of the coronary artery atherosclerosis, grade Ⅳ stenosis. |
| 4 | Early of MI | 63 | Female | Sudden cardiac death caused by acute myocardial infarction | Acute MI | All three branches of the coronary artery atherosclerosis, left anterior descending coronary artery and right coronary artery stenosis of grade Ⅳ; left circumflex artery stenosis of grade Ⅲ. |
| 5 | Early of MI | 59 | Male | Sudden cardiac death caused by acute myocardial infarction | Acute MI | All three branches of the coronary artery were atherosclerosis, with left anterior descending coronary artery stenosis of grade Ⅳ; left circumflex artery and right coronary artery stenosis of grade Ⅲ. |
| 6 | Early of MI | 62 | Female | Sudden cardiac death caused by acute myocardial infarction. | Acute MI | Left anterior descending artery thrombosis left anterior descending coronary artery, left circumflex artery atherosclerosis grade Ⅳ; right coronary artery atherosclerosis grade Ⅲ. |

Late of MI

| Serial number | Group | Age | Gender | Cause of Death | Pathological diagnosis | Coronary Artery AS |
| --- | --- | --- | --- | --- | --- | --- |
| 1 | Late of MI | 65 | Male | Severe pulmonary infection leads to respiratory failure. | Coronary heart disease with MI tissue repair | Left anterior descending artery atherosclerosis with stenosis of grade Ⅲ; left circumflex artery atherosclerosis with stenosis of grade Ⅰ;right coronary artery atherosclerosis with stenosis of grade Ⅲ. |
| 2 | Late of MI | 66 | Female | Hemorrhagic shock due to traffic accident trauma. | Coronary heart disease with MI tissue repair | Left anterior descending artery atherosclerosis with stenosis of grade Ⅲ; left circumflex artery atherosclerosis with stenosis of grade Ⅱ; right coronary artery atherosclerosis with stenosis of grade Ⅲ. |
| 3 | Late of MI | 54 | Male | Death due to aspiration pneumonia and renal failure following a traffic accident. | Coronary heart disease with MI tissue repair | Left anterior descending artery atherosclerosis with stenosis of grade Ⅳ; left circumflex artery atherosclerosis with stenosis of grade Ⅰ;right coronary artery atherosclerosis with stenosis of grade Ⅱ. |
| 4 | Late of MI | 58 | Female | Acute gastrointestinal bleeding with hemorrhagic shock. | Coronary heart disease with MI tissue repair | Left anterior descending artery atherosclerosis with stenosis of grade Ⅲ; left circumflex artery atherosclerosis with stenosis of grade Ⅲ; right coronary artery atherosclerosis with stenosis of grade Ⅲ. |
| 5 | Late of MI | 50 | Male | Fatal trauma from accidental fall with severe head injury | Coronary heart disease with MI tissue repair | Left anterior descending artery atherosclerosis with stenosis of grade Ⅲ; left circumflex artery atherosclerosis with stenosis of grade Ⅰ; right coronary artery atherosclerosis with stenosis of grade Ⅳ. |
| 6 | Late of MI | 71 | Female | Multi-organ failure due to metastatic liver cancer. | Coronary heart disease with MI tissue repair | Left anterior descending artery atherosclerosis with stenosis of grade Ⅳ; left circumflex artery atherosclerosis with stenosis of grade Ⅲ; right coronary artery atherosclerosis with stenosis of grade Ⅲ. |
