# Supplementary figures and images for: Post-infarction KLHL40-mediated regulation of cardiac sarcomeric integrity and function (part 1 of 5)
Source: PeerJ. 2026 Jun 5;14:e21375. doi: 10.7717/peerj.21375 (PMC13245431; doi:10.7717/peerj.21375)

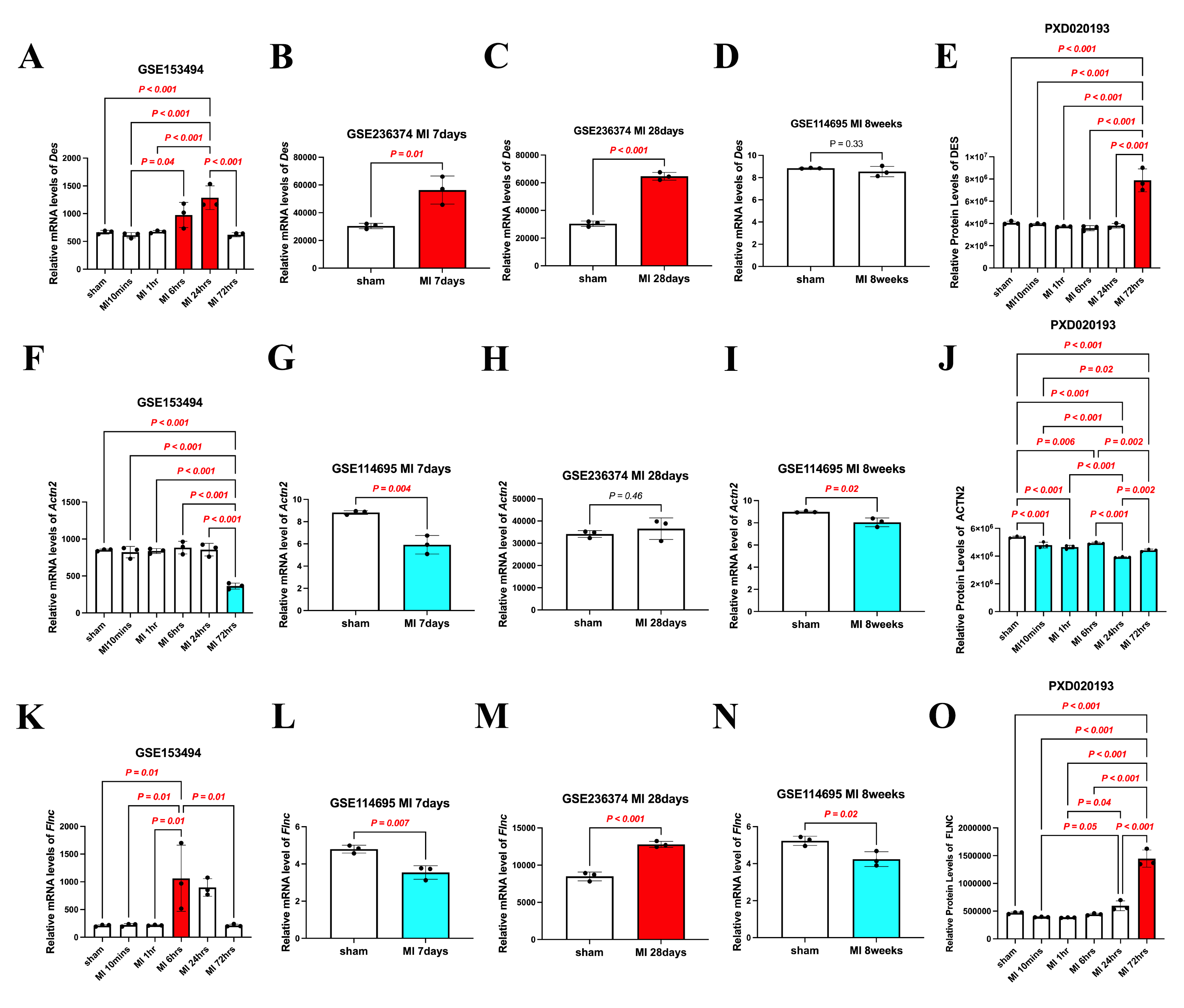

Supplement: Supplemental Information 3 — (A) GSE153494: Des mRNA expression at 0 h, 10 min, 1 h, 6 h, 1 day, and 3 days after MI. (B) GSE236374: Des mRNA expression at 7 days after MI. (C) GSE236374: Des mRNA expression at 28 days after MI. (D) GSE114695: Des mRNA expression at 8 weeks after MI. (E) PXD020193: DES protein expression at 0 h, 10 min, 1 h, 6 h, 1 day, and 3 days after MI. (F) GSE153494: Actn2 mRNA expression at 0 h, 10 min, 1 h, 6 h, 1 day, and 3 days after MI. (G) GSE114695: Actn2 mRNA expression at 7 days after MI. (H) GSE236374: Actn2 mRNA expression at 28 days after MI. (I) GSE114695: Actn2 mRNA expression at 8 weeks after MI. (J) PXD020193: ACTN2 protein expression at 0 h, 10 min, 1 h, 6 h, 1 day, and 3 days after MI. (K) GSE153494: Flnc mRNA expression at 0 h, 10 min, 1 h, 6 h, 1 day, and 3 days after MI. (L) GSE114695: Flnc mRNA expression at 7 days after MI. (M) GSE236374: Flnc mRNA expression at 28 days after MI. (N) GSE114695: Flnc mRNA expression at 8 weeks after MI. (O) PXD020193: FLNC protein expression at 0 h, 10 min, 1 h, 6 h, 1 day, and 3 days after MI. ns indicates no significant difference. *P <0.05, **P <0.01, ***P <0.001. [file peerj-14-21375-s003.png]

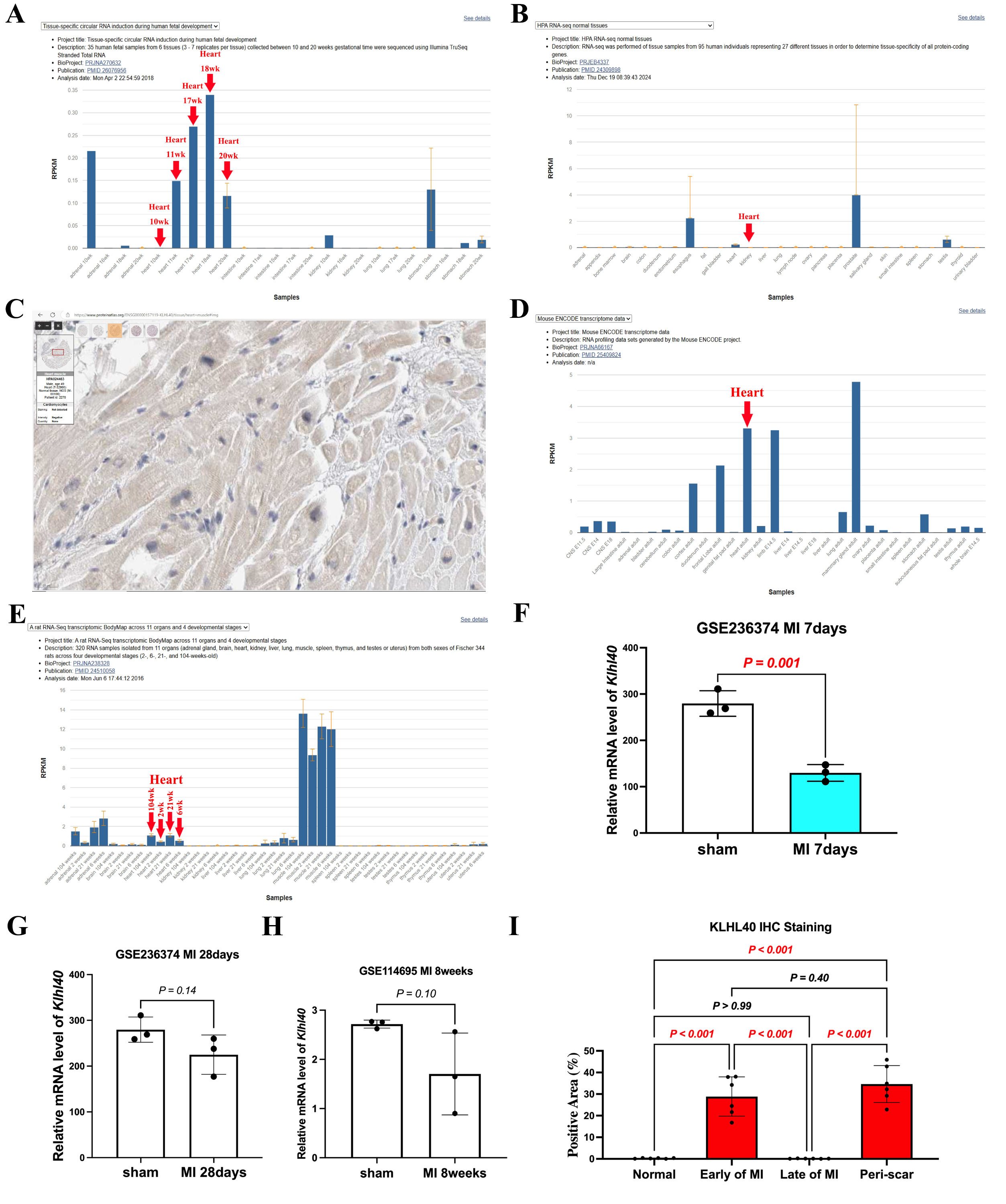

Supplement: Supplemental Information 4 — (A) KLHL40 expression status during human embryonic development ( https://www.ncbi.nlm.nih.gov/gene/131377, The red arrows indicate KLHL40 expression at 11, 17, 18 , and 20 weeks of embryonic development). (B) KLHL40 expression in adult tissues ( https://www.ncbi.nlm.nih.gov/gene/131377, The expression of KLHL40 in the adult heart is shown in the red arrow). (C) The Human Protein Atlas database IHC shows KLHL40 expression in human myocardial tissue ( https://www.proteinatlas.org/ENSG00000157119-KLHL40/tissue/heart+muscle#img). (D) Expression of Klhl40 in mouse myocardial tissue ( https://www.ncbi.nlm.nih.gov/gene/72330, The red arrow shows Klhl40 expression). (E) Klhl40 expression in rat myocardial tissue (https://www.ncbi.nlm.nih.gov/gene/316088/, the red arrow shows Klhl40 expression). (F–H) G EO dataset analysis showed that the mRNA expression levels of KLHL40 at 7 days, 28 days, and 8 weeks after MI. (I) Quantitative analysis of IHC staining intensity in myocardial tissues. ns indicates no significant difference . * P < 0.05, ** P < 0.01, *** P < 0.001 . [file peerj-14-21375-s004.png]

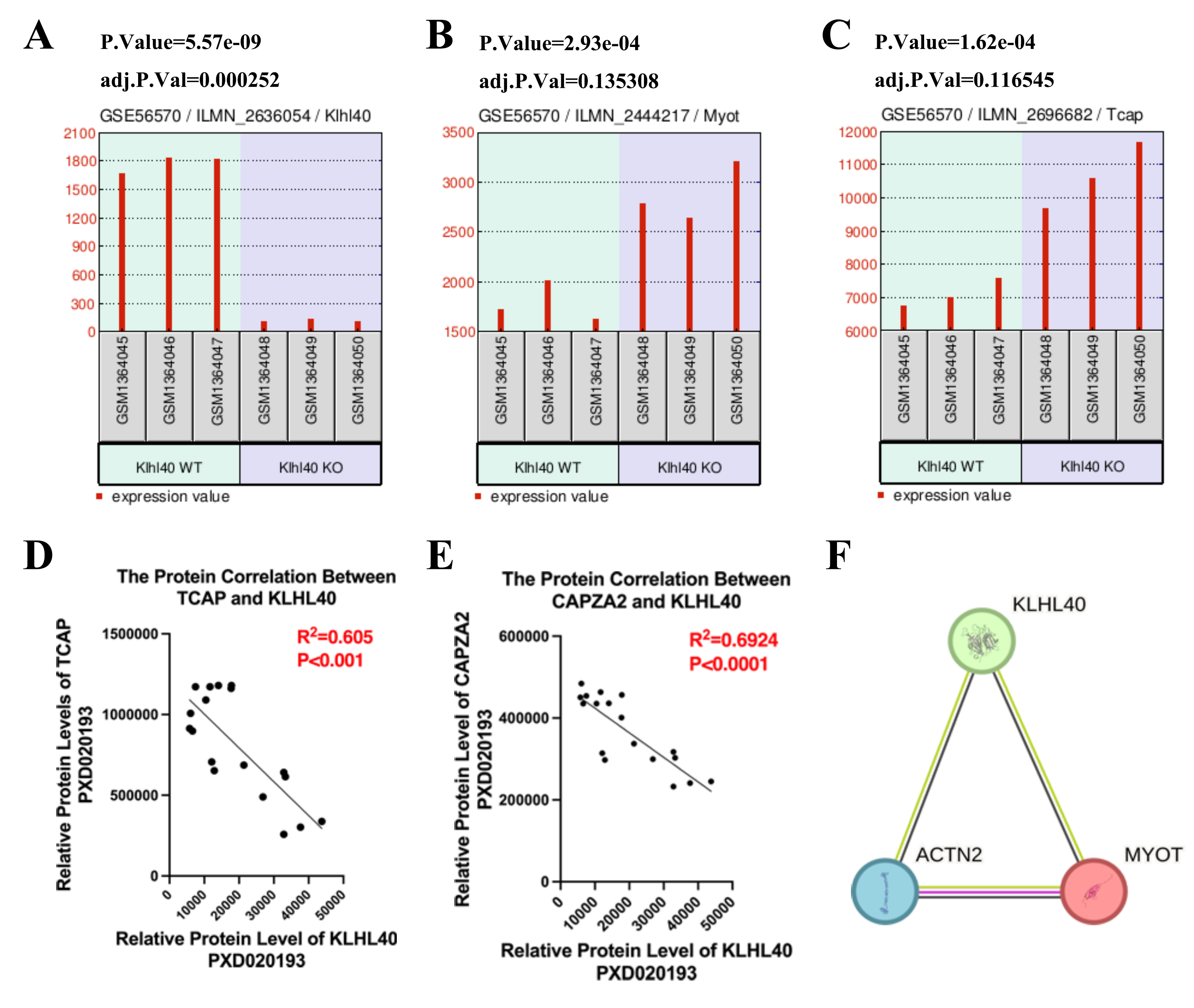

Supplement: Supplemental Information 5 — (A–C) GSE56570 dataset showing expression of Klhl40, Myot and Tcap in Klhl40 WT and KO mice. (D) PXD020193 dataset analysis showed a scatter plot analysis of the protein correlation between TCAP and KLHL40 protein levels (R2 = 0.6051, P = 0.0001). (E) PXD020193 dataset analysis showed a scatter plot analysis of the protein correlation between CAPZA2 and KLHL40 (R2 = 0.6924, P < 0.0001). (F) Protein interacting diagram (https://cn.string-db.org/cgi/network?taskId=bfq6iHMekZSe&sessionId=bjIq9l0N74xX). [file peerj-14-21375-s005.png]

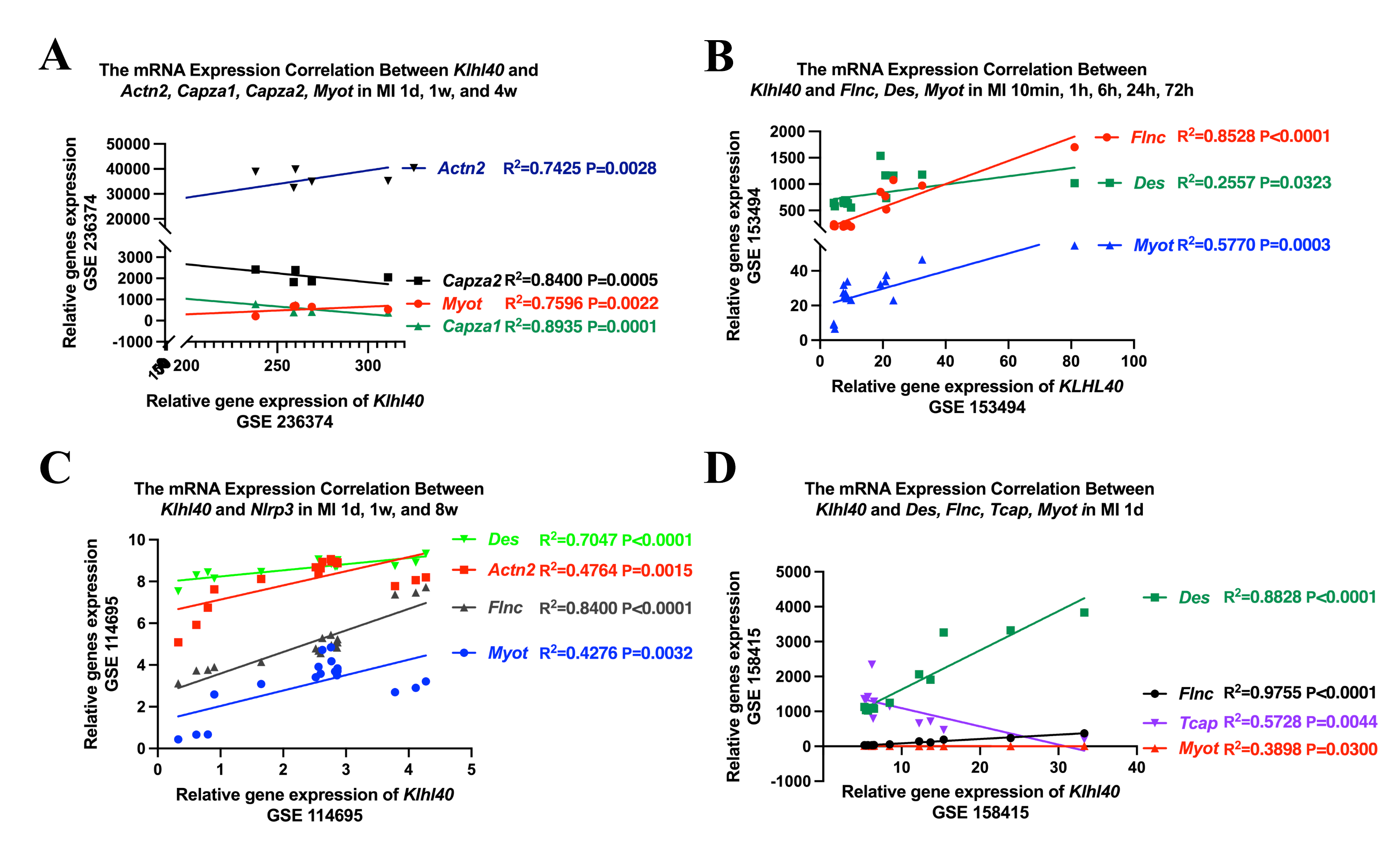

Supplement: Supplemental Information 6 — (A) GSE236374: Correlation analyses between Klhl40 and Actn2, Capza1, Capza2, and Myot. (B) GSE153494: Correlation analyses between Klhl40 and Flnc, Des, and Myot. (C) GSE114695: Correlation analyses between Klhl40 and Flnc, Des, Myot, and Actn2. (D) GSE158415: Correlation analyses between Klhl40 and Des, Flnc, Tcap, and Myot. [file peerj-14-21375-s006.png]

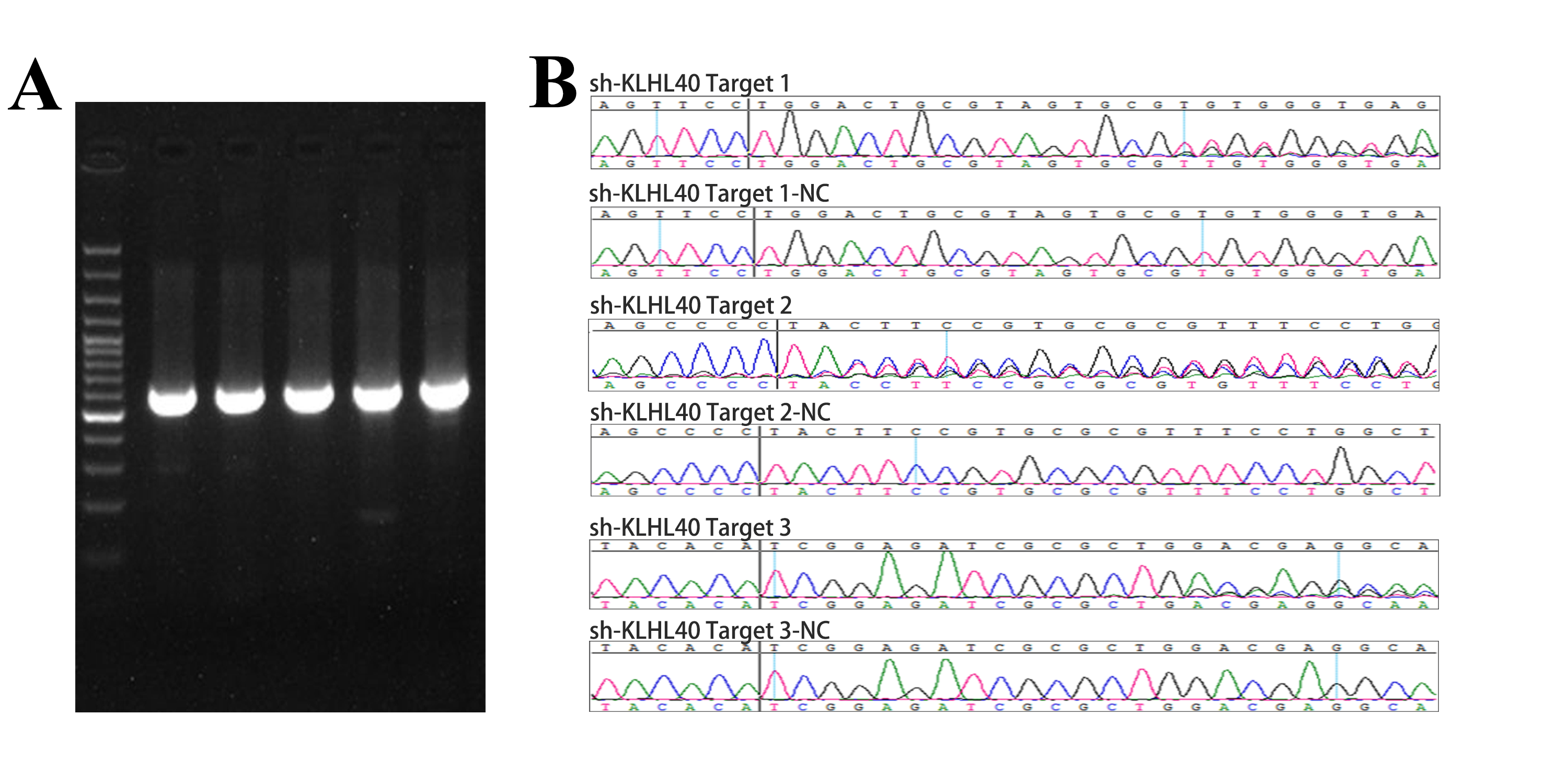

Supplement: Supplemental Information 7 — (A) Genetic identification of mouse genotype. (B) Three sh-KLHL40 sequences. [file peerj-14-21375-s007.png]

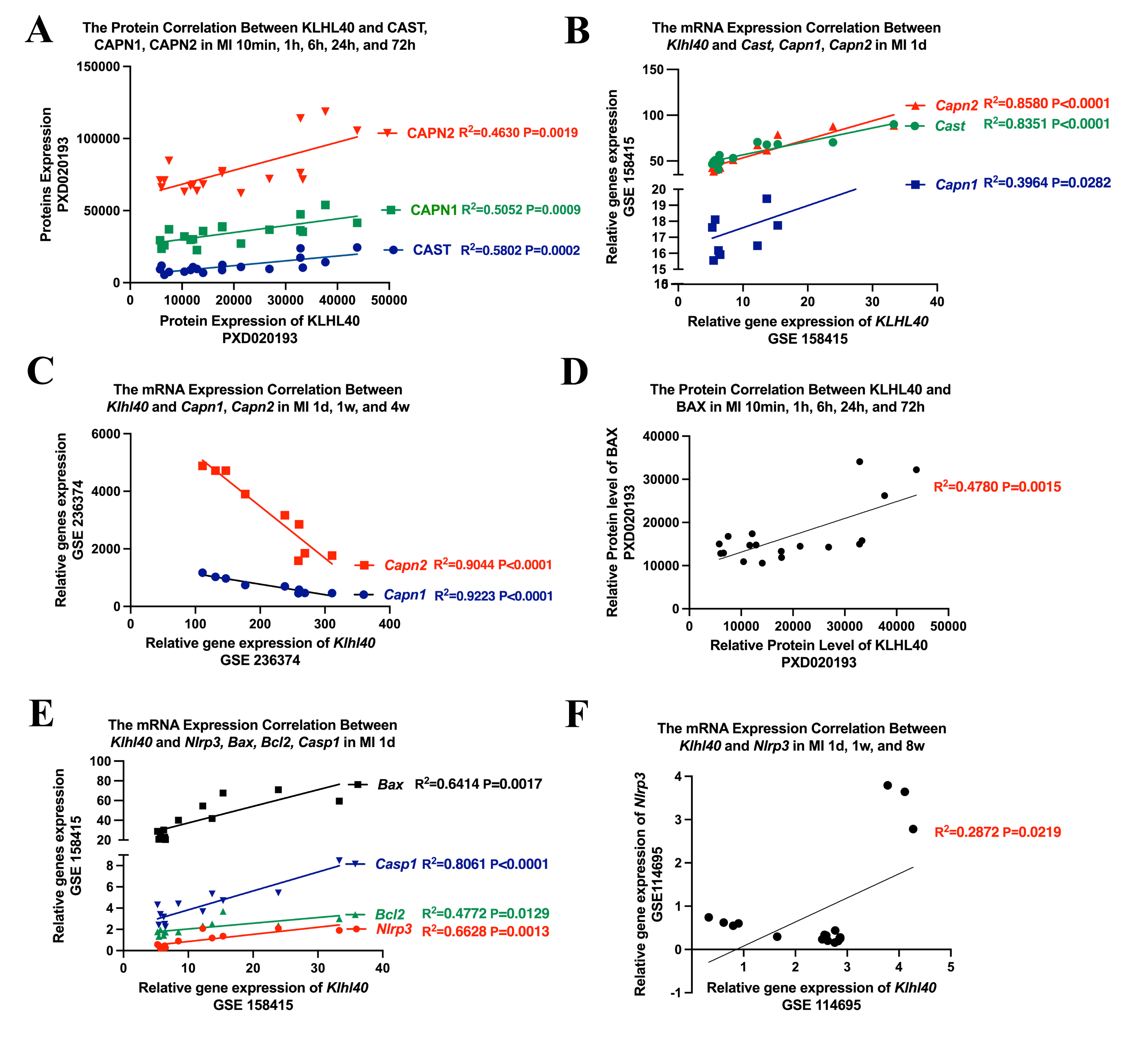

Supplement: Supplemental Information 8 — (A) PXD020193: Correlation analyses between KLHL40 and CAST, CAPN1, and CAPN2. (B) GSE158415: Correlation analyses between Klhl40 and Cast, Capn1, and Capn2. (C) GSE236374: Correlation analyses between Klhl40 and Capn1 and Capn2. (D) PXD020193: Correlation analyses between KLHL40 and BAX. (E) GSE158415: Correlation analyses between Klhl40 and Nlrp3, Bax, Bcl2, and Casp1. (F) GSE114695: Correlation analyses between Klhl40 and Nlrp3. [file peerj-14-21375-s008.png]

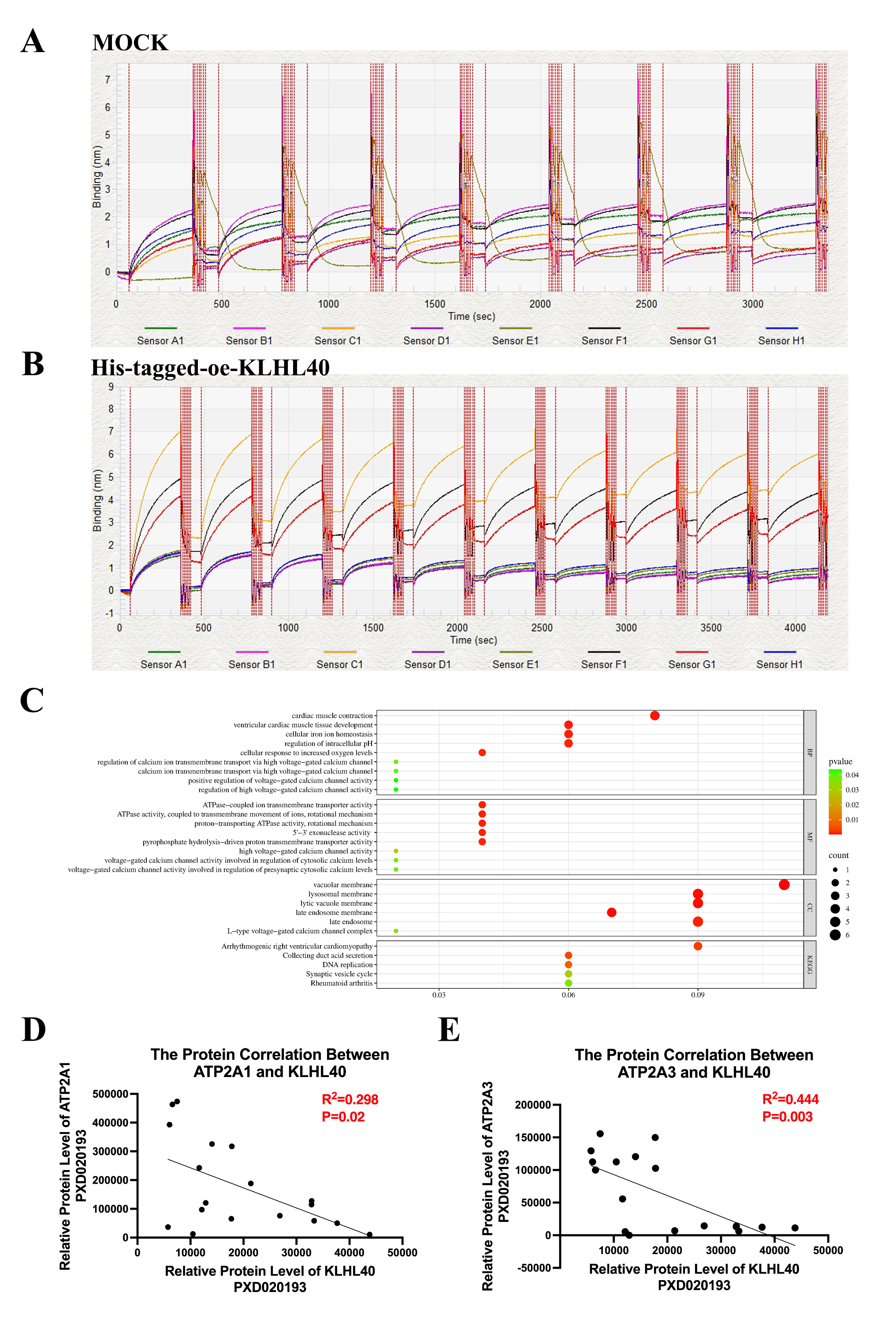

Supplement: Supplemental Information 9 — (A) Protein captured by the NTA sensor in the Mock group. (B) Protein captured by the NTA sensor in the His-tagged-oe-KLHL40 group. (C) Proteomic enrichment analysis performed using https://www.bioinformatics.com.cn/. (D) PXD020193 dataset analysis showed a s catter plot analysis of the c orrelation between ATP2A1 and KLHL40 (R2 = 0.2984, P = 0.0190). (E) PXD020193 dataset analysis showed a s catter plot analysis of the c orrelation between ATP2A3 and KLHL40 (R2 = 0.444, P = 0.0025). [file peerj-14-21375-s009.png]

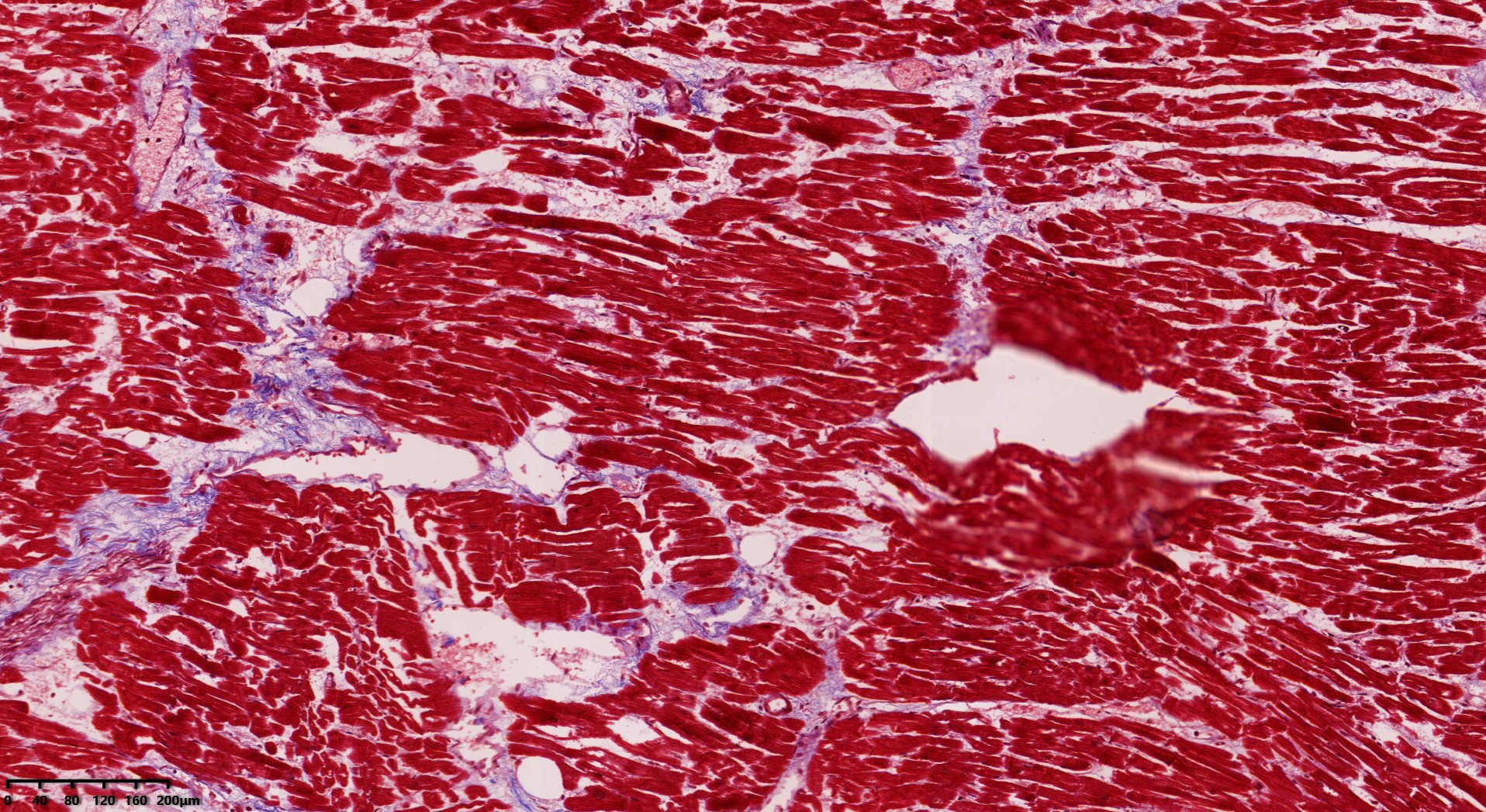

Supplement: Supplemental Information 10 [file peerj-14-21375-s010.zip › IHC in figure/Early of MI Border zone 200a╠m Masson.jpg]

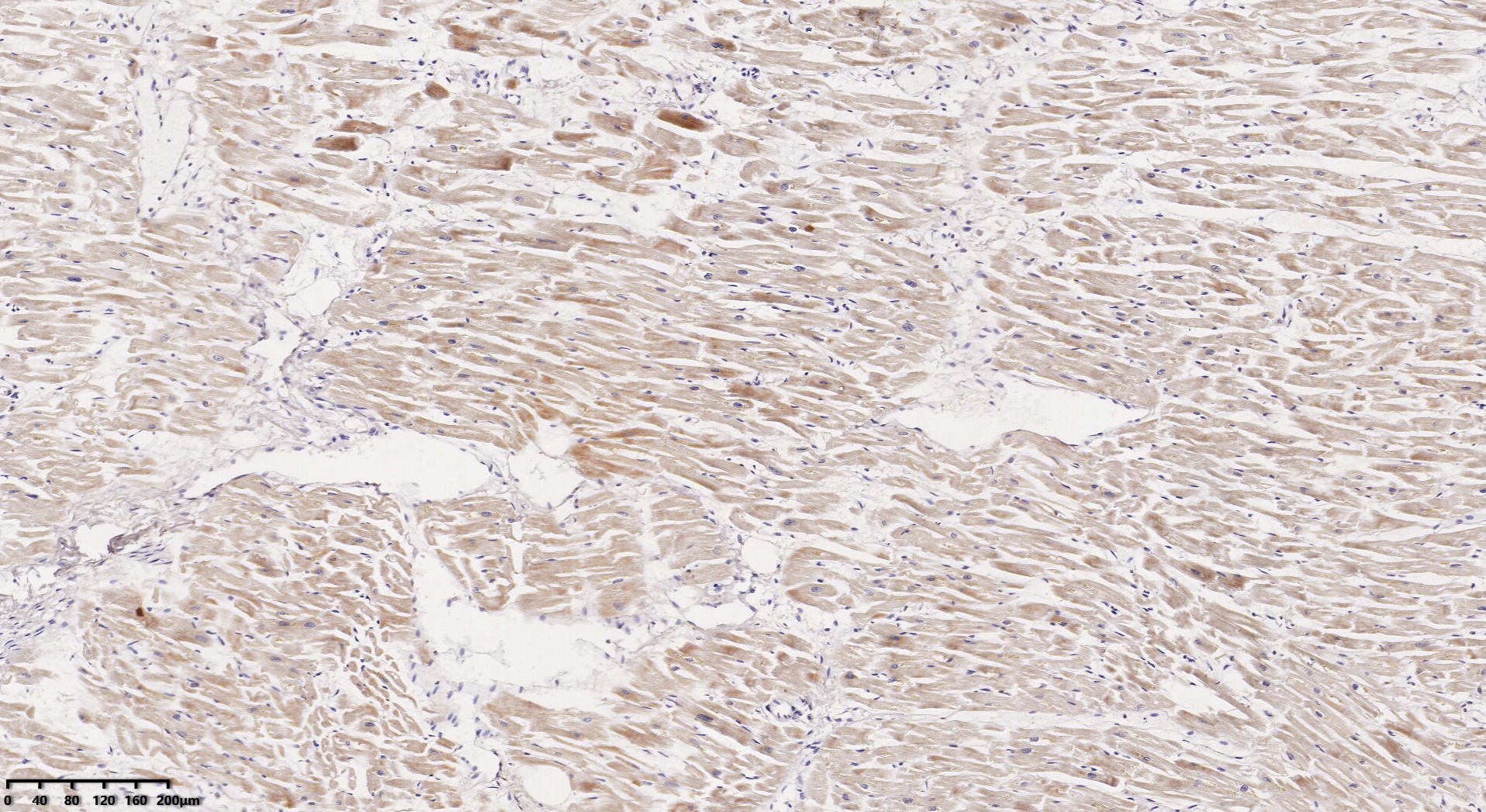

Supplement: Supplemental Information 10 [file peerj-14-21375-s010.zip › IHC in figure/Early of MI Border zone IHC 200a╠m.jpg]

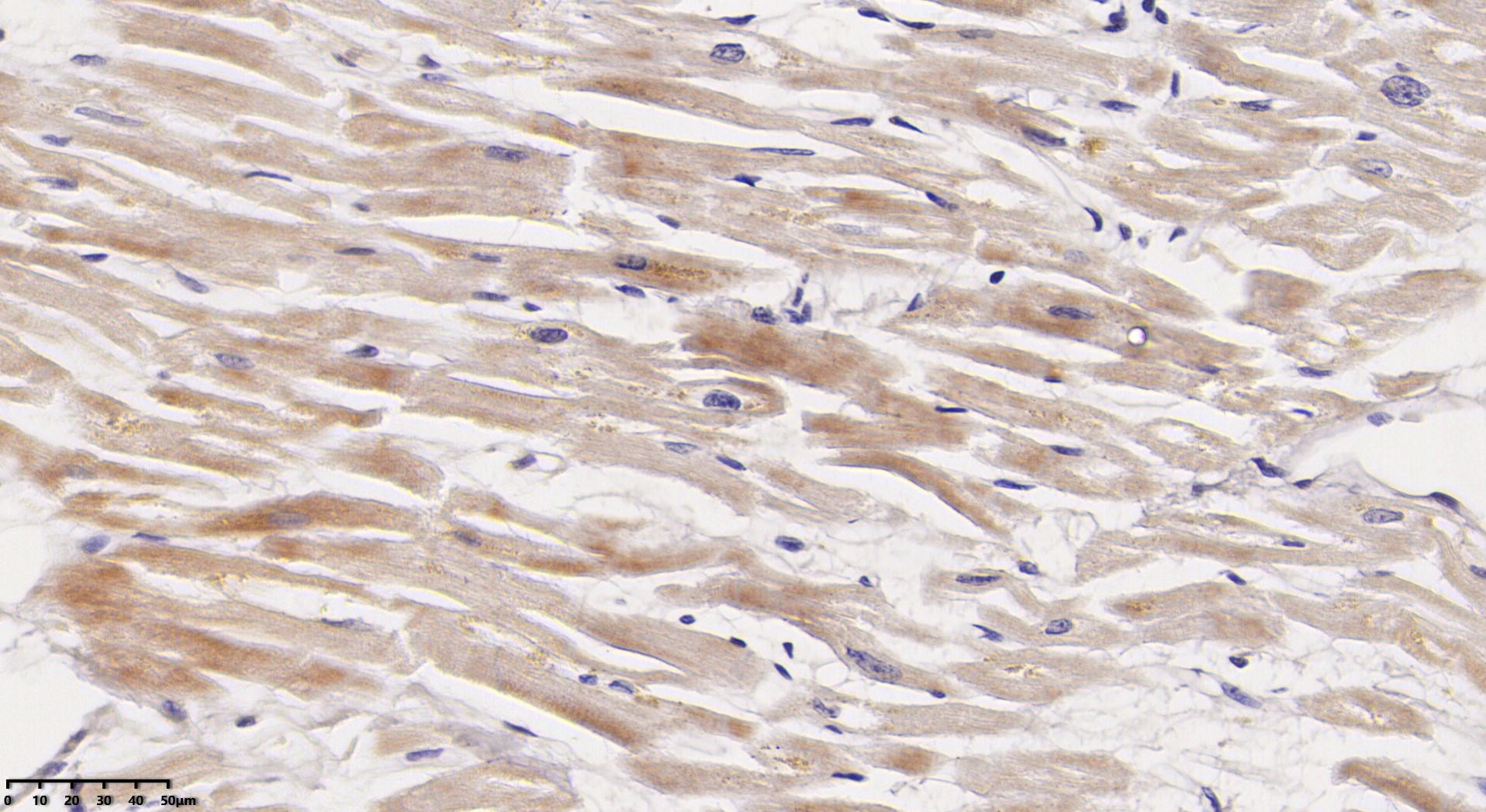

Supplement: Supplemental Information 10 [file peerj-14-21375-s010.zip › IHC in figure/Early of MI Border zone IHC 50a╠m.jpg]

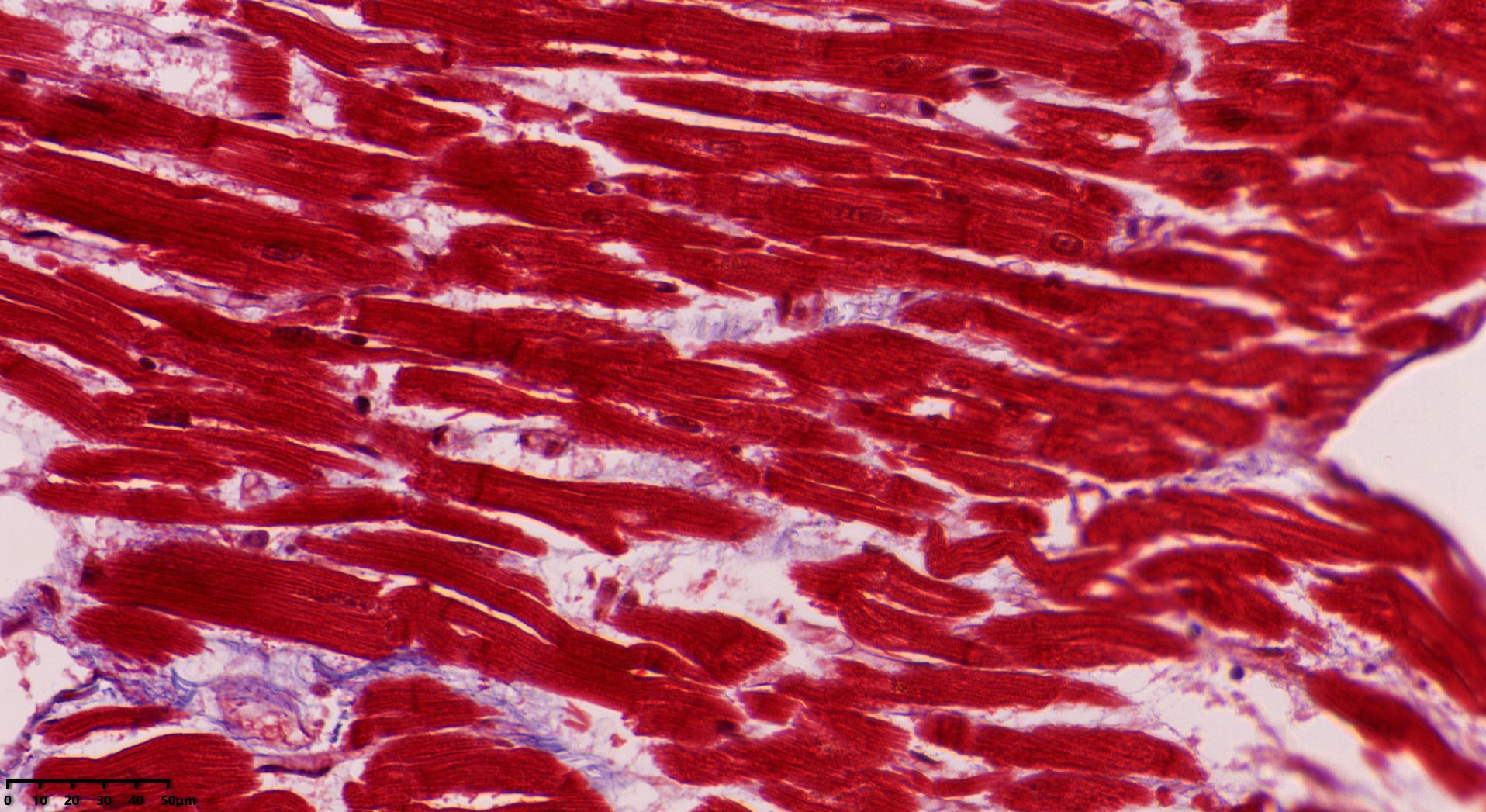

Supplement: Supplemental Information 10 [file peerj-14-21375-s010.zip › IHC in figure/Early of MI Border zone Masson 50a╠m.jpg]

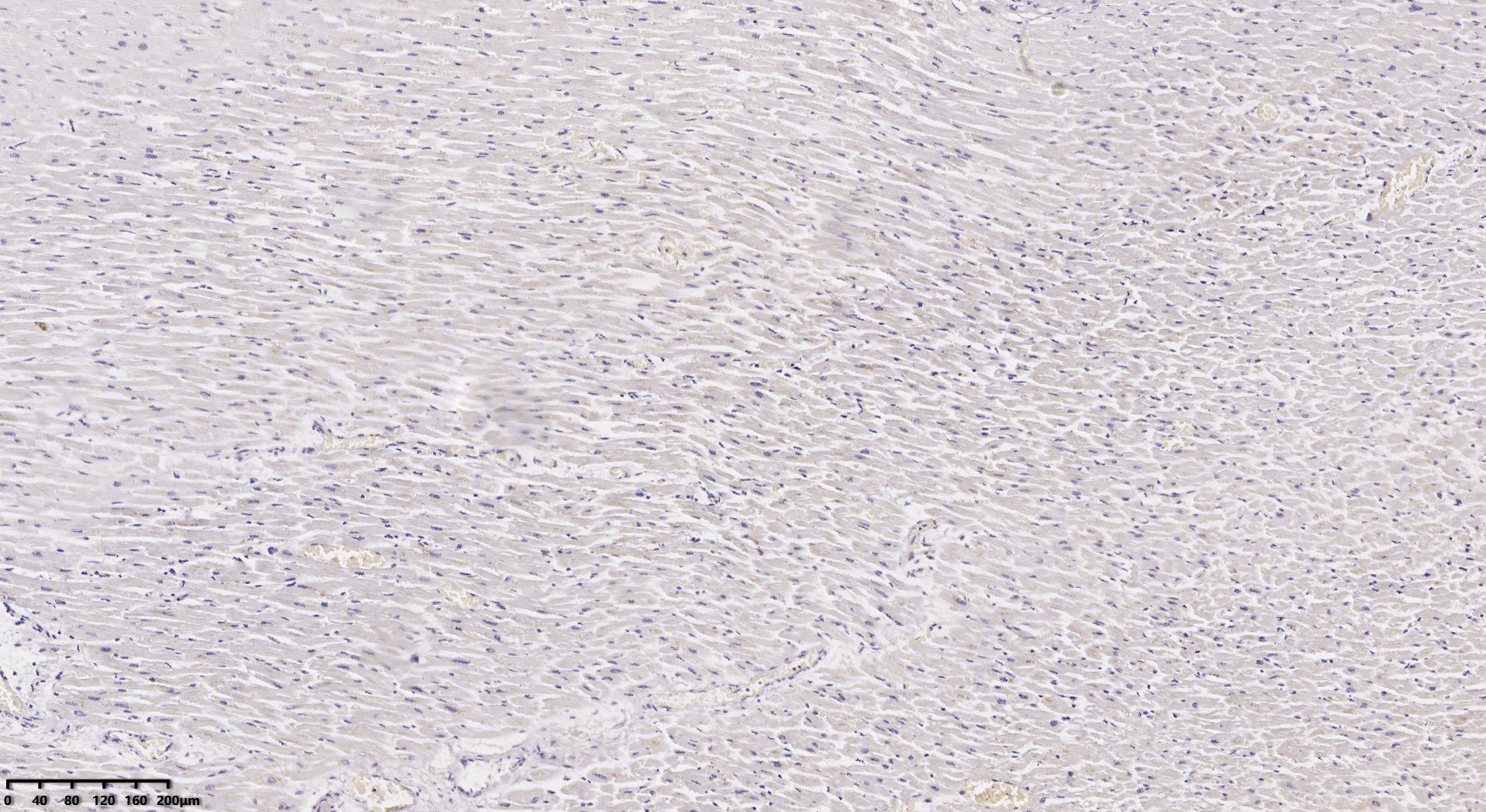

Supplement: Supplemental Information 10 [file peerj-14-21375-s010.zip › IHC in figure/Normal IHC 200a╠m.jpg]

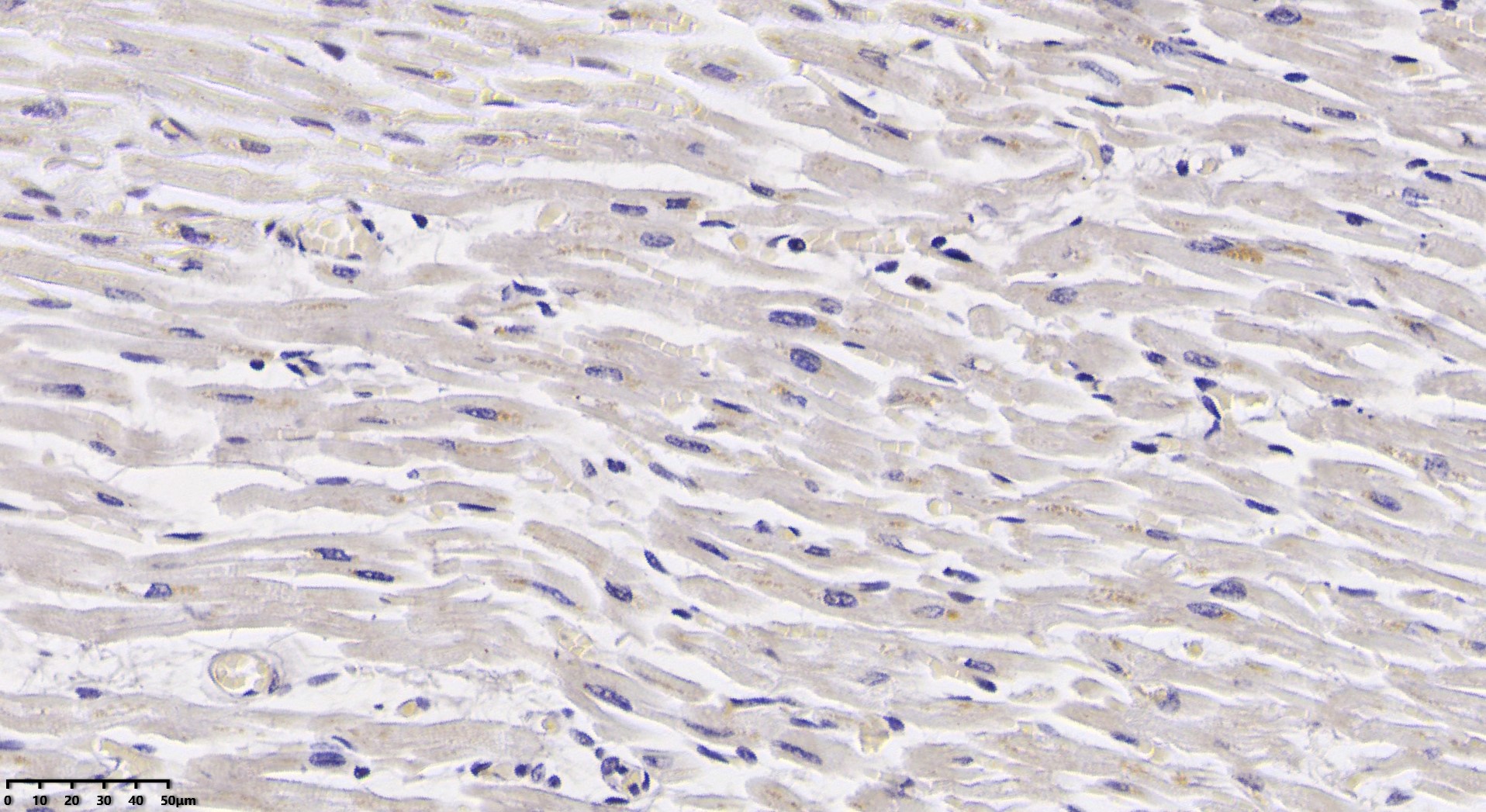

Supplement: Supplemental Information 10 [file peerj-14-21375-s010.zip › IHC in figure/Normal IHC 50a╠m.jpg]

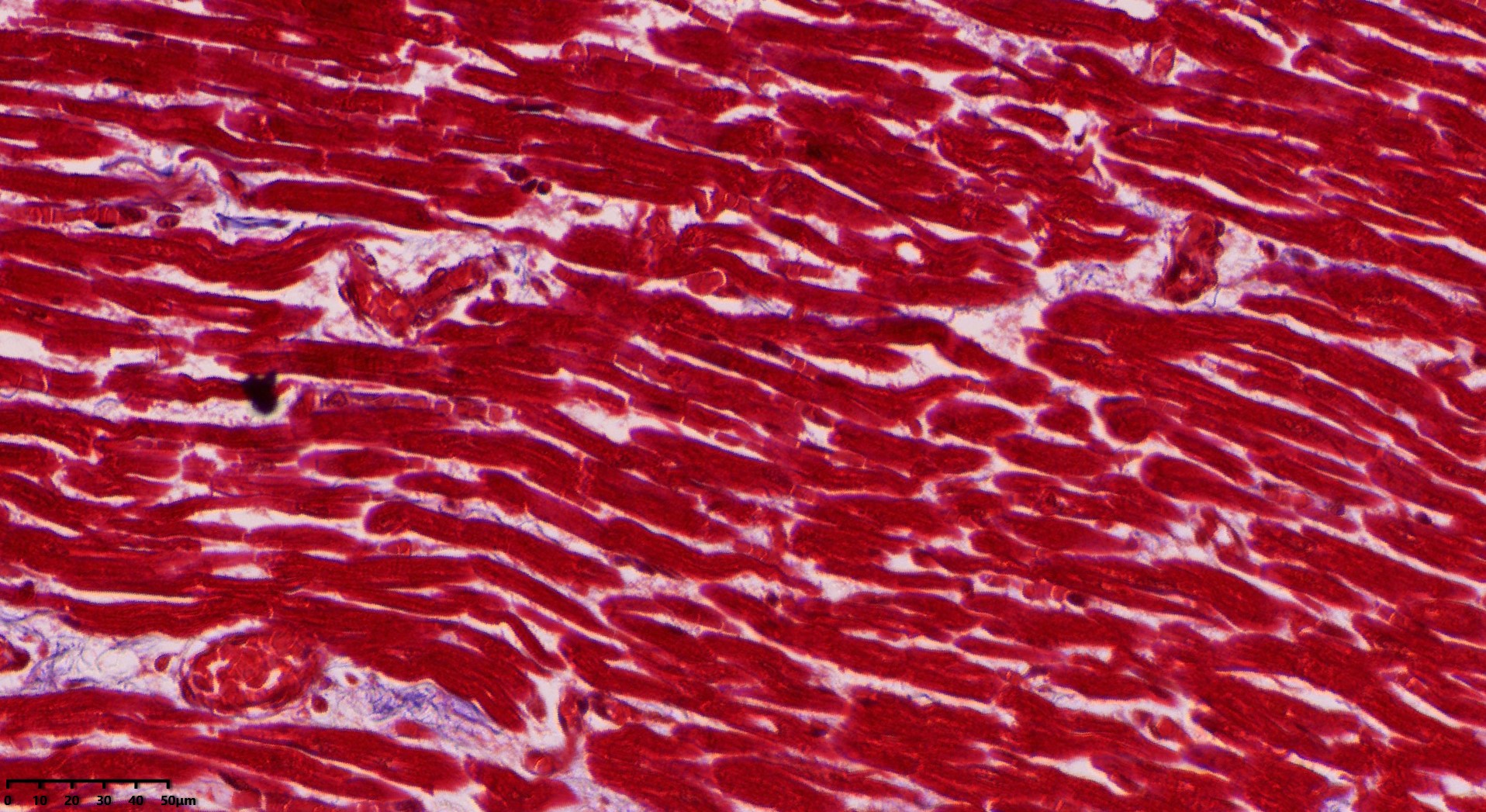

Supplement: Supplemental Information 10 [file peerj-14-21375-s010.zip › IHC in figure/Normal Masson 50a╠m.jpg]

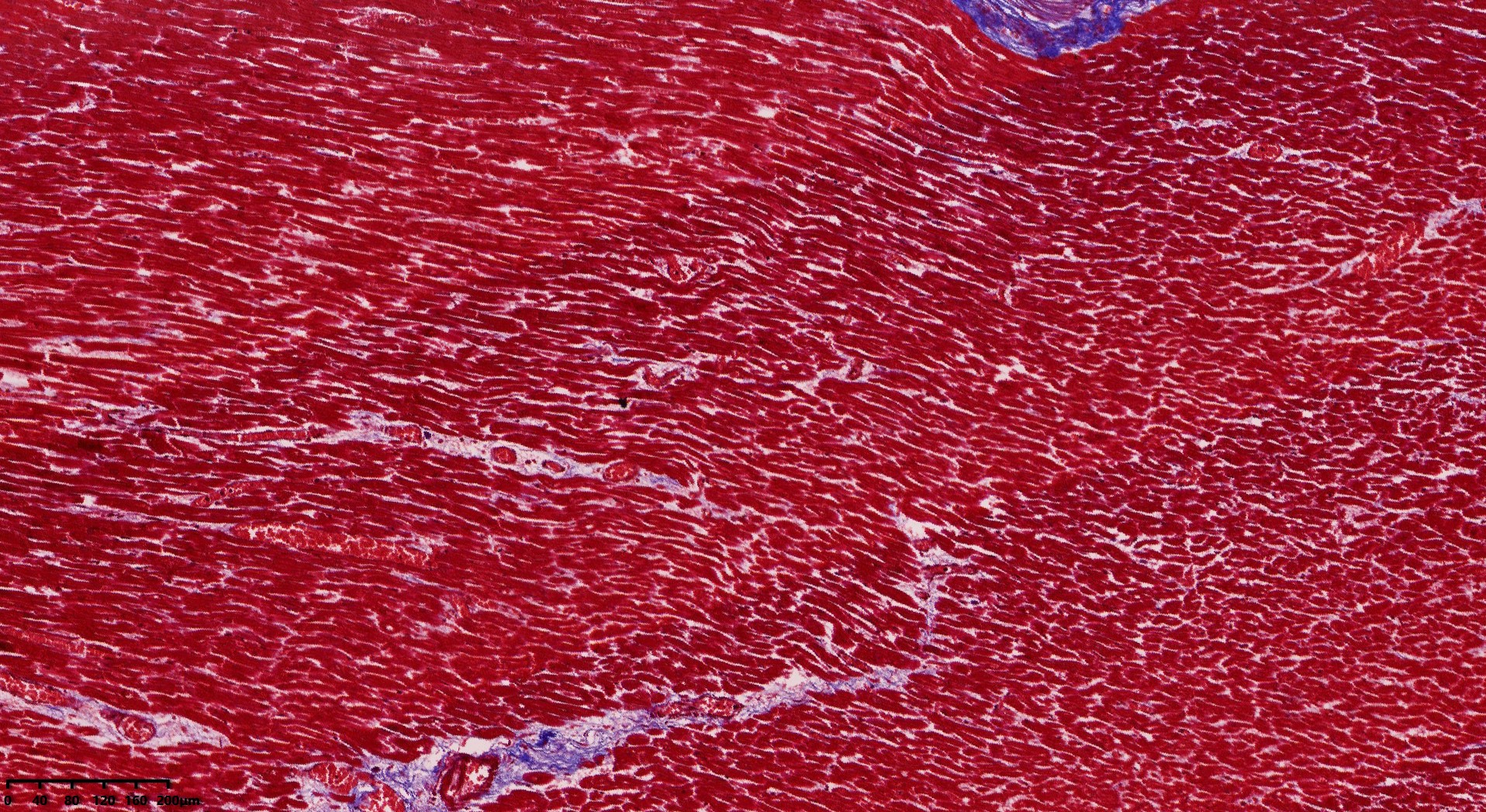

Supplement: Supplemental Information 10 [file peerj-14-21375-s010.zip › IHC in figure/normal-Masson 200a╠m.jpg]

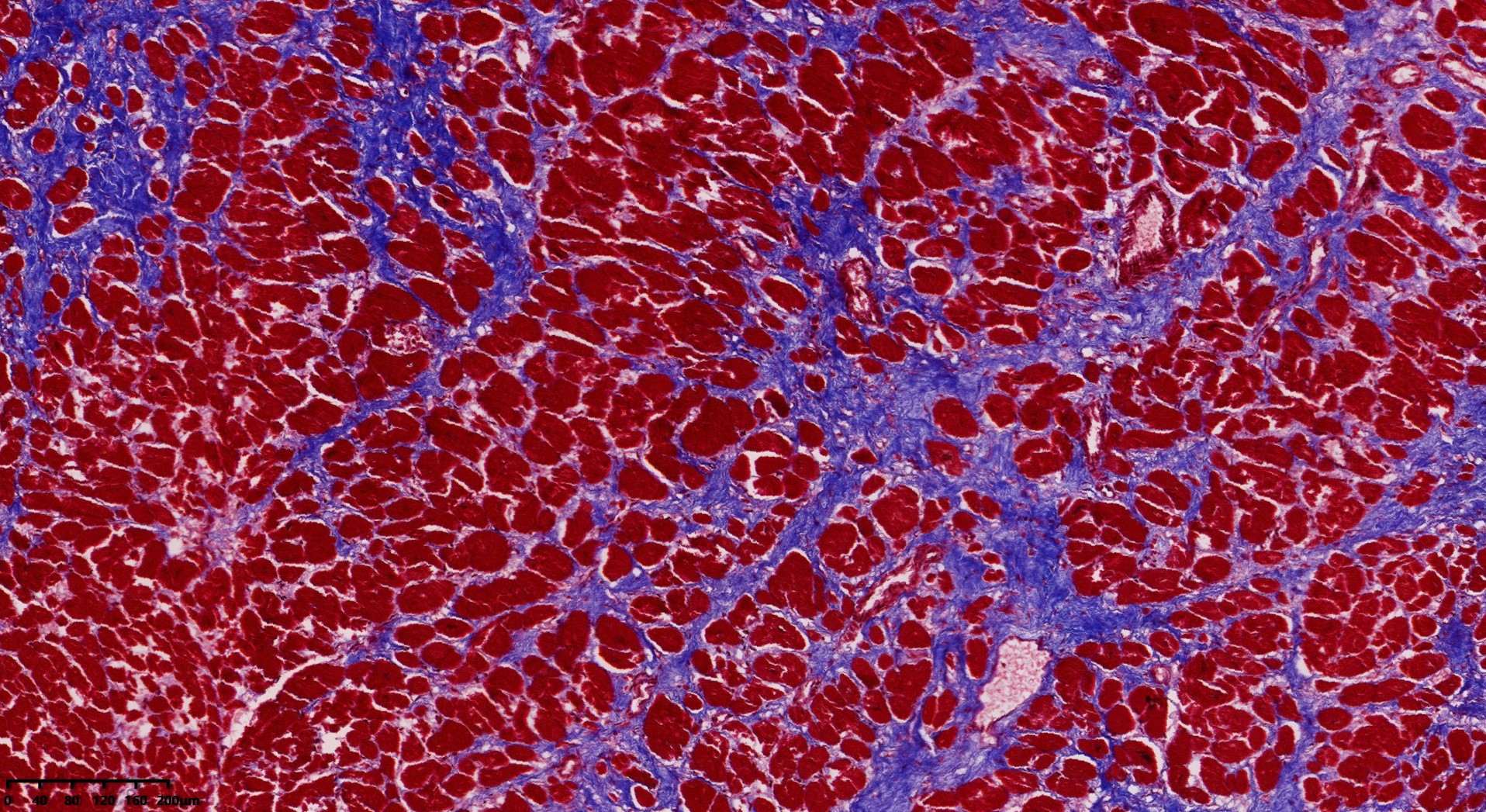

Supplement: Supplemental Information 10 [file peerj-14-21375-s010.zip › IHC in figure/Per-Scar 200a╠m Masson.jpg]

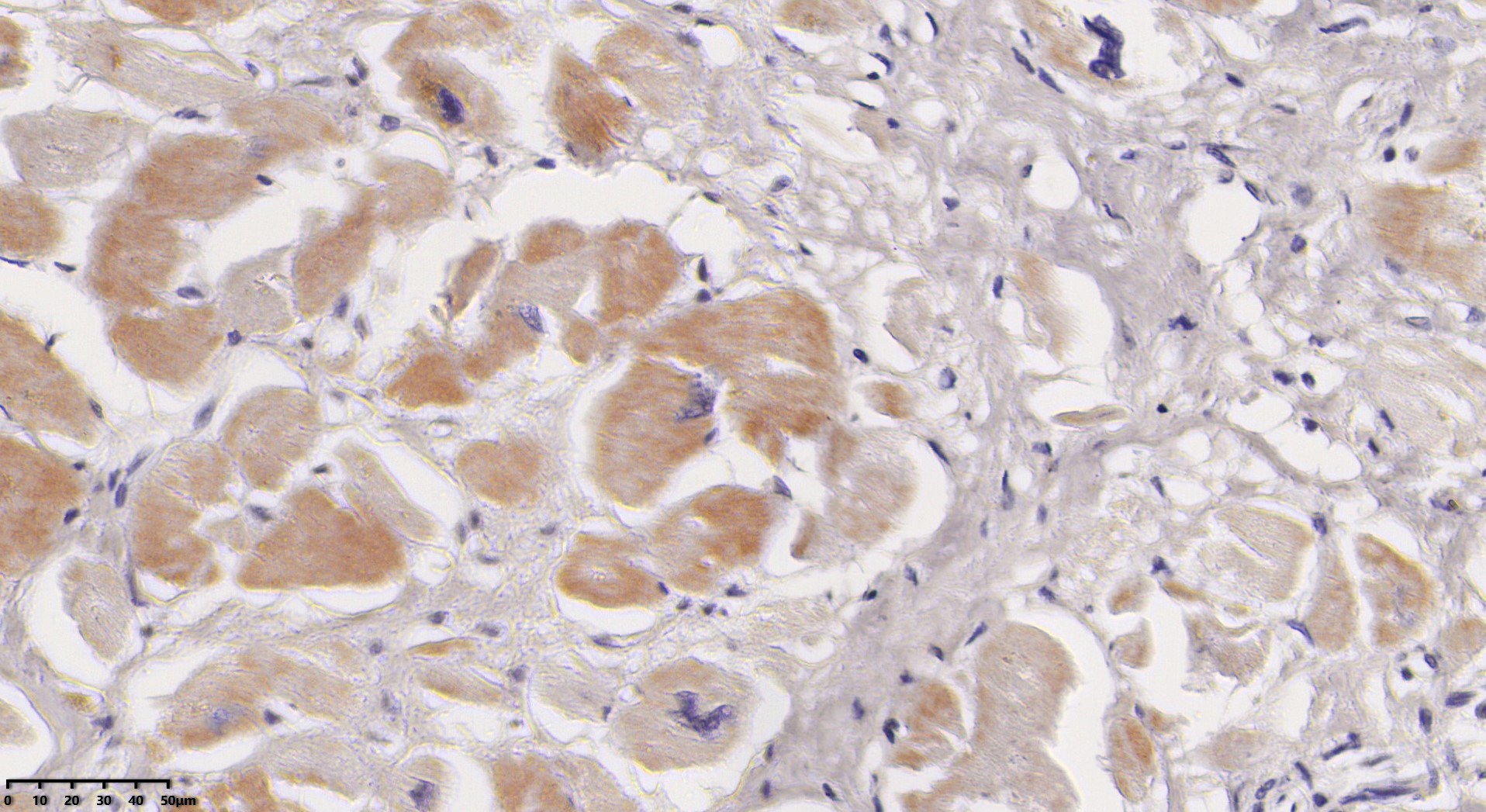

Supplement: Supplemental Information 10 [file peerj-14-21375-s010.zip › IHC in figure/Per-Scar IHC 50a╠m.jpg]

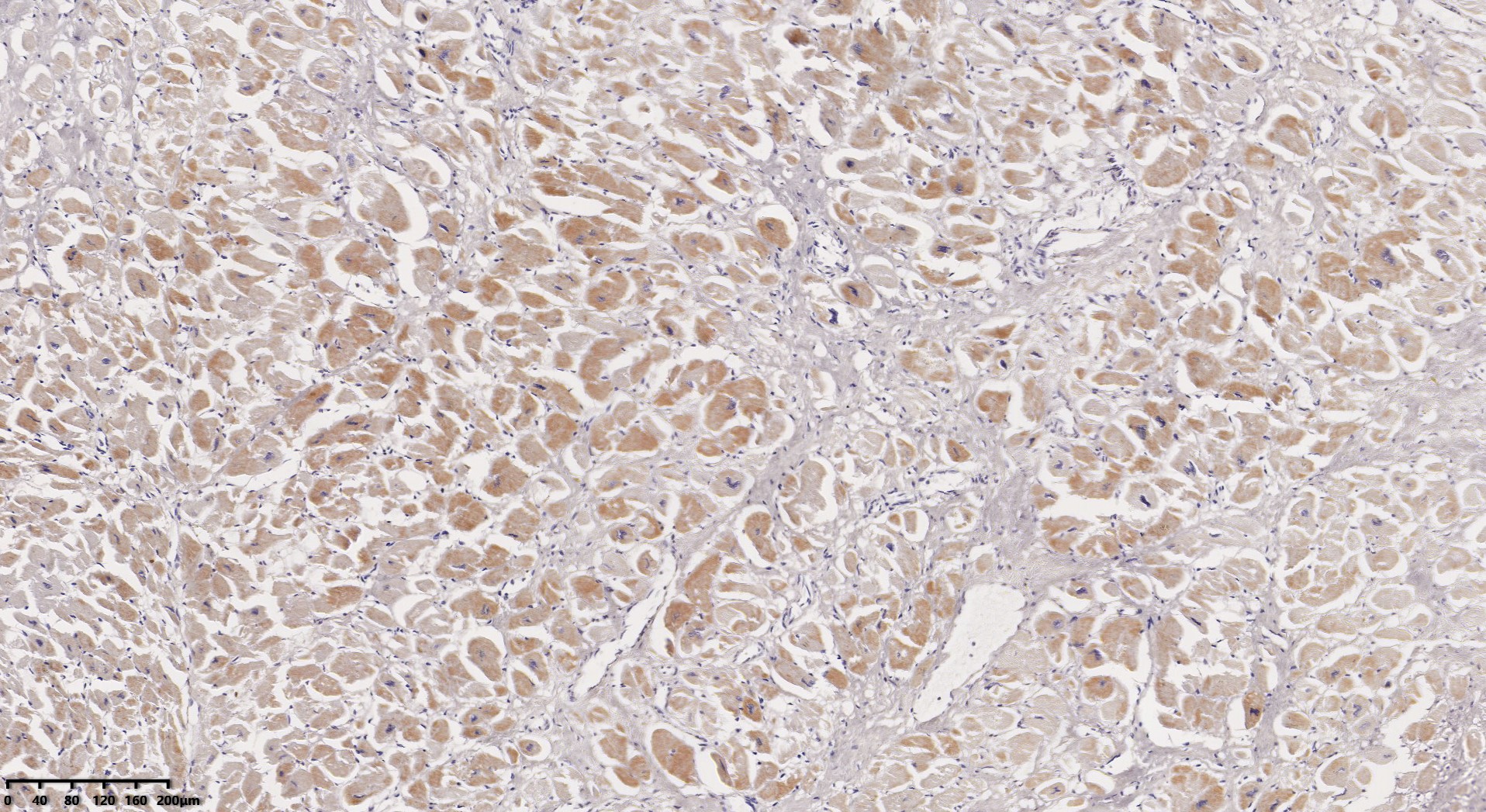

Supplement: Supplemental Information 10 [file peerj-14-21375-s010.zip › IHC in figure/Per-Scar IHC IHC 200a╠m.jpg]

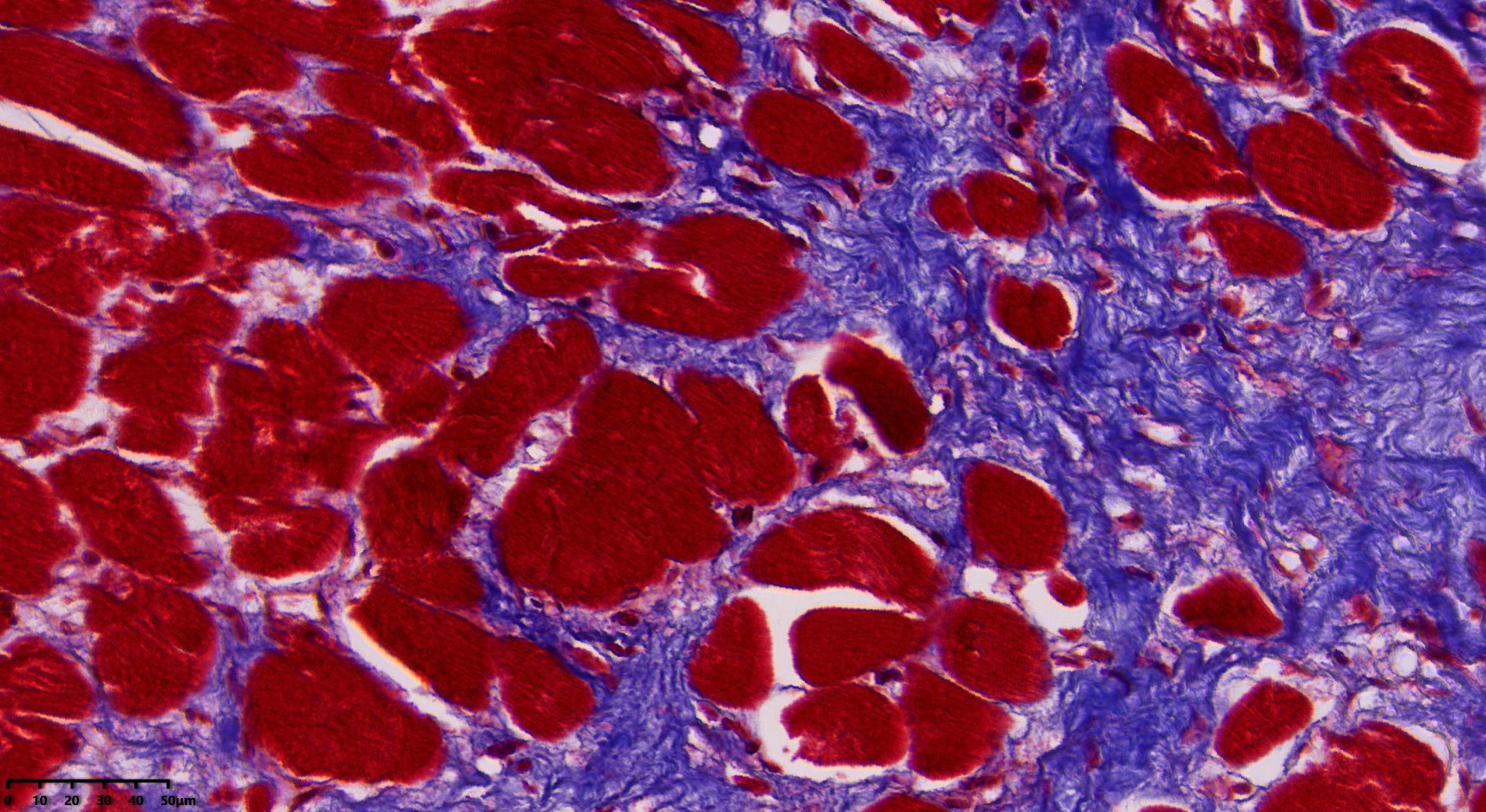

Supplement: Supplemental Information 10 [file peerj-14-21375-s010.zip › IHC in figure/Per-Scar Masson 50a╠m.jpg]

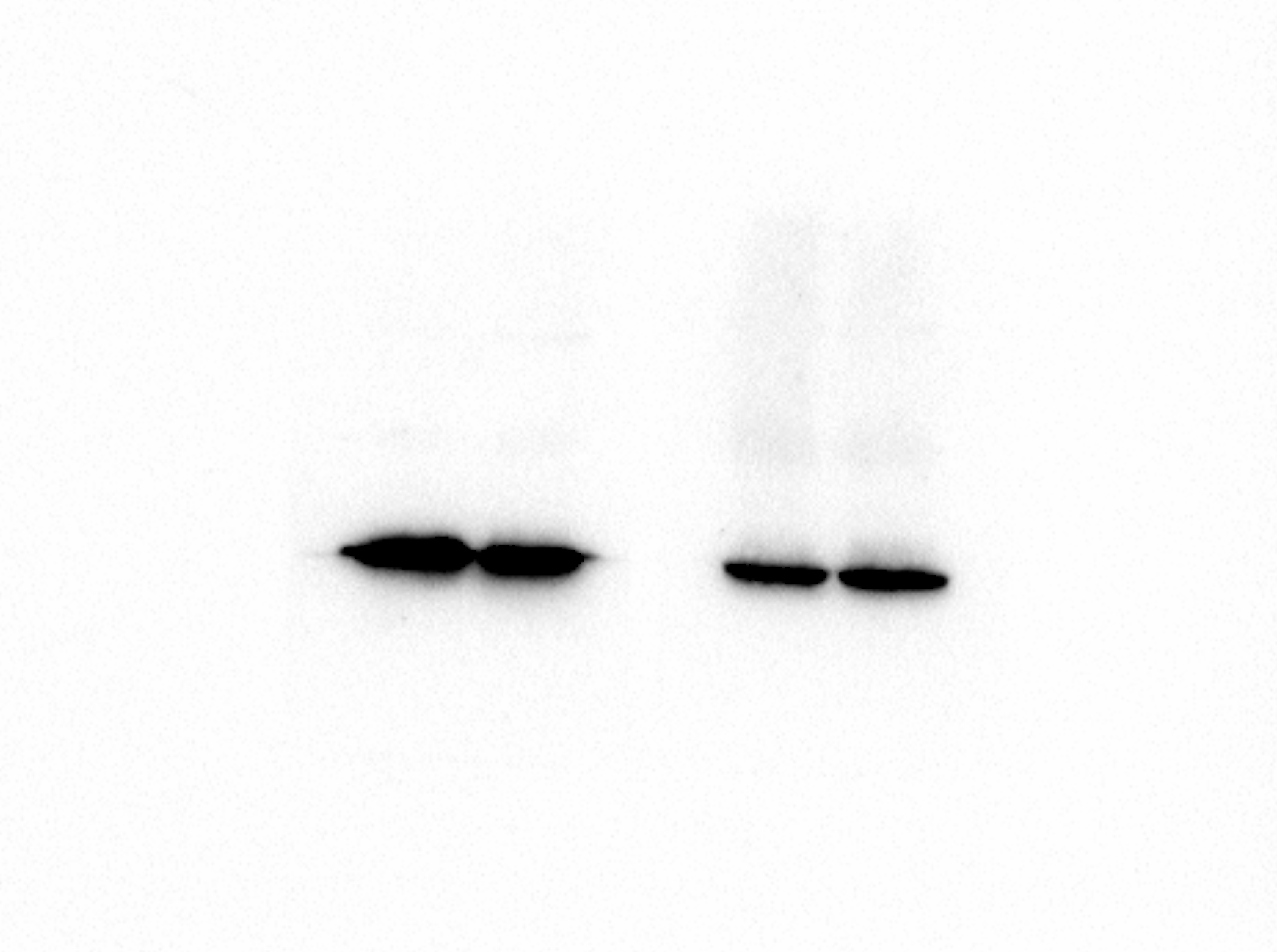

Supplement: Supplemental Information 18 [file peerj-14-21375-s018.zip › Figure 2I WB RAW Early KLHL40/KLHL40-1 GAPDH.tif]

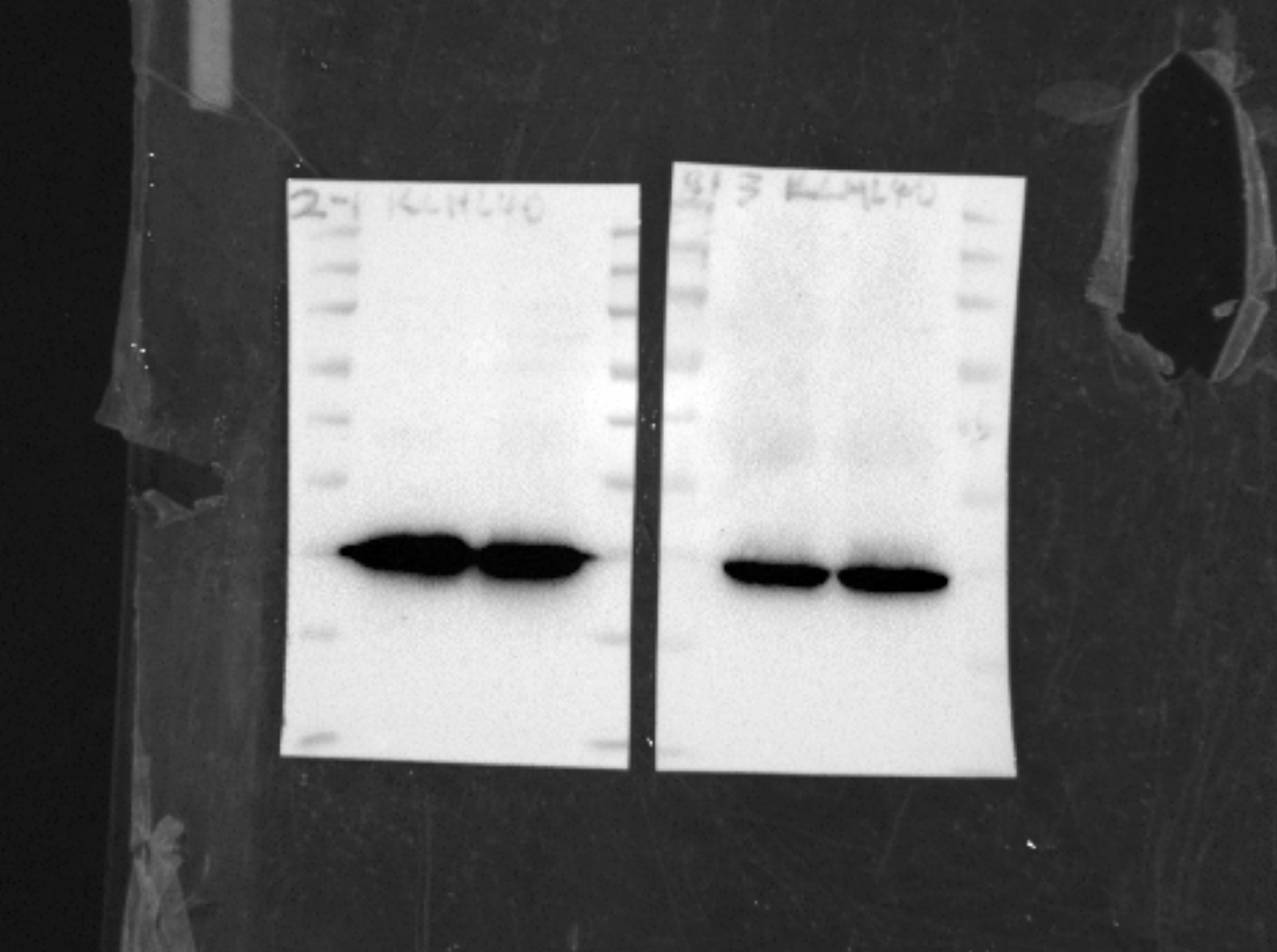

Supplement: Supplemental Information 18 [file peerj-14-21375-s018.zip › Figure 2I WB RAW Early KLHL40/KLHL40-1 GAPDH+MARK.tif]

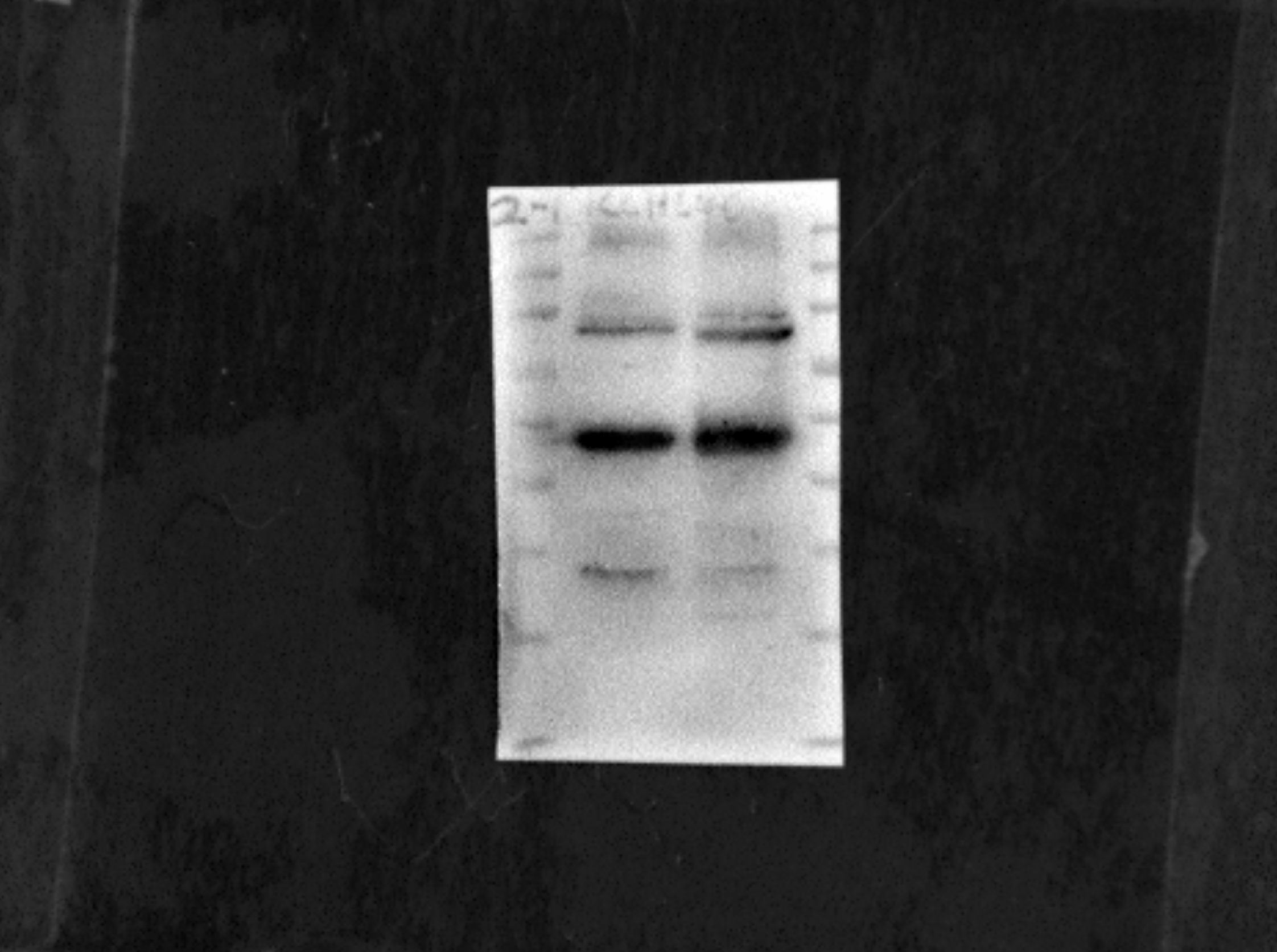

Supplement: Supplemental Information 18 [file peerj-14-21375-s018.zip › Figure 2I WB RAW Early KLHL40/klhl40-1 MARK.tif]

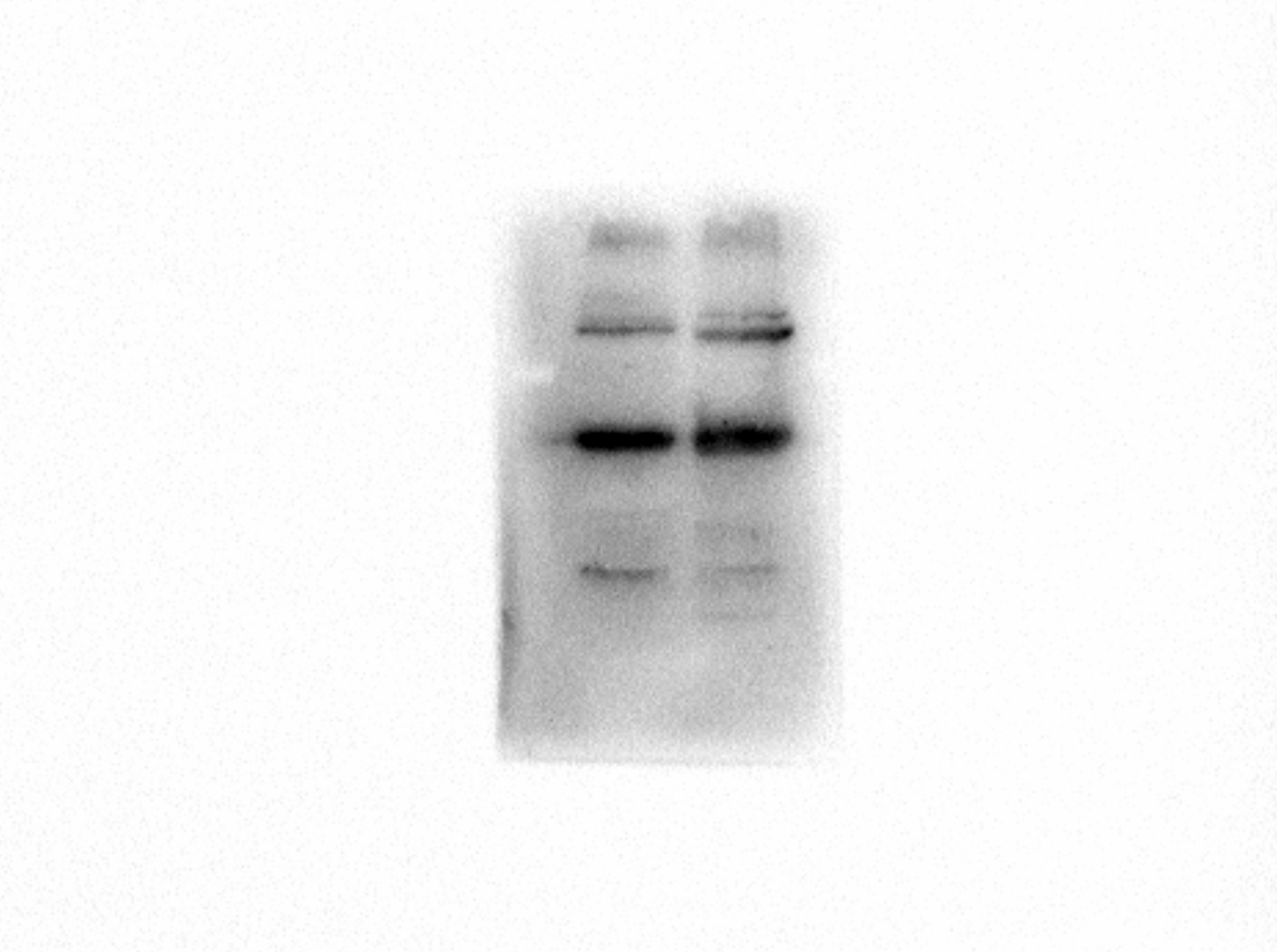

Supplement: Supplemental Information 18 [file peerj-14-21375-s018.zip › Figure 2I WB RAW Early KLHL40/klhl40-1.tif]

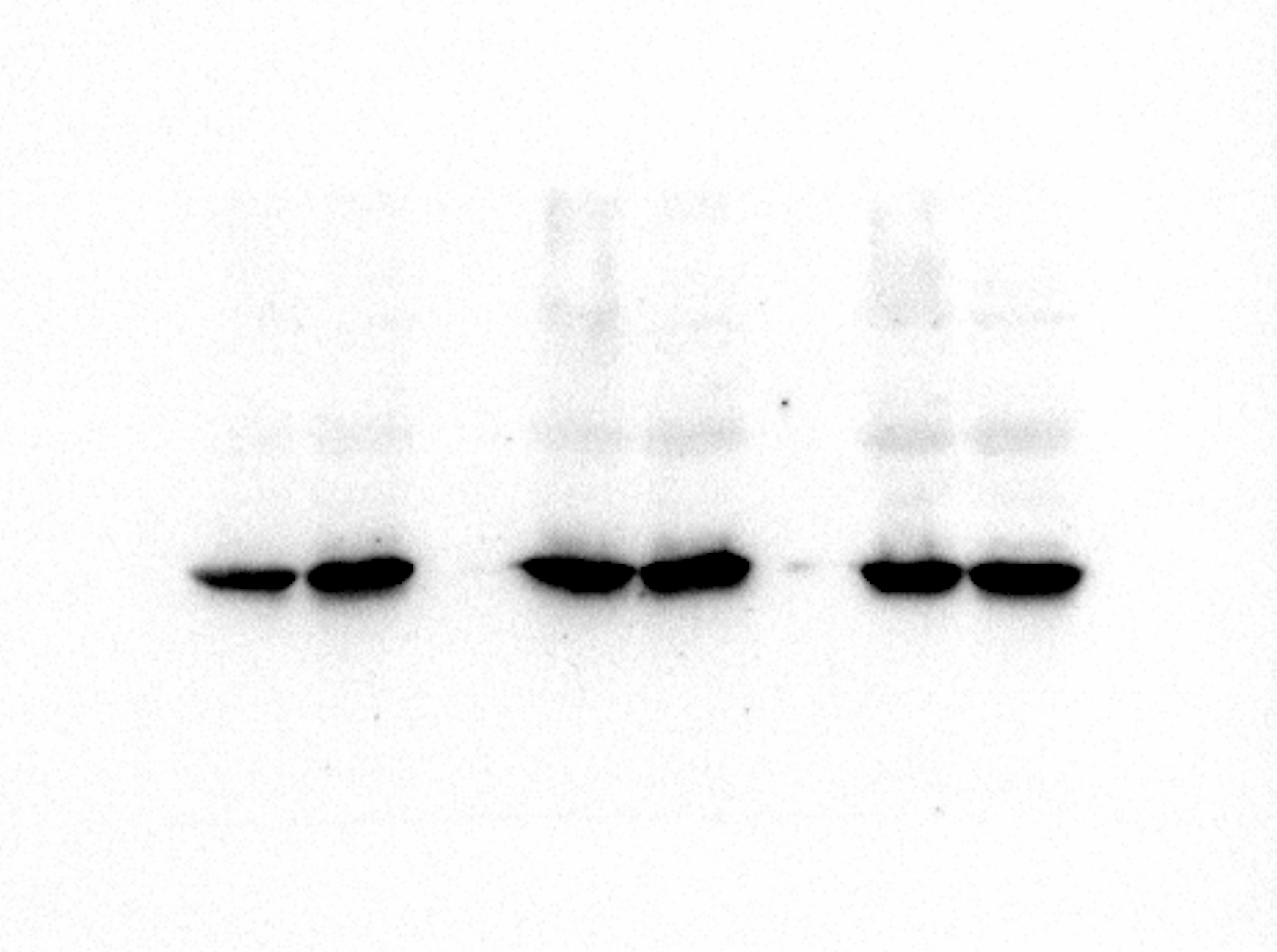

Supplement: Supplemental Information 18 [file peerj-14-21375-s018.zip › Figure 2I WB RAW Early KLHL40/KLHL40-2-3 GAPDH.tif]

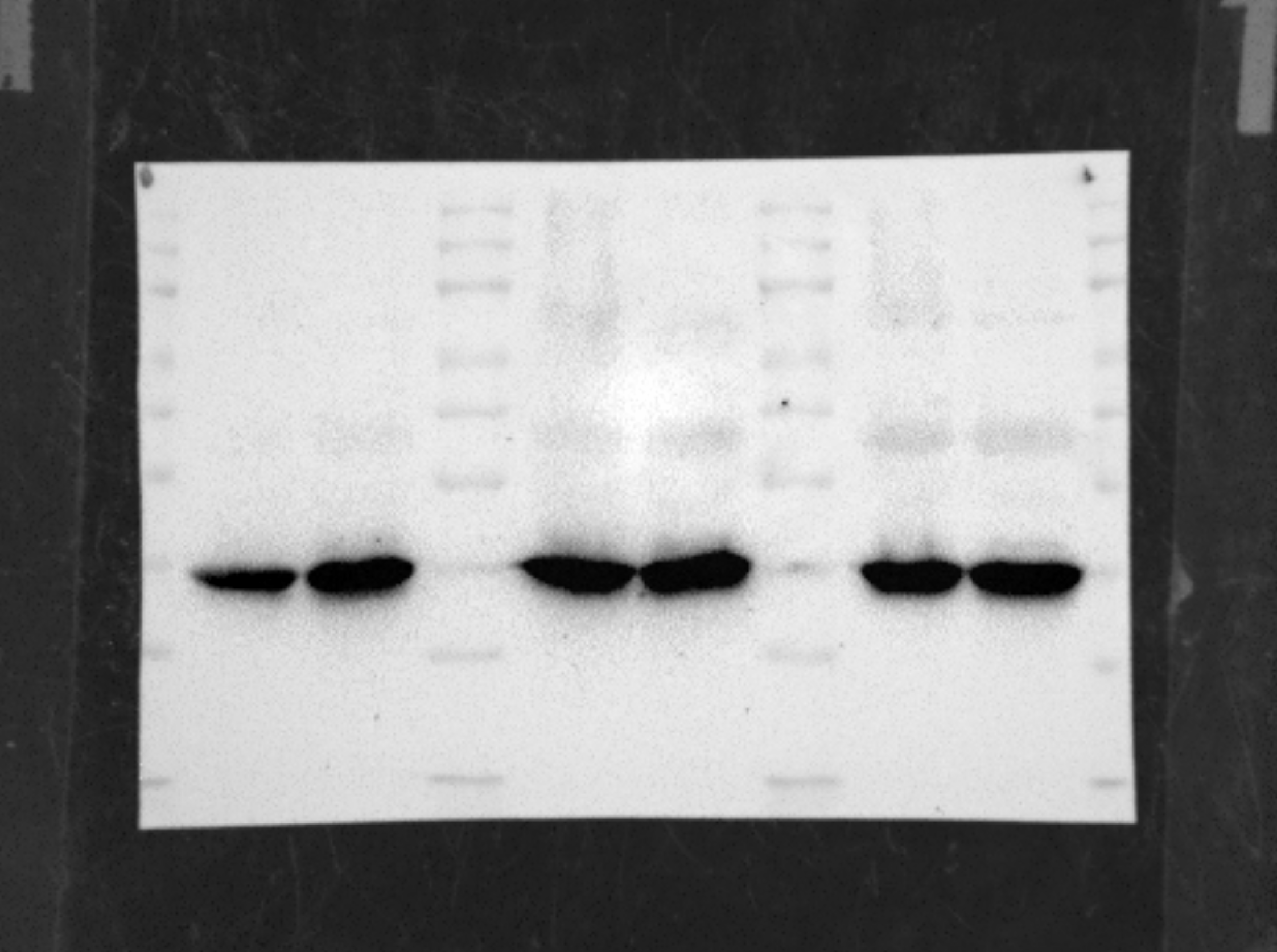

Supplement: Supplemental Information 18 [file peerj-14-21375-s018.zip › Figure 2I WB RAW Early KLHL40/KLHL40-2-3 GAPDH+MARK.tif]

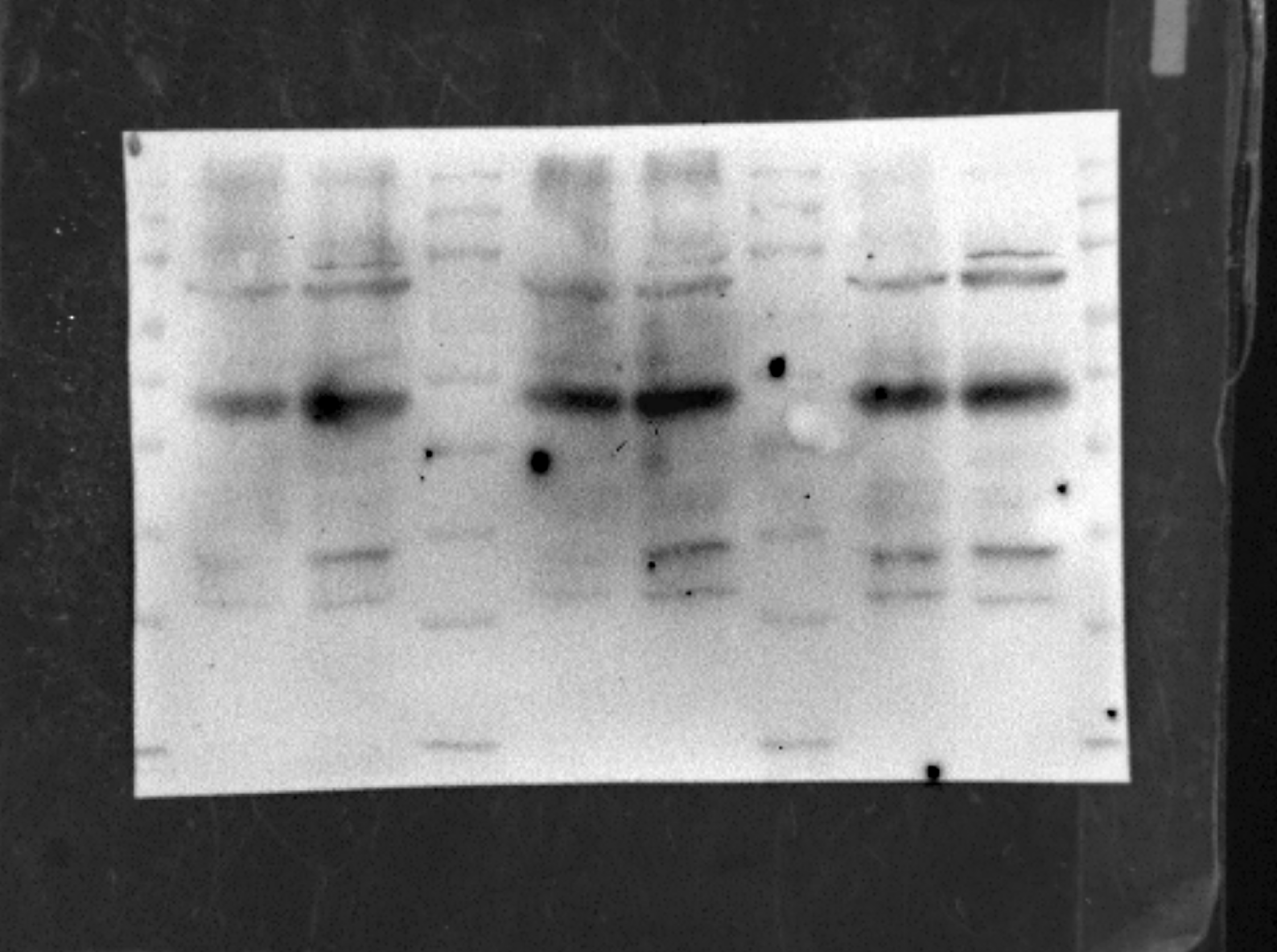

Supplement: Supplemental Information 18 [file peerj-14-21375-s018.zip › Figure 2I WB RAW Early KLHL40/KLHL40-2-3 MARK.tif]

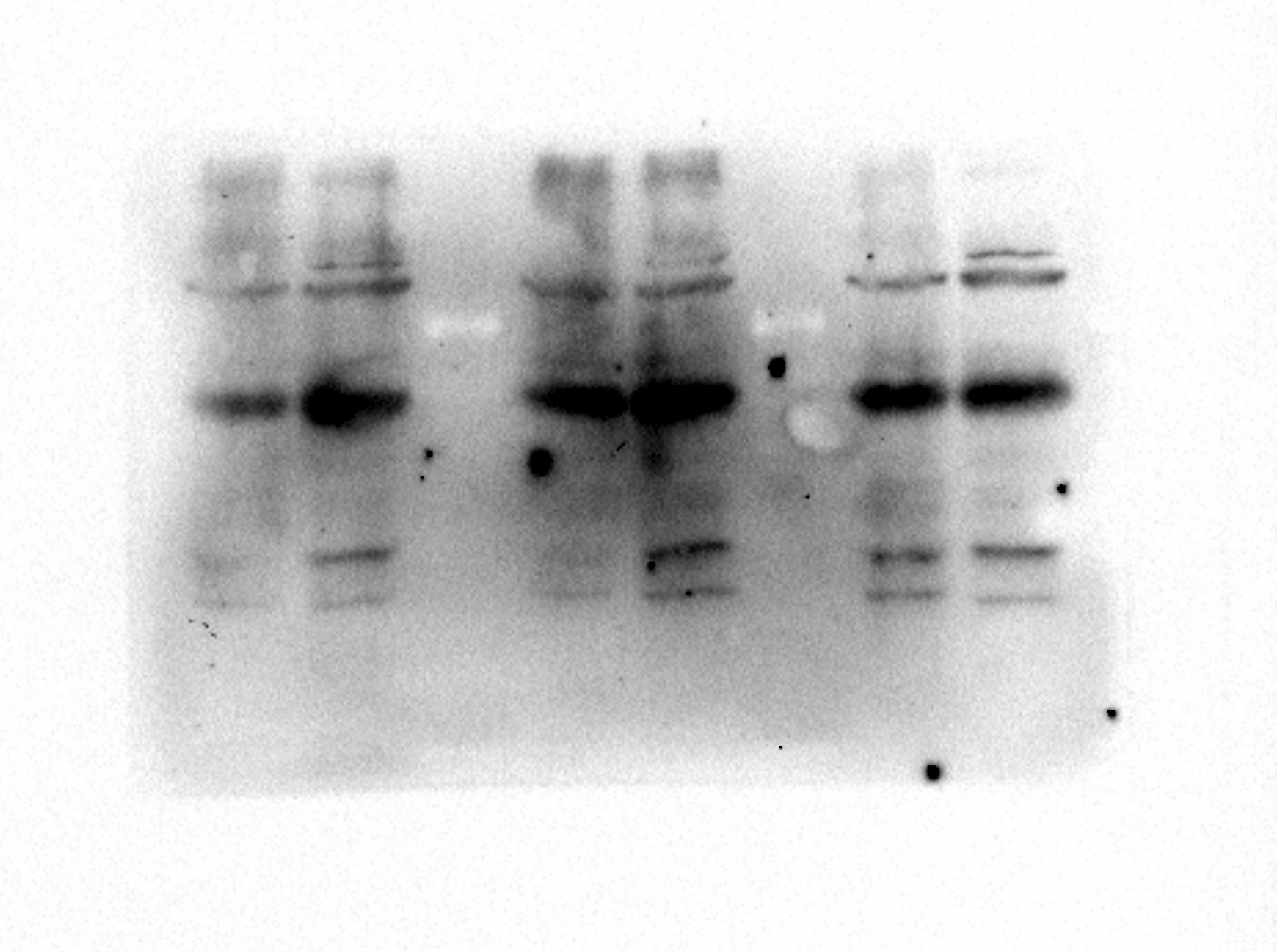

Supplement: Supplemental Information 18 [file peerj-14-21375-s018.zip › Figure 2I WB RAW Early KLHL40/KLHL40-2-3.tif]

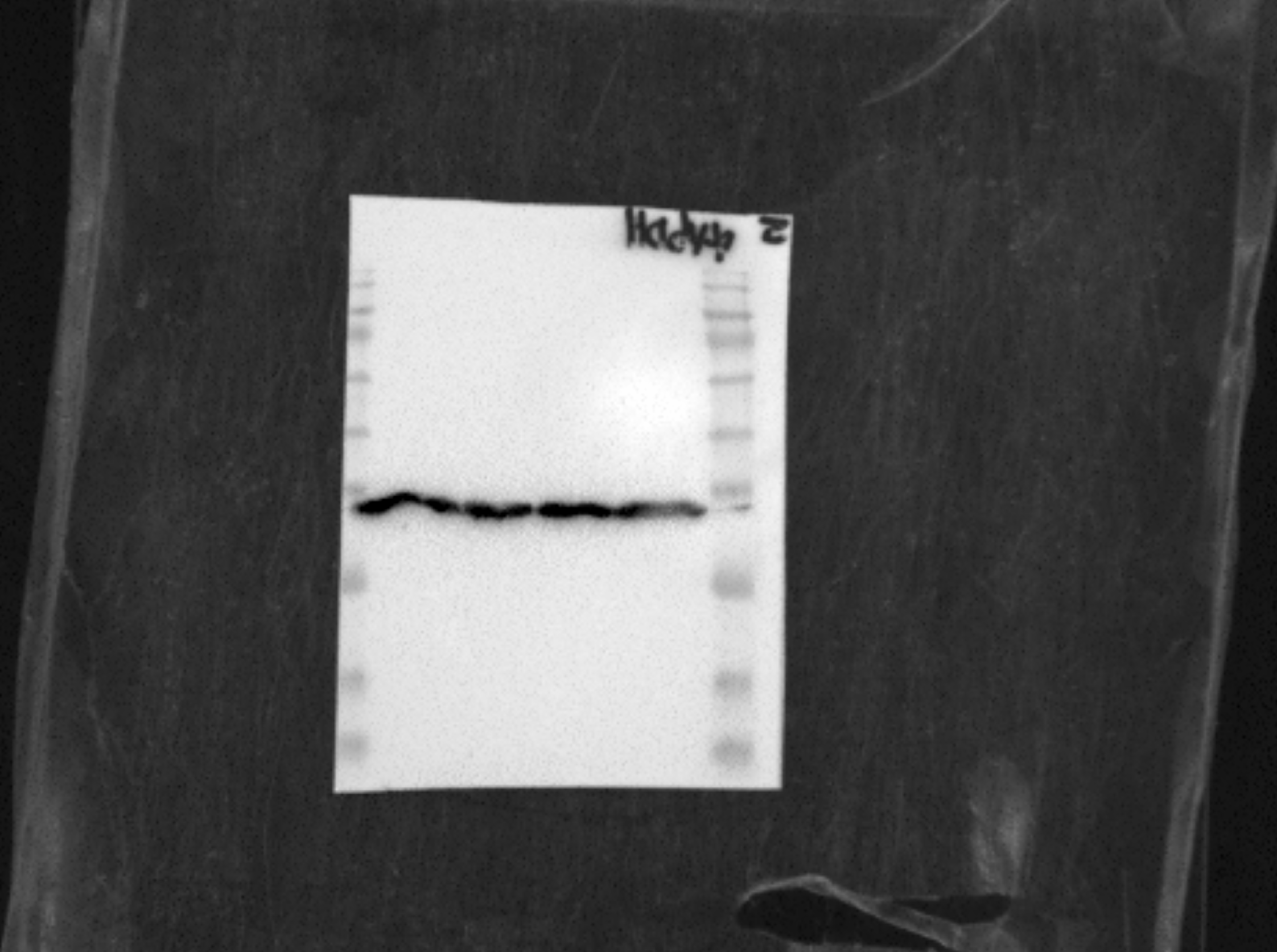

Supplement: Supplemental Information 18 [file peerj-14-21375-s018.zip › Figure 2I WB RAW Early KLHL40/klhl40-4 gapdh mark.tif]

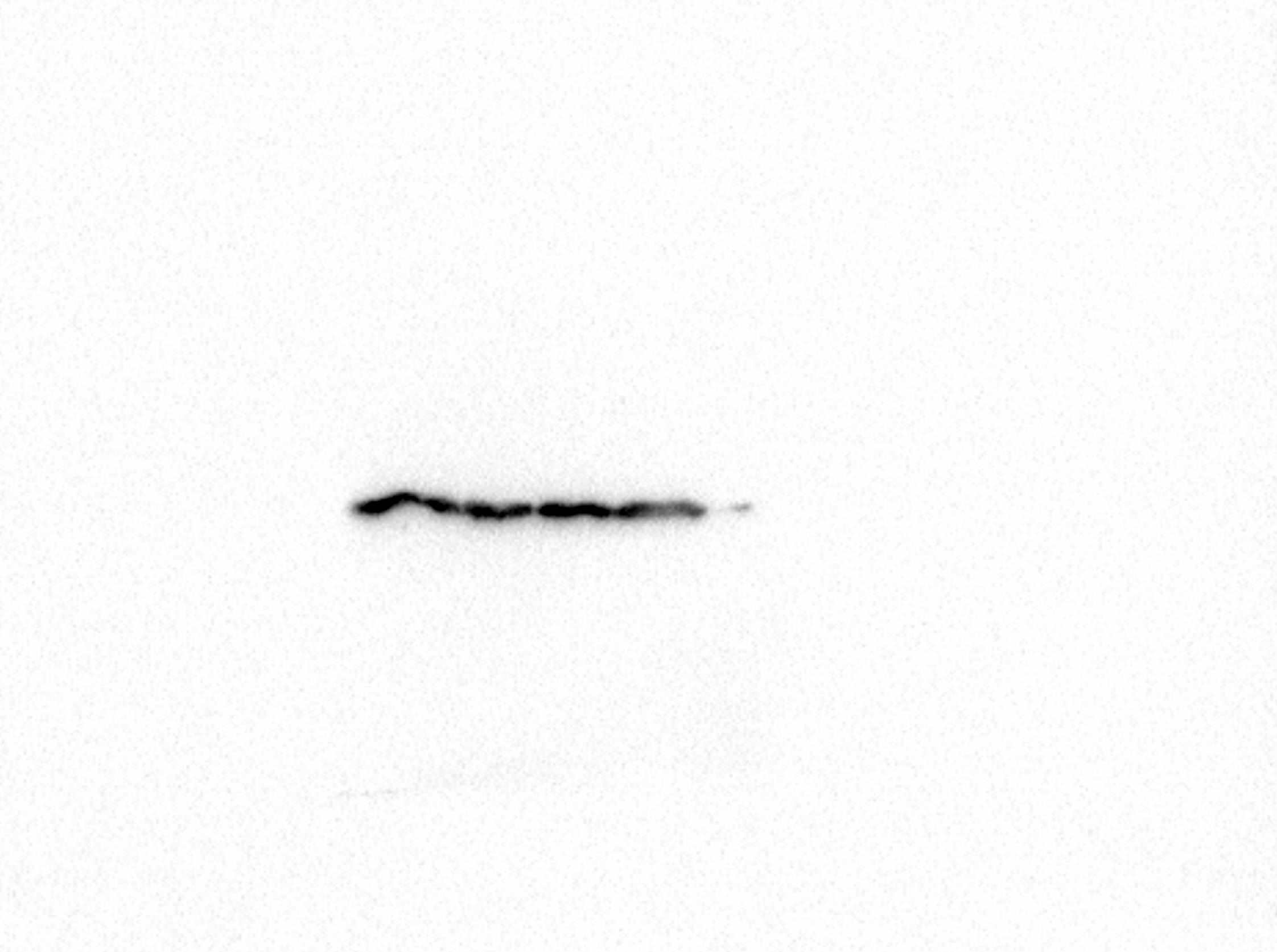

Supplement: Supplemental Information 18 [file peerj-14-21375-s018.zip › Figure 2I WB RAW Early KLHL40/klhl40-4 gapdh.tif]

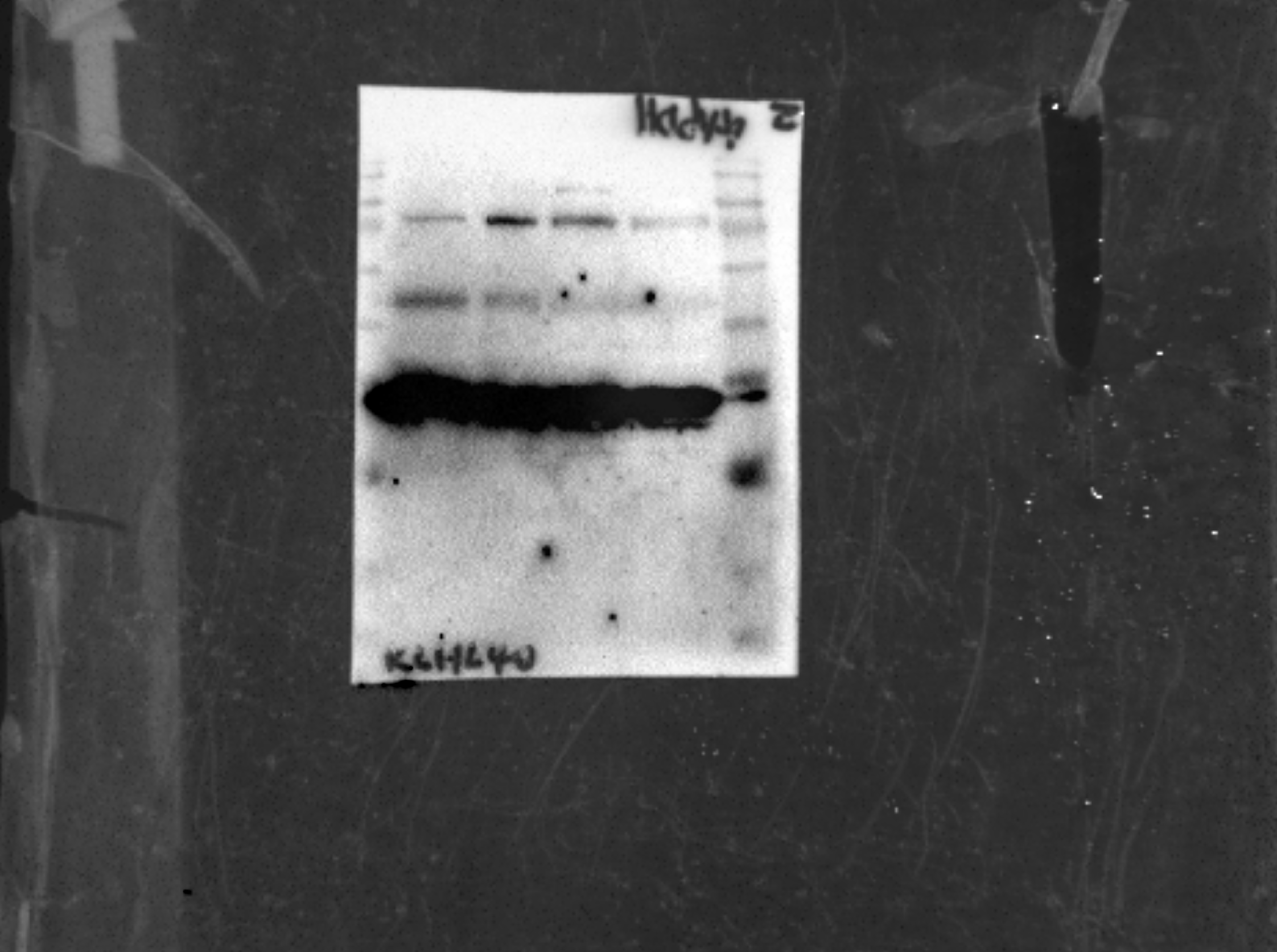

Supplement: Supplemental Information 18 [file peerj-14-21375-s018.zip › Figure 2I WB RAW Early KLHL40/KLHL40-4 mark.tif]

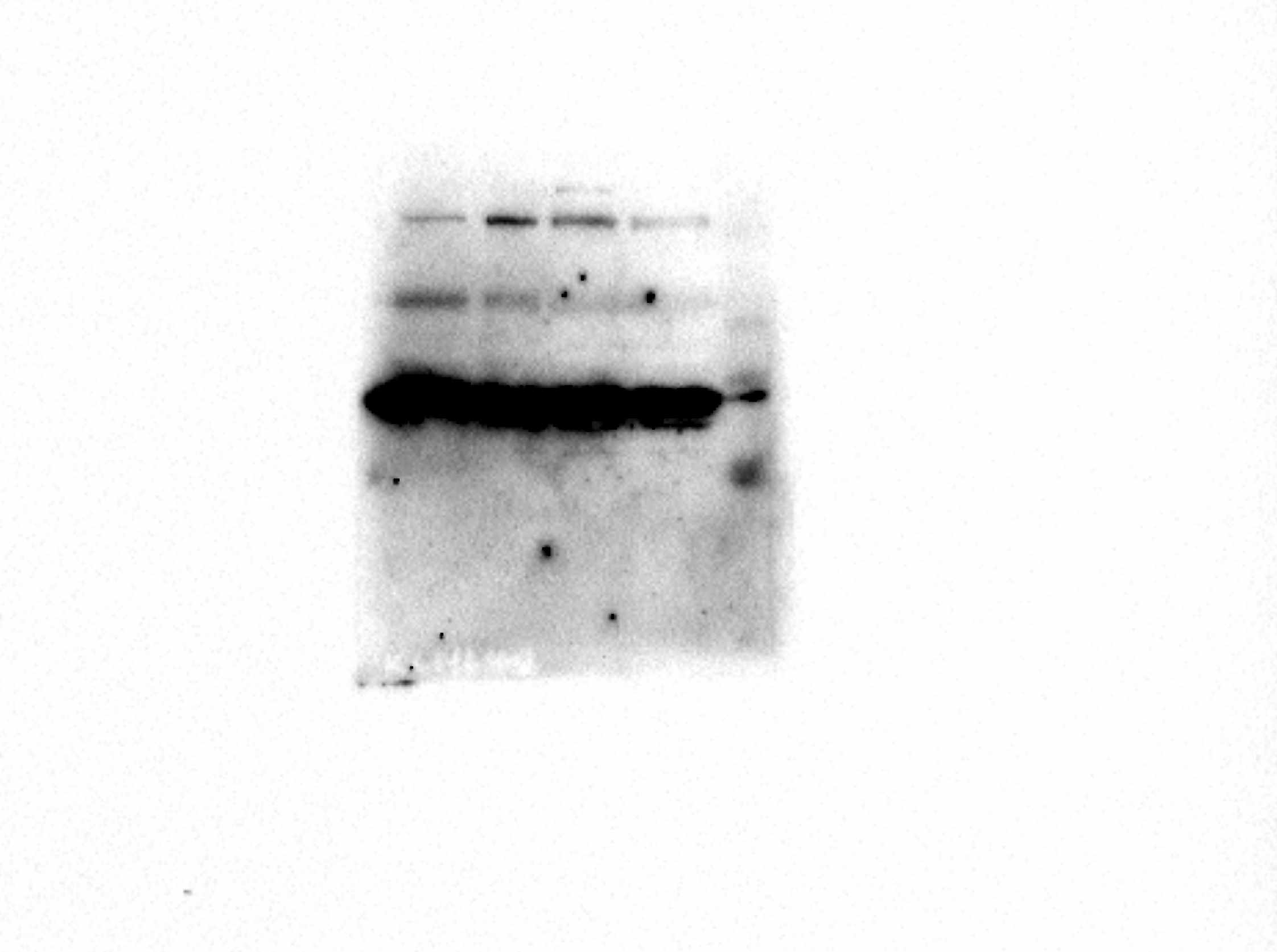

Supplement: Supplemental Information 18 [file peerj-14-21375-s018.zip › Figure 2I WB RAW Early KLHL40/KLHL40-4.tif]

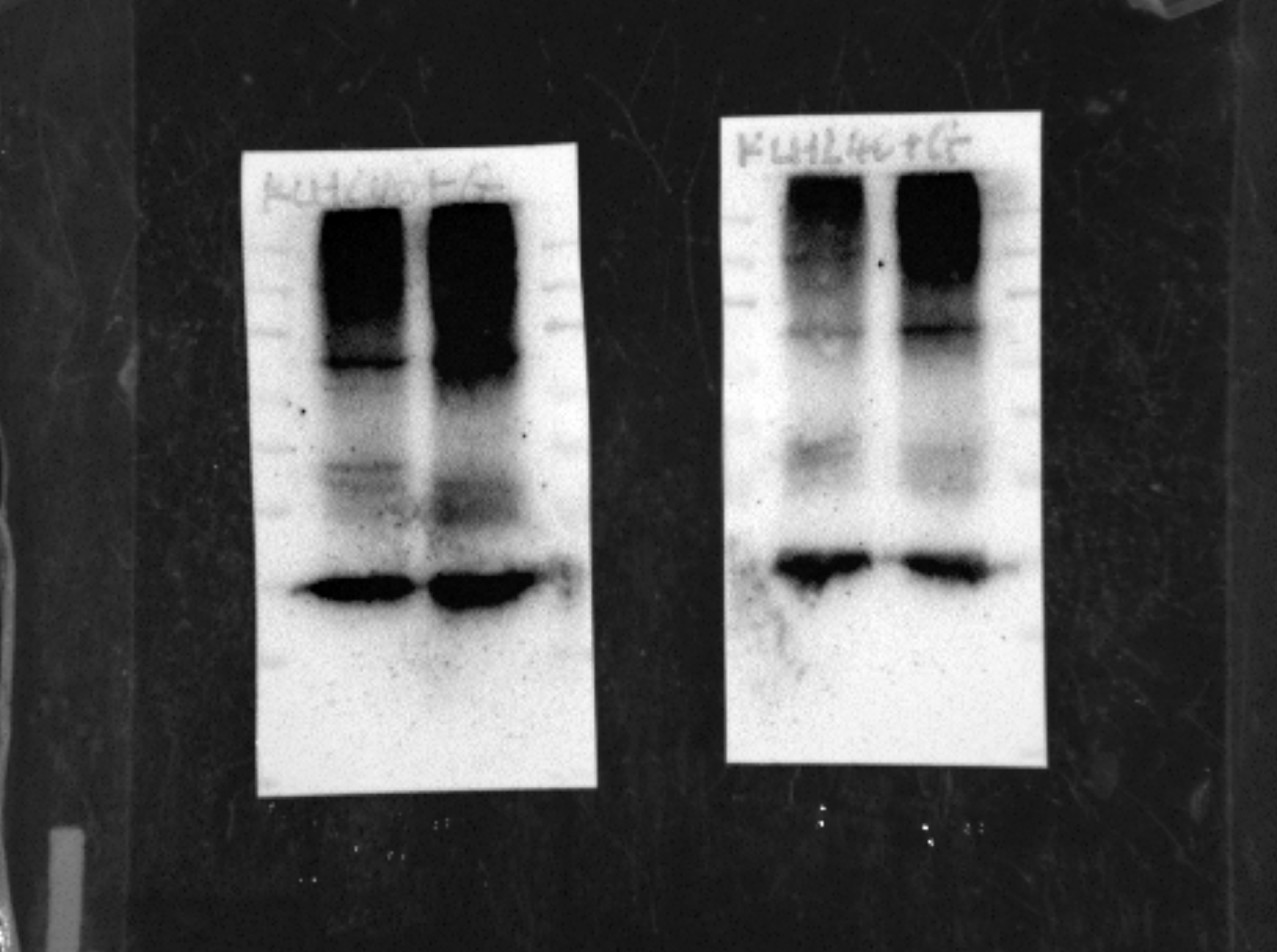

Supplement: Supplemental Information 18 [file peerj-14-21375-s018.zip › Figure 2I WB RAW Early KLHL40/KLHL40-5 AND GAPDH mark.tif]

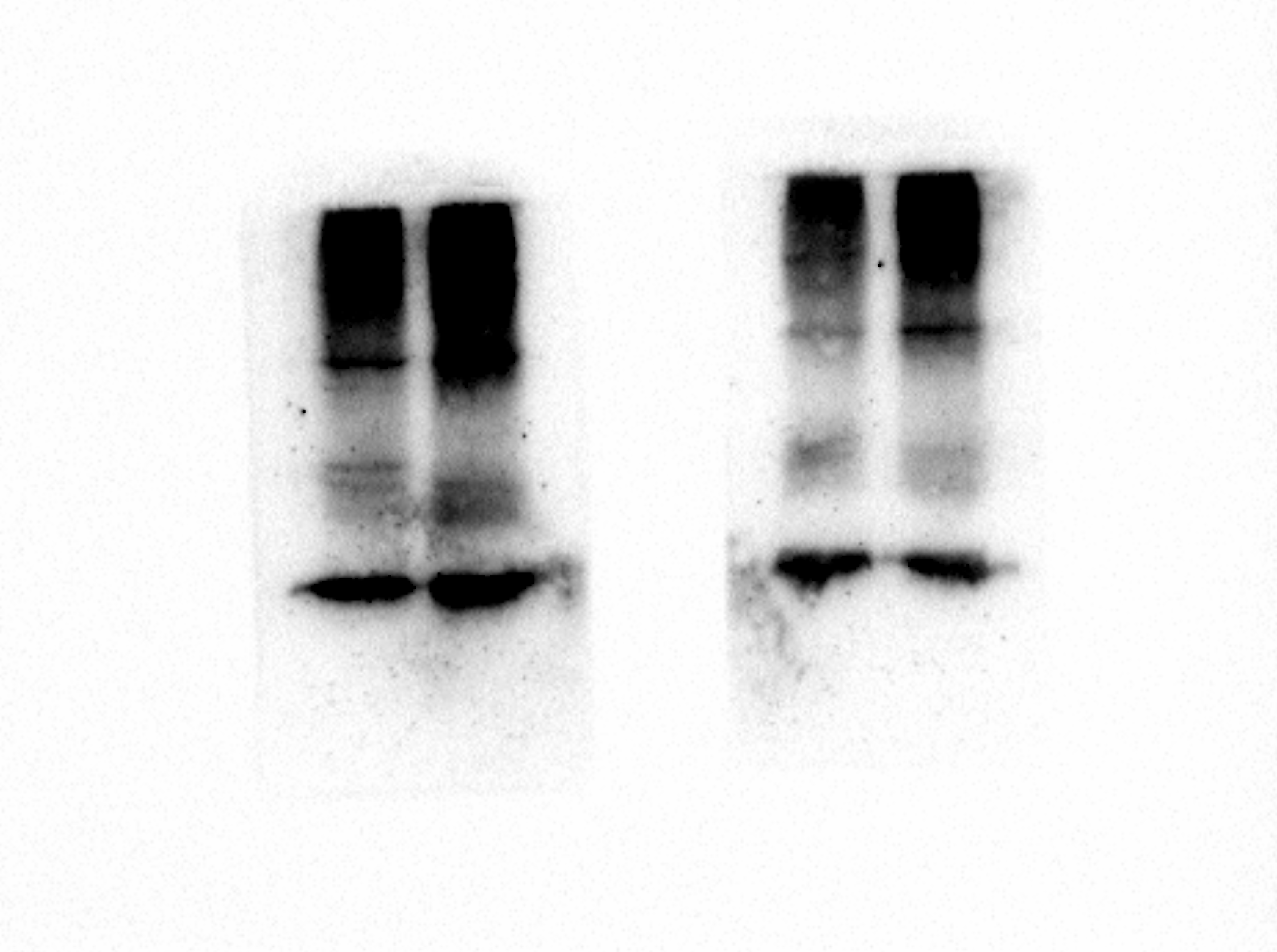

Supplement: Supplemental Information 18 [file peerj-14-21375-s018.zip › Figure 2I WB RAW Early KLHL40/KLHL40-5 AND GAPDH.tif]

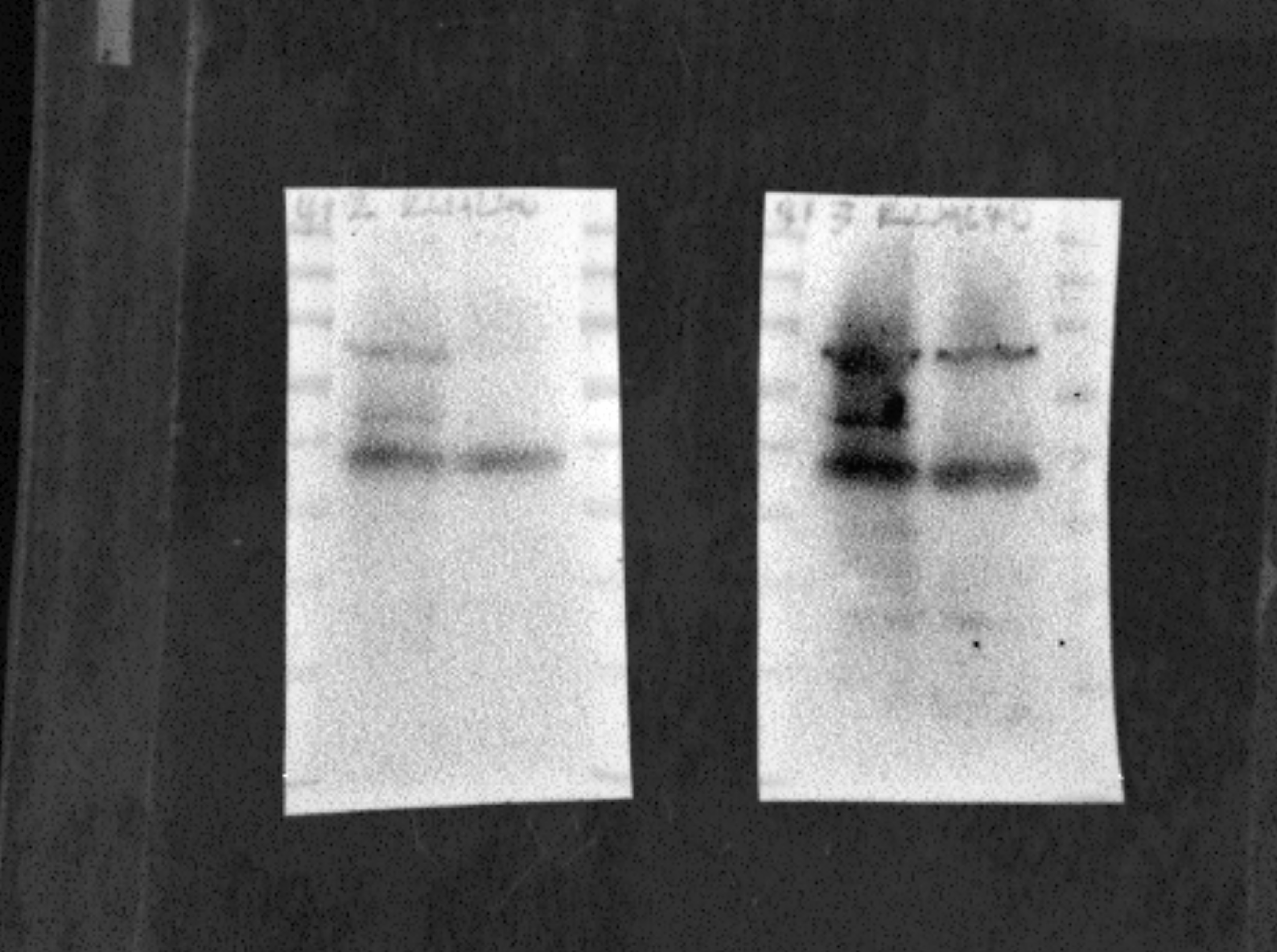

Supplement: Supplemental Information 19 [file peerj-14-21375-s019.zip › Figure 2J WB RAW Late KLHL40/klhl40 -1 +MARK.tif]

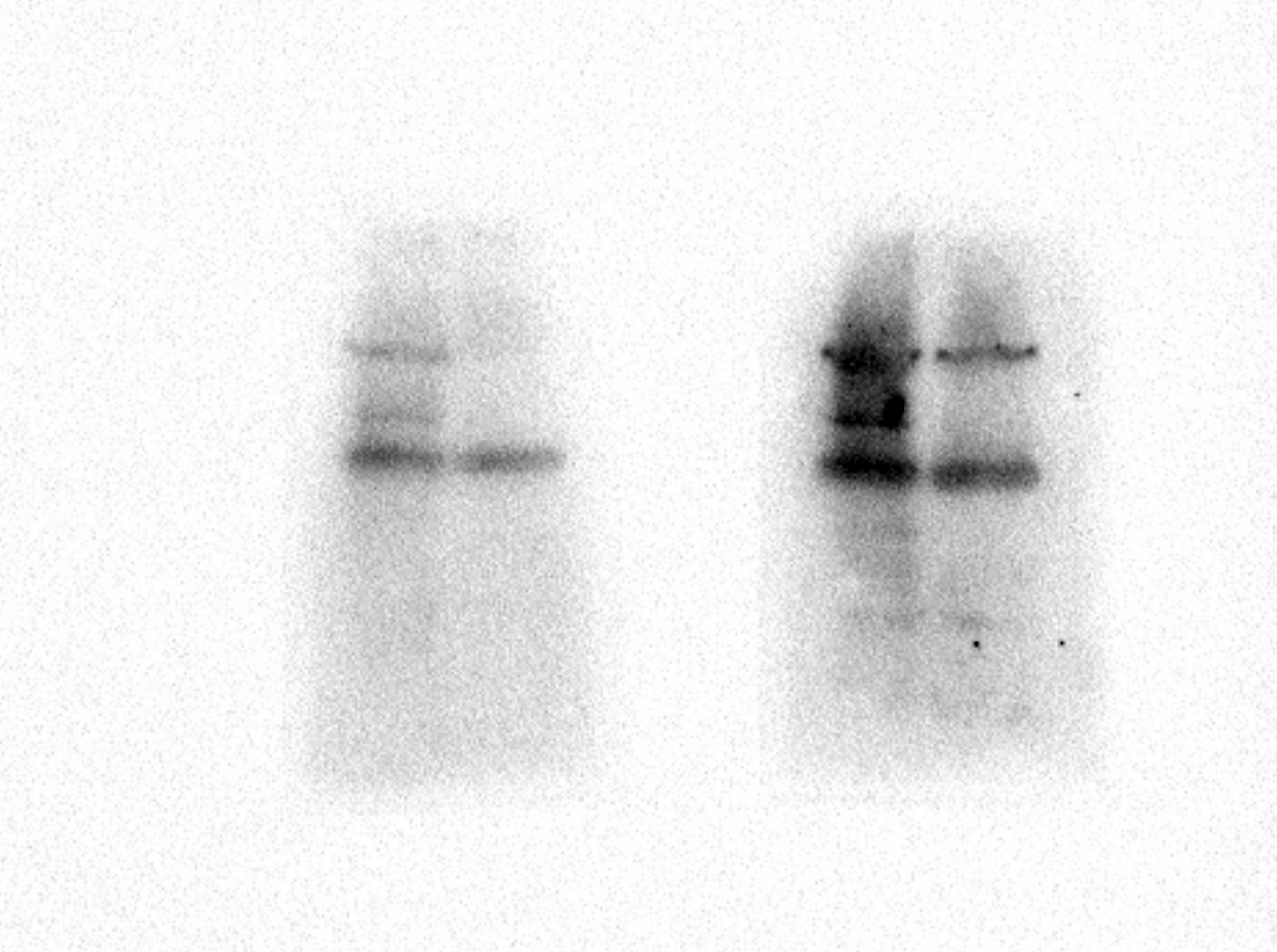

Supplement: Supplemental Information 19 [file peerj-14-21375-s019.zip › Figure 2J WB RAW Late KLHL40/klhl40-1 (2).tif]

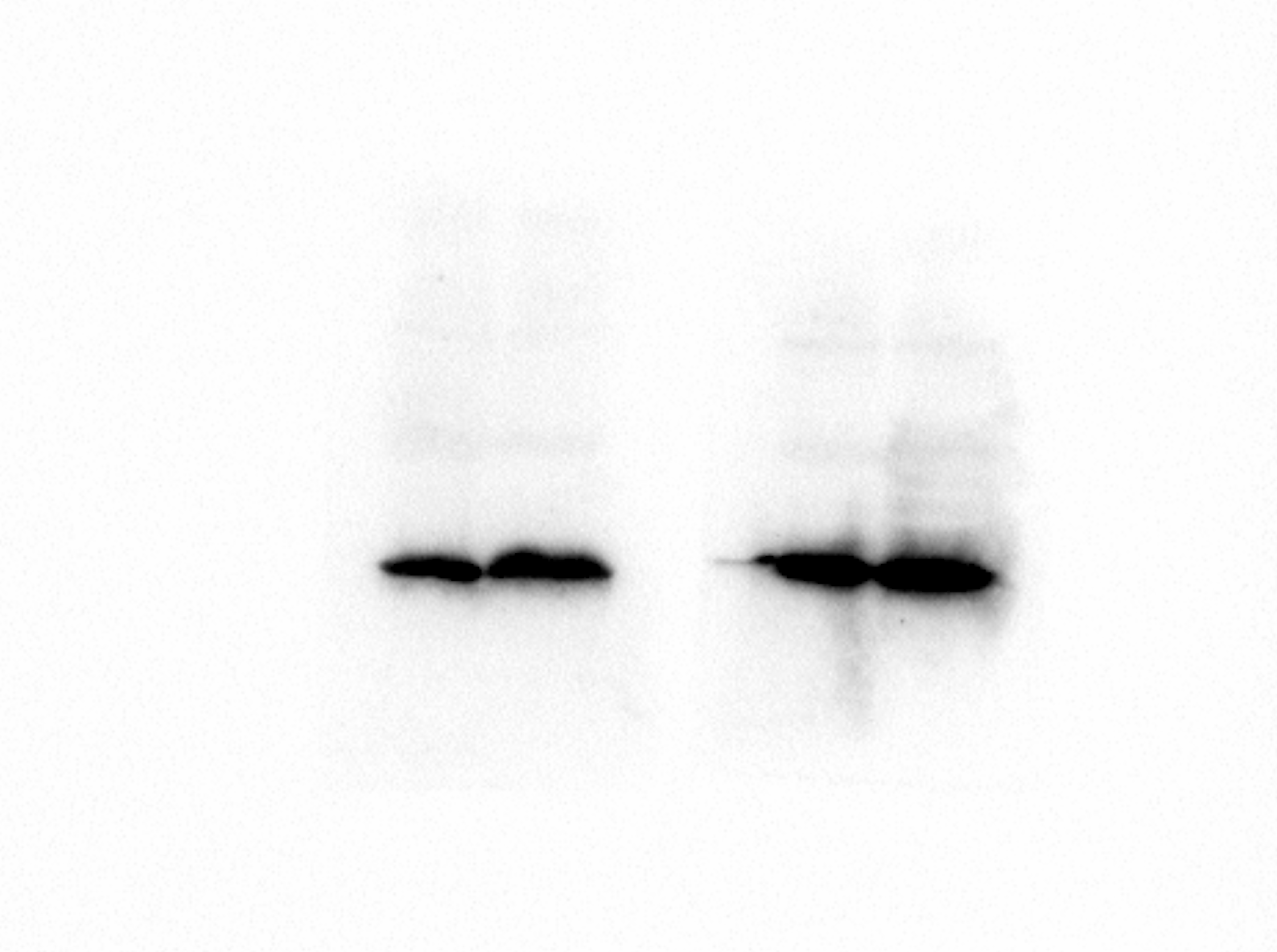

Supplement: Supplemental Information 19 [file peerj-14-21375-s019.zip › Figure 2J WB RAW Late KLHL40/KLHL40-1 GAP.tif]

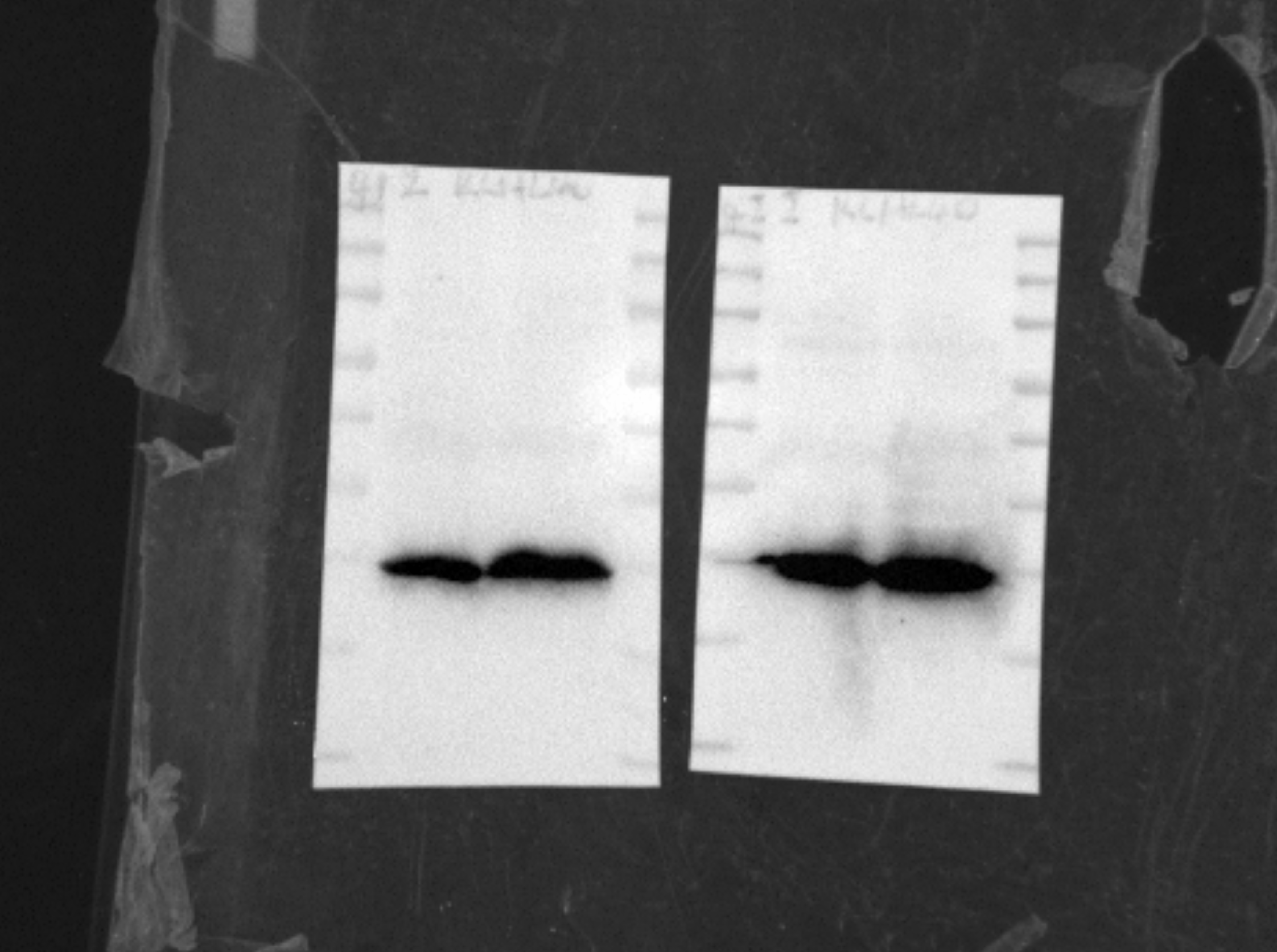

Supplement: Supplemental Information 19 [file peerj-14-21375-s019.zip › Figure 2J WB RAW Late KLHL40/KLHL40-1 GAP+MARK.tif]

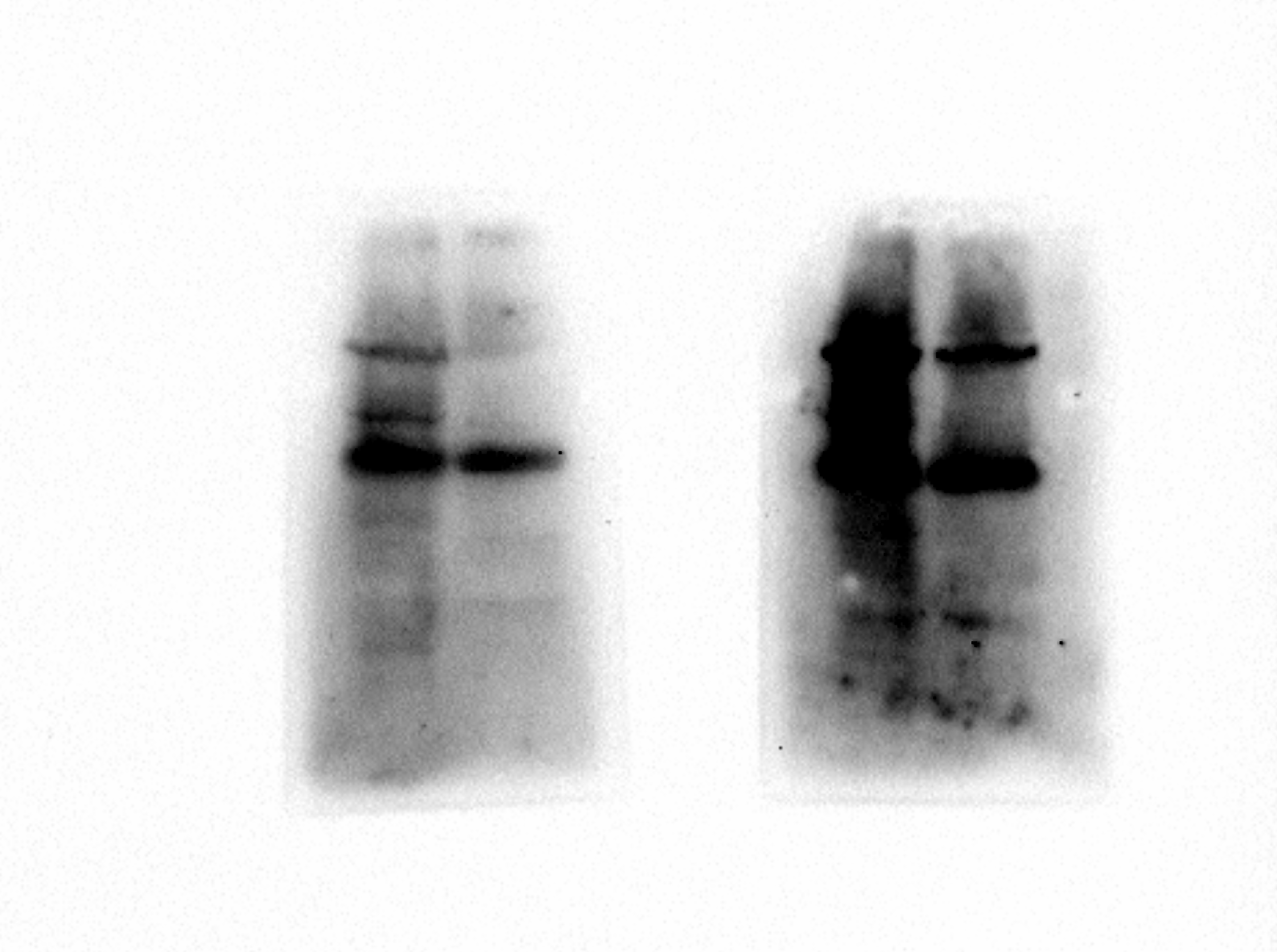

Supplement: Supplemental Information 19 [file peerj-14-21375-s019.zip › Figure 2J WB RAW Late KLHL40/klhl40-1.tif]

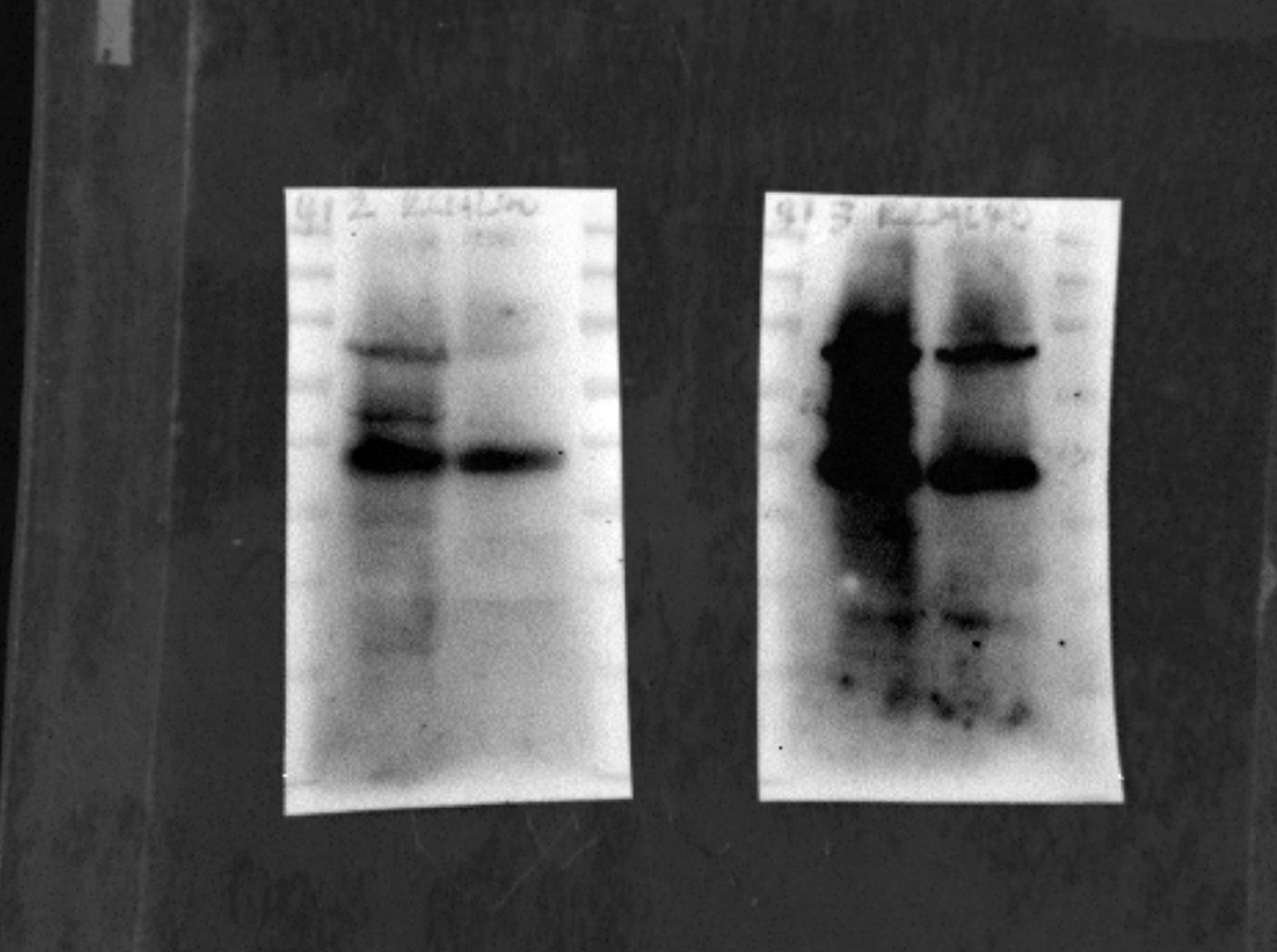

Supplement: Supplemental Information 19 [file peerj-14-21375-s019.zip › Figure 2J WB RAW Late KLHL40/klhl40-1+MARK.tif]

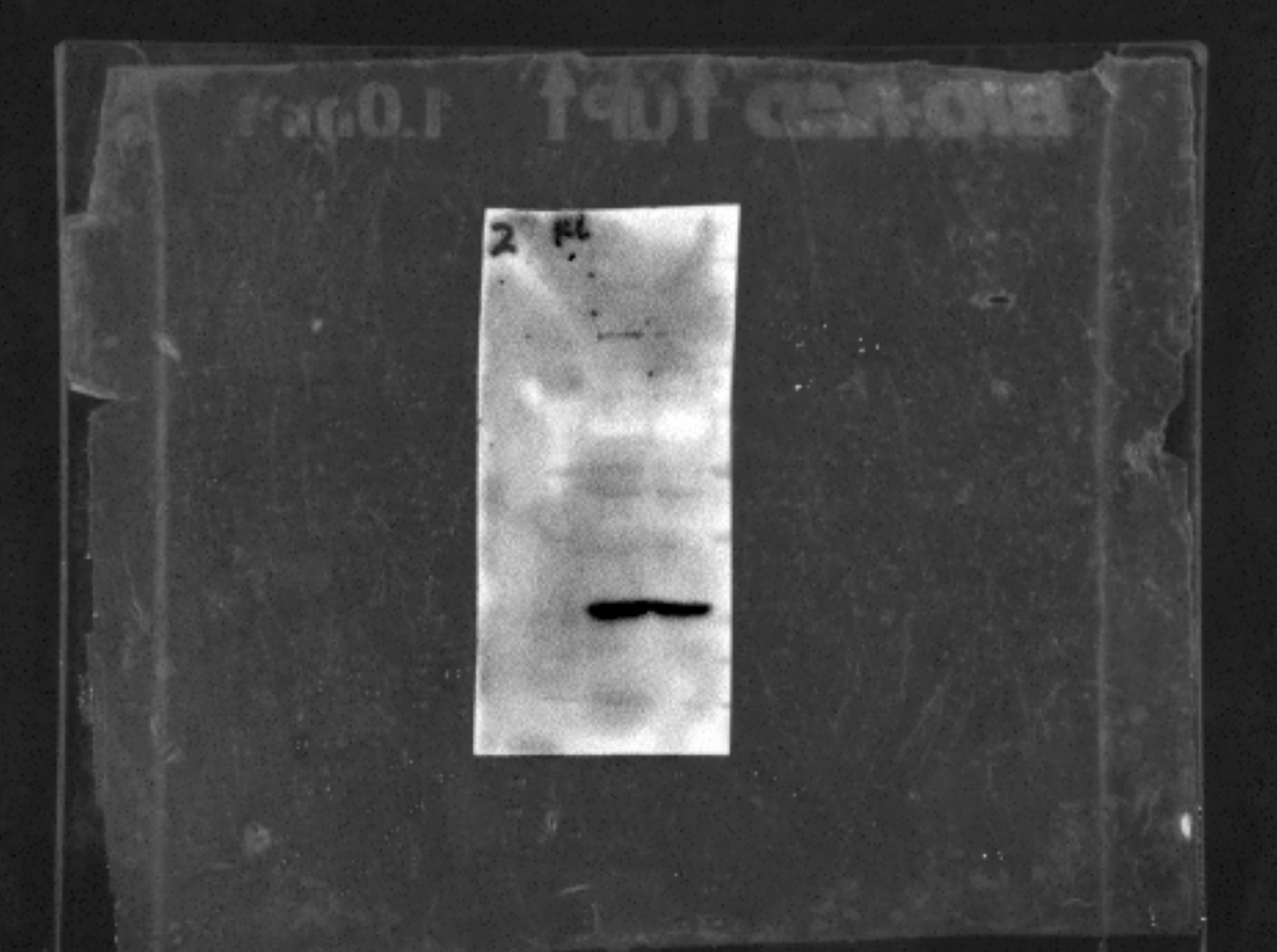

Supplement: Supplemental Information 19 [file peerj-14-21375-s019.zip › Figure 2J WB RAW Late KLHL40/klhl40-3+gap marker.tif]

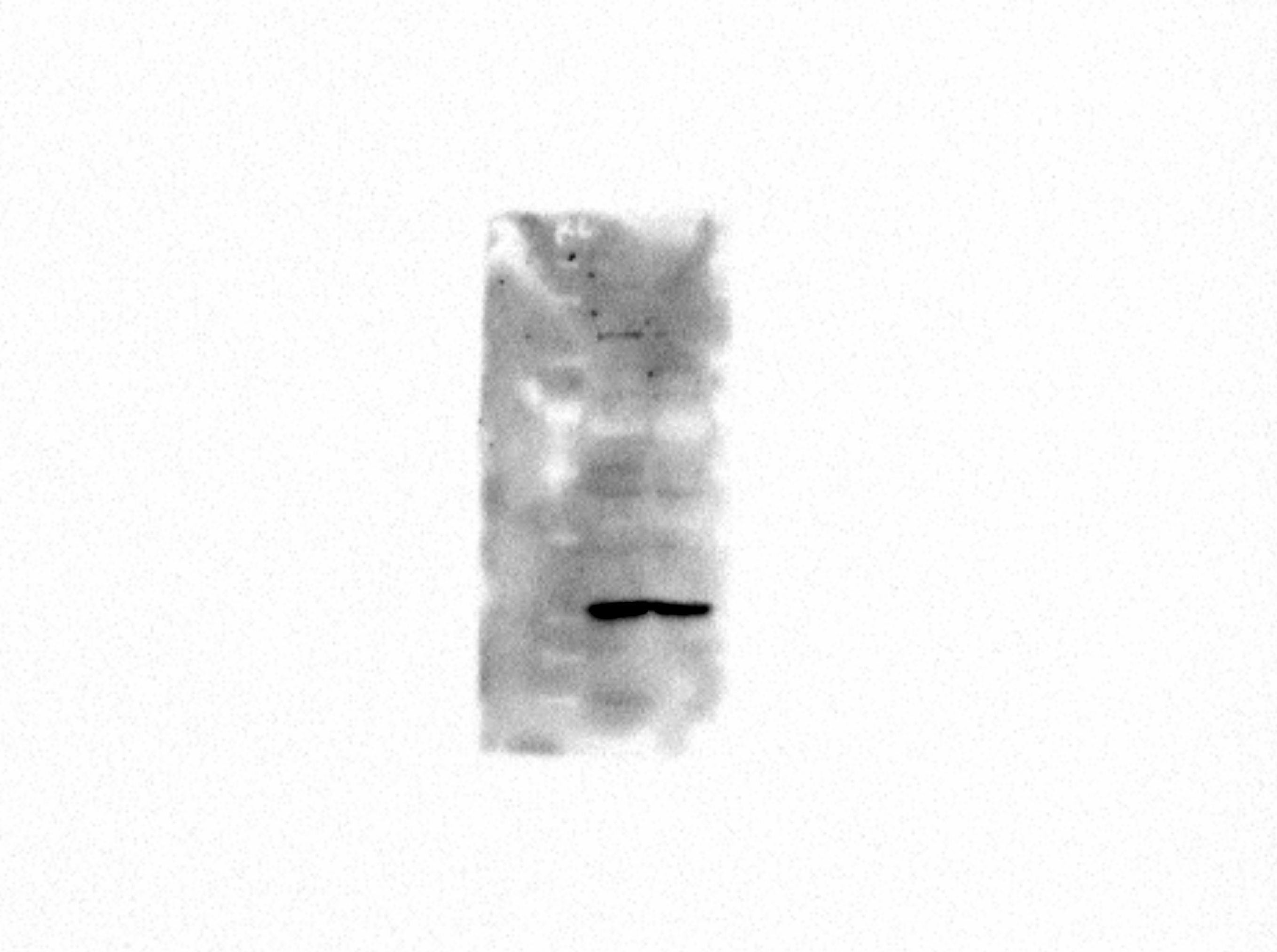

Supplement: Supplemental Information 19 [file peerj-14-21375-s019.zip › Figure 2J WB RAW Late KLHL40/klhl40-3+gap.tif]

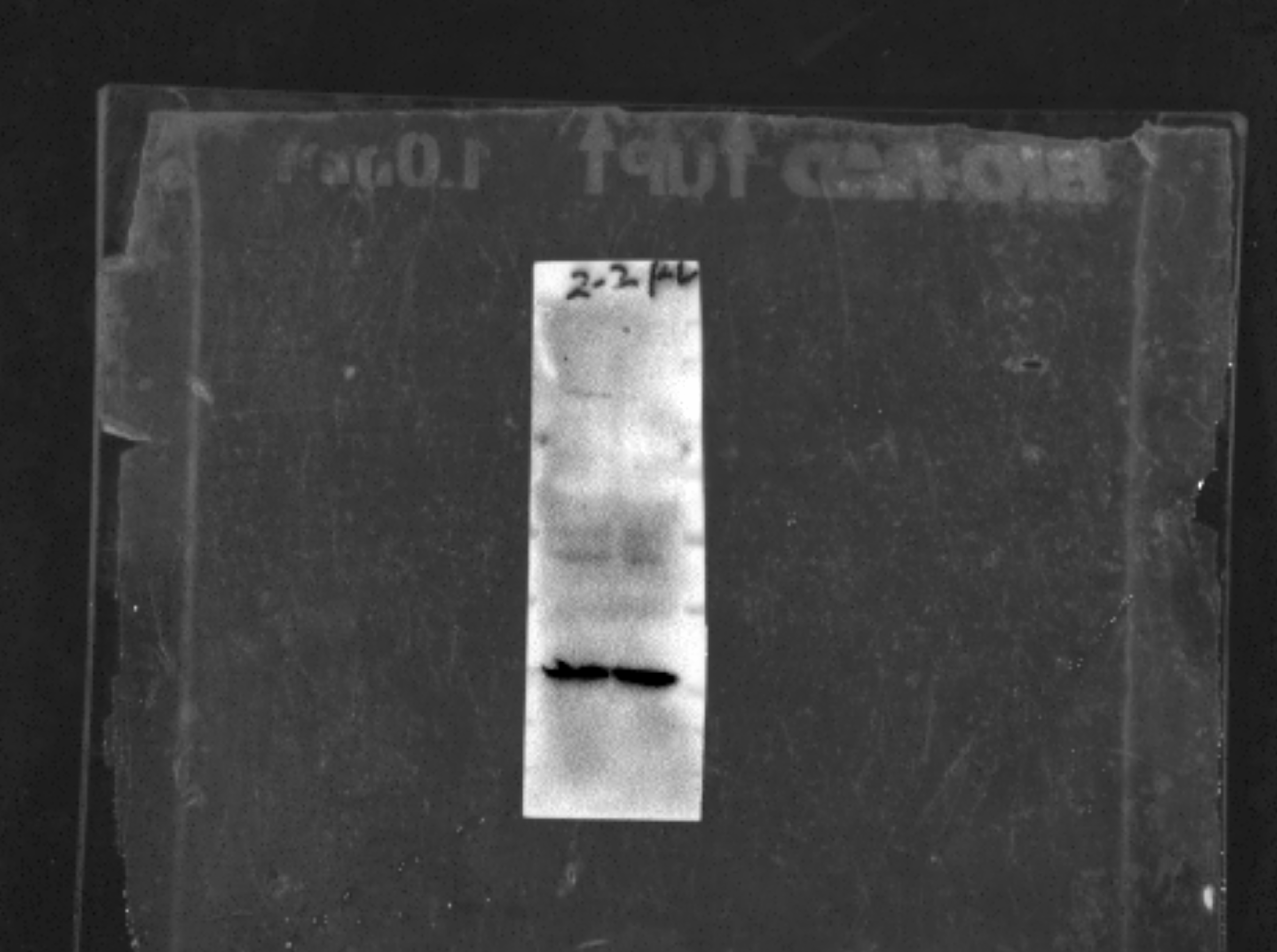

Supplement: Supplemental Information 19 [file peerj-14-21375-s019.zip › Figure 2J WB RAW Late KLHL40/klhl40-4+gap marker.tif]

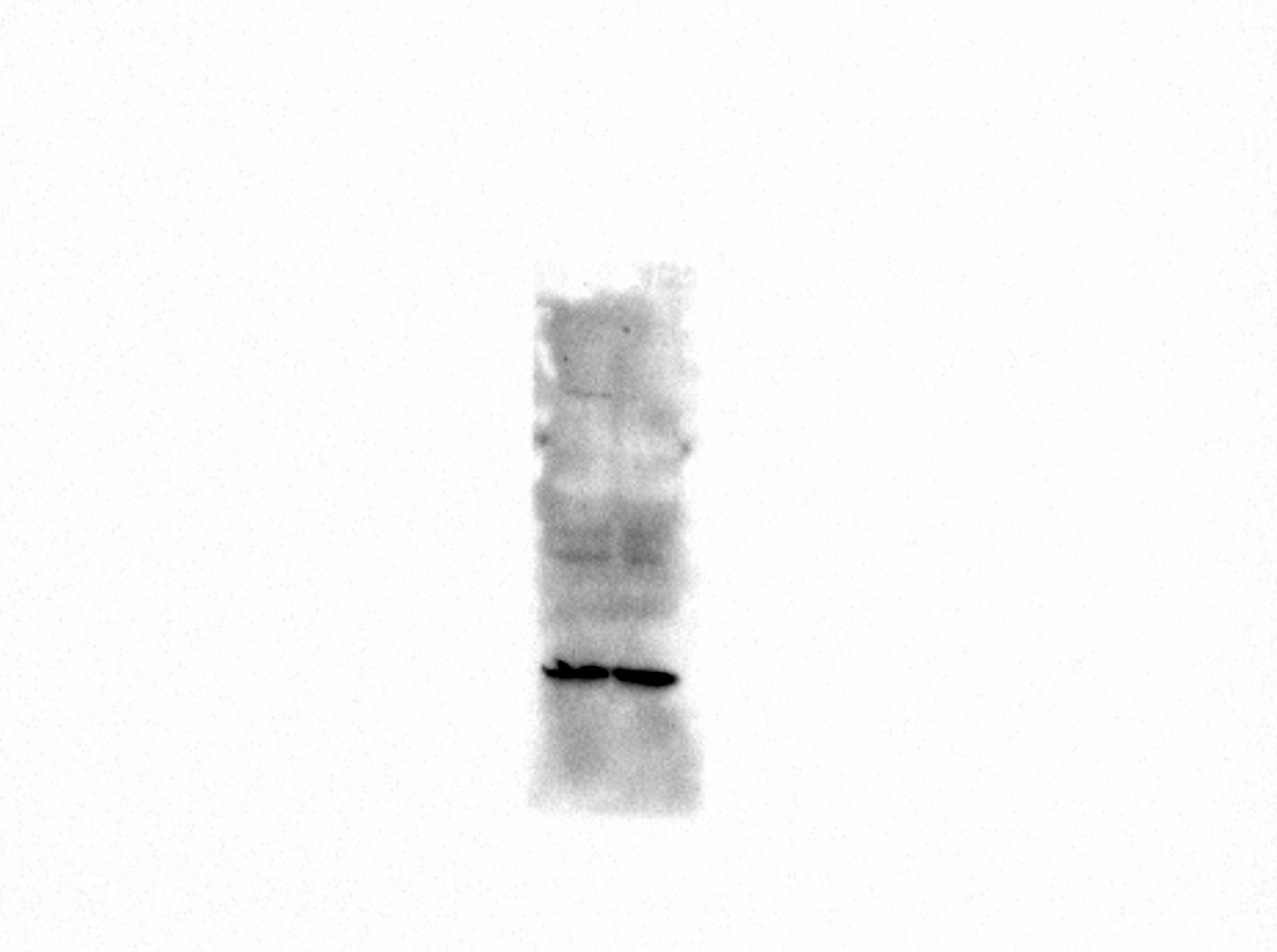

Supplement: Supplemental Information 19 [file peerj-14-21375-s019.zip › Figure 2J WB RAW Late KLHL40/klhl40-4+gap.tif]

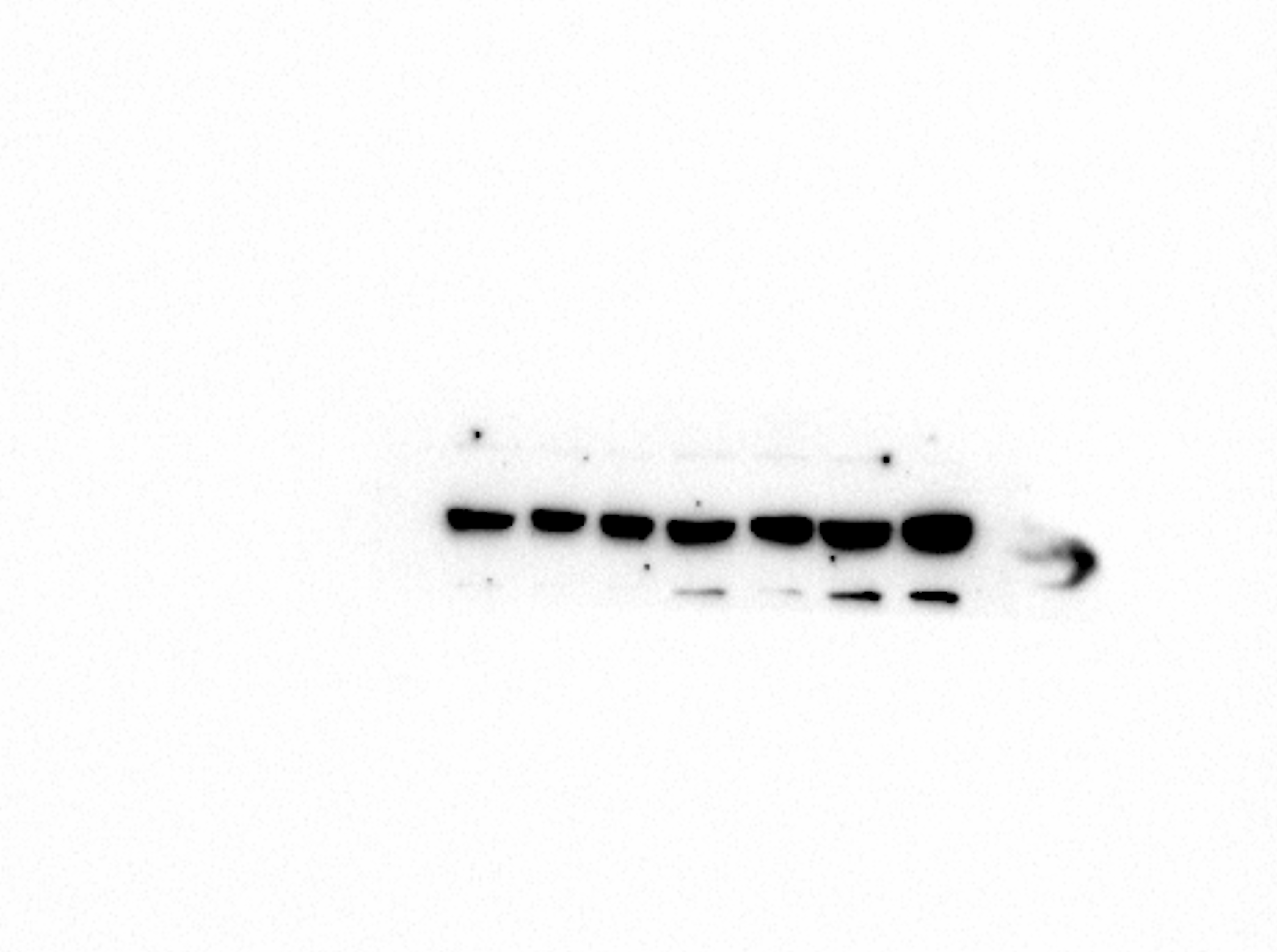

Supplement: Supplemental Information 20 [file peerj-14-21375-s020.zip › Figure 2M WB RAW 0-24h/1ACTIN.tif]

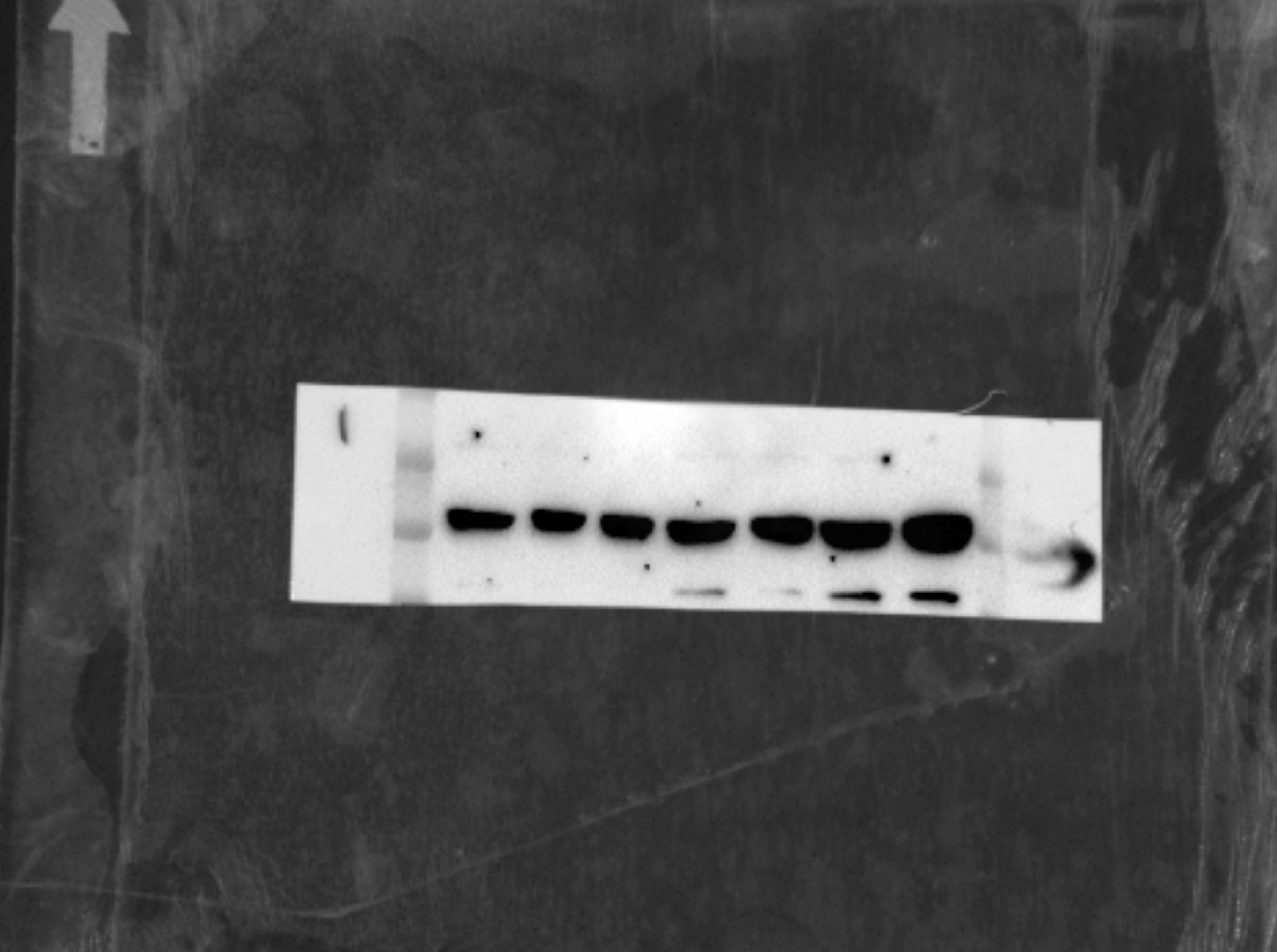

Supplement: Supplemental Information 20 [file peerj-14-21375-s020.zip › Figure 2M WB RAW 0-24h/1ACTIN+MARKER.tif]

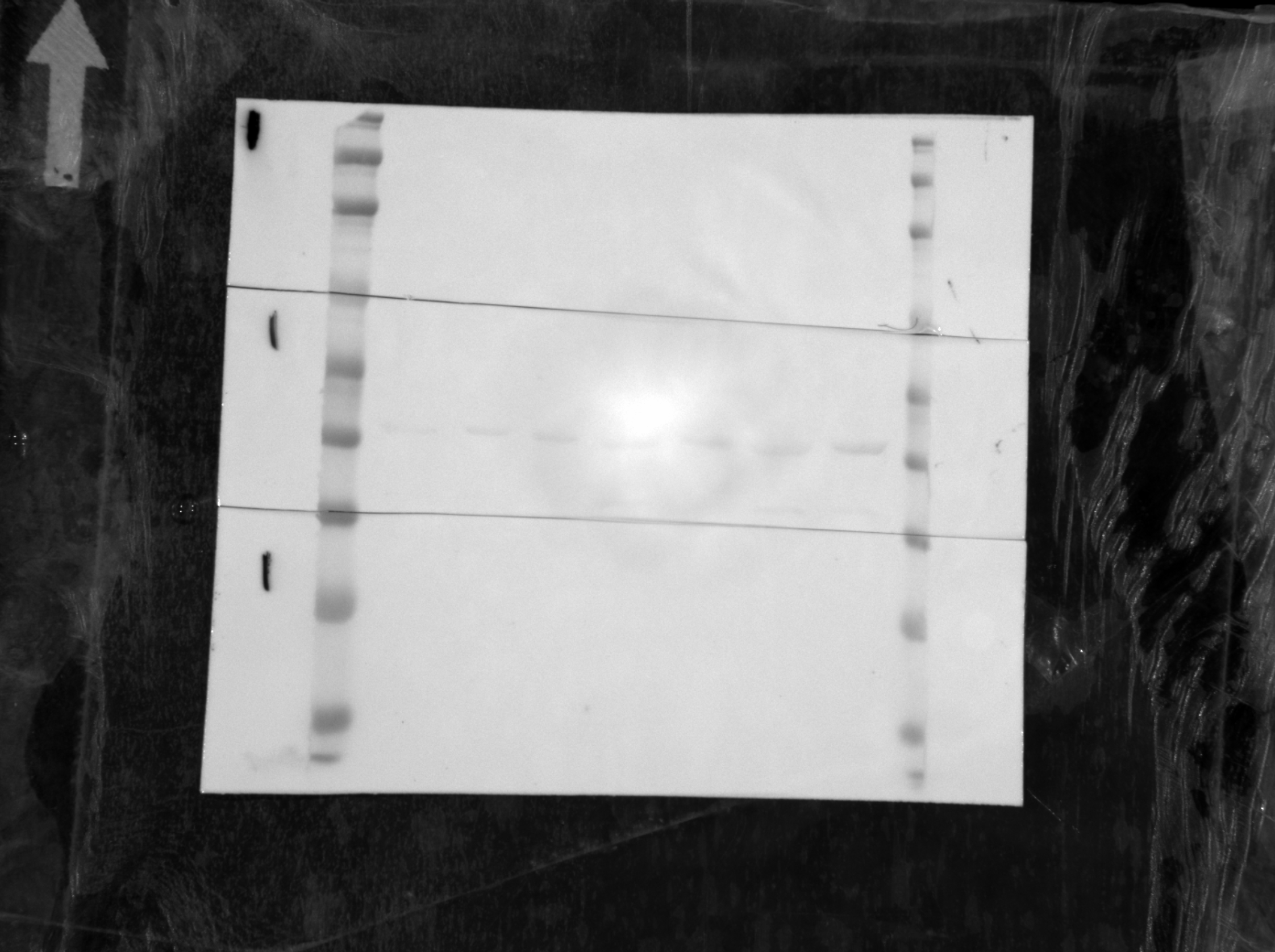

Supplement: Supplemental Information 20 [file peerj-14-21375-s020.zip › Figure 2M WB RAW 0-24h/1ALL.tif]

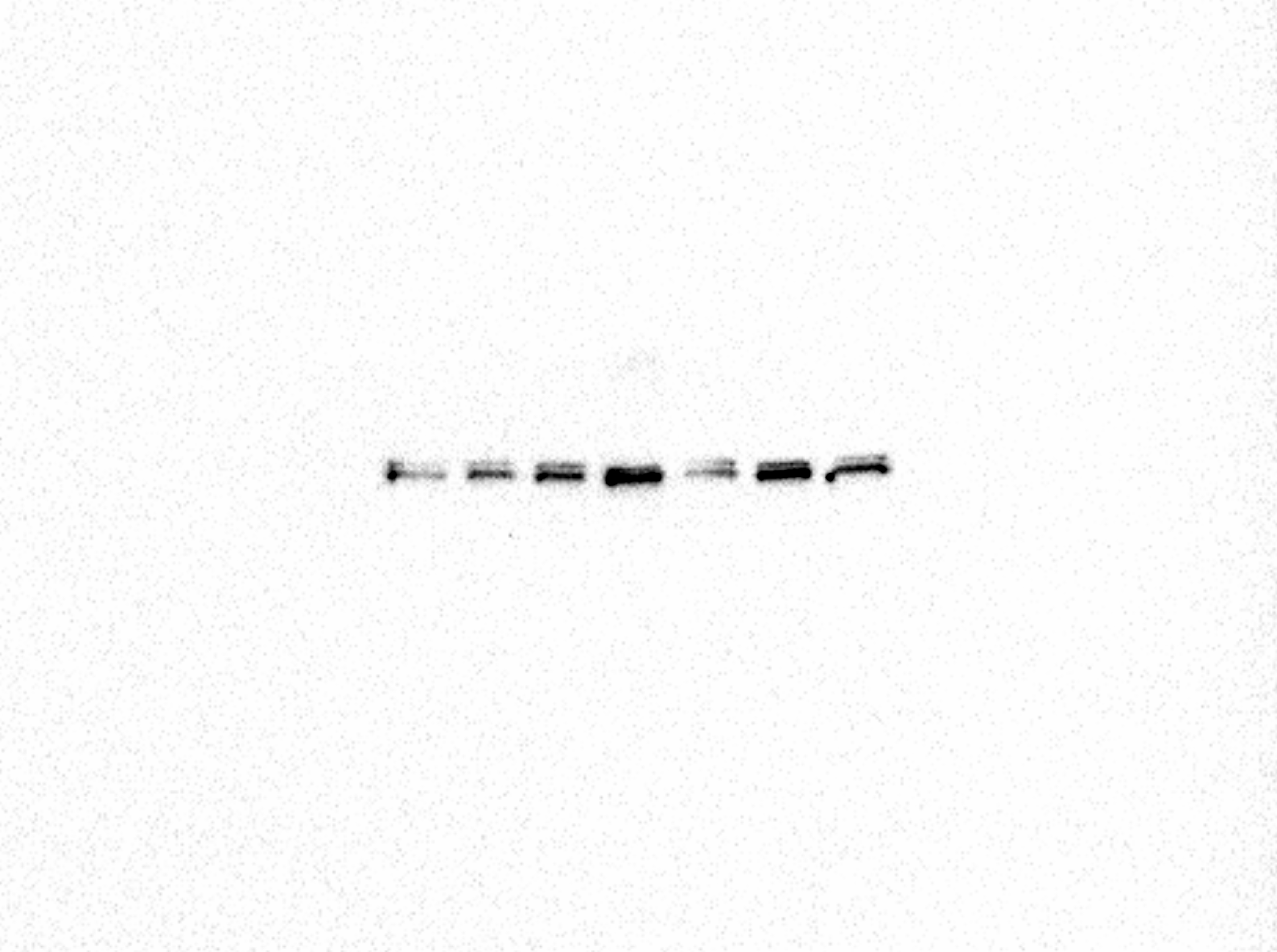

Supplement: Supplemental Information 20 [file peerj-14-21375-s020.zip › Figure 2M WB RAW 0-24h/1KLHL40.tif]

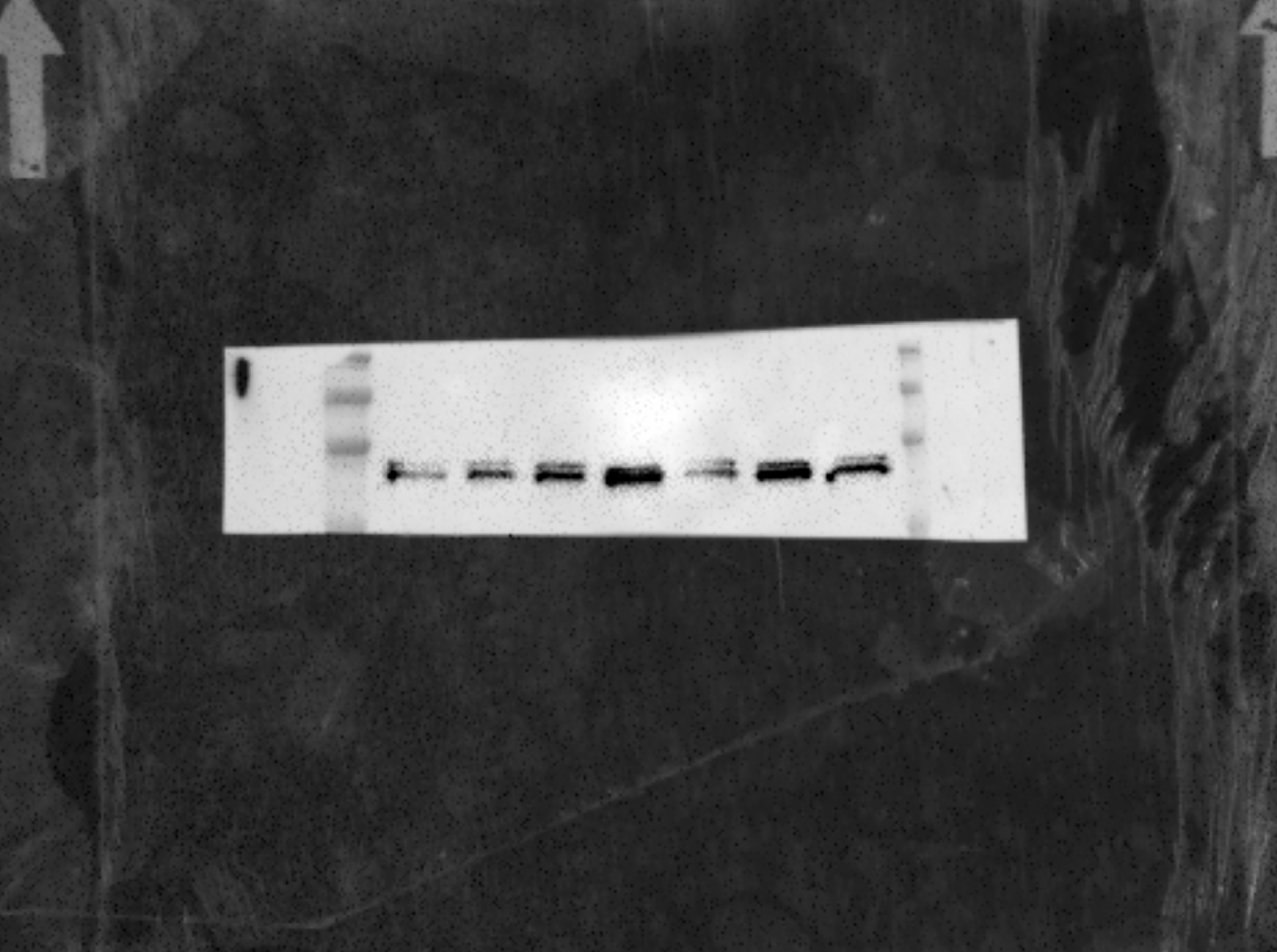

Supplement: Supplemental Information 20 [file peerj-14-21375-s020.zip › Figure 2M WB RAW 0-24h/1KLHL40+MARKER.tif]

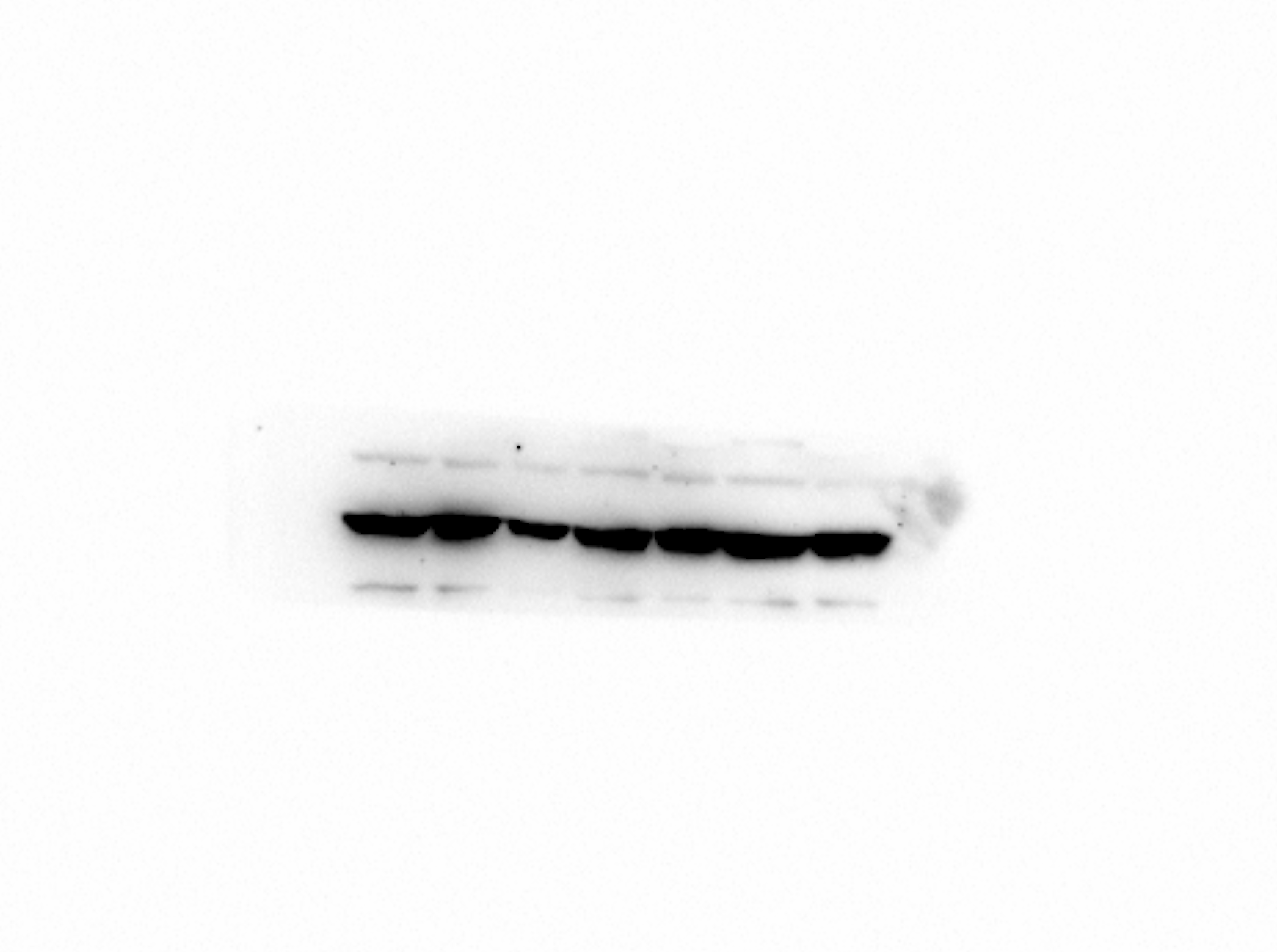

Supplement: Supplemental Information 20 [file peerj-14-21375-s020.zip › Figure 2M WB RAW 0-24h/2ACTIN.tif]

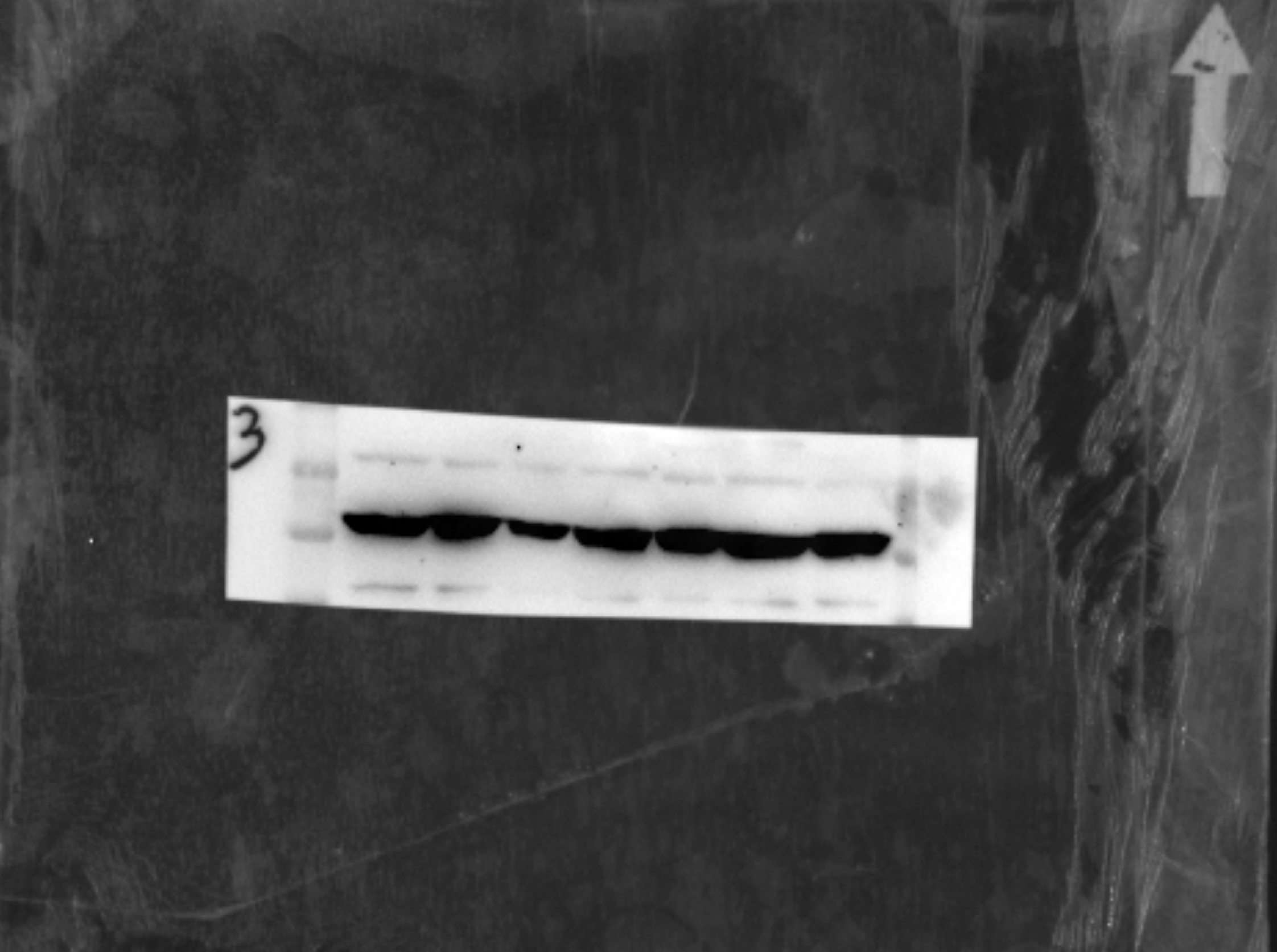

Supplement: Supplemental Information 20 [file peerj-14-21375-s020.zip › Figure 2M WB RAW 0-24h/2ACTIN+MARKER.tif]

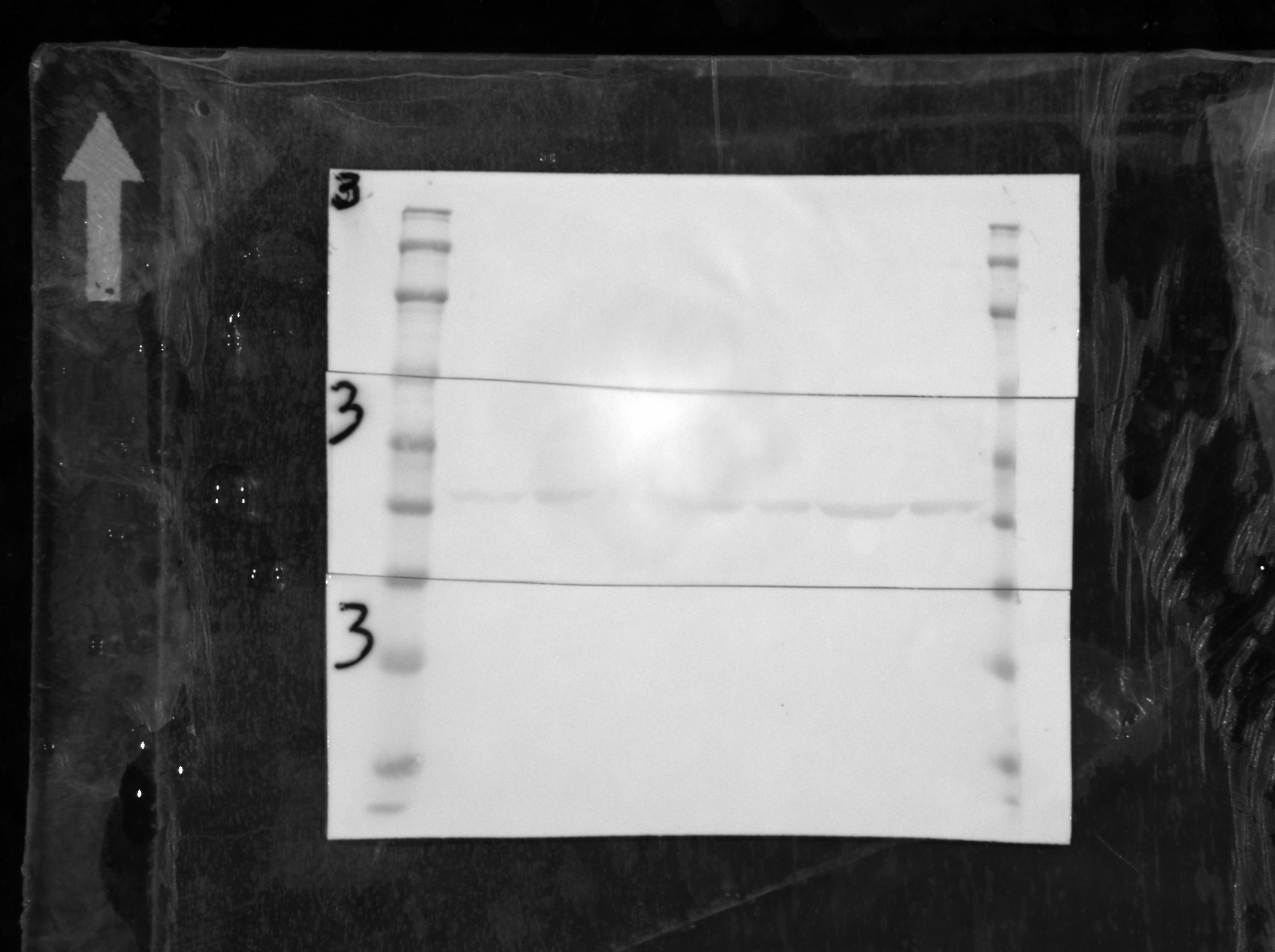

Supplement: Supplemental Information 20 [file peerj-14-21375-s020.zip › Figure 2M WB RAW 0-24h/2ALL.tif]

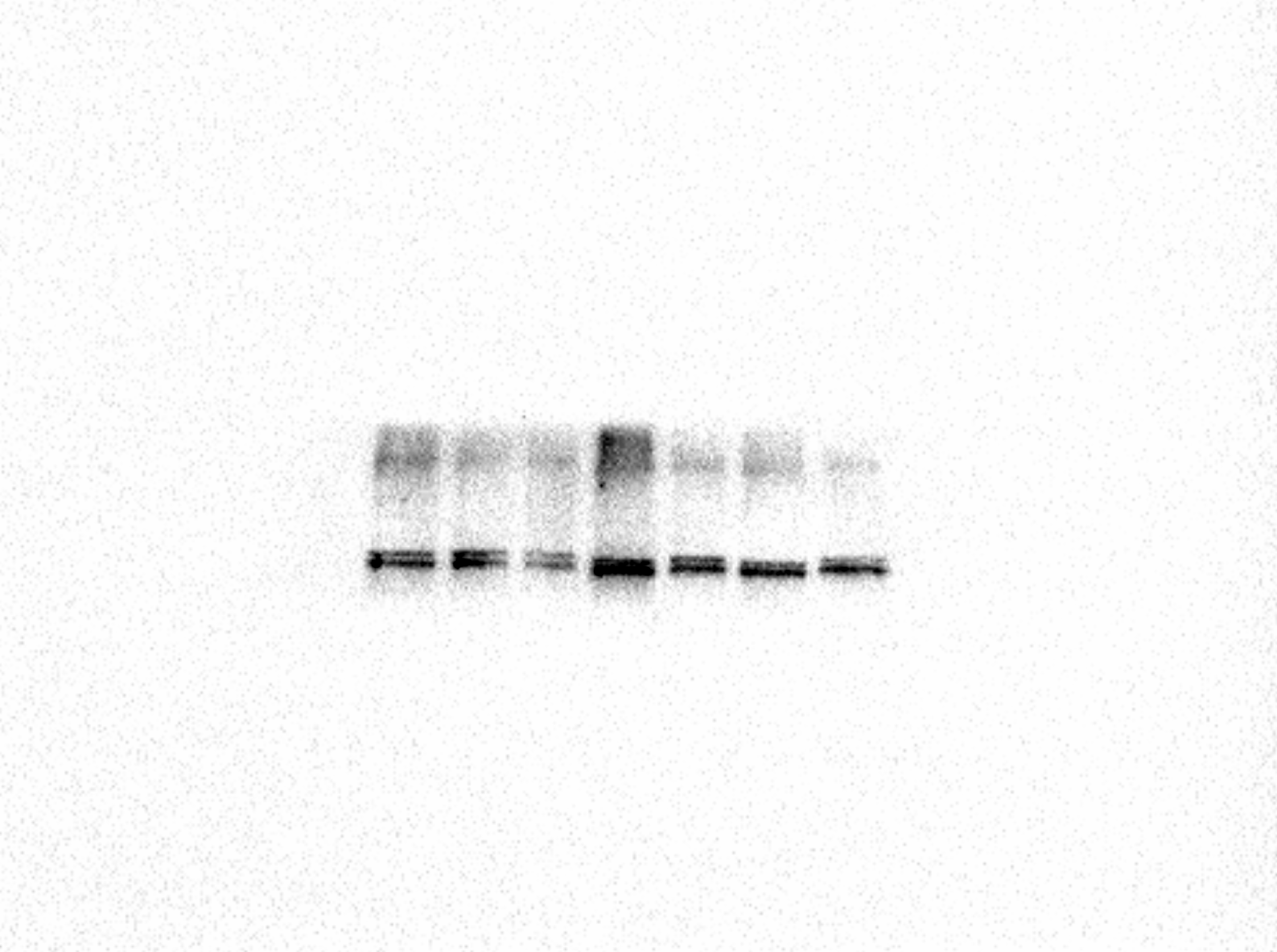

Supplement: Supplemental Information 20 [file peerj-14-21375-s020.zip › Figure 2M WB RAW 0-24h/2KLHL40.tif]

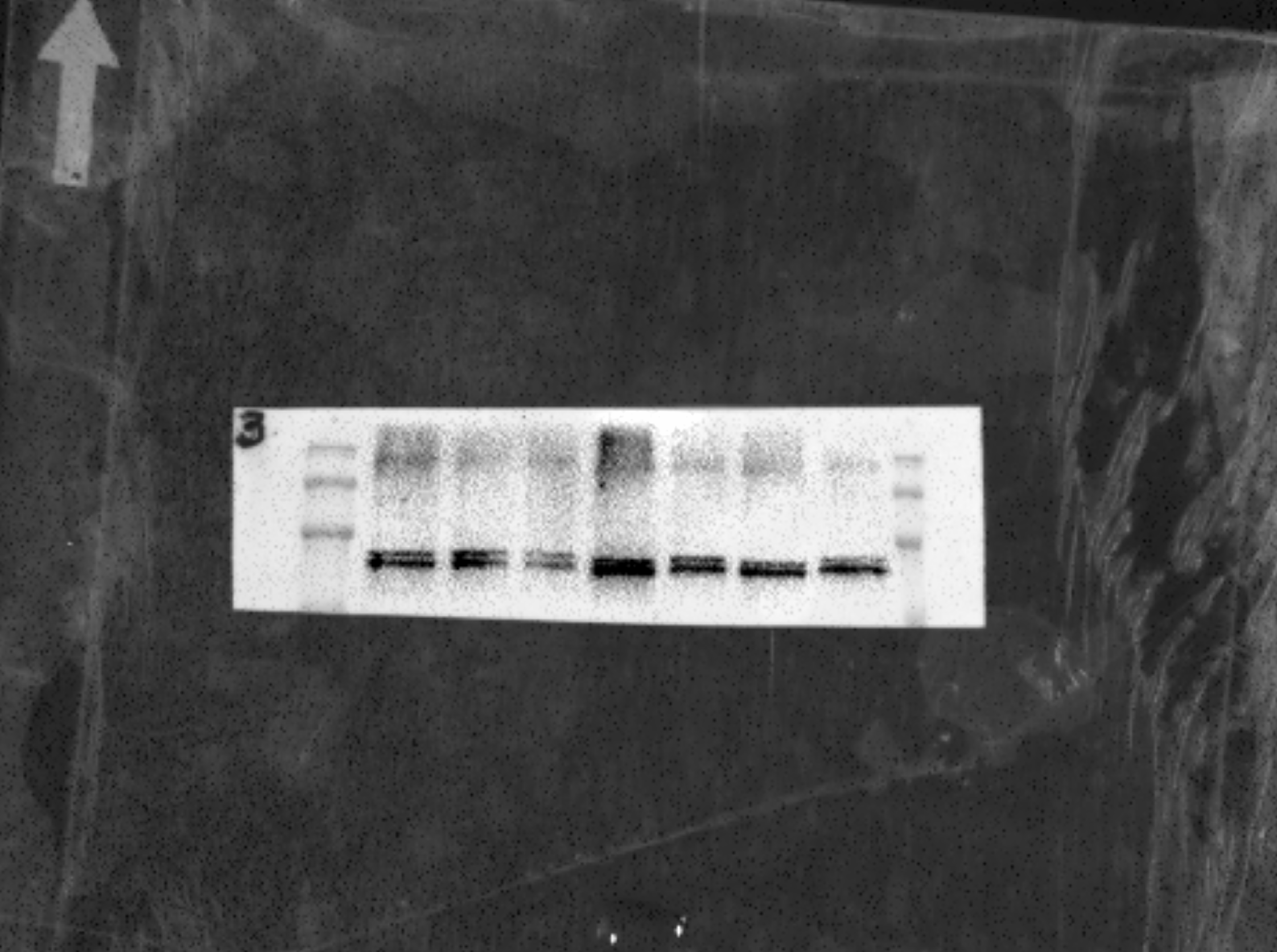

Supplement: Supplemental Information 20 [file peerj-14-21375-s020.zip › Figure 2M WB RAW 0-24h/2KLHL40+MARKER.tif]

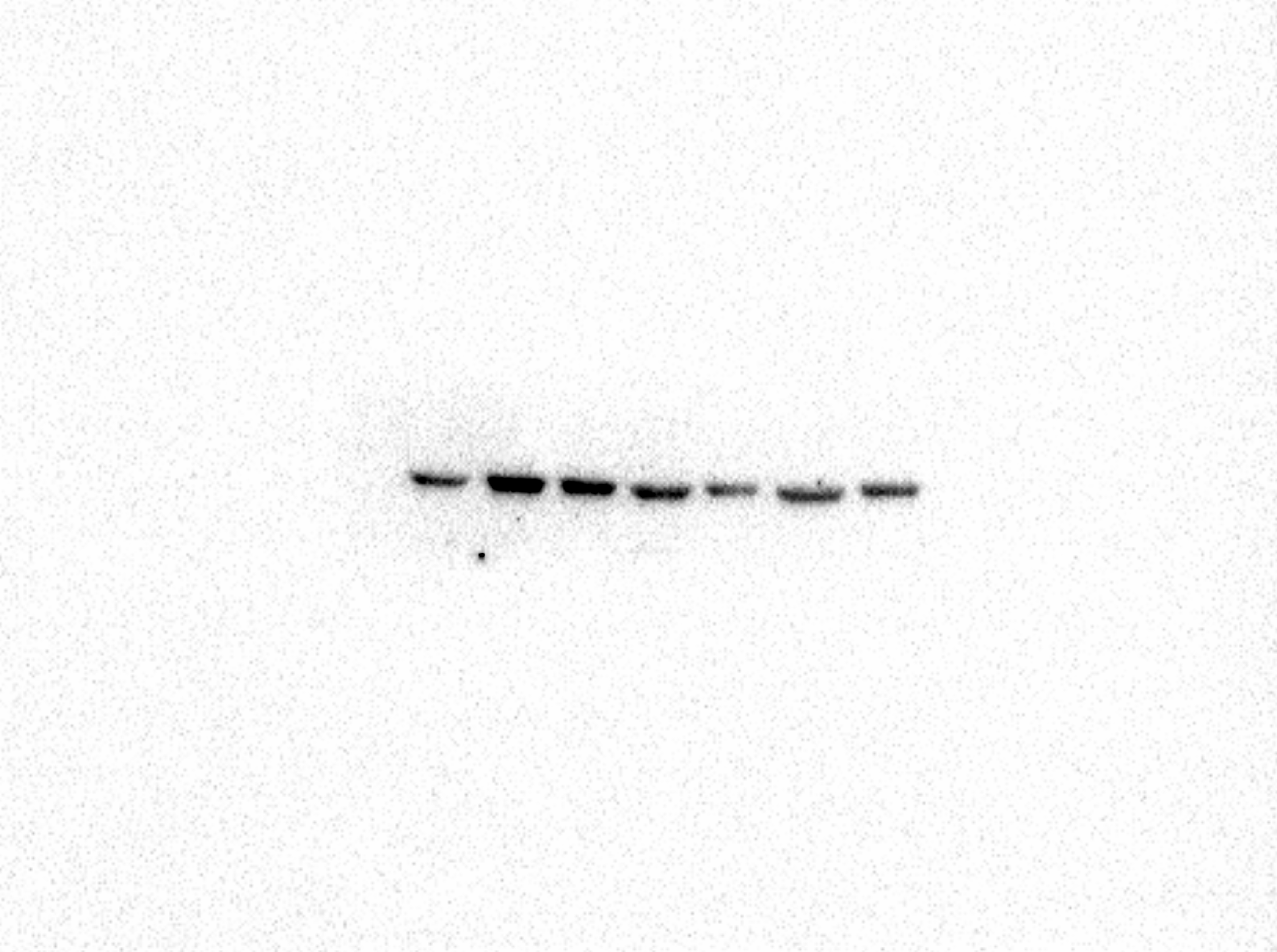

Supplement: Supplemental Information 20 [file peerj-14-21375-s020.zip › Figure 2M WB RAW 0-24h/3ACTIN.tif]

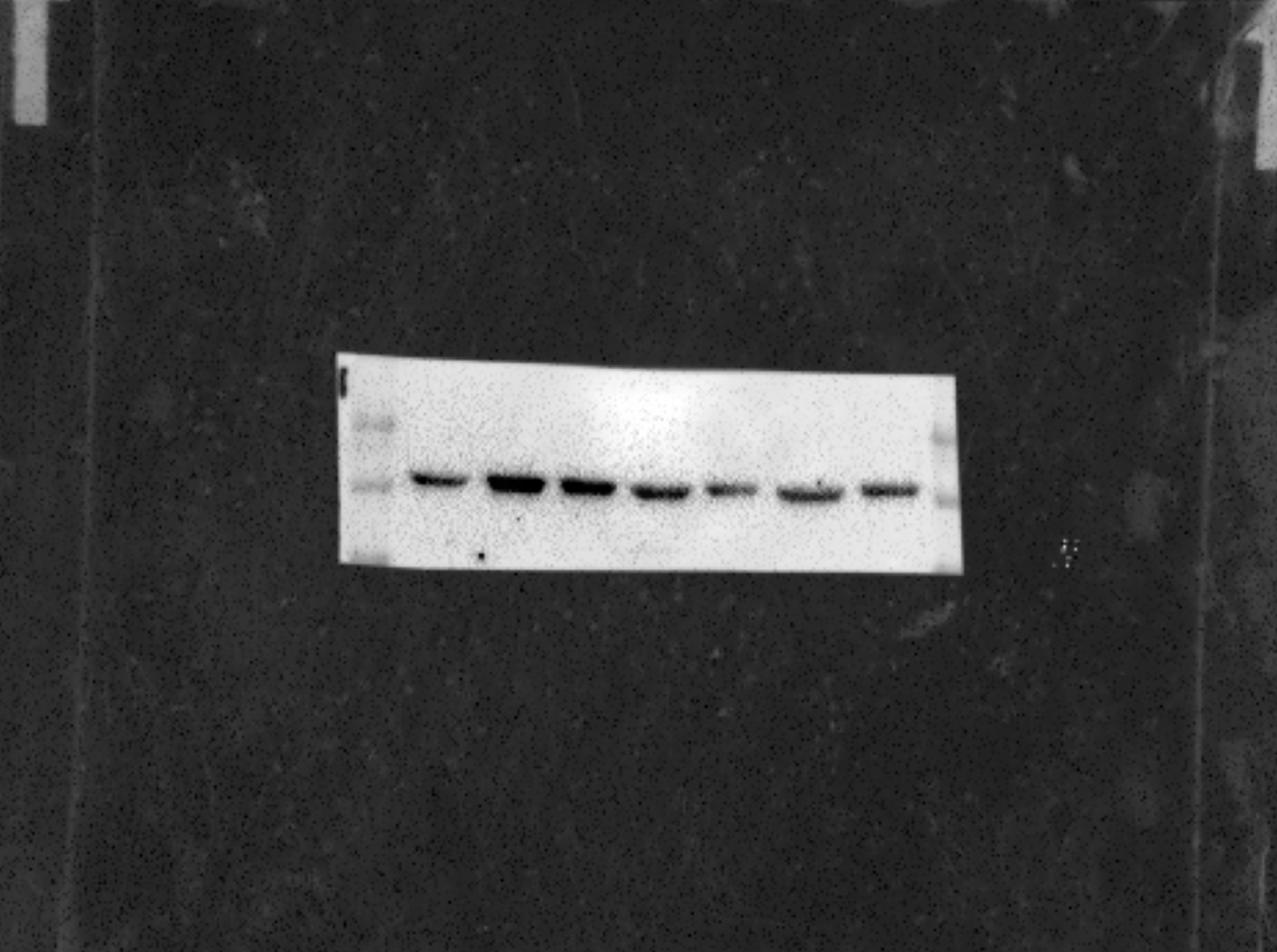

Supplement: Supplemental Information 20 [file peerj-14-21375-s020.zip › Figure 2M WB RAW 0-24h/3ACTIN+MARKER.tif]

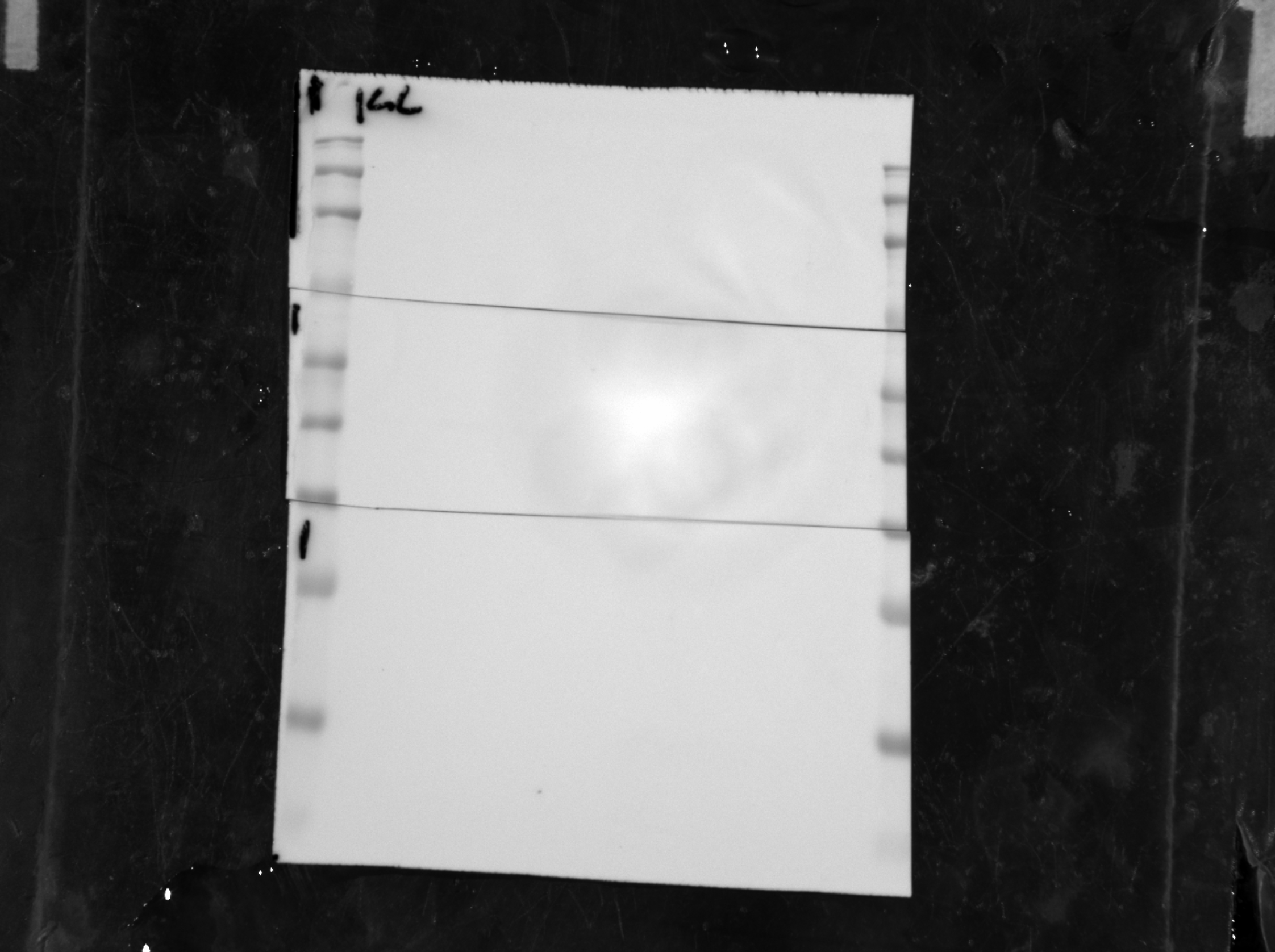

Supplement: Supplemental Information 20 [file peerj-14-21375-s020.zip › Figure 2M WB RAW 0-24h/3ALL.tif]

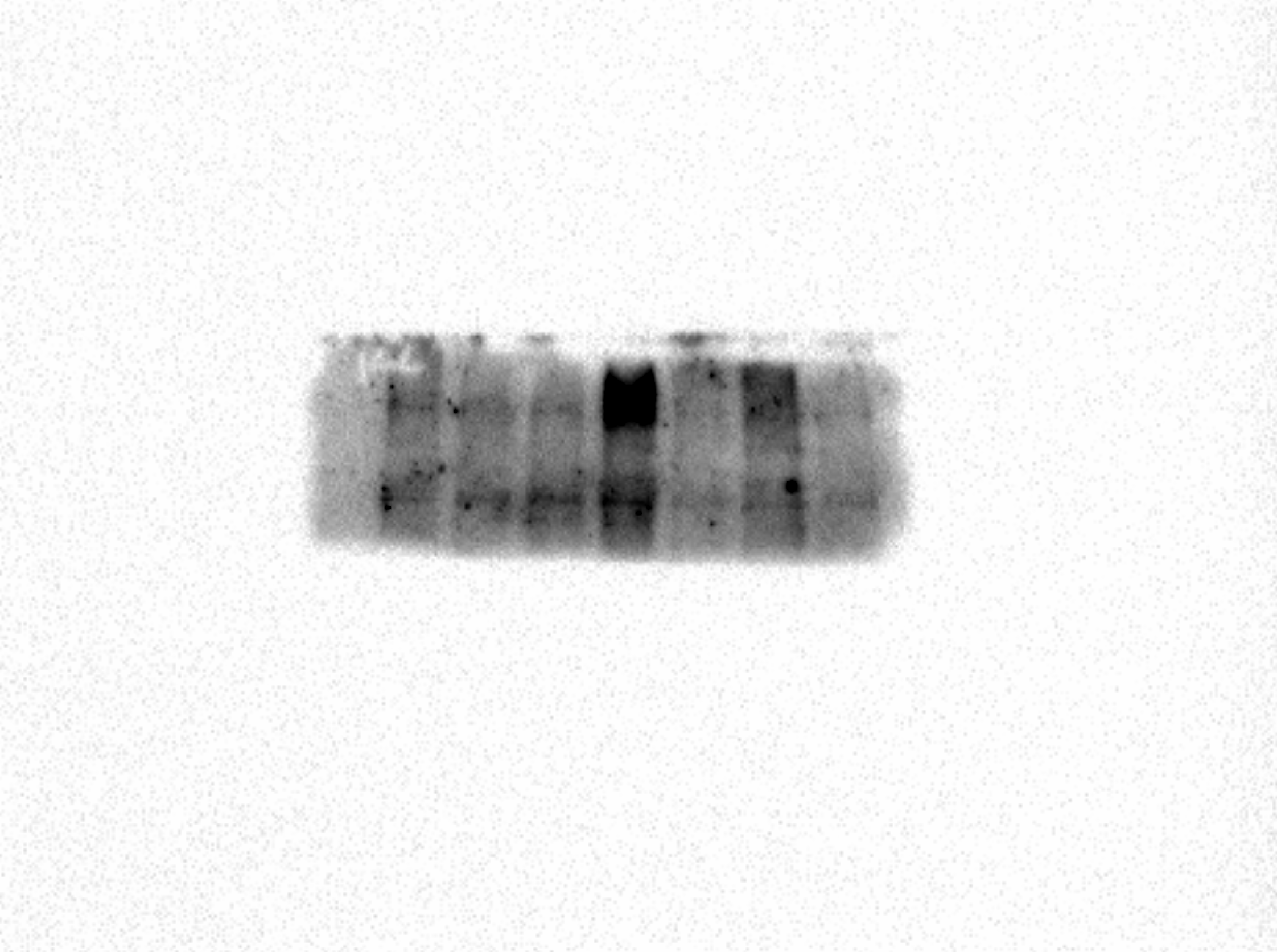

Supplement: Supplemental Information 20 [file peerj-14-21375-s020.zip › Figure 2M WB RAW 0-24h/3KLHL40.tif]

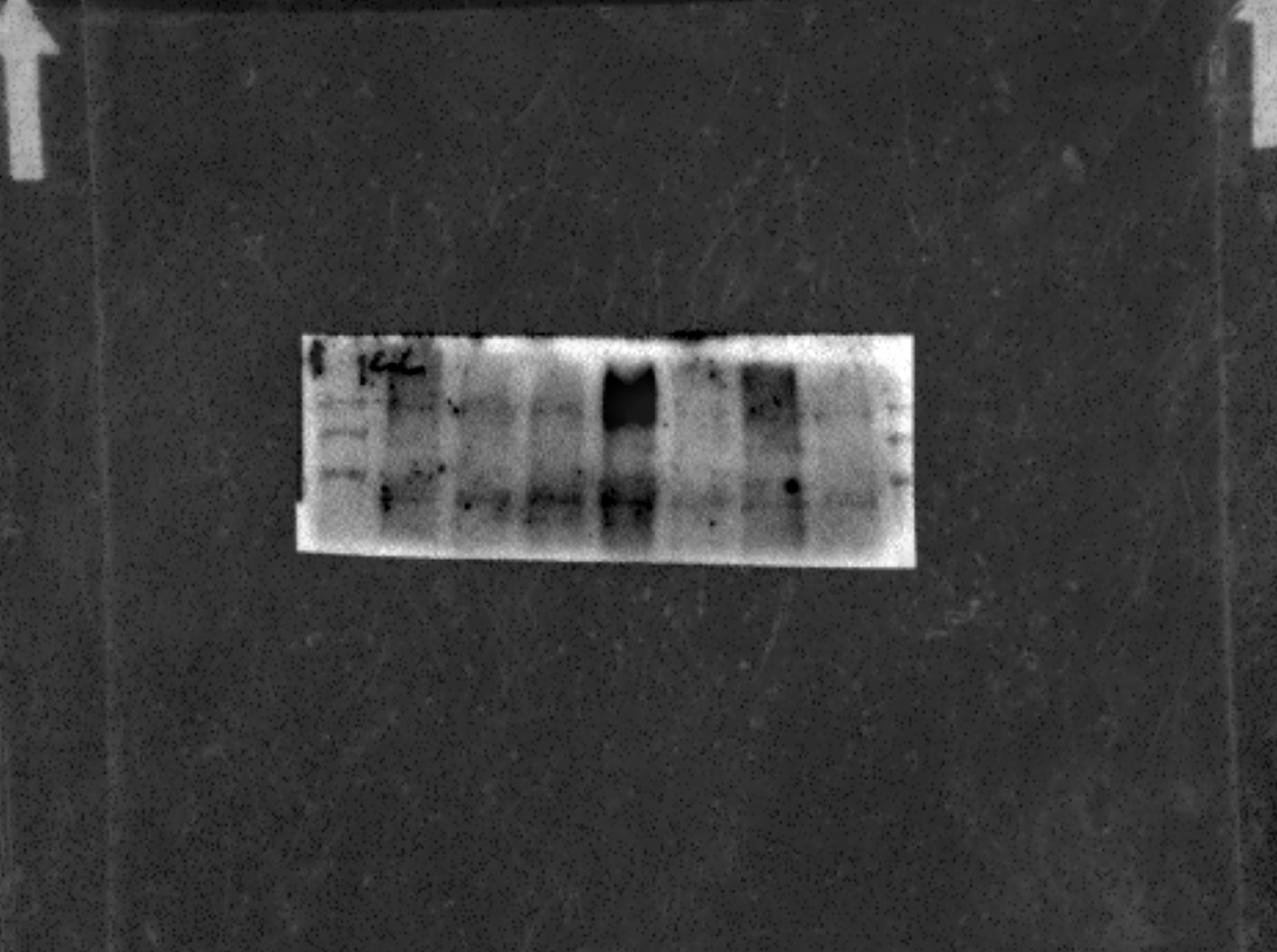

Supplement: Supplemental Information 20 [file peerj-14-21375-s020.zip › Figure 2M WB RAW 0-24h/3KLHL40+MARKER.tif]

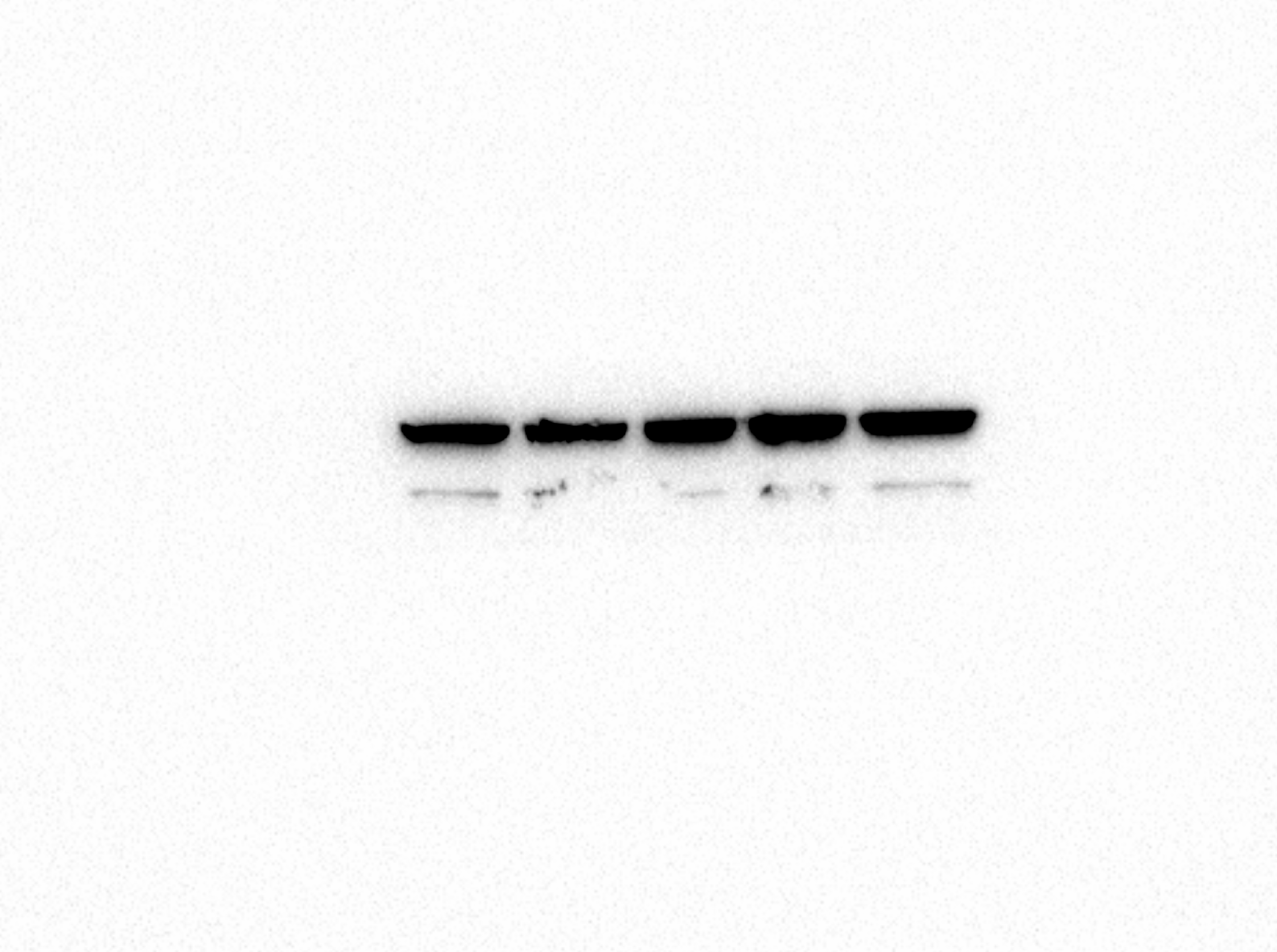

Supplement: Supplemental Information 21 [file peerj-14-21375-s021.zip › Figure 3H WB RAW SH-KLHL40/1ACTIN.tif]

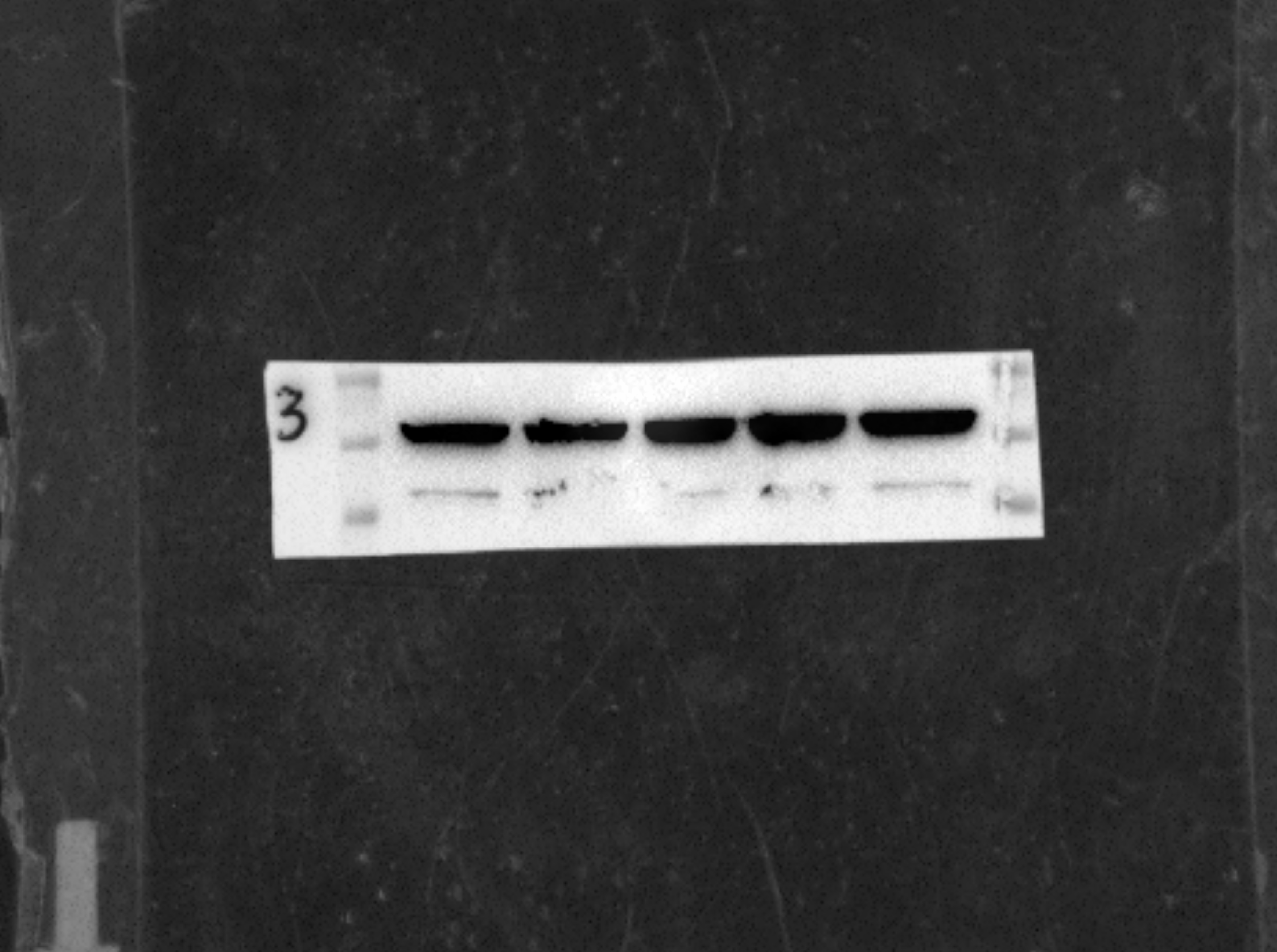

Supplement: Supplemental Information 21 [file peerj-14-21375-s021.zip › Figure 3H WB RAW SH-KLHL40/1ACTIN+MARKER.tif]

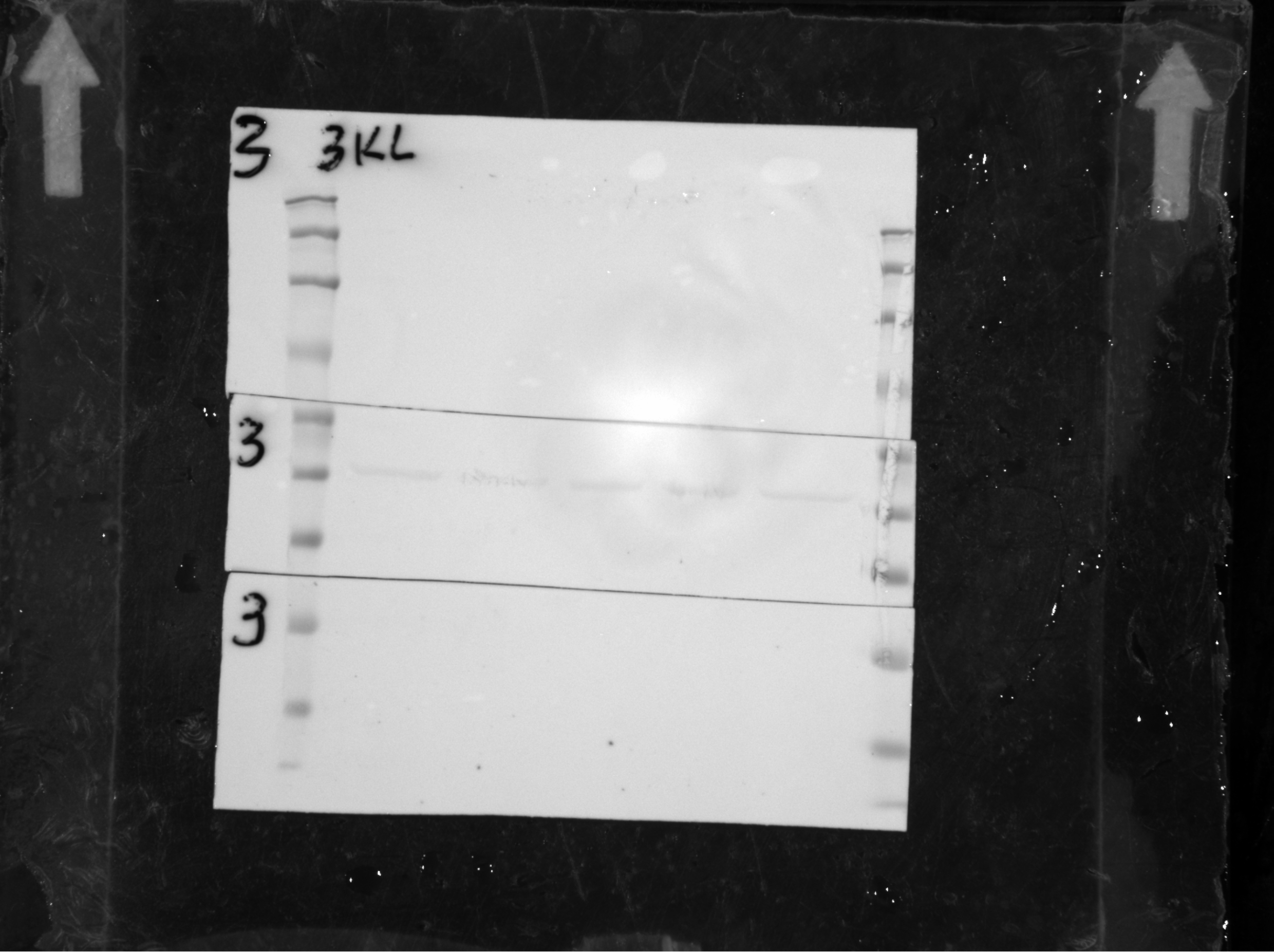

Supplement: Supplemental Information 21 [file peerj-14-21375-s021.zip › Figure 3H WB RAW SH-KLHL40/1ALL.tif]

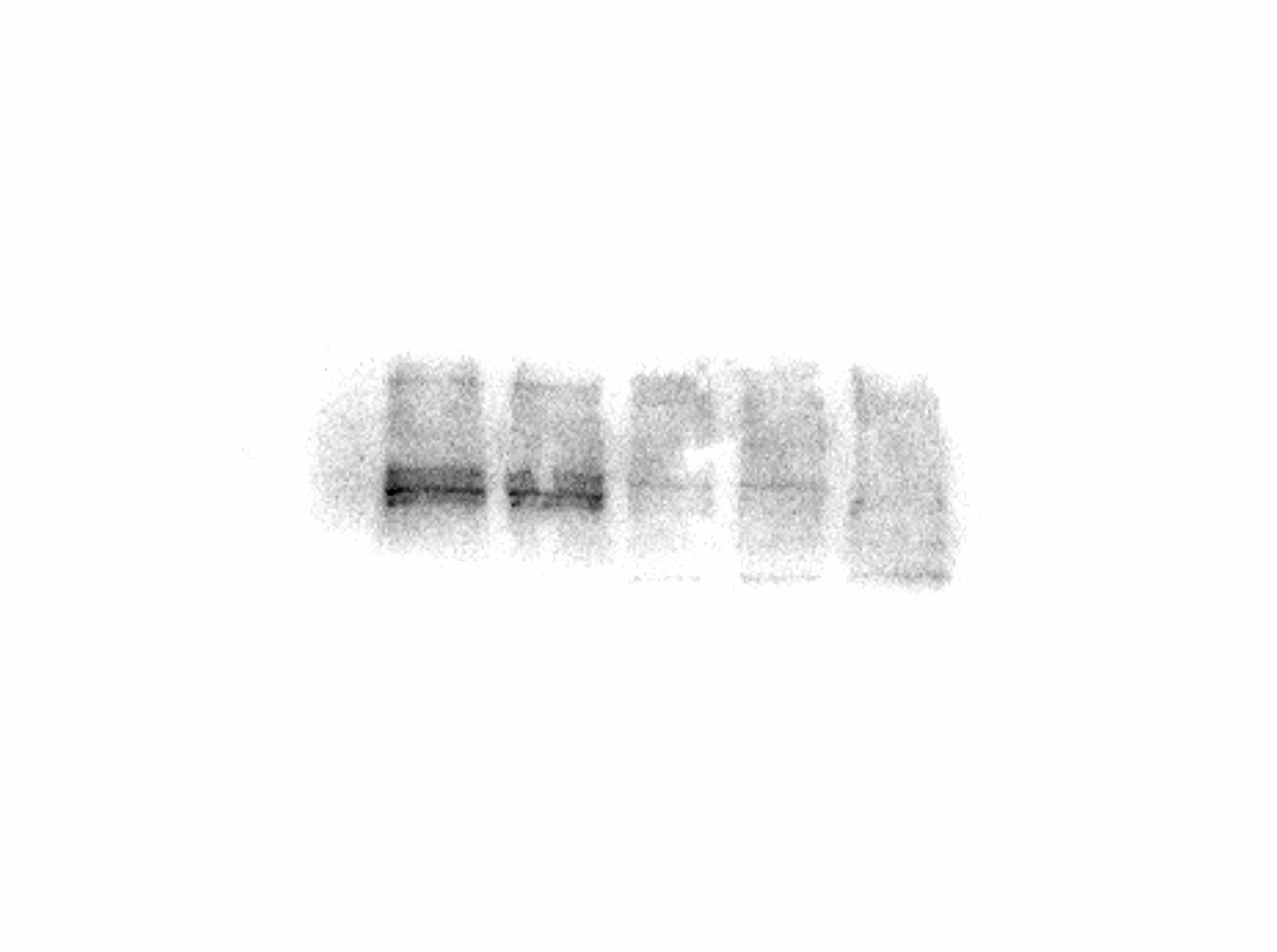

Supplement: Supplemental Information 21 [file peerj-14-21375-s021.zip › Figure 3H WB RAW SH-KLHL40/1KLHL40.tif]

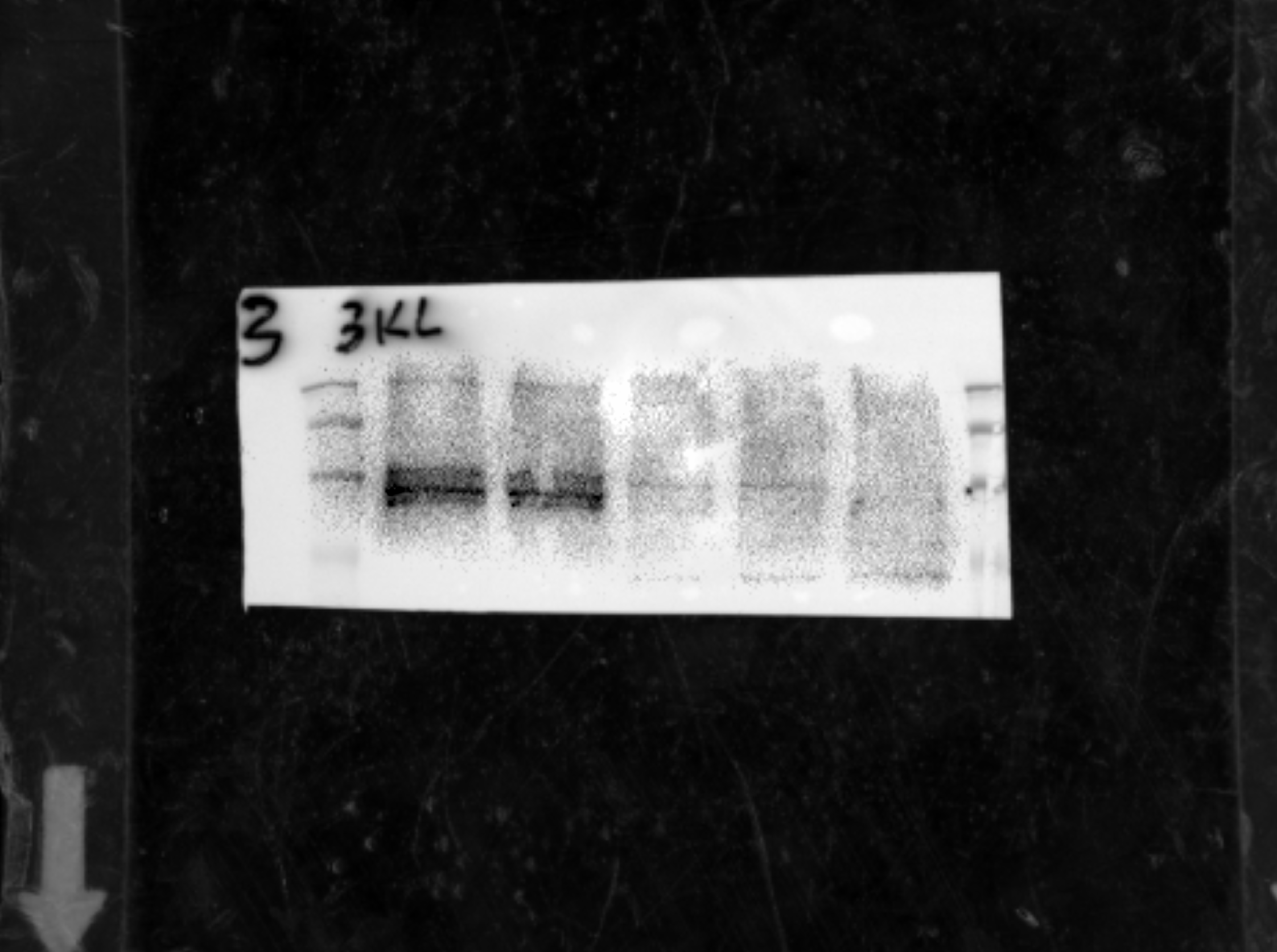

Supplement: Supplemental Information 21 [file peerj-14-21375-s021.zip › Figure 3H WB RAW SH-KLHL40/1KLHL40+MARKER.tif]

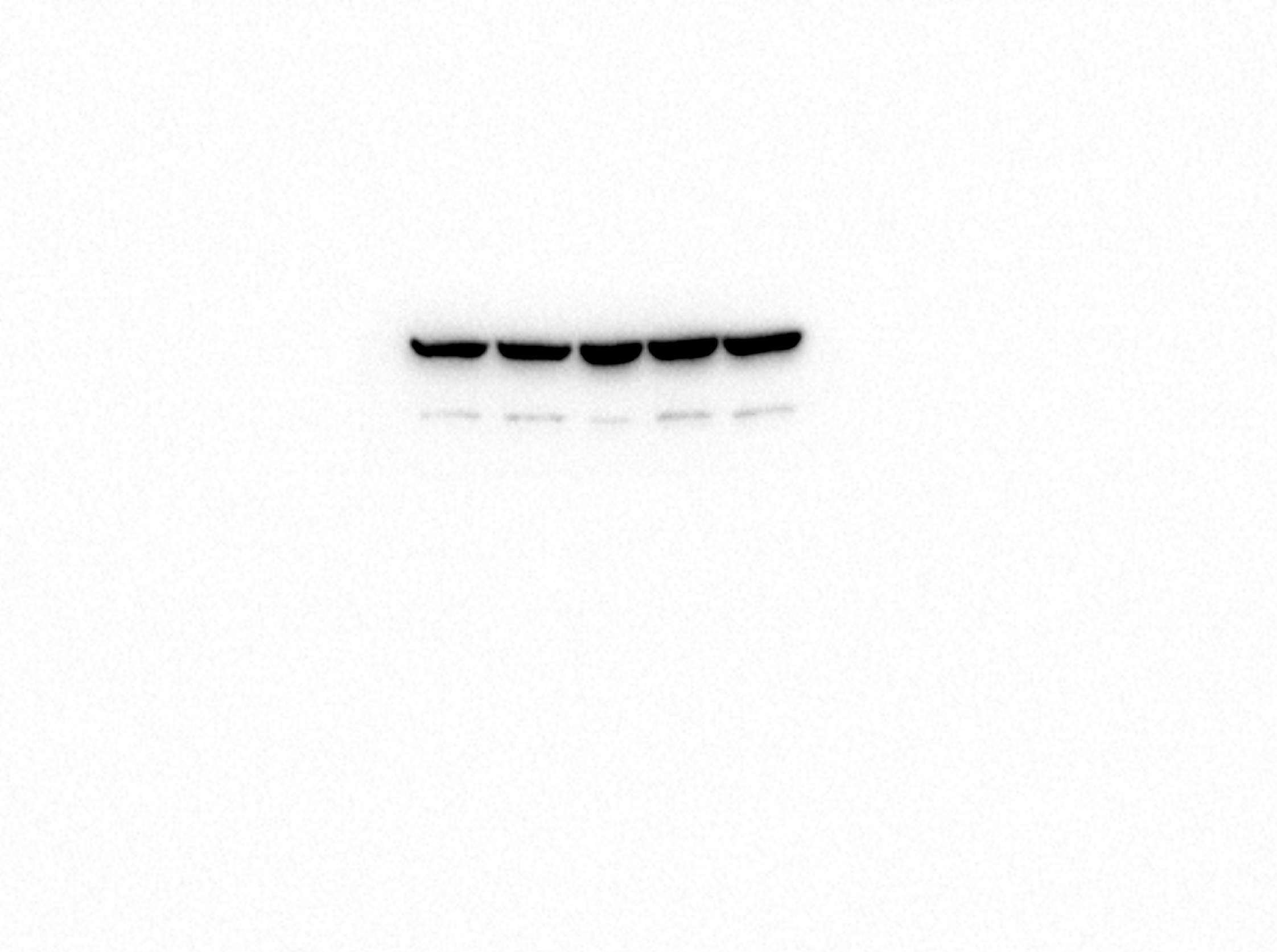

Supplement: Supplemental Information 21 [file peerj-14-21375-s021.zip › Figure 3H WB RAW SH-KLHL40/2ACTIN.tif]

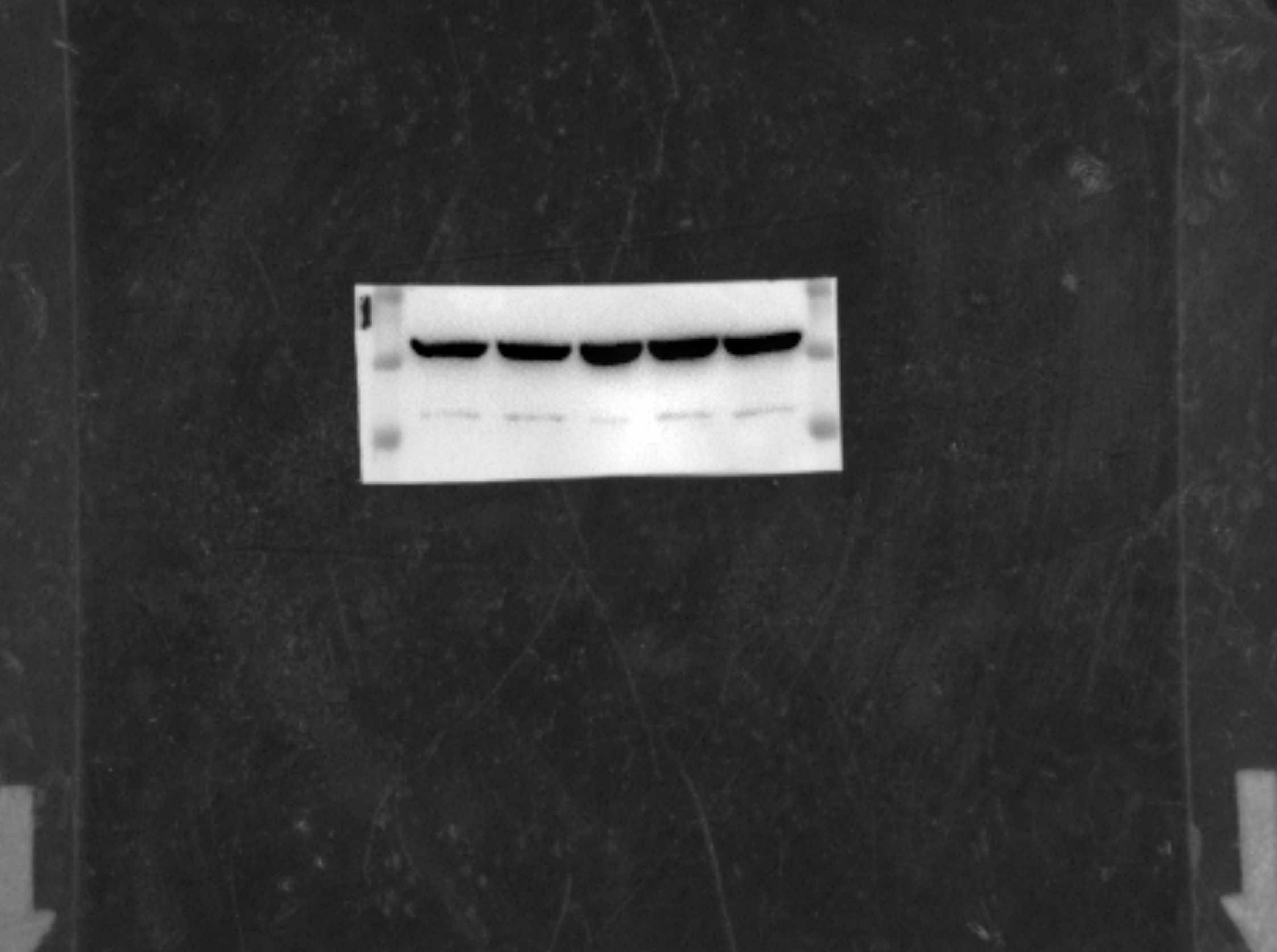

Supplement: Supplemental Information 21 [file peerj-14-21375-s021.zip › Figure 3H WB RAW SH-KLHL40/2ACTIN-MARKER.tif]

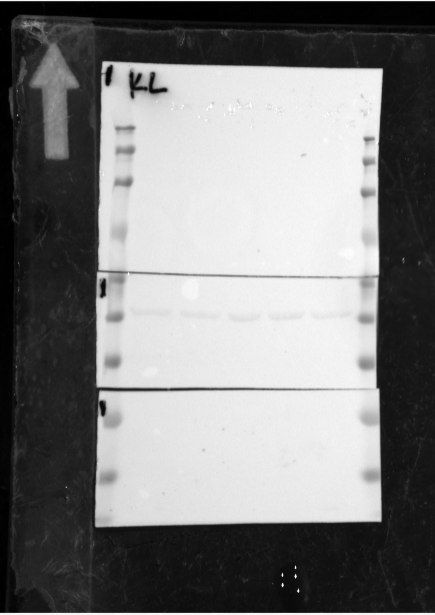

Supplement: Supplemental Information 21 [file peerj-14-21375-s021.zip › Figure 3H WB RAW SH-KLHL40/2ALL.png]

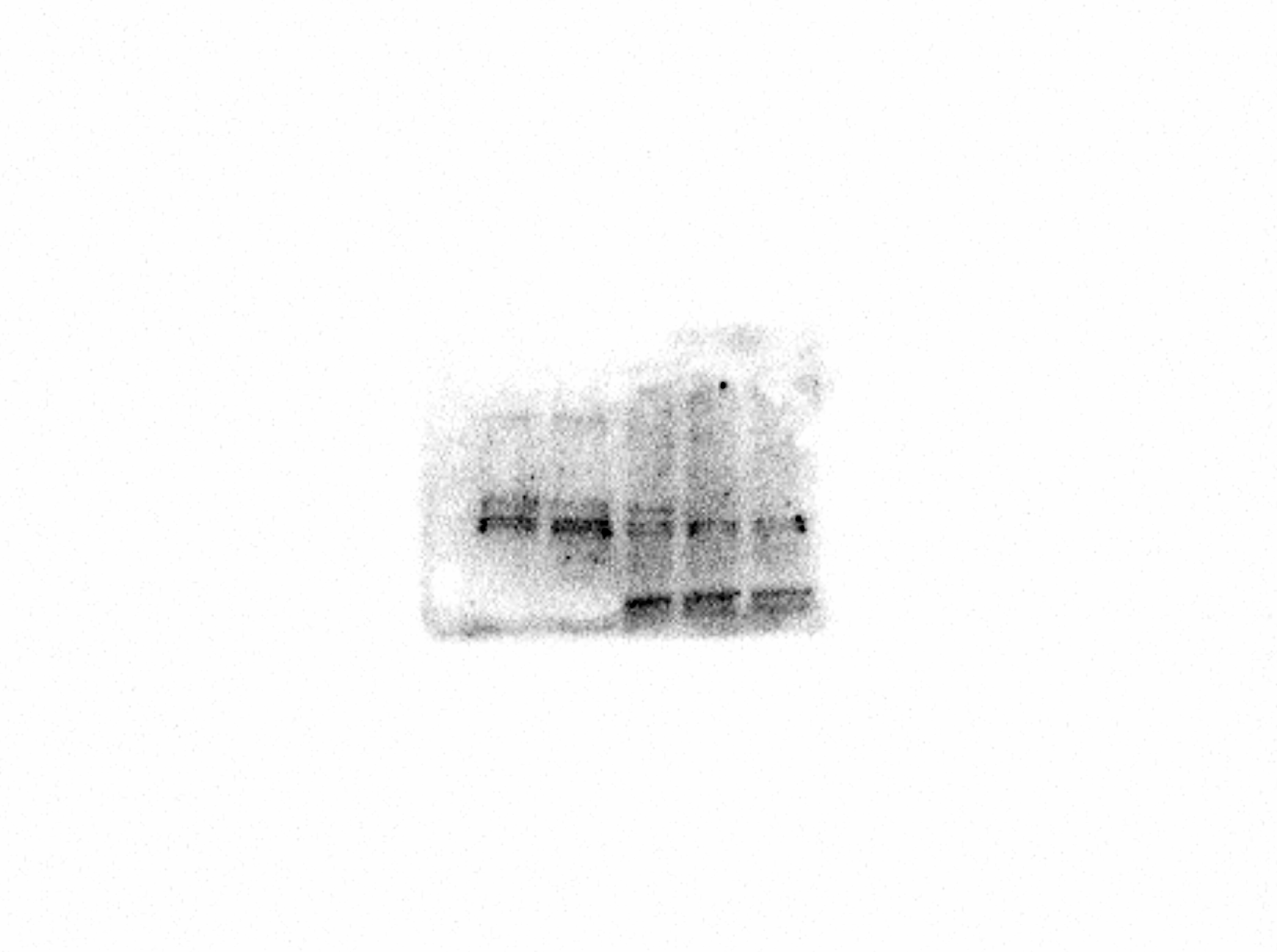

Supplement: Supplemental Information 21 [file peerj-14-21375-s021.zip › Figure 3H WB RAW SH-KLHL40/2KLHL40.tif]

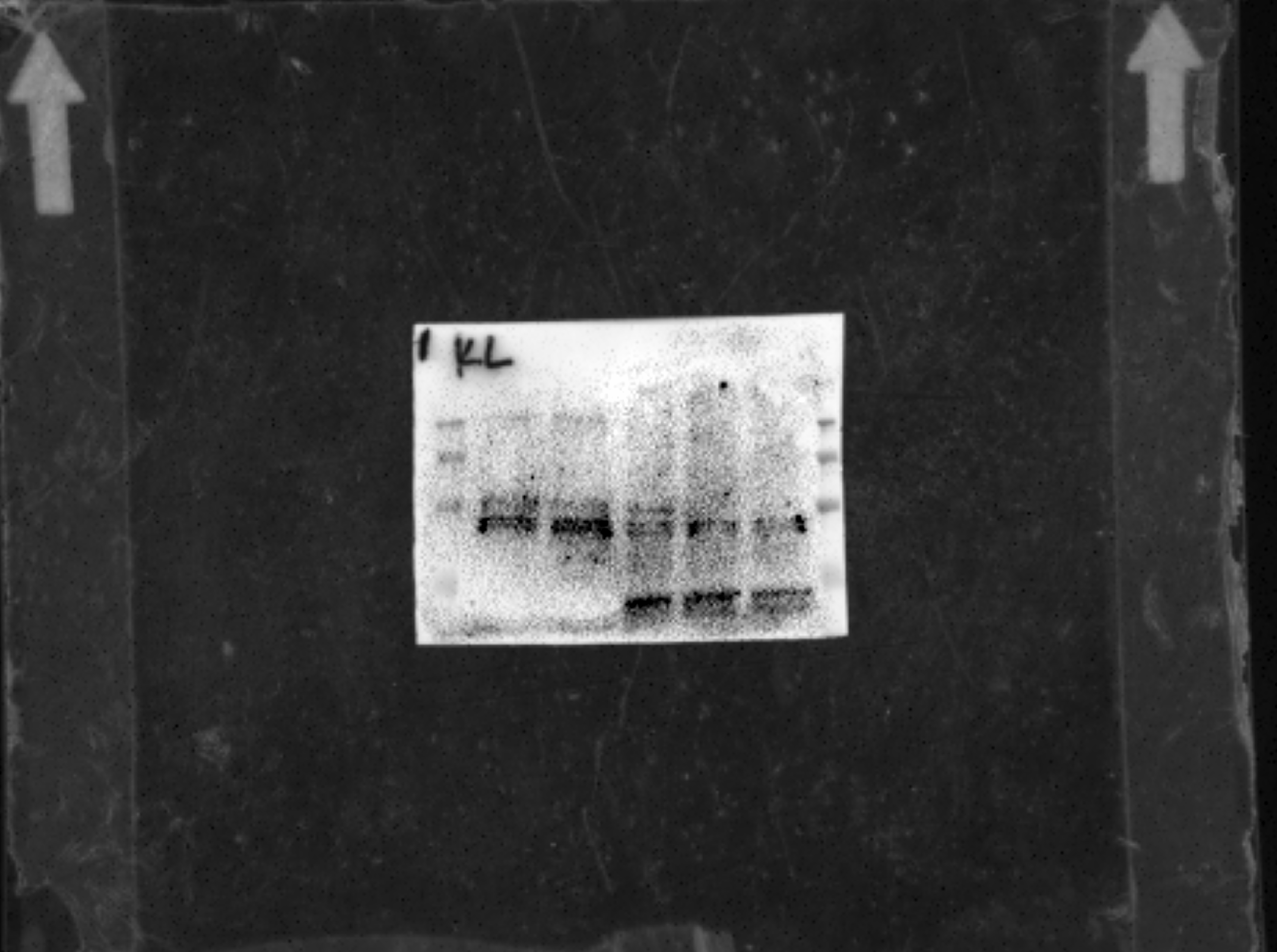

Supplement: Supplemental Information 21 [file peerj-14-21375-s021.zip › Figure 3H WB RAW SH-KLHL40/2KLHL40+MARKER.tif]

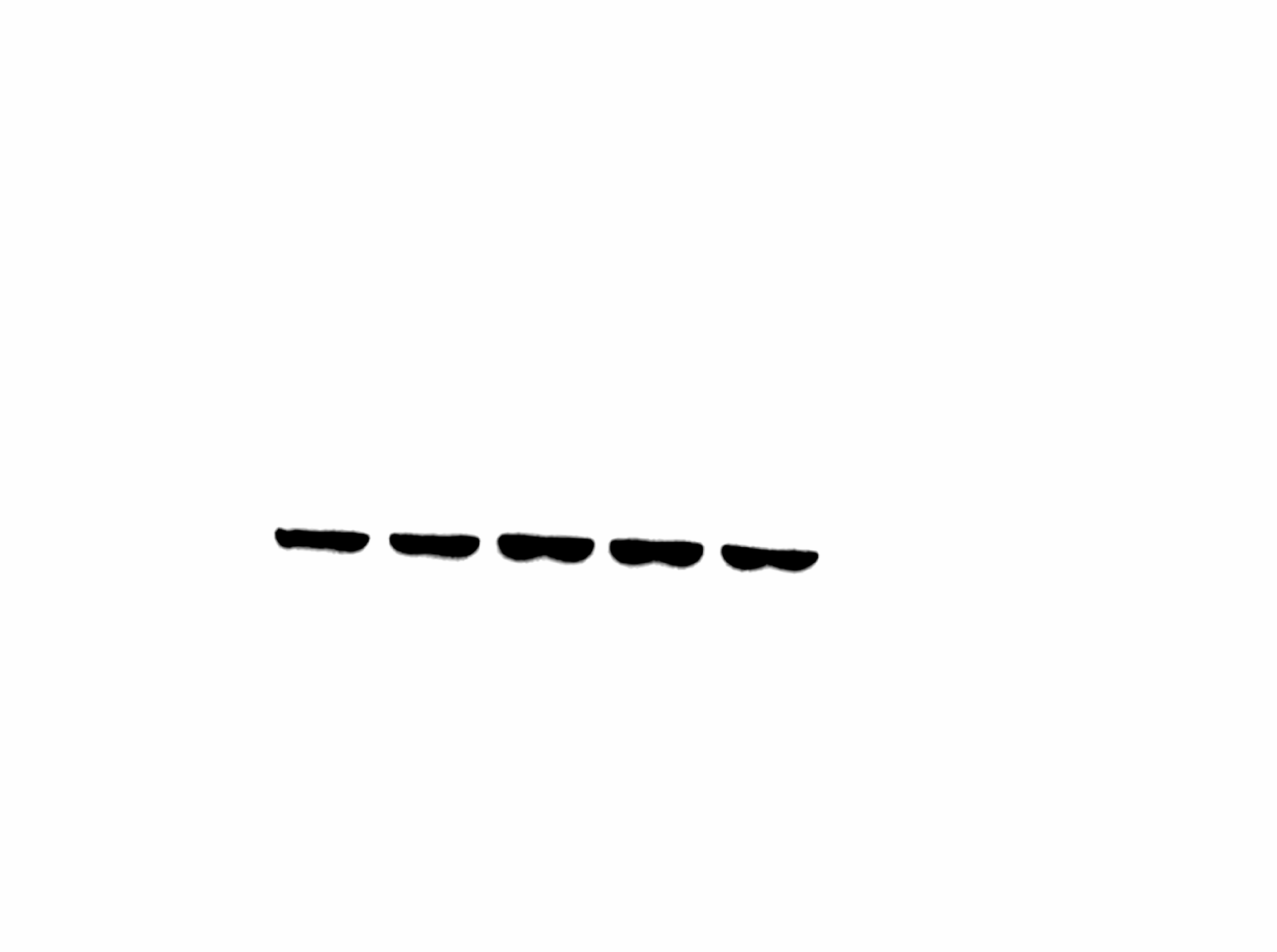

Supplement: Supplemental Information 21 [file peerj-14-21375-s021.zip › Figure 3H WB RAW SH-KLHL40/3ACTIN.tif]

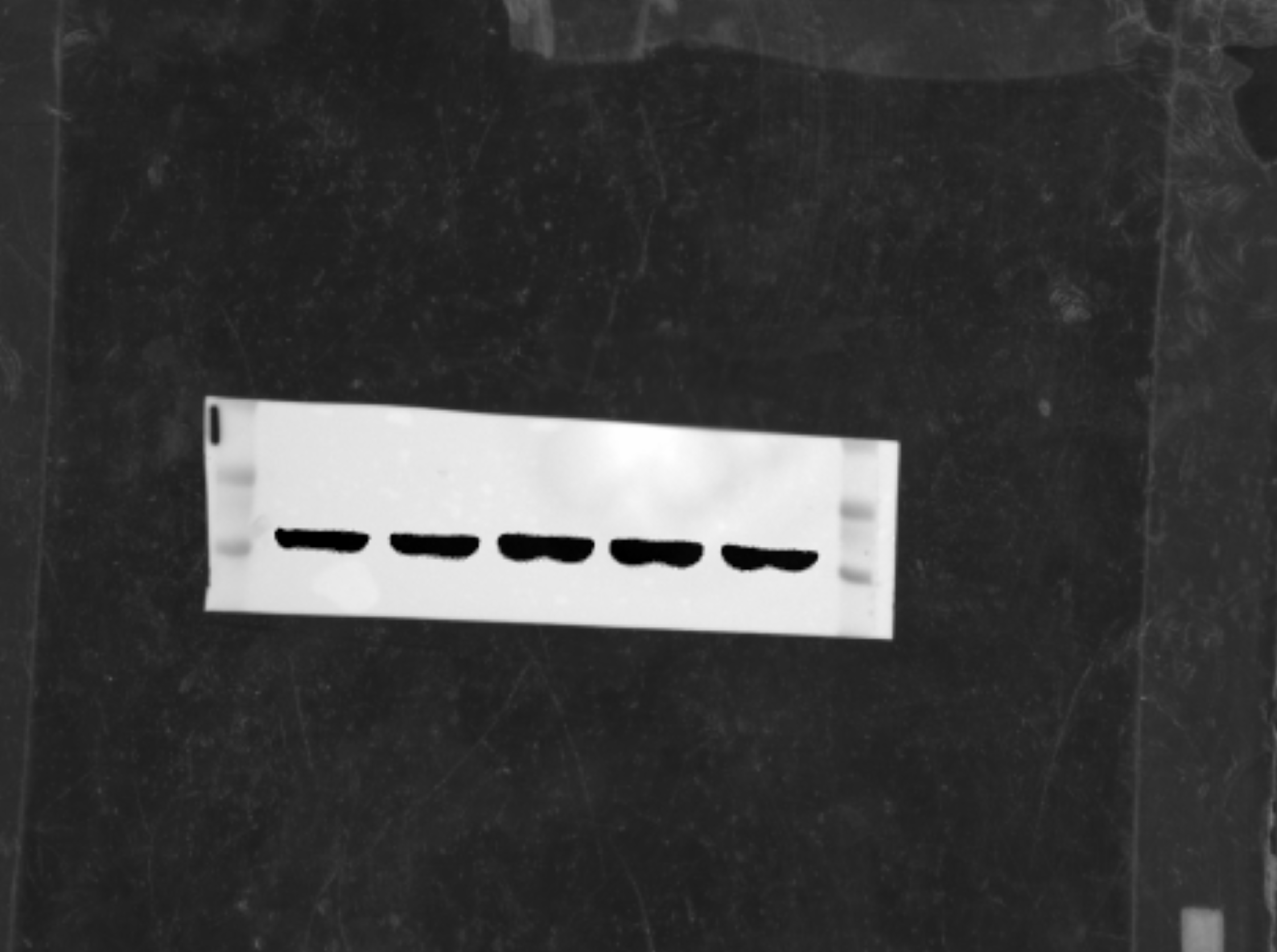

Supplement: Supplemental Information 21 [file peerj-14-21375-s021.zip › Figure 3H WB RAW SH-KLHL40/3ACTIN+MARKER.tif]

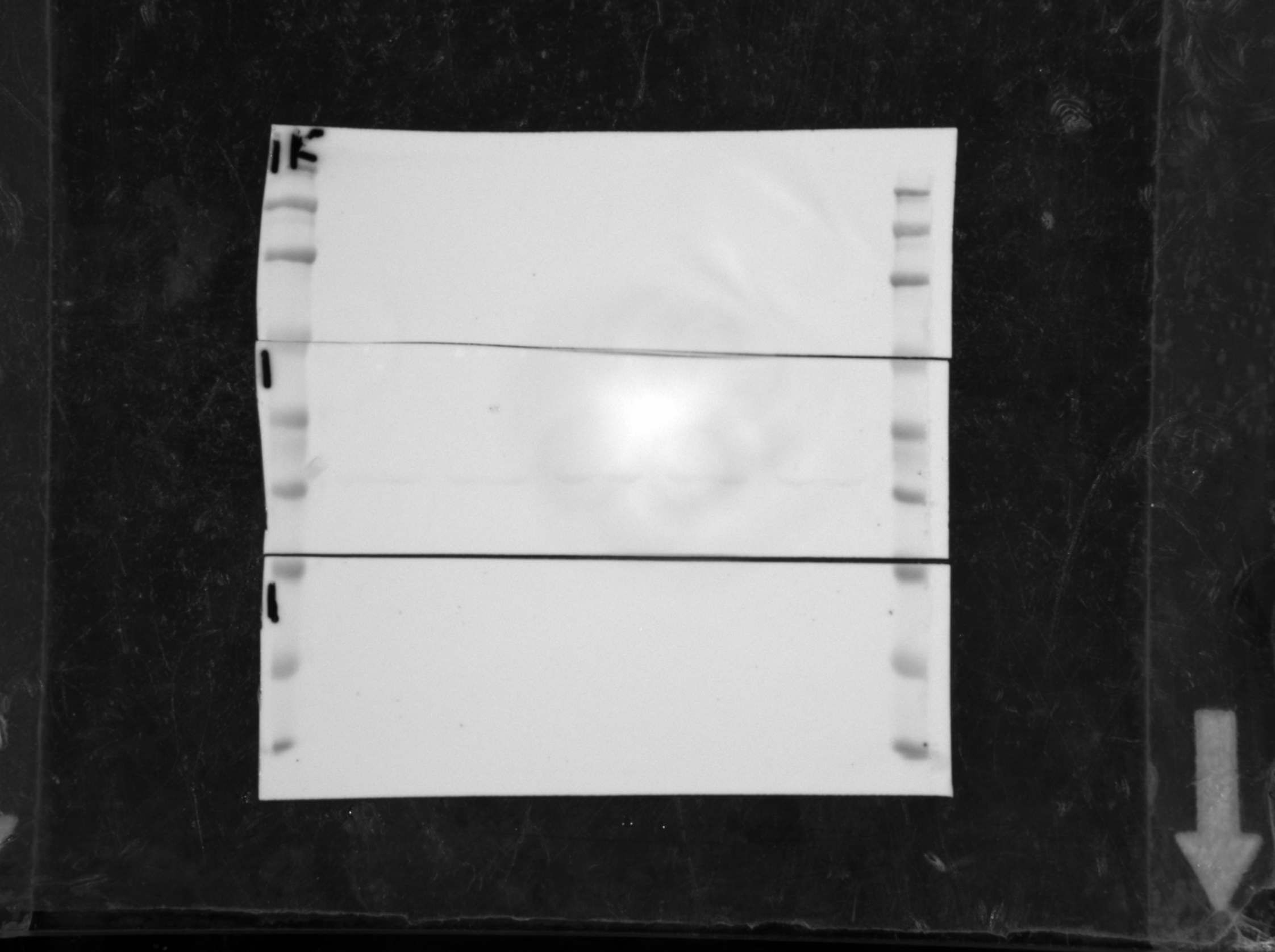

Supplement: Supplemental Information 21 [file peerj-14-21375-s021.zip › Figure 3H WB RAW SH-KLHL40/3ALL.tif]

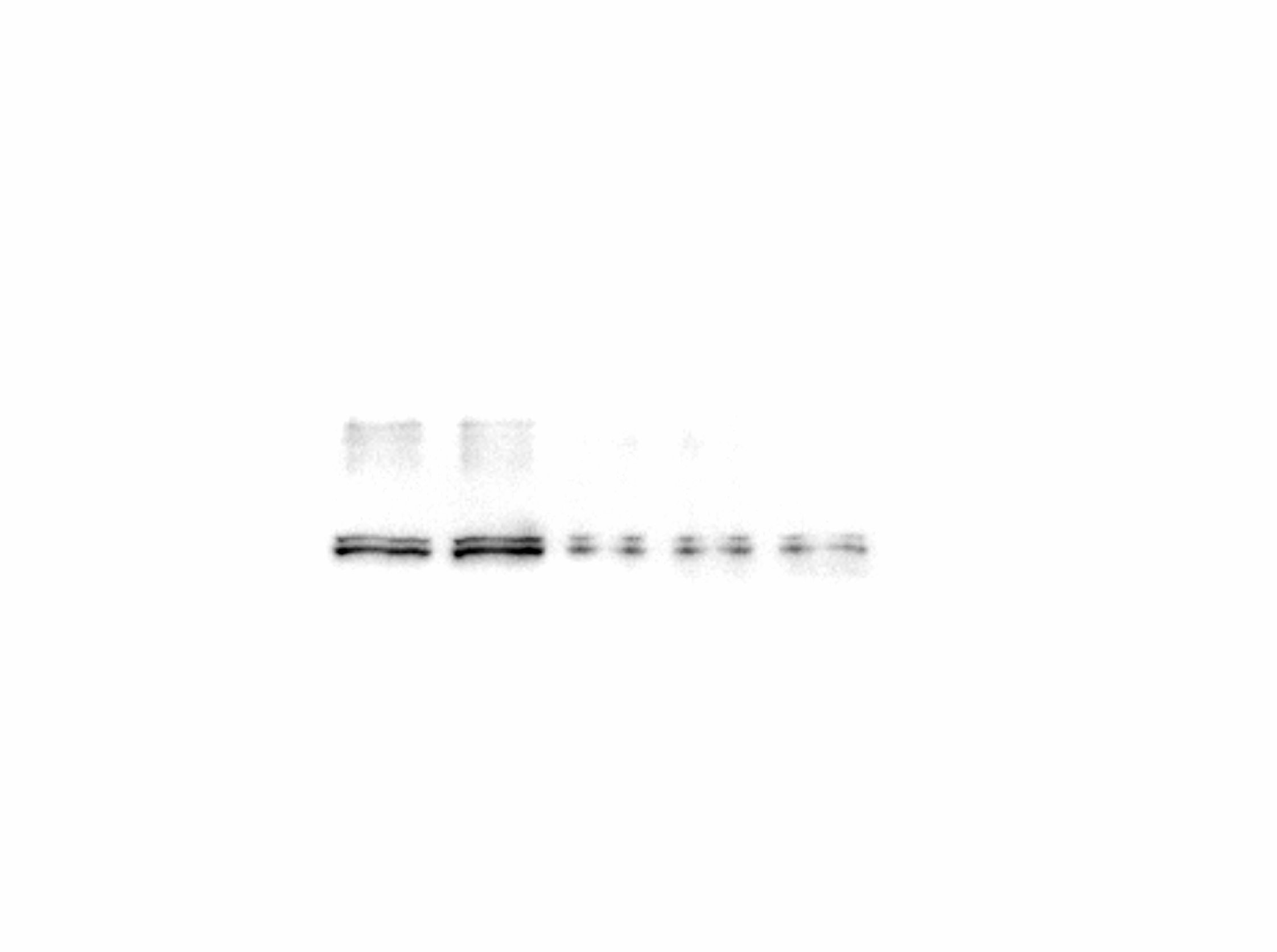

Supplement: Supplemental Information 21 [file peerj-14-21375-s021.zip › Figure 3H WB RAW SH-KLHL40/3KLHL40.tif]

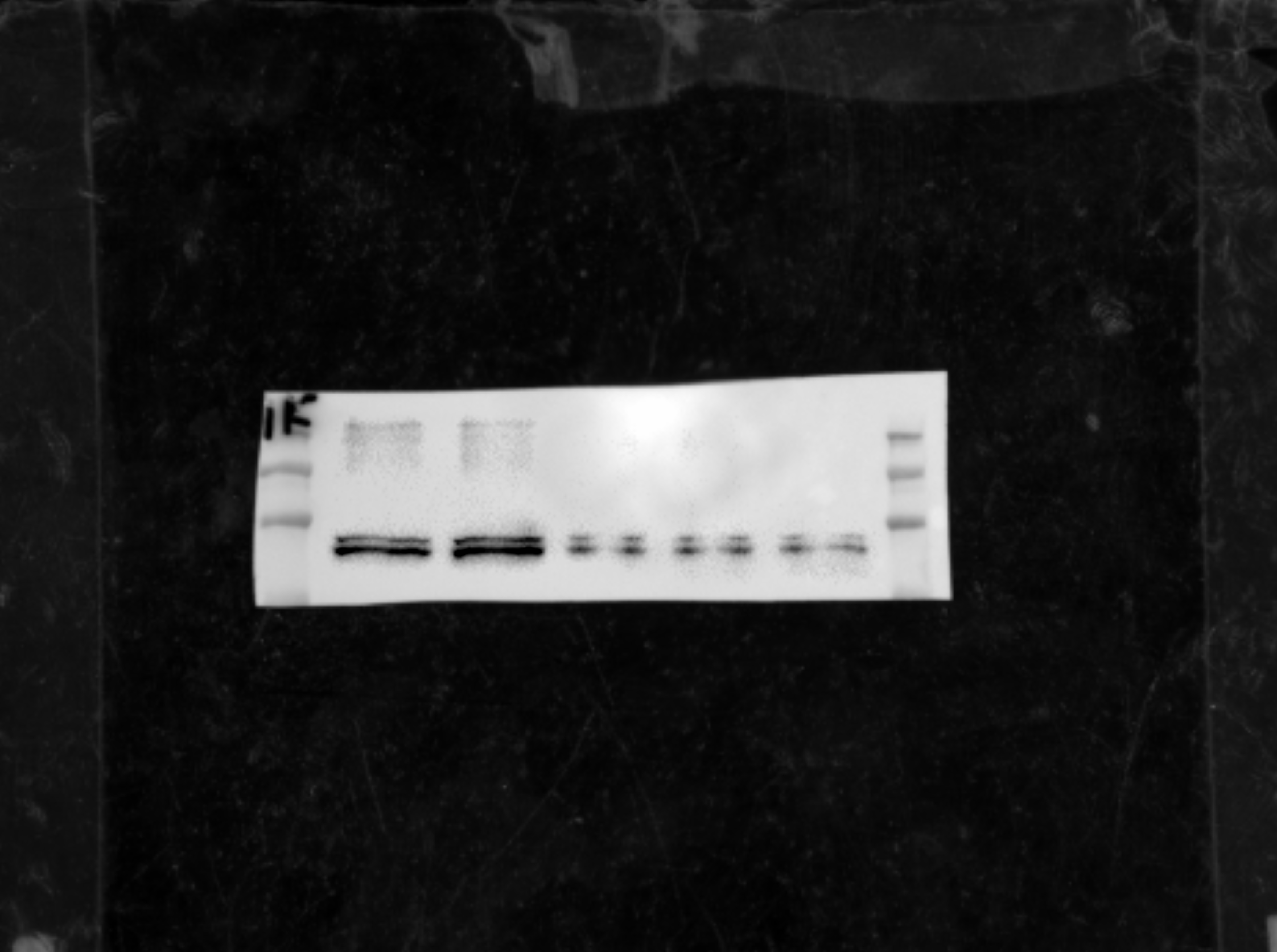

Supplement: Supplemental Information 21 [file peerj-14-21375-s021.zip › Figure 3H WB RAW SH-KLHL40/3KLHL40+MARKER.tif]

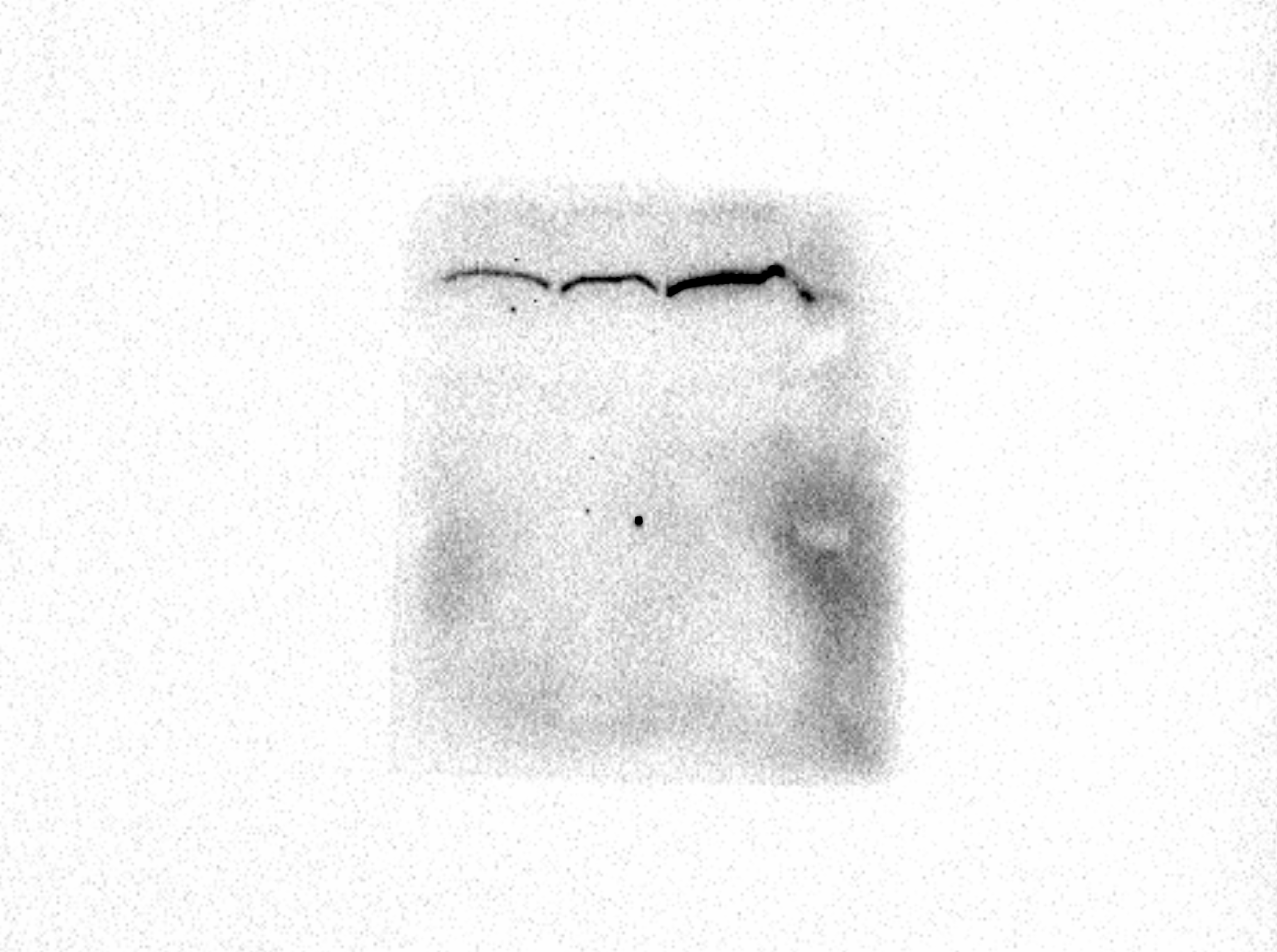

Supplement: Supplemental Information 22 [file peerj-14-21375-s022.zip › Figure 3J WB RAW OE-KLHL40/KLHL40-1.tif]

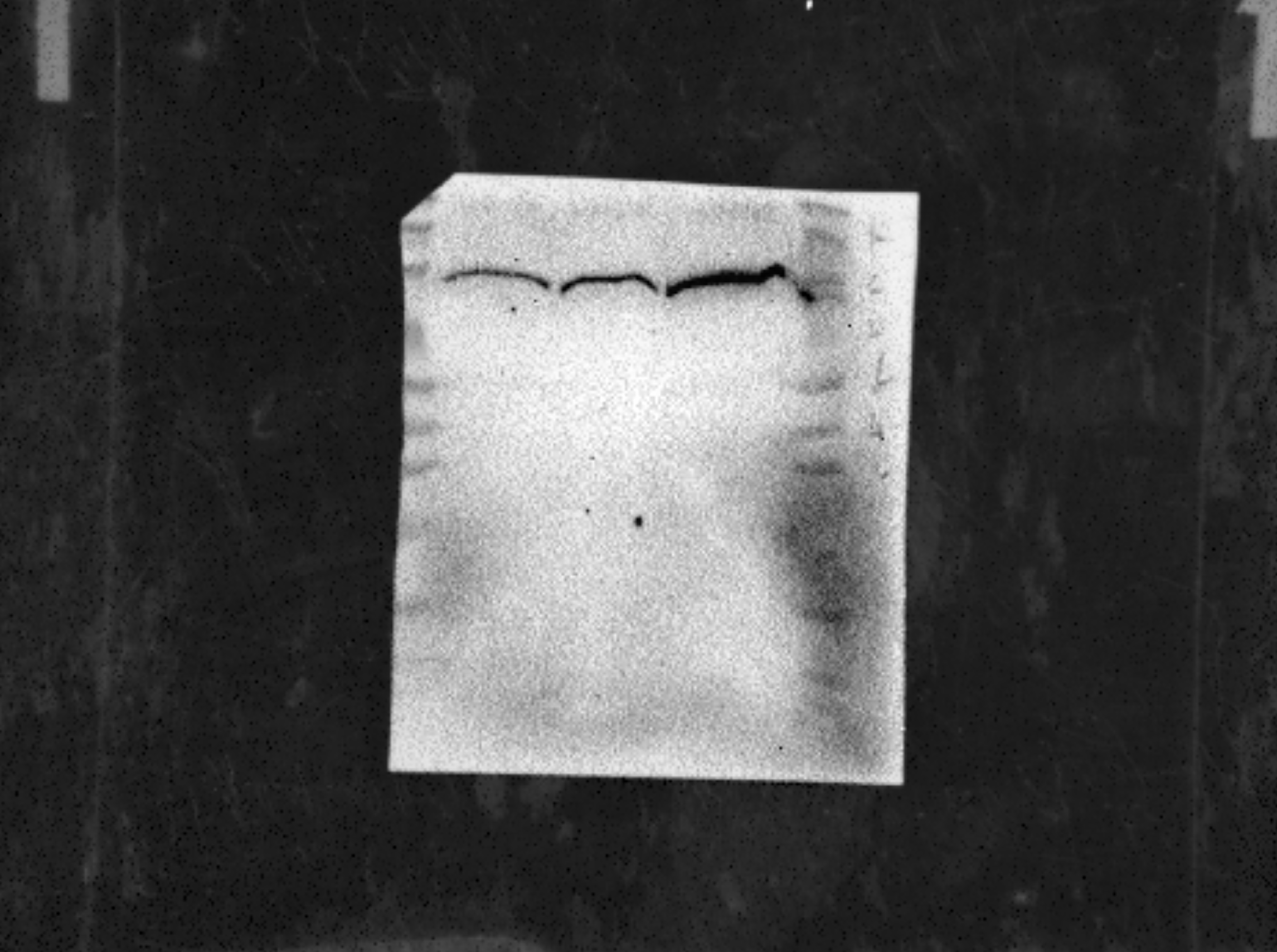

Supplement: Supplemental Information 22 [file peerj-14-21375-s022.zip › Figure 3J WB RAW OE-KLHL40/KLHL40-1+MARK.tif]

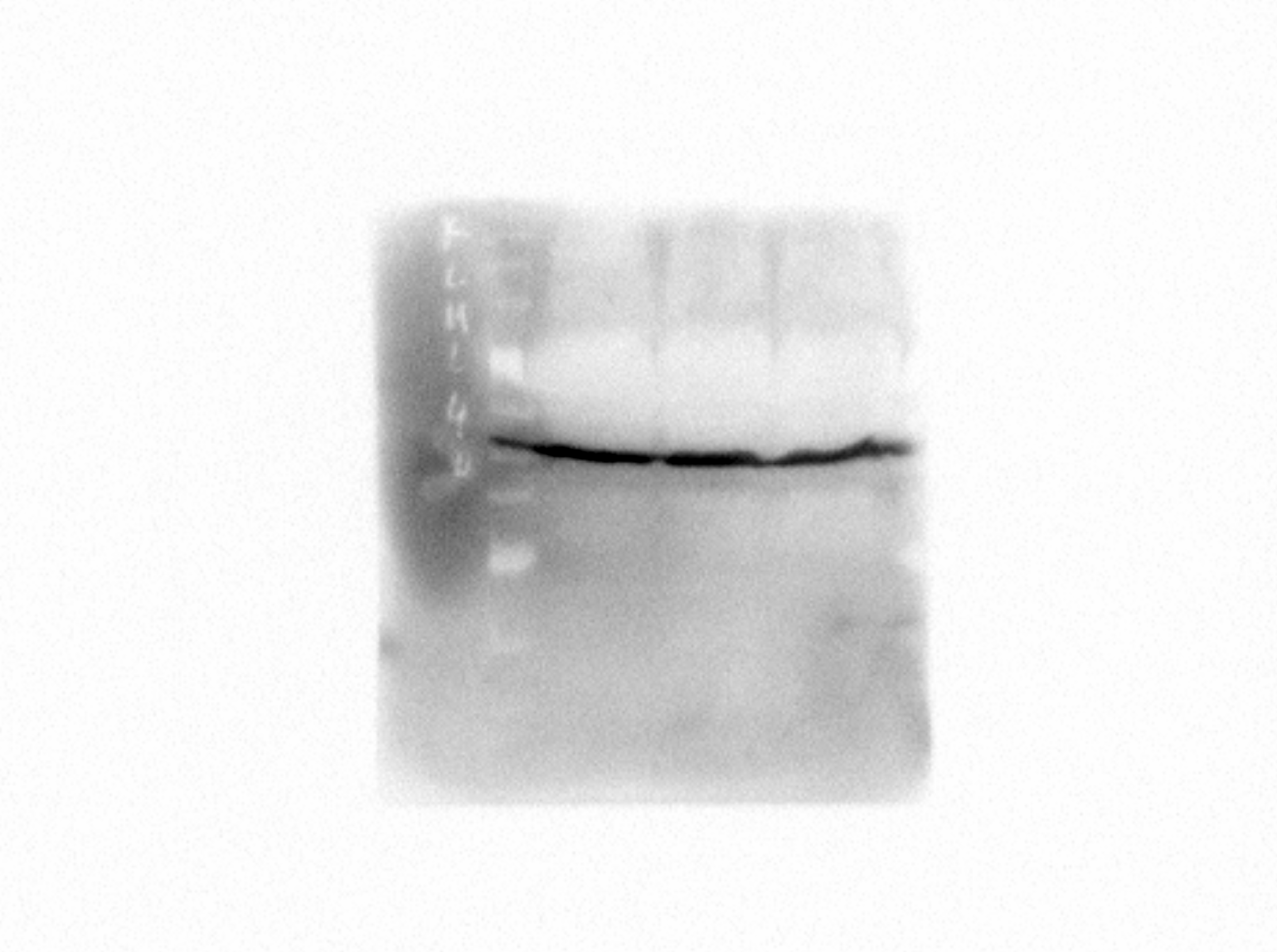

Supplement: Supplemental Information 22 [file peerj-14-21375-s022.zip › Figure 3J WB RAW OE-KLHL40/KLHL40-1-ACTB.tif]

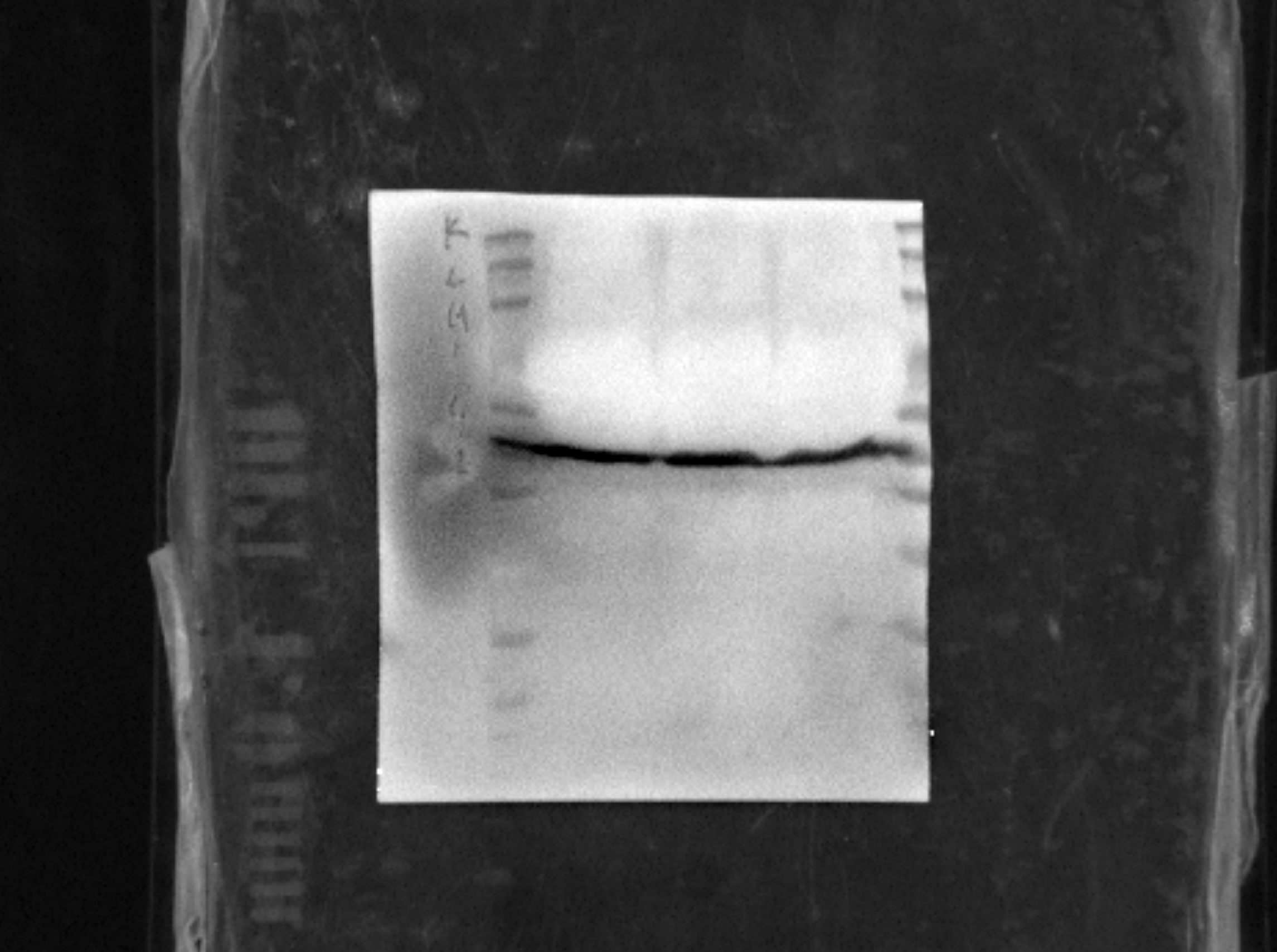

Supplement: Supplemental Information 22 [file peerj-14-21375-s022.zip › Figure 3J WB RAW OE-KLHL40/KLHL40-1-ACTB+MARK.tif]

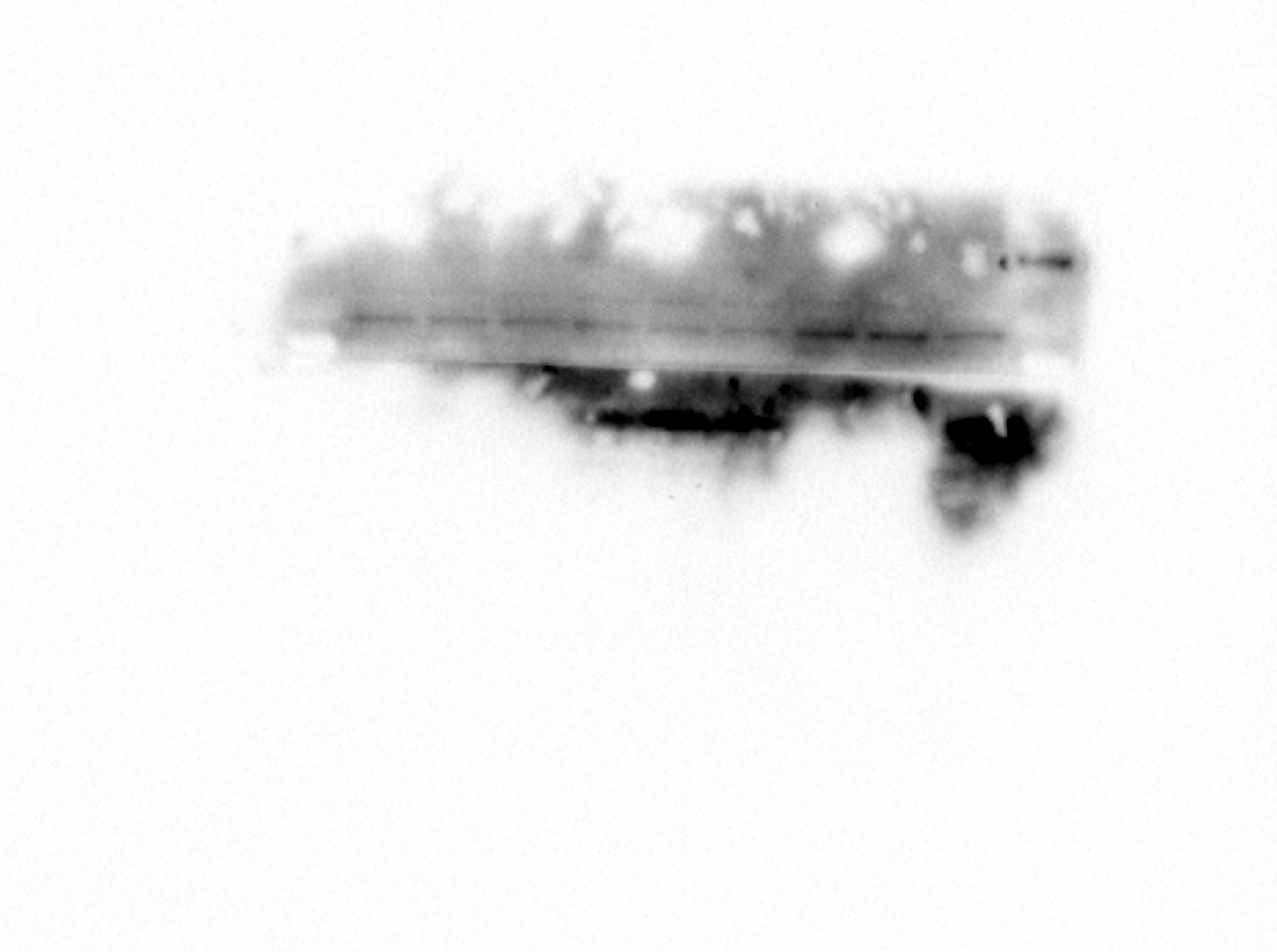

Supplement: Supplemental Information 22 [file peerj-14-21375-s022.zip › Figure 3J WB RAW OE-KLHL40/KLHL40-2 CCC MMM OEOEOE.tif]

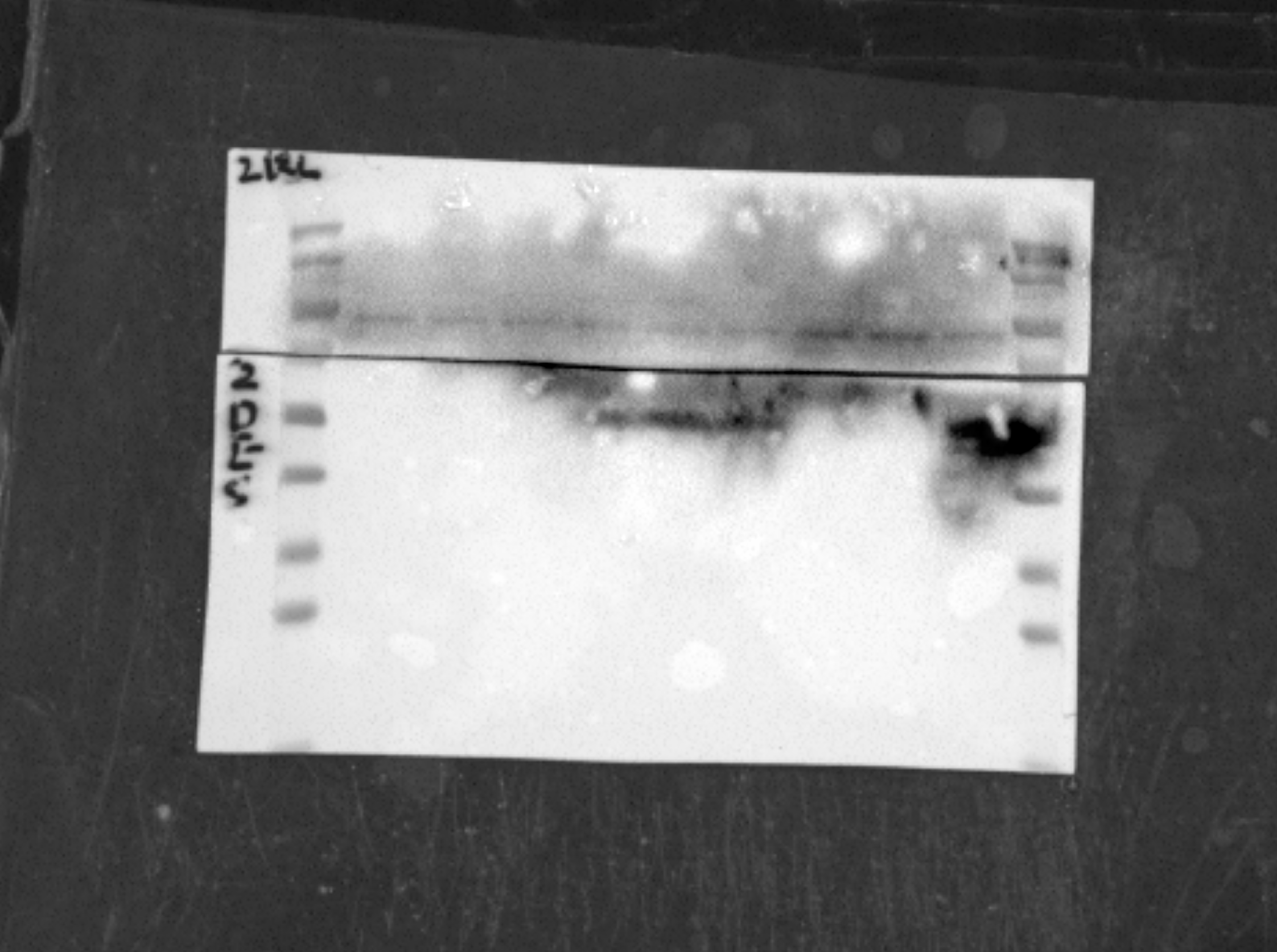

Supplement: Supplemental Information 22 [file peerj-14-21375-s022.zip › Figure 3J WB RAW OE-KLHL40/KLHL40-2+MARK CCC MMM OEOEOE.tif]

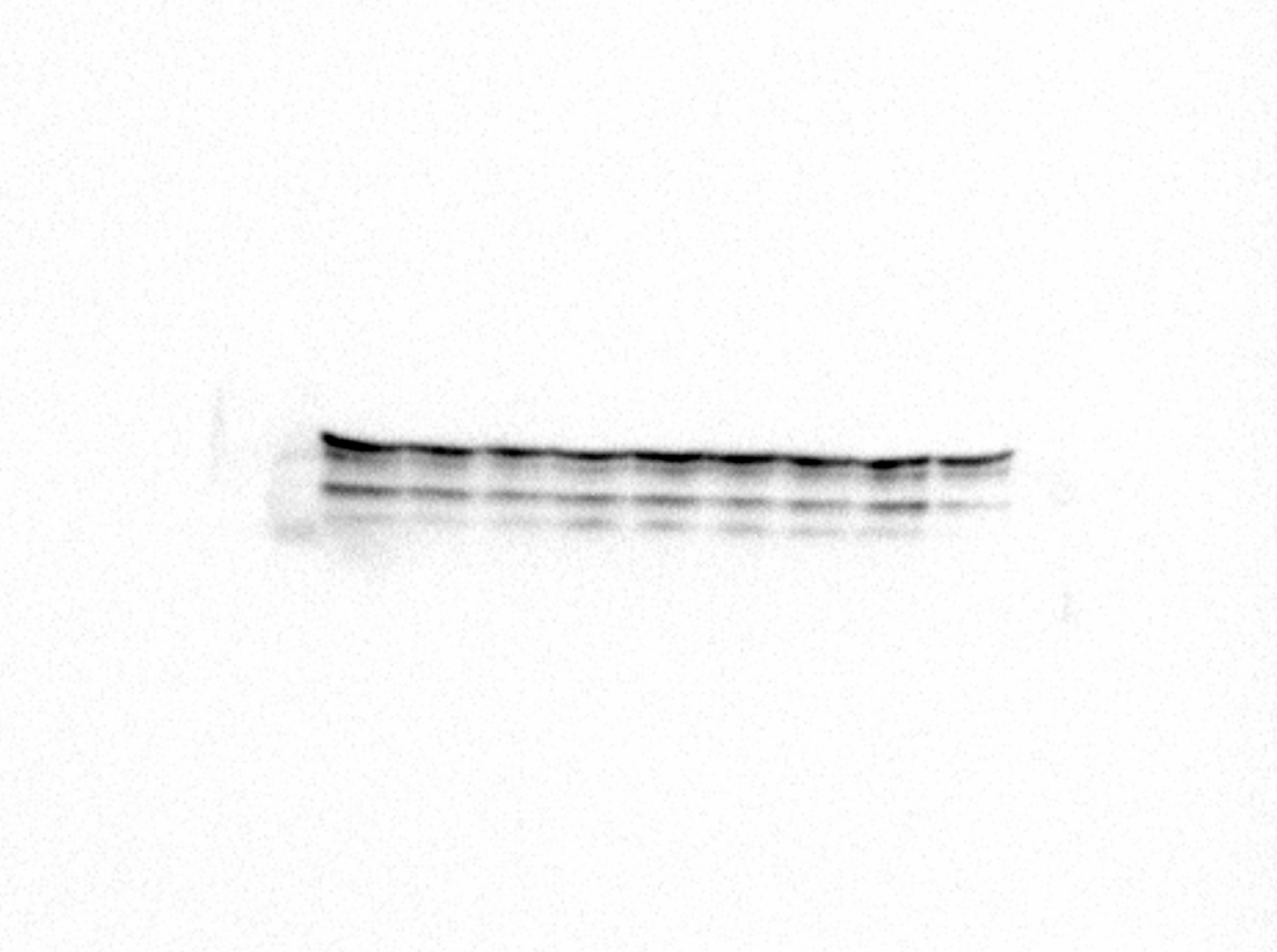

Supplement: Supplemental Information 22 [file peerj-14-21375-s022.zip › Figure 3J WB RAW OE-KLHL40/KLHL40-2-ACTB CCC MMM OEOEOE.tif]

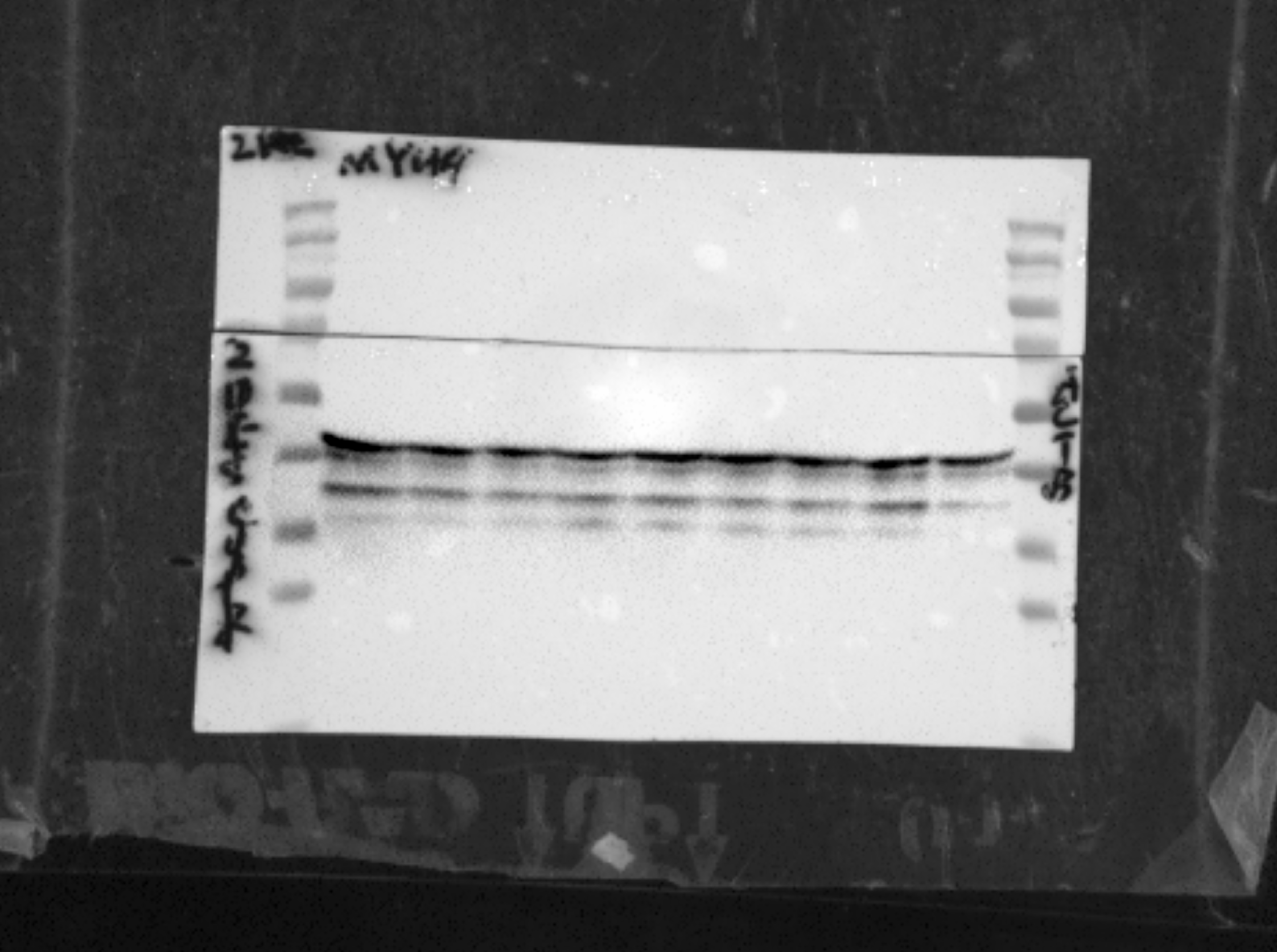

Supplement: Supplemental Information 22 [file peerj-14-21375-s022.zip › Figure 3J WB RAW OE-KLHL40/KLHL40-2-ACTB+MARK CCC MMM OEOEOE.tif]

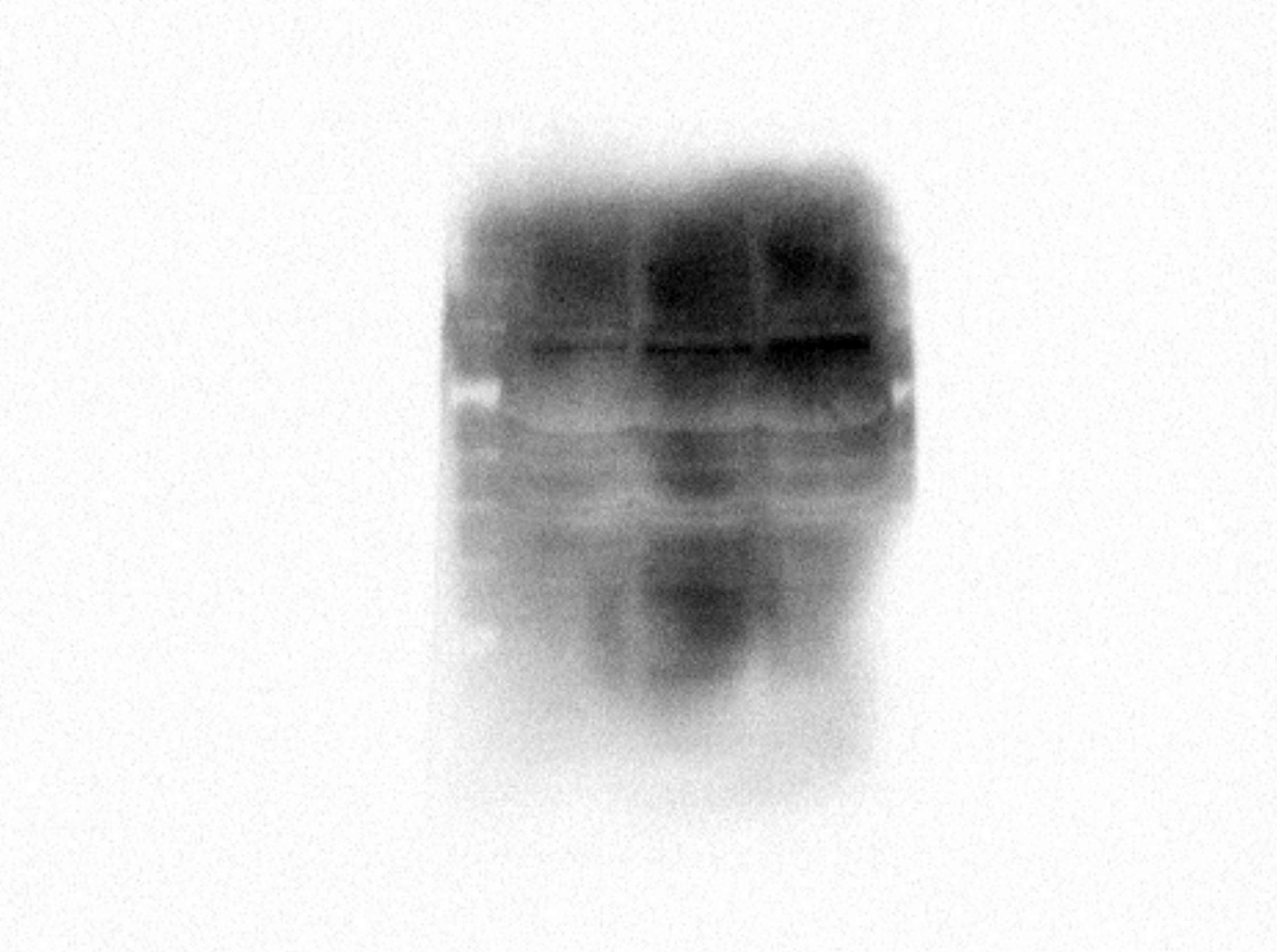

Supplement: Supplemental Information 22 [file peerj-14-21375-s022.zip › Figure 3J WB RAW OE-KLHL40/KLHL40-3.tif]

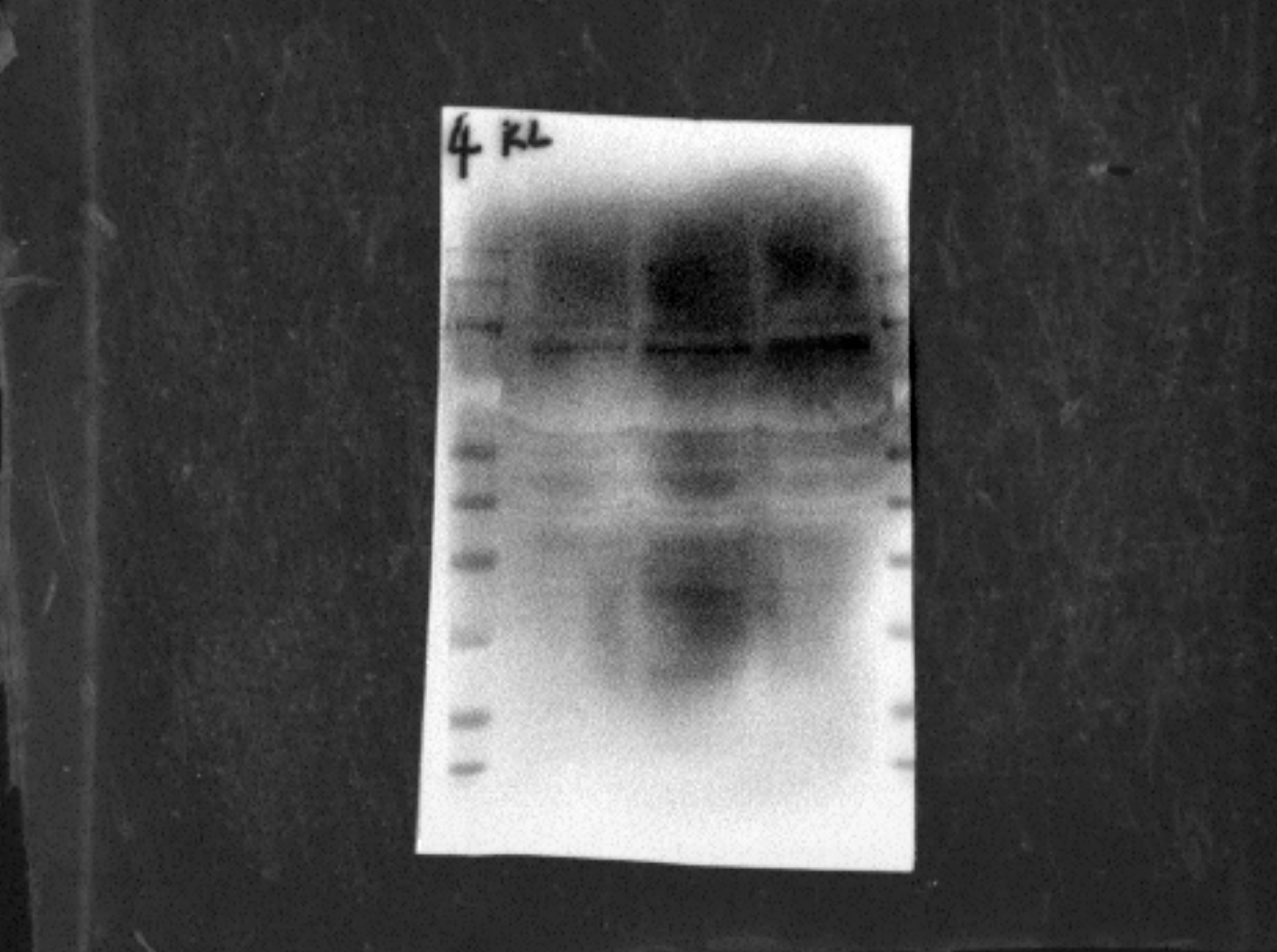

Supplement: Supplemental Information 22 [file peerj-14-21375-s022.zip › Figure 3J WB RAW OE-KLHL40/KLHL40-3+MARK.tif]

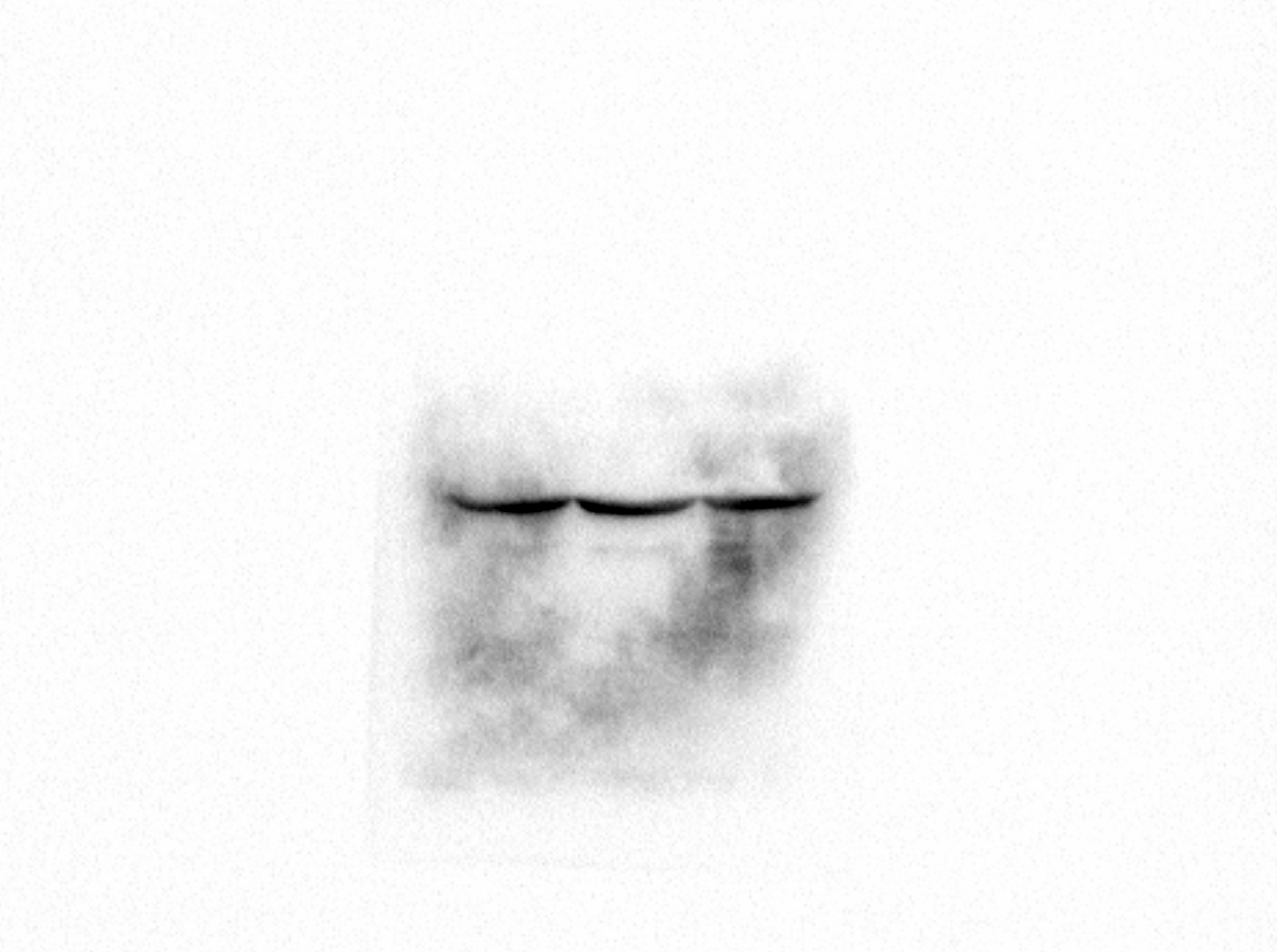

Supplement: Supplemental Information 22 [file peerj-14-21375-s022.zip › Figure 3J WB RAW OE-KLHL40/KLHL40-3-ACTB.tif]

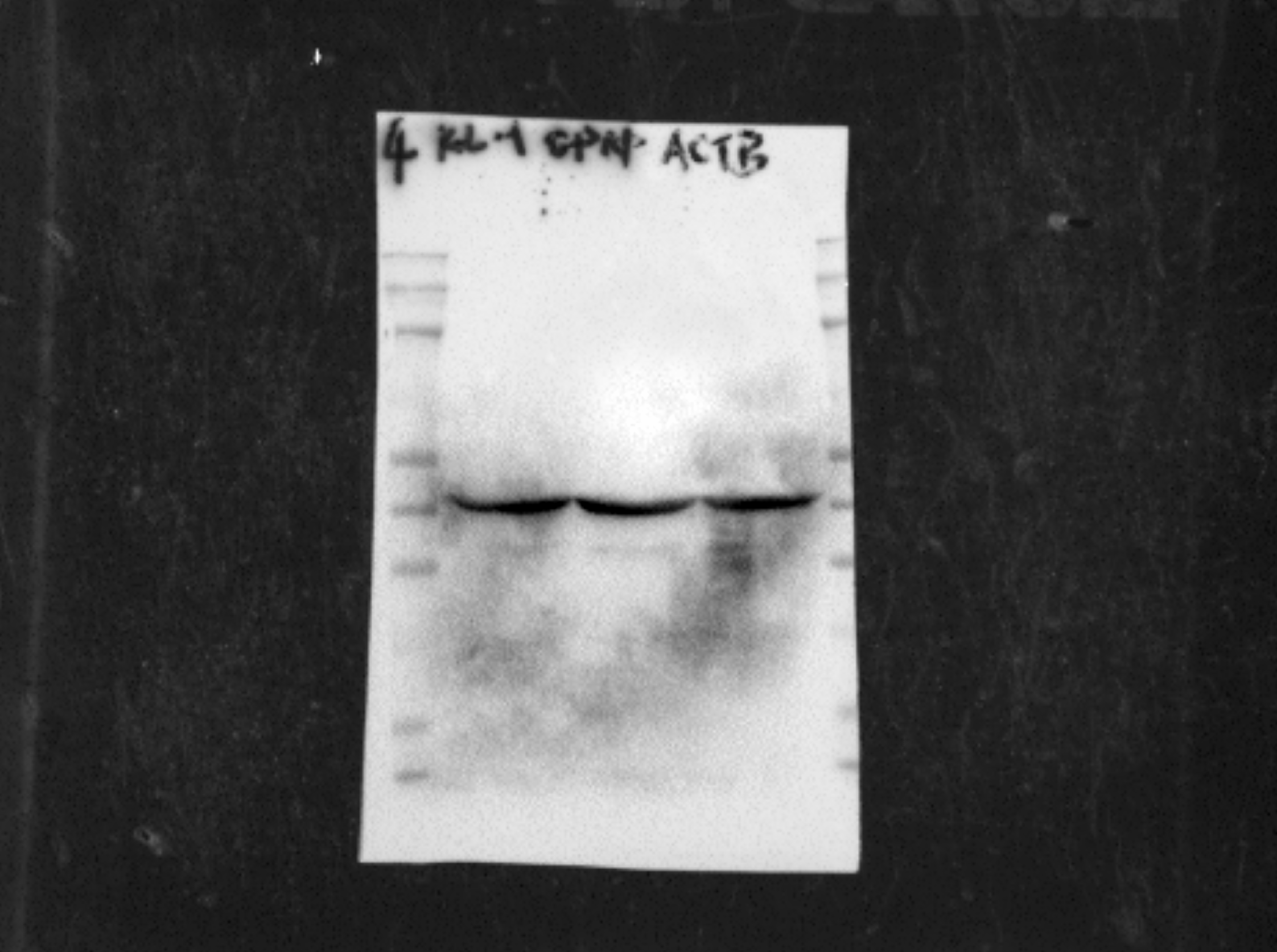

Supplement: Supplemental Information 22 [file peerj-14-21375-s022.zip › Figure 3J WB RAW OE-KLHL40/KLHL40-3-ACTB+MARK.tif]

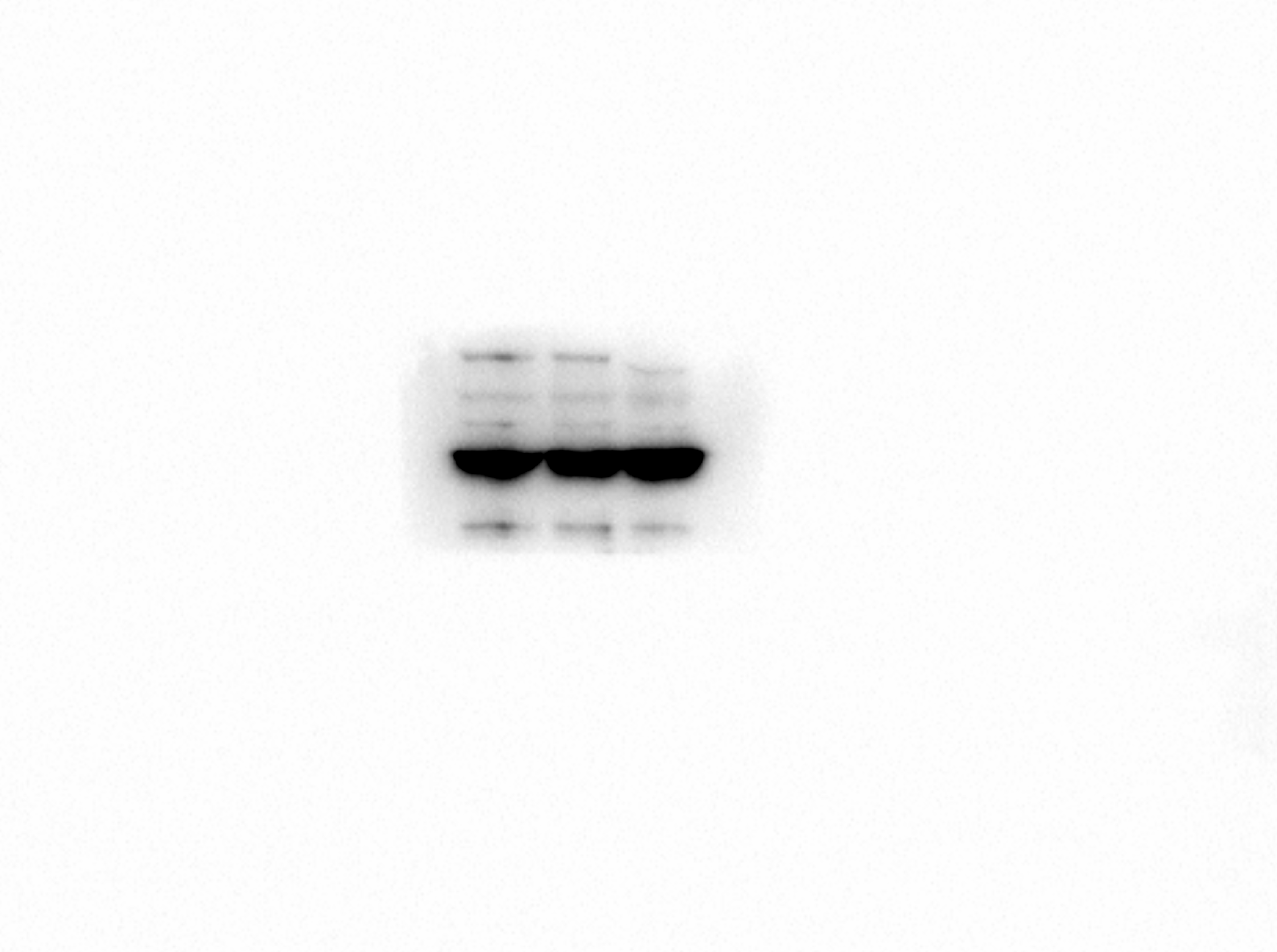

Supplement: Supplemental Information 24 [file peerj-14-21375-s024.zip › Figure 4A WB RAW SH-KLHL40 MYOT/1ACTIN.tif]

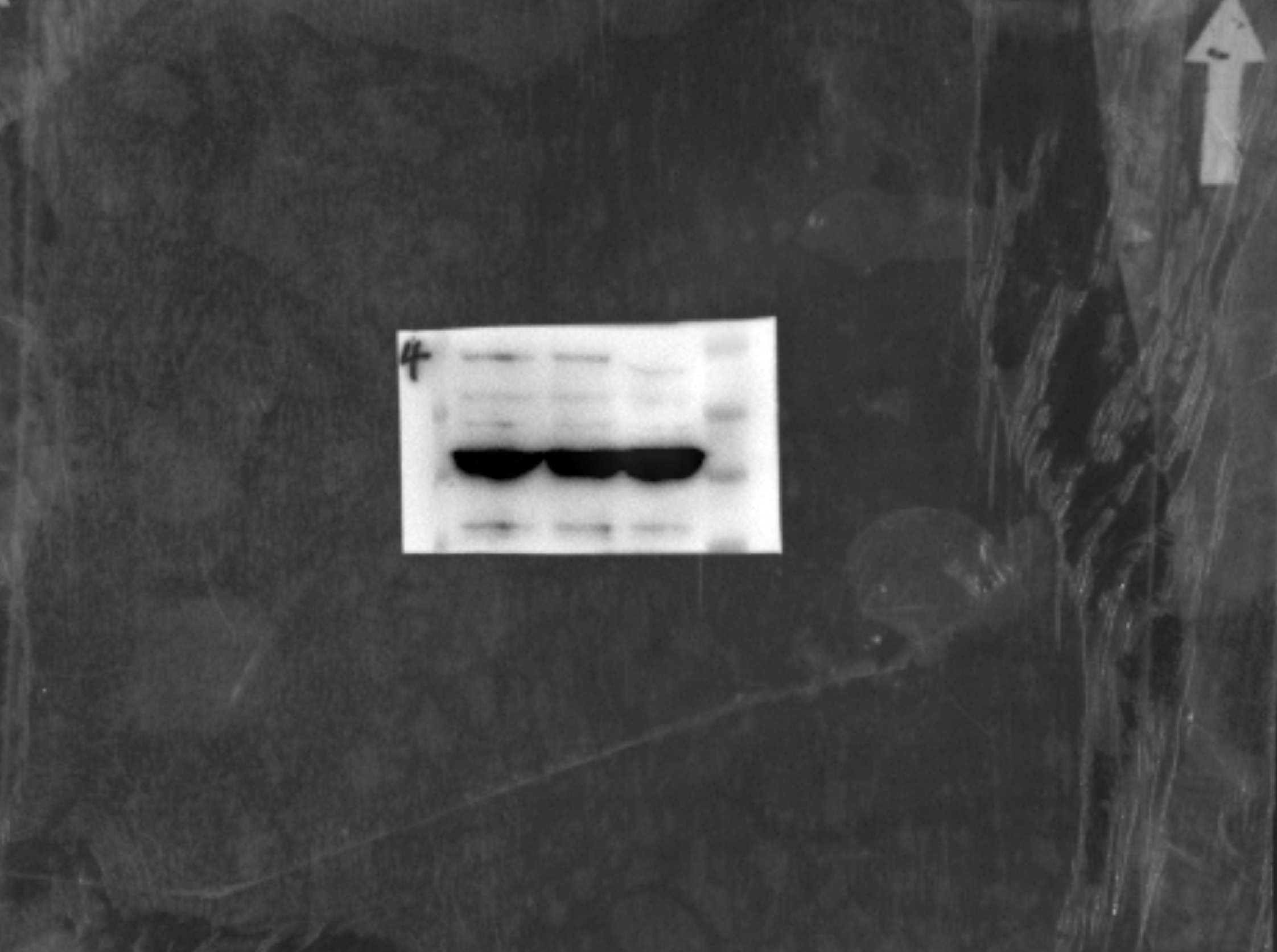

Supplement: Supplemental Information 24 [file peerj-14-21375-s024.zip › Figure 4A WB RAW SH-KLHL40 MYOT/1ACTIN+MARKER.tif]

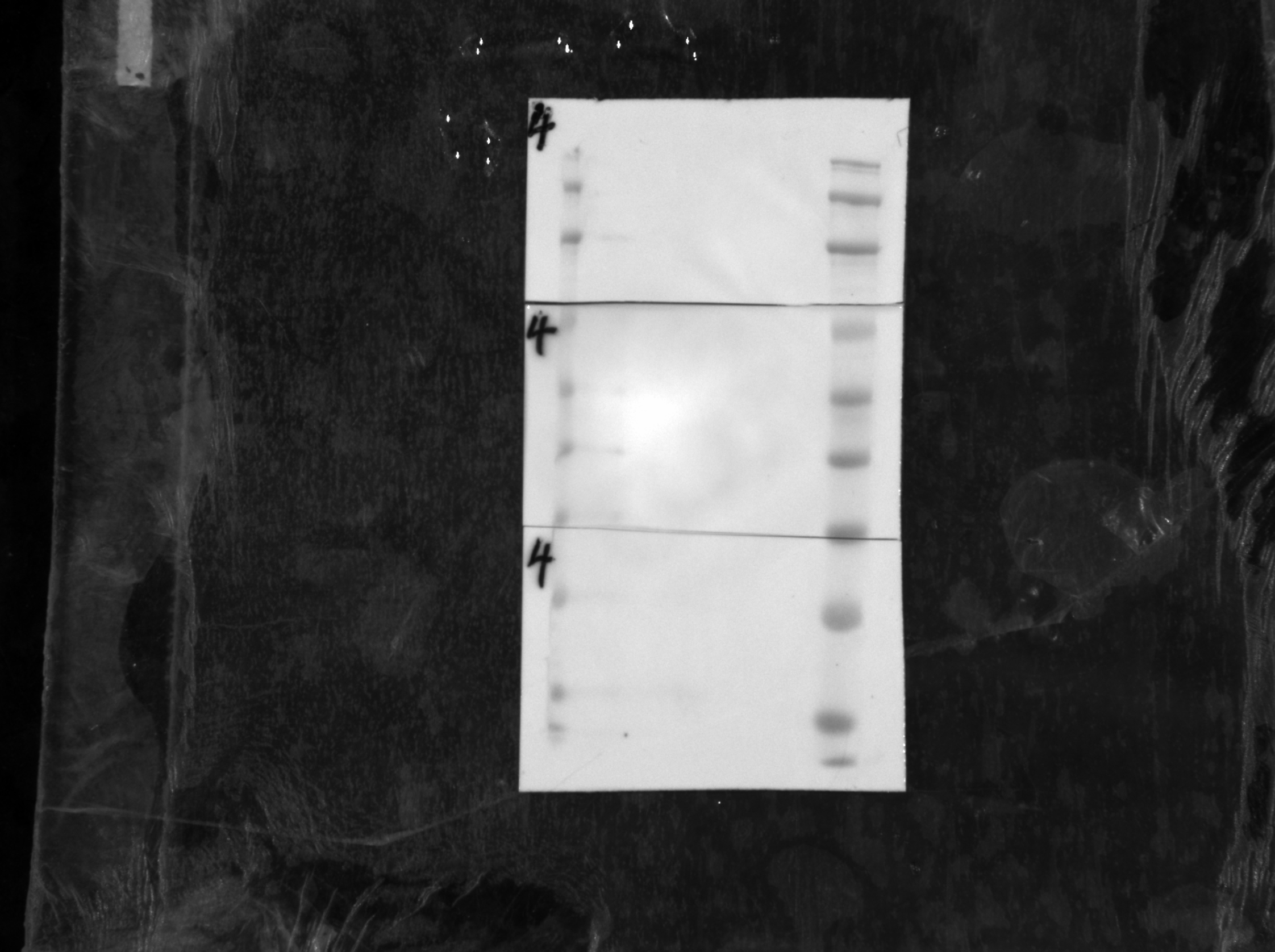

Supplement: Supplemental Information 24 [file peerj-14-21375-s024.zip › Figure 4A WB RAW SH-KLHL40 MYOT/1ALL.tif]

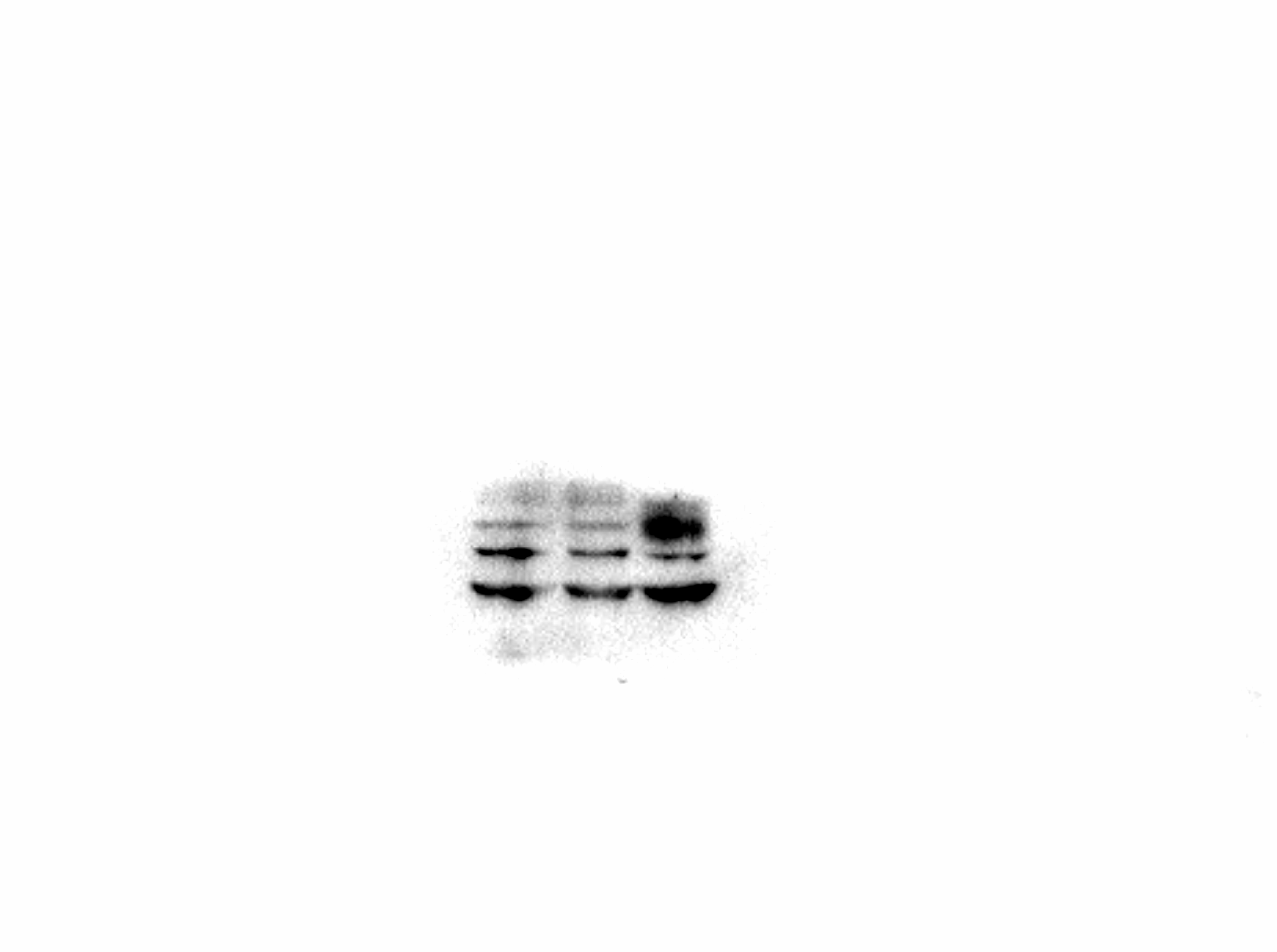

Supplement: Supplemental Information 24 [file peerj-14-21375-s024.zip › Figure 4A WB RAW SH-KLHL40 MYOT/1MYOT.tif]

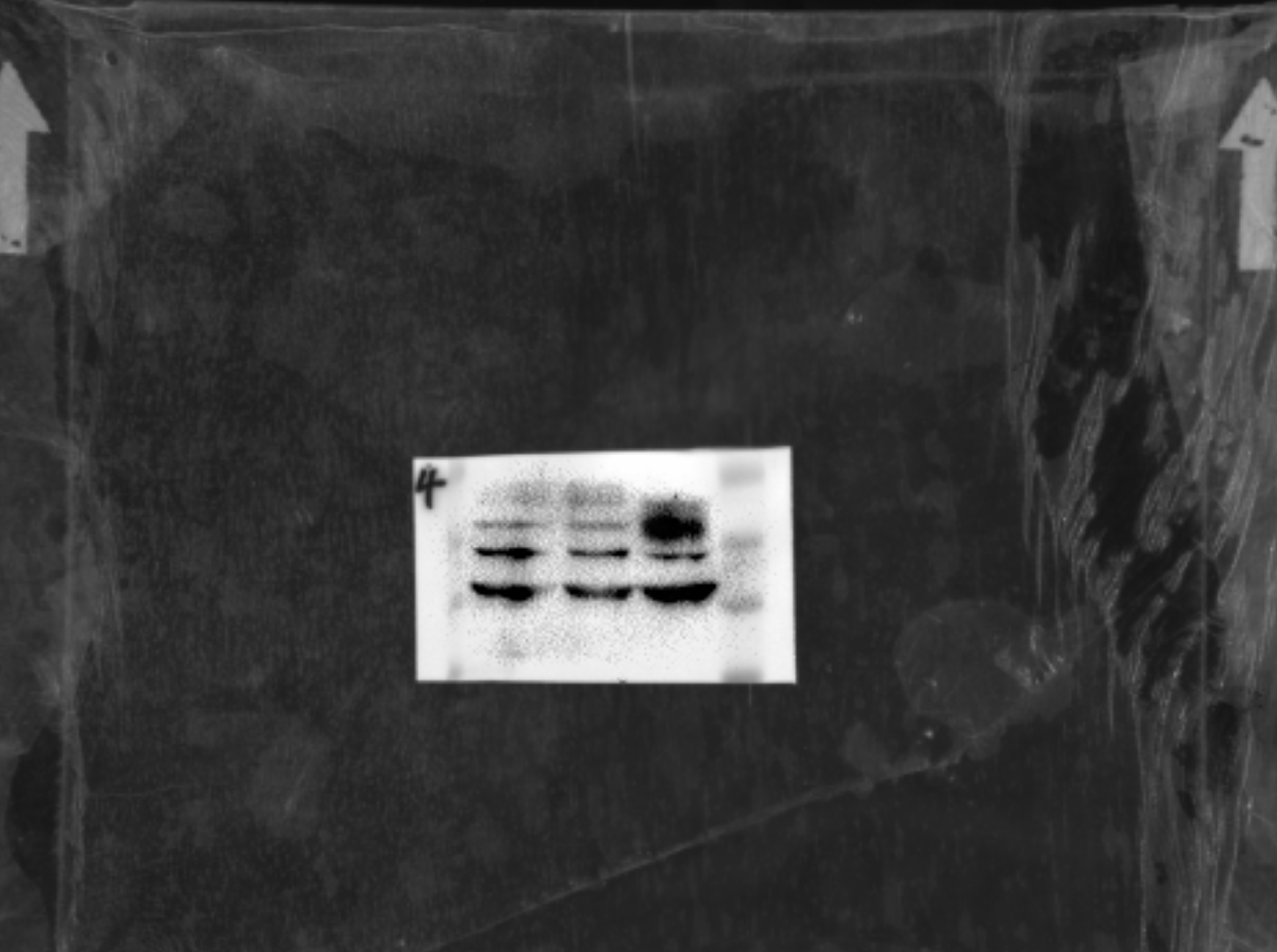

Supplement: Supplemental Information 24 [file peerj-14-21375-s024.zip › Figure 4A WB RAW SH-KLHL40 MYOT/1MYOT+MARKER.tif]

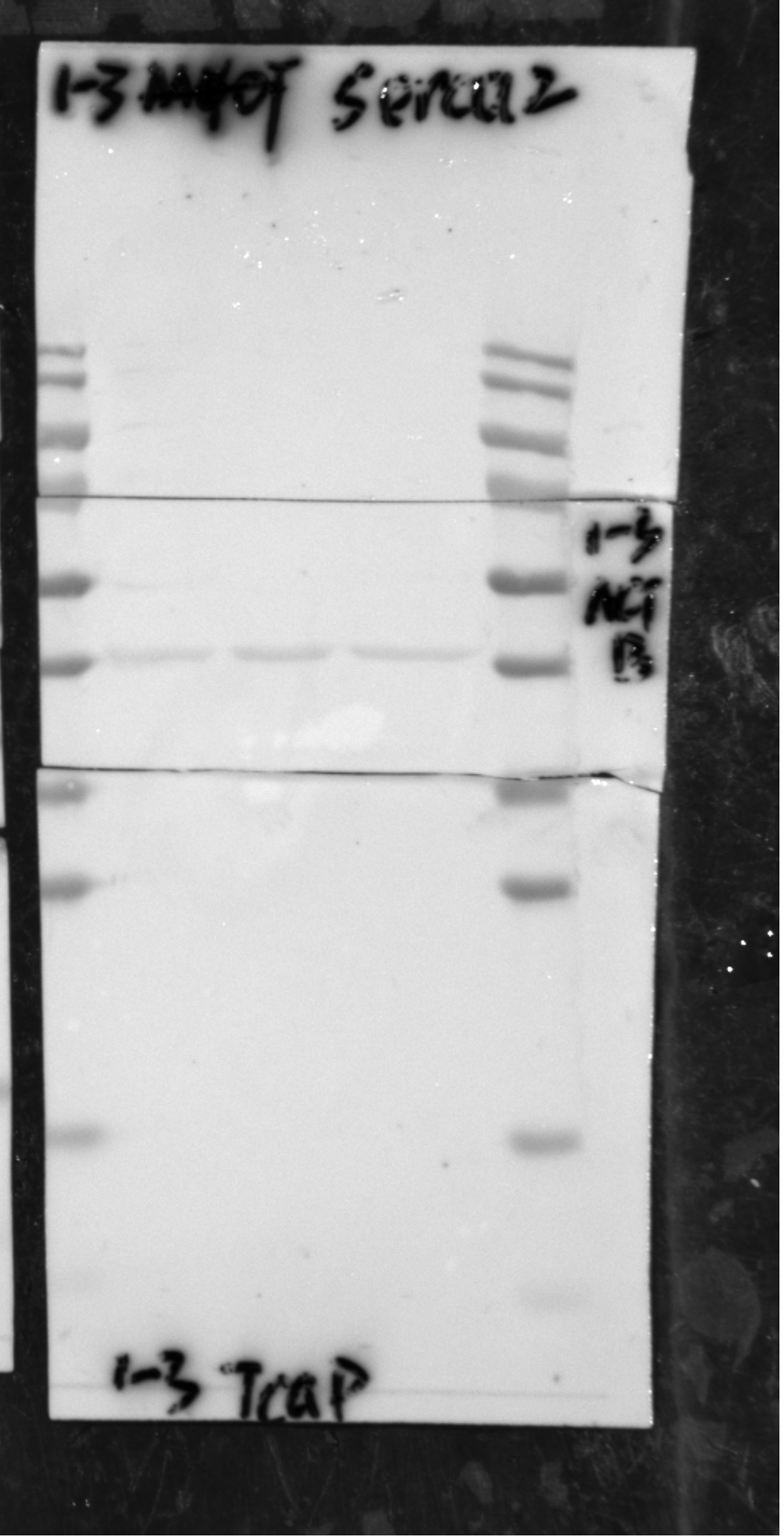

Supplement: Supplemental Information 24 [file peerj-14-21375-s024.zip › Figure 4A WB RAW SH-KLHL40 MYOT/3ALL.jpg]

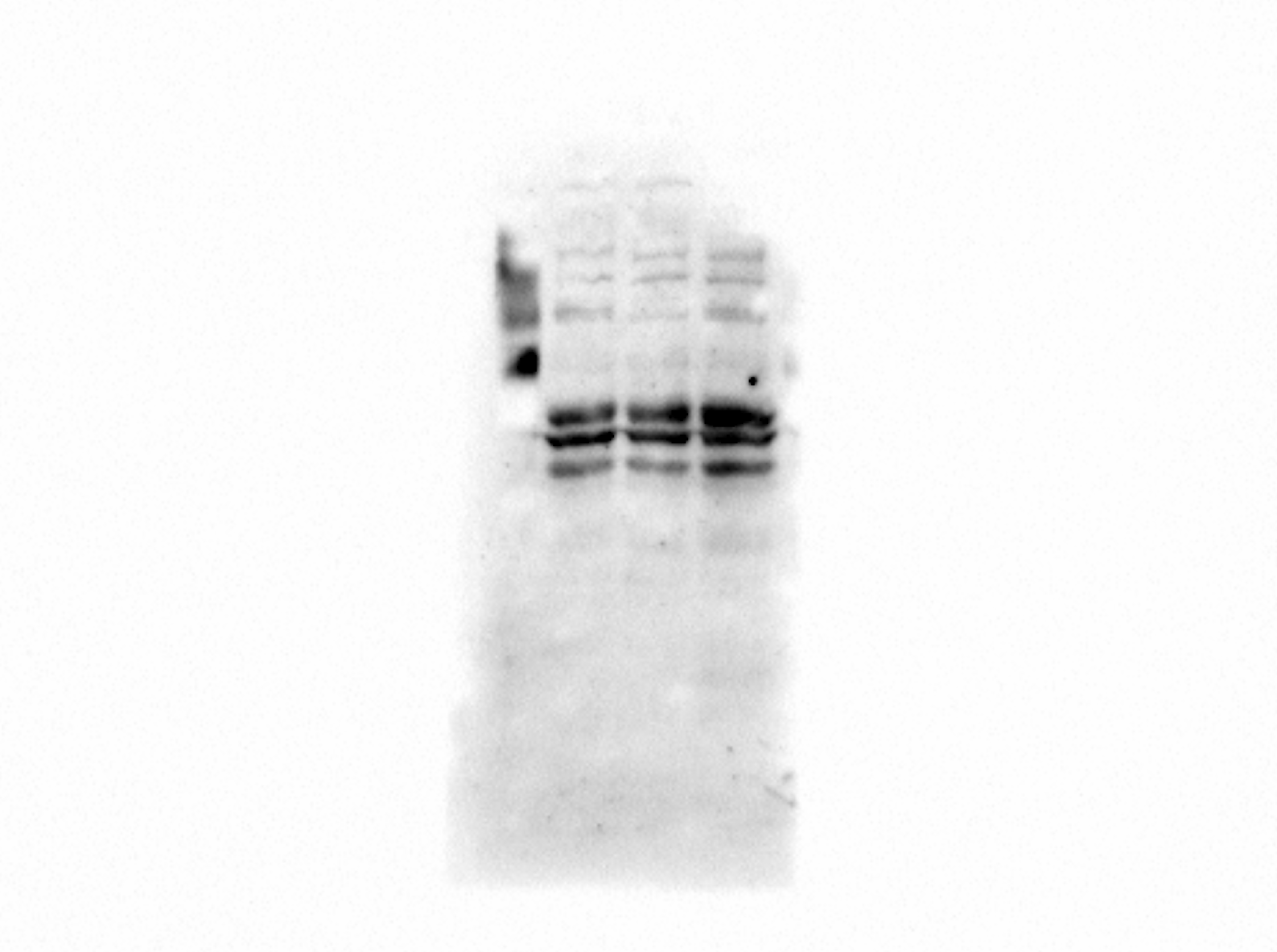

Supplement: Supplemental Information 24 [file peerj-14-21375-s024.zip › Figure 4A WB RAW SH-KLHL40 MYOT/MYOT-2 sh-KLHL40.tif]

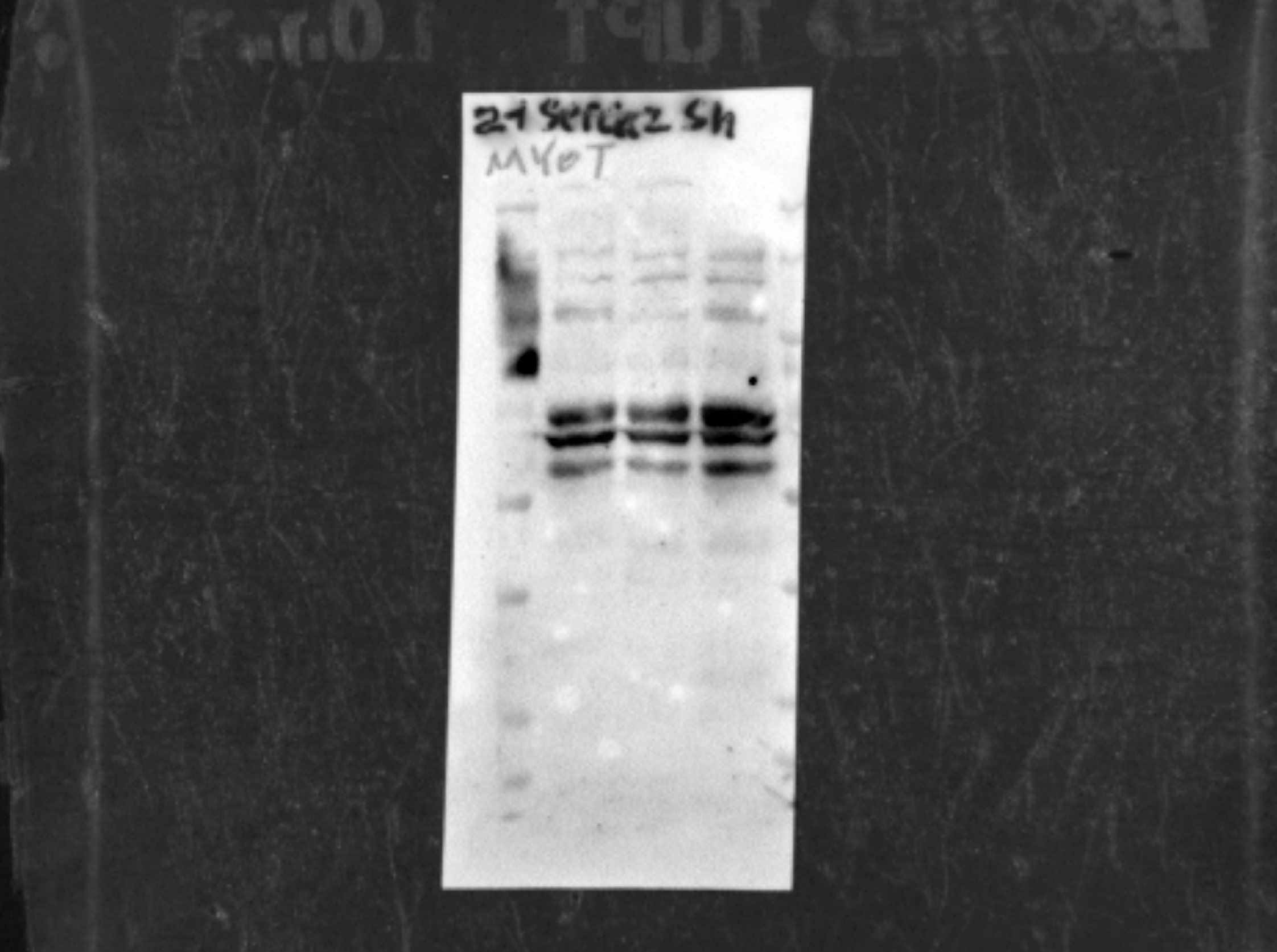

Supplement: Supplemental Information 24 [file peerj-14-21375-s024.zip › Figure 4A WB RAW SH-KLHL40 MYOT/MYOT-2 sh-KLHL40+MARK.tif]

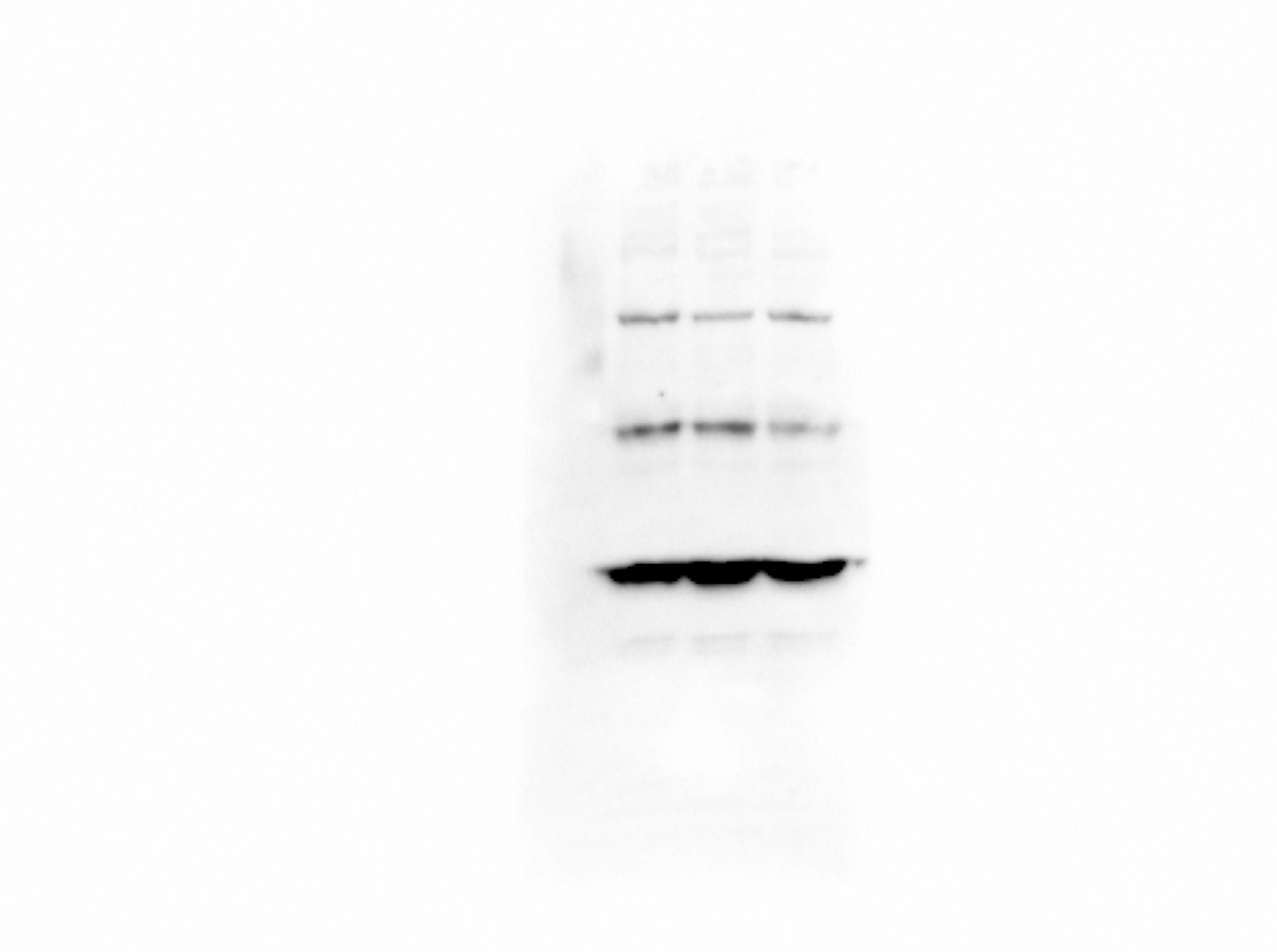

Supplement: Supplemental Information 24 [file peerj-14-21375-s024.zip › Figure 4A WB RAW SH-KLHL40 MYOT/MYOT-2 sh-KLHL40-ACTB.tif]

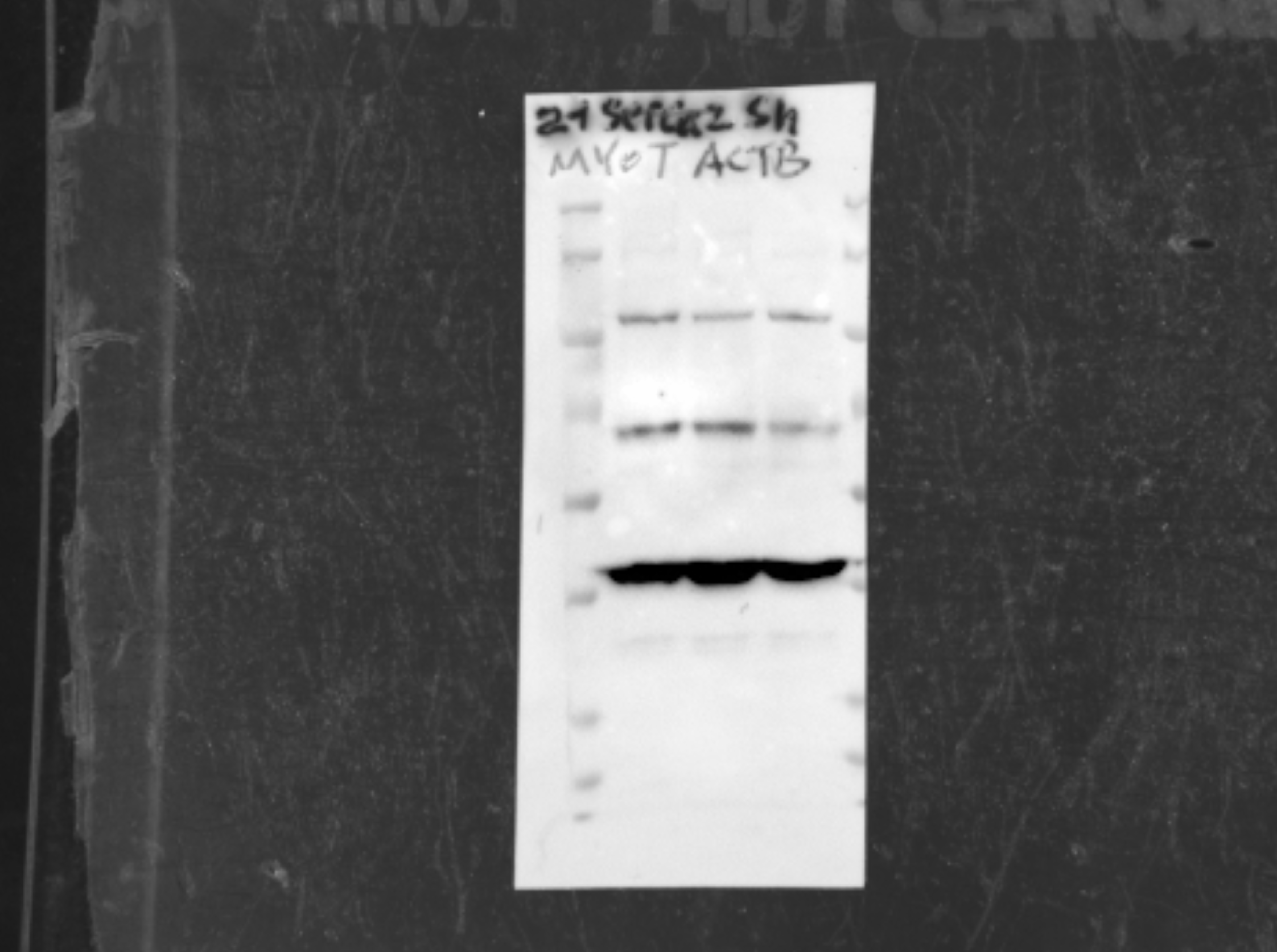

Supplement: Supplemental Information 24 [file peerj-14-21375-s024.zip › Figure 4A WB RAW SH-KLHL40 MYOT/MYOT-2 sh-KLHL40-ACTB+MARK.tif]

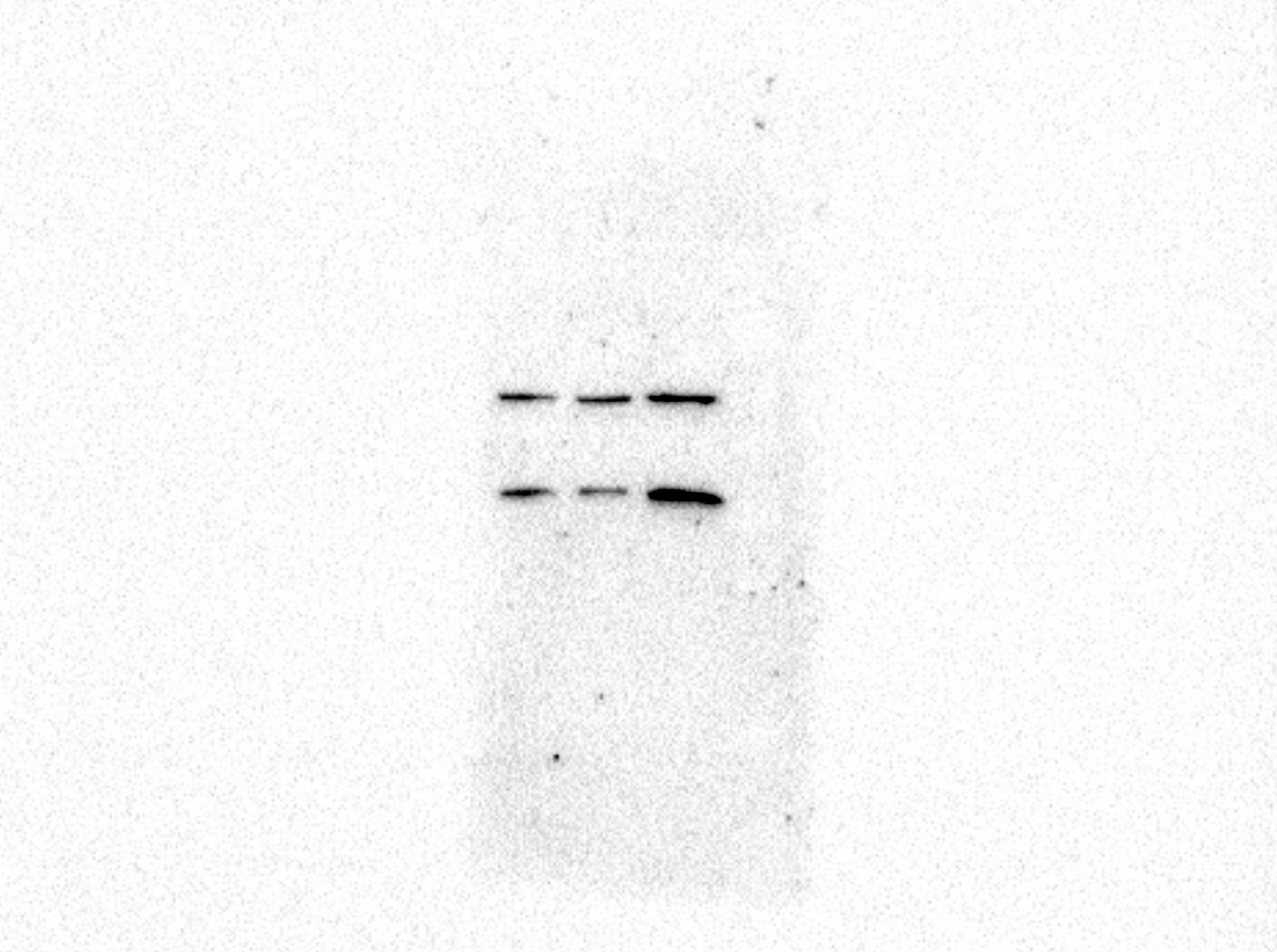

Supplement: Supplemental Information 24 [file peerj-14-21375-s024.zip › Figure 4A WB RAW SH-KLHL40 MYOT/MYOT-3 sh-KLHL40.tif]

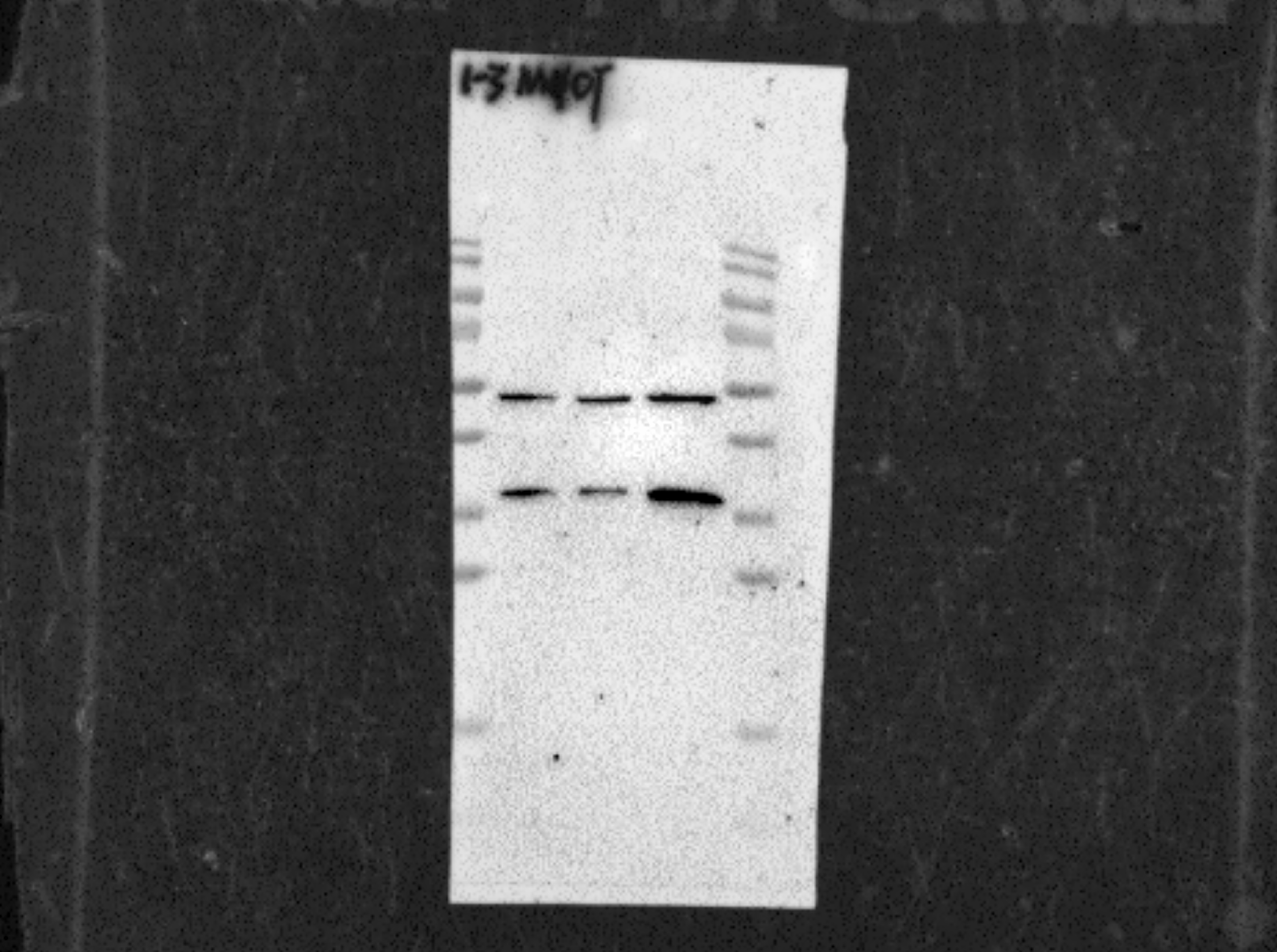

Supplement: Supplemental Information 24 [file peerj-14-21375-s024.zip › Figure 4A WB RAW SH-KLHL40 MYOT/MYOT-3 sh-KLHL40+MARK.tif]

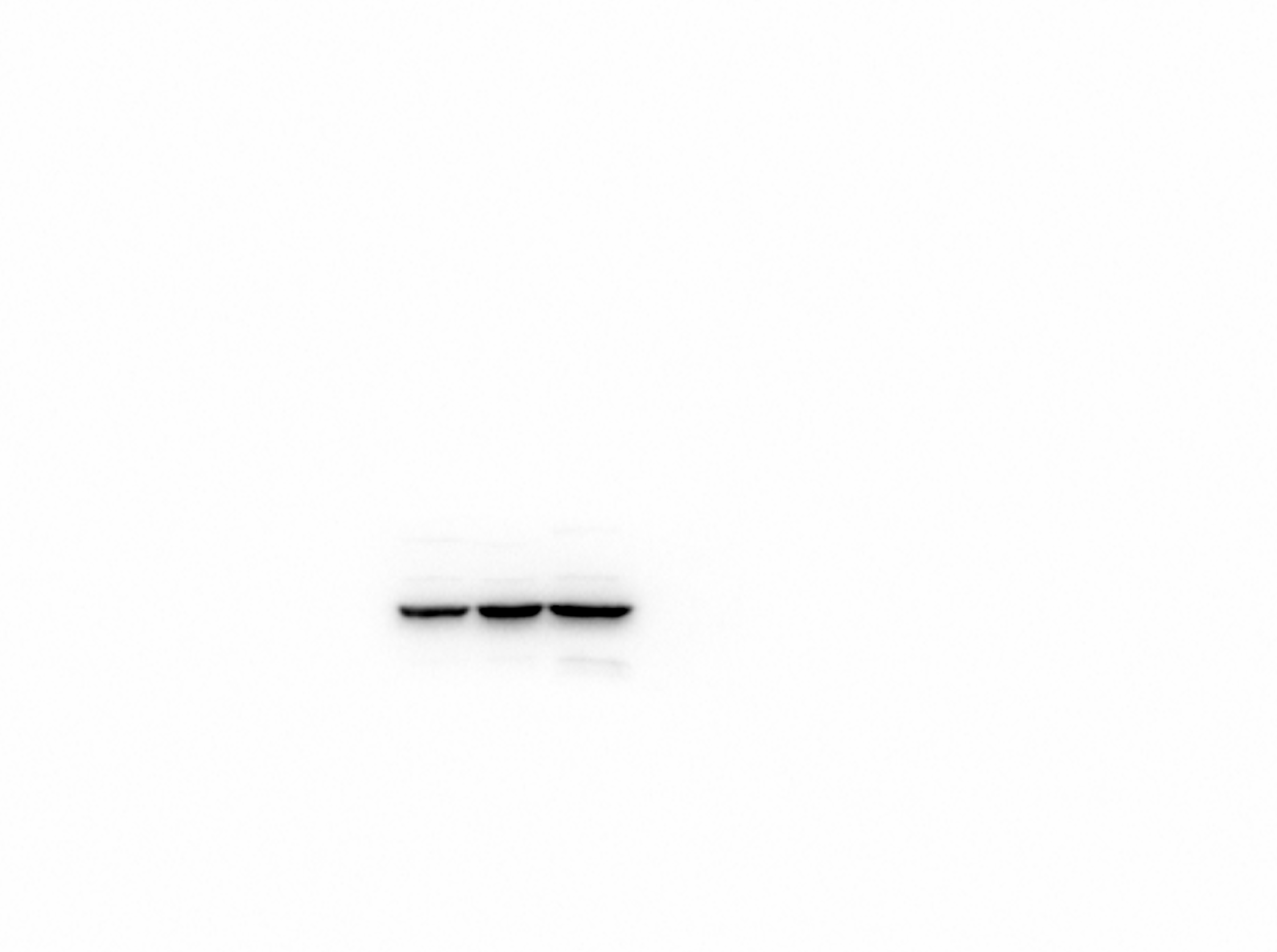

Supplement: Supplemental Information 24 [file peerj-14-21375-s024.zip › Figure 4A WB RAW SH-KLHL40 MYOT/MYOT-3 sh-KLHL40-ACTB.tif]

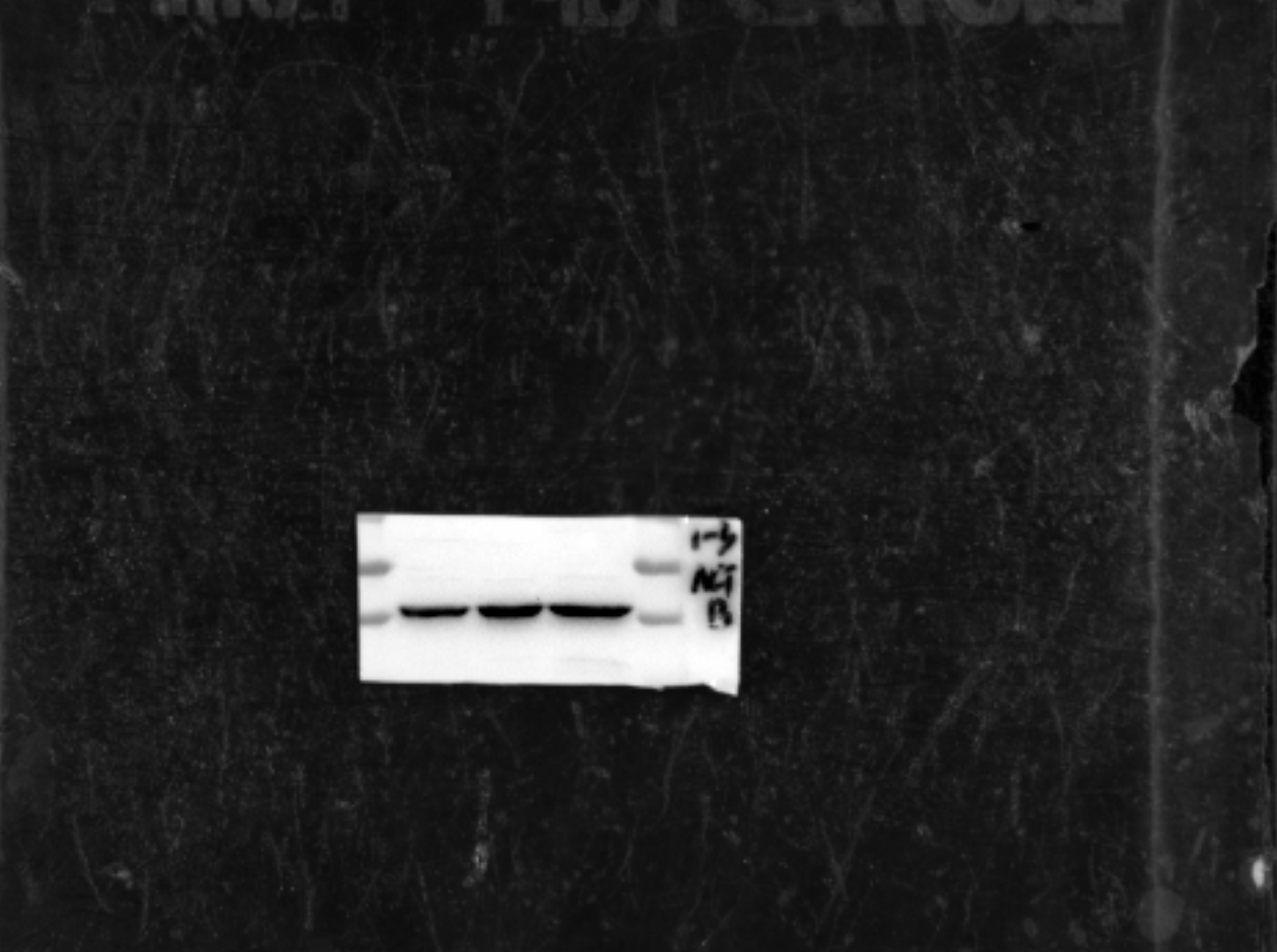

Supplement: Supplemental Information 24 [file peerj-14-21375-s024.zip › Figure 4A WB RAW SH-KLHL40 MYOT/MYOT-3 sh-KLHL40-ACTB+MARK.tif]

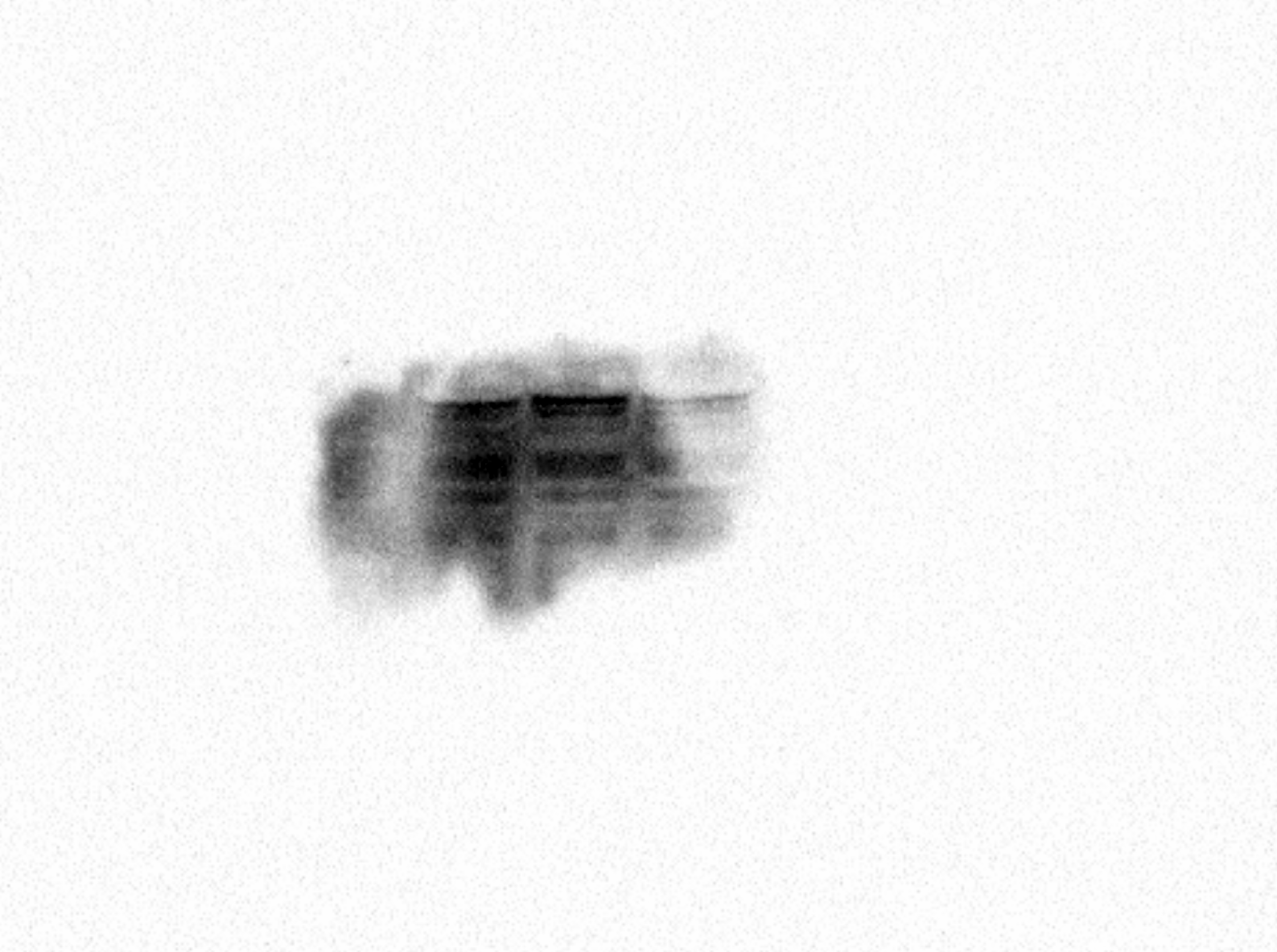

Supplement: Supplemental Information 25 [file peerj-14-21375-s025.zip › Figure 4B WB RAW oe-KLHL40 MYOT/MYOT-1 oe-KLHL40.tif]
