# Supplementary material for: Post-infarction KLHL40-mediated regulation of cardiac sarcomeric integrity and function
Source: PeerJ. 2026 Jun 5;14:e21375. doi: 10.7717/peerj.21375 (PMC13245431; doi:10.7717/peerj.21375)
Supplement: Supplemental Information 11 [file peerj-14-21375-s011.zip › IHC+IF+Morphology.docx]

**Table of Contents**

[Figure. 2 IHC human heart tissue 2](#_Toc7073)

[Figure. 5 Fluo-4 9](#_Toc9427)

[Figure. 7, H and M Morphology 12](#_Toc27155)

[Heart view from all perspectives 13](#_Toc24448)

[Dalian Medical University Biomedical Ethics Committee Review Decision 15](#_Toc14496)

[Informed Consent 16](#_Toc9786)

[Figure S 5 Sanger Sequencing 19](#_Toc17136)

# Figure. 2 IHC human heart tissue

| **Early of MI** | |
| --- | --- |
| **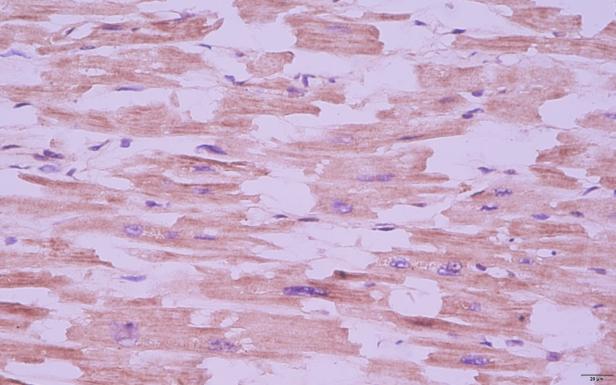** | **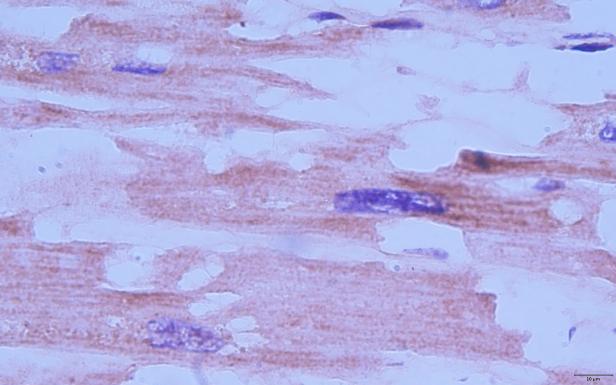** |
| 1-1 Scale 20μm | 1-2 Scale 10μm |
| **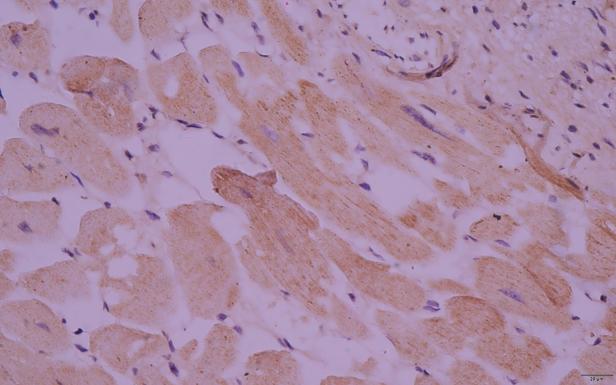** | **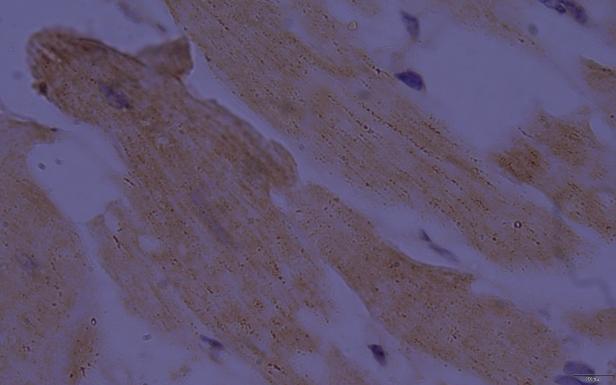** |
| 2-1 Scale 20μm | 2-2 Scale 10μm |
| **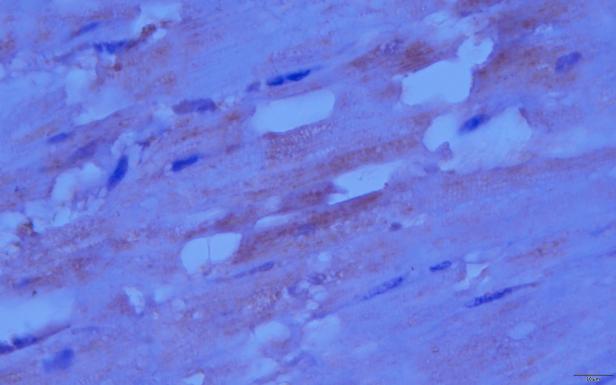** | **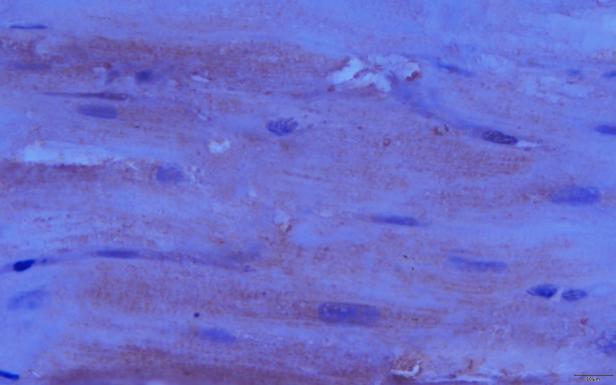** |
| 3-1 Scale 10μm | 3-2 Scale 10μm |
| **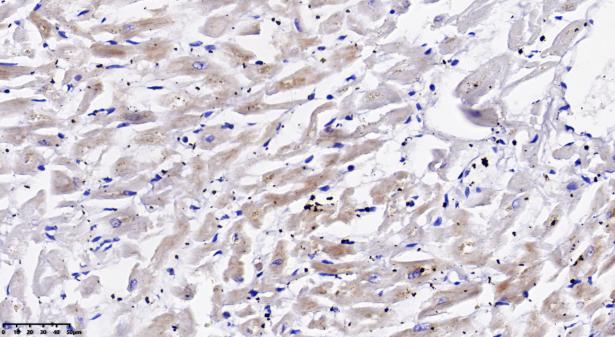** | **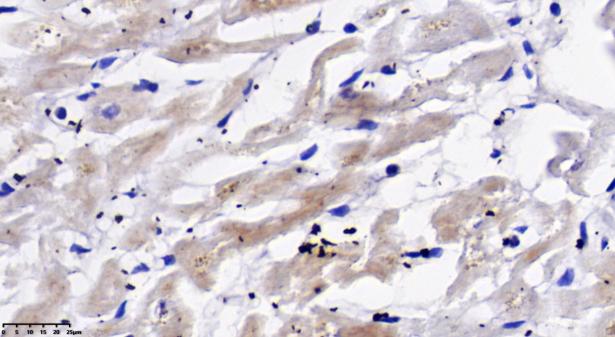** |
| 4-1 Scale 50μm | 4-2 Scale 25μm |
| **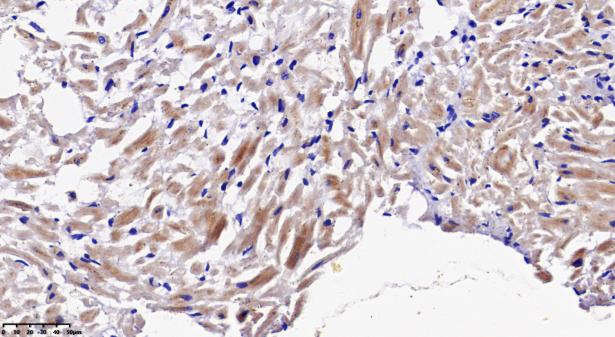** | **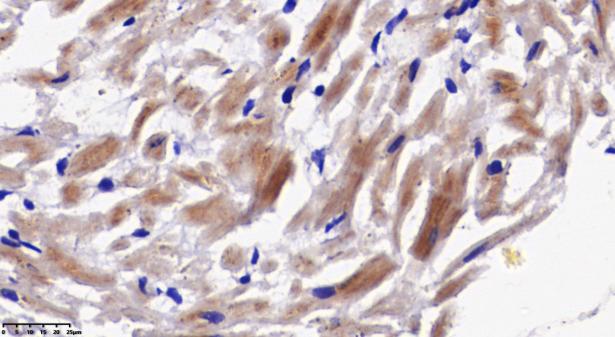** |
| 5-1 Scale 50μm | 5-2 Scale 25μm |
| **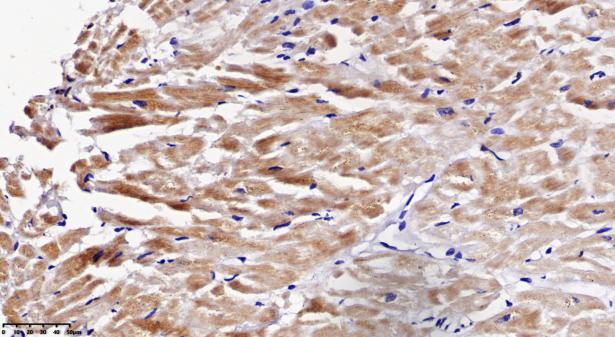** | **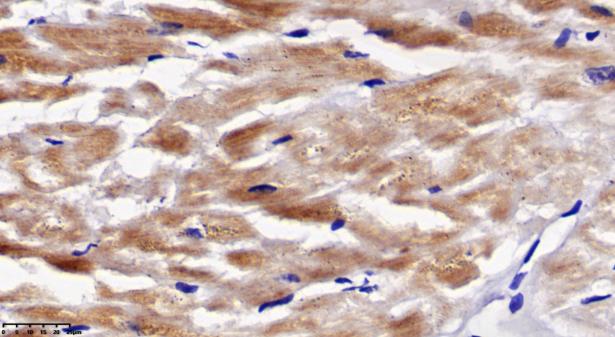** |
| 6-1 Scale 50μm | 6-2 Scale 25μm |

| **Late of MI** | |
| --- | --- |
| **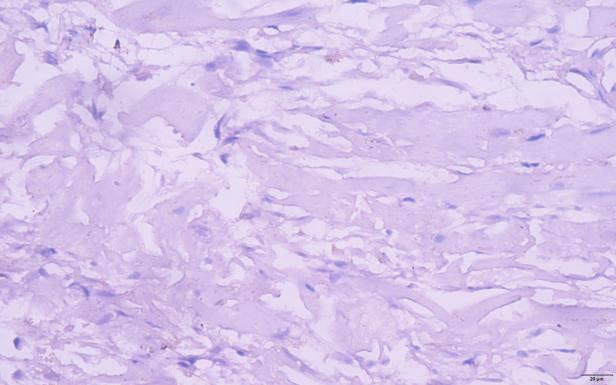** | **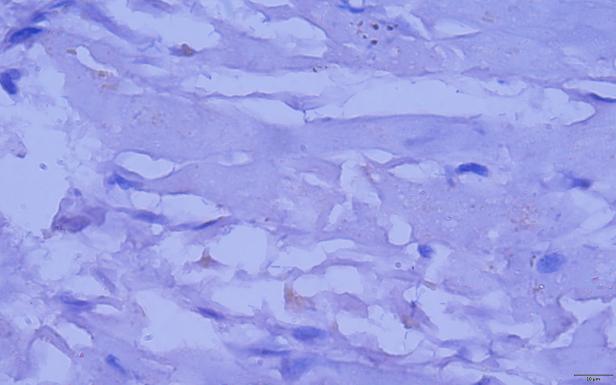** |
| 1-1 Scale 20μm | 1-2 Scale 10μm |
| **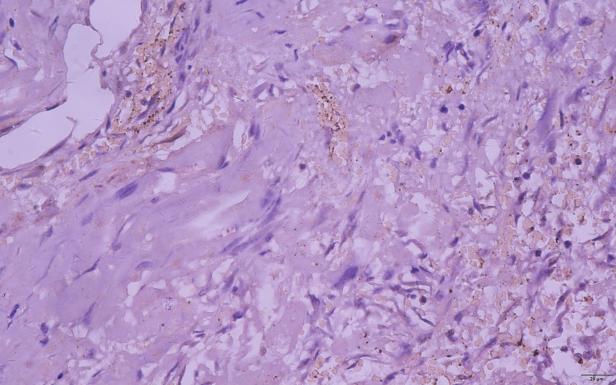** | **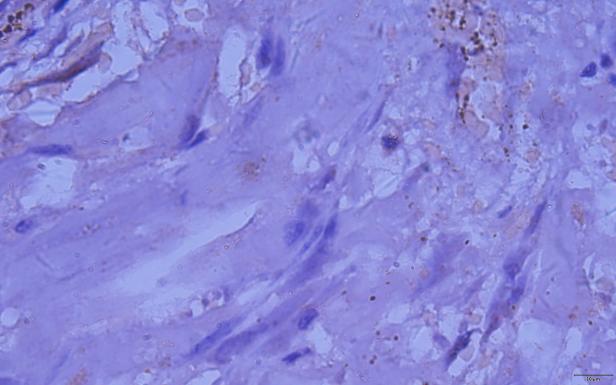** |
| 2-1 Scale 20μm | 2-2 Scale 10μm |
| **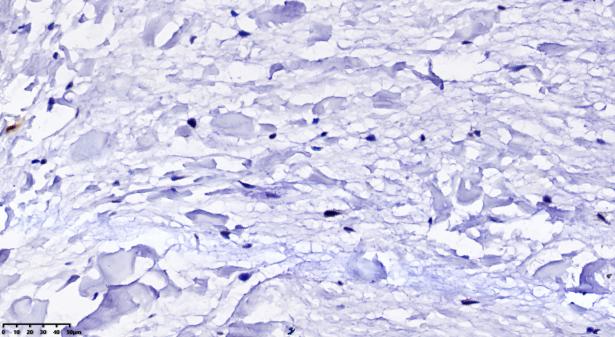** | **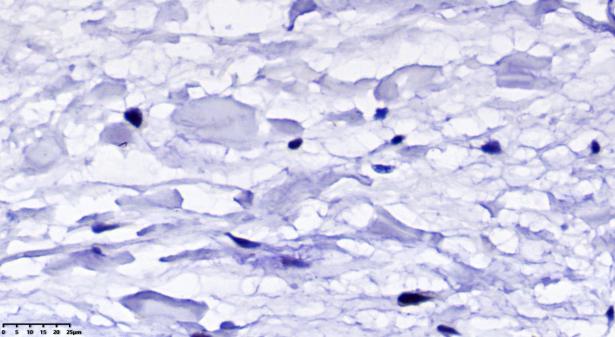** |
| 3-1 Scale 50μm | 3-2 Scale 25μm |
| **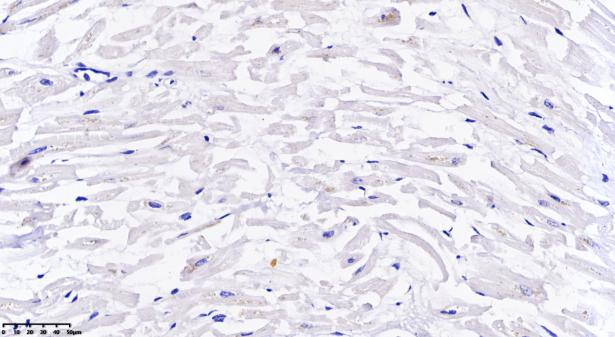** | **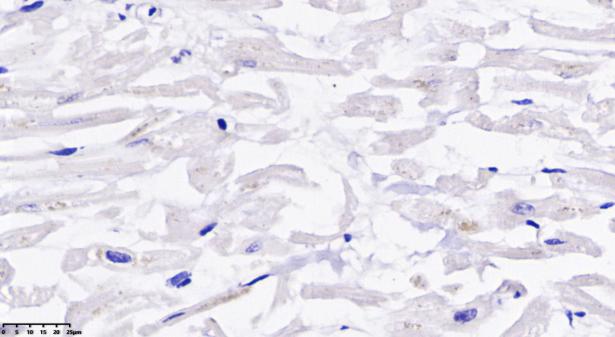** |
| 4-1 Scale 50μm | 4-2 Scale 25μm |
| **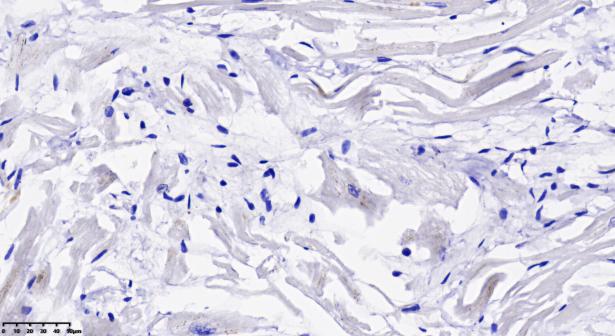** | **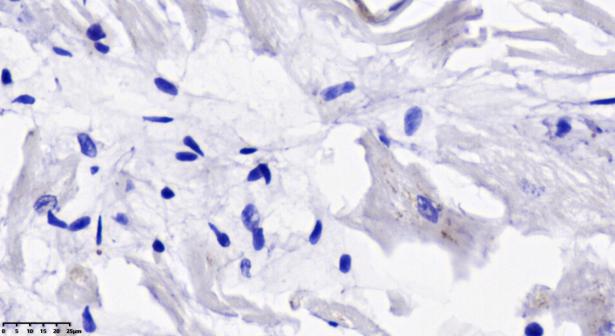** |
| 5-1 Scale 50μm | 5-2 Scale 25μm |
| **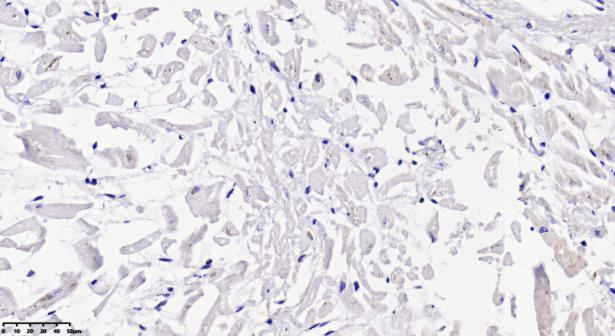** | **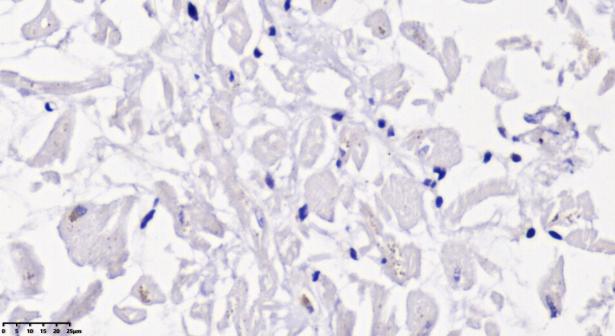** |
| 6-1 Scale 50μm | 6-2 Scale 25μm |

| **Normal** | |
| --- | --- |
| **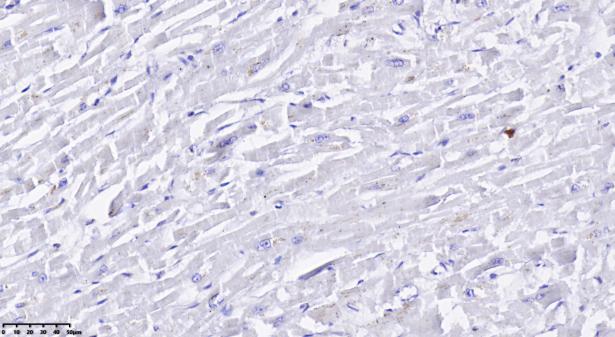** | **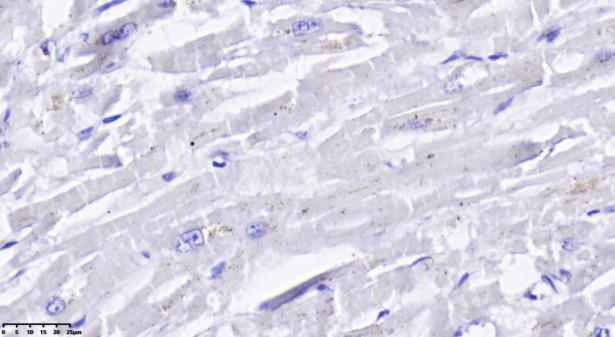** |
| 1-1 Scale 50μm | 1-2 Scale 25μm |
| **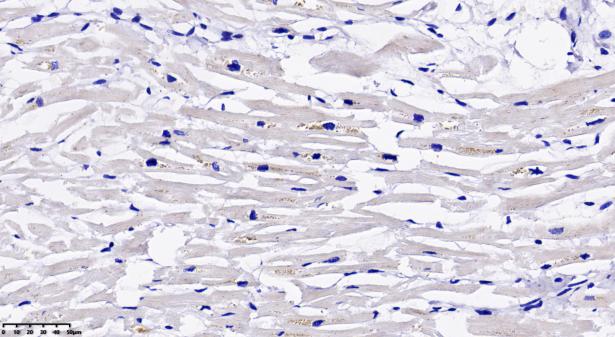** | **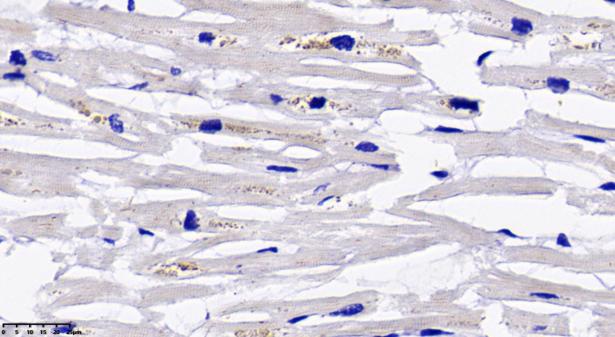** |
| 2-1 Scale 50μm | 2-2 Scale 25μm |
| **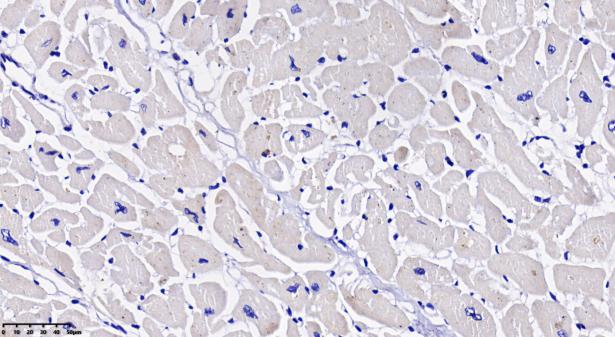** | **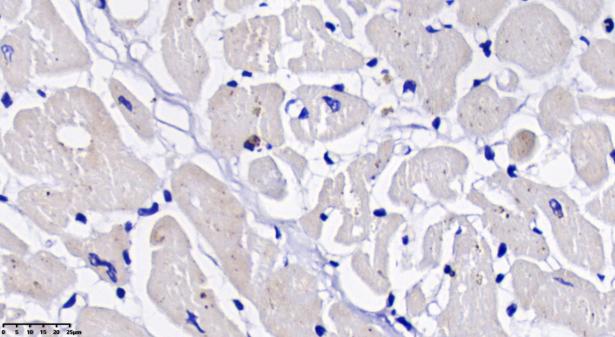** |
| 3-1 Scale 50μm | 3-2 Scale 25μm |
| **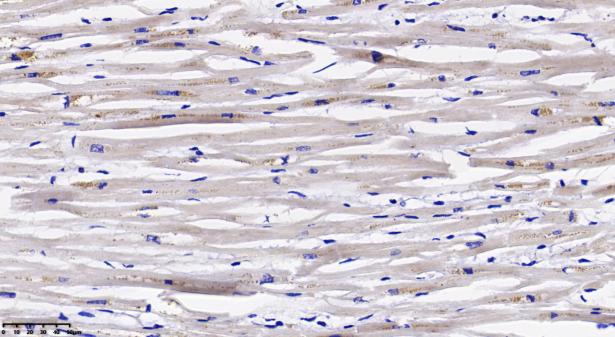** | **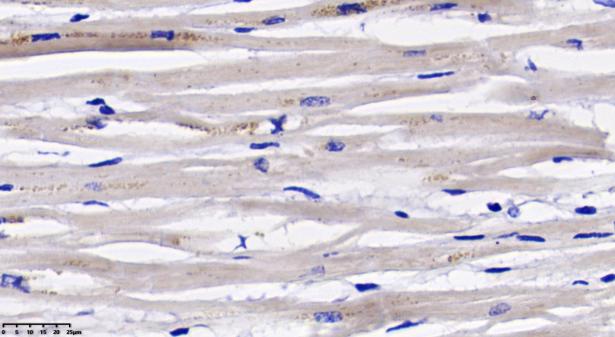** |
| 4-1 Scale 50μm | 4-2 Scale 25μm |
| **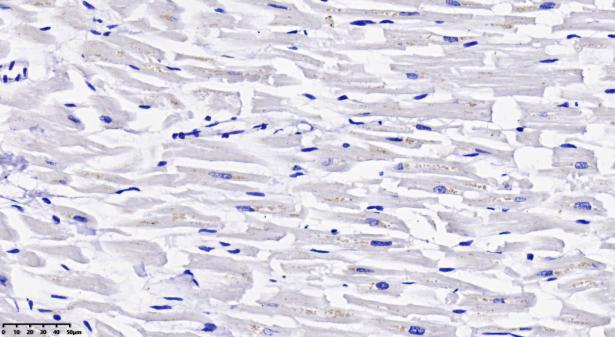** | **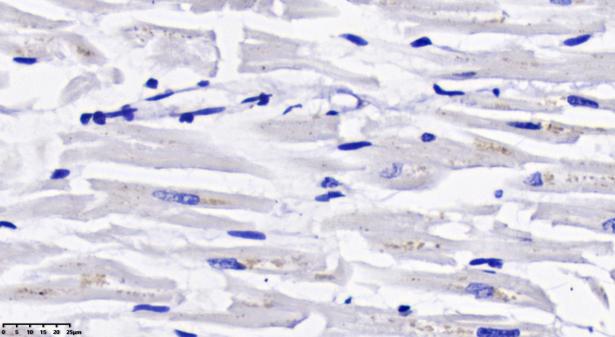** |
| 5-1 Scale 50μm | 5-2 Scale 25μm |
| **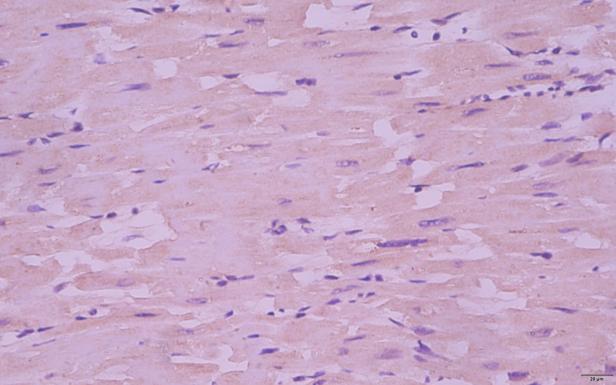** | **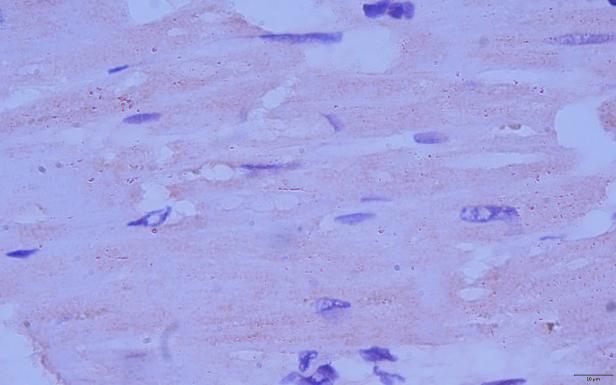** |
| 6-1 Scale 20μm | 6-2 Scale 10μm |

| **Per-scar** | |
| --- | --- |
| **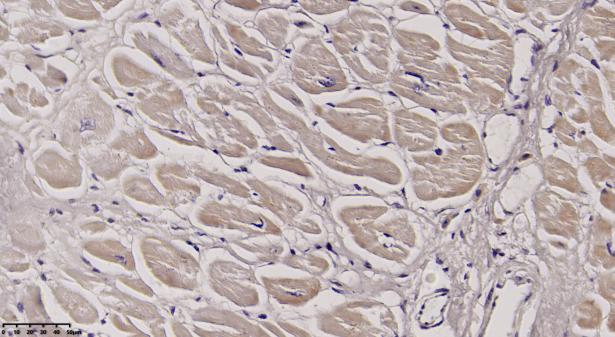** | **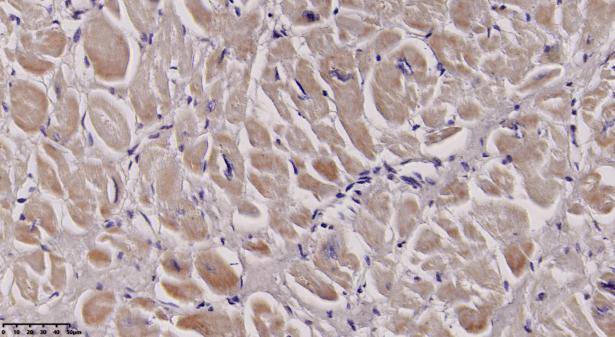** |
| Per-scar-1 | Per-scar-2 |
| **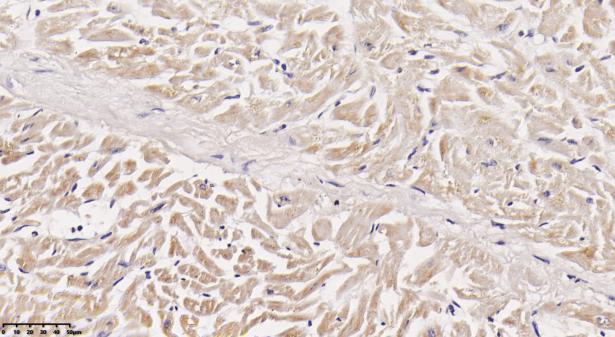** | **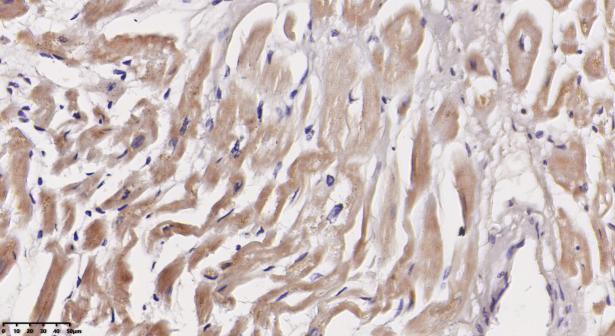** |
| Per-scar-3 | Per-scar-4 |
| **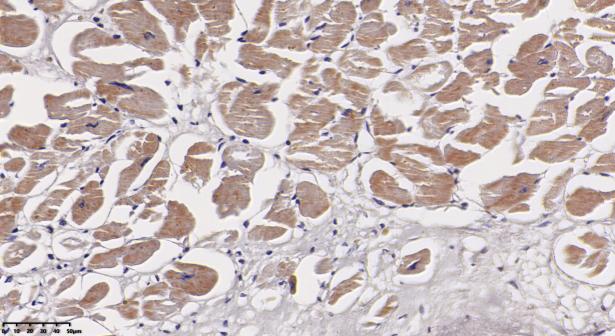** | **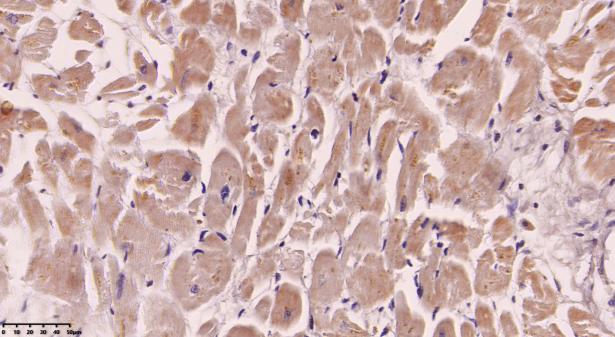** |
| Per-scar-5 | Per-scar-6 |

# Figure. 5 Fluo-4

| **Control** | | |  |
| --- | --- | --- | --- |
| **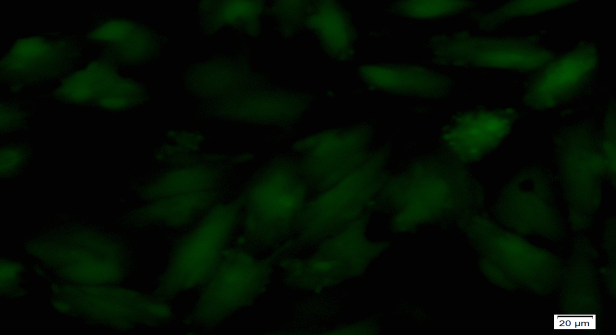** | **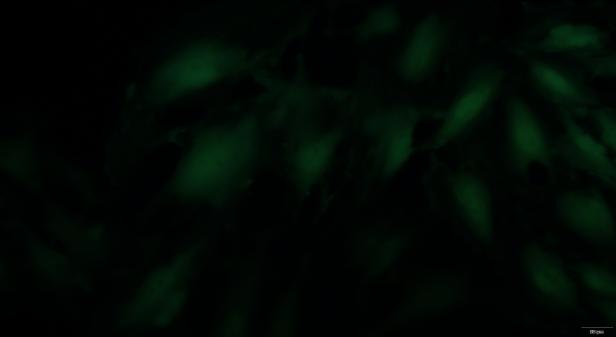** | |  |
| **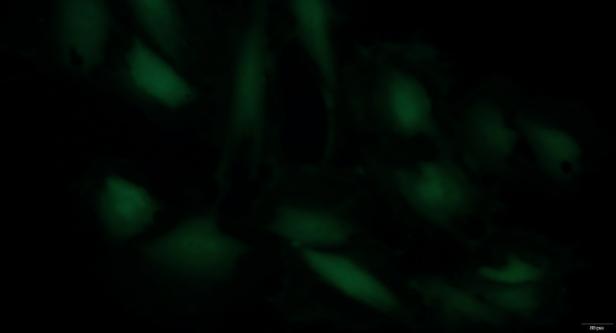** | **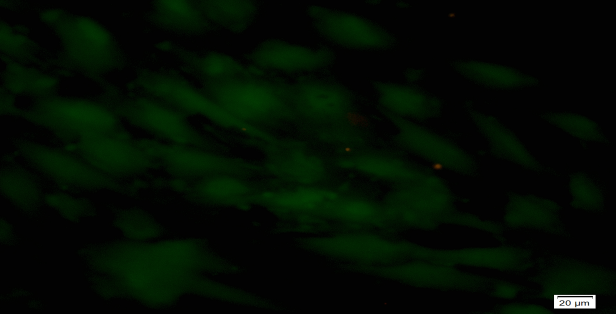** | |  |
| **oe MOCK** | | | |
| **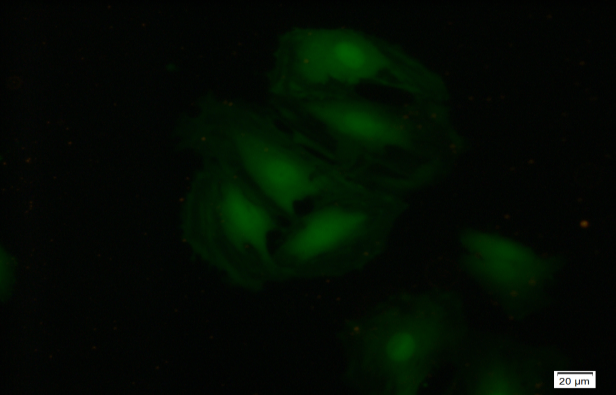** | | **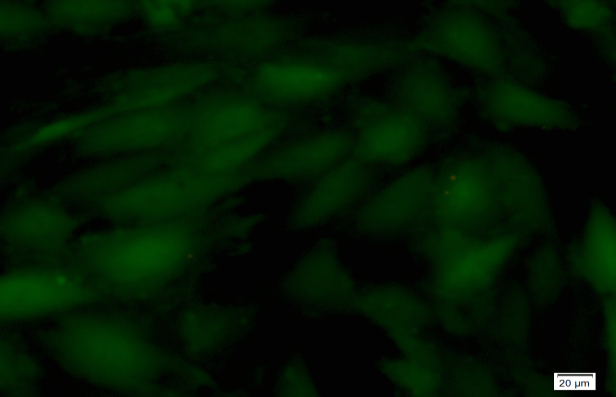** | |
| **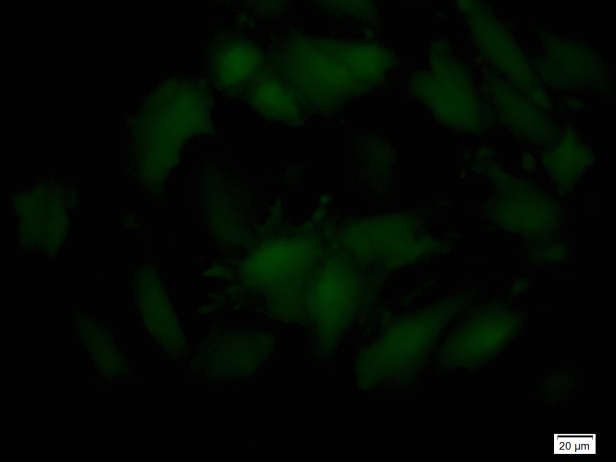** | | **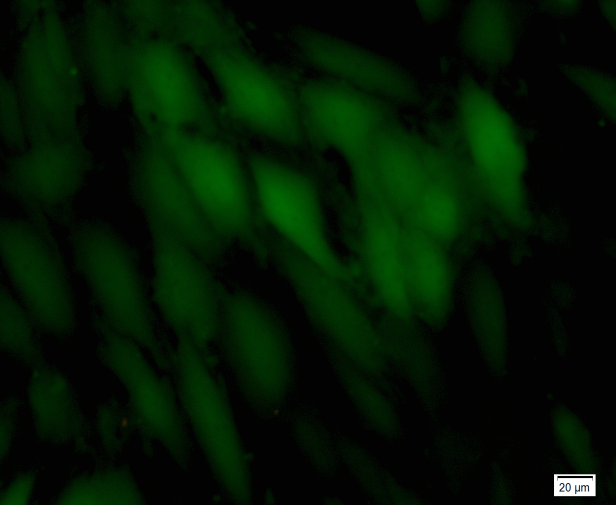** | |
| **oe-klhl40** | | | |
| **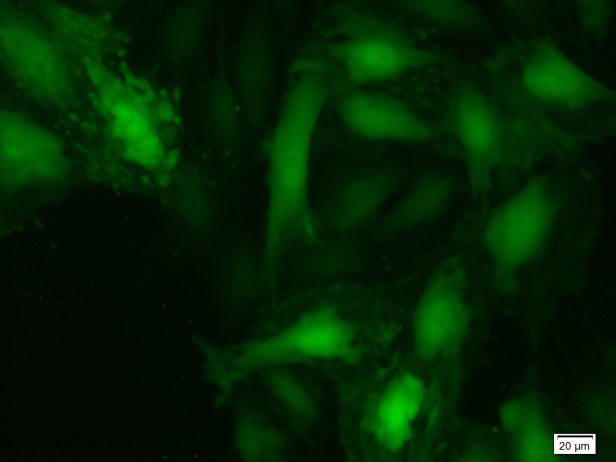** | | **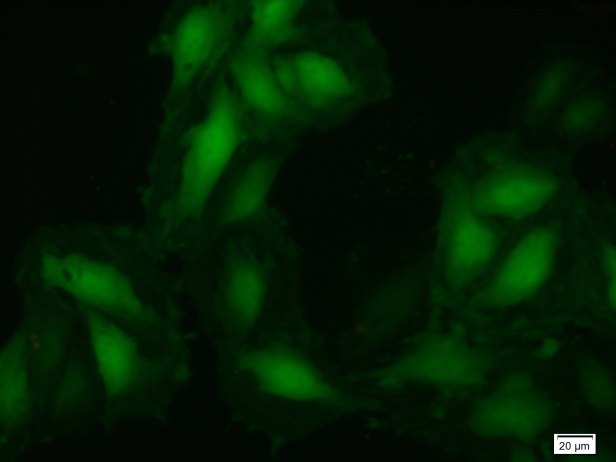** | |
| **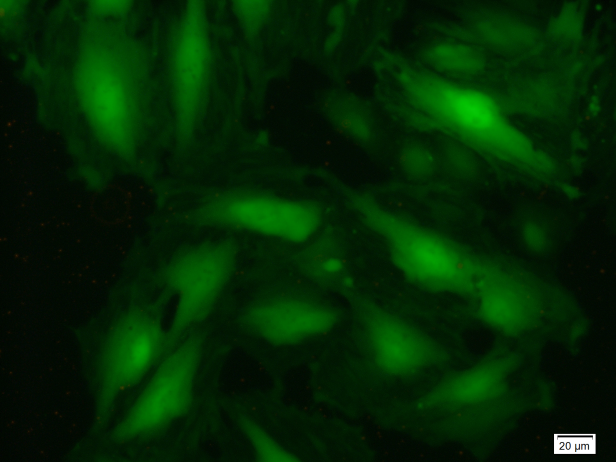** | | **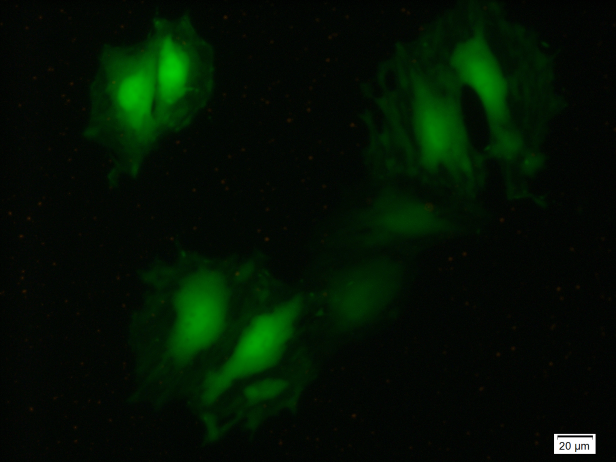** | |
| **sh-klhl40** | | | |
| **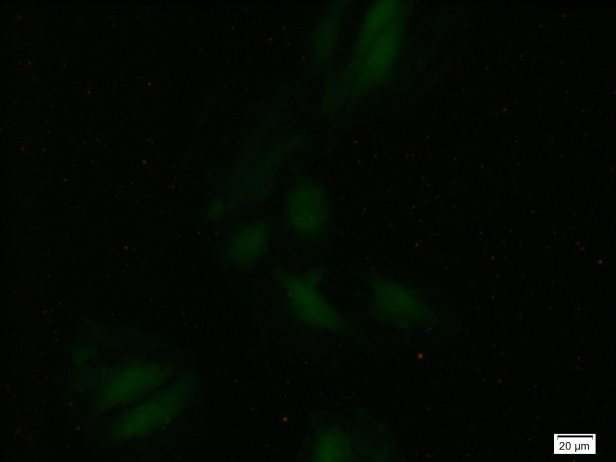** | | **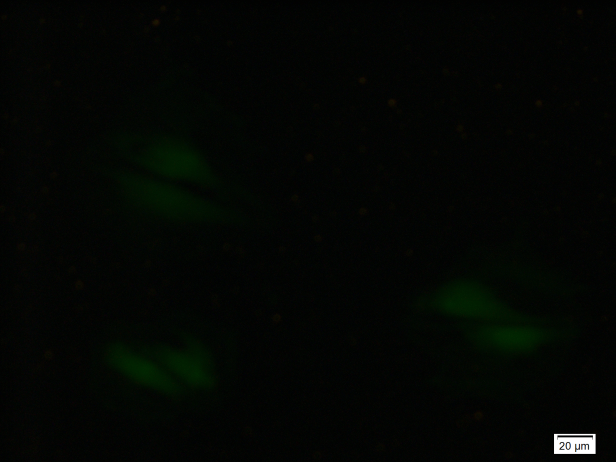** | |
| **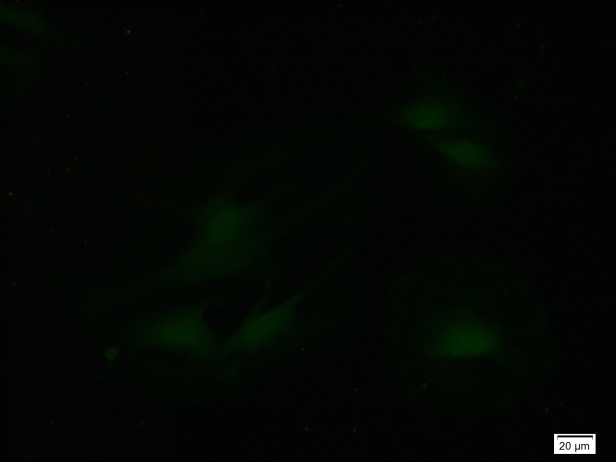** | | **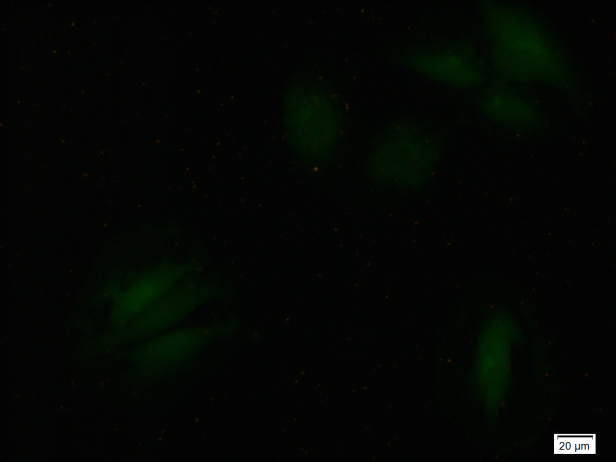** | |

| **sh-klhl40-NC** | |
| --- | --- |
| **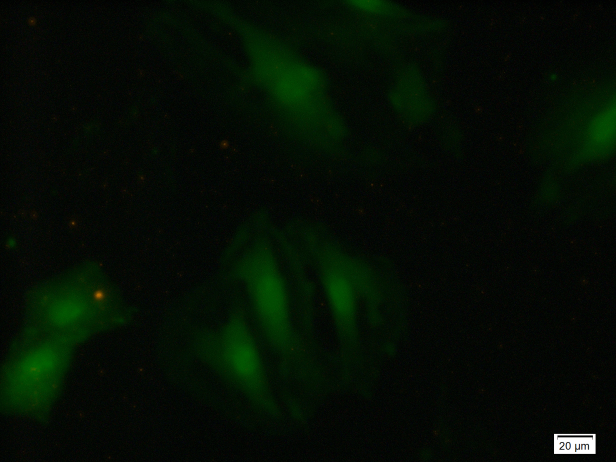** | **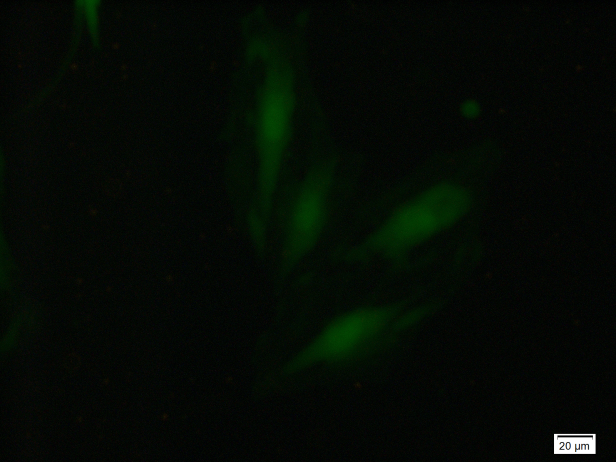** |
| **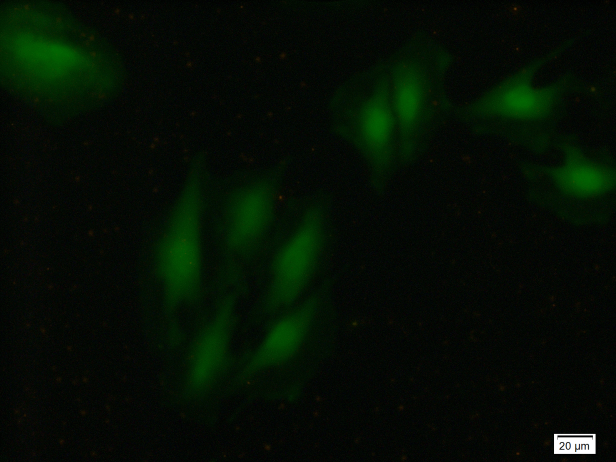** | **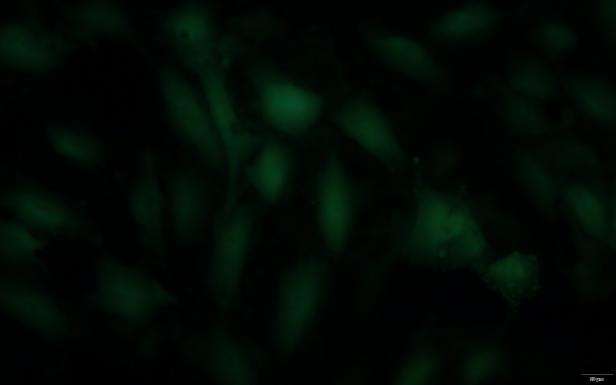** |

# Figure. 7, H and M Morphology

| **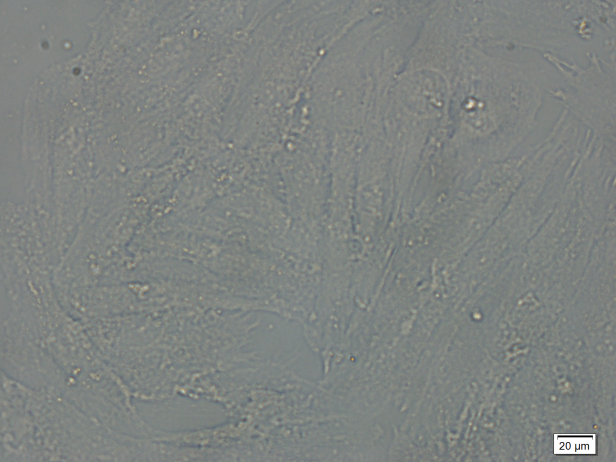** | **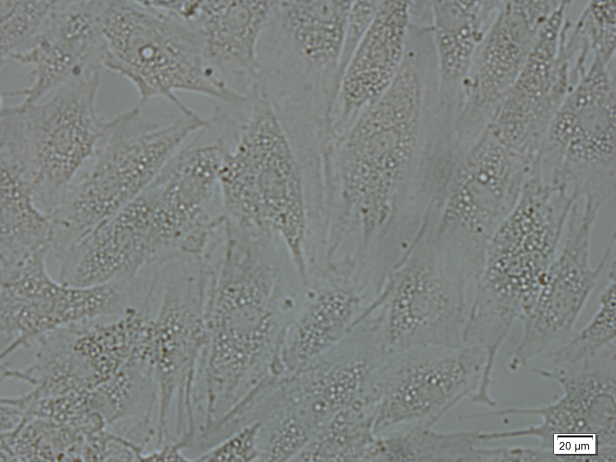** |
| --- | --- |
| Chloroquine 0μM | Chloroquine 50μM |
| 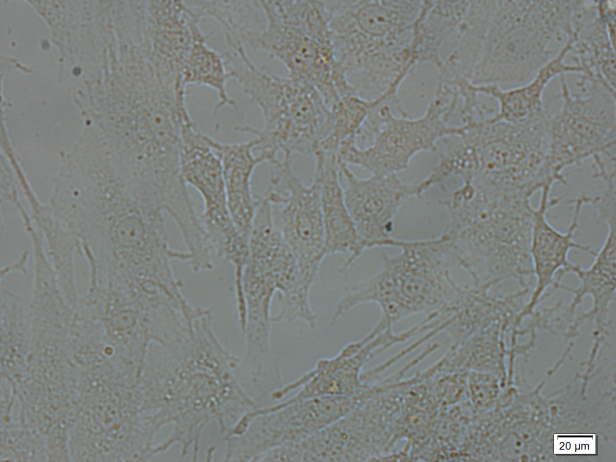 | 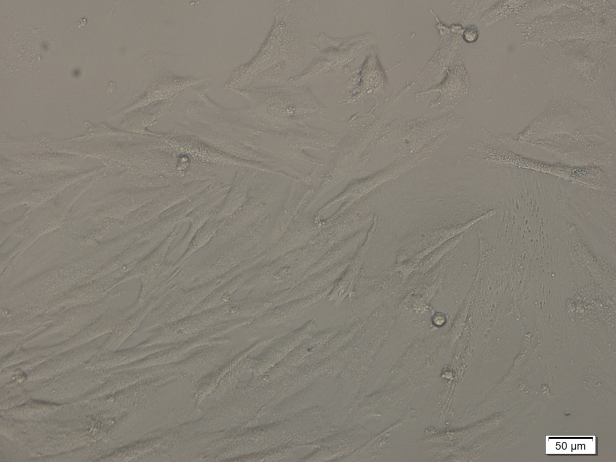 |
| Chloroquine 100μM | MG132 0μM |
| 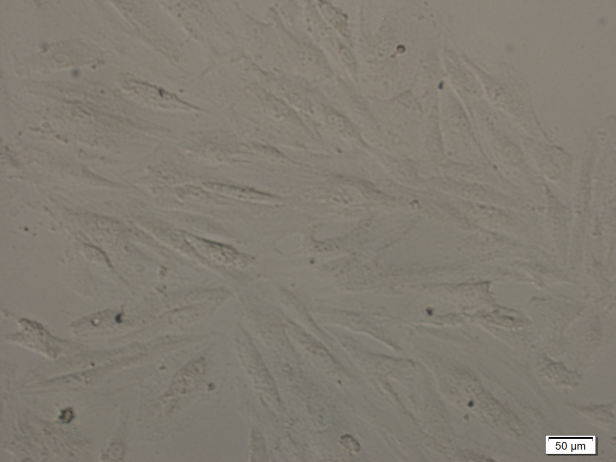 | 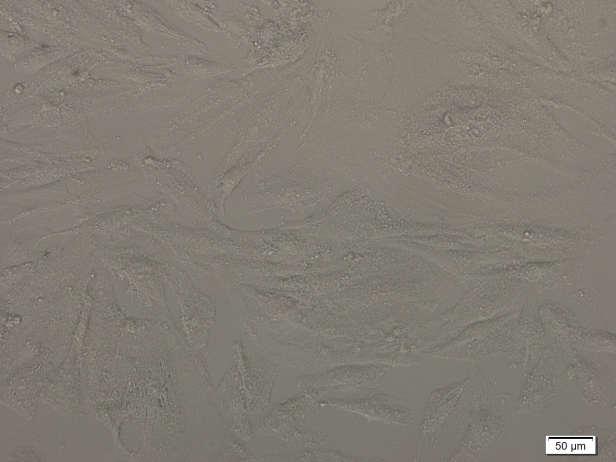 |
| MG132 5μM | MG132 10μM |

# Heart view from all perspectives

**
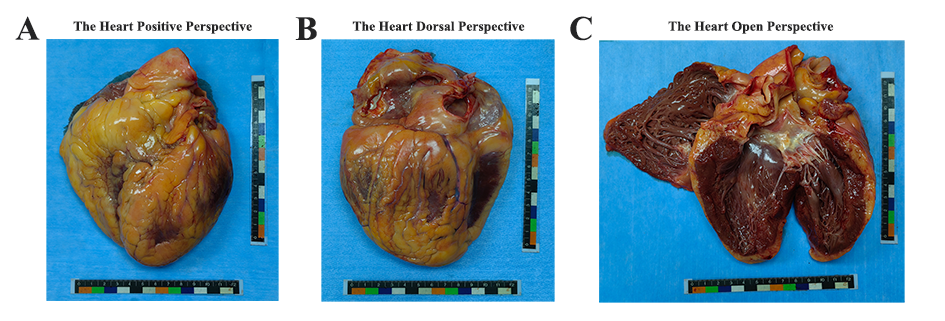
**

**Dalian Medical University Biomedical Ethics Committee Review Decision**
**Decision No.: DMU-EC2024-039**

**To: Yu Xiao**

Regarding your manuscript titled "KLHL40-Mediated Regulation of Cardiovascular Integrity and Function After Myocardial Infarction", which was submitted for publication, the Dalian Medical University Biomedical Ethics Committee has reviewed the manuscript and reached the following decision:

**1.The manuscript has passed the ethical review and is approved for publication.√**

**2.The manuscript has passed the ethical review in principle and is approved for publication after revisions.**

**3.The manuscript has not passed the ethical review and is not approved for publication.**

**Dalian Medical University Biomedical Ethics Committee**


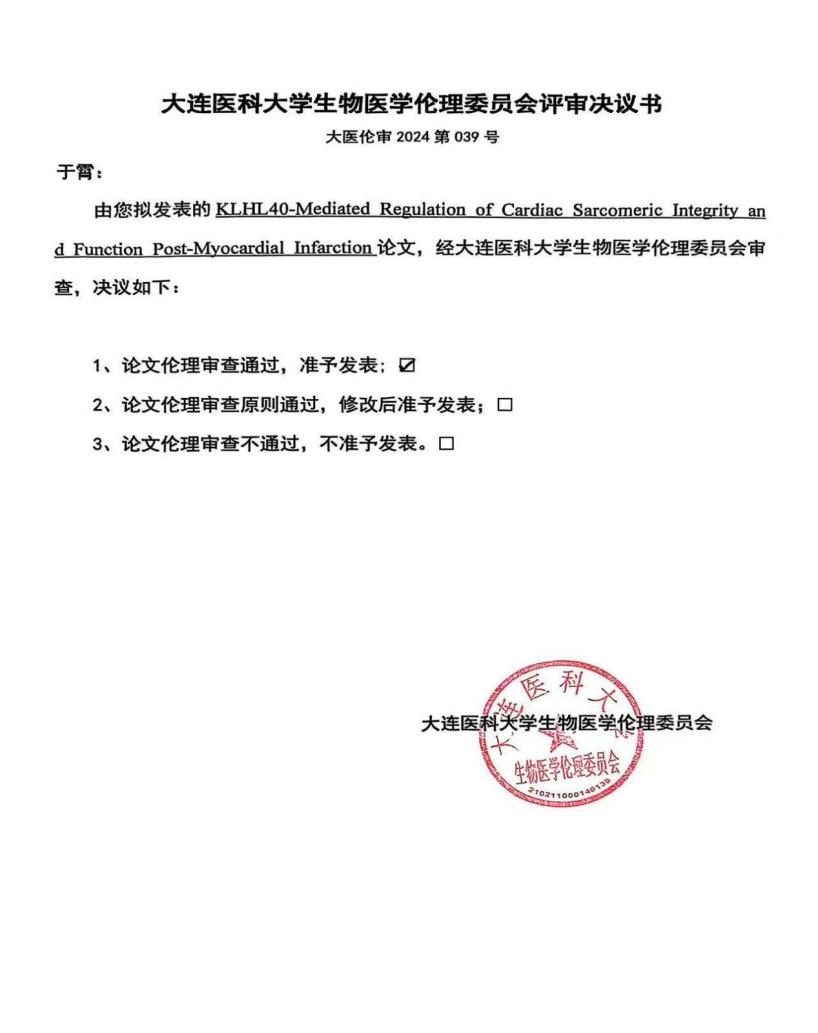


# Informed Consent

Dear Participant,

We are currently conducting a study titled "The Mechanism of KLHL40 in Sarcomere Remodeling and Calcium Signaling Regulation After Myocardial Infarction."

Before you decide to participate, please carefully read this informed consent form and consider whether you wish to join this study. You may ask the researcher in charge any questions about aspects you do not fully understand. If you are currently participating in other research studies, please inform the responsible researcher.

**1. Research Background and Objectives**

Following myocardial infarction (MI), the structure and function of cardiomyocytes undergo significant changes, particularly in sarcomeric protein remodeling and calcium ion homeostasis disruption, which are critical factors in cardiac functional deterioration. KLHL40 is an important protein expressed in muscle tissue, and previous studies have demonstrated its key role in skeletal muscle development. This study aims to investigate its mechanistic role in Z-disc protein degradation and calcium signaling regulation after MI. By utilizing residual postmortem myocardial tissue samples, we seek to elucidate the protein’s potential function in disease progression, providing a theoretical foundation for future therapeutic strategies.

**2. Study Procedures**This research utilizes residual postmortem myocardial tissue samples obtained during autopsy. No additional interventions or procedures will be performed on participants.

**3. Potential Benefits**The findings may advance the understanding of myocardial infarction mechanisms and provide a scientific basis for early intervention and therapeutic strategies, offering potential societal value.

**4. Risks and Compensation**As this study exclusively involves postmortem tissue specimens, there are no foreseeable physical risks, privacy breaches, or harm to the deceased individuals.

**5. Participant Responsibilities**No specific requirements apply before, during, or after the study, as only archived tissue samples are analyzed.

**6. Privacy and Confidentiality**All personal information linked to the deceased donors will remain strictly confidential. Data will be securely encrypted and accessible only to the research team. Any published results will be anonymized, with no identifiable information disclosed. In compliance with legal regulations, the privacy of donors and their families will be protected. Families may request access to or corrections of their relative’s data.

**7. Your Rights**Participation is entirely voluntary. You retain the right to withdraw consent at any time. During the study, you may request additional information or address concerns by contacting the principal investigator.

**8. Contact Information**For questions, please contact:
Dr. Xiao Yu
Affiliation: 9 West Section, Lvshun South Road, Dalian, Liaoning, China
Tel: *+86-411-86110297*

**Consent Statement**

As the legal next-of-kin, I have read and understood the purpose, risks, and benefits of this study. I voluntarily consent to provide relevant information and my deceased relative’s myocardial tissue samples for the research titled "The Mechanism of KLHL40 in Sarcomere Remodeling and Calcium Signaling Regulation After Myocardial Infarction."

Family Representative Signature: _________ Date: _________

**Investigator Declaration:**We have thoroughly explained this consent form and addressed all questions. The family representative fully comprehends and agrees to participate.

Principal Investigator Signature: _________ Date: _________

# Figure S 5 Sanger Sequencing

1.F stands for primer forward and R stands for primer reverse
 The number after the primer sequence represents: primer start position - primer end position = product length (e.g. 210-630 = 421)

It is not for genomic location, only for the design protocol, so the location can be ignored

2. Order number - primer number - sample number - date sent for testing - primer sequencing direction - well number
Example: SC842-1-1-3.6X-1F_F03

3. The sample number SCD651

| \| **Client Number** \| \| --- \| | 1 | 2 | 3 |
| --- | --- | --- | --- | --- |
| 1 | Not knocked out | Not knocked out | Not knocked out |
| 2 | Not knocked out | Not knocked out | Not knocked out |
| 3 | \| Knocked out \| \| --- \| |  |  |
| 4 |  | \| Knocked out \| \| --- \| |  |
| 5 |  |  | \| Knocked out \| \| --- \| |

4.
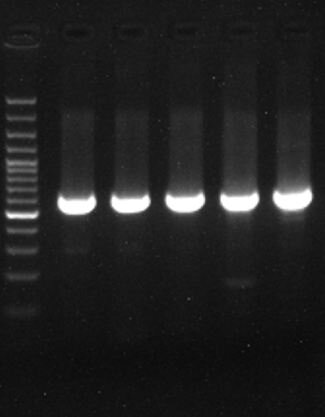
SCD651
