# Supplementary material for: Post-infarction KLHL40-mediated regulation of cardiac sarcomeric integrity and function
Source: PeerJ. 2026 Jun 5;14:e21375. doi: 10.7717/peerj.21375 (PMC13245431; doi:10.7717/peerj.21375)
Supplement: Supplemental Information 12 [file peerj-14-21375-s012.zip › Figure S 5 Sanger Sequencing.docx]

# Figure S 5 Sanger Sequencing

1.F stands for primer forward and R stands for primer reverse
 The number after the primer sequence represents: primer start position - primer end position = product length (e.g. 210-630 = 421)

It is not for genomic location, only for the design protocol, so the location can be ignored

2. Order number - primer number - sample number - date sent for testing - primer sequencing direction - well number
Example: SC842-1-1-3.6X-1F_F03

3. The sample number SCD651

| \| **Client Number** \| \| --- \| | 1 | 2 | 3 |
| --- | --- | --- | --- | --- |
| 1 | Not knocked out | Not knocked out | Not knocked out |
| 2 | Not knocked out | Not knocked out | Not knocked out |
| 3 | \| Knocked out \| \| --- \| |  |  |
| 4 |  | \| Knocked out \| \| --- \| |  |
| 5 |  |  | \| Knocked out \| \| --- \| |

4.
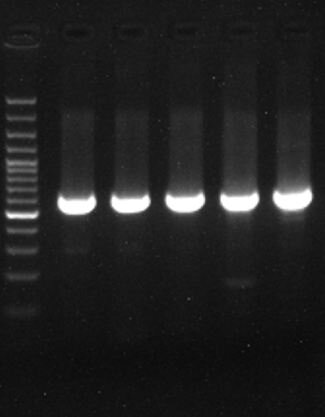
SCD651
