# Supplementary material for: Post-infarction KLHL40-mediated regulation of cardiac sarcomeric integrity and function
Source: PeerJ. 2026 Jun 5;14:e21375. doi: 10.7717/peerj.21375 (PMC13245431; doi:10.7717/peerj.21375)
Supplement: Supplemental Information 17 [file peerj-14-21375-s017.zip › Figure 2+Figure 3 Labeled Western blot.docx]

# Figure. 2I Wb Early of MI

| 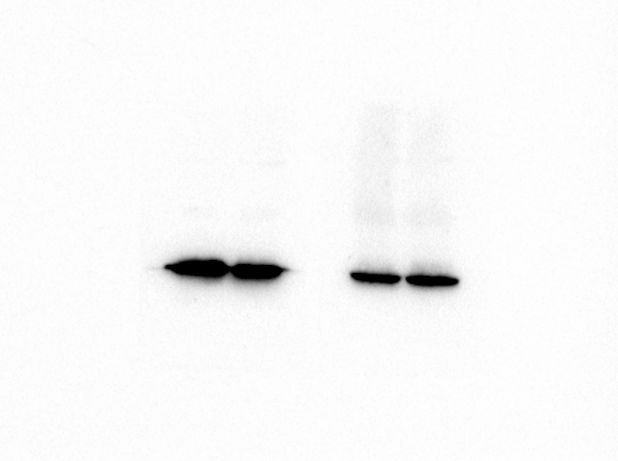 | 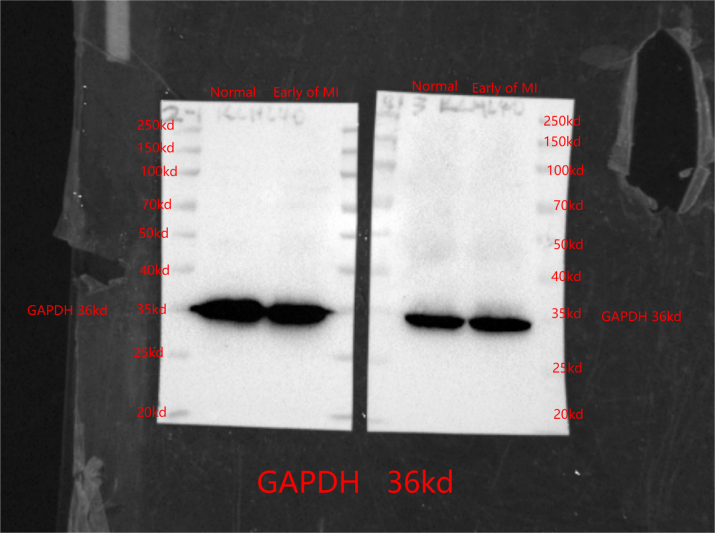 |
| --- | --- |
| GAPDH | GAPDH+Mark |
| 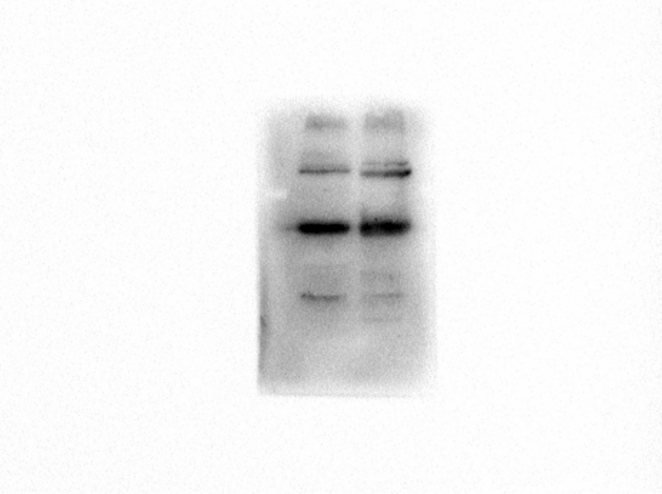 | 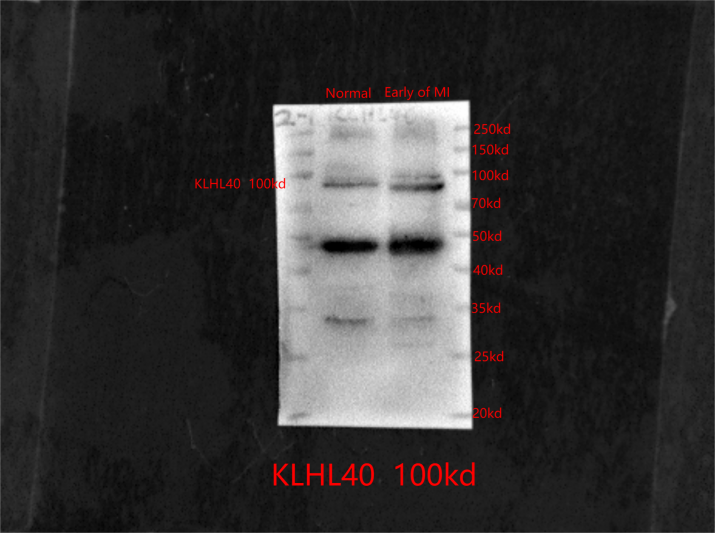 |
| KLHL40-1 | KLHL40-1 Merge |
| 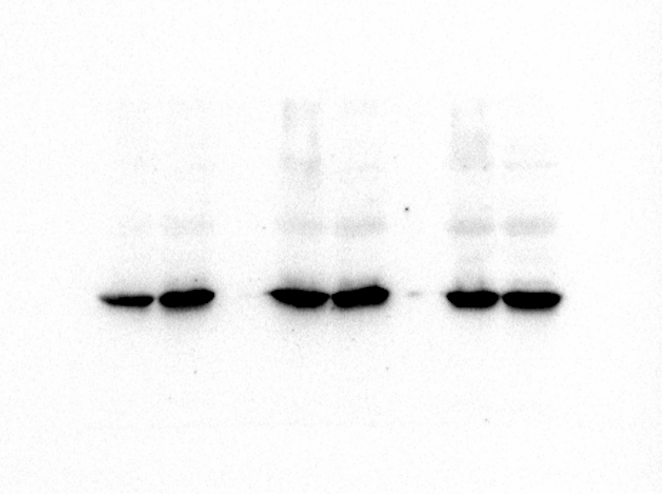 | 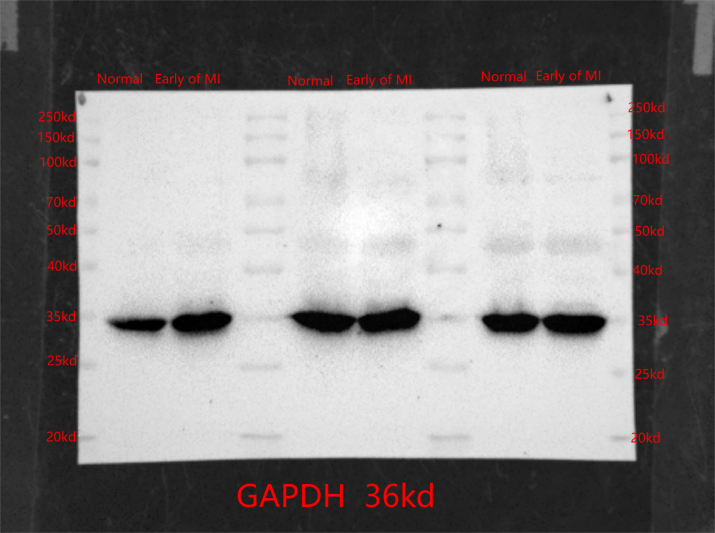 |
| KLHL40-2-3 GAPDH | KLHL40-2-3 GAPDH merge |
| 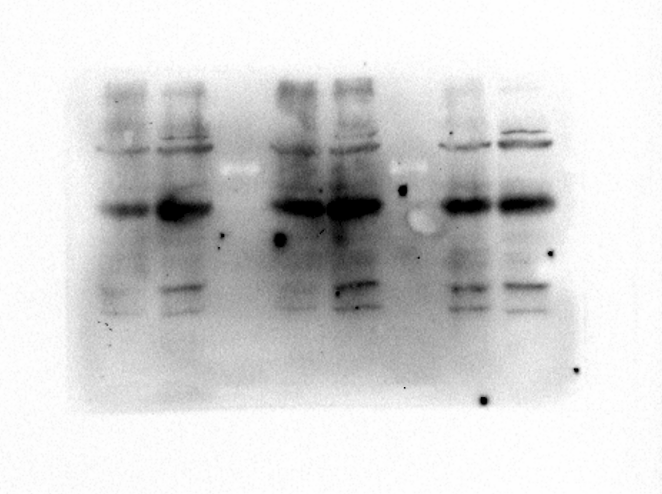 | 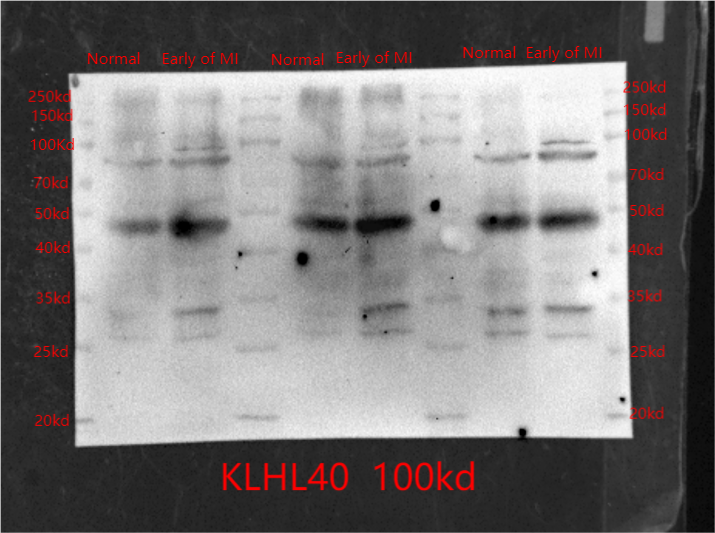 |
| KLHL40-2-3 | KLHL40-2-3 MARK |
| 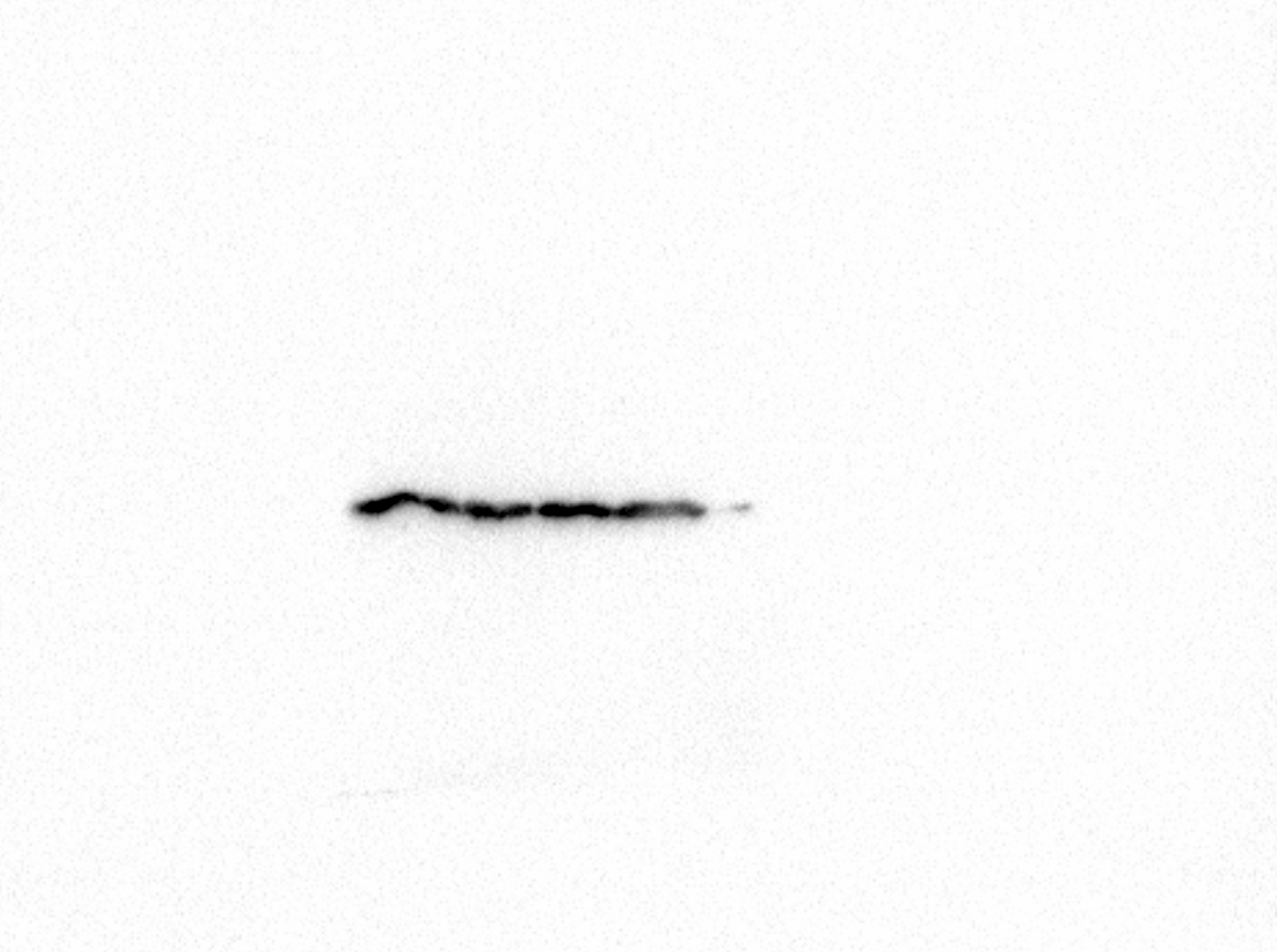 | 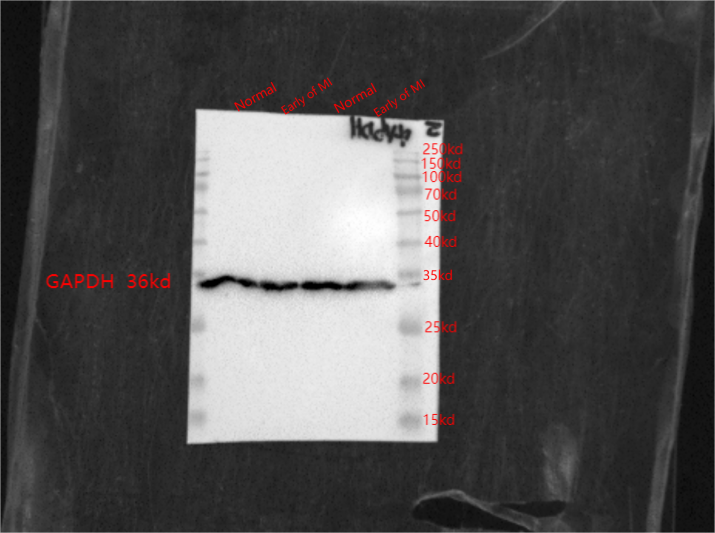 |
| klhl40-4 gapdh | klhl40-4 gapdh MARK |
| 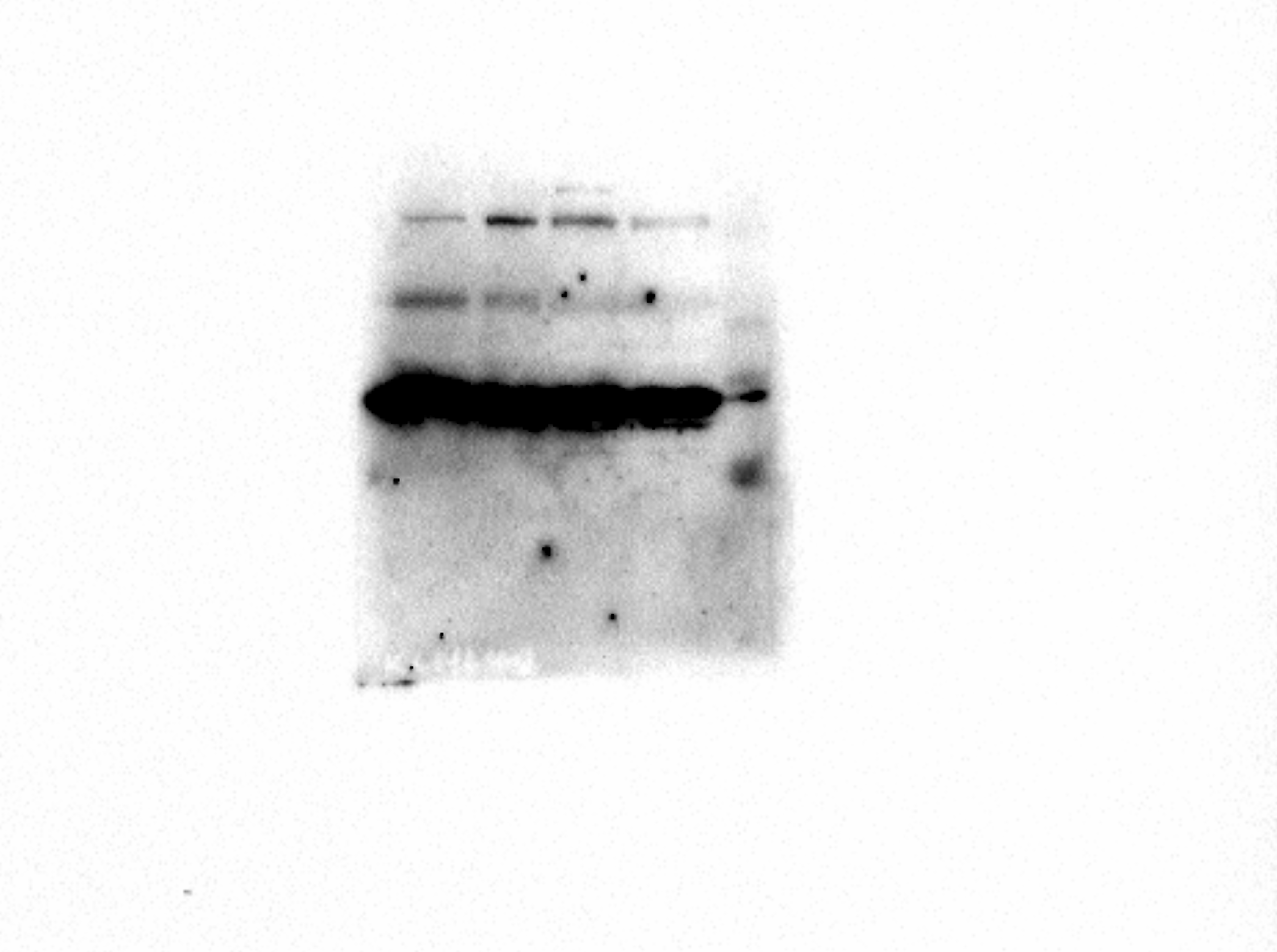 | 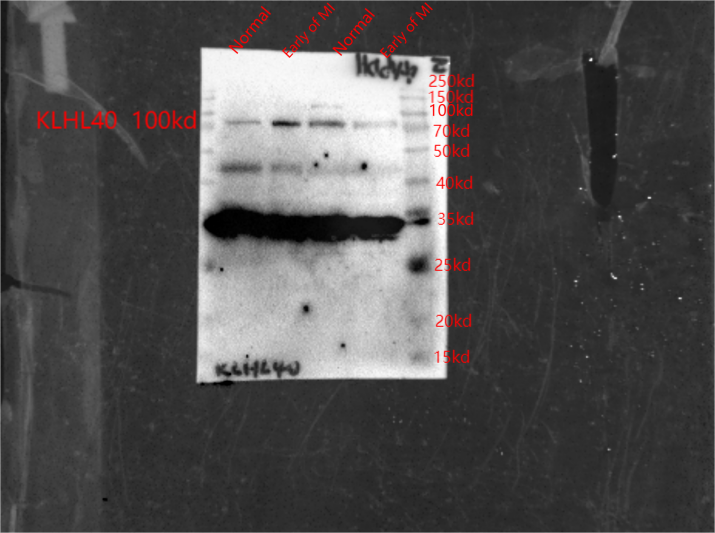 |
| KLHL40-4 | KLHL40-4 MA |
| 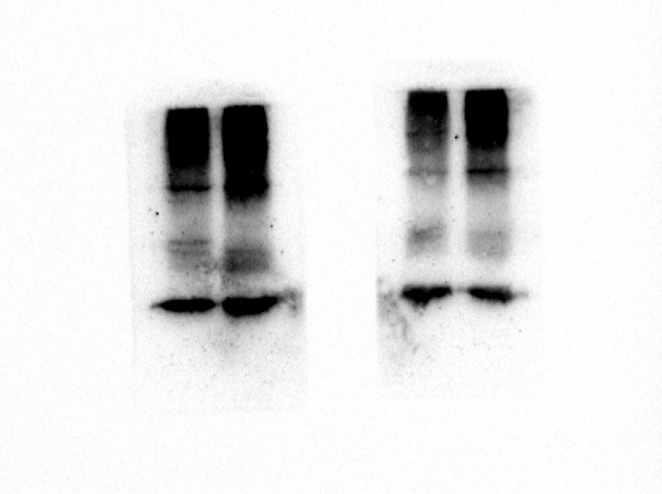 | 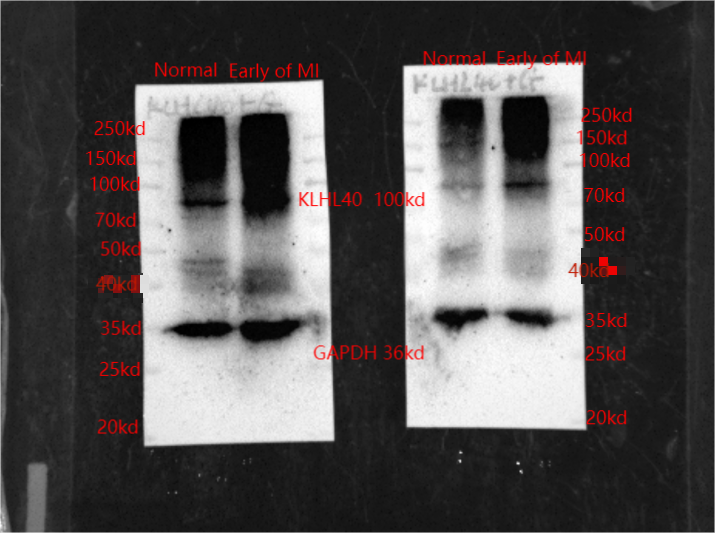 |
| KLHL40-5 AND GAPDH | KLHL40-5 AND GAPDH MARK |

# Figure. 2J Wb Late of MI

| **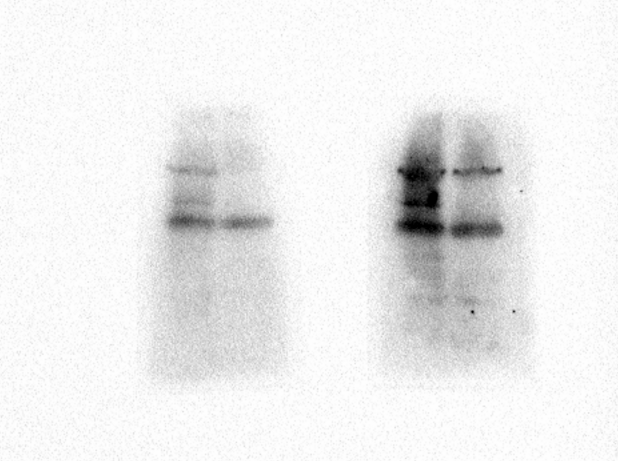** | **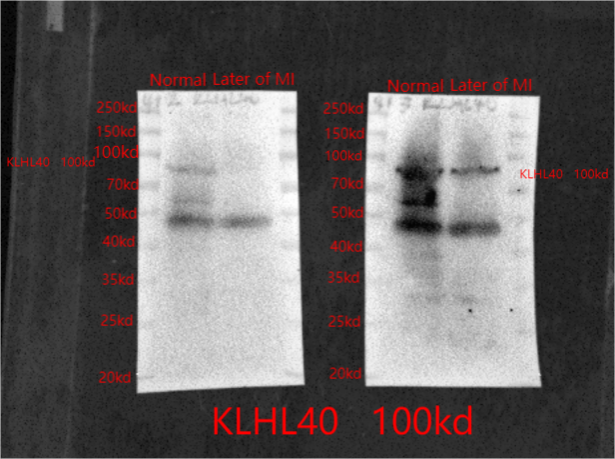** |
| --- | --- |
| klhl40-1 | klhl40-1 MARK |
| **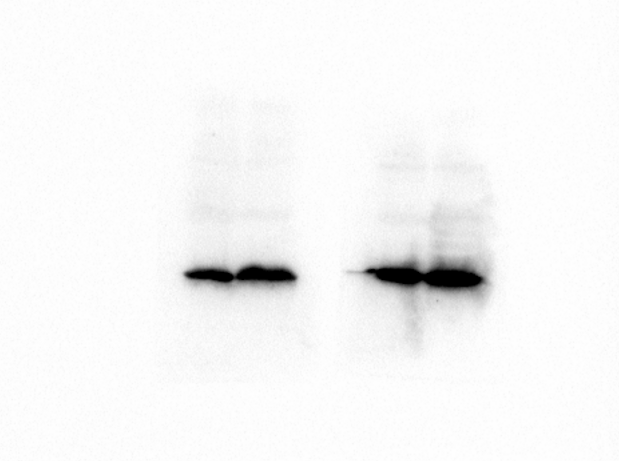** | **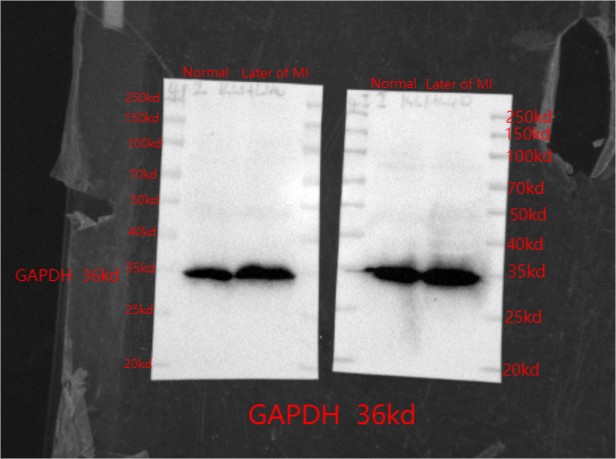** |
| KLHL40-1 GAP | KLHL40-1 GAP MARK |
| **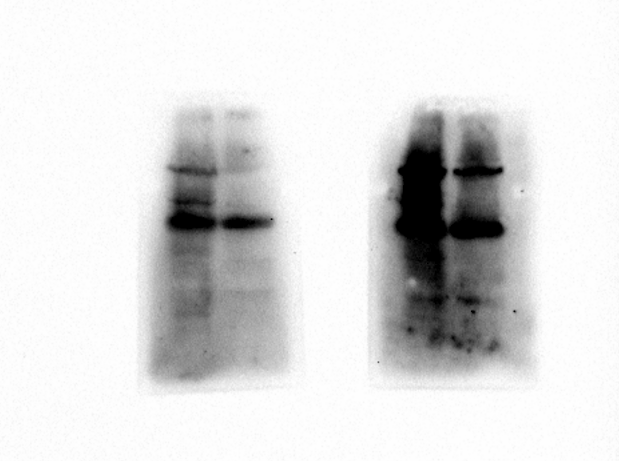** | **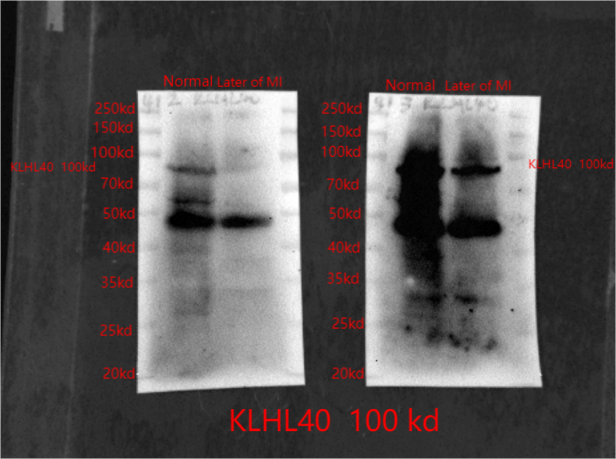** |
| klhl40-1 | klhl40-1MARK |
| **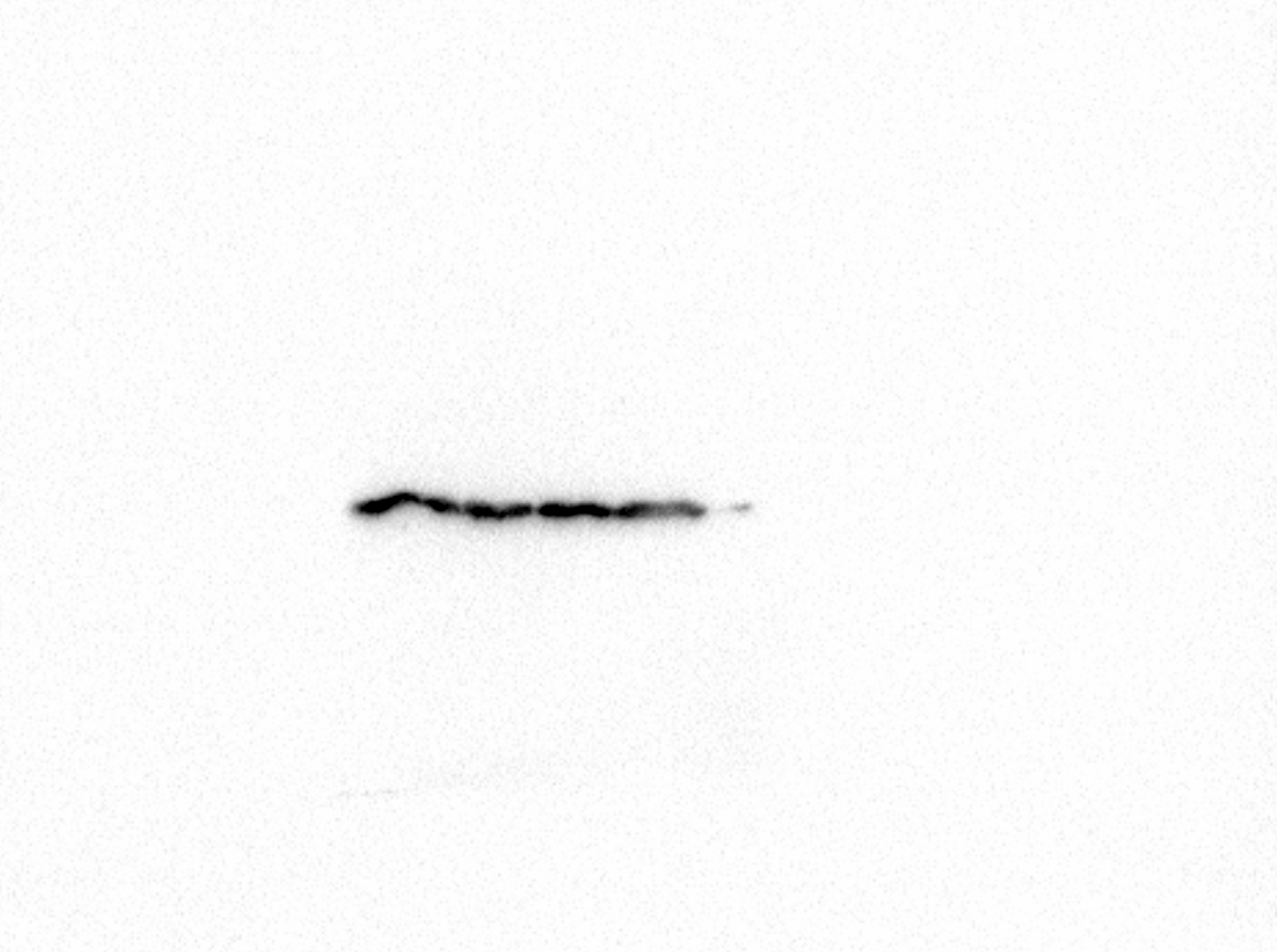** | **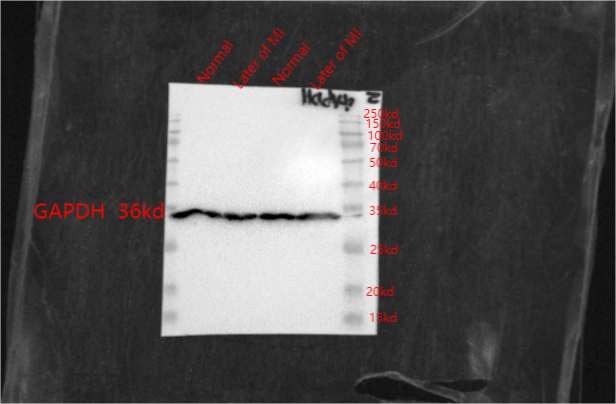** |
| klhl40-2 gapdh | klhl40-2 gapdh MARK |
| **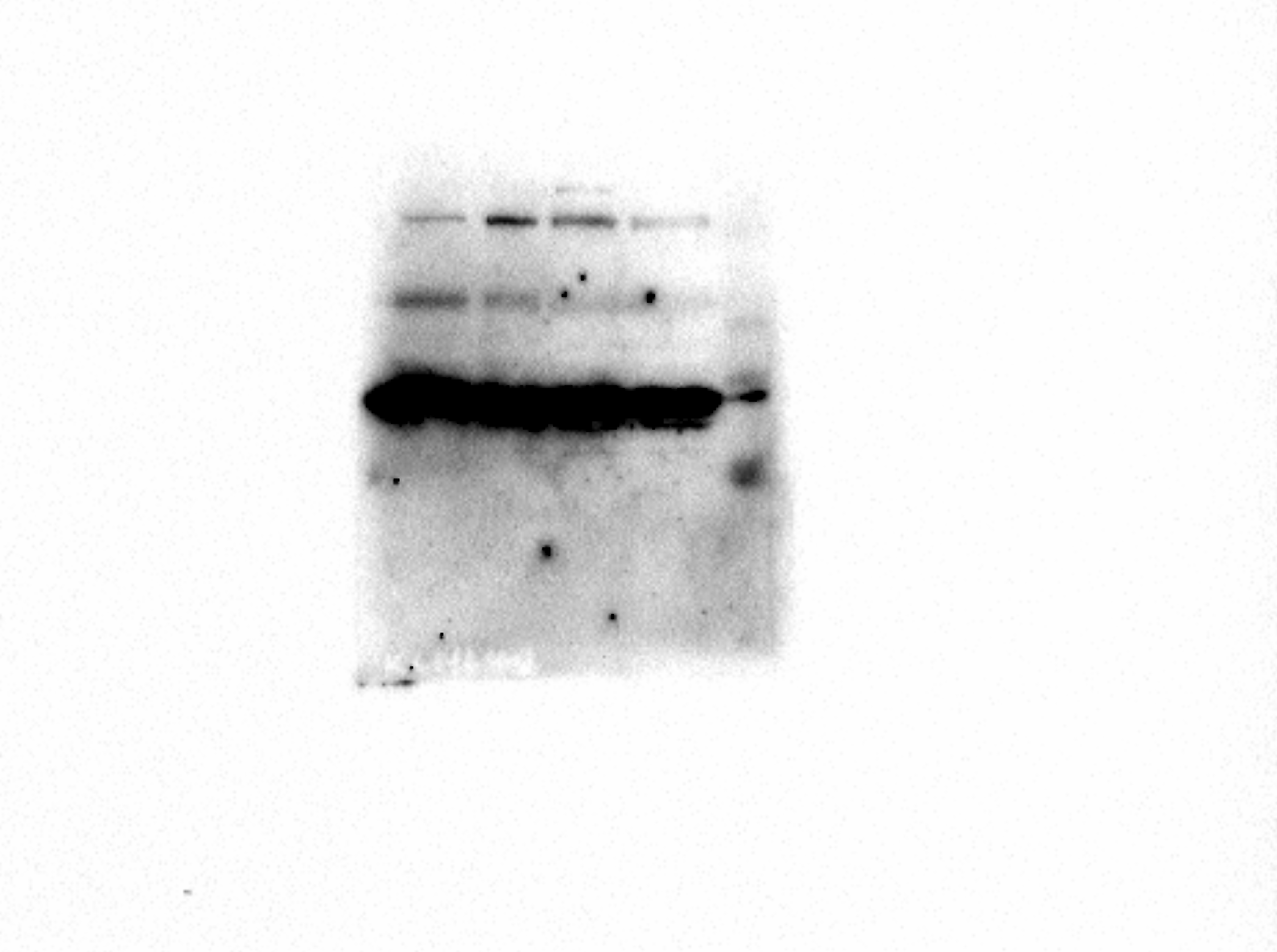** | **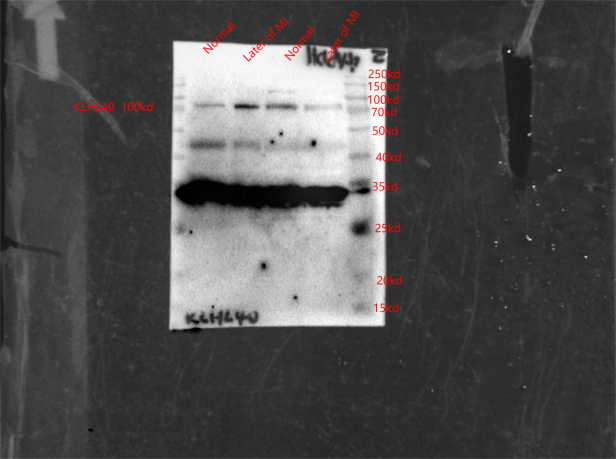** |
| KLHL40-2 | KLHL40-2 MARK |
| **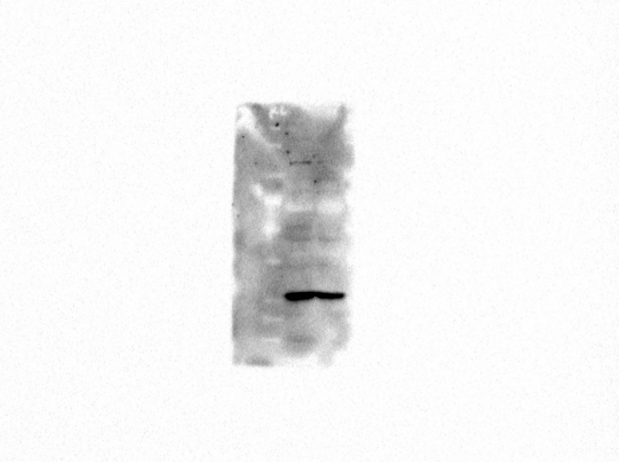** | **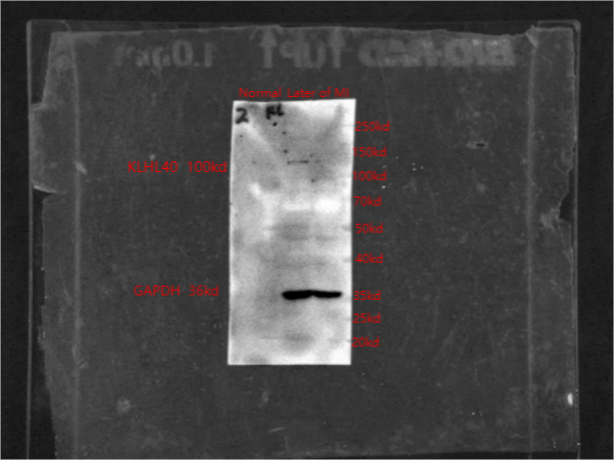** |
| klhl40-3+gap | klhl40-3+gap MARK |
| **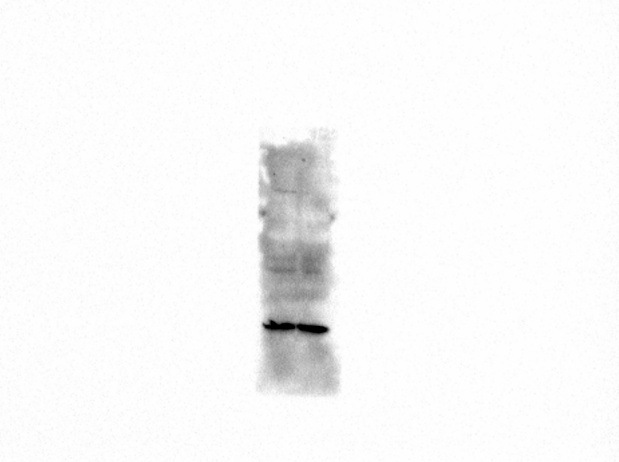** | **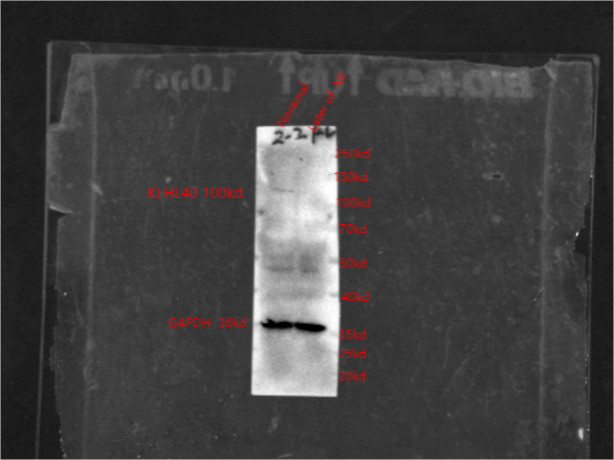** |
| klhl40-4+gap | klhl40-4+gap MARK |

# Figure. 2M Wb 0-24 hypoxia

| KLHL40 | KLHL40+MARKER | β-ACTIN | β-ACTIN+MARKER | ALL |
| --- | --- | --- | --- | --- |
| 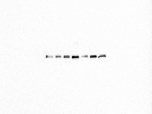 | 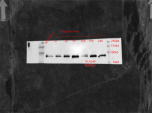 | 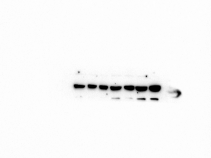 | 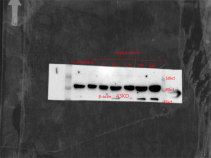 | 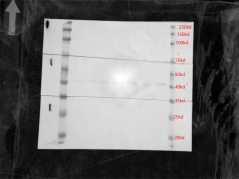 |
| 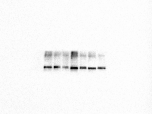 | 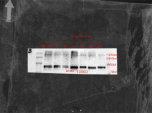 | 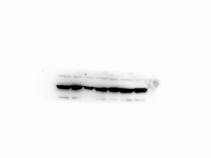 | 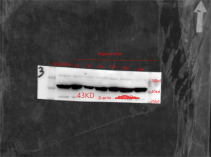 | 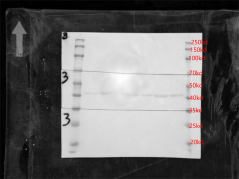 |
| 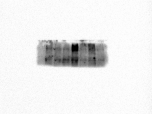 | 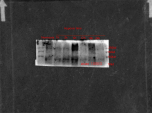 | 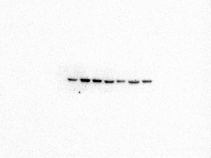 | 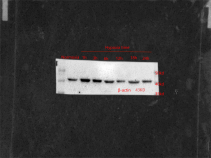 | 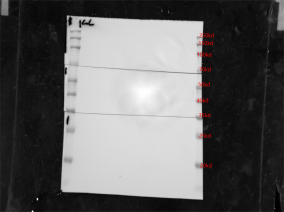 |

# Figure.3H Wb SH-KLHL40

| KLHL40 | KLHL40+MARKER | β-ACTIN | β-ACTIN+MARKER | ALL |
| --- | --- | --- | --- | --- |
| 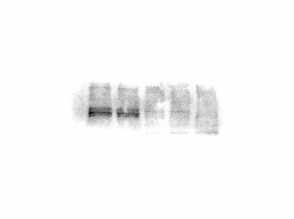 | 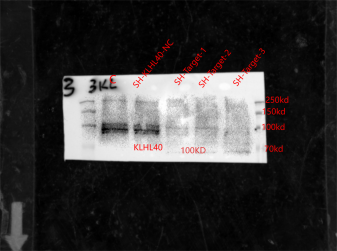 | 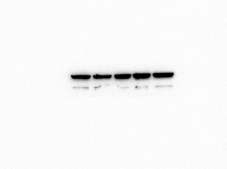 | 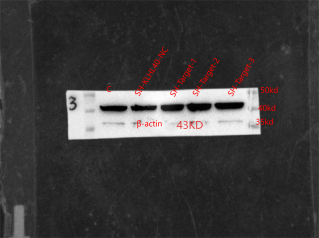 | 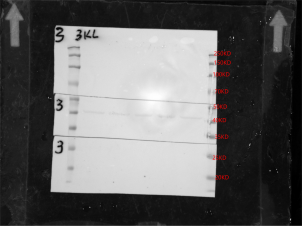 |
| 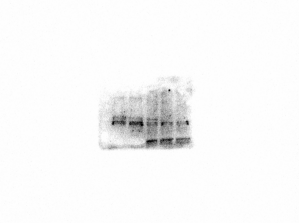 | 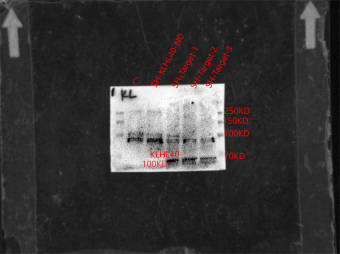 | 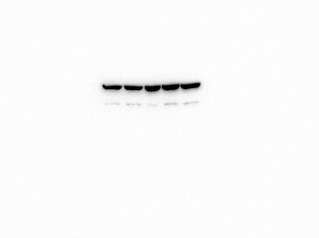 | 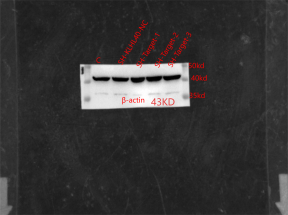 | 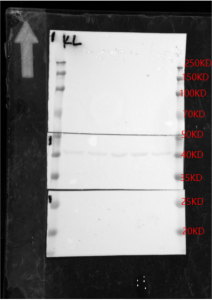 |
| 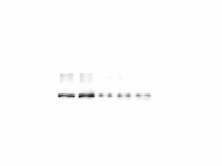 | 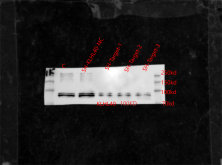 | 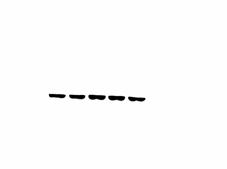 | 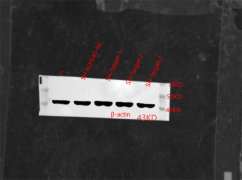 | 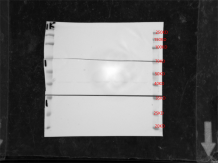 |

# Figure. 3J Wb oe-KLHL40

| **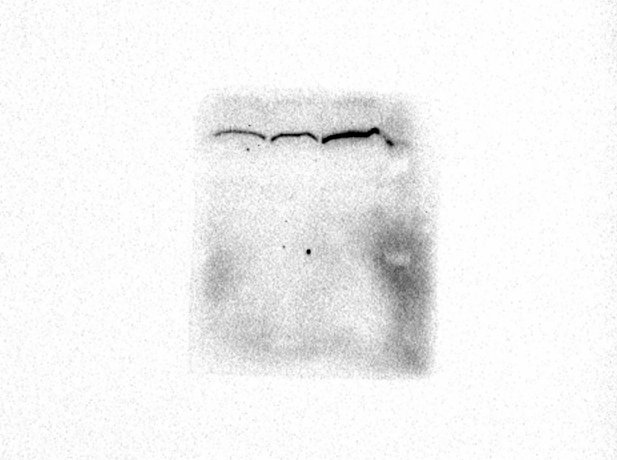** | **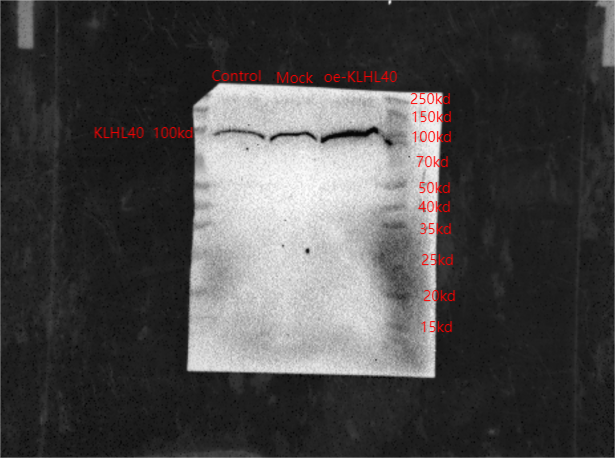** |
| --- | --- |
| KLHL40-1 | KLHL40-1+MARK |
| 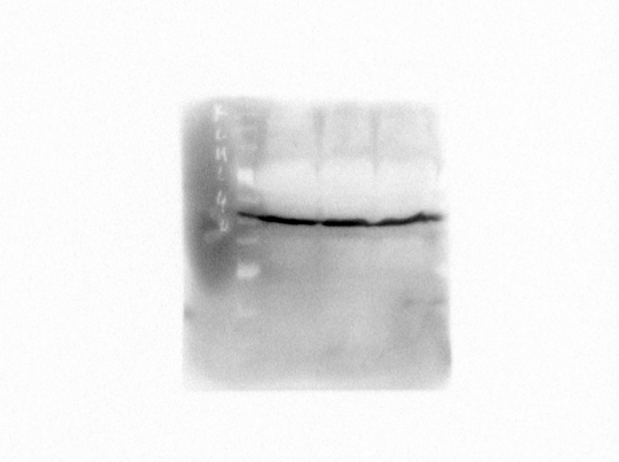 | 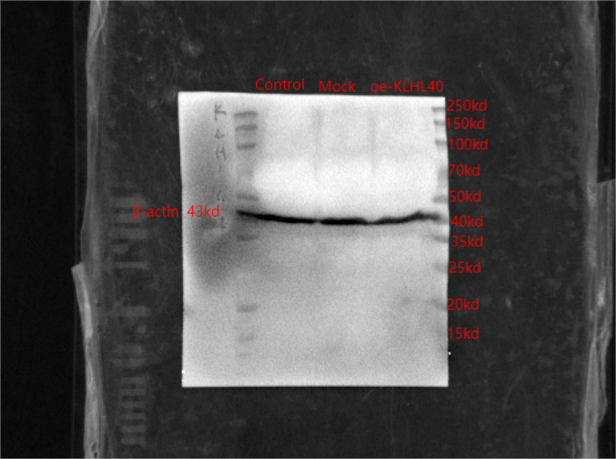 |
| KLHL40-1-ACTB | KLHL40-1-ACTB+MARK |
| 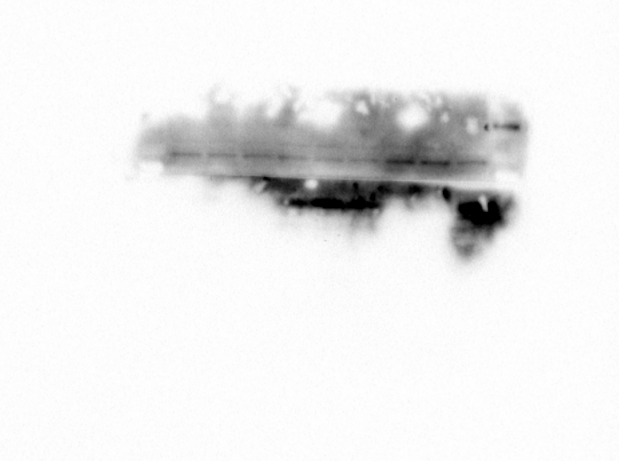 | 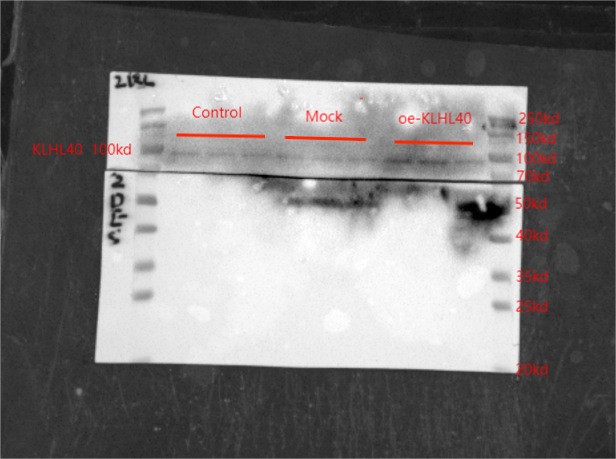 |
| KLHL40-2 CCC MMM OEOEOE | KLHL40-2+MARK CCC MMM OEOEOE |
| **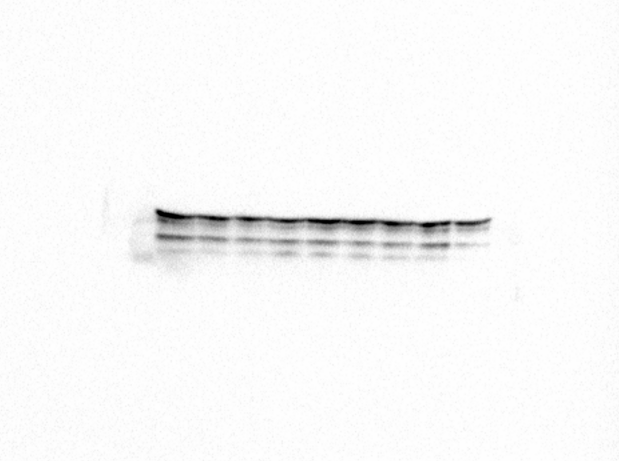** | **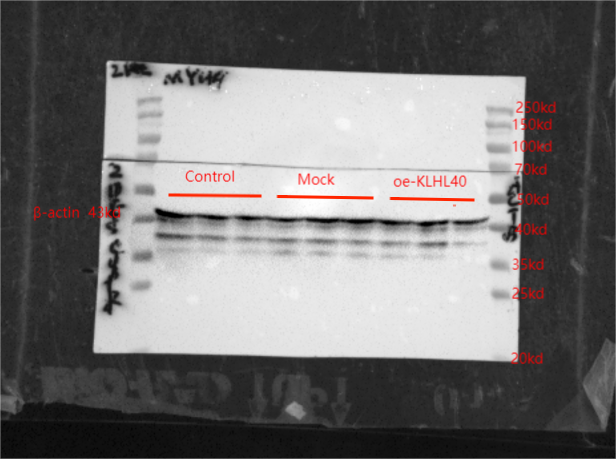** |
| KLHL40-2-ACTB CCC MMM OEOEOE | KLHL40-2-ACTB+MARK CCC MMM OEOEOE |
| 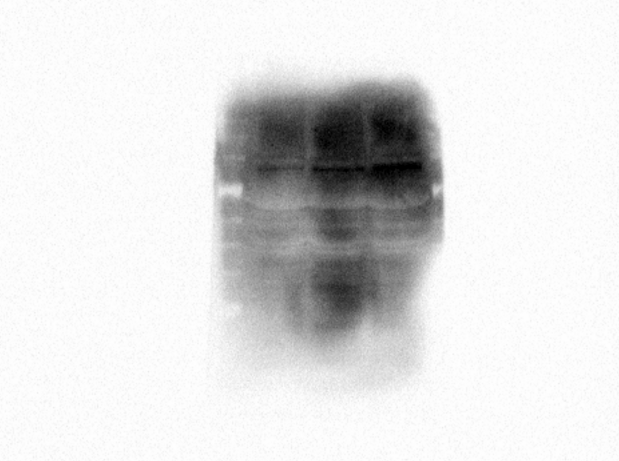 | 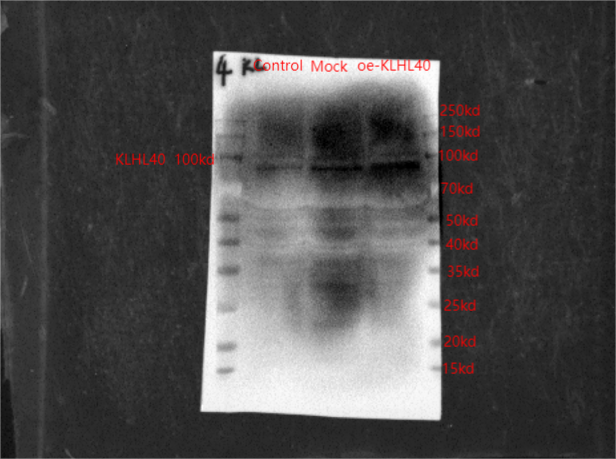 |
| KLHL40-3 | KLHL40-3+MARK |
| 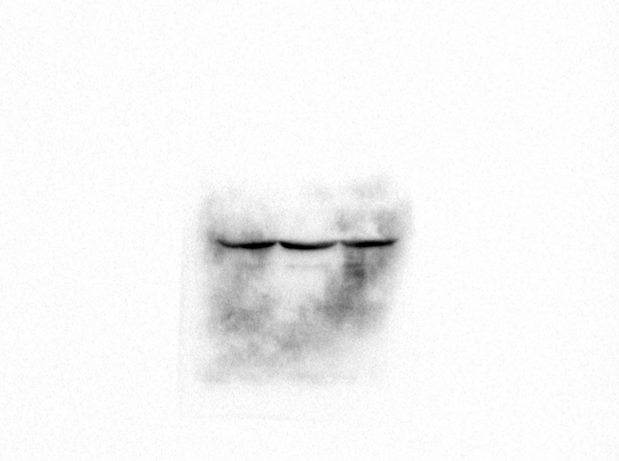 | 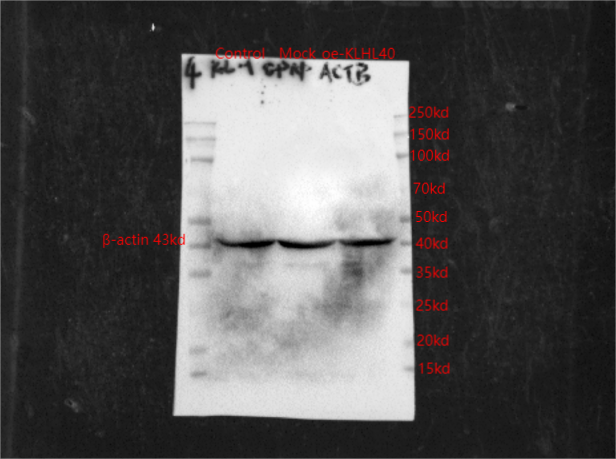 |
| KLHL40-3-ACTB | KLHL40-3-ACTB+MARK |
