# Supplementary material for: Post-infarction KLHL40-mediated regulation of cardiac sarcomeric integrity and function
Source: PeerJ. 2026 Jun 5;14:e21375. doi: 10.7717/peerj.21375 (PMC13245431; doi:10.7717/peerj.21375)
Supplement: Supplemental Information 23 [file peerj-14-21375-s023.zip › Figure 4 Labeled Western blot.docx]

# Figure. 4A Wb sh-KLHL40 MYOT

| 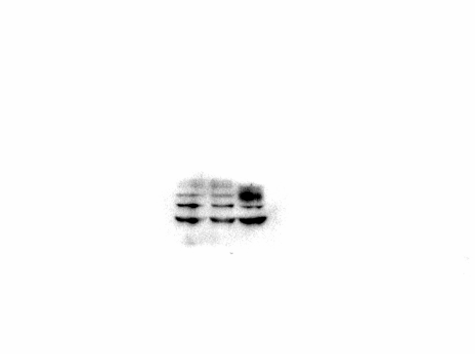 | 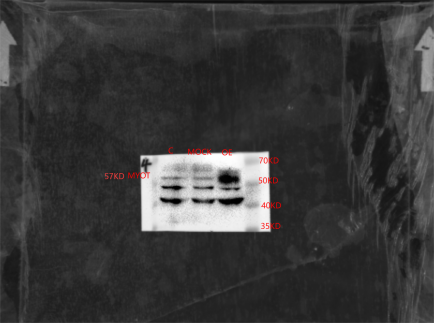 | 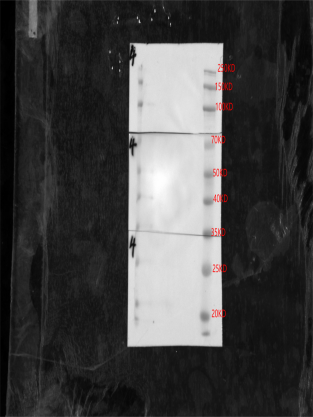 |
| --- | --- | --- |
| MYOT-1 sh-KLHL40 | MYOT-1 sh-KLHL40+MARK |  |
| 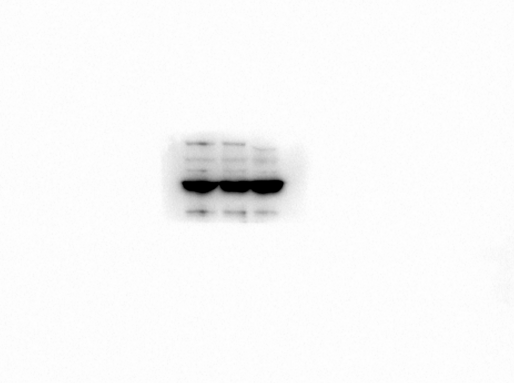 | 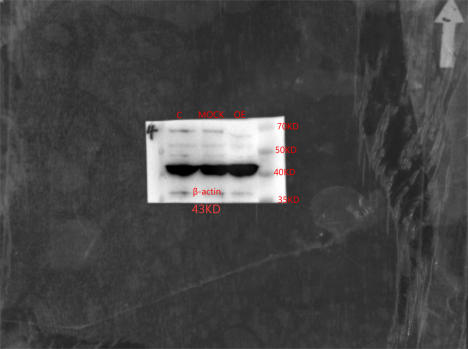 |  |
| MYOT-1 sh-KLHL40-ACTB | MYOT-1 sh-KLHL40-ACTB+MARK |  |
| 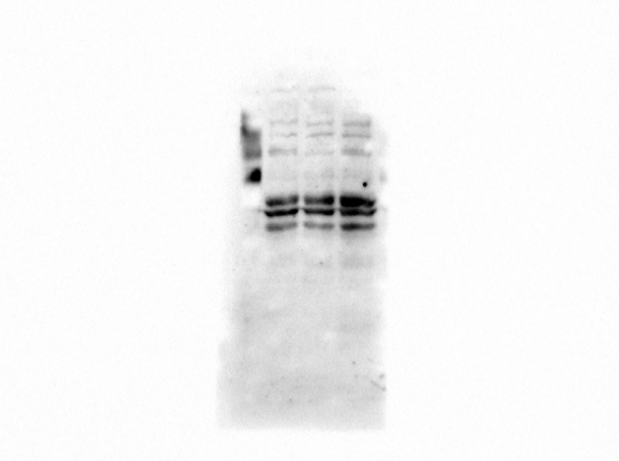 | 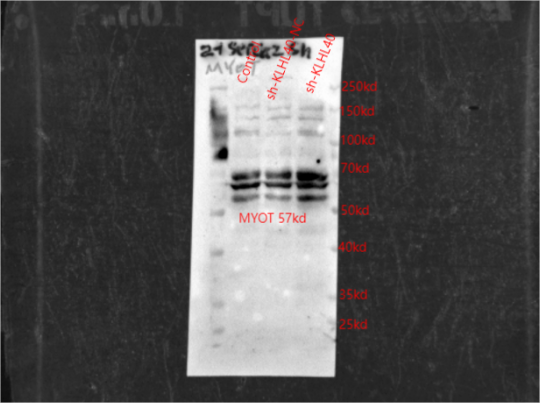 |  |
| MYOT-2 sh-KLHL40 | MYOT-2 sh-KLHL40+MARK |  |
| **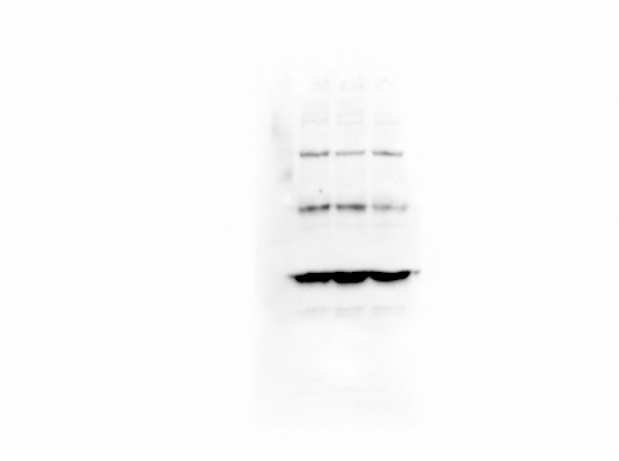** | **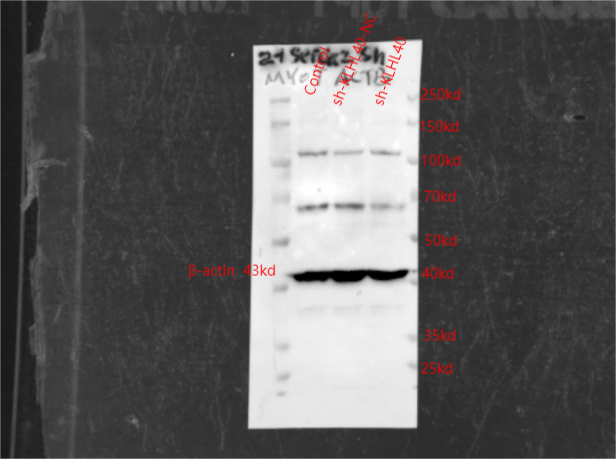** |  |
| MYOT-2 sh-KLHL40-ACTB | MYOT-2 sh-KLHL40-ACTB+MARK |  |
| 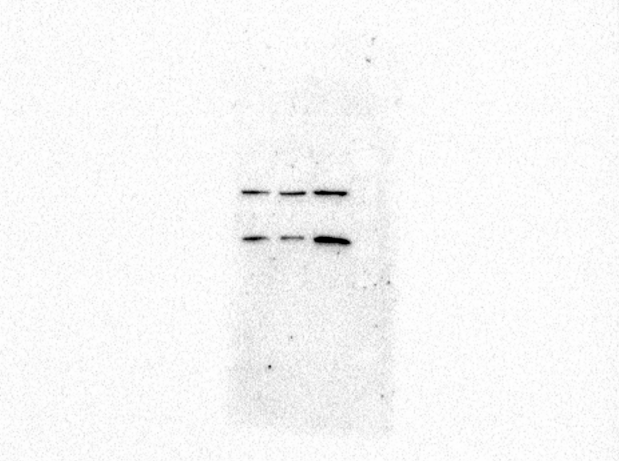 | 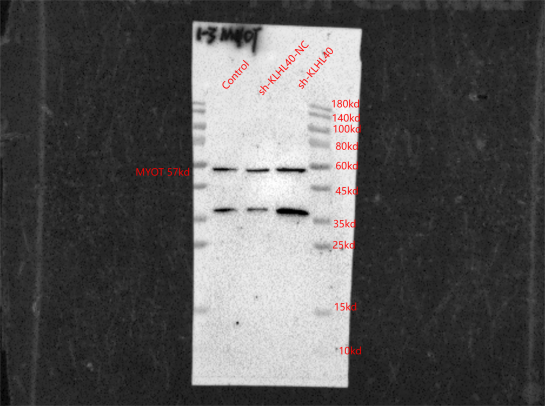 | 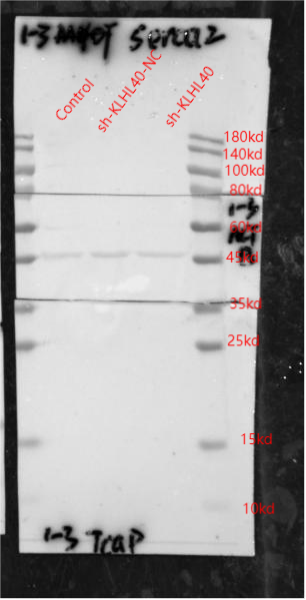 |
| MYOT-3 sh-KLHL40 | MYOT-3 sh-KLHL40+MARK |  |
| 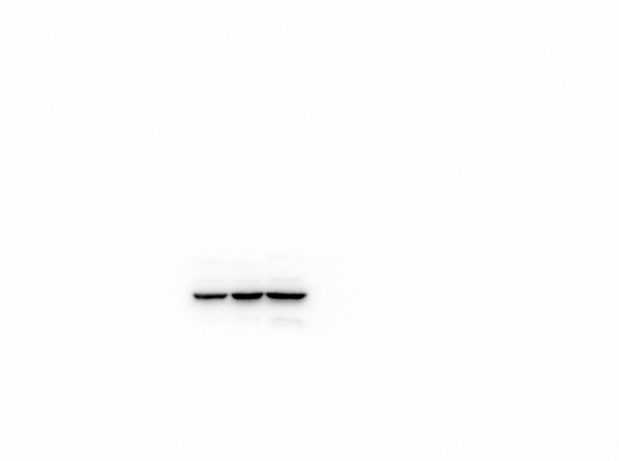 | 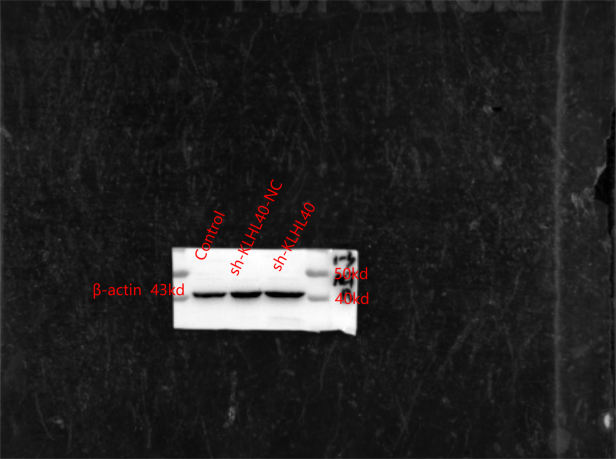 |  |
| MYOT-3 sh-KLHL40-ACTB | MYOT-3 sh-KLHL40-ACTB+MARK | TOTAL3 |

# Figure. 6B Wb oe-KLHL40 MYOT

| **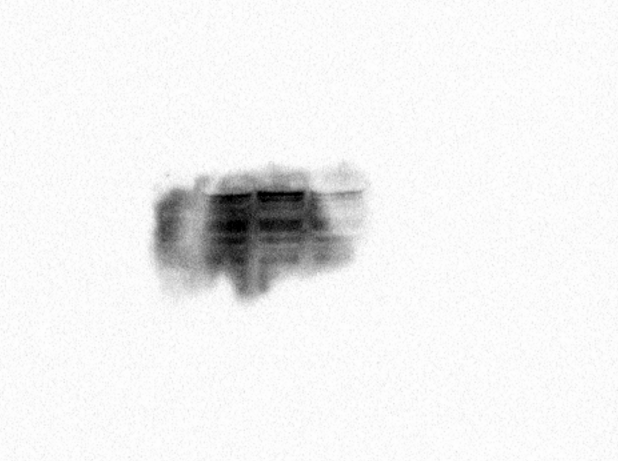** | **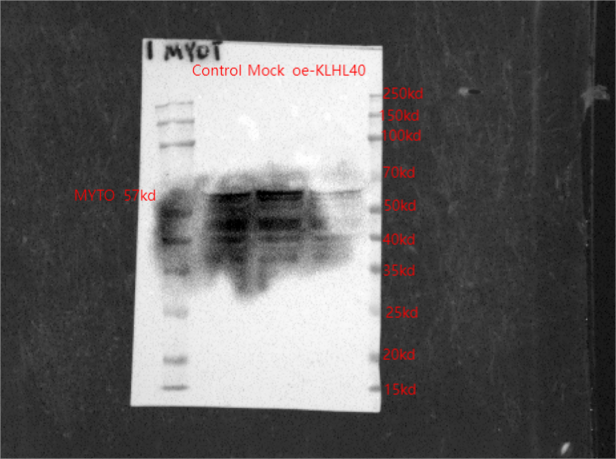** |
| --- | --- |
| MYOT-1 oe-KLHL40 | MYOT-1 oe-KLHL40+MARK |
| 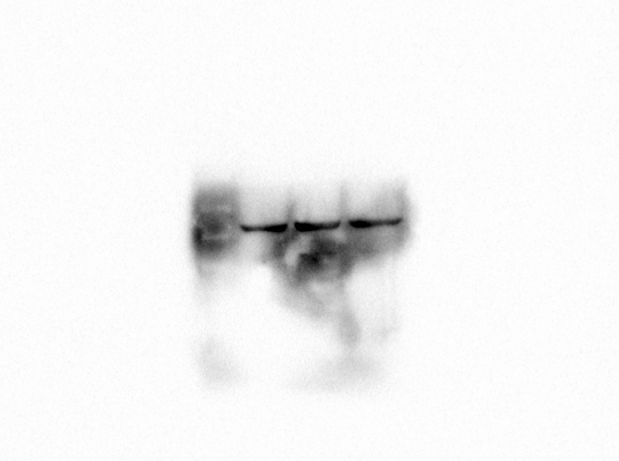 | 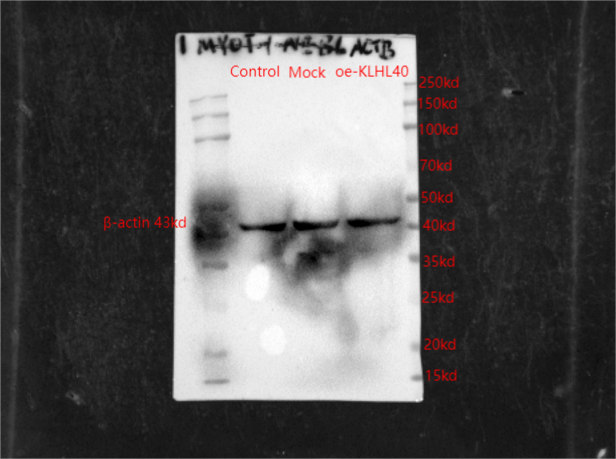 |
| MYOT-1 oe-KLHL40-ACTB | MYOT-1 oe-KLHL40-ACTB+MARK |
| 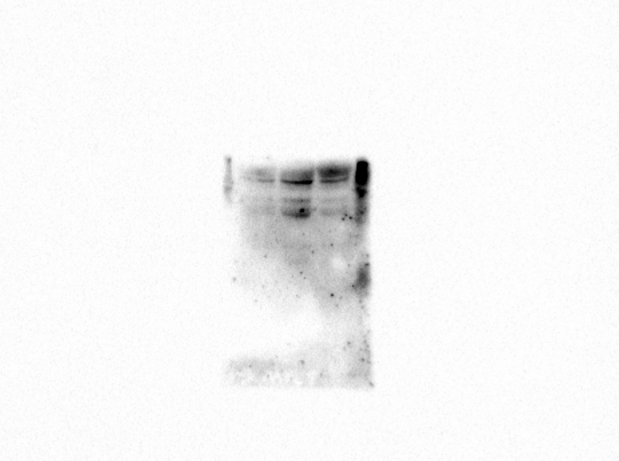 | 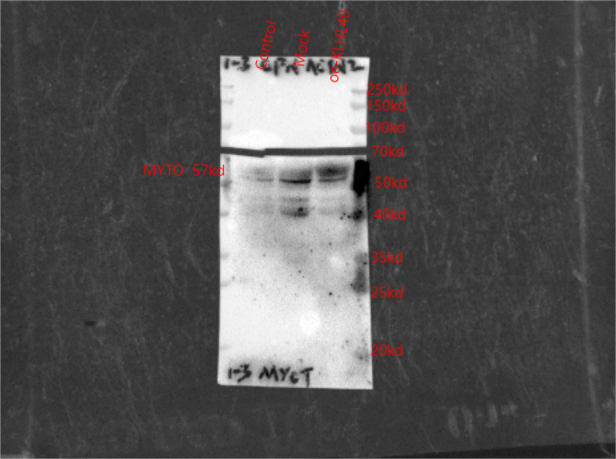 |
| MYOT-2 oe-KLHL40 | MYOT-2 oe-KLHL40+MARK |
| 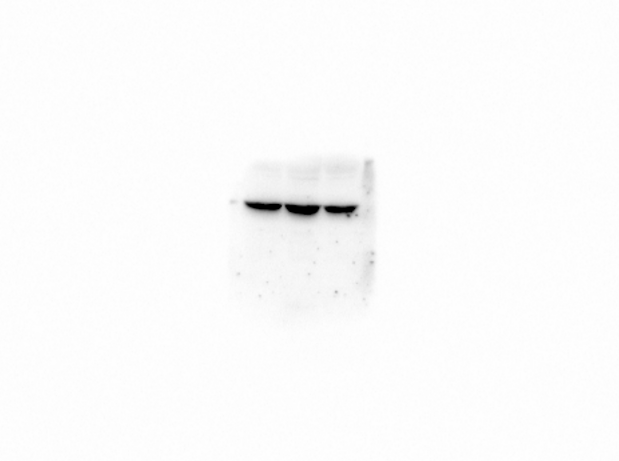 | 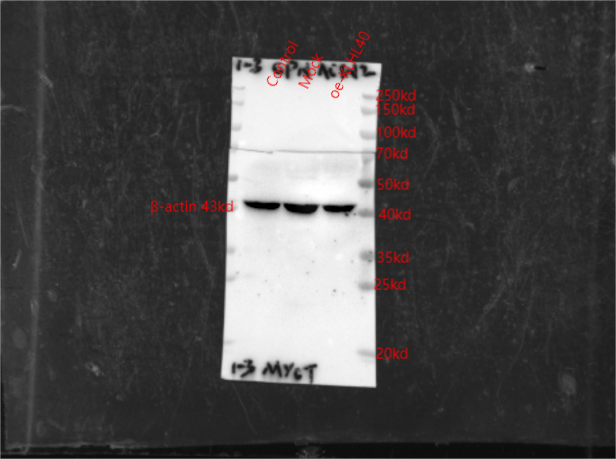 |
| MYOT-2 oe-KLHL40-ACTB | MYOT-2 oe-KLHL40-ACTB+MARK |
| 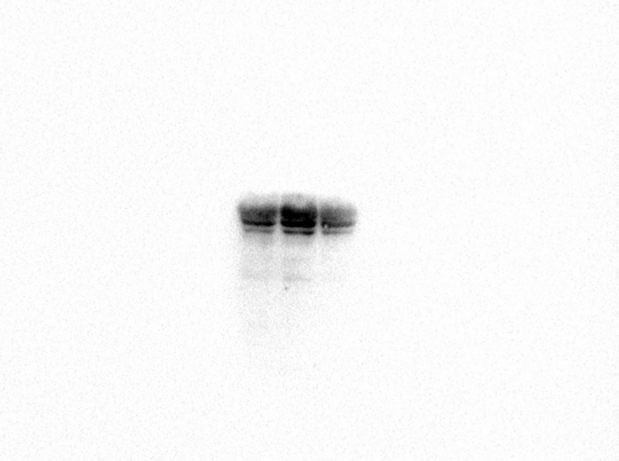 | 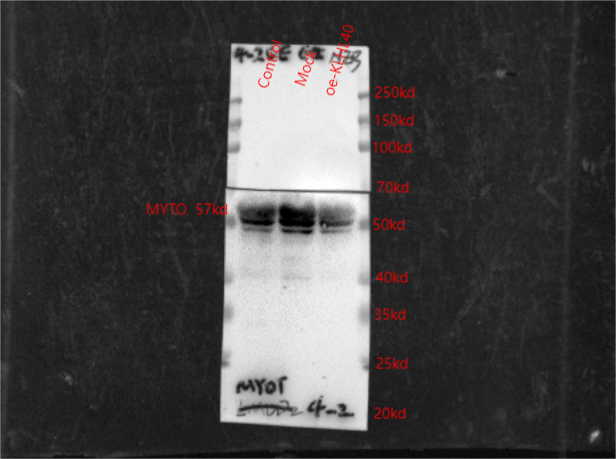 |
| MYOT-3 oe-KLHL40 | MYOT-3 oe-KLHL40+MARK |
| 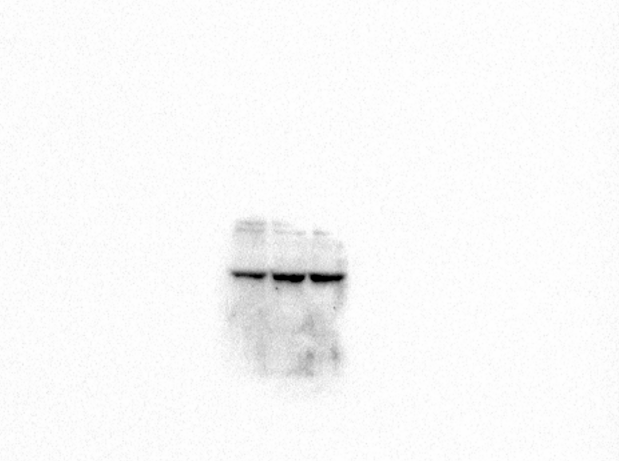 | 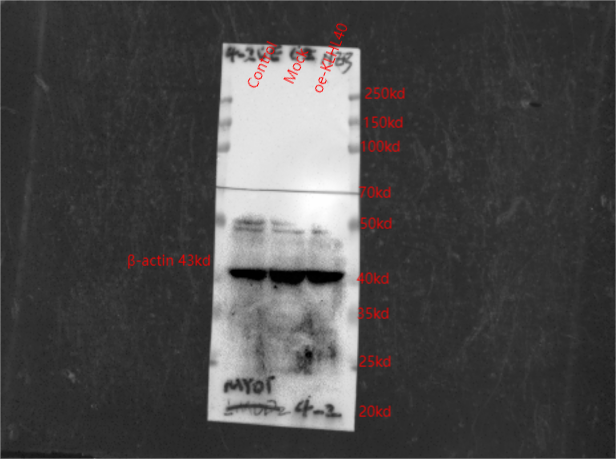 |
| MYOT-3 oe-KLHL40-ACTB | MYOT-3 oe-KLHL40-ACTB+MARK |

# Figure. 6C Wb sh-KLHL40 CAPZA

| **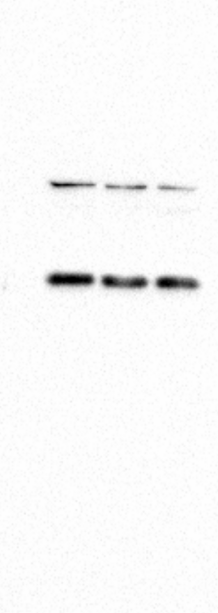** | 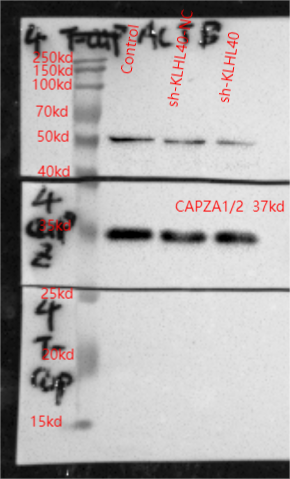 |  |
| --- | --- | --- |
| CAPZA-1 sh-KLHL40 | CAPZA-1 sh-KLHL40+MARK |  |
| 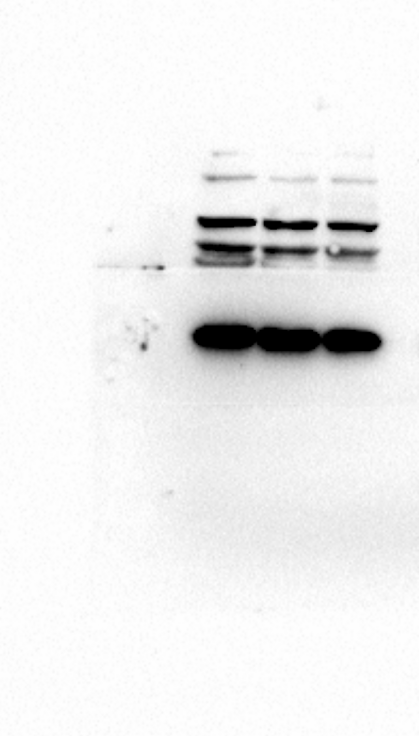 | 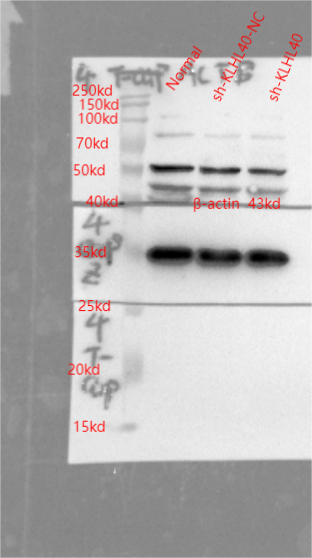 |  |
| CAPZA-1 sh-KLHL40+ACTB | CAPZA-1 sh-KLHL40+ACTB+MARK |  |
| 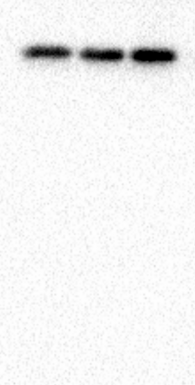 | 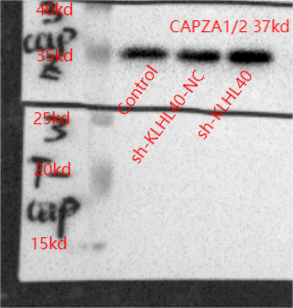 | 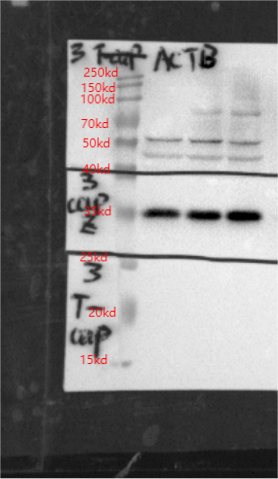 |
| CAPZA-2 sh-KLHL40 | CAPZA-2 sh-KLHL40+MARK |  |
| 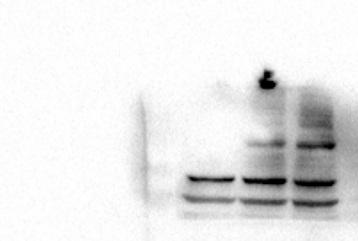 | 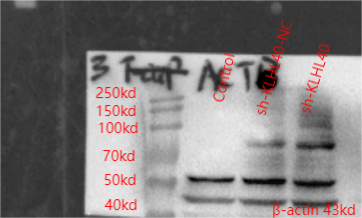 |  |
| CAPZA-2 sh-KLHL40-ACTB | CAPZA-2 sh-KLHL40-ACTB+MARK | TOTAL |
| 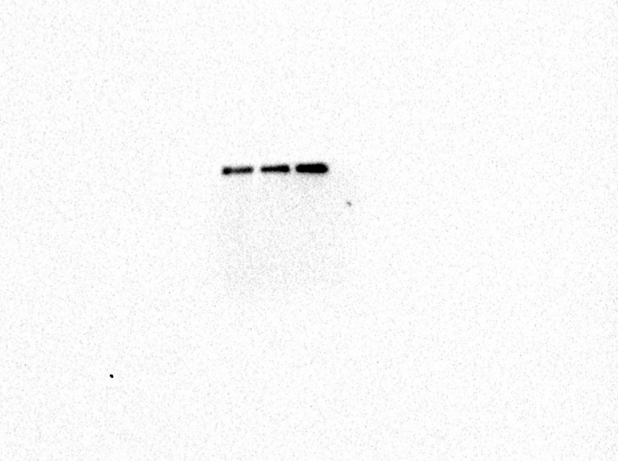 | 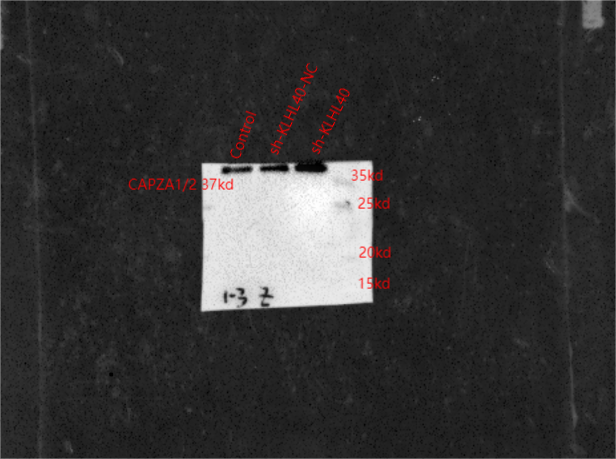 | 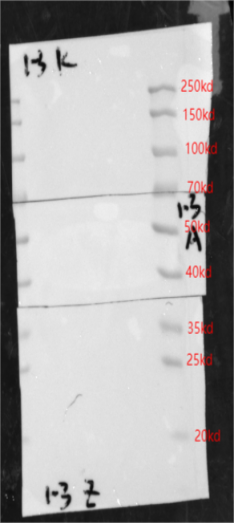 |
| CAPZA-3 sh-KLHL40 | CAPZA-3 sh-KLHL40-MARK |  |
| 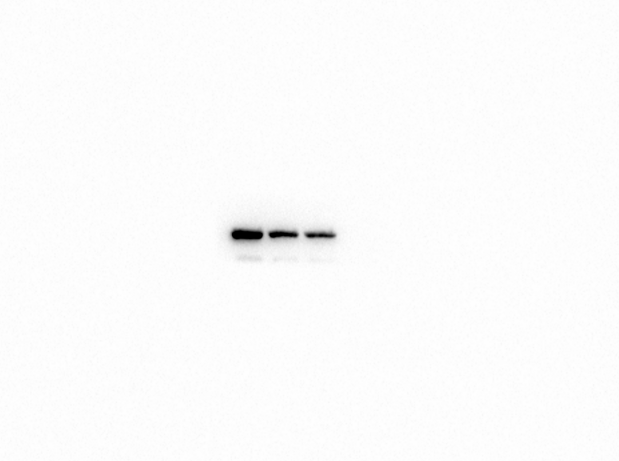 | 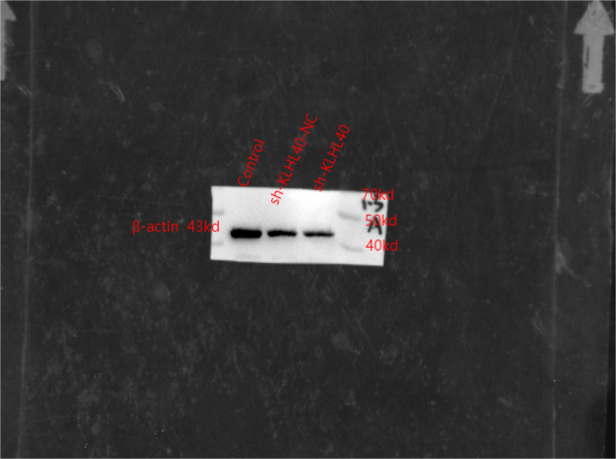 |  |
| CAPZA-3 sh-KLHL40-ACTB | CAPZA-3 sh-KLHL40-ACTB+MARK | TOTAL |

# Figure. 6D Wb oe-KLHL40 CAPZA

| **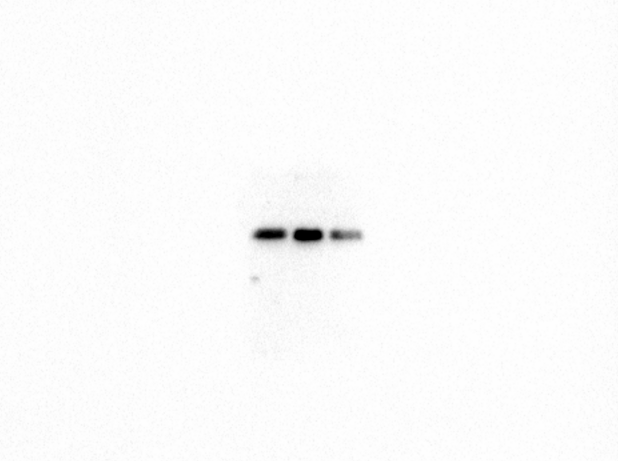** | **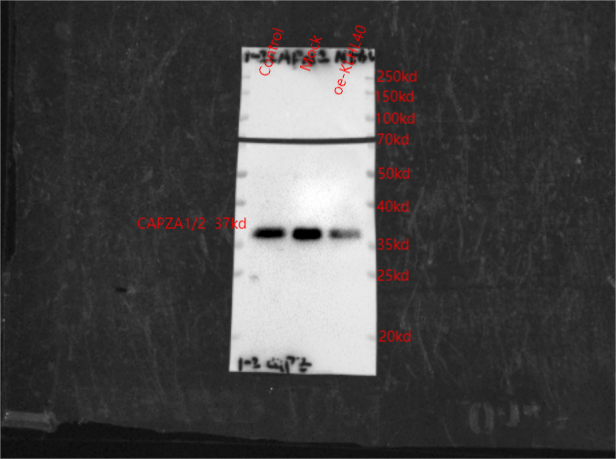** |  |
| --- | --- | --- |
| CAPZA-1 oe-KLHL40 | CAPZA-1 oe-KLHL40+MARK |  |
| 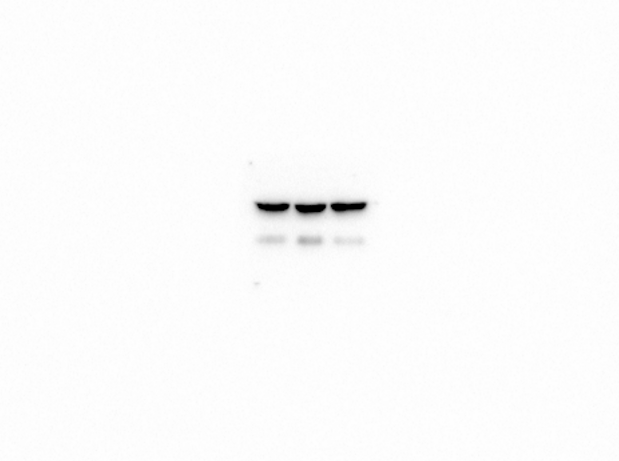 | 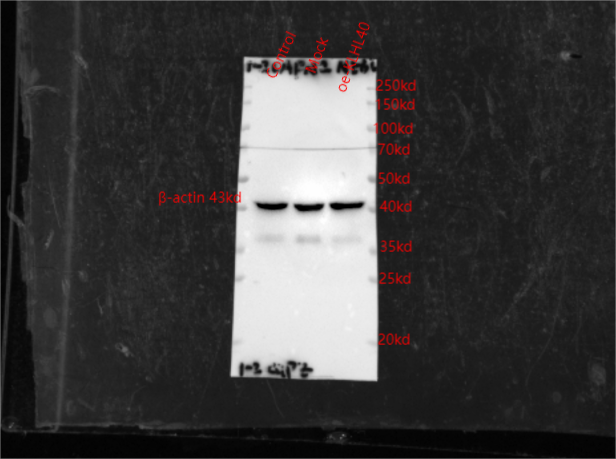 |  |
| CAPZA-1 oe-KLHL40-ACTB | CAPZA-1 oe-KLHL40-ACTB+MARK |  |
| 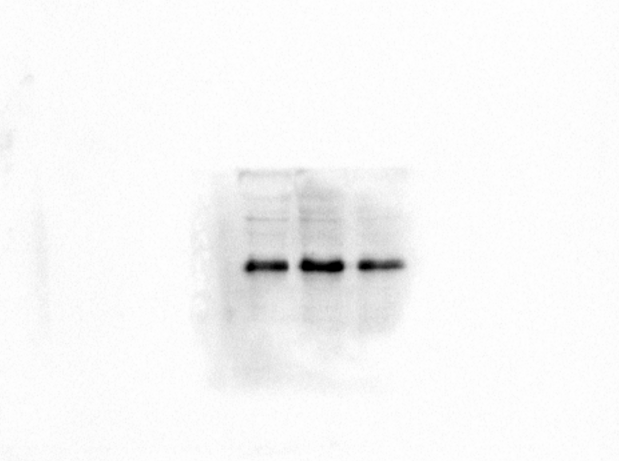 | 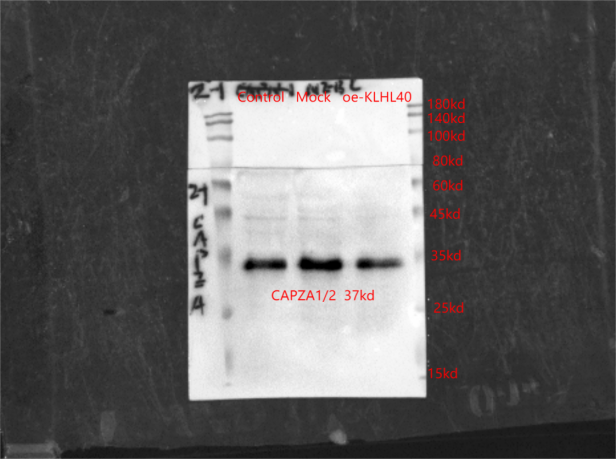 |  |
| CAPZA-2 oe-KLHL40 | CAPZA-2 oe-KLHL40+MARK |  |
| 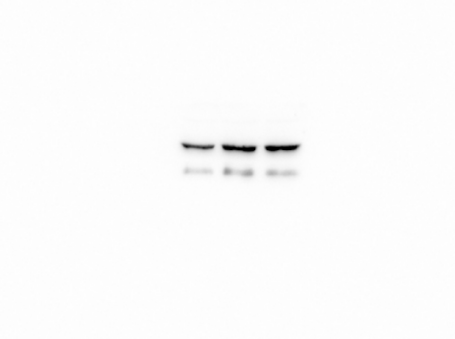 | 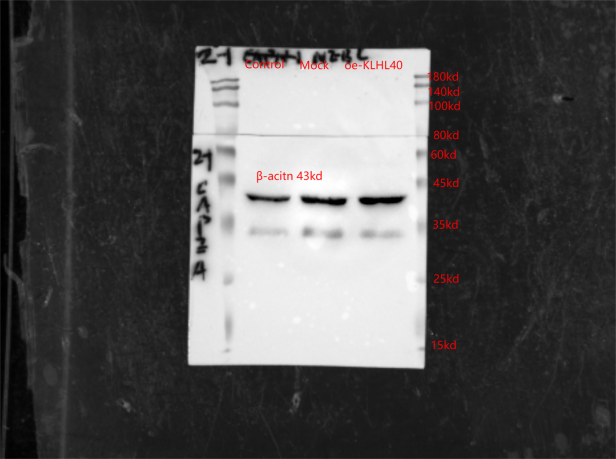 |  |
| CAPZA-2 oe-KLHL40-ACTB | CAPZA-2 oe-KLHL40-ACTB+MARK |  |
| 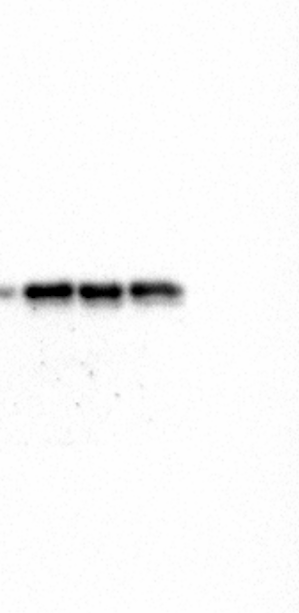 | 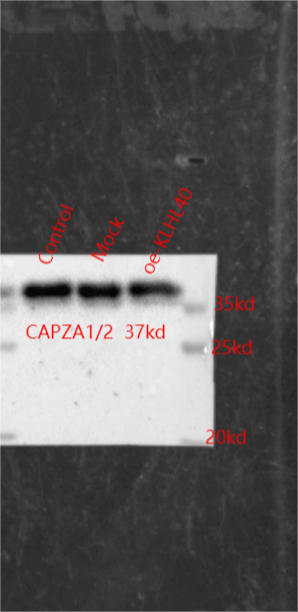 | 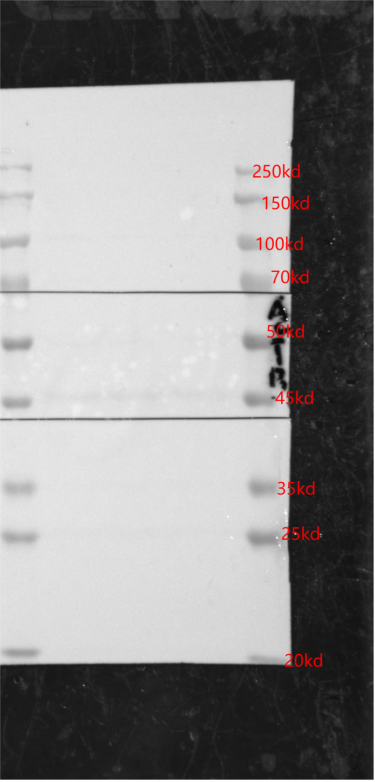 |
| CAPZA-3 oe-KLHL40 | CAPZA-3 oe-KLHL40+MARK |  |
| 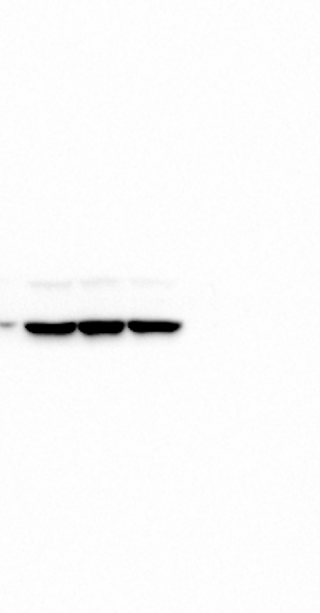 | 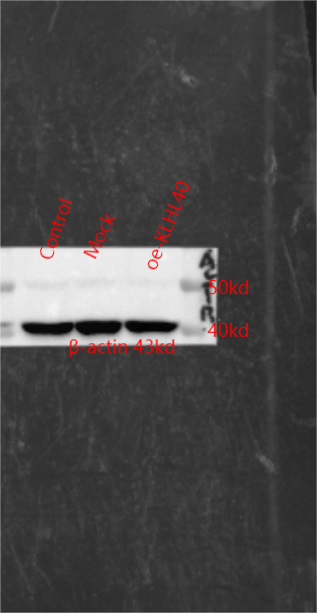 |  |
| CAPZA-3 oe-KLHL40-ACTB | CAPZA-3 oe-KLHL40-ACTB+MARK | TOTAL |

# Figure. 6E Wb sh-KLHL40 TCAP

| 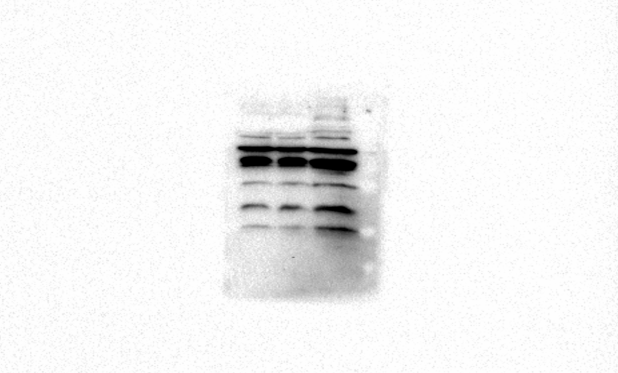 | 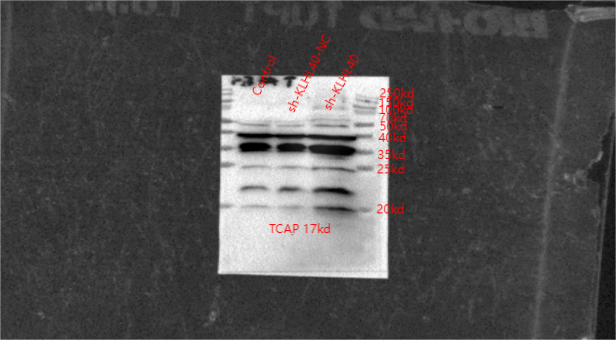 |  |
| --- | --- | --- |
| TCAP-1 sh-KLHL40 | TCAP-1 sh-KLHL40 MARK |  |
| 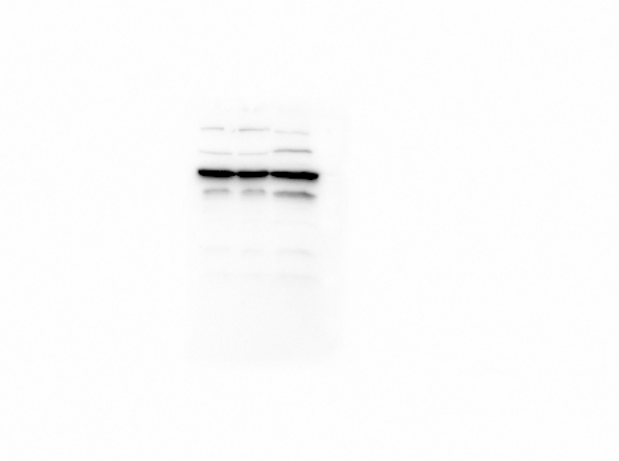 | 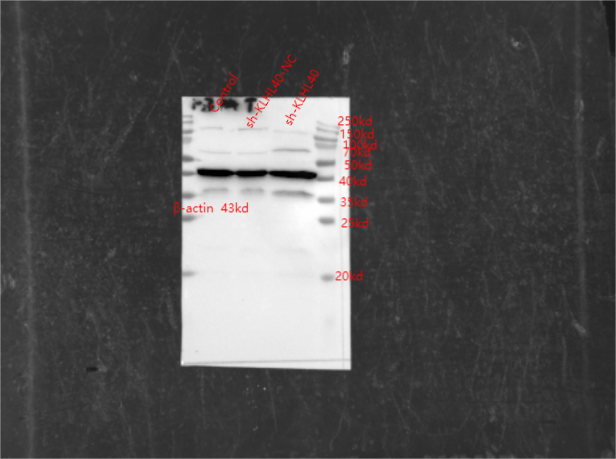 |  |
| TCAP-1 sh-KLHL40-ACTB | TCAP-1 sh-KLHL40-ACTB MARK |  |
| 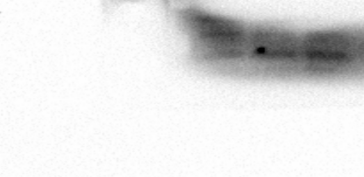 | 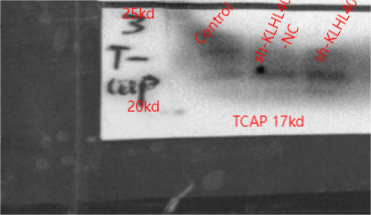 | 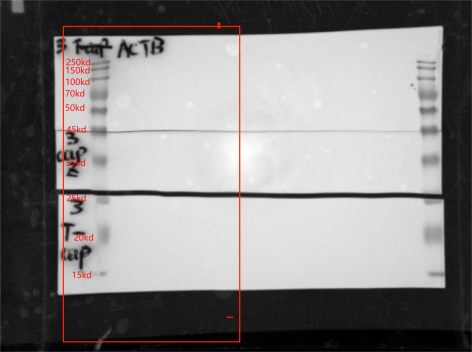 |
| TCAP-2 sh-KLHL40 | TCAP-2 sh-KLHL40 MARK |  |
| 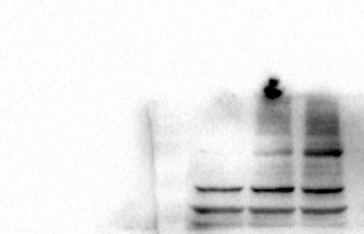 | 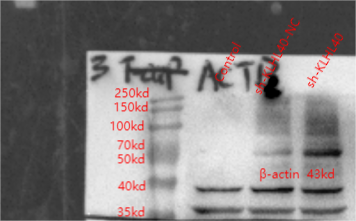 |  |
| TCAP-2 sh-KLHL40-ACTB | TCAP-2 sh-KLHL40-ACTB MARK | TOTAL |
| 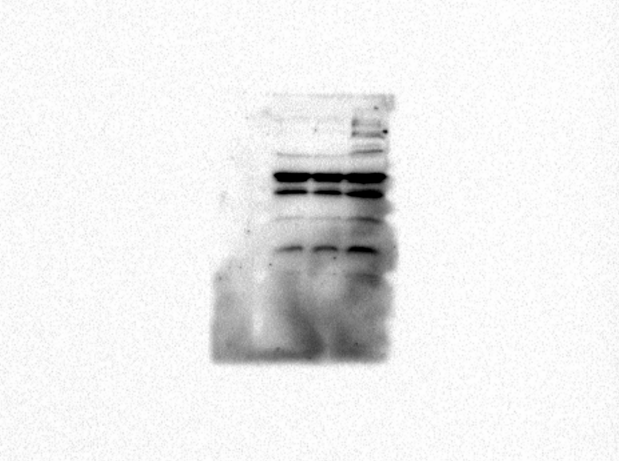 | 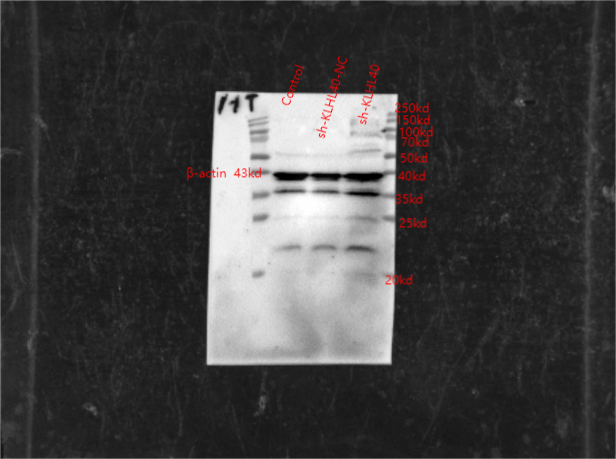 |  |
| TCAP-3 sh-KLHL40 | TCAP-3 sh-KLHL40 MARK |  |
| 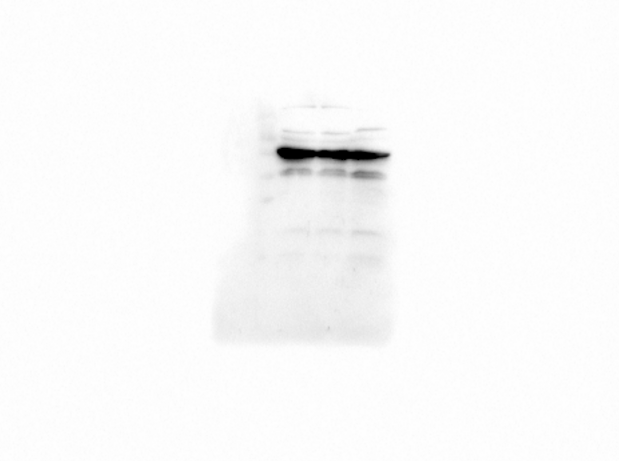 | 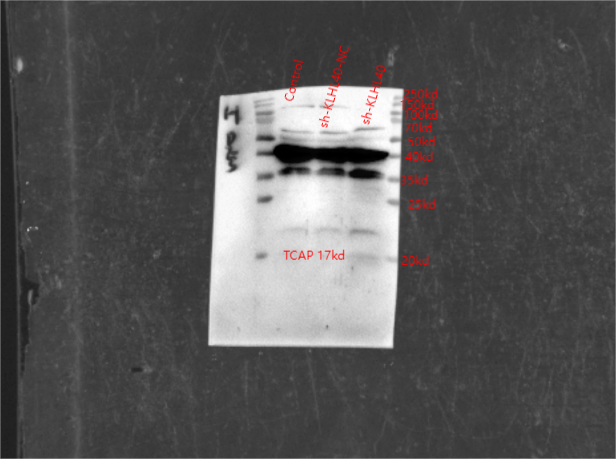 |  |
| TCAP-3 sh-KLHL40-ACTB | TCAP-3 sh-KLHL40-ACTB MARK |  |

# Figure. 6F Wb oe-KLHL40 TCAP

| **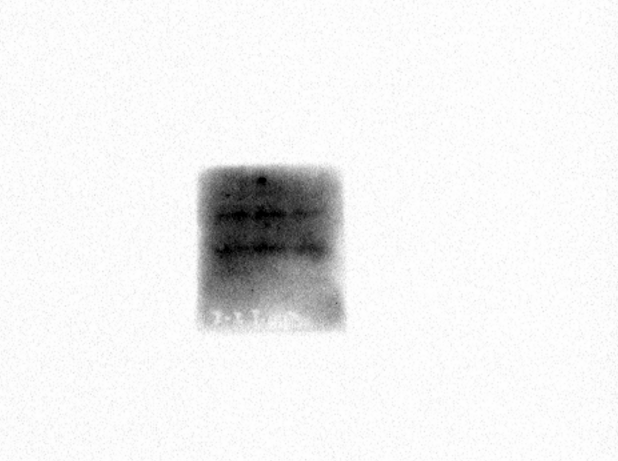** | **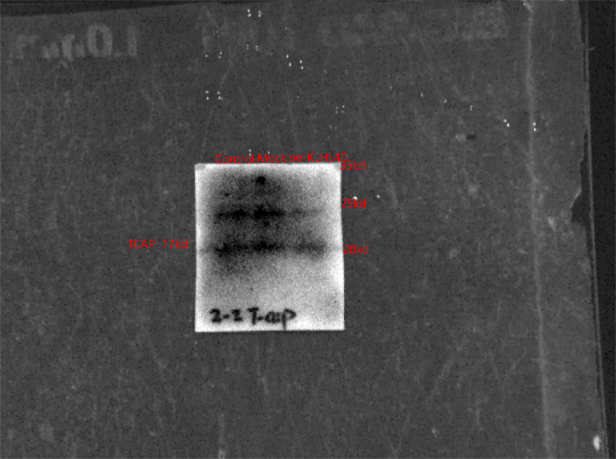** | **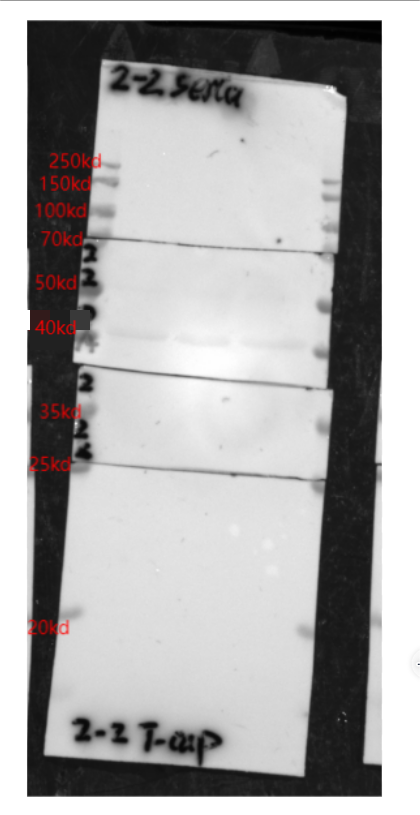** |
| --- | --- | --- |
| TCAP-1 oe-KLHL40 | TCAP-1 oe-KLHL40 MARK |  |
| 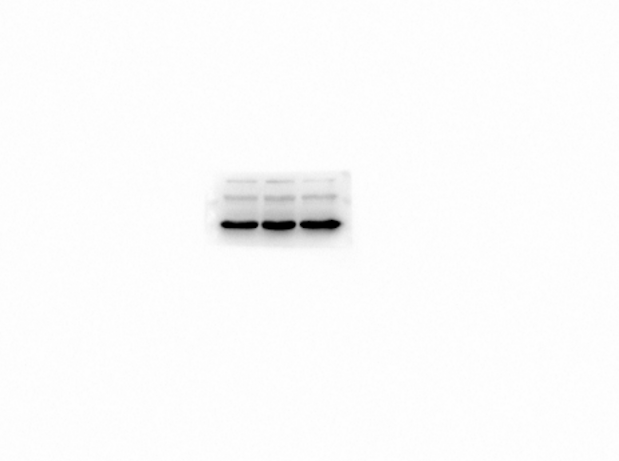 | 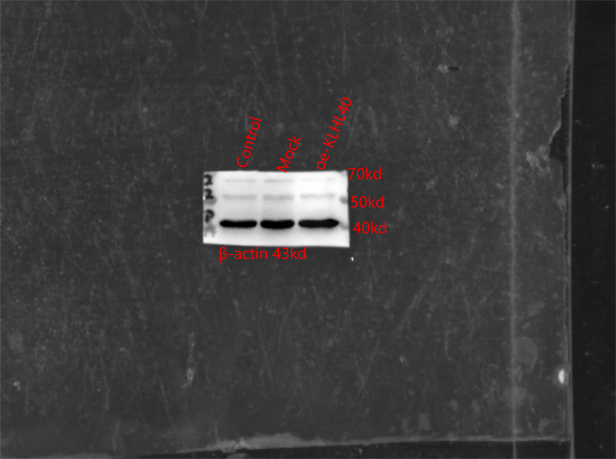 |  |
| TCAP-1 oe-KLHL40-ACTB | TCAP-1 oe-KLHL40-ACTB MARK | TOTAL |
| 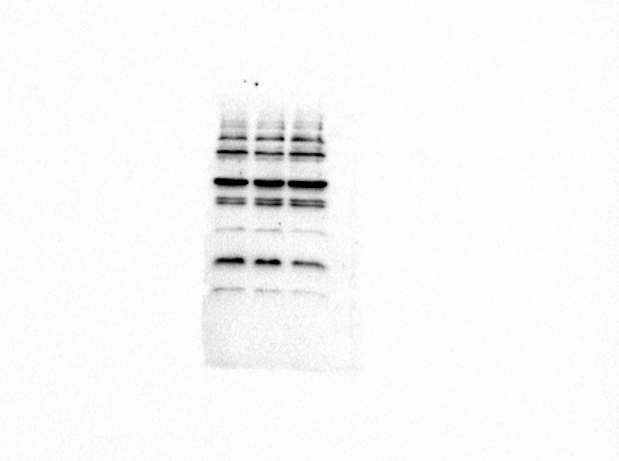 | 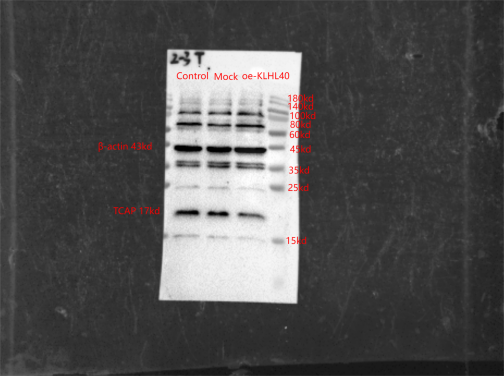 |  |
| TCAP-2 oe-KLHL40+ACTB | TCAP-2 oe-KLHL40+ACTB MARK |  |
| 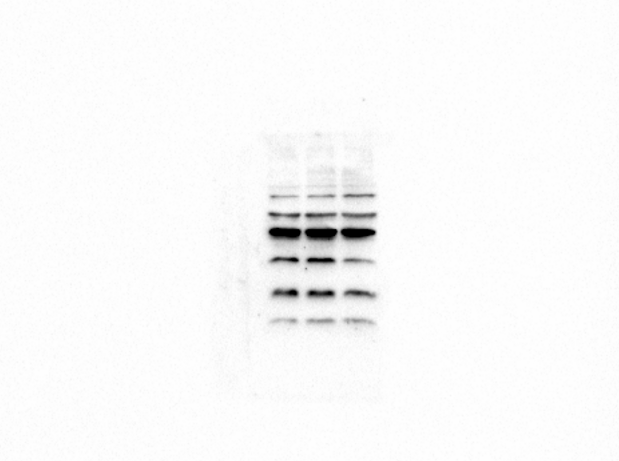 | 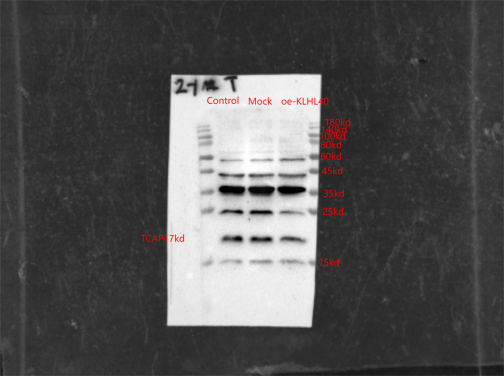 |  |
| TCAP-3 oe-KLHL40 | TCAP-3 oe-KLHL40 MARK |  |
| 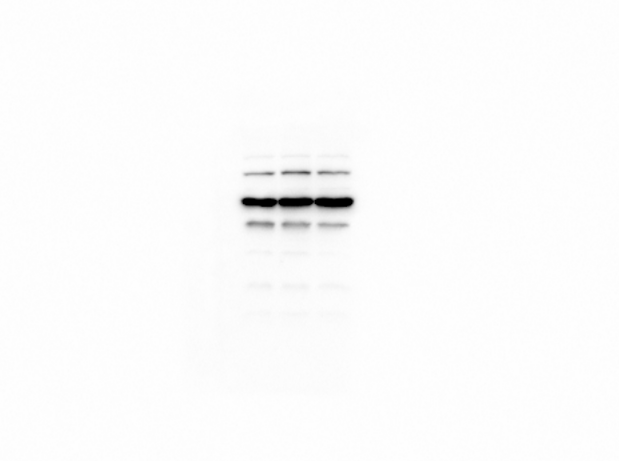 | 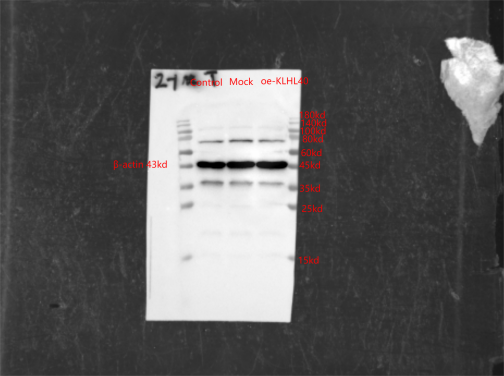 |  |
| TCAP-3 oe-KLHL40-ACTB | TCAP-3 oe-KLHL40-ACTB MARK |  |

# Figure. 6G Wb sh-KLHL40 ACTN2

| **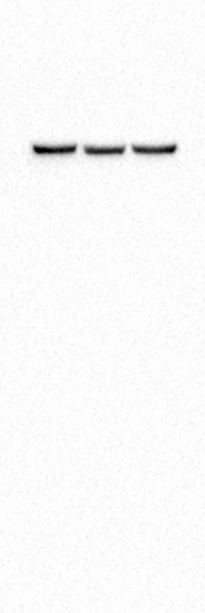** | **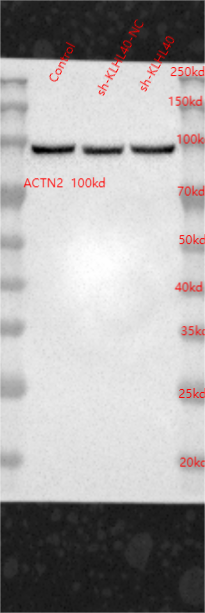** |
| --- | --- |
| ACTN2-1 sh-KLHL40 | ACTN2-1 sh-KLHL40 MARK |
| 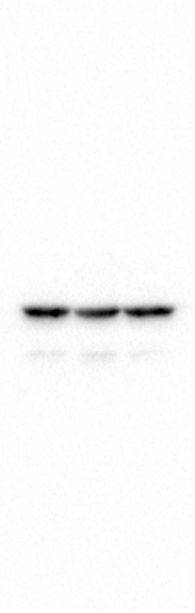 | 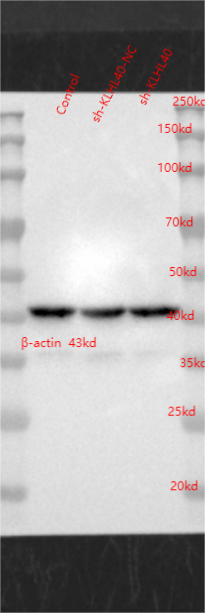 |
| ACTN2-1 sh-KLHL40-ACTB | ACTN2-1 sh-KLHL40-ACTB MARK |
| 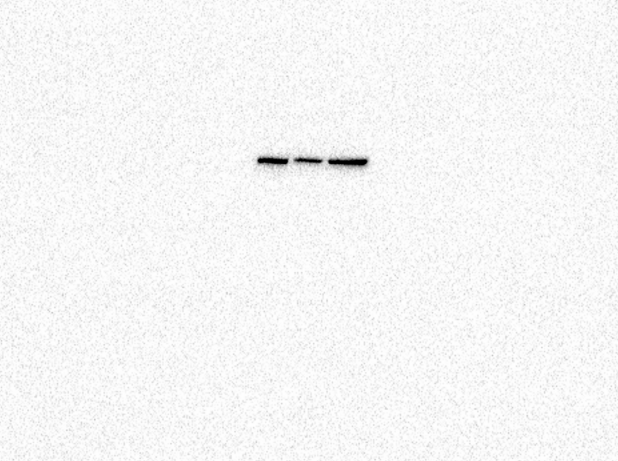 | 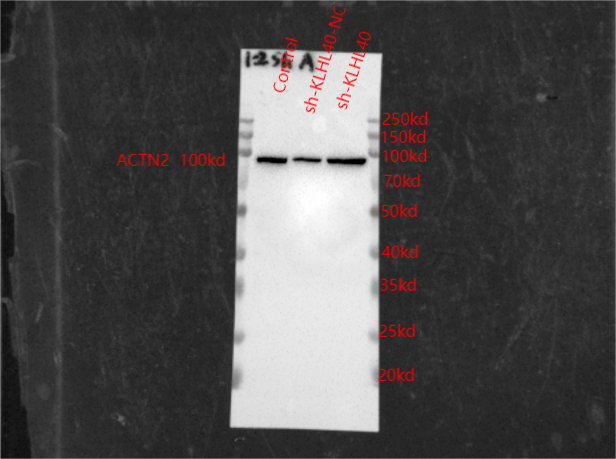 |
| ACTN2-2 sh-KLHL40 | ACTN2-2 sh-KLHL40 MARK |
| 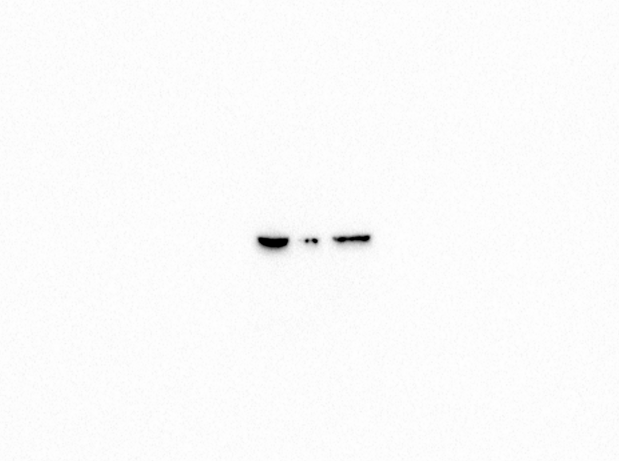 | 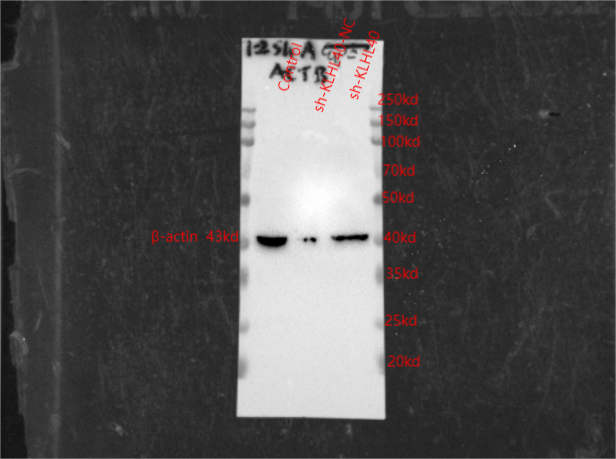 |
| ACTN2-2 sh-KLHL40-ACTB | ACTN2-2 sh-KLHL40-ACTB MARK |
| 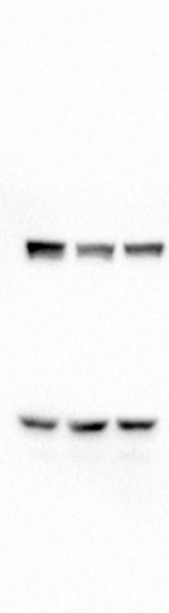 | 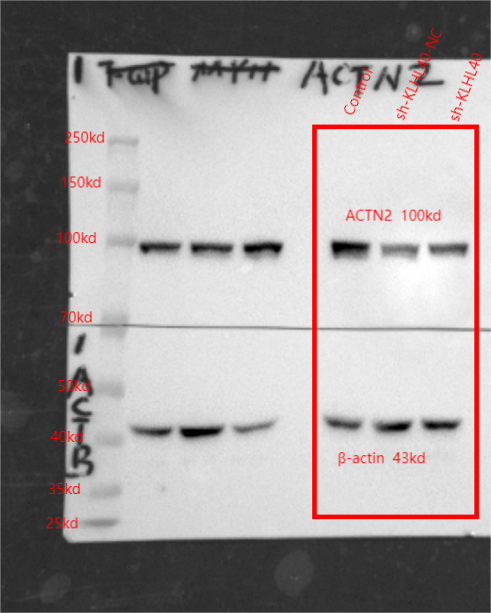 |
| ACTN2-3-sh-KLHL40+ACTB | ACTN2-3-sh-KLHL40+ACTB MARK |

# Figure. 6H Wb oe-KLHL40 ACTN2

| **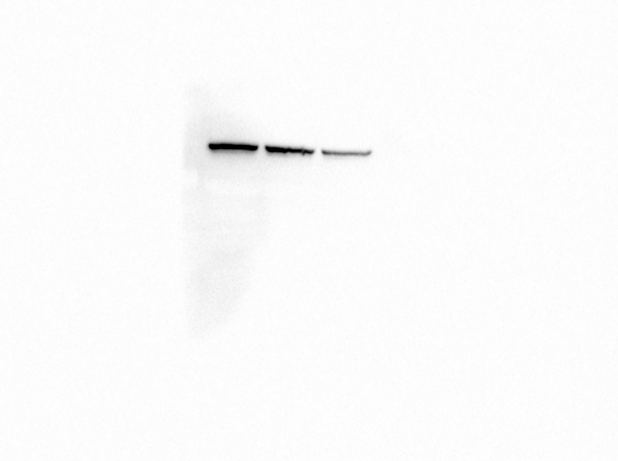** | **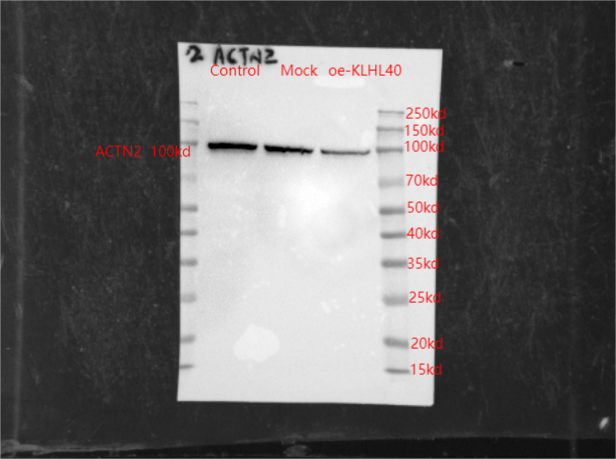** |
| --- | --- |
| ACTN2-1 oe-KLHL40 | ACTN2-1 oe-KLHL40 MARK |
| 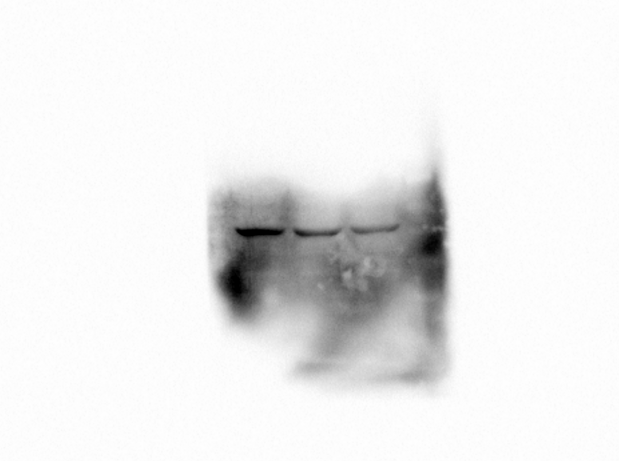 | 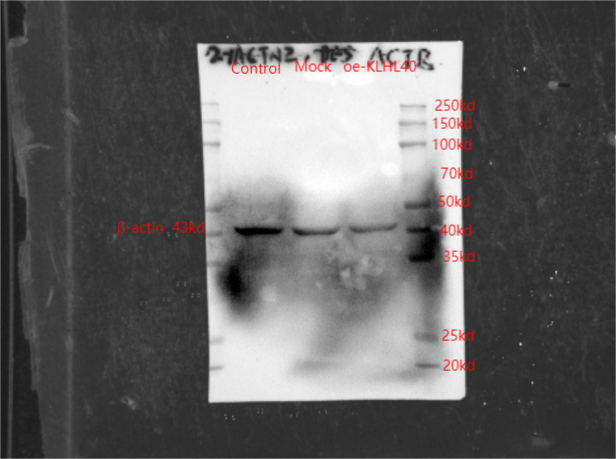 |
| ACTN2-1 oe-KLHL40-ACTB | ACTN2-1 oe-KLHL40-ACTB MARK |
| 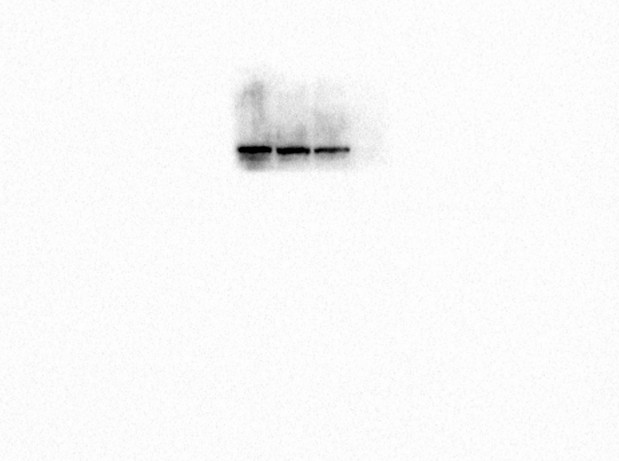 | 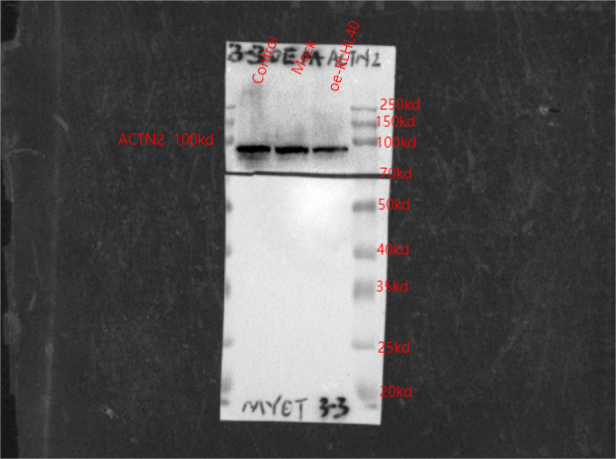 |
| ACTN2-2 oe-KLHL40 | ACTN2-2 oe-KLHL40 MARK |
| 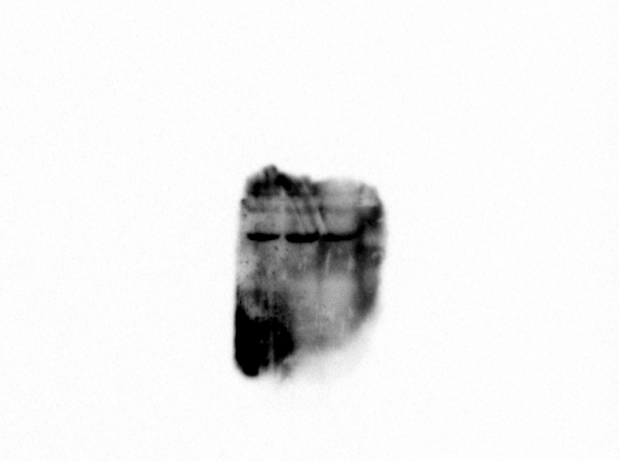 | 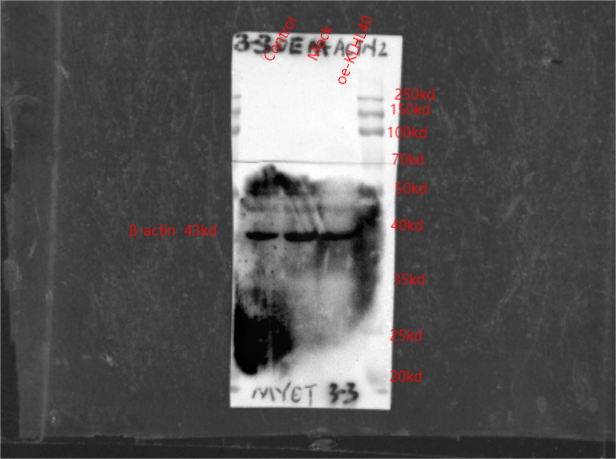 |
| ACTN2-2 oe-KLHL40-ACTB | ACTN2-2 oe-KLHL40-ACTB MARK |
| 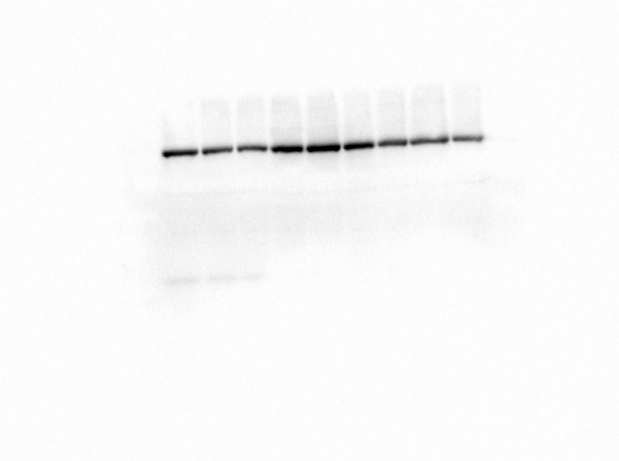 | 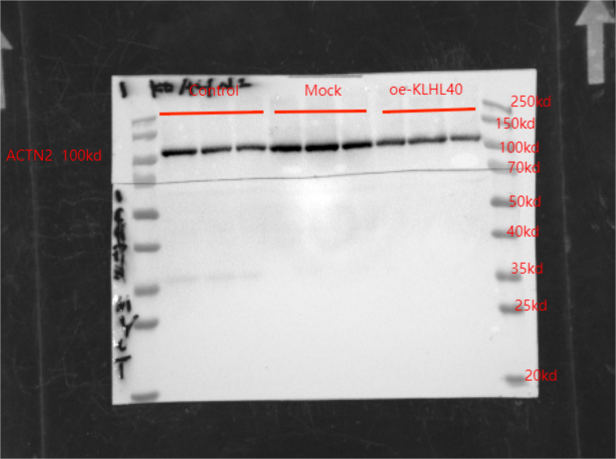 |
| ACTN2-3 oe-KLHL40 | ACTN2-3 oe-KLHL40 MARK |
| 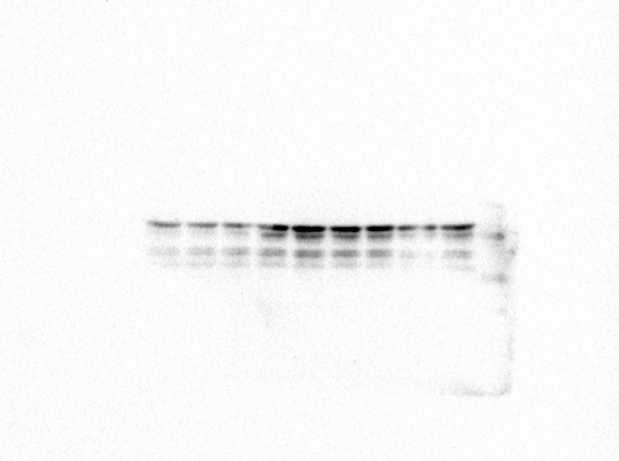 | 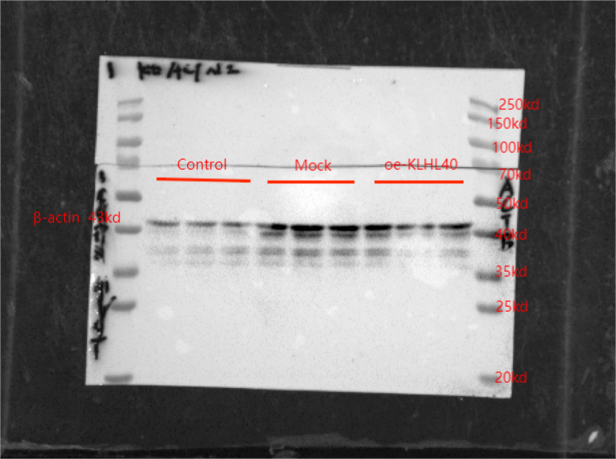 |
| ACTN2-3 oe-KLHL40-ACTB | ACTN2-3 oe-KLHL40-ACTB MARK |

# Figure. 6I Wb sh-KLHL40 FLNC

| **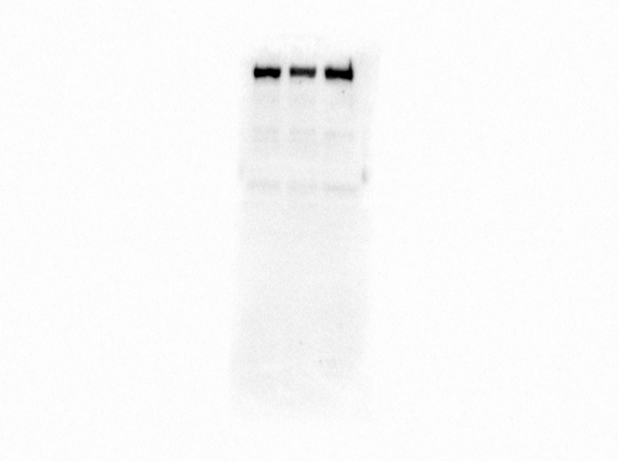** |  |  |
| --- | --- | --- |
| FLNC-1 sh-KLHL40 | FLNC-1 sh-KLHL40 MARK |  |
|  |  |  |
| FLNC-1 sh-KLHL40-ACTB | FLNC-1 sh-KLHL40-ACTB MARK |  |
|  |  |  |
| FLNC-2 sh-KLHL40 | FLNC-2 sh-KLHL40 MARK |  |
|  |  |  |
| FLNC-2 sh-KLHL40-ACTB | FLNC-2 sh-KLHL40-ACTB MARK | TOTAL |
|  |  |  |
| FLNC-3 sh-KLHL40 | FLNC-3 sh-KLHL40 MARK |  |
|  |  |  |
| FLNC-3 sh-KLHL40-ACTB | FLNC-3 sh-KLHL40-ACTB MARK | TOTAL |

# Figure. 6J Wb oe-KLHL40 FLNC

|  |  |  |
| --- | --- | --- |
| FLNC-1 oe-KLHL40 | FLNC-1 oe-KLHL40 MARK |  |
|  |  |  |
| FLNC-1 oe-KLHL40-ACTB | FLNC-1 oe-KLHL40-ACTB MARK |  |
|  |  |  |
| FLNC-2 oe-KLHL40 | FLNC-2 oe-KLHL40 MARK |  |
|  |  |  |
| FLNC-2 oe-KLHL40-ACTB | FLNC-2 oe-KLHL40-ACTB MARK | TOTAL |
|  |  |  |
| FLNC-3 oe-KLHL40 | FLNC-3 oe-KLHL40 MARK |  |
|  |  |  |
| FLNC-3 oe-KLHL40-ACTB | FLNC-3 oe-KLHL40-ACTB MARK | TOTAL |
