# Supplementary figures and images for: Post-infarction KLHL40-mediated regulation of cardiac sarcomeric integrity and function (part 2 of 5)
Source: PeerJ. 2026 Jun 5;14:e21375. doi: 10.7717/peerj.21375 (PMC13245431; doi:10.7717/peerj.21375)

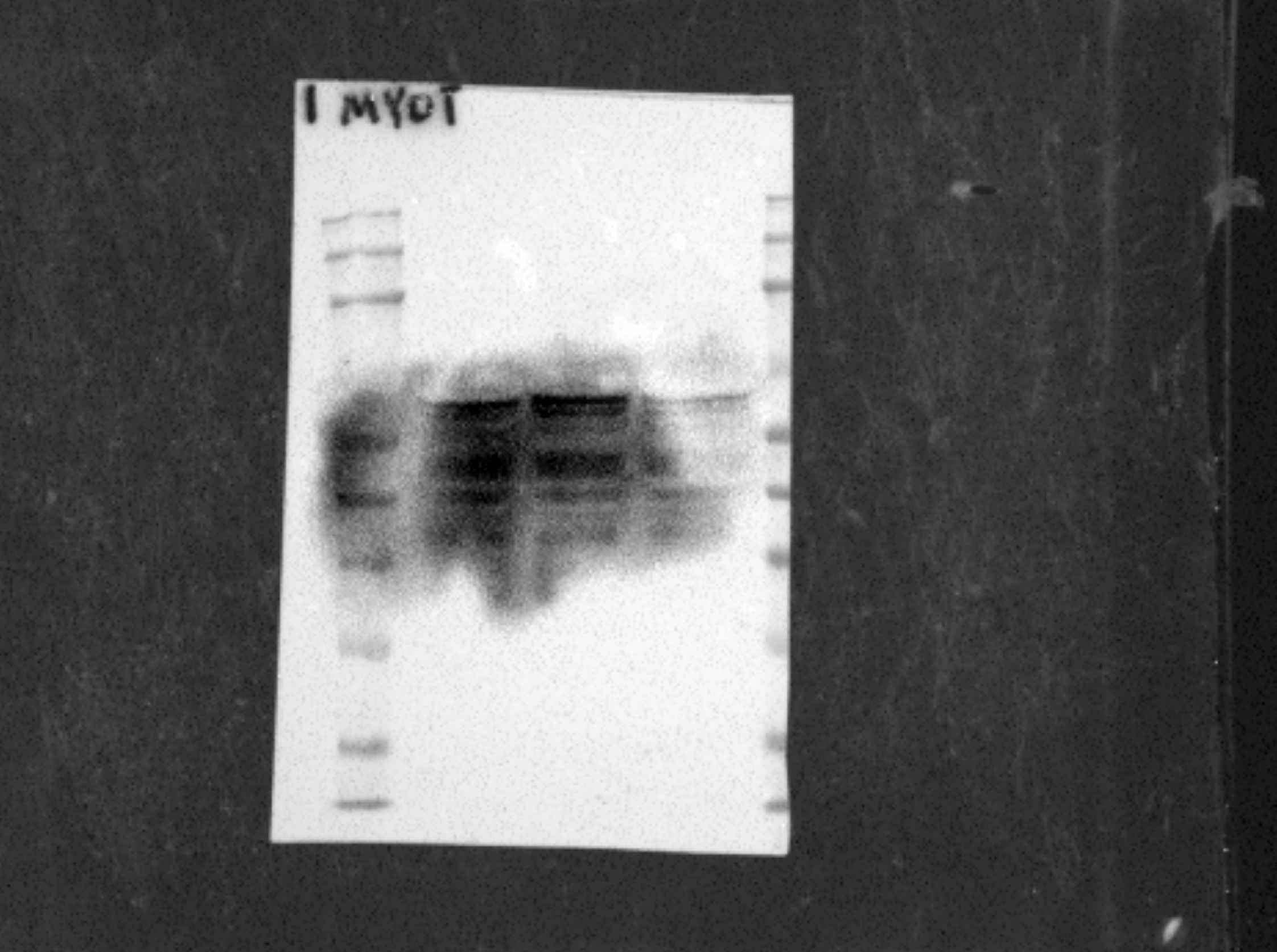

Supplement: Supplemental Information 25 [file peerj-14-21375-s025.zip › Figure 4B WB RAW oe-KLHL40 MYOT/MYOT-1 oe-KLHL40+MARK.tif]

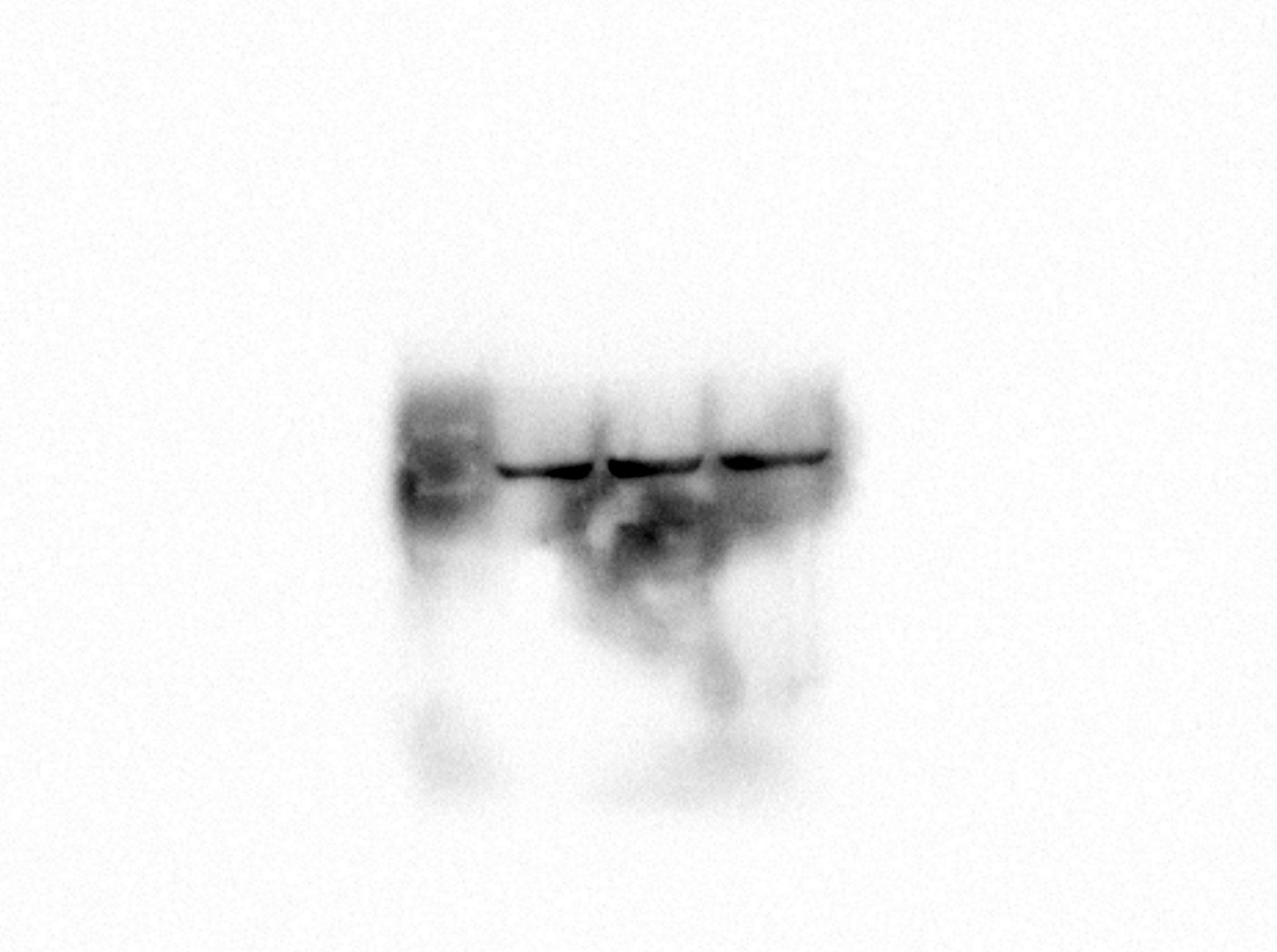

Supplement: Supplemental Information 25 [file peerj-14-21375-s025.zip › Figure 4B WB RAW oe-KLHL40 MYOT/MYOT-1 oe-KLHL40-ACTB.tif]

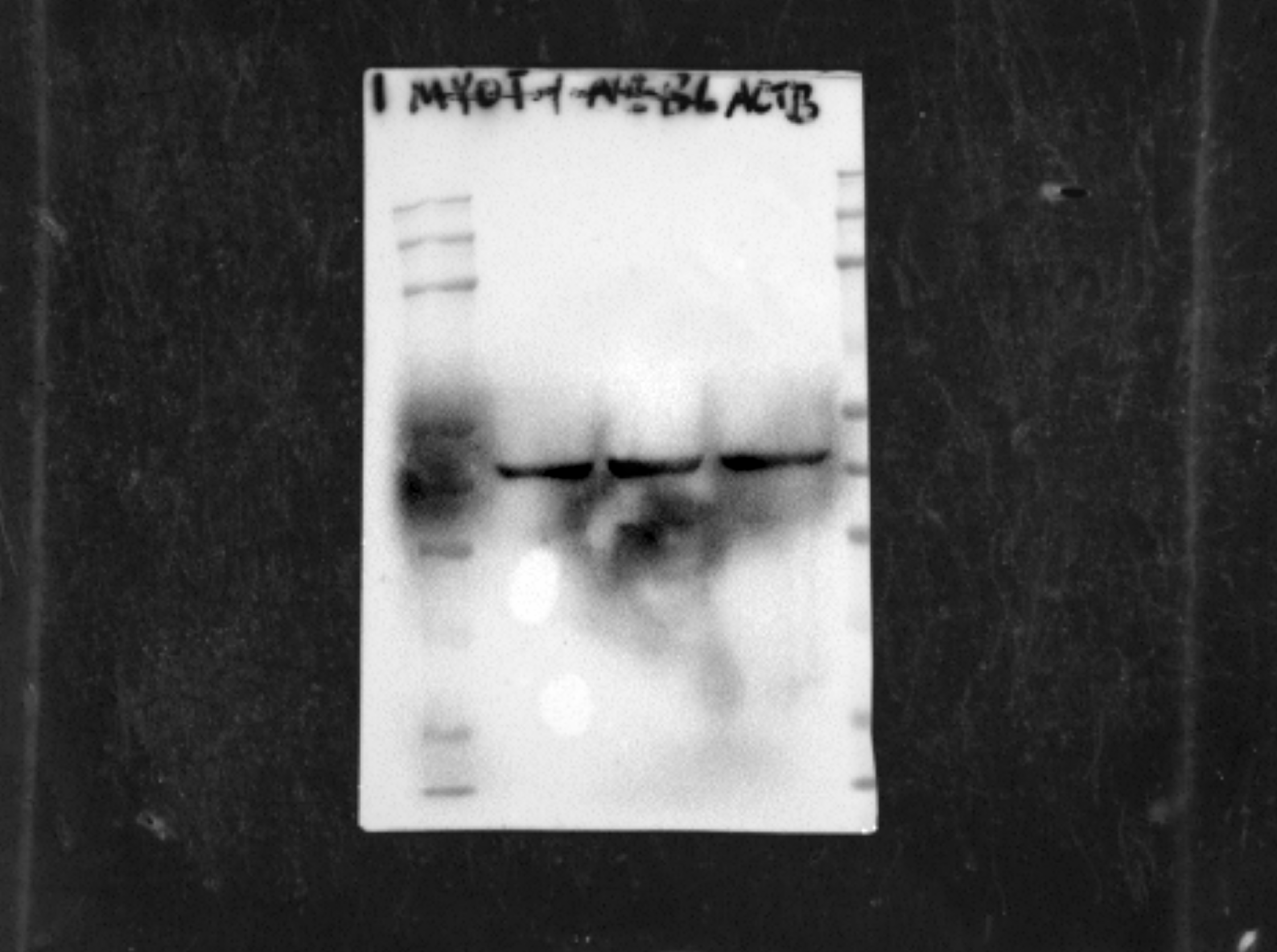

Supplement: Supplemental Information 25 [file peerj-14-21375-s025.zip › Figure 4B WB RAW oe-KLHL40 MYOT/MYOT-1 oe-KLHL40-ACTB+MARK.tif]

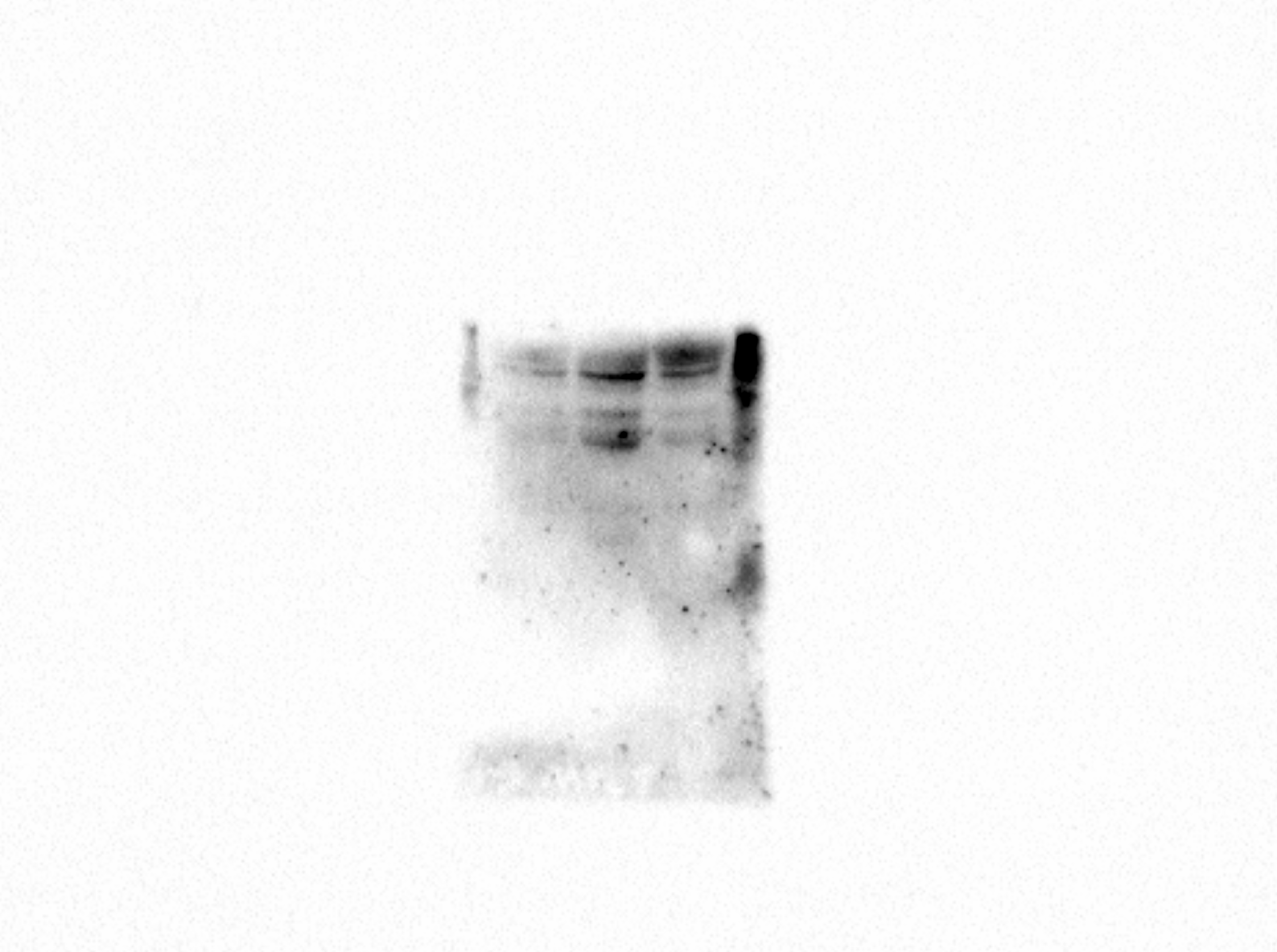

Supplement: Supplemental Information 25 [file peerj-14-21375-s025.zip › Figure 4B WB RAW oe-KLHL40 MYOT/MYOT-2 oe-KLHL40.tif]

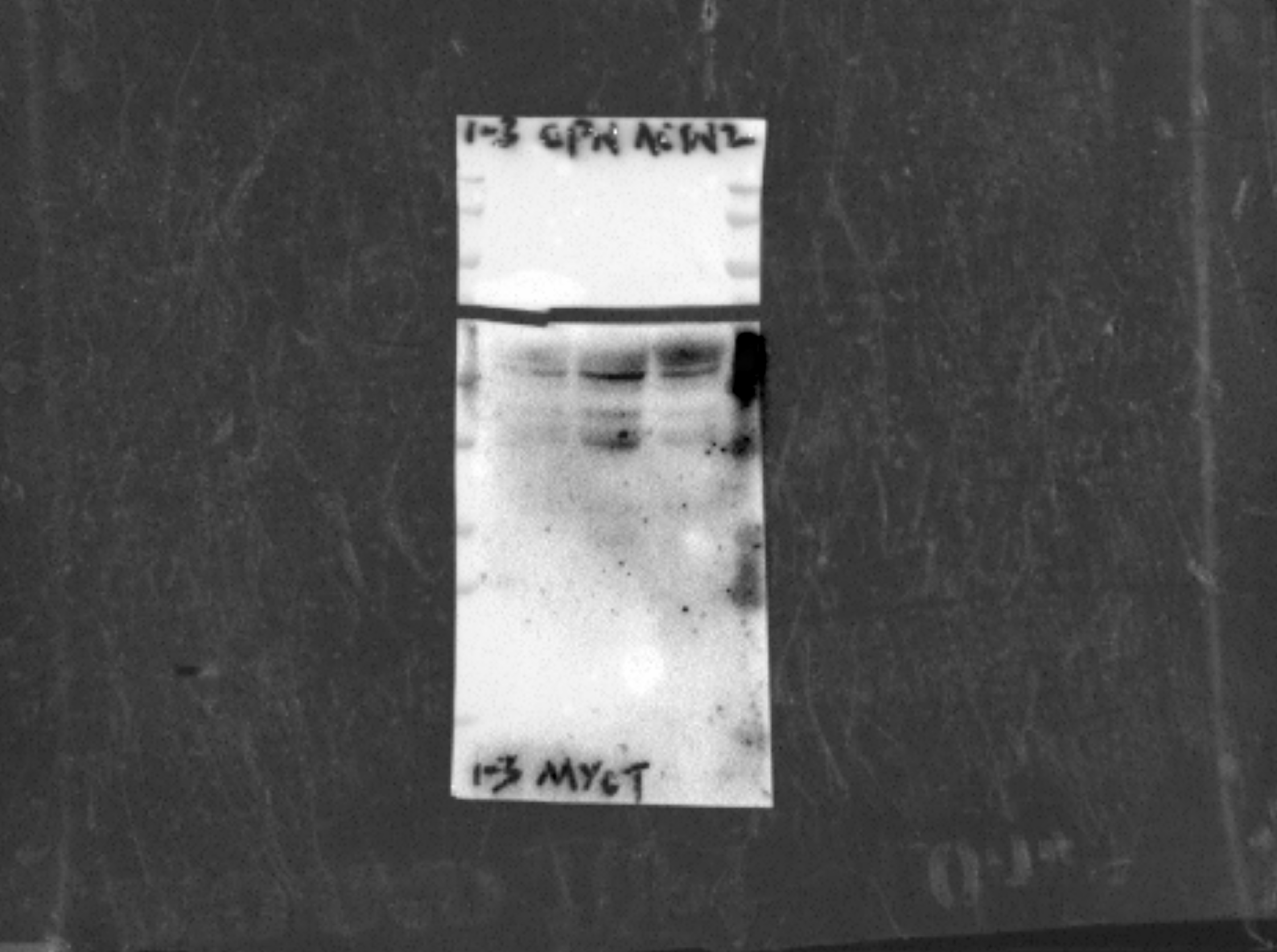

Supplement: Supplemental Information 25 [file peerj-14-21375-s025.zip › Figure 4B WB RAW oe-KLHL40 MYOT/MYOT-2 oe-KLHL40+MARK.tif]

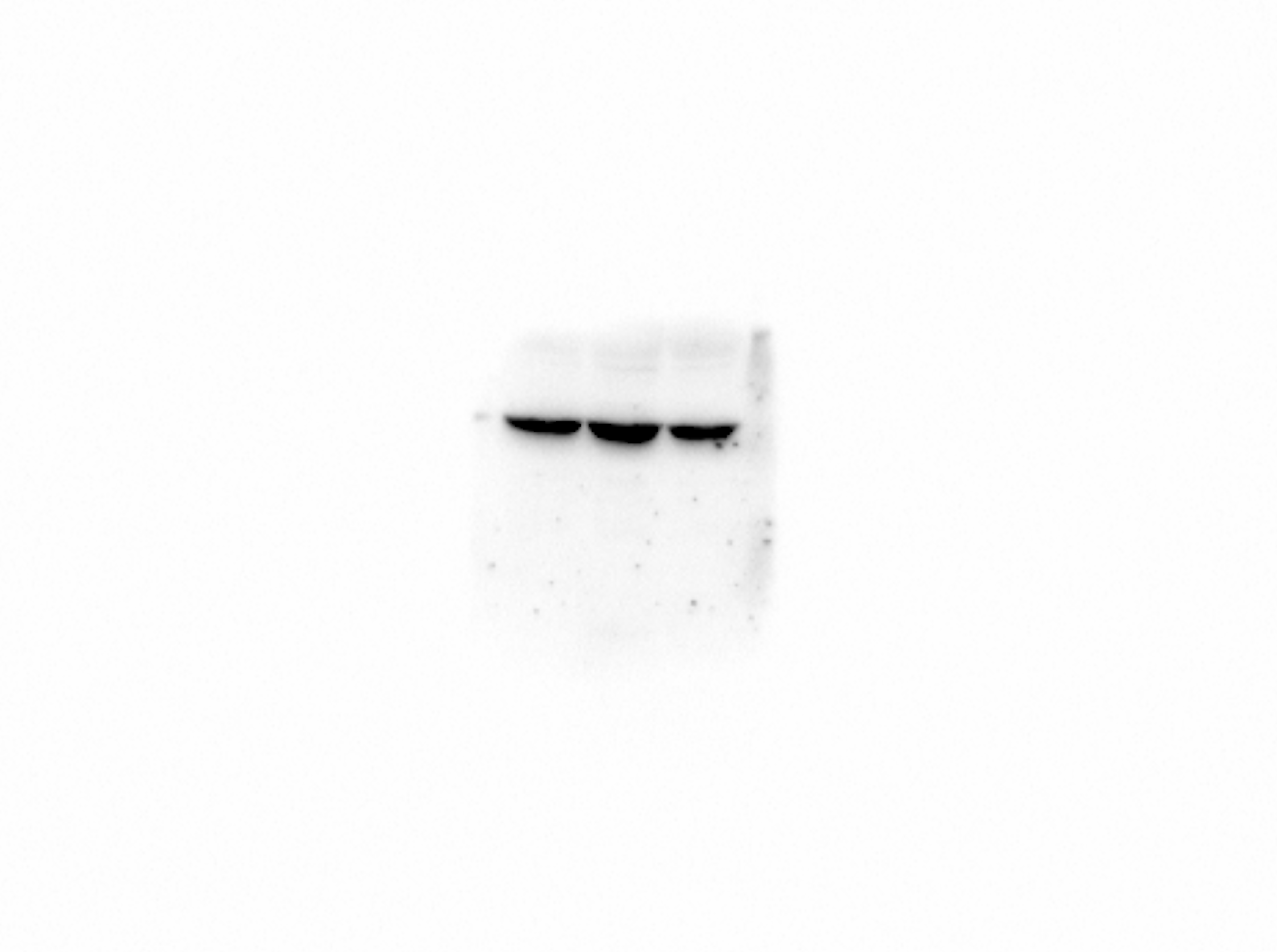

Supplement: Supplemental Information 25 [file peerj-14-21375-s025.zip › Figure 4B WB RAW oe-KLHL40 MYOT/MYOT-2 oe-KLHL40-ACTB.tif]

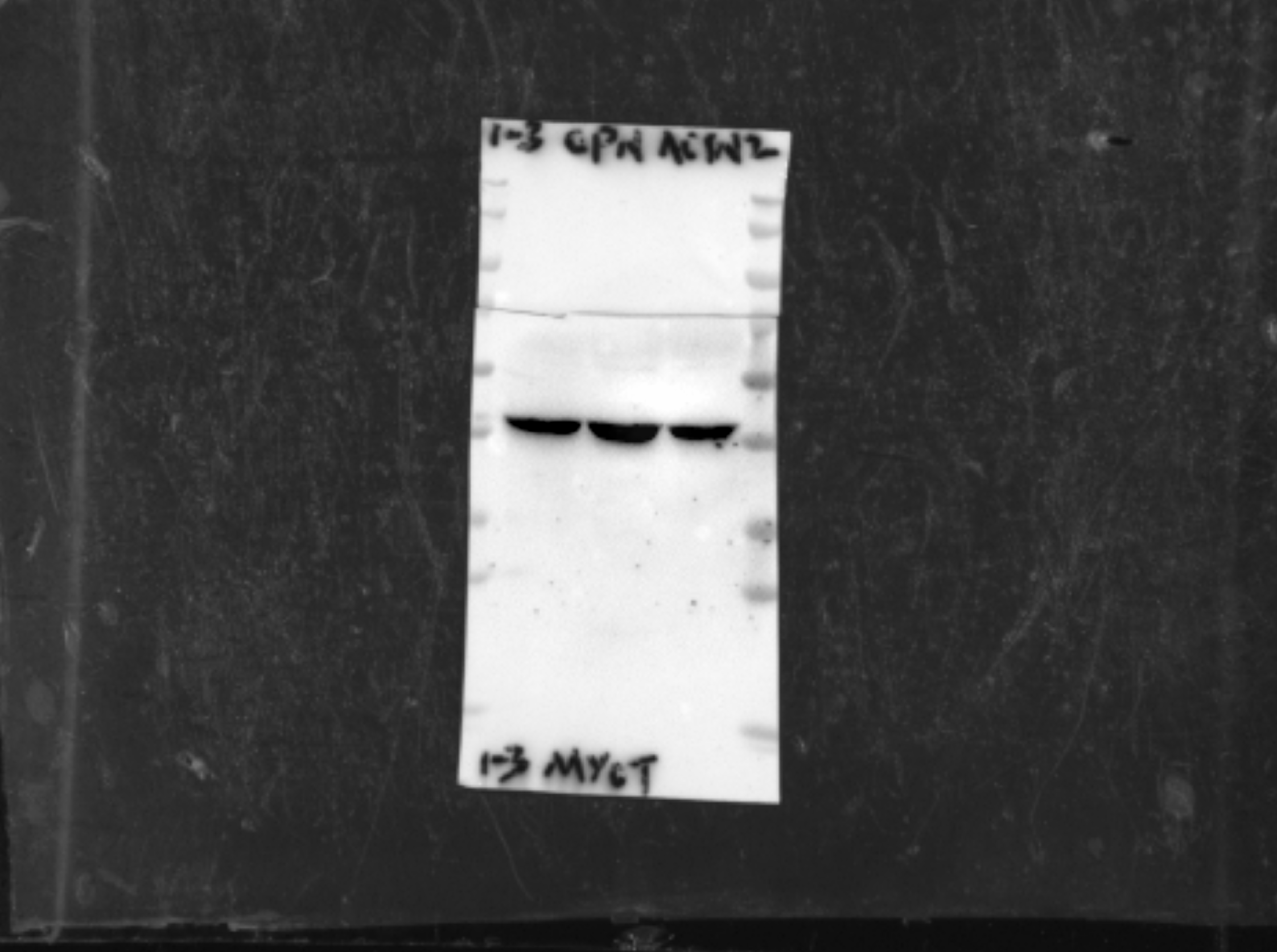

Supplement: Supplemental Information 25 [file peerj-14-21375-s025.zip › Figure 4B WB RAW oe-KLHL40 MYOT/MYOT-2 oe-KLHL40-ACTB+MARK.tif]

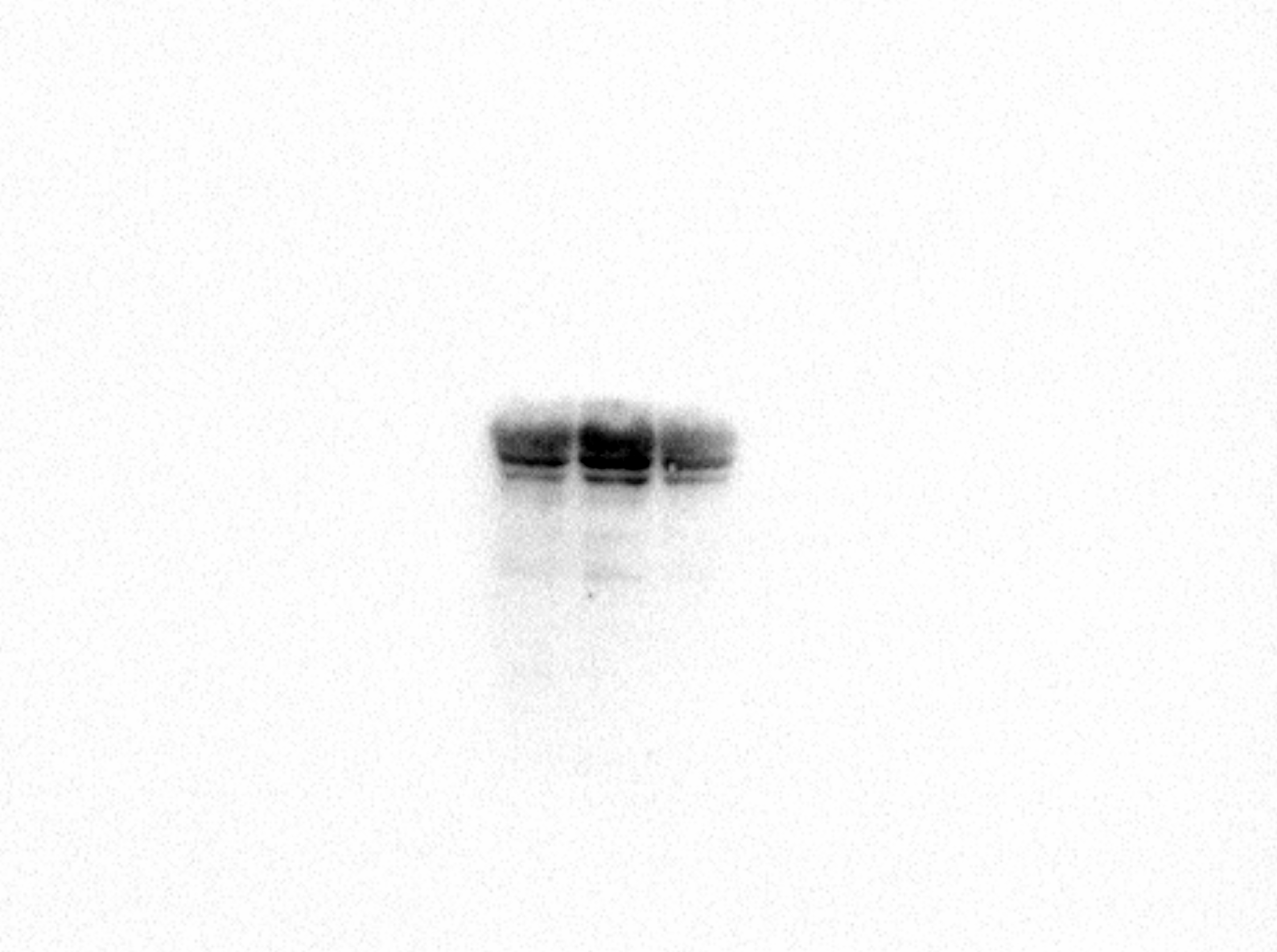

Supplement: Supplemental Information 25 [file peerj-14-21375-s025.zip › Figure 4B WB RAW oe-KLHL40 MYOT/MYOT-3 oe-KLHL40.tif]

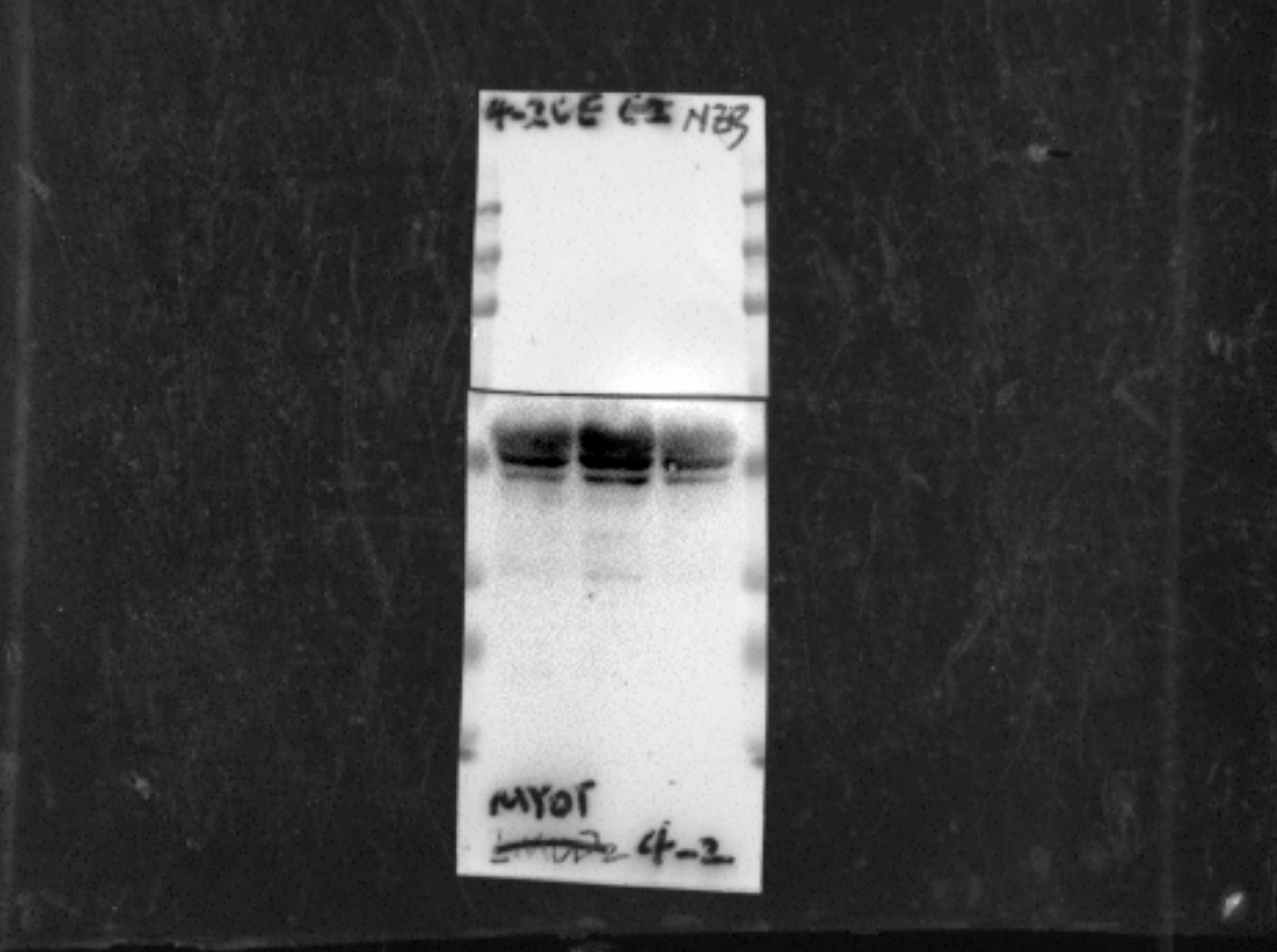

Supplement: Supplemental Information 25 [file peerj-14-21375-s025.zip › Figure 4B WB RAW oe-KLHL40 MYOT/MYOT-3 oe-KLHL40+MARK.tif]

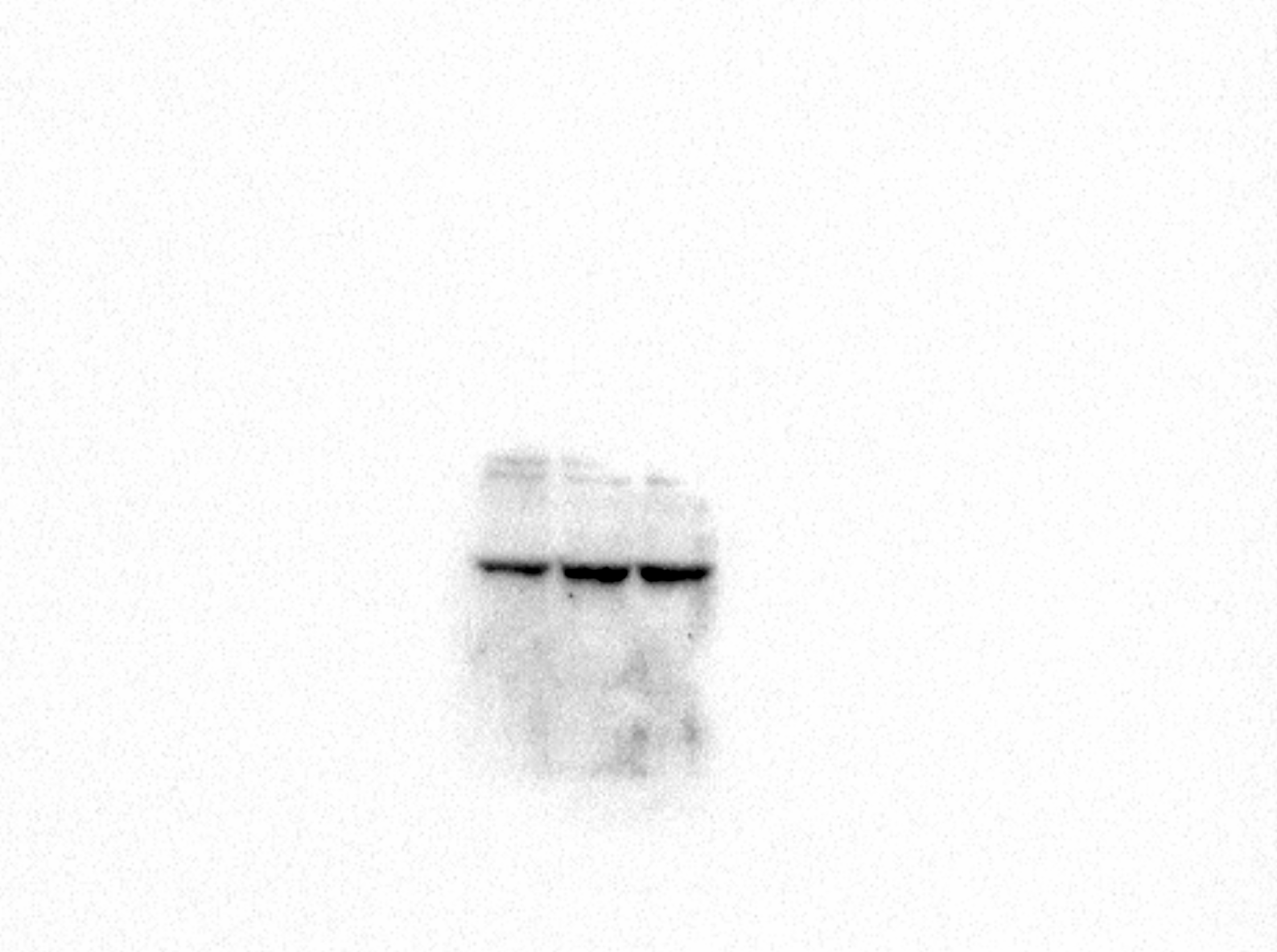

Supplement: Supplemental Information 25 [file peerj-14-21375-s025.zip › Figure 4B WB RAW oe-KLHL40 MYOT/MYOT-3 oe-KLHL40-ACTB.tif]

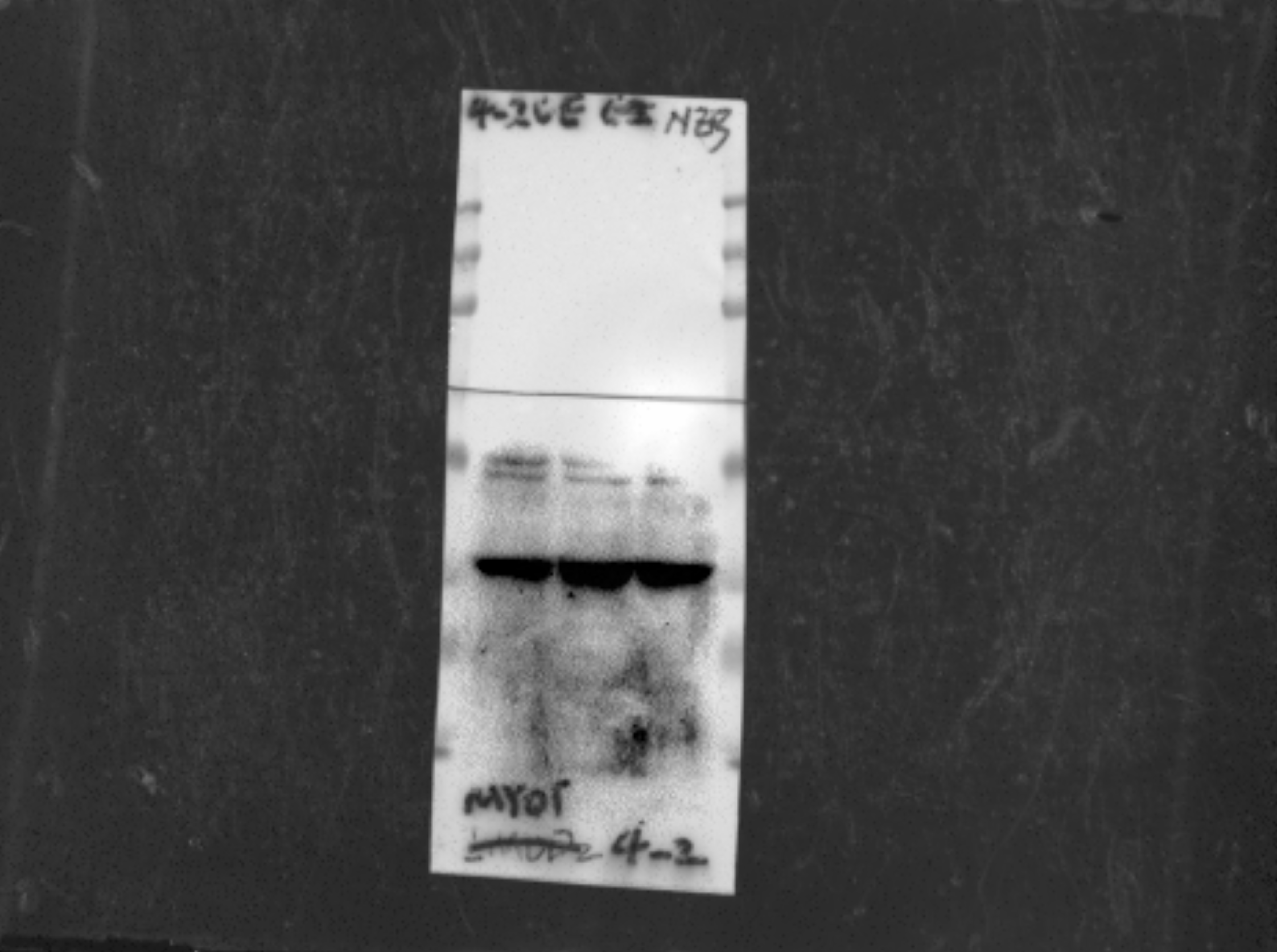

Supplement: Supplemental Information 25 [file peerj-14-21375-s025.zip › Figure 4B WB RAW oe-KLHL40 MYOT/MYOT-3 oe-KLHL40-ACTB+MARK.tif]

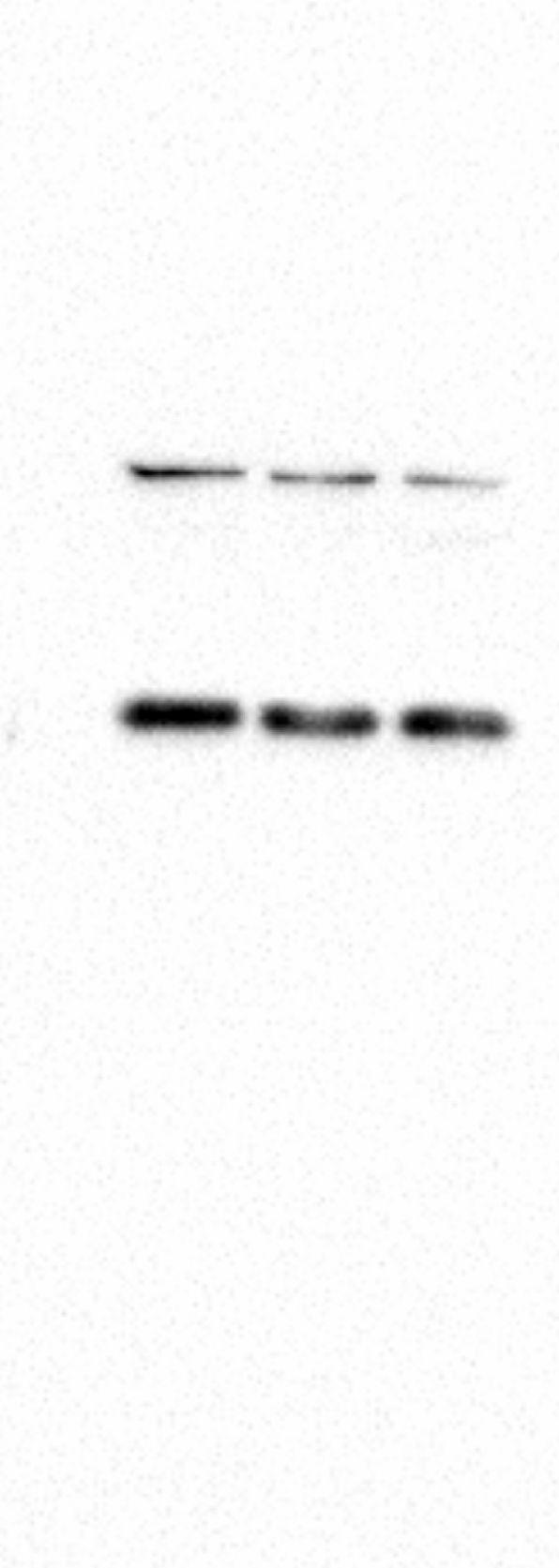

Supplement: Supplemental Information 26 [file peerj-14-21375-s026.zip › Figure 4C WB RAW sh-KLHL40 CAPZA/CAPZA-1 sh-KLHL40.tif]

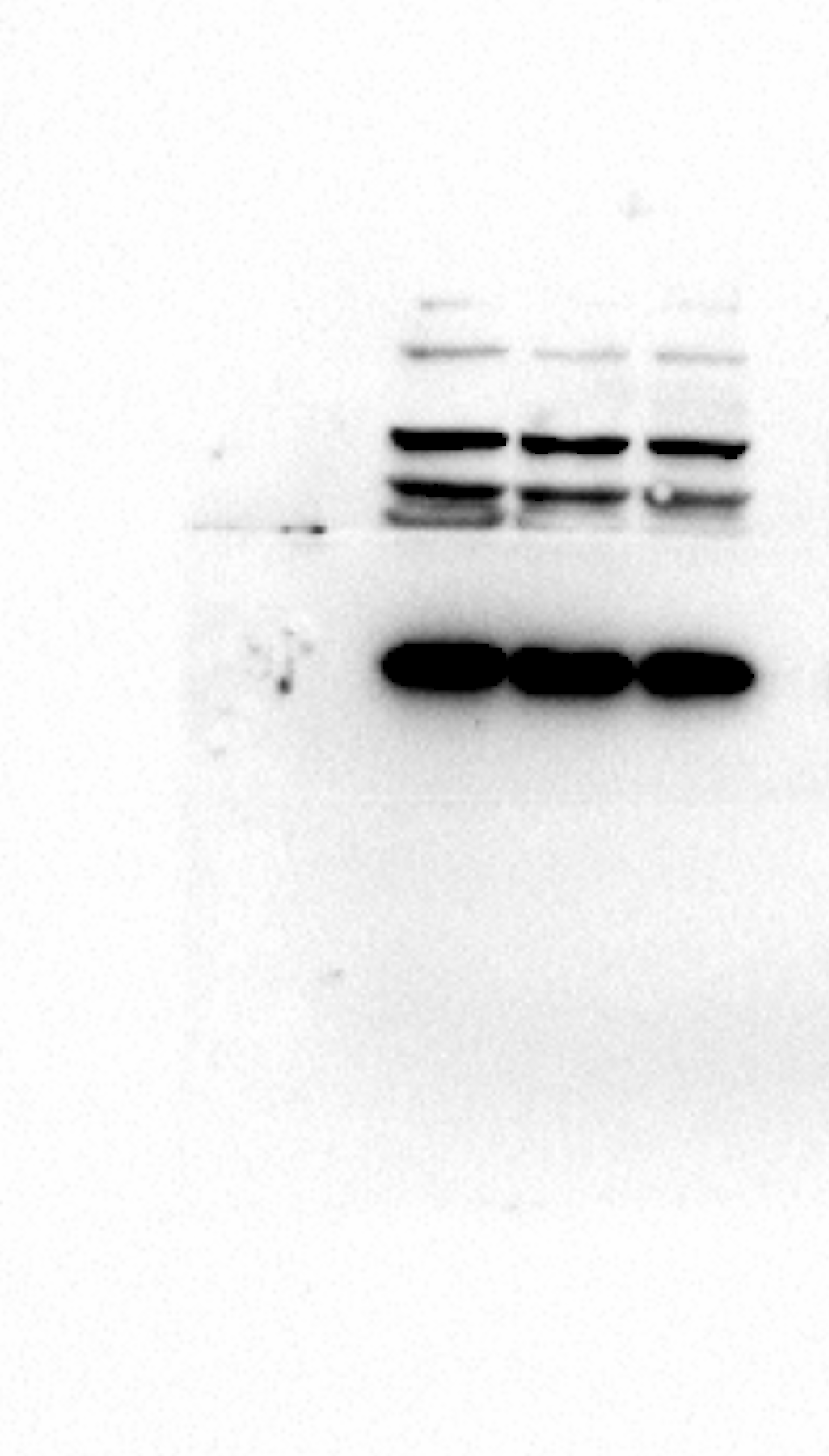

Supplement: Supplemental Information 26 [file peerj-14-21375-s026.zip › Figure 4C WB RAW sh-KLHL40 CAPZA/CAPZA-1 sh-KLHL40+ACTB.tif]

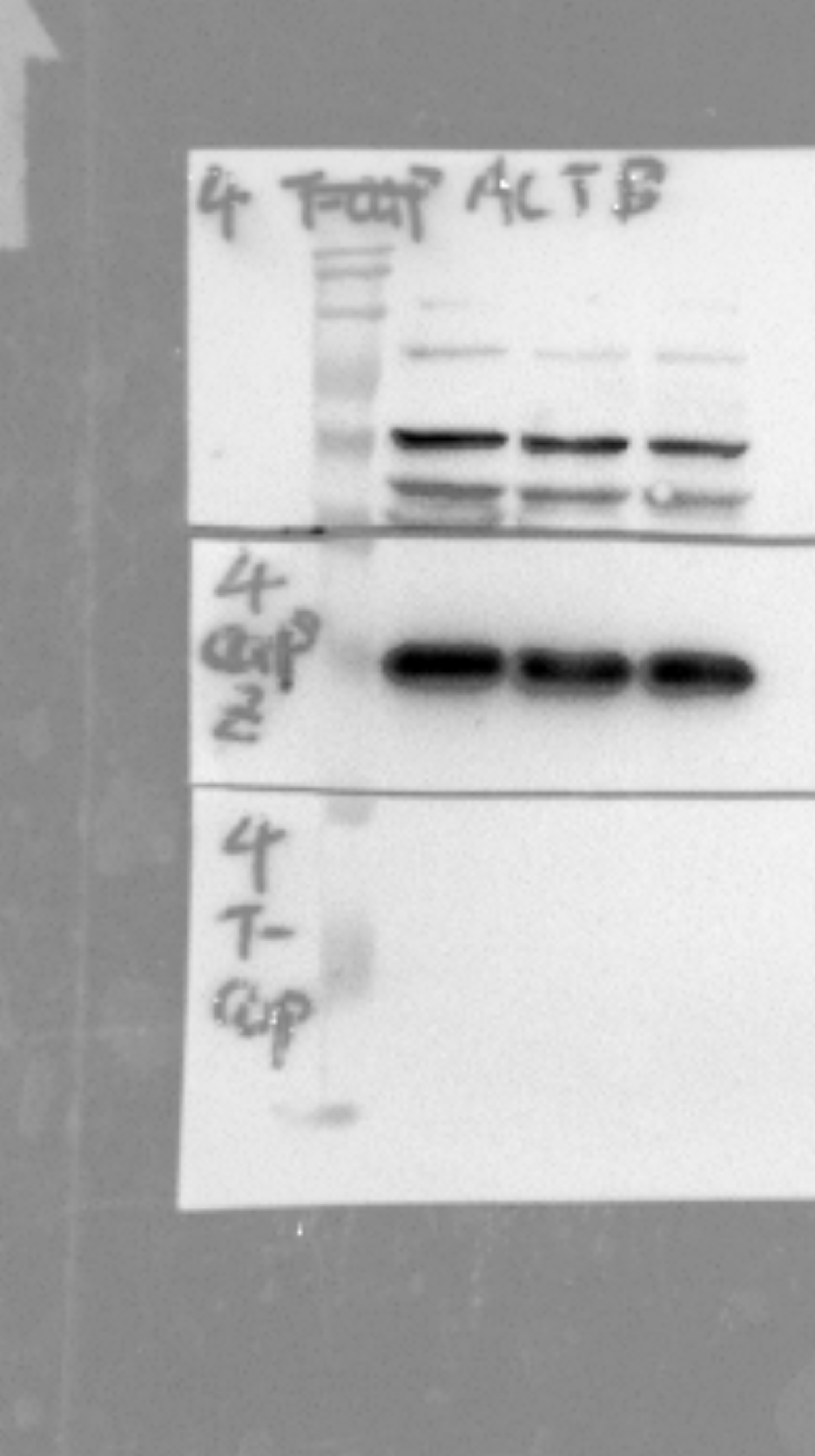

Supplement: Supplemental Information 26 [file peerj-14-21375-s026.zip › Figure 4C WB RAW sh-KLHL40 CAPZA/CAPZA-1 sh-KLHL40+ACTB+MARK.tif]

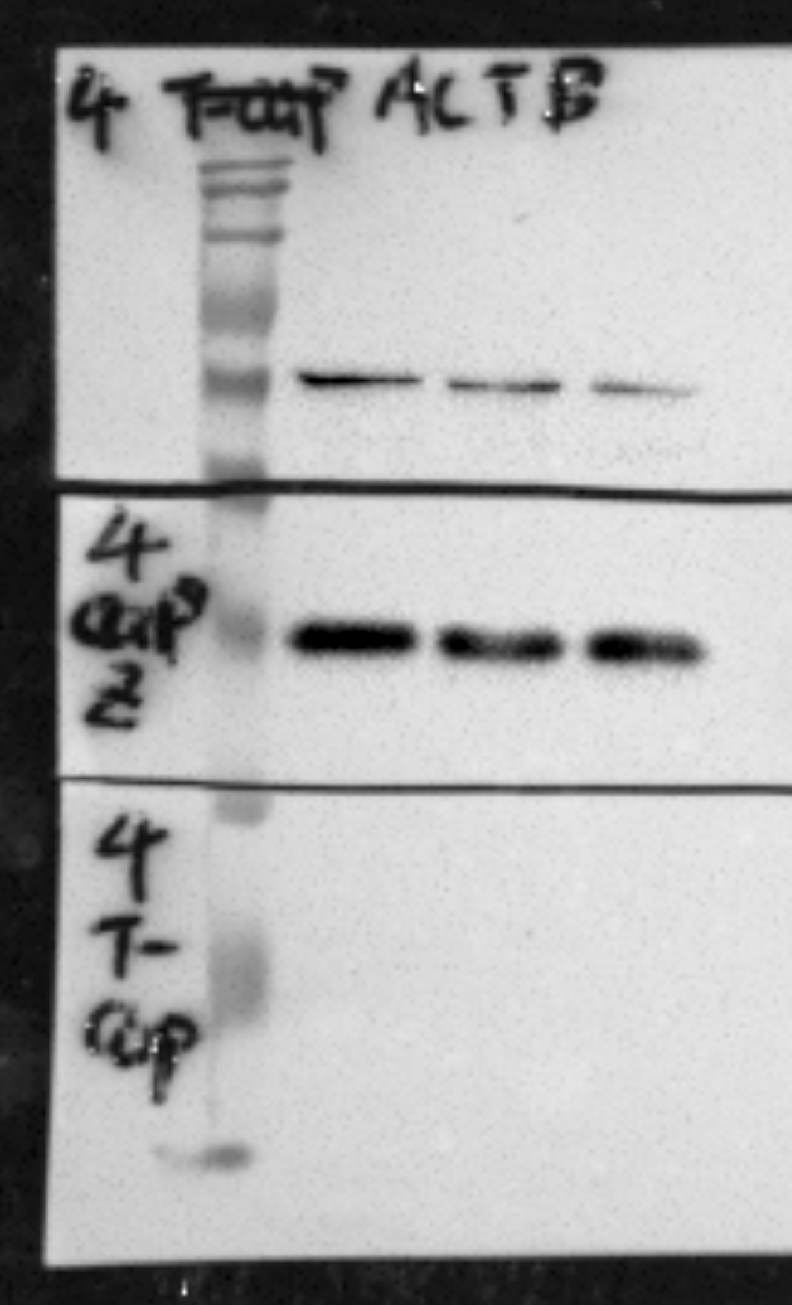

Supplement: Supplemental Information 26 [file peerj-14-21375-s026.zip › Figure 4C WB RAW sh-KLHL40 CAPZA/CAPZA-1 sh-KLHL40+MARK.tif]

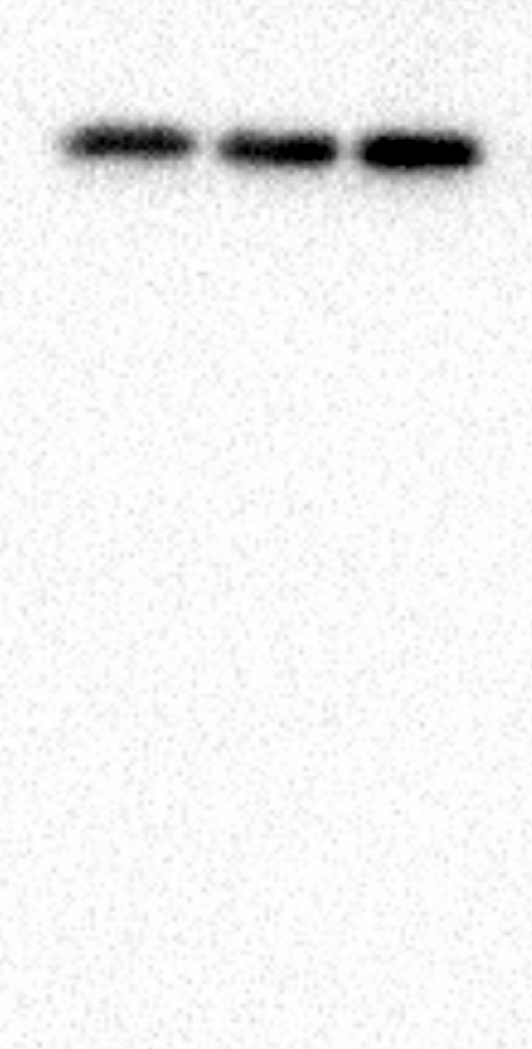

Supplement: Supplemental Information 26 [file peerj-14-21375-s026.zip › Figure 4C WB RAW sh-KLHL40 CAPZA/CAPZA-2 sh-KLHL40.tif]

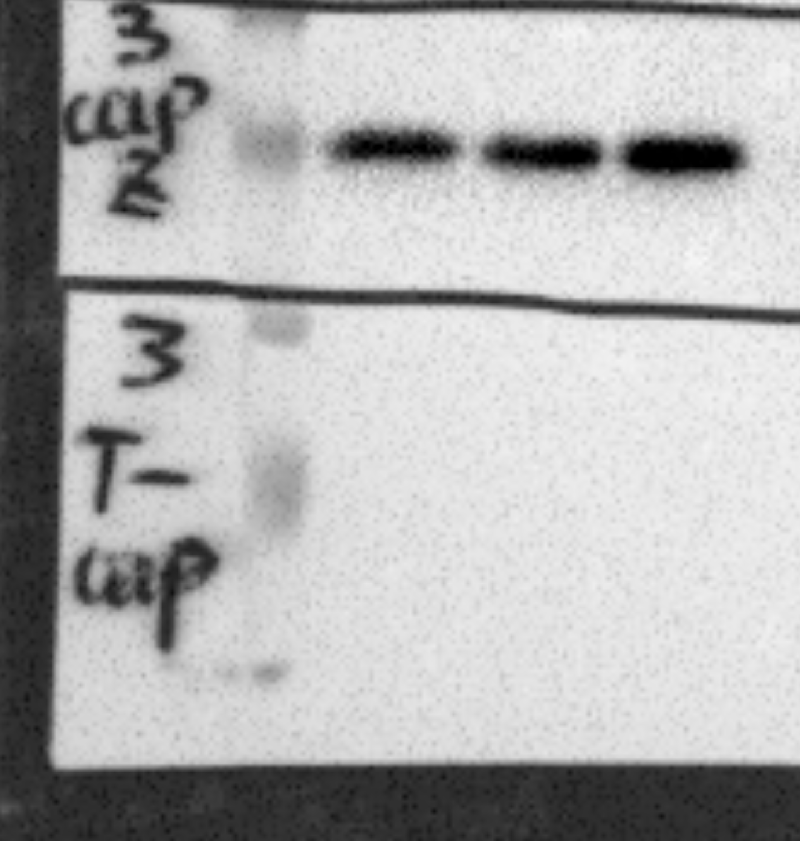

Supplement: Supplemental Information 26 [file peerj-14-21375-s026.zip › Figure 4C WB RAW sh-KLHL40 CAPZA/CAPZA-2 sh-KLHL40+MARK.tif]

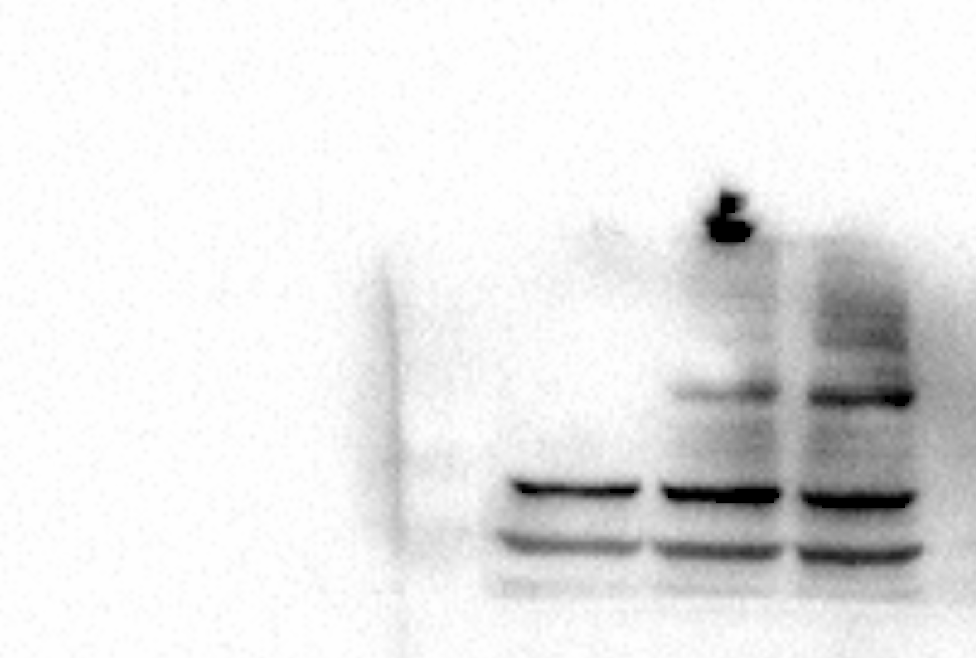

Supplement: Supplemental Information 26 [file peerj-14-21375-s026.zip › Figure 4C WB RAW sh-KLHL40 CAPZA/CAPZA-2 sh-KLHL40-ACTB.tif]

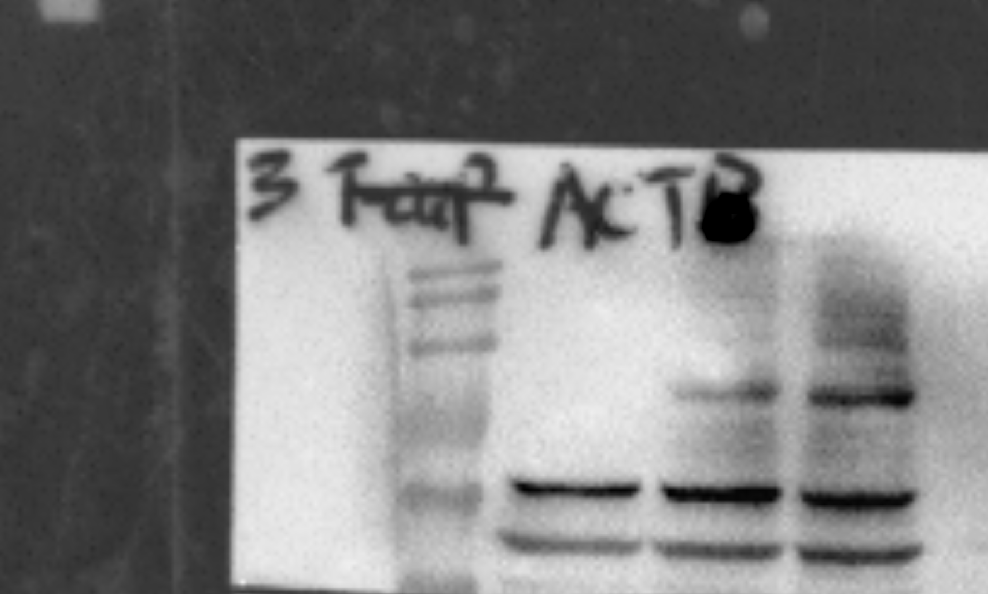

Supplement: Supplemental Information 26 [file peerj-14-21375-s026.zip › Figure 4C WB RAW sh-KLHL40 CAPZA/CAPZA-2 sh-KLHL40-ACTB+MARK.tif]

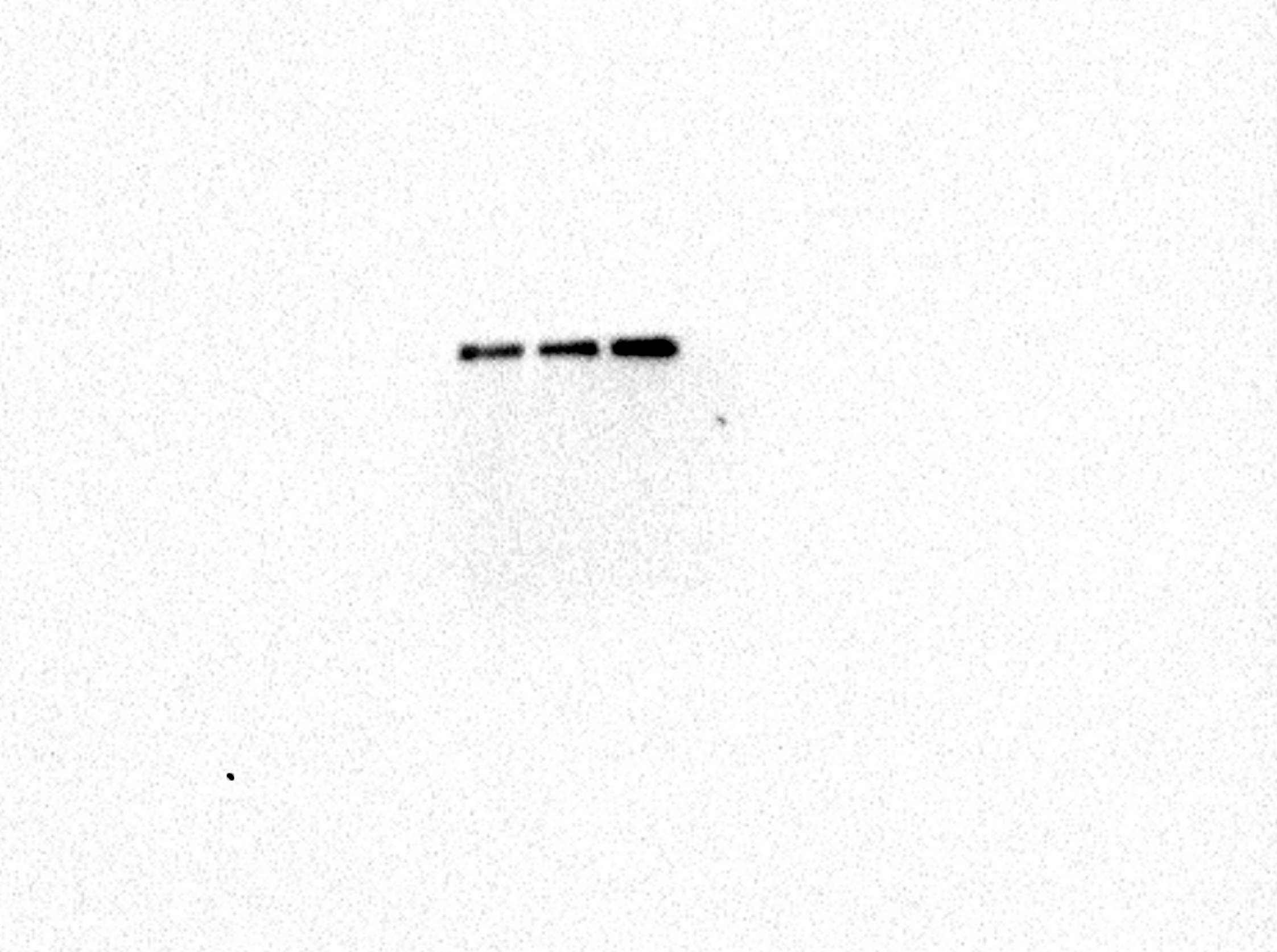

Supplement: Supplemental Information 26 [file peerj-14-21375-s026.zip › Figure 4C WB RAW sh-KLHL40 CAPZA/CAPZA-3 sh-KLHL40.tif]

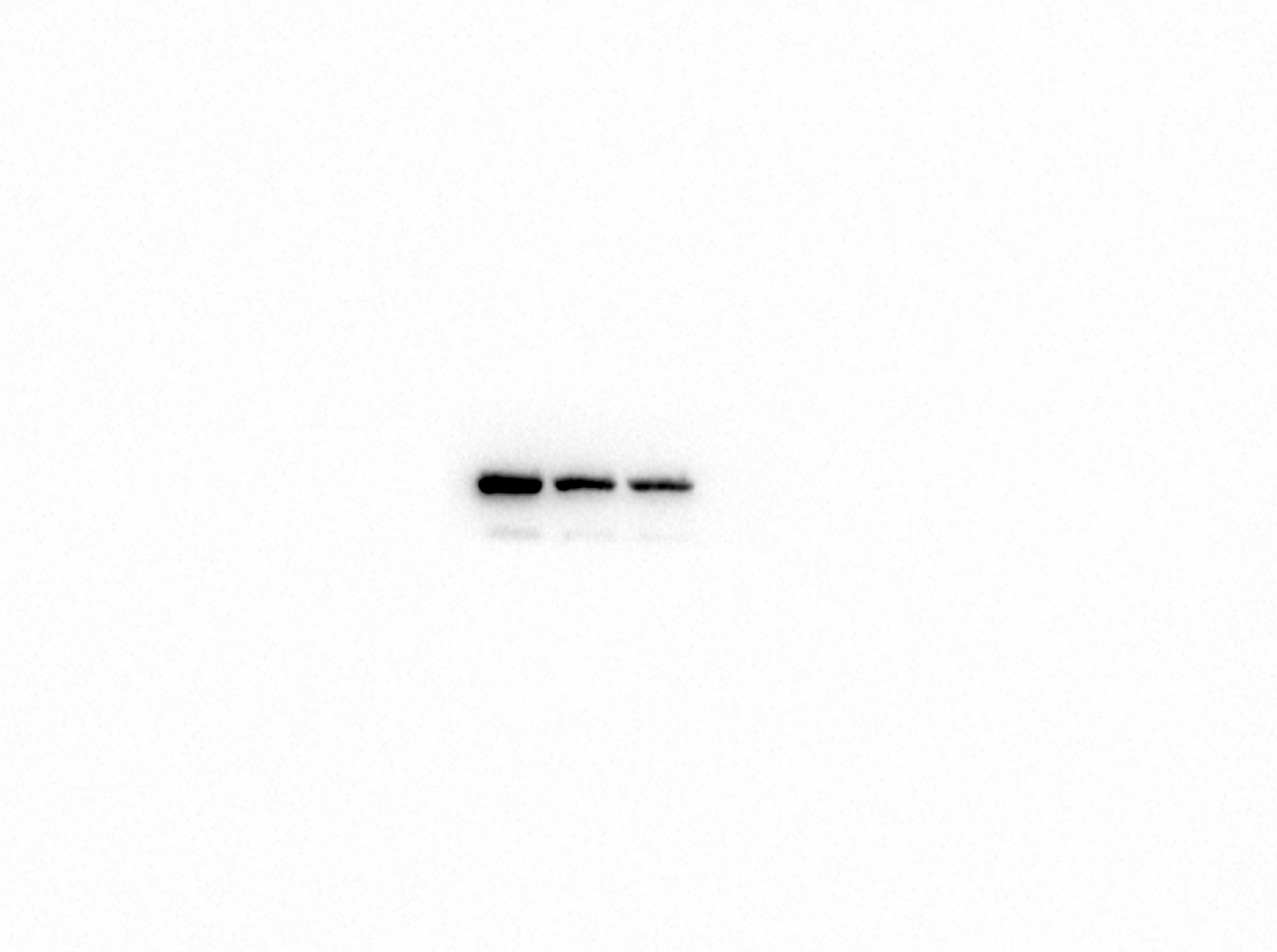

Supplement: Supplemental Information 26 [file peerj-14-21375-s026.zip › Figure 4C WB RAW sh-KLHL40 CAPZA/CAPZA-3 sh-KLHL40-ACTB.tif]

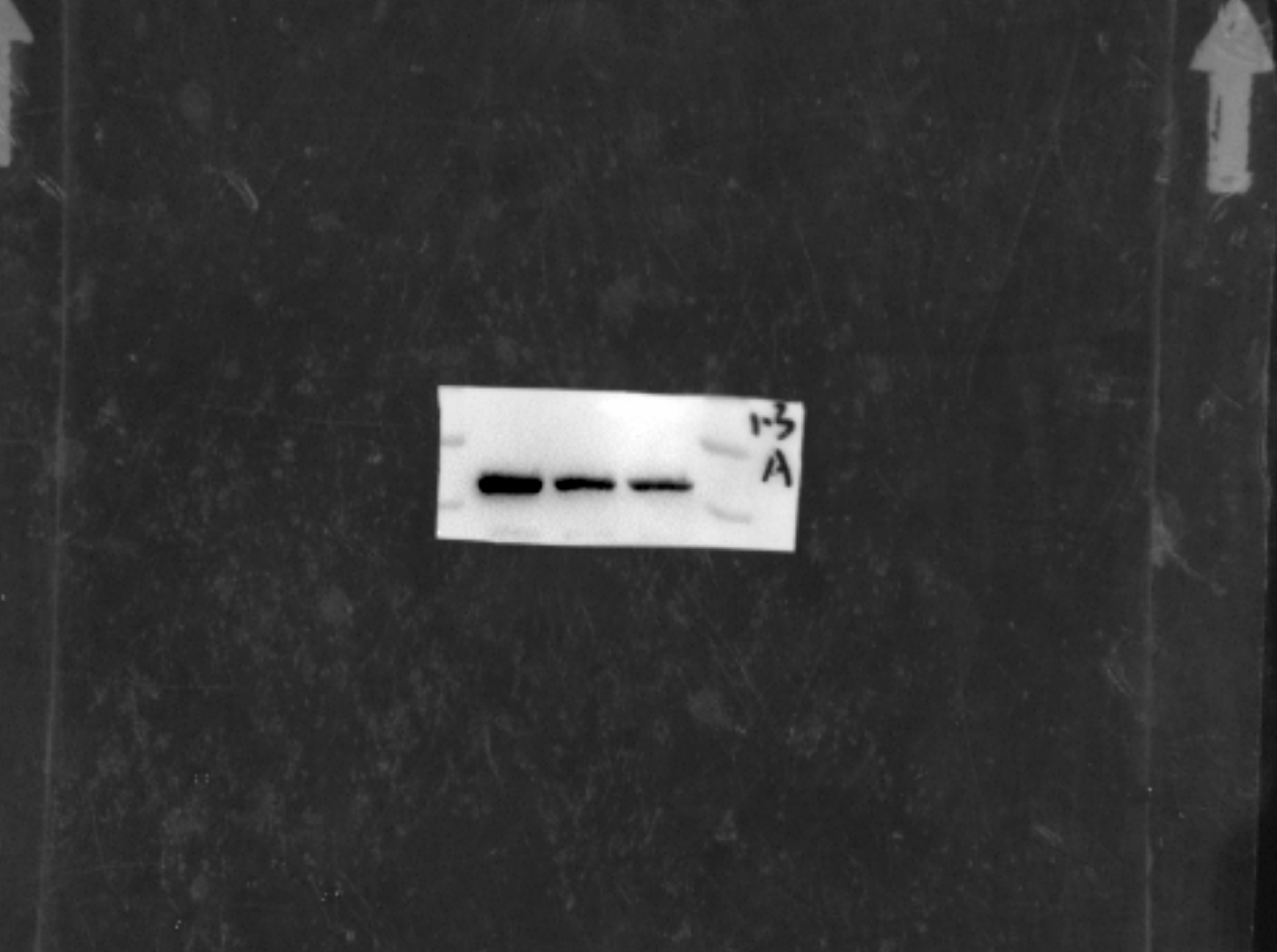

Supplement: Supplemental Information 26 [file peerj-14-21375-s026.zip › Figure 4C WB RAW sh-KLHL40 CAPZA/CAPZA-3 sh-KLHL40-ACTB+MARK.tif]

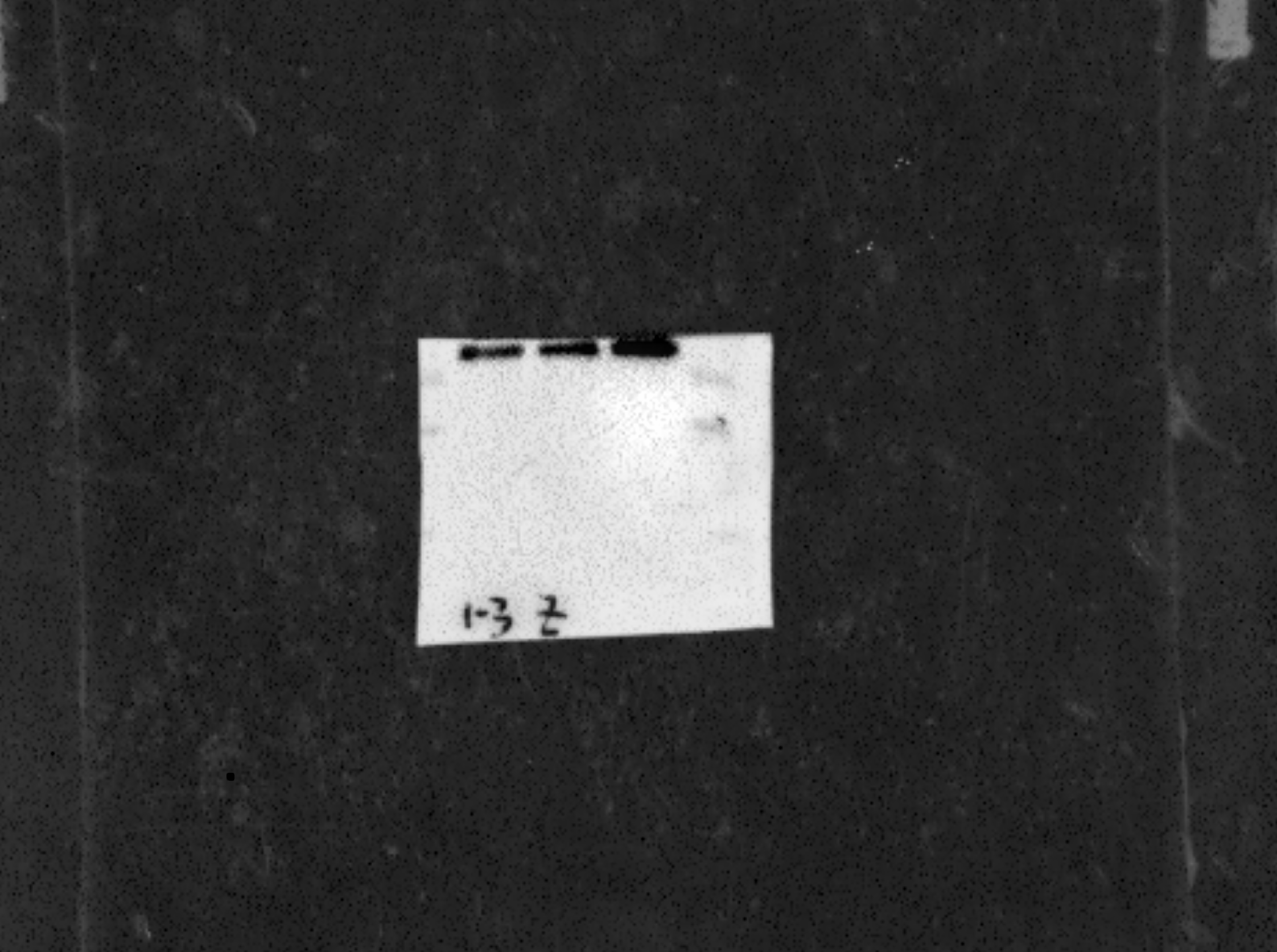

Supplement: Supplemental Information 26 [file peerj-14-21375-s026.zip › Figure 4C WB RAW sh-KLHL40 CAPZA/CAPZA-3 sh-KLHL40-MARK.tif]

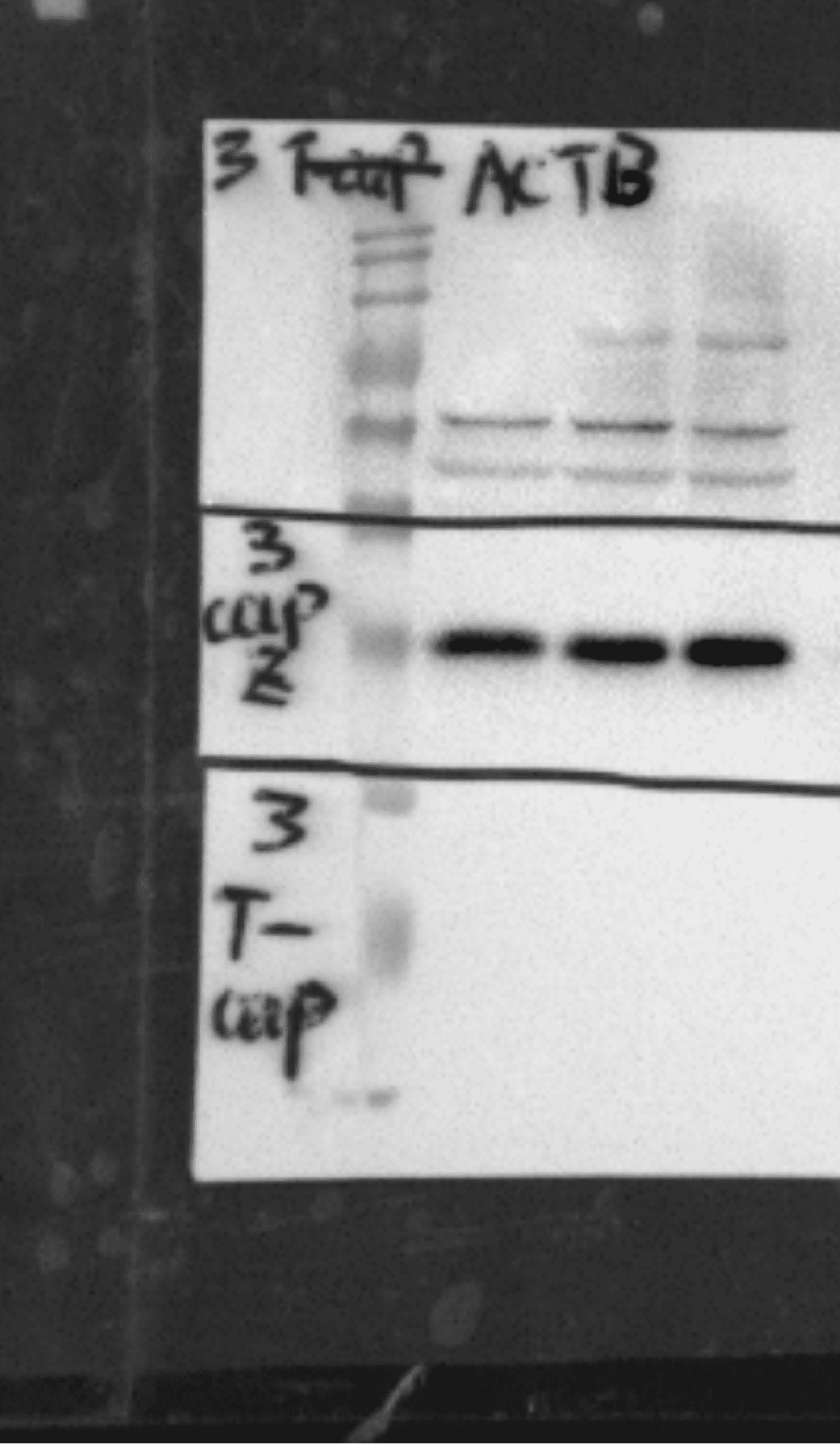

Supplement: Supplemental Information 26 [file peerj-14-21375-s026.zip › Figure 4C WB RAW sh-KLHL40 CAPZA/TOTAL-2.png]

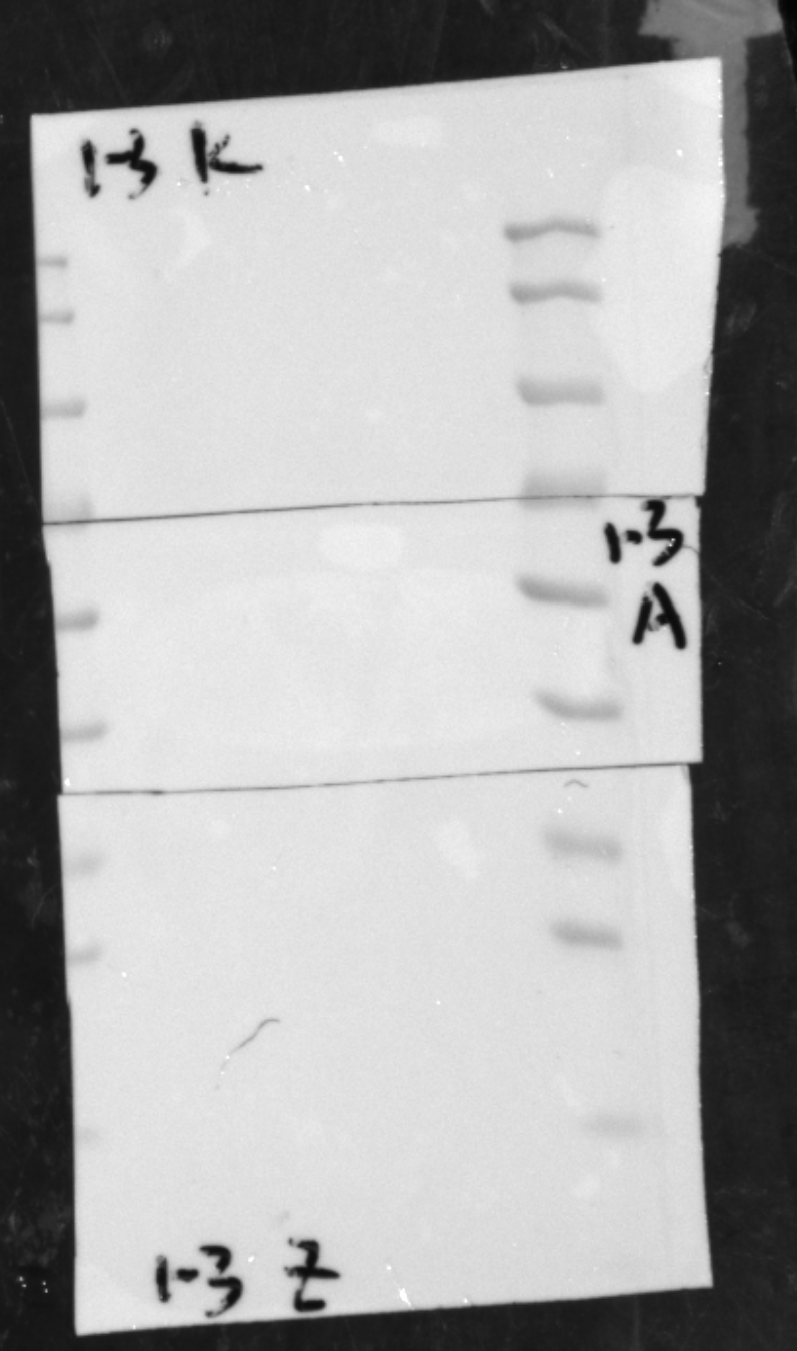

Supplement: Supplemental Information 26 [file peerj-14-21375-s026.zip › Figure 4C WB RAW sh-KLHL40 CAPZA/TOTAL-3.tif]

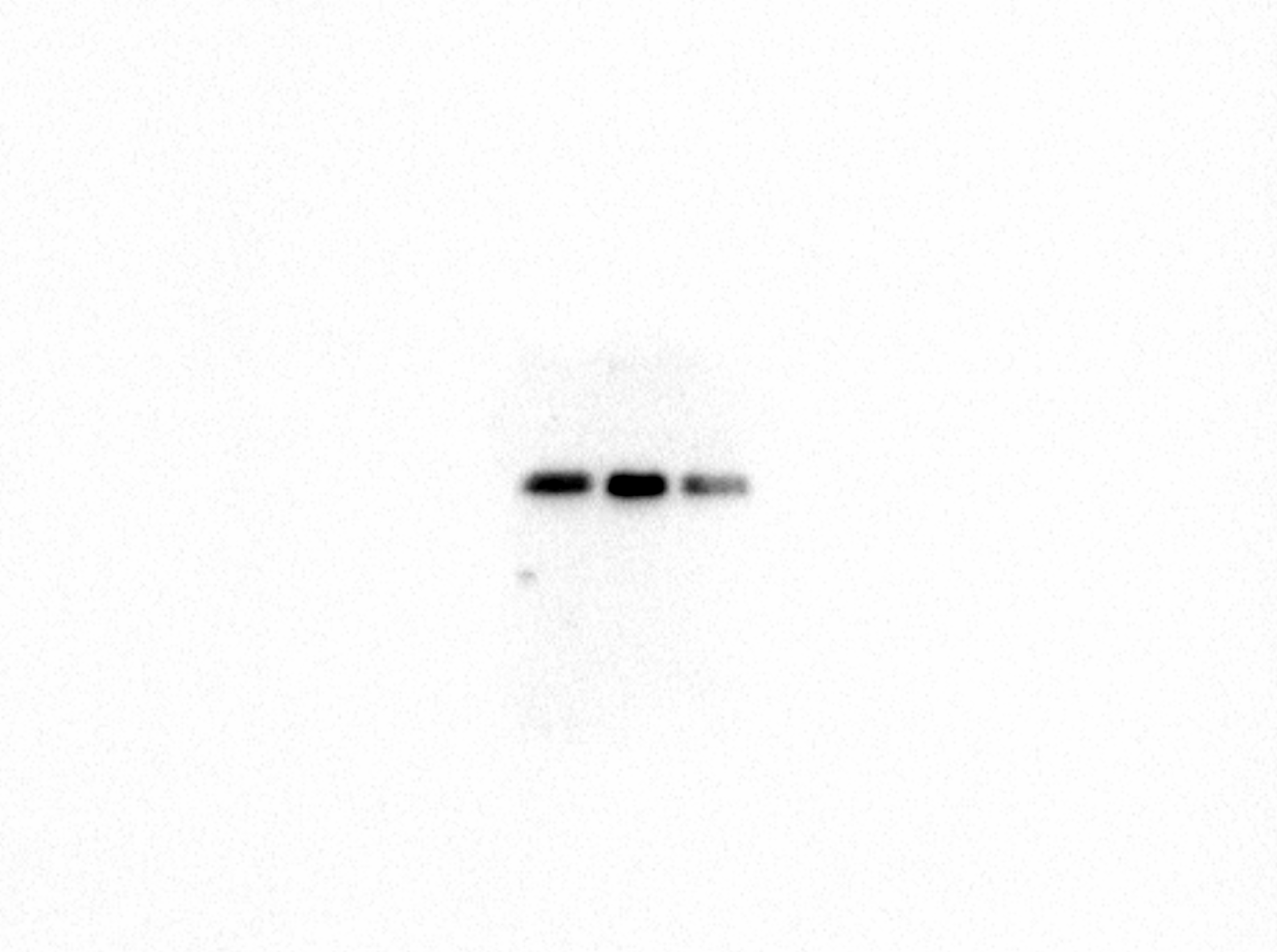

Supplement: Supplemental Information 27 [file peerj-14-21375-s027.zip › Figure 4D WB RAW oe-KLHL40 CAPZA/CAPZA-1 oe-KLHL40.tif]

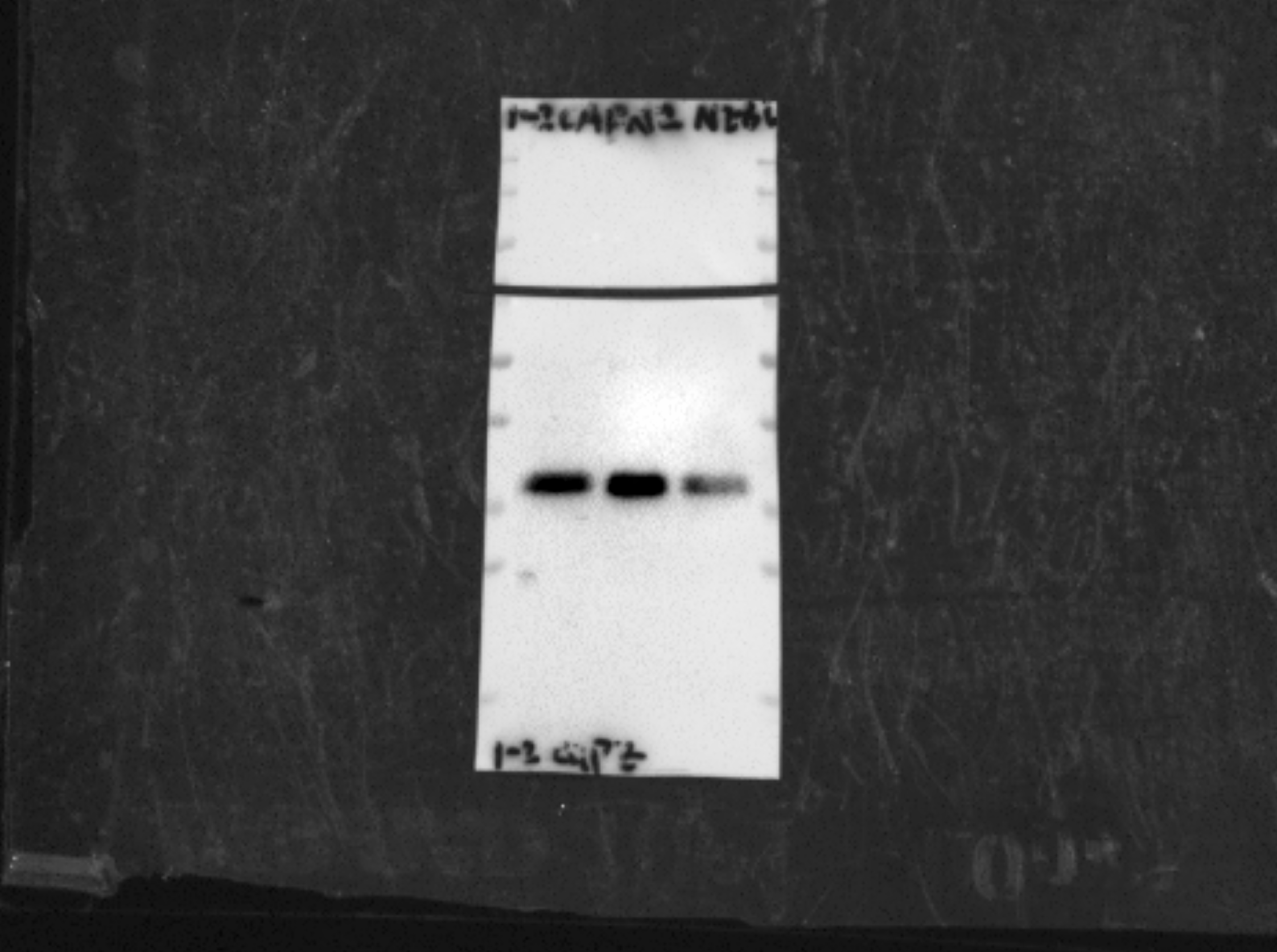

Supplement: Supplemental Information 27 [file peerj-14-21375-s027.zip › Figure 4D WB RAW oe-KLHL40 CAPZA/CAPZA-1 oe-KLHL40+MARK.tif]

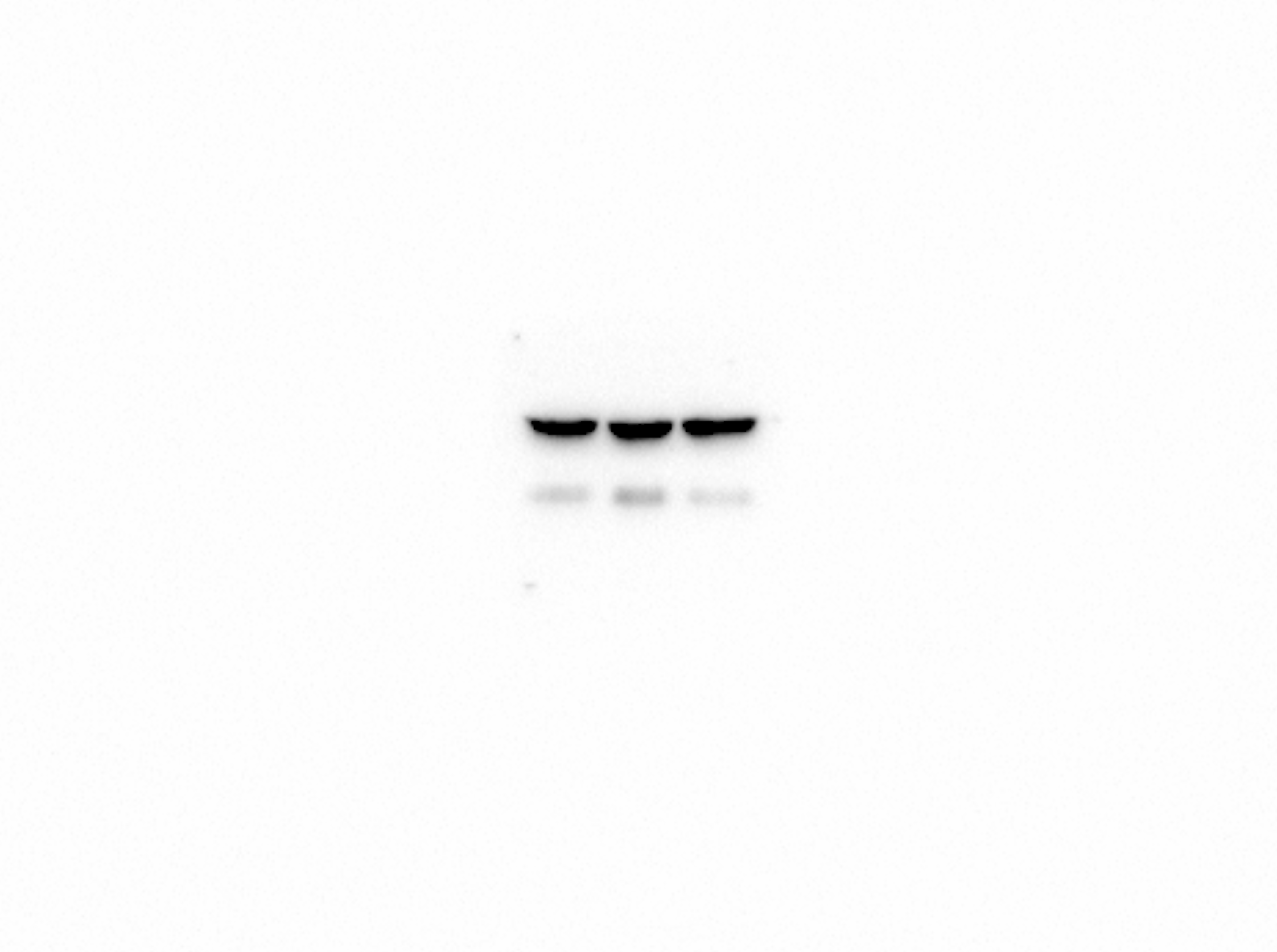

Supplement: Supplemental Information 27 [file peerj-14-21375-s027.zip › Figure 4D WB RAW oe-KLHL40 CAPZA/CAPZA-1 oe-KLHL40-ACTB.tif]

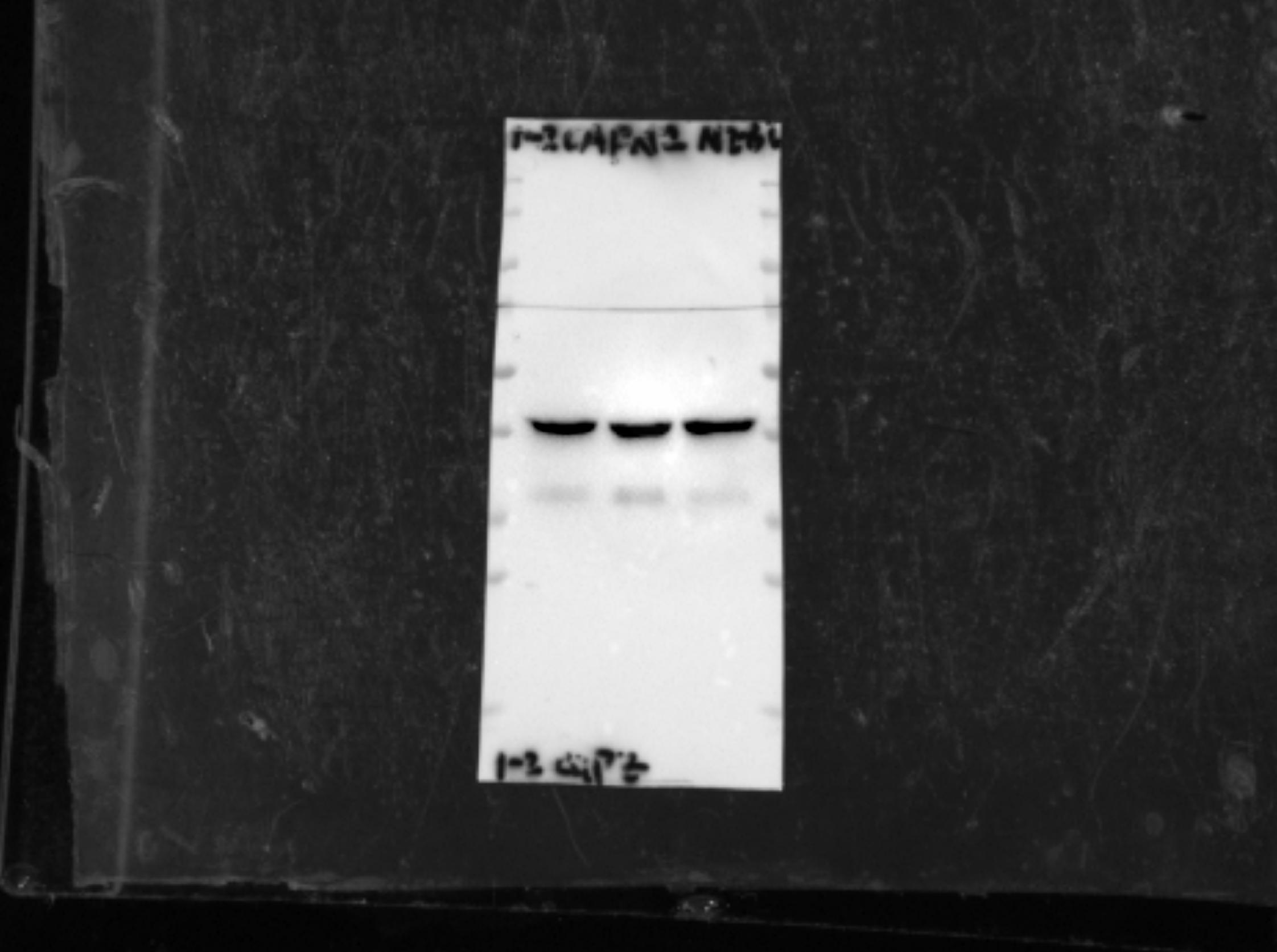

Supplement: Supplemental Information 27 [file peerj-14-21375-s027.zip › Figure 4D WB RAW oe-KLHL40 CAPZA/CAPZA-1 oe-KLHL40-ACTB+MARK.tif]

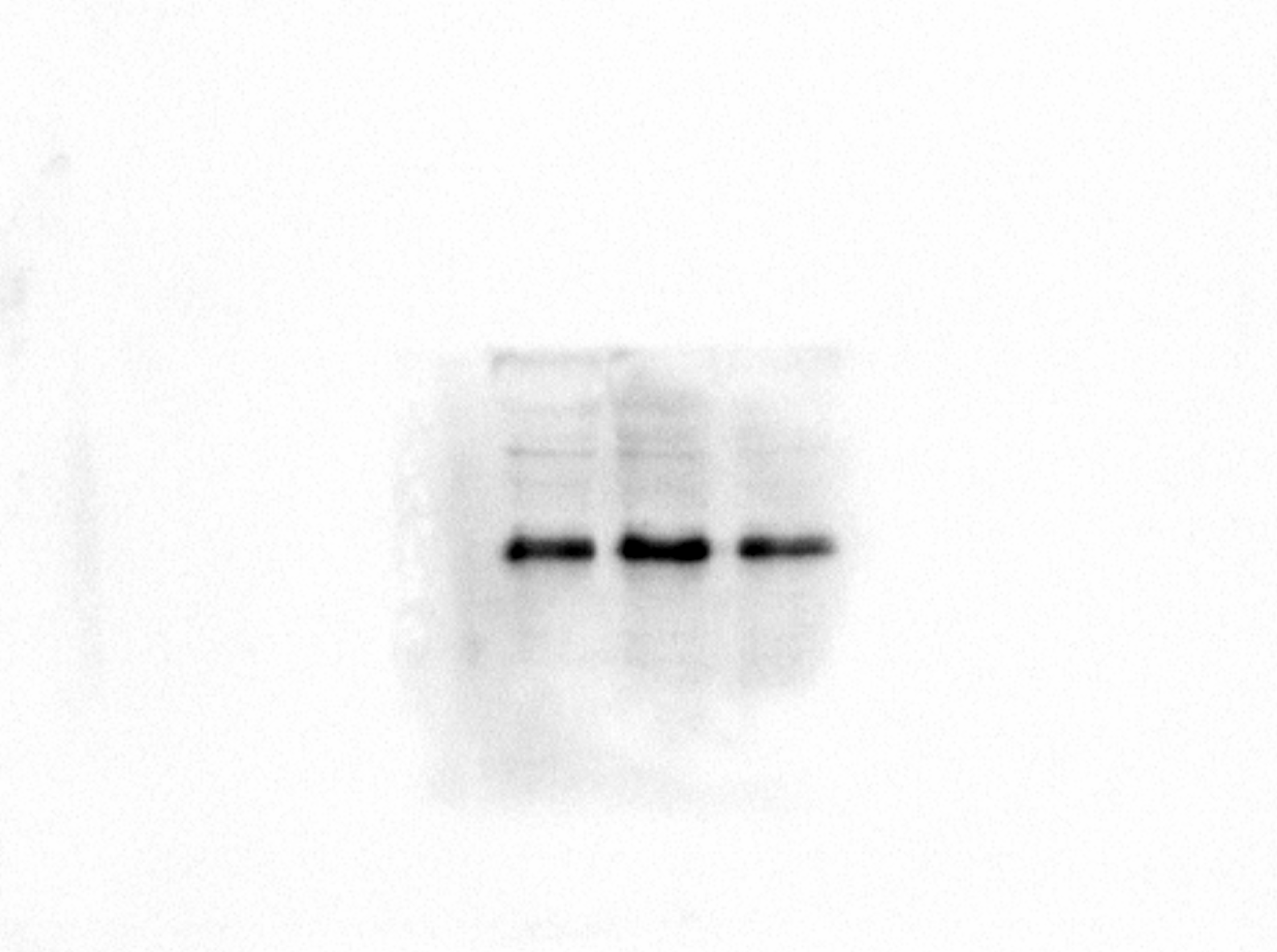

Supplement: Supplemental Information 27 [file peerj-14-21375-s027.zip › Figure 4D WB RAW oe-KLHL40 CAPZA/CAPZA-2 oe-KLHL40.tif]

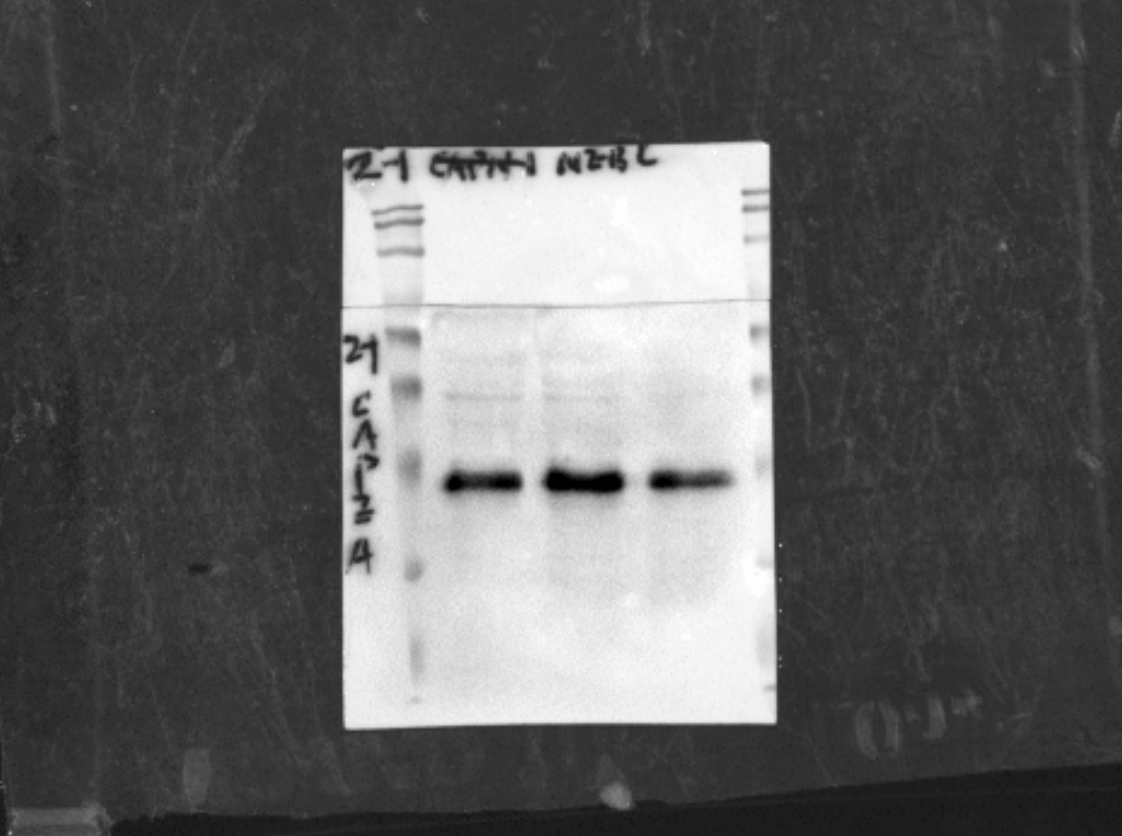

Supplement: Supplemental Information 27 [file peerj-14-21375-s027.zip › Figure 4D WB RAW oe-KLHL40 CAPZA/CAPZA-2 oe-KLHL40+MARK.tif]

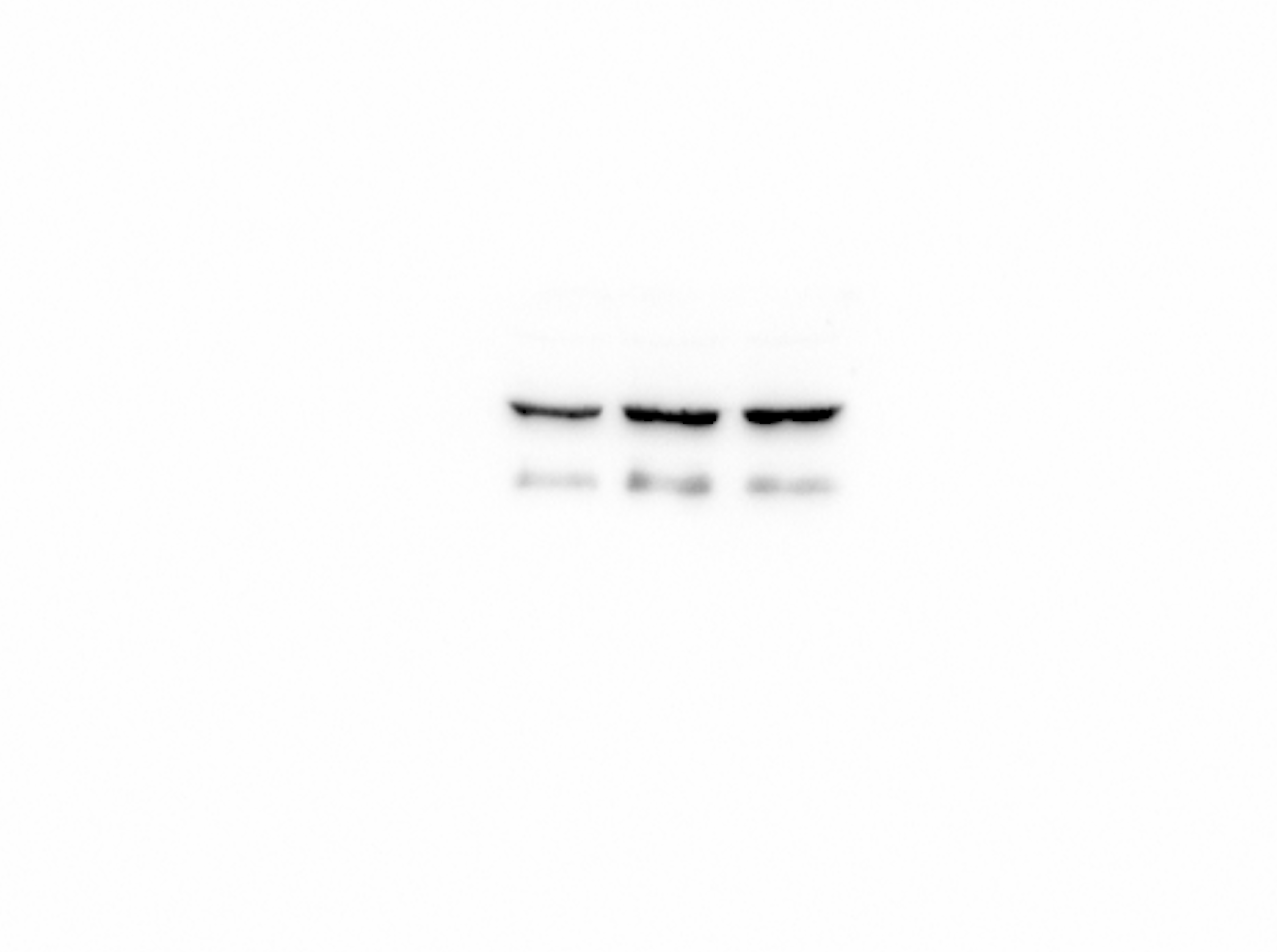

Supplement: Supplemental Information 27 [file peerj-14-21375-s027.zip › Figure 4D WB RAW oe-KLHL40 CAPZA/CAPZA-2 oe-KLHL40-ACTB.tif]

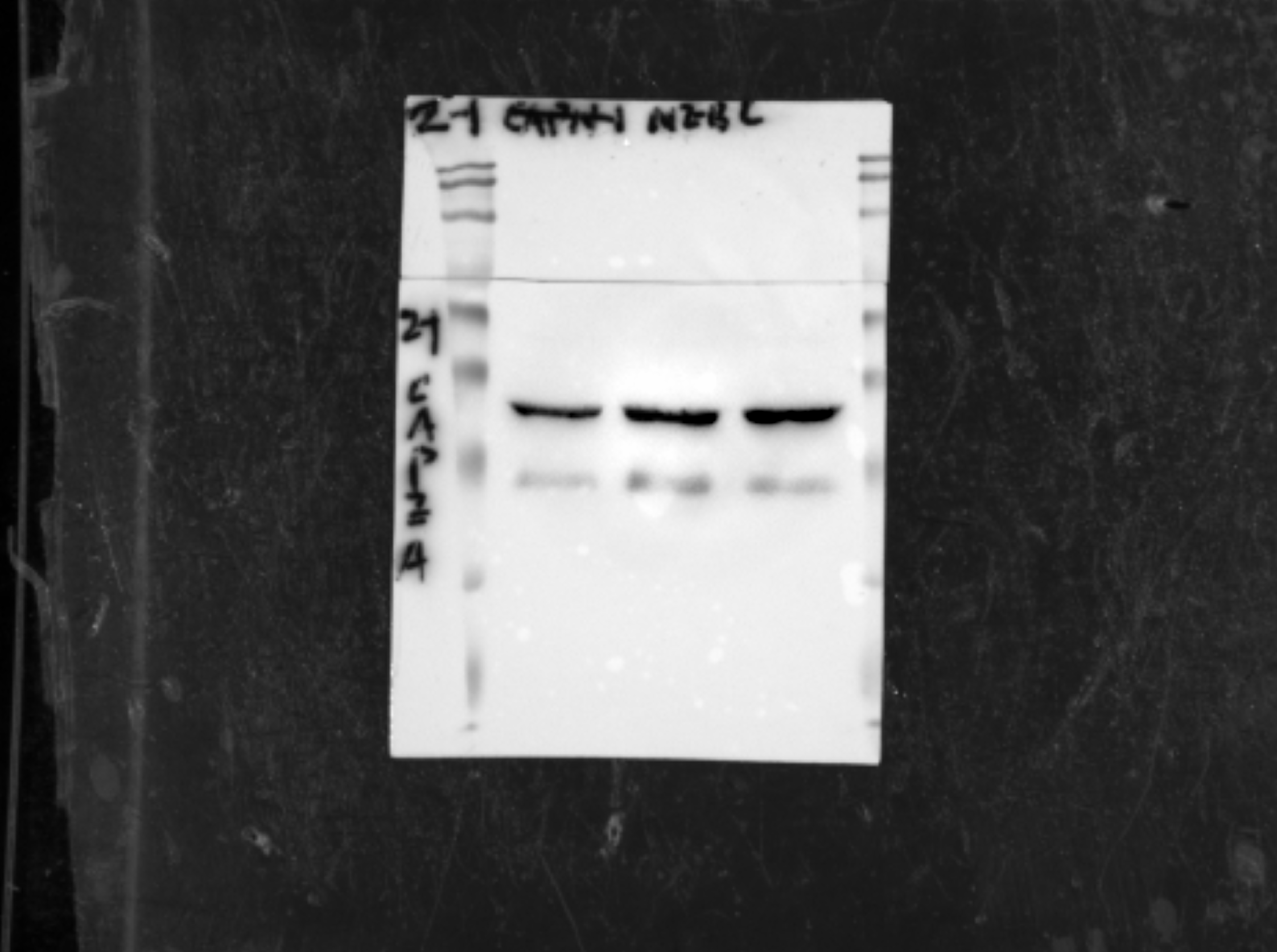

Supplement: Supplemental Information 27 [file peerj-14-21375-s027.zip › Figure 4D WB RAW oe-KLHL40 CAPZA/CAPZA-2 oe-KLHL40-ACTB+MARK.tif]

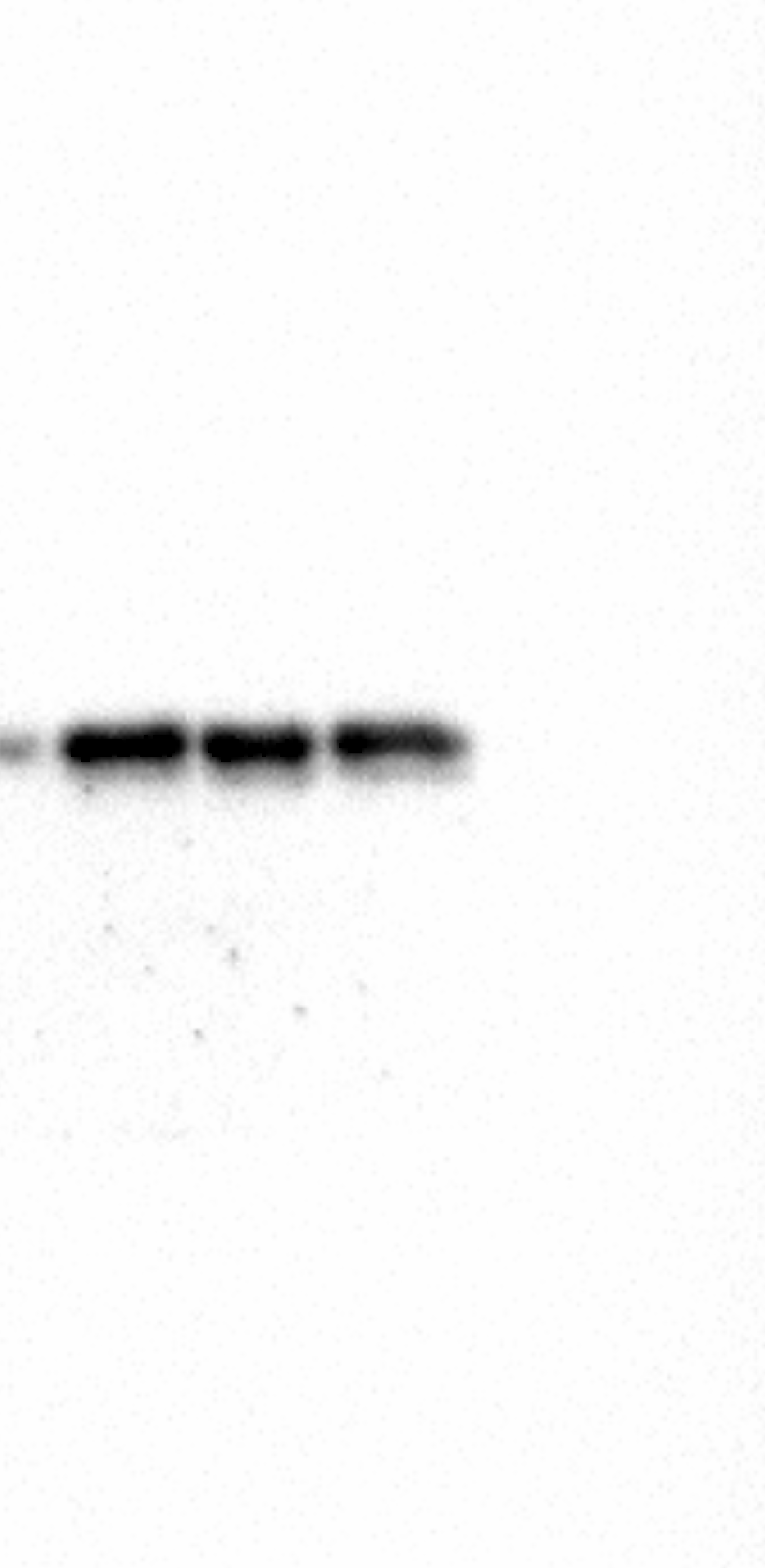

Supplement: Supplemental Information 27 [file peerj-14-21375-s027.zip › Figure 4D WB RAW oe-KLHL40 CAPZA/CAPZA-3 oe-KLHL40.tif]

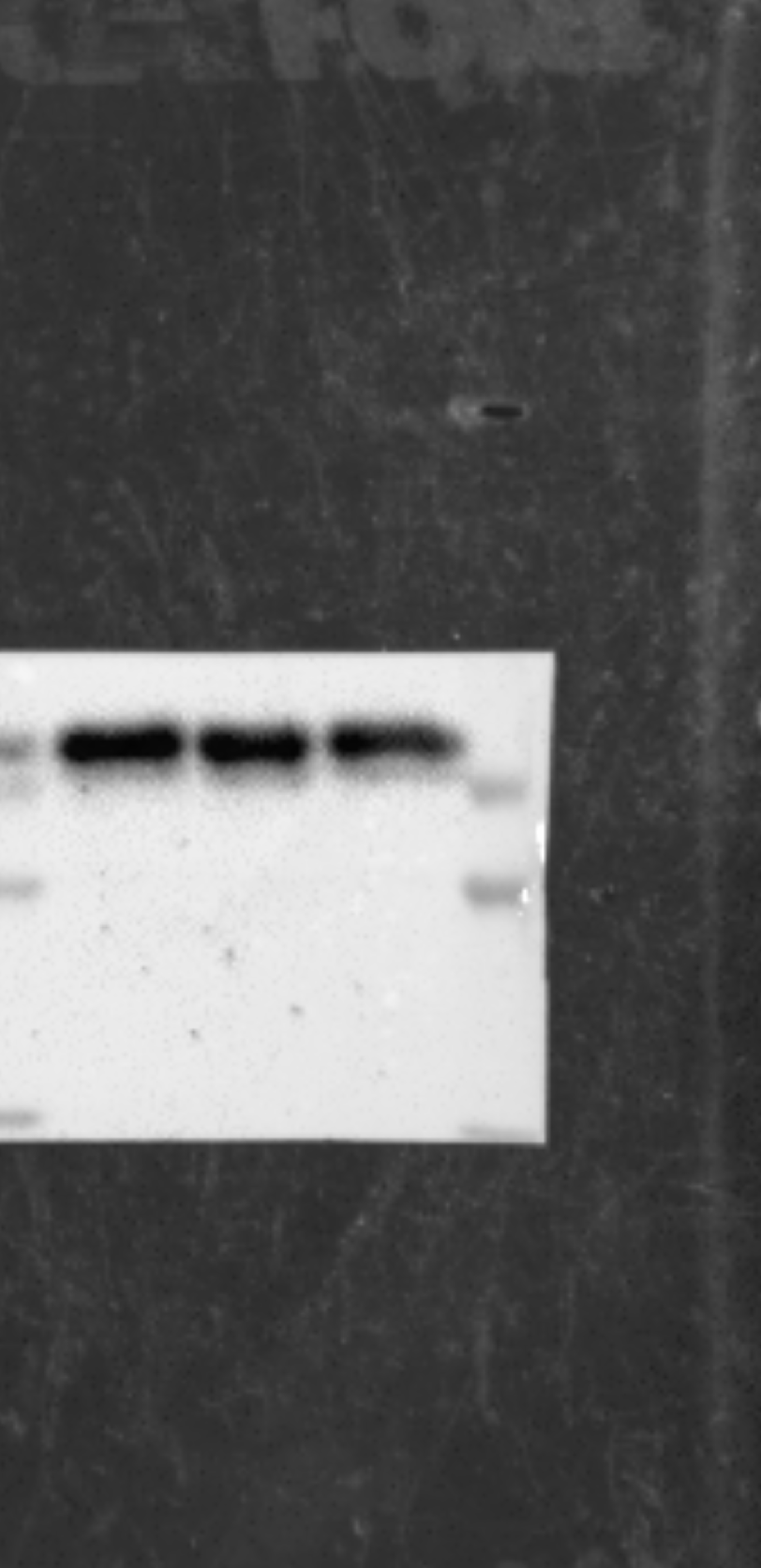

Supplement: Supplemental Information 27 [file peerj-14-21375-s027.zip › Figure 4D WB RAW oe-KLHL40 CAPZA/CAPZA-3 oe-KLHL40+MARK.tif]

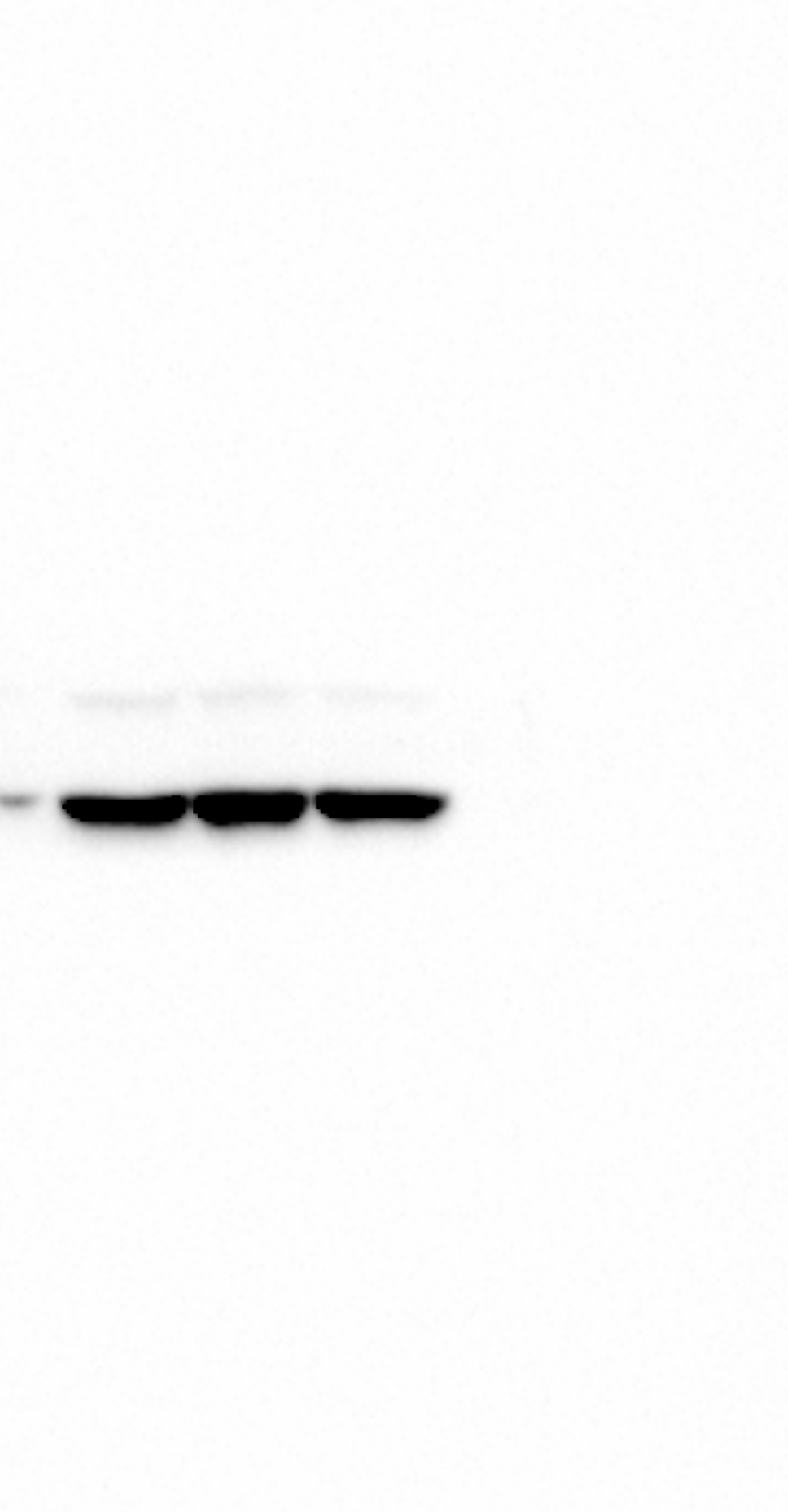

Supplement: Supplemental Information 27 [file peerj-14-21375-s027.zip › Figure 4D WB RAW oe-KLHL40 CAPZA/CAPZA-3 oe-KLHL40-ACTB.tif]

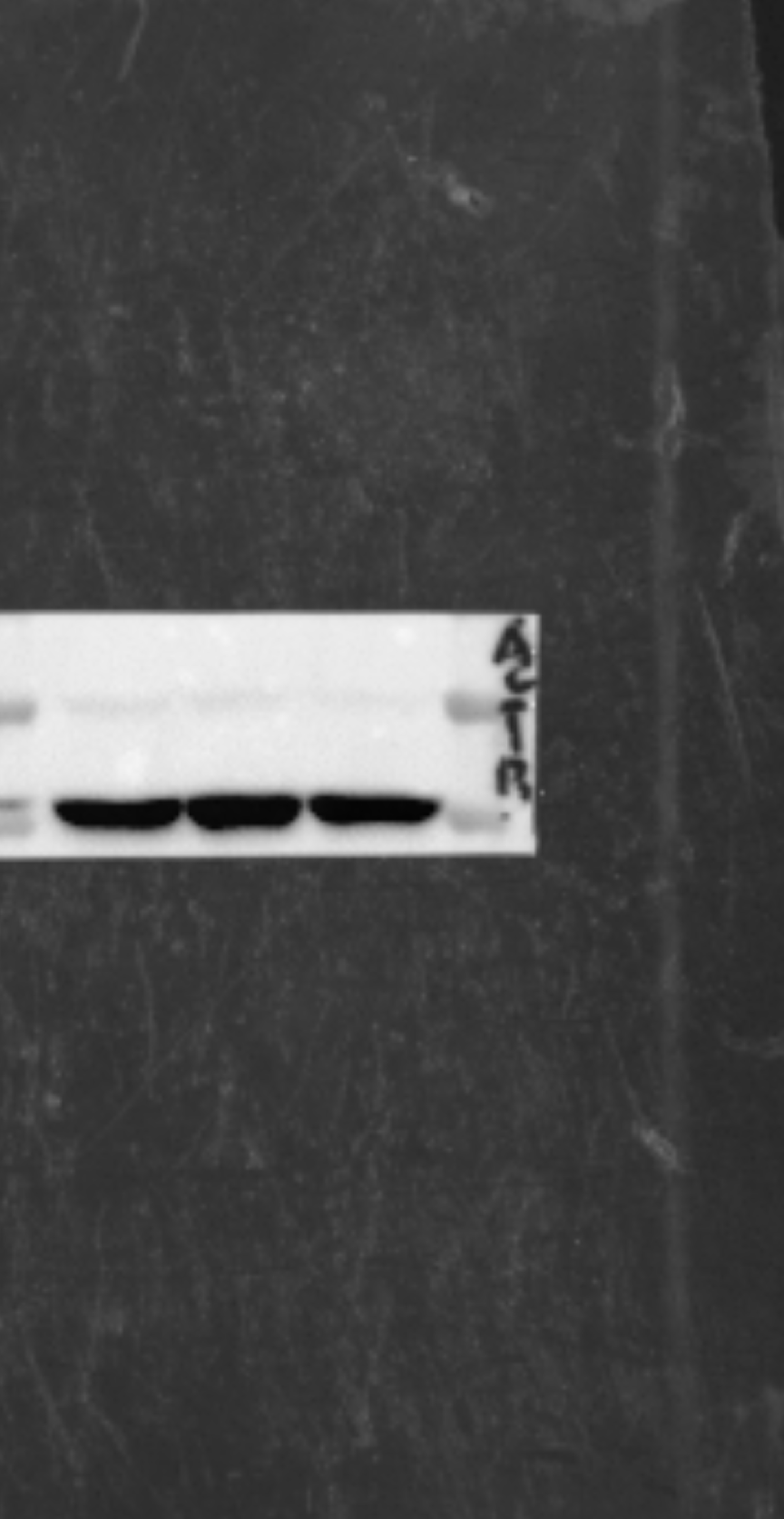

Supplement: Supplemental Information 27 [file peerj-14-21375-s027.zip › Figure 4D WB RAW oe-KLHL40 CAPZA/CAPZA-3 oe-KLHL40-ACTB+MARK.tif]

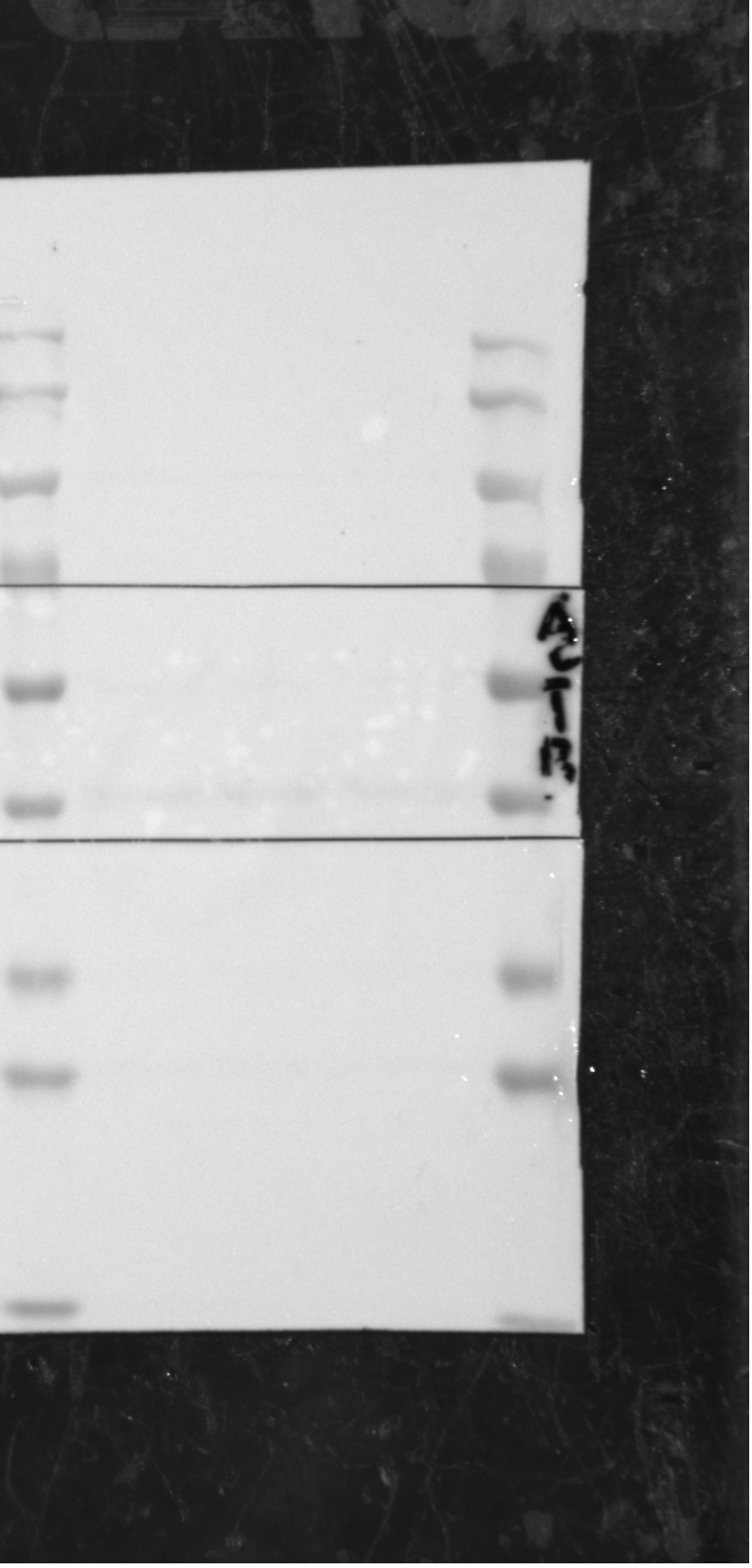

Supplement: Supplemental Information 27 [file peerj-14-21375-s027.zip › Figure 4D WB RAW oe-KLHL40 CAPZA/TOTAL-3.jpg]

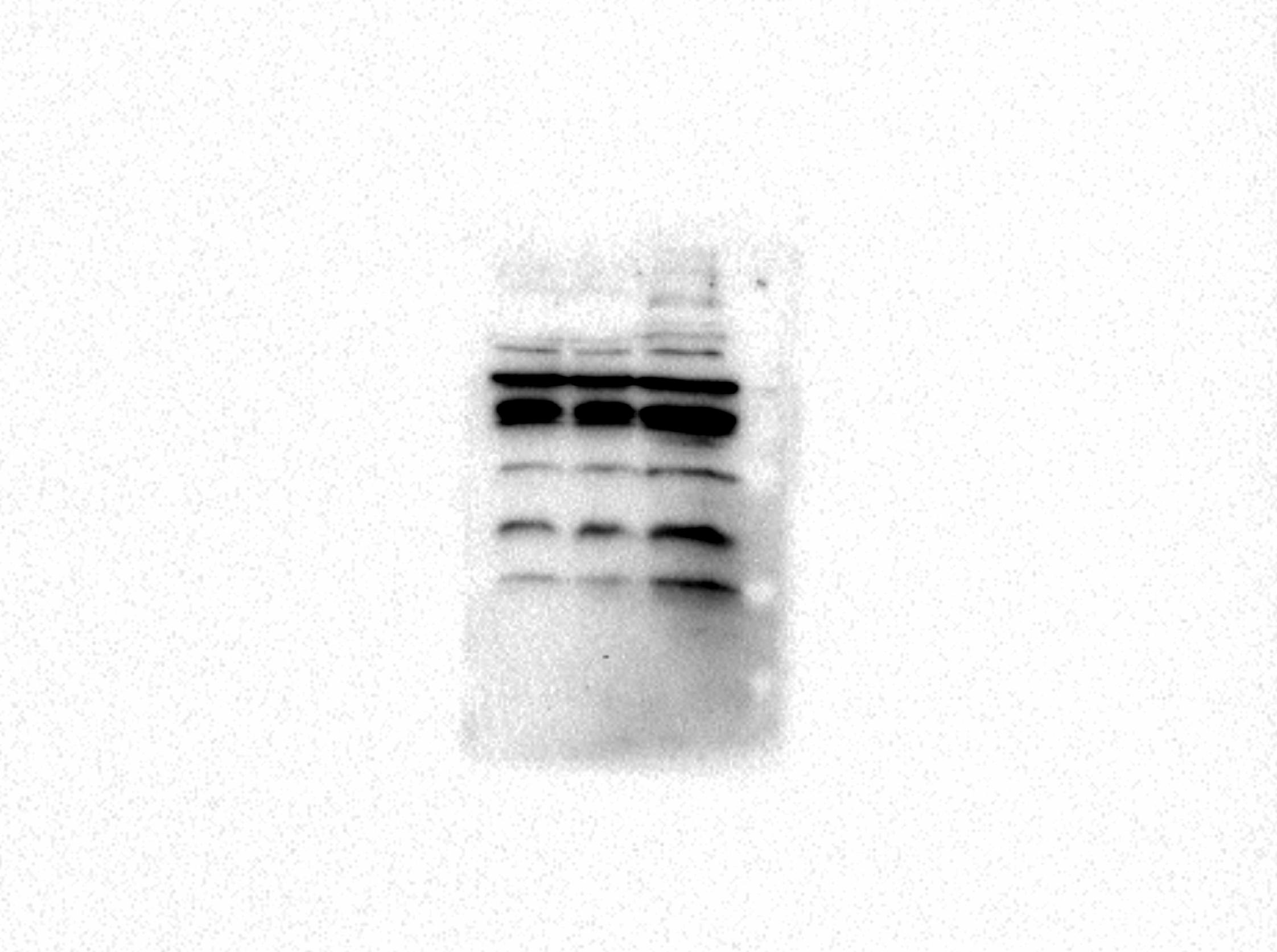

Supplement: Supplemental Information 28 [file peerj-14-21375-s028.zip › Figure 4E WB RAW sh-KLHL40 TCAP/TCAP-1 sh-KLHL40.tif]

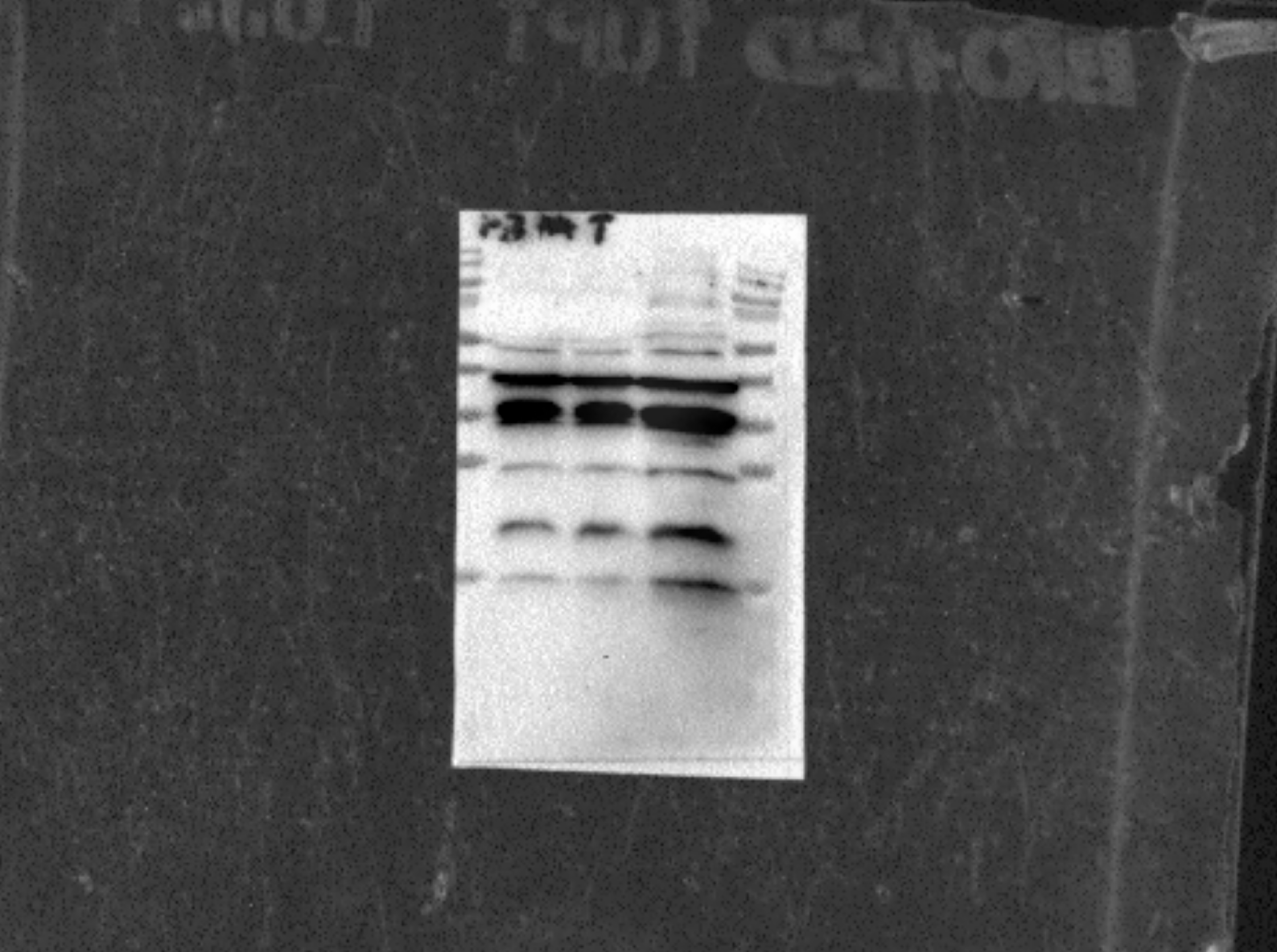

Supplement: Supplemental Information 28 [file peerj-14-21375-s028.zip › Figure 4E WB RAW sh-KLHL40 TCAP/TCAP-1 sh-KLHL40+MARK.tif]

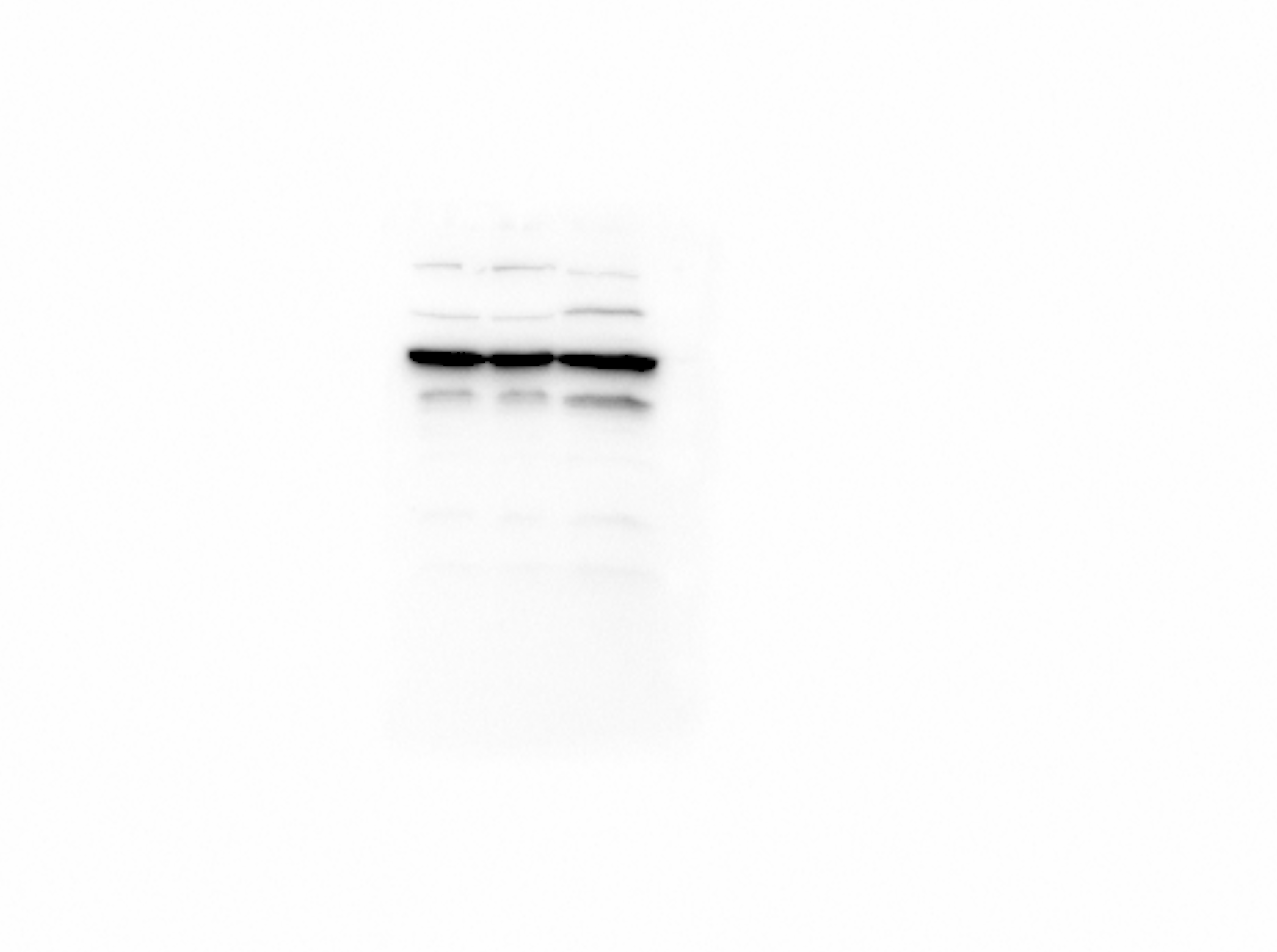

Supplement: Supplemental Information 28 [file peerj-14-21375-s028.zip › Figure 4E WB RAW sh-KLHL40 TCAP/TCAP-1 sh-KLHL40-ACTB.tif]

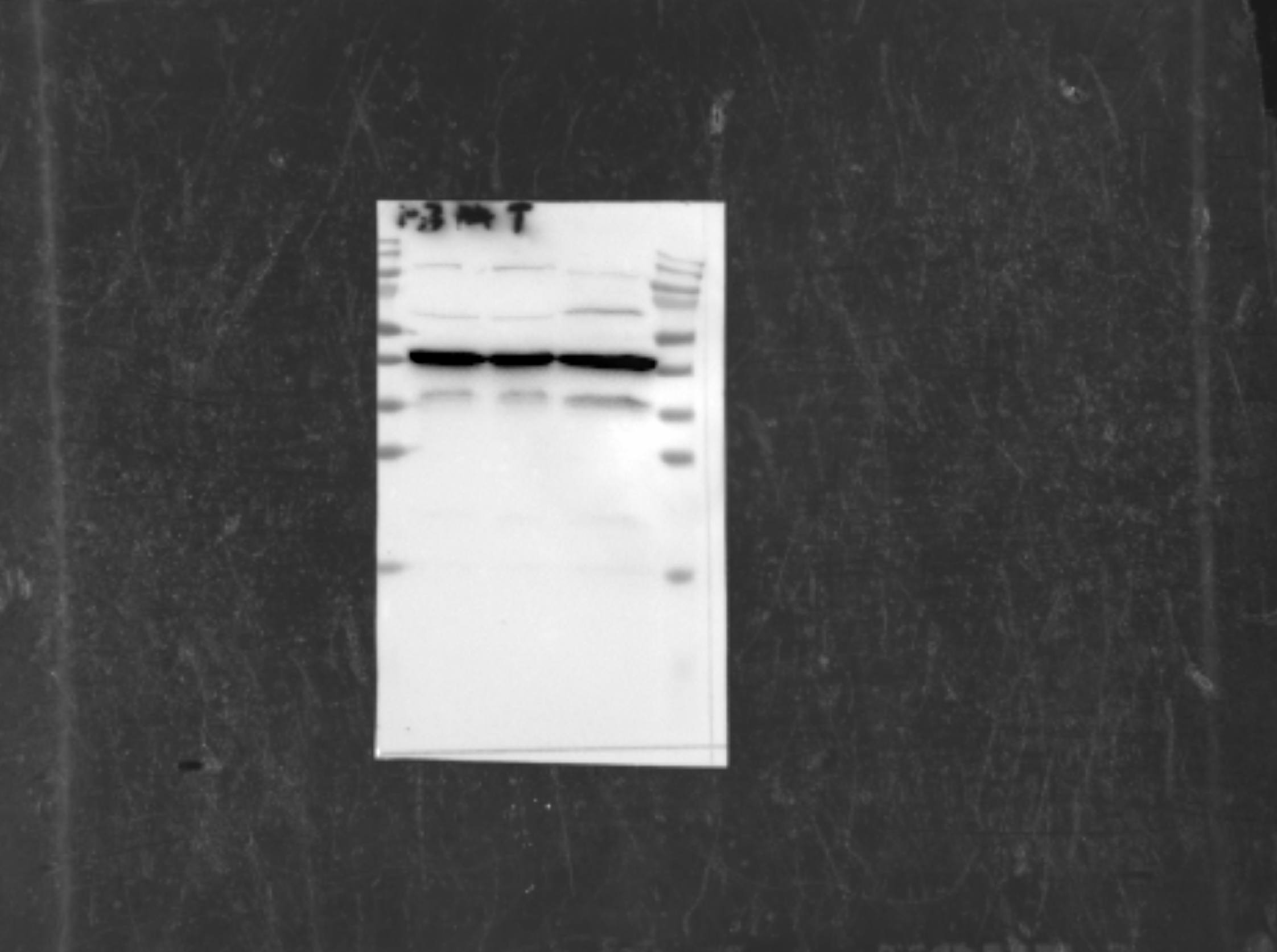

Supplement: Supplemental Information 28 [file peerj-14-21375-s028.zip › Figure 4E WB RAW sh-KLHL40 TCAP/TCAP-1 sh-KLHL40-ACTB+MARK.tif]

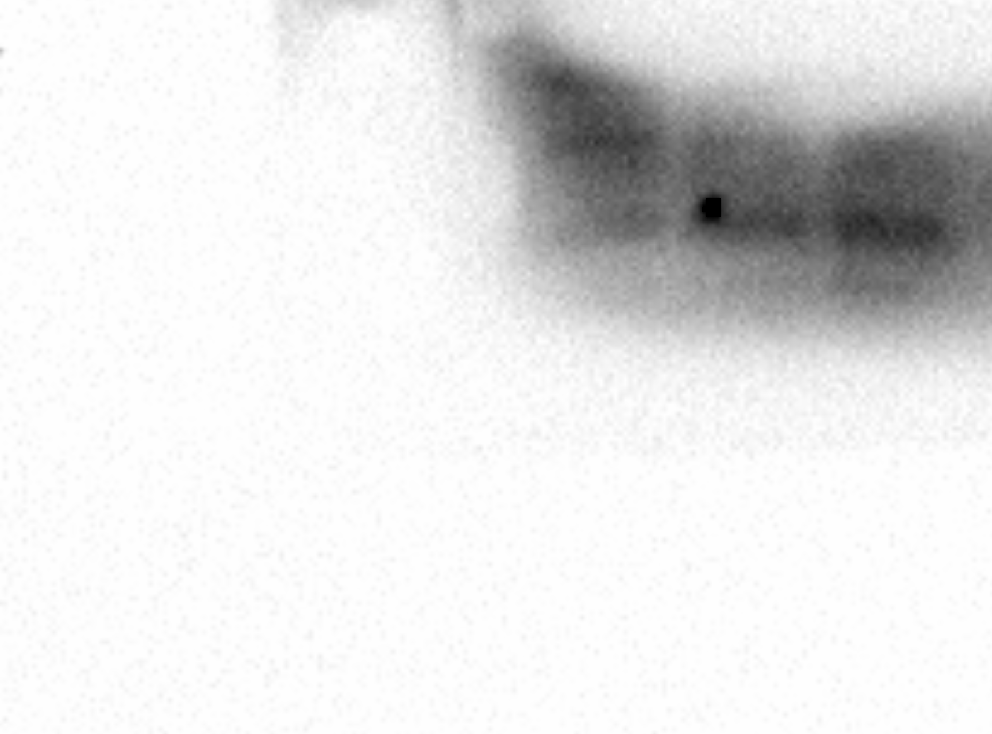

Supplement: Supplemental Information 28 [file peerj-14-21375-s028.zip › Figure 4E WB RAW sh-KLHL40 TCAP/TCAP-2 sh-KLHL40.tif]

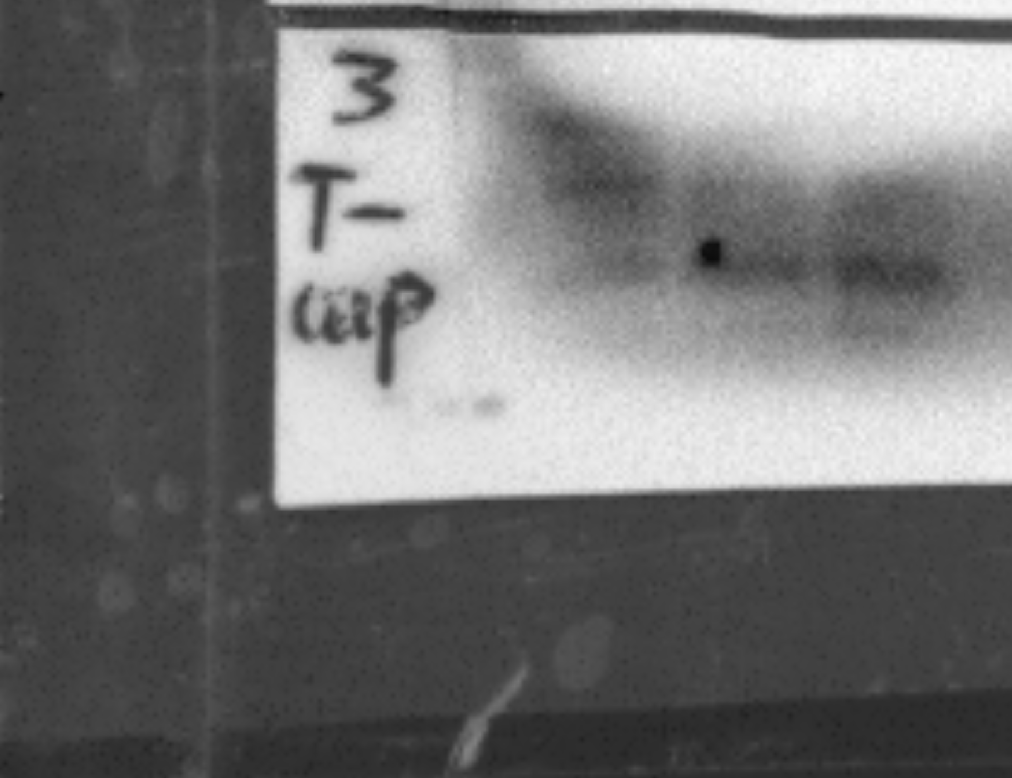

Supplement: Supplemental Information 28 [file peerj-14-21375-s028.zip › Figure 4E WB RAW sh-KLHL40 TCAP/TCAP-2 sh-KLHL40+MARK.tif]

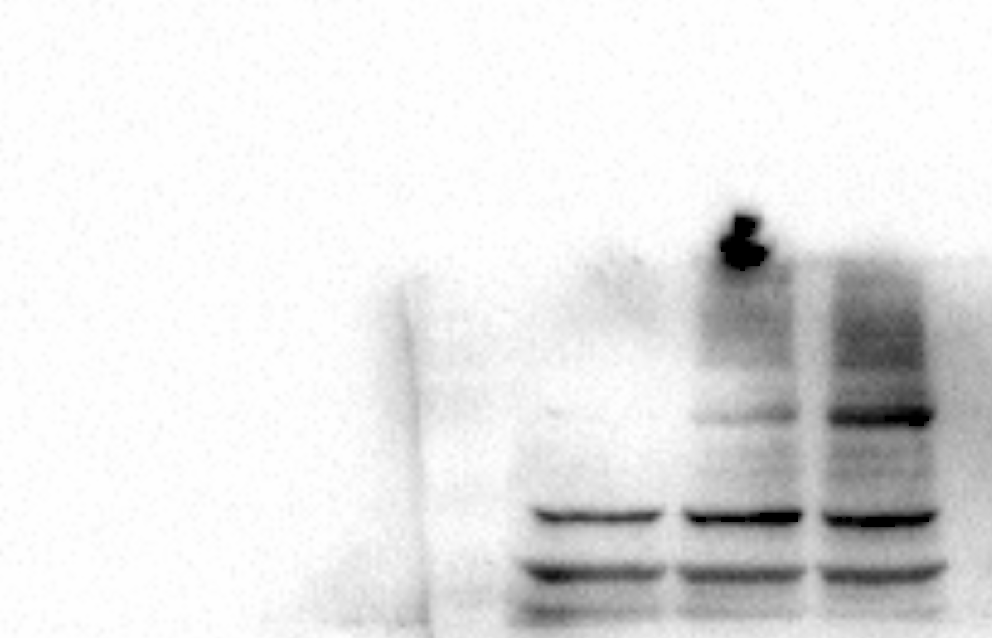

Supplement: Supplemental Information 28 [file peerj-14-21375-s028.zip › Figure 4E WB RAW sh-KLHL40 TCAP/TCAP-2 sh-KLHL40-ACTB.tif]

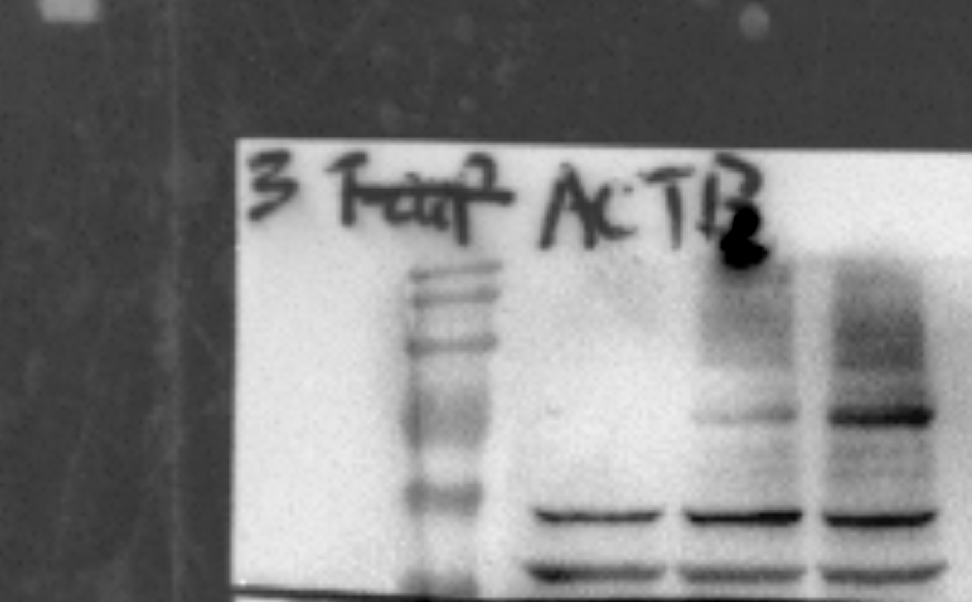

Supplement: Supplemental Information 28 [file peerj-14-21375-s028.zip › Figure 4E WB RAW sh-KLHL40 TCAP/TCAP-2-sh-KLHL40-ACTB+MARK.tif]

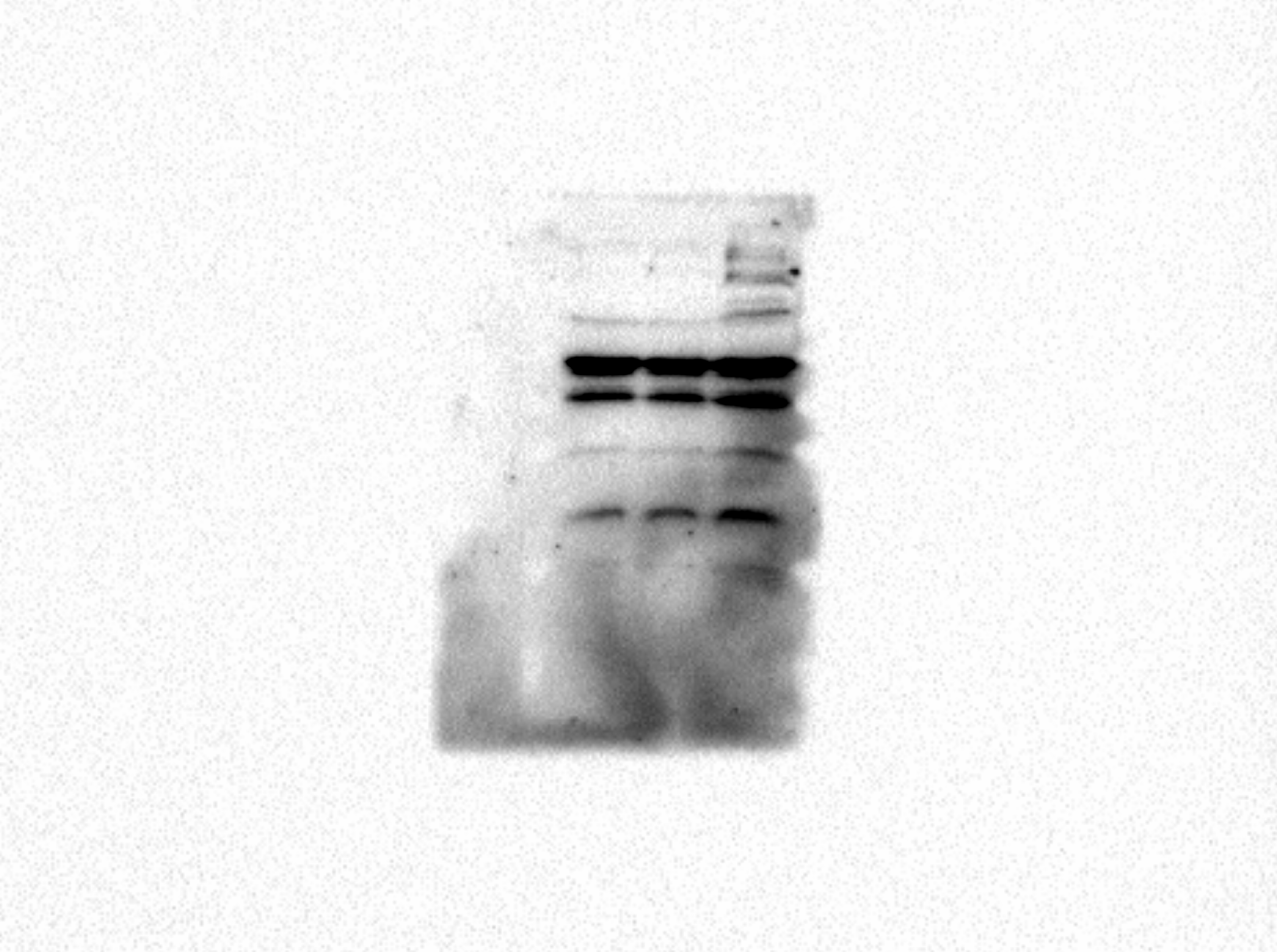

Supplement: Supplemental Information 28 [file peerj-14-21375-s028.zip › Figure 4E WB RAW sh-KLHL40 TCAP/TCAP-3 sh-KLHL40.tif]

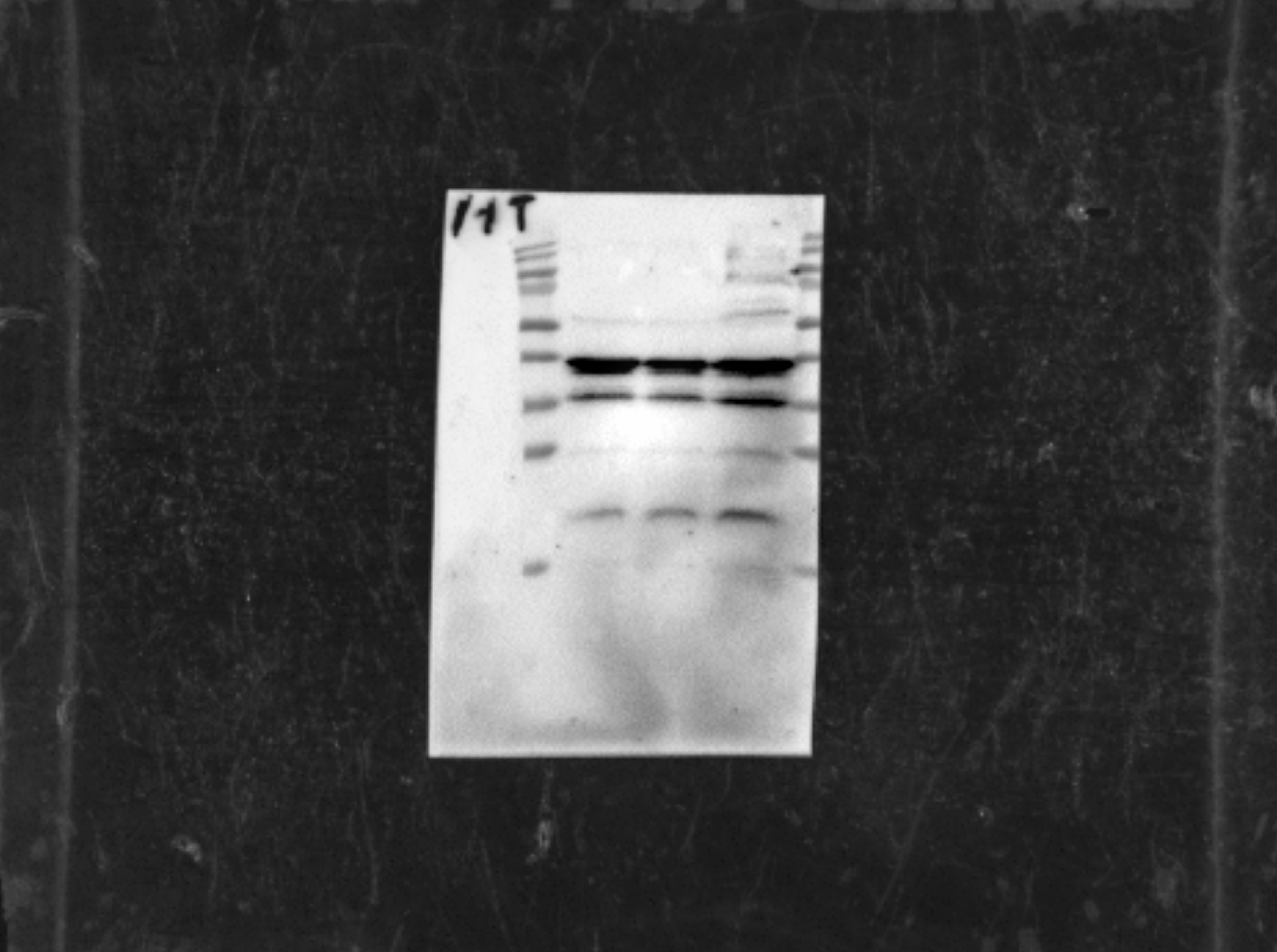

Supplement: Supplemental Information 28 [file peerj-14-21375-s028.zip › Figure 4E WB RAW sh-KLHL40 TCAP/TCAP-3 sh-KLHL40+MARK.tif]

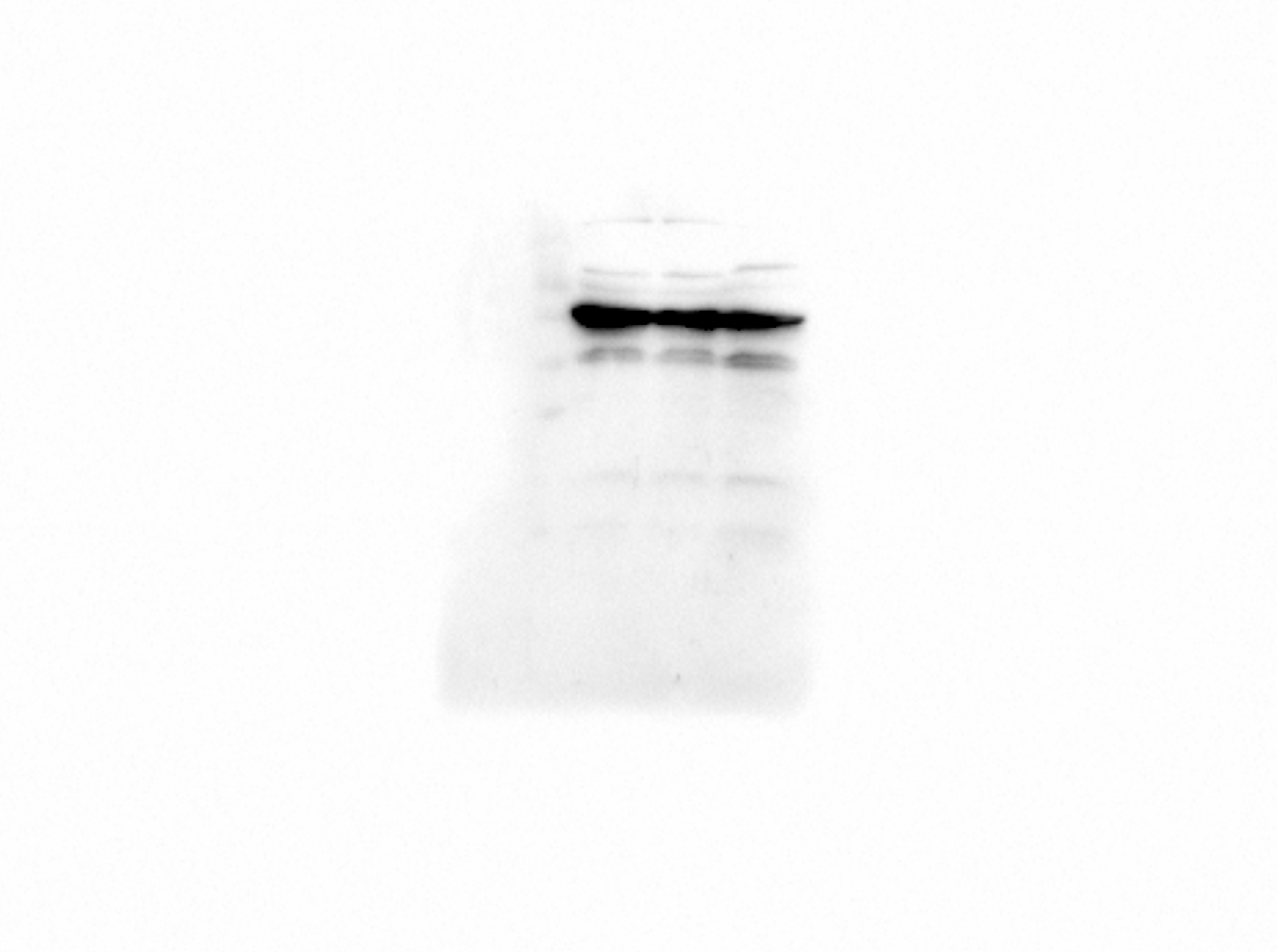

Supplement: Supplemental Information 28 [file peerj-14-21375-s028.zip › Figure 4E WB RAW sh-KLHL40 TCAP/TCAP-3 sh-KLHL40-ACTB.tif]

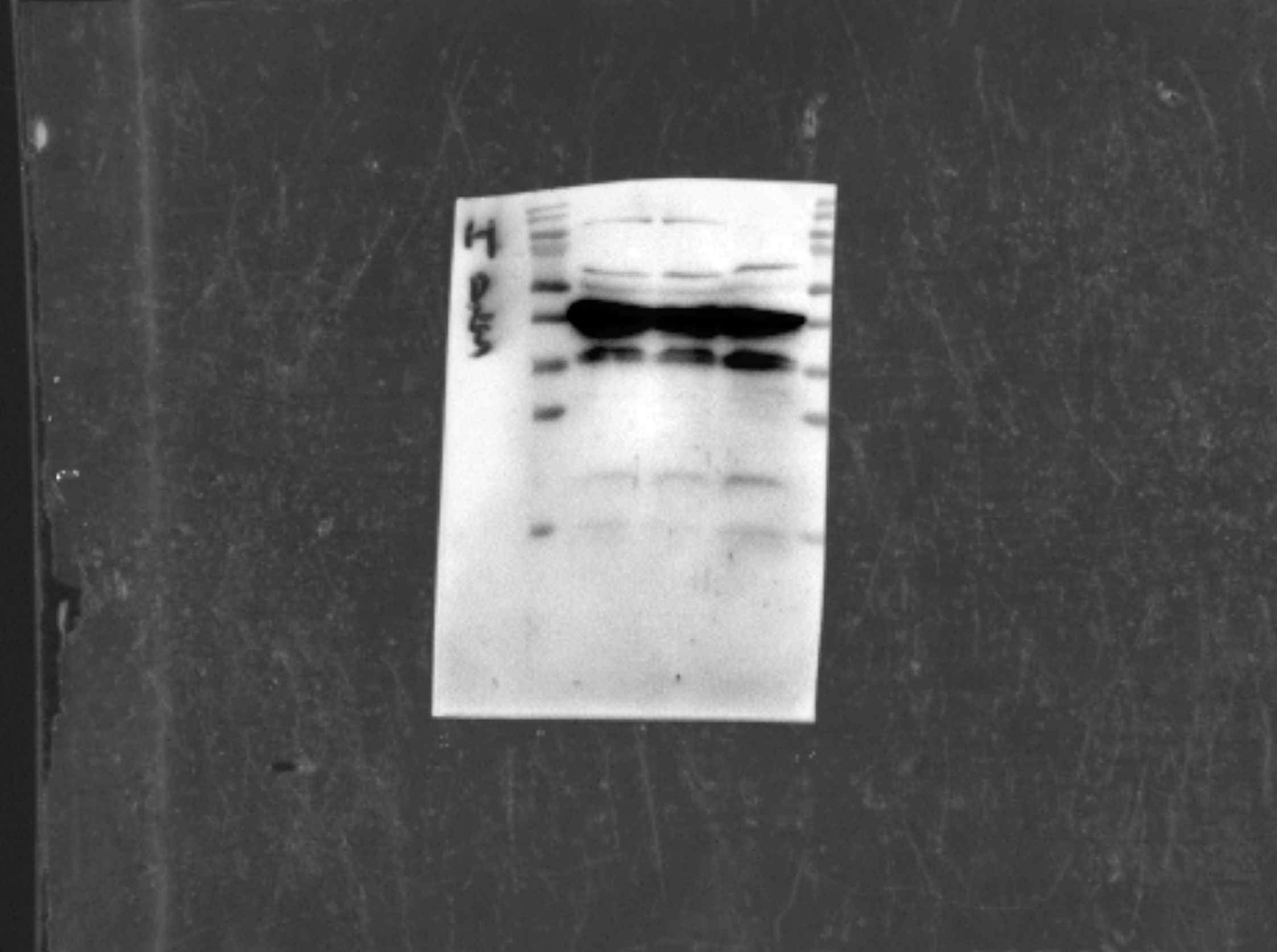

Supplement: Supplemental Information 28 [file peerj-14-21375-s028.zip › Figure 4E WB RAW sh-KLHL40 TCAP/TCAP-3-sh-KLHL40-ACTB+MARK.tif]

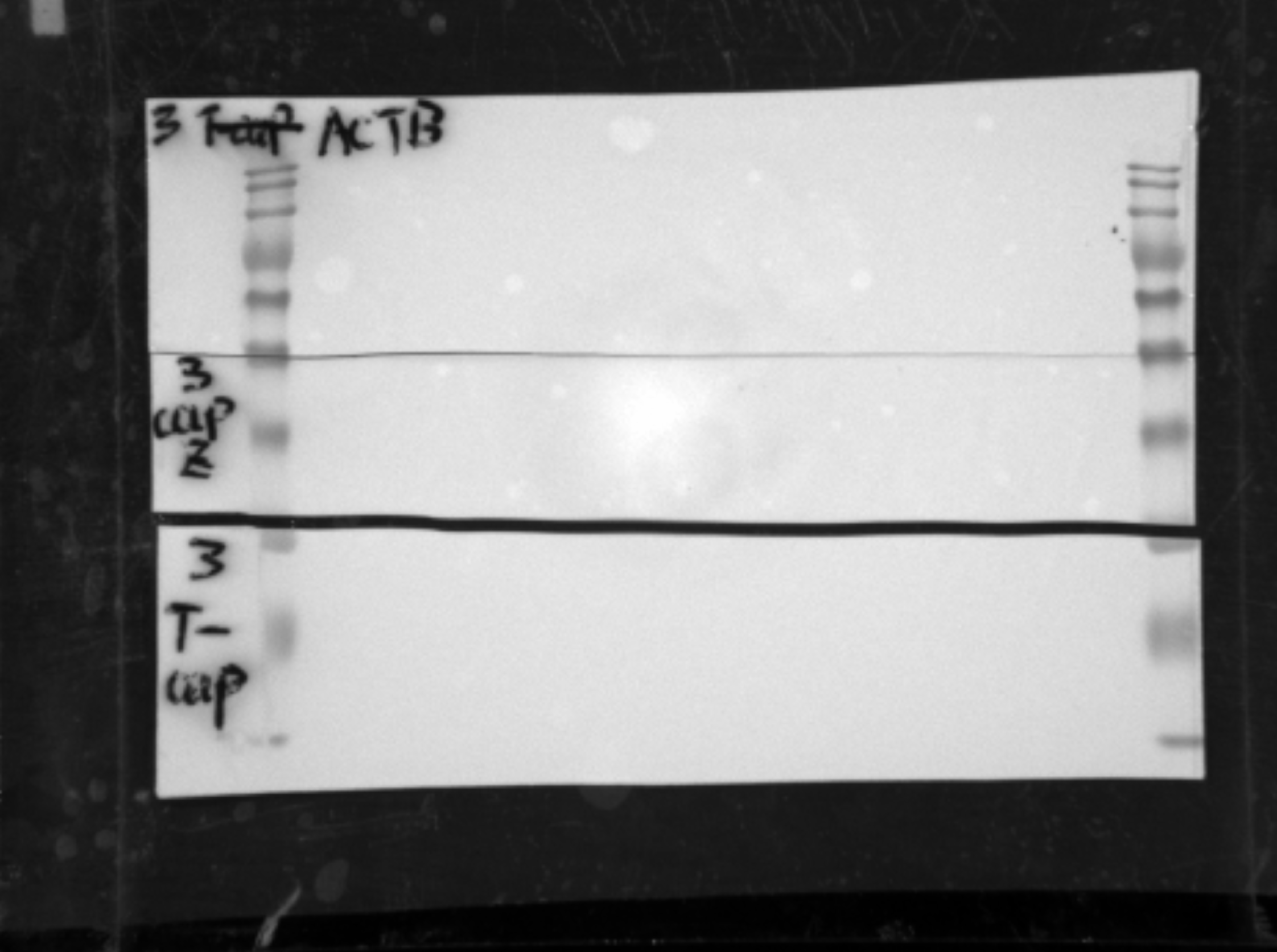

Supplement: Supplemental Information 28 [file peerj-14-21375-s028.zip › Figure 4E WB RAW sh-KLHL40 TCAP/TOTAL-2.tif]

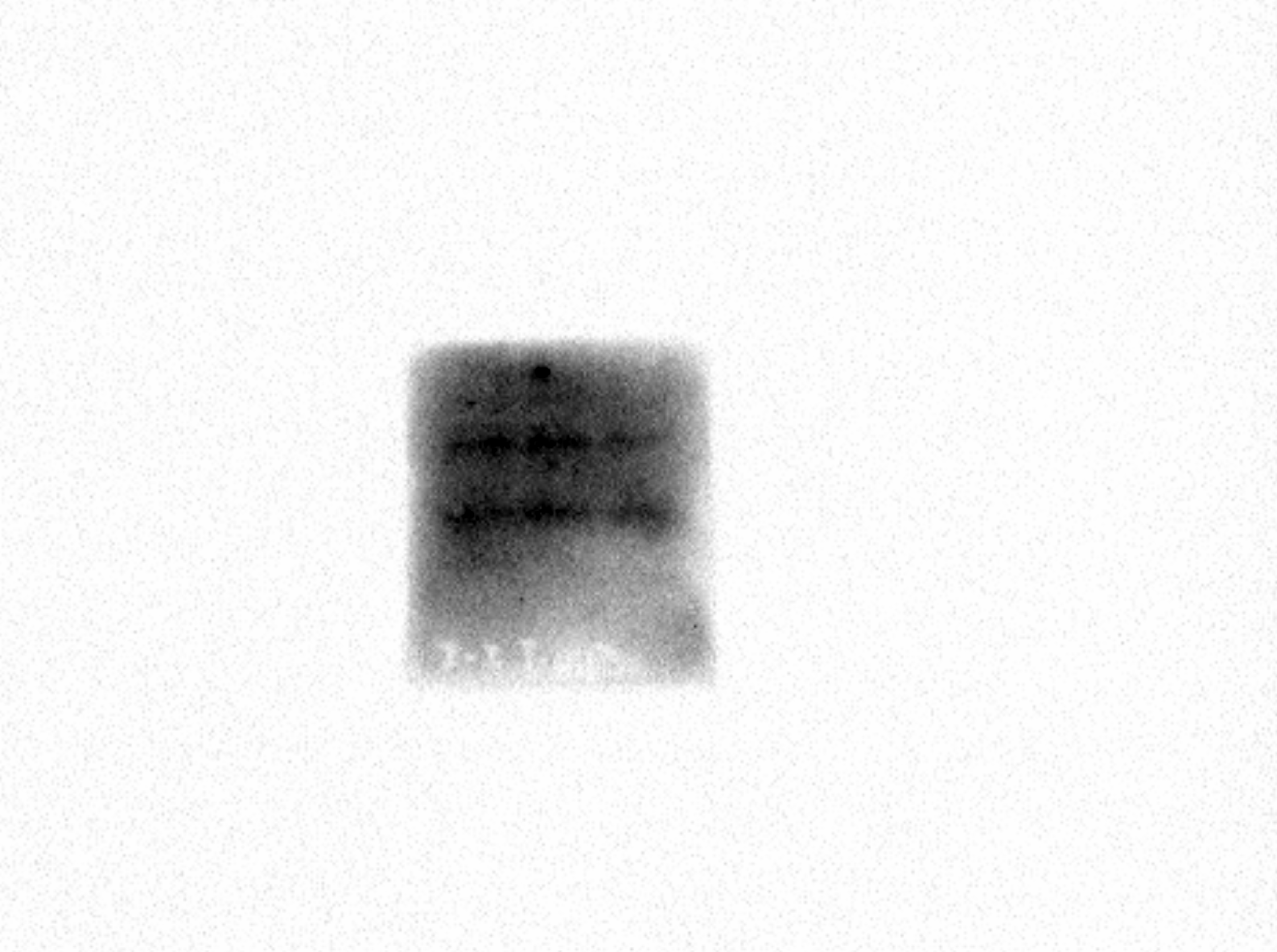

Supplement: Supplemental Information 29 [file peerj-14-21375-s029.zip › Figure 4F WB RAW oe-KLHL40 TCAP/TCAP-1 oe-KLHL40.tif]

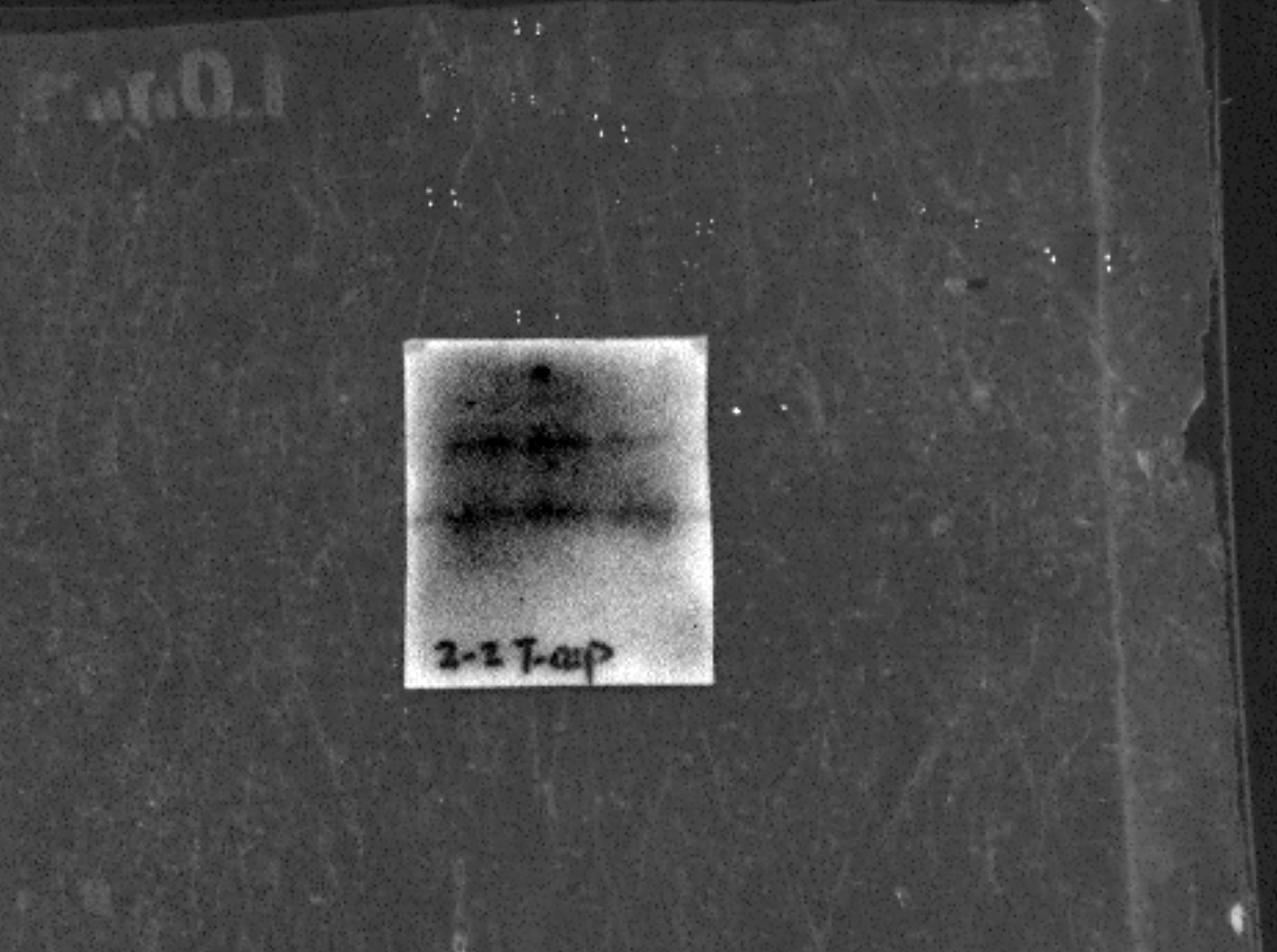

Supplement: Supplemental Information 29 [file peerj-14-21375-s029.zip › Figure 4F WB RAW oe-KLHL40 TCAP/TCAP-1 oe-KLHL40+MARK.tif]

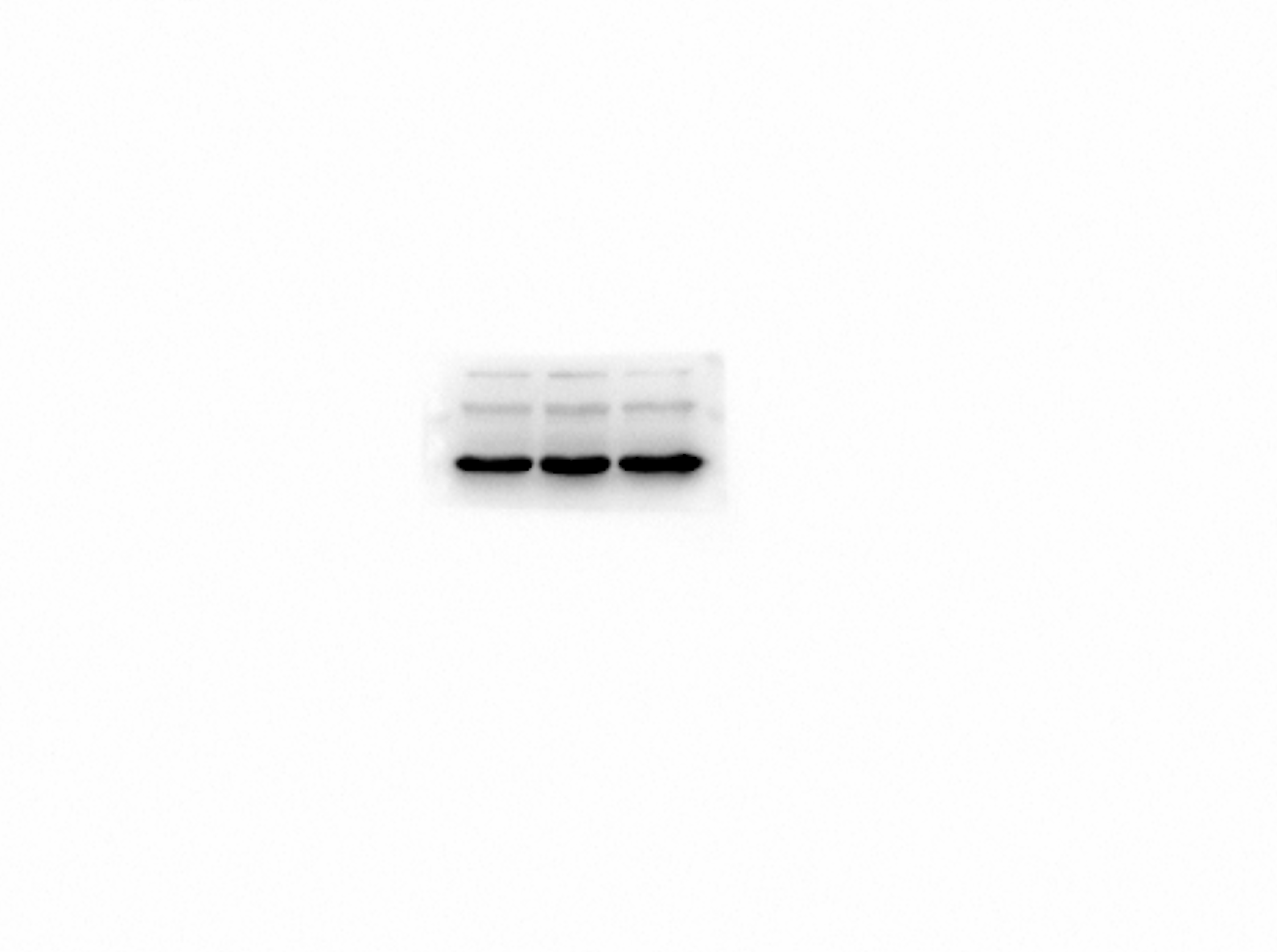

Supplement: Supplemental Information 29 [file peerj-14-21375-s029.zip › Figure 4F WB RAW oe-KLHL40 TCAP/TCAP-1 oe-KLHL40-ACTB.tif]

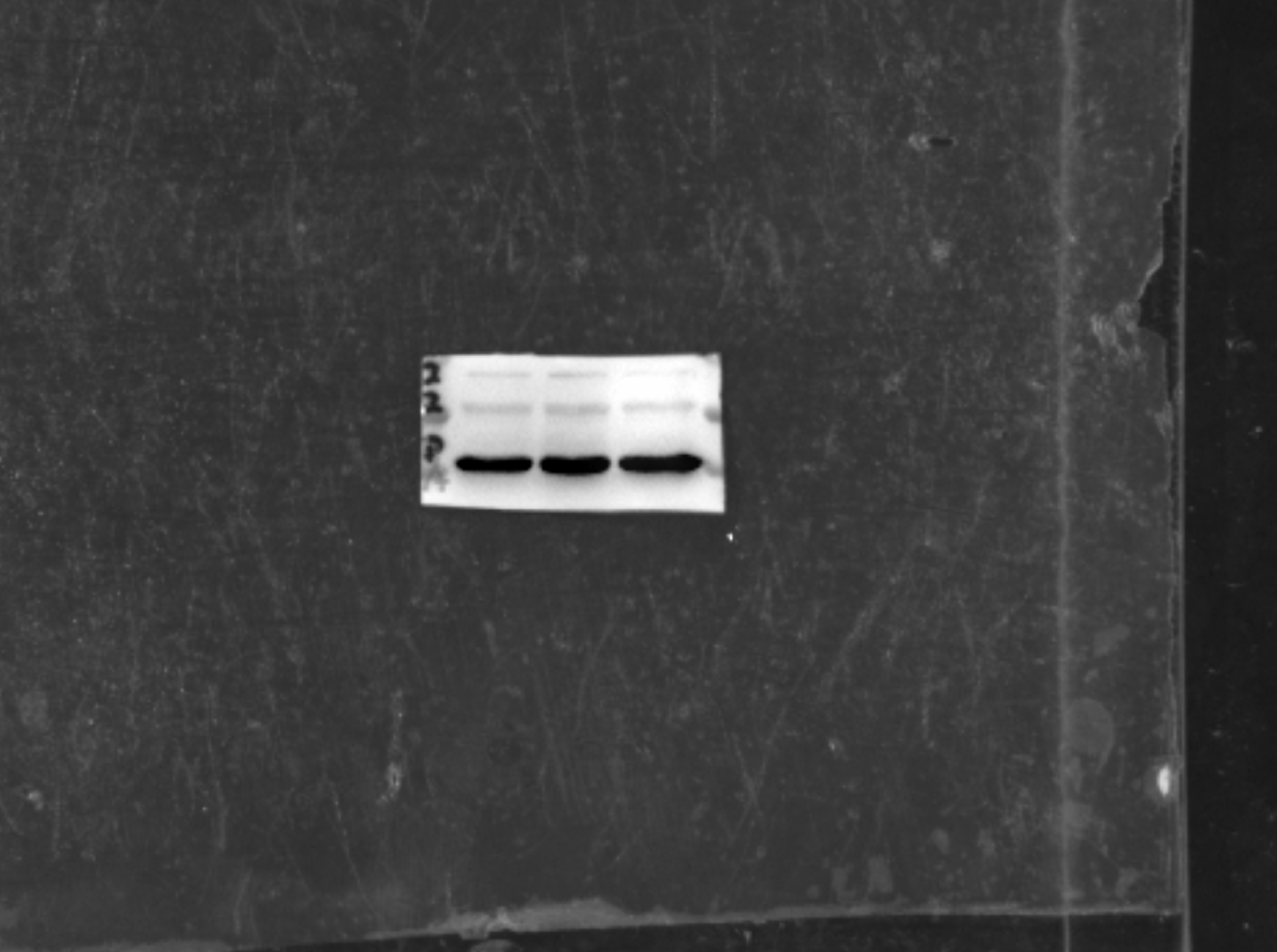

Supplement: Supplemental Information 29 [file peerj-14-21375-s029.zip › Figure 4F WB RAW oe-KLHL40 TCAP/TCAP-1 oe-KLHL40-ACTB+MARK.tif]

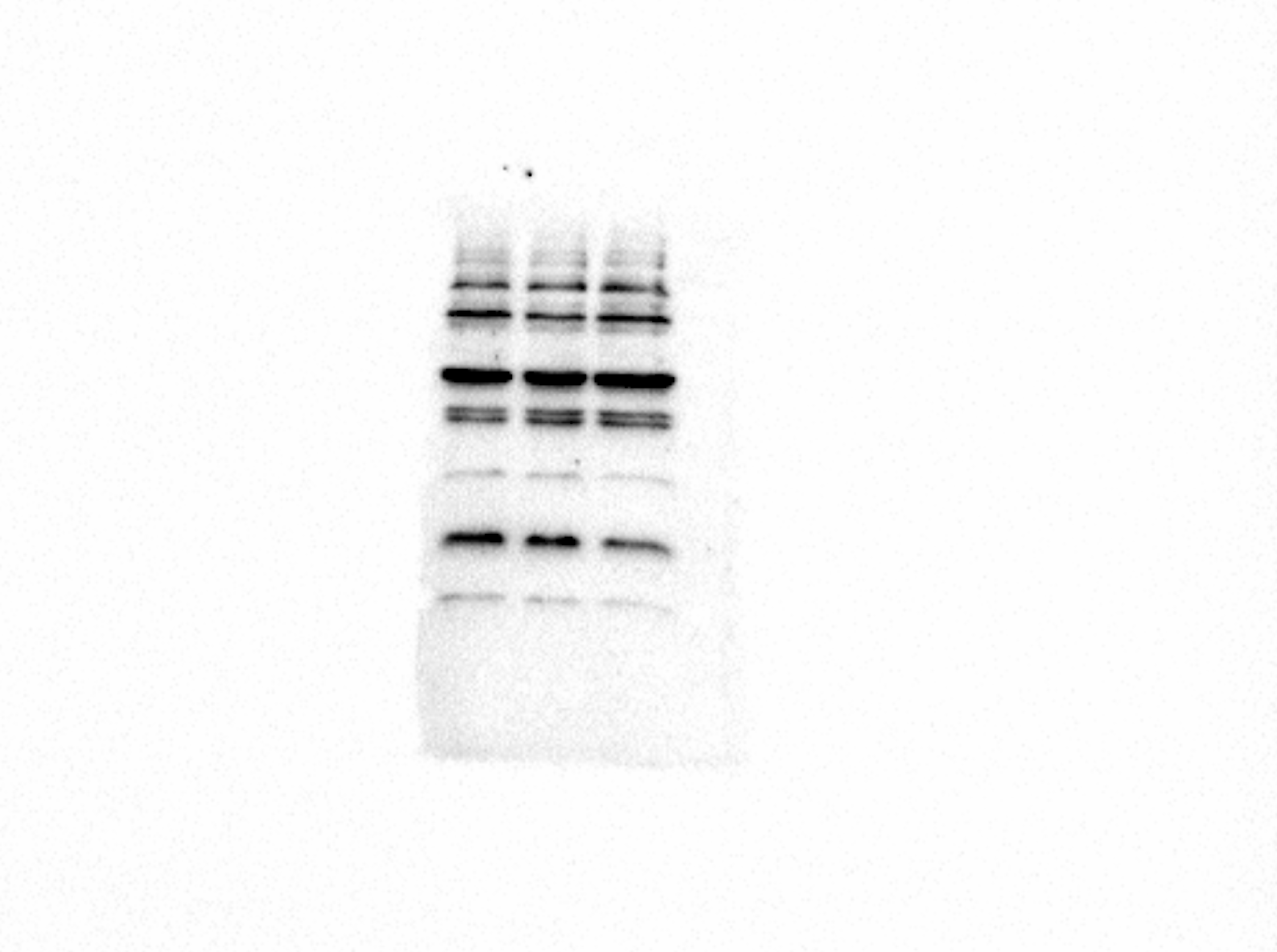

Supplement: Supplemental Information 29 [file peerj-14-21375-s029.zip › Figure 4F WB RAW oe-KLHL40 TCAP/TCAP-2 oe-KLHL40+ACTB.tif]

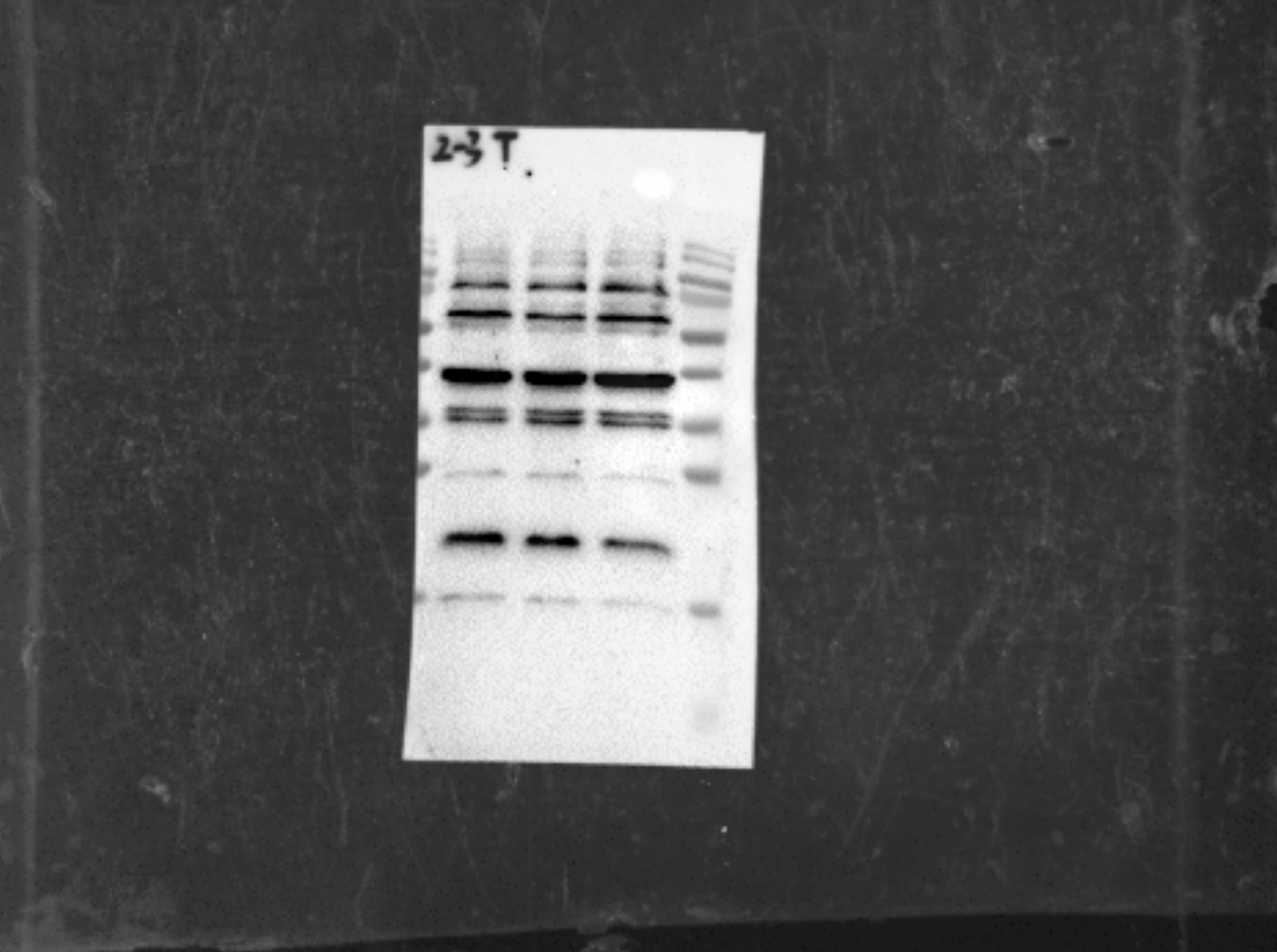

Supplement: Supplemental Information 29 [file peerj-14-21375-s029.zip › Figure 4F WB RAW oe-KLHL40 TCAP/TCAP-2 oe-KLHL40+ACTB+MARK.tif]

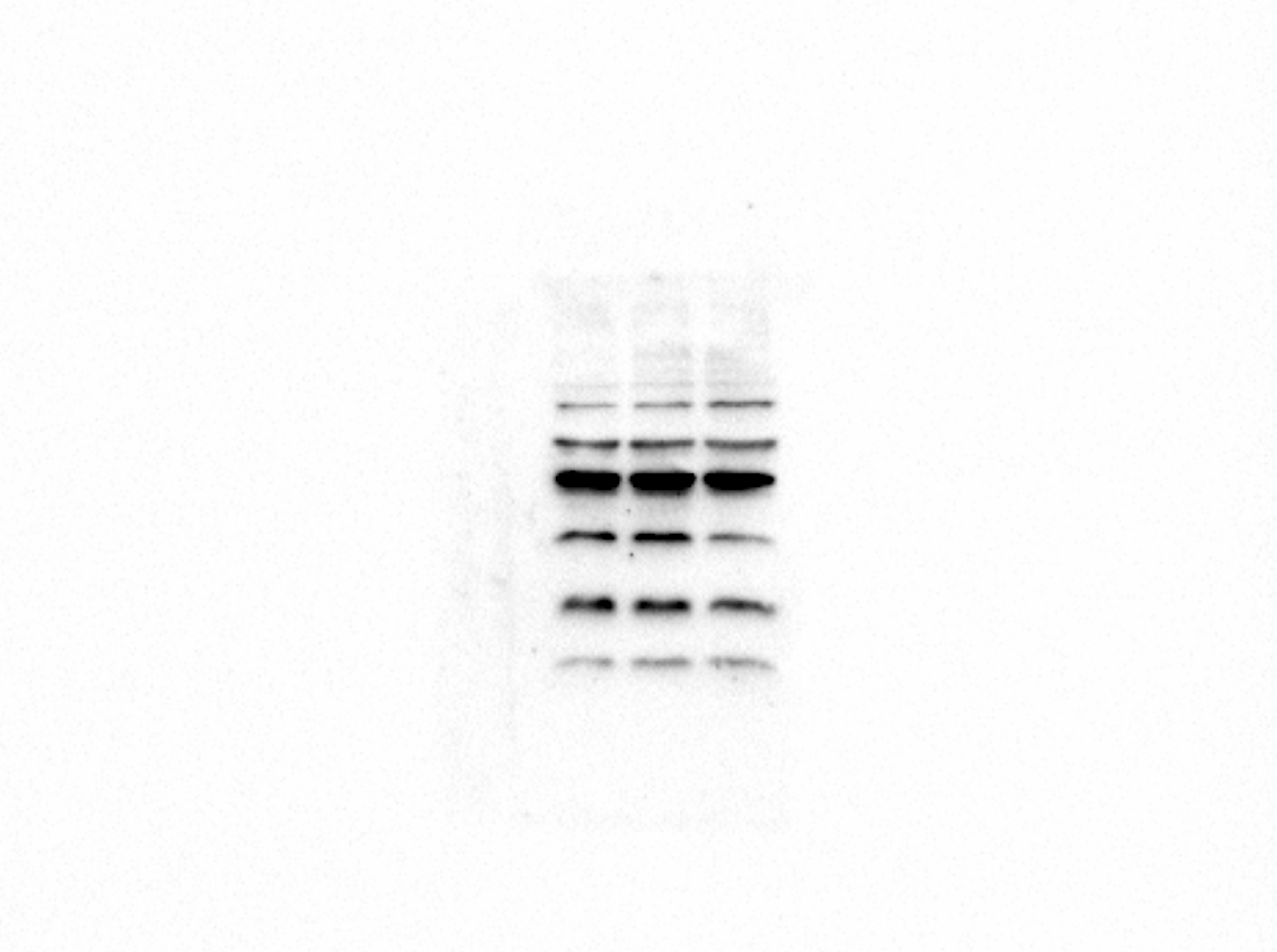

Supplement: Supplemental Information 29 [file peerj-14-21375-s029.zip › Figure 4F WB RAW oe-KLHL40 TCAP/TCAP-3 oe-KLHL40.tif]

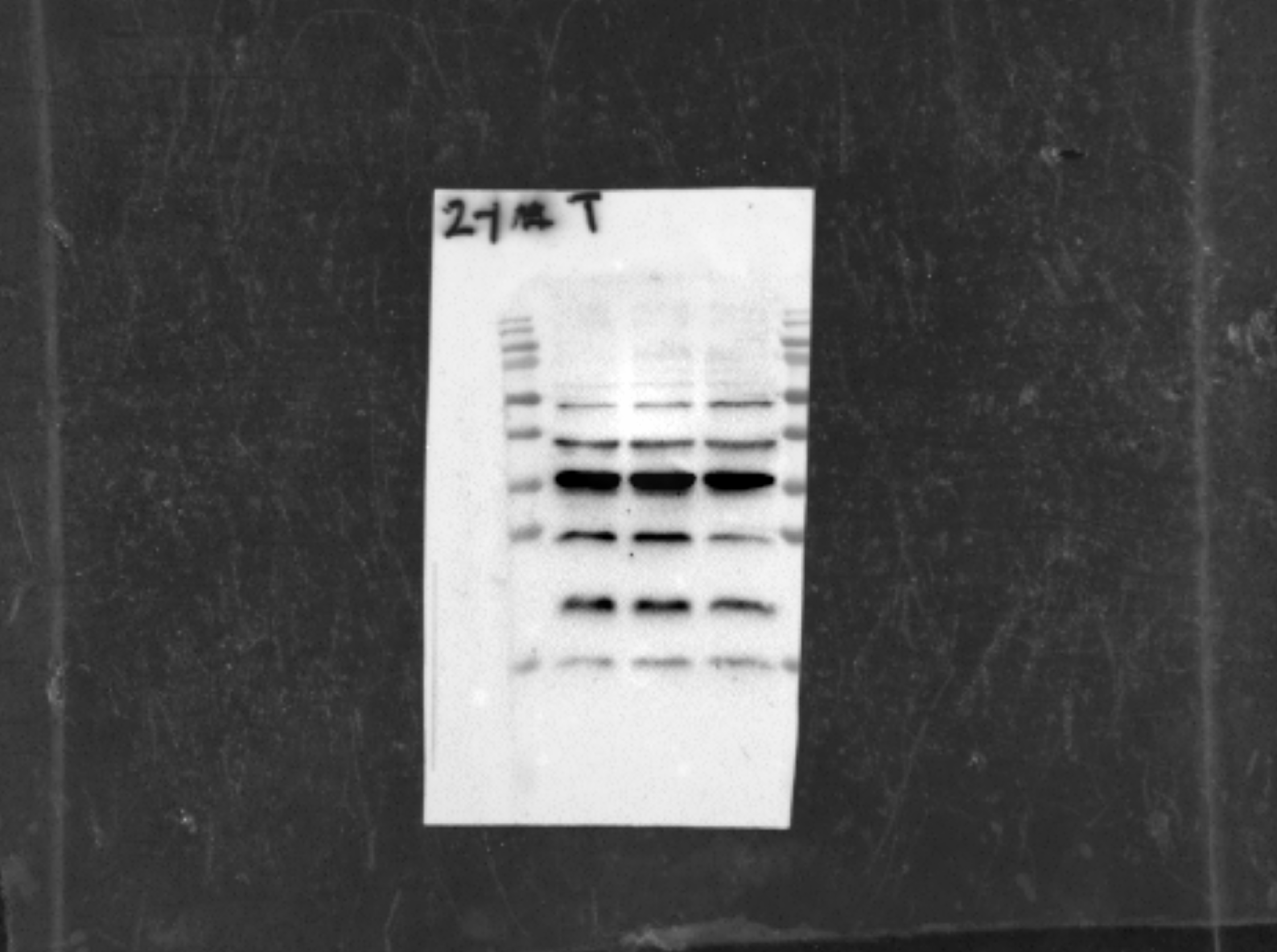

Supplement: Supplemental Information 29 [file peerj-14-21375-s029.zip › Figure 4F WB RAW oe-KLHL40 TCAP/TCAP-3 oe-KLHL40+MARK.tif]

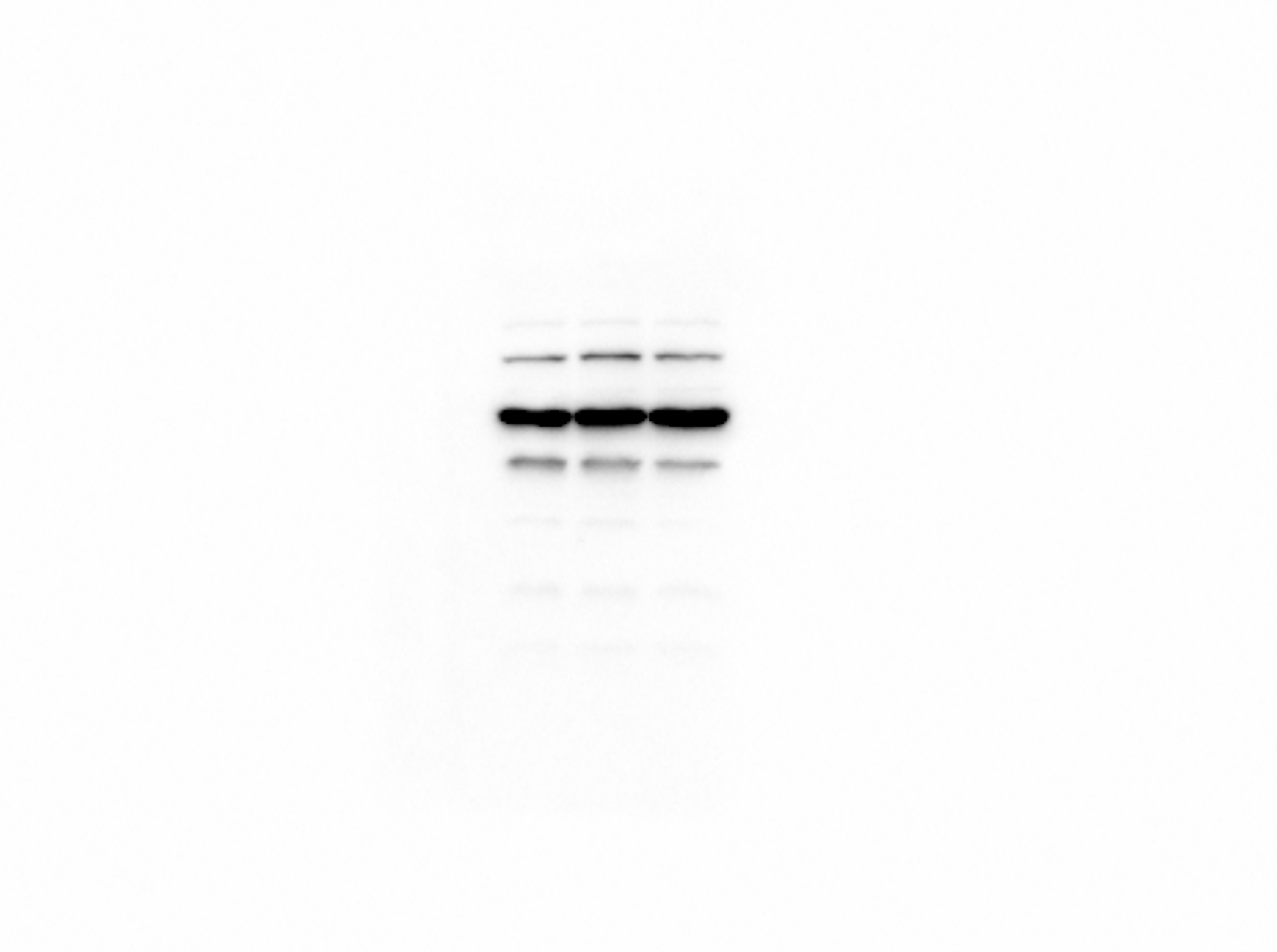

Supplement: Supplemental Information 29 [file peerj-14-21375-s029.zip › Figure 4F WB RAW oe-KLHL40 TCAP/TCAP-3 oe-KLHL40-ACTB.tif]

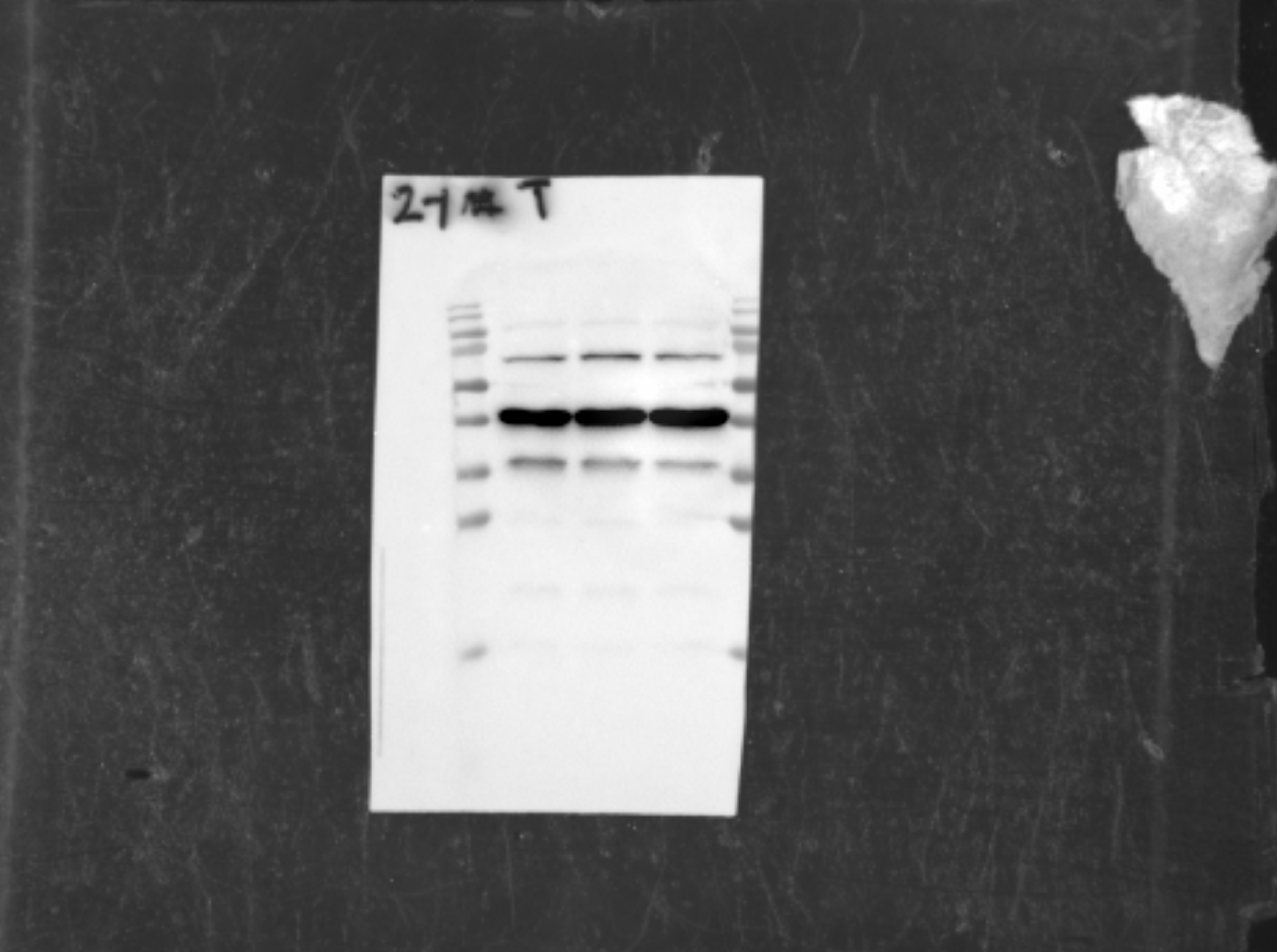

Supplement: Supplemental Information 29 [file peerj-14-21375-s029.zip › Figure 4F WB RAW oe-KLHL40 TCAP/TCAP-3 oe-KLHL40-ACTB+MARK+.tif]

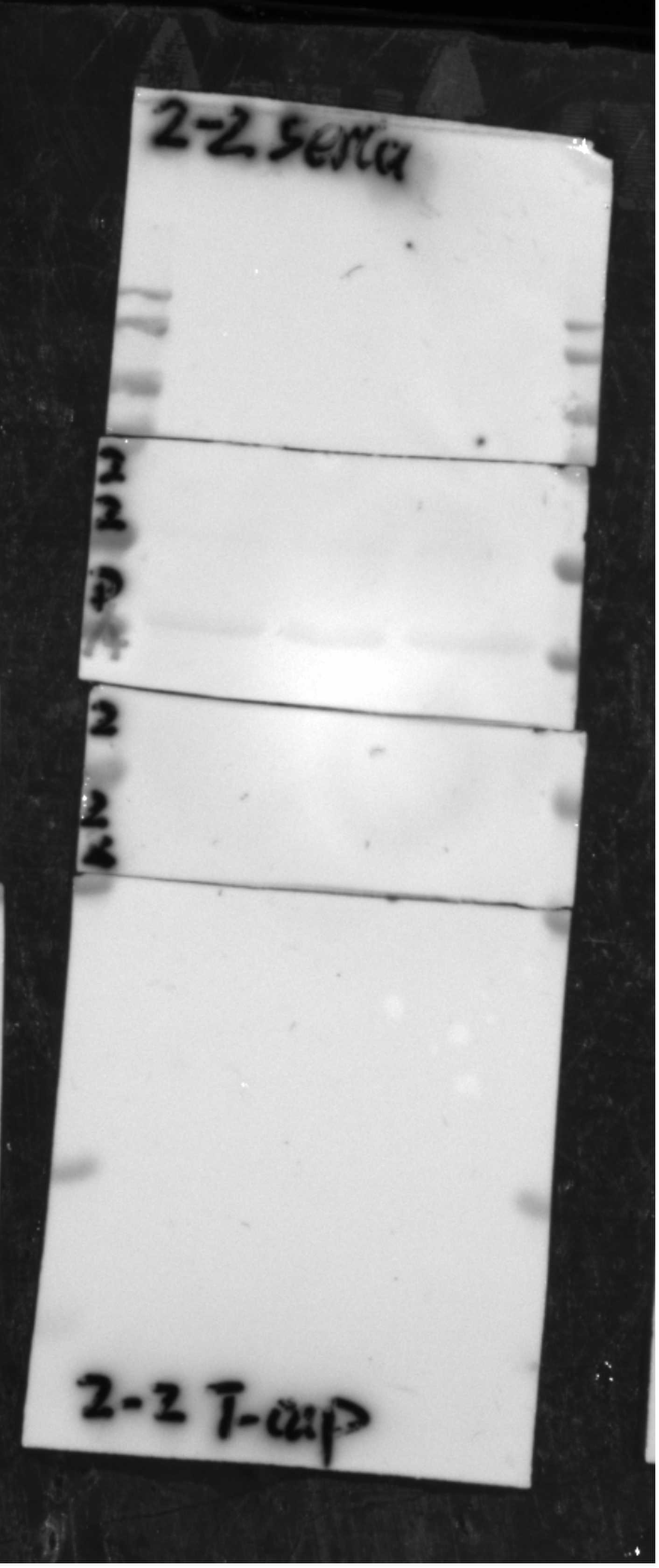

Supplement: Supplemental Information 29 [file peerj-14-21375-s029.zip › Figure 4F WB RAW oe-KLHL40 TCAP/TOTAL-1.jpg]

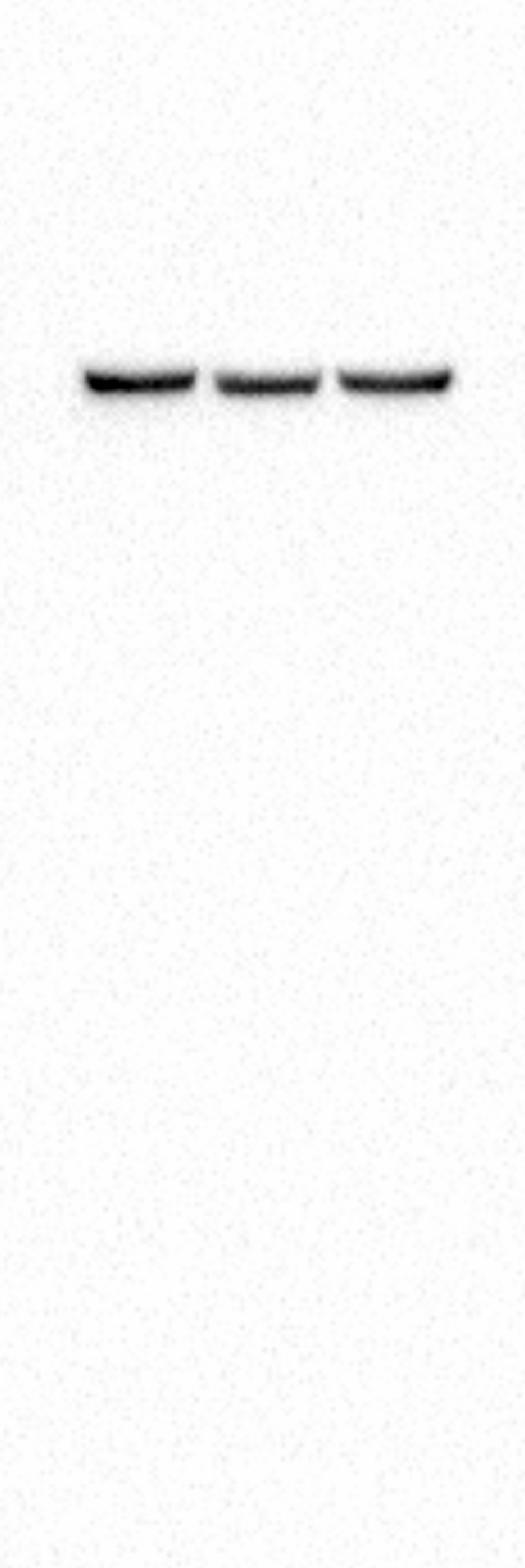

Supplement: Supplemental Information 30 [file peerj-14-21375-s030.zip › Figure 4G WB RAW sh-KLHL40 ACTN2/ACTN2-1 sh-KLHL40.tif]

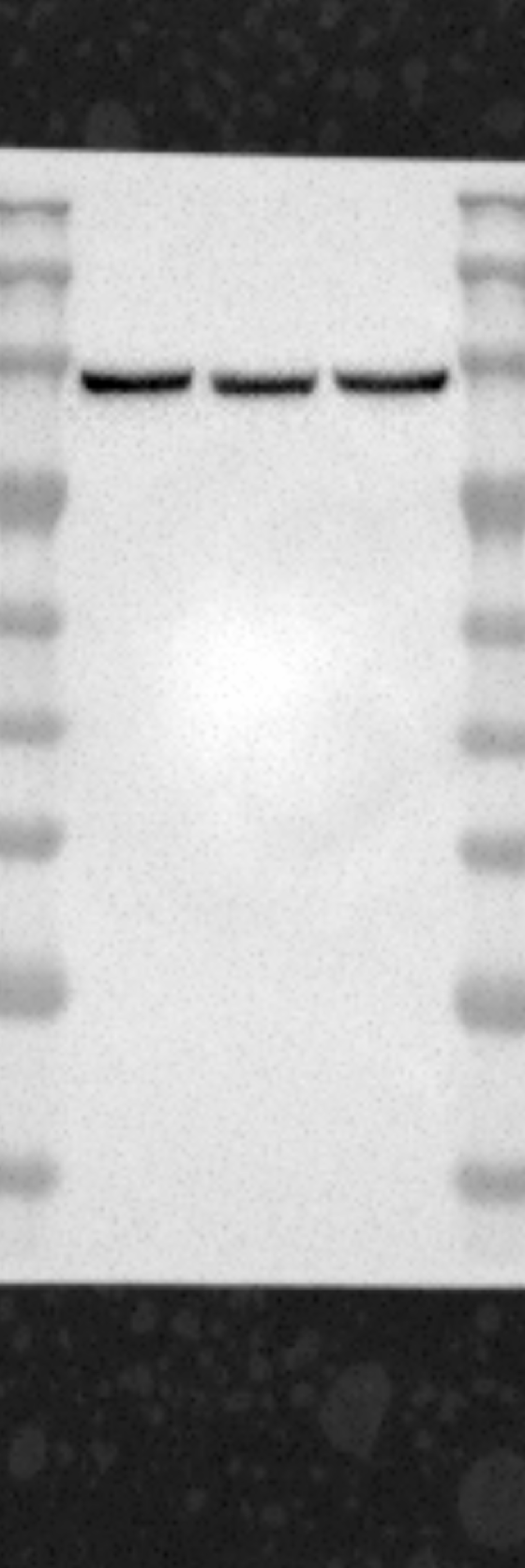

Supplement: Supplemental Information 30 [file peerj-14-21375-s030.zip › Figure 4G WB RAW sh-KLHL40 ACTN2/ACTN2-1 sh-KLHL40+mark.tif]

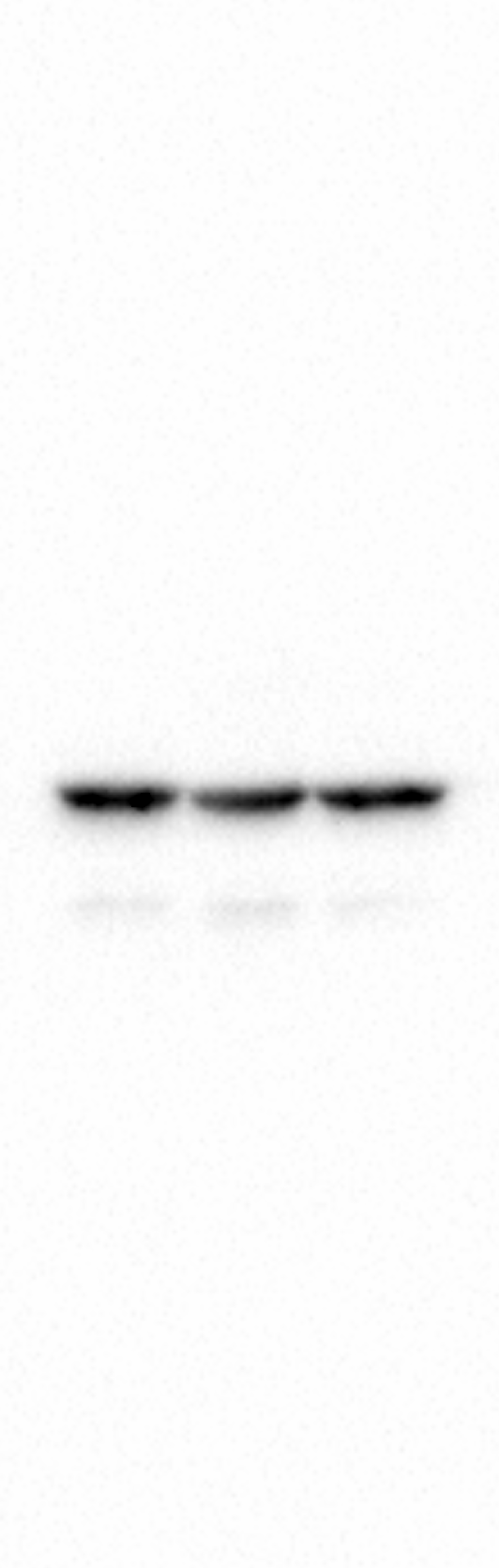

Supplement: Supplemental Information 30 [file peerj-14-21375-s030.zip › Figure 4G WB RAW sh-KLHL40 ACTN2/ACTN2-1 sh-KLHL40-ACTB.tif]

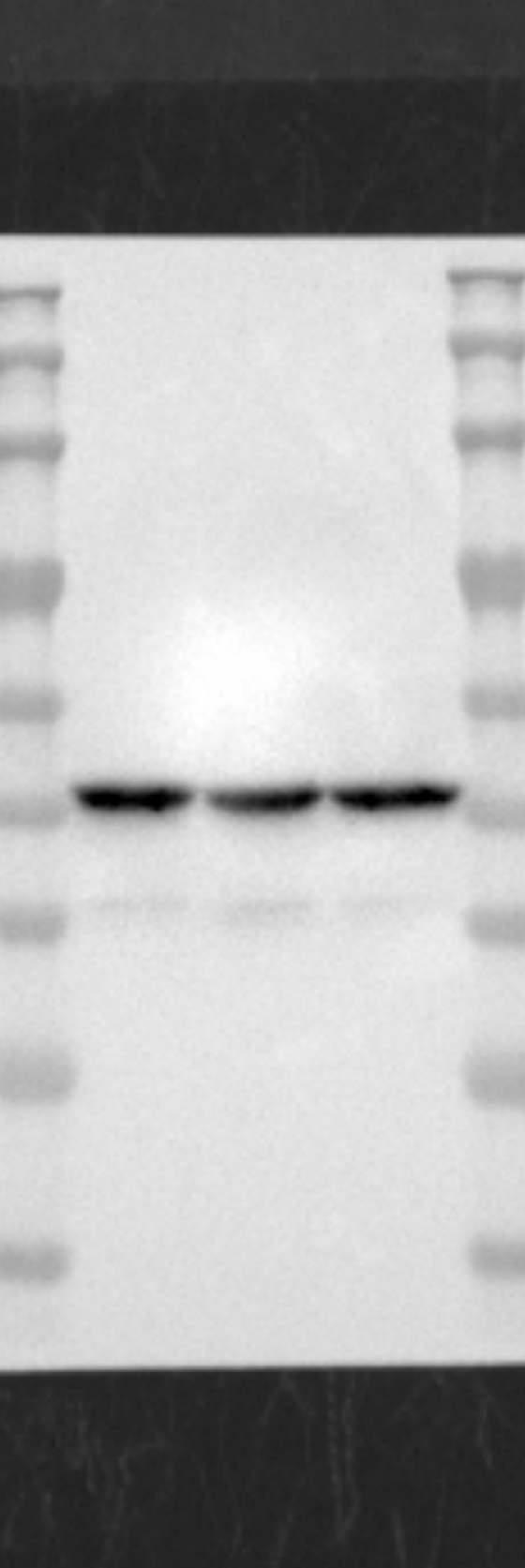

Supplement: Supplemental Information 30 [file peerj-14-21375-s030.zip › Figure 4G WB RAW sh-KLHL40 ACTN2/ACTN2-1 sh-KLHL40-ACTB+MARK.tif]

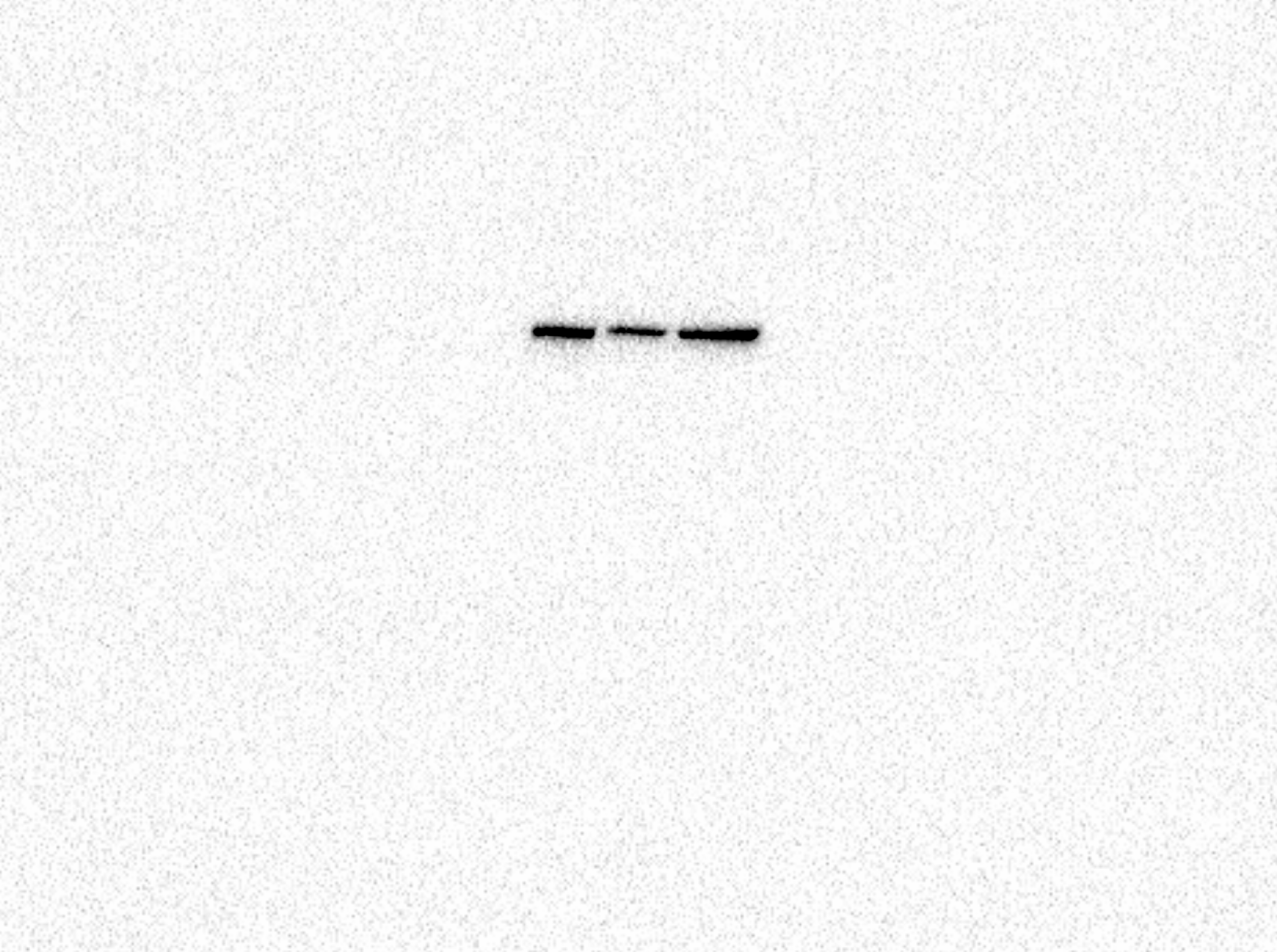

Supplement: Supplemental Information 30 [file peerj-14-21375-s030.zip › Figure 4G WB RAW sh-KLHL40 ACTN2/ACTN2-2 sh-KLHL40.tif]

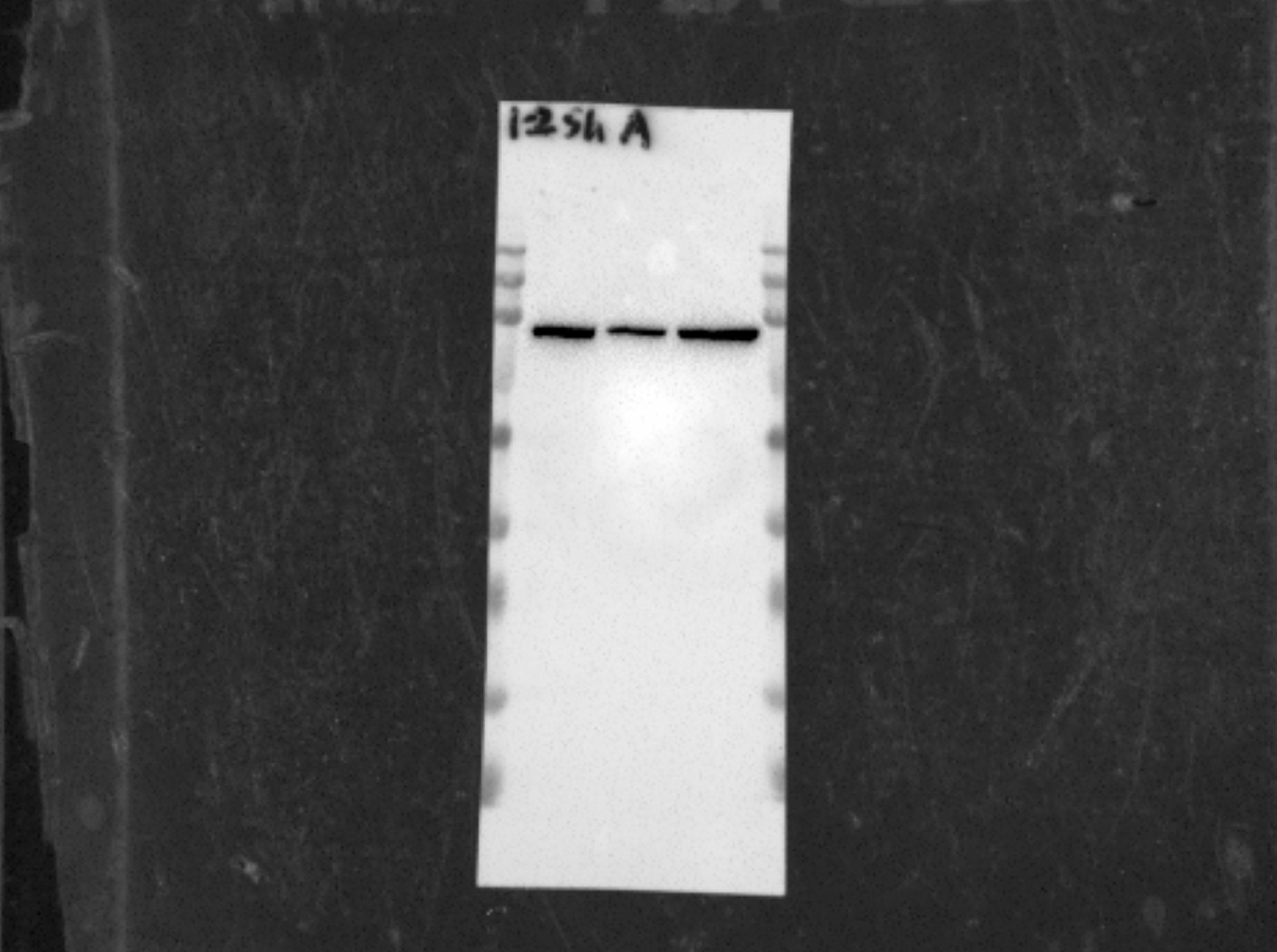

Supplement: Supplemental Information 30 [file peerj-14-21375-s030.zip › Figure 4G WB RAW sh-KLHL40 ACTN2/ACTN2-2 sh-KLHL40+mark.tif]

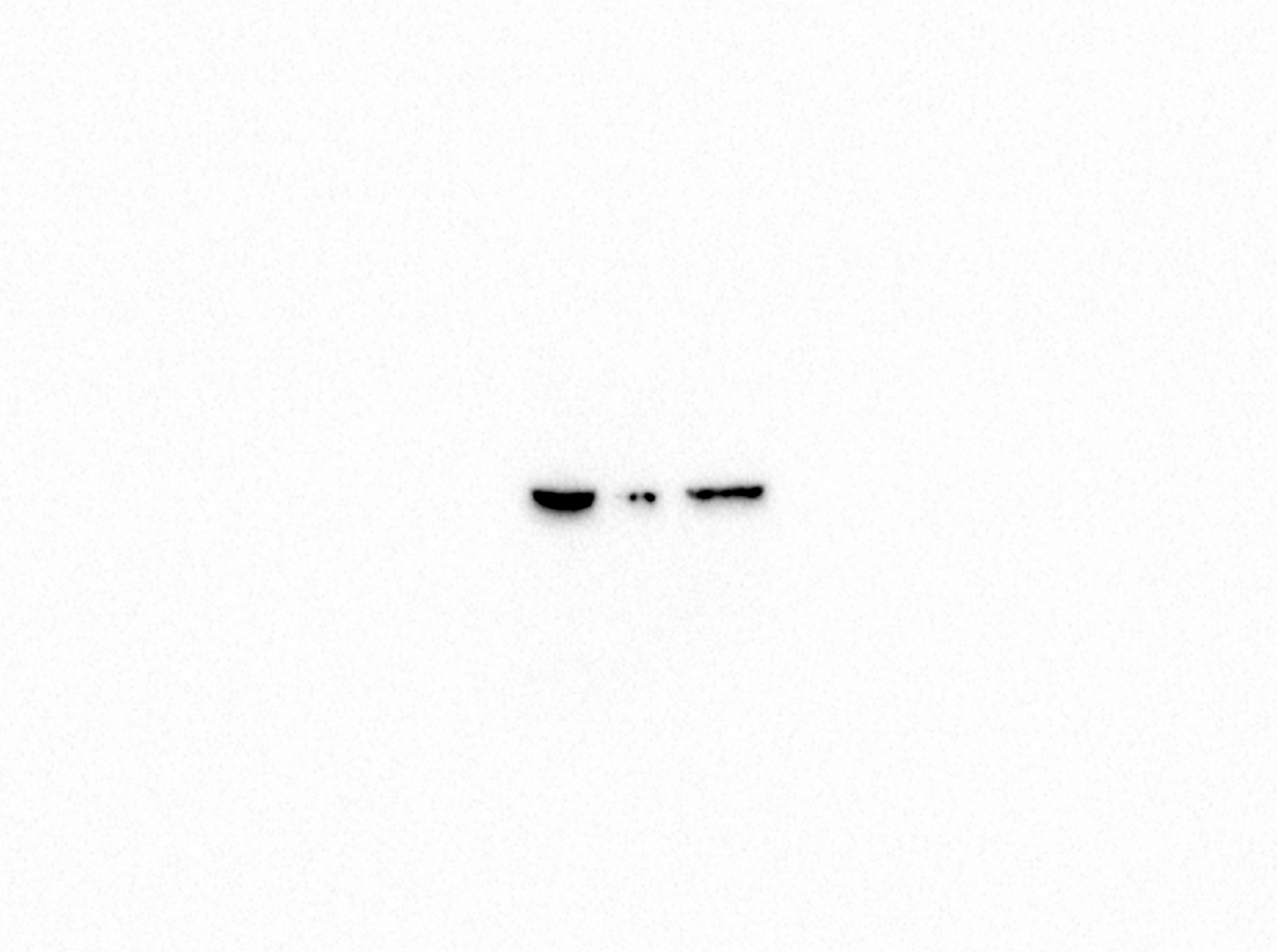

Supplement: Supplemental Information 30 [file peerj-14-21375-s030.zip › Figure 4G WB RAW sh-KLHL40 ACTN2/ACTN2-2 sh-KLHL40-ACTB.tif]

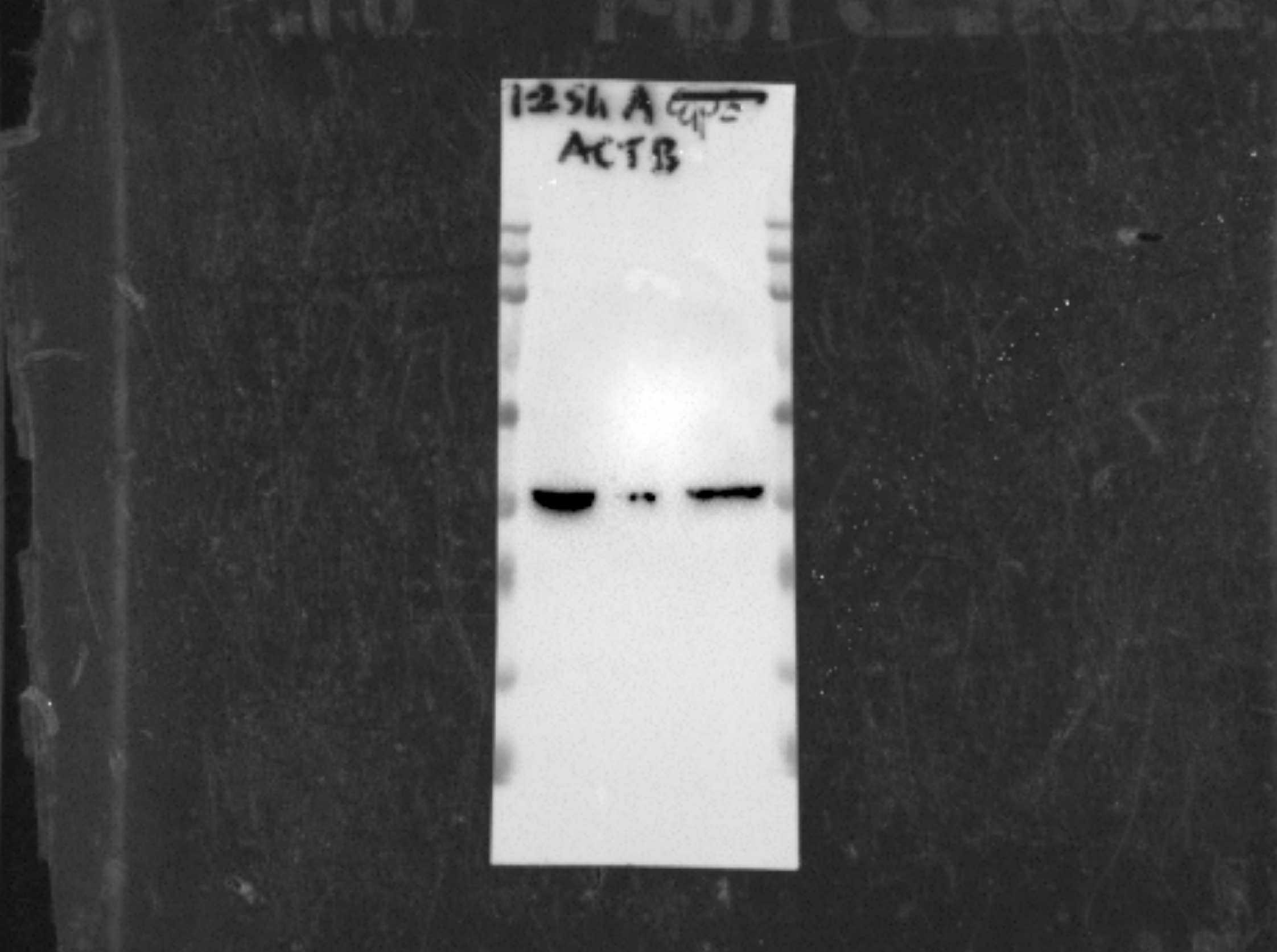

Supplement: Supplemental Information 30 [file peerj-14-21375-s030.zip › Figure 4G WB RAW sh-KLHL40 ACTN2/ACTN2-2 sh-KLHL40-ACTB+MARK.tif]

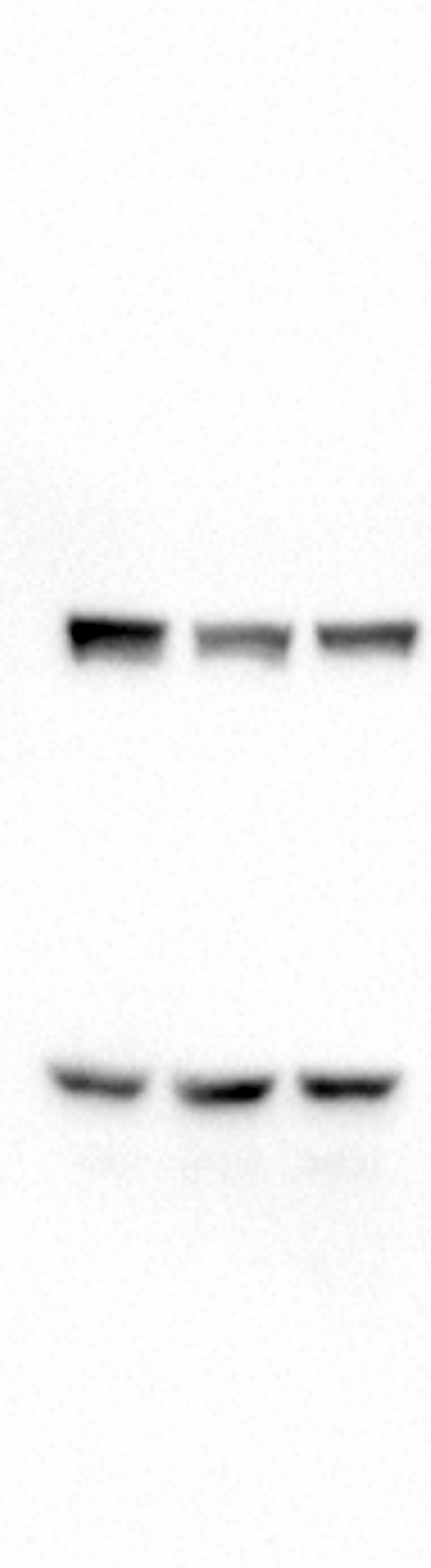

Supplement: Supplemental Information 30 [file peerj-14-21375-s030.zip › Figure 4G WB RAW sh-KLHL40 ACTN2/ACTN2-3-sh-KLHL40+ACTB.tif]

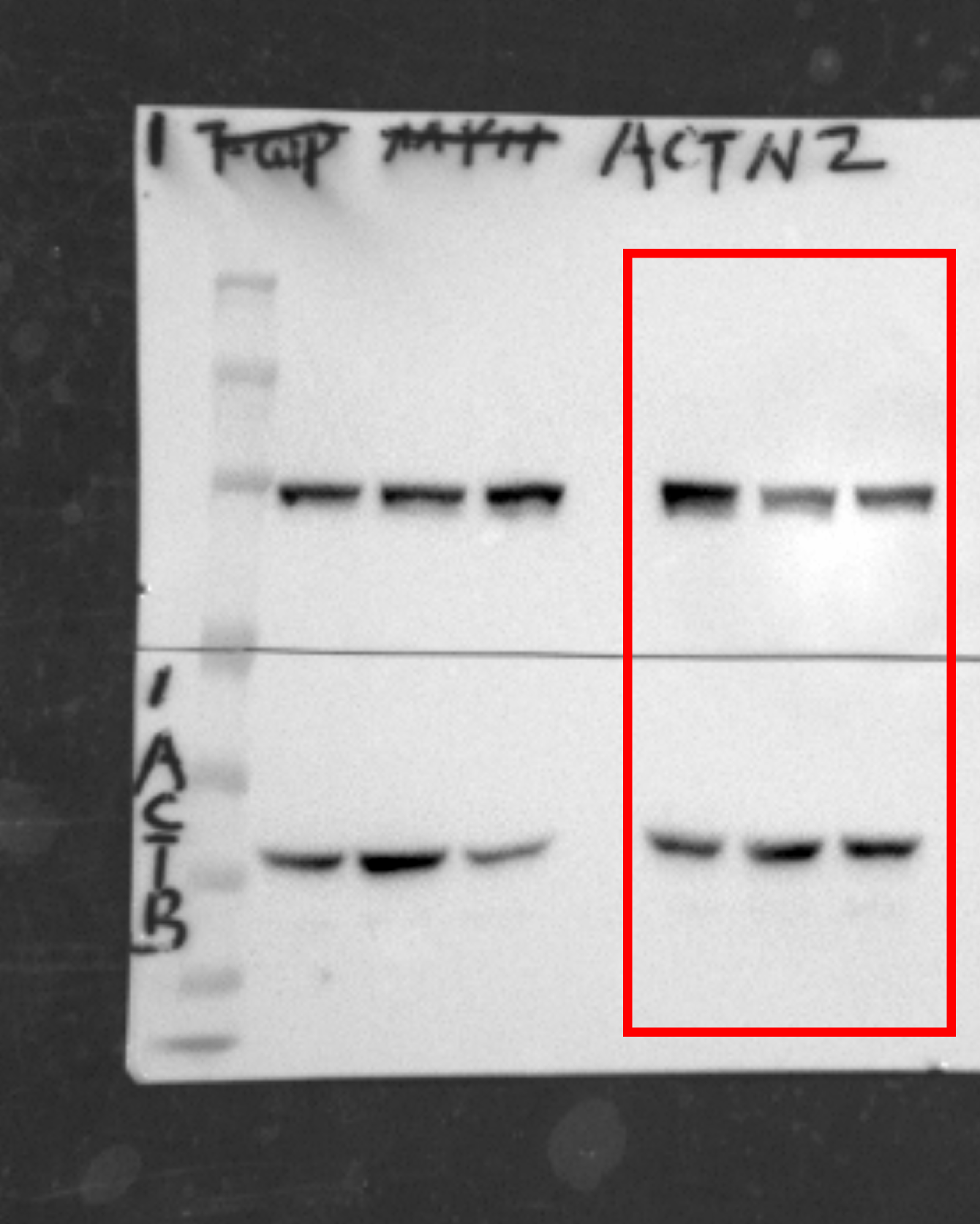

Supplement: Supplemental Information 30 [file peerj-14-21375-s030.zip › Figure 4G WB RAW sh-KLHL40 ACTN2/ACTN2-3-sh-KLHL40+ACTB+mark╕▒▒╛.tif]

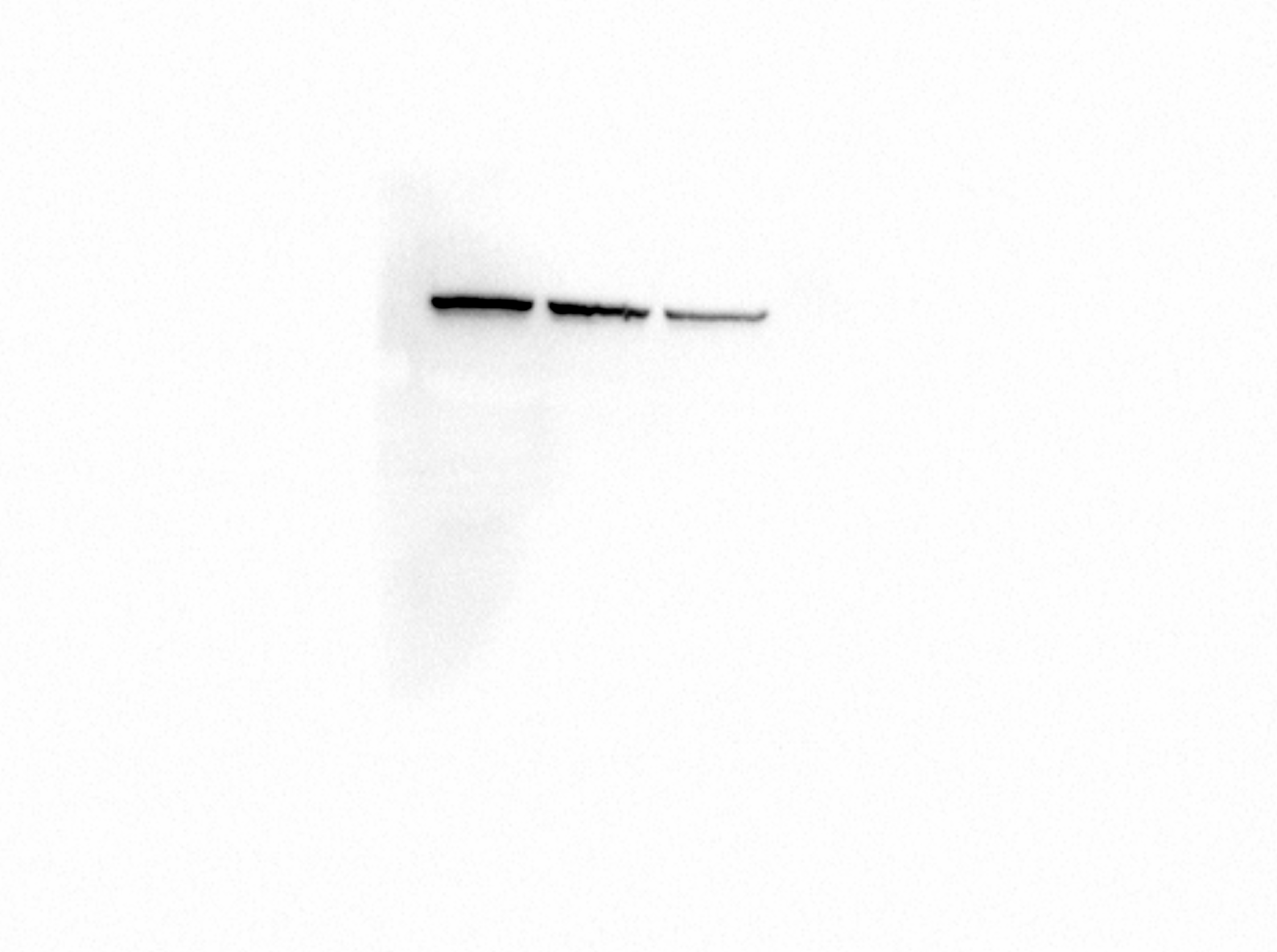

Supplement: Supplemental Information 31 [file peerj-14-21375-s031.zip › Figure 4H WB RAW oe-KLHL40 ACTN2/ACTN2-1 oe-KLHL40.tif]

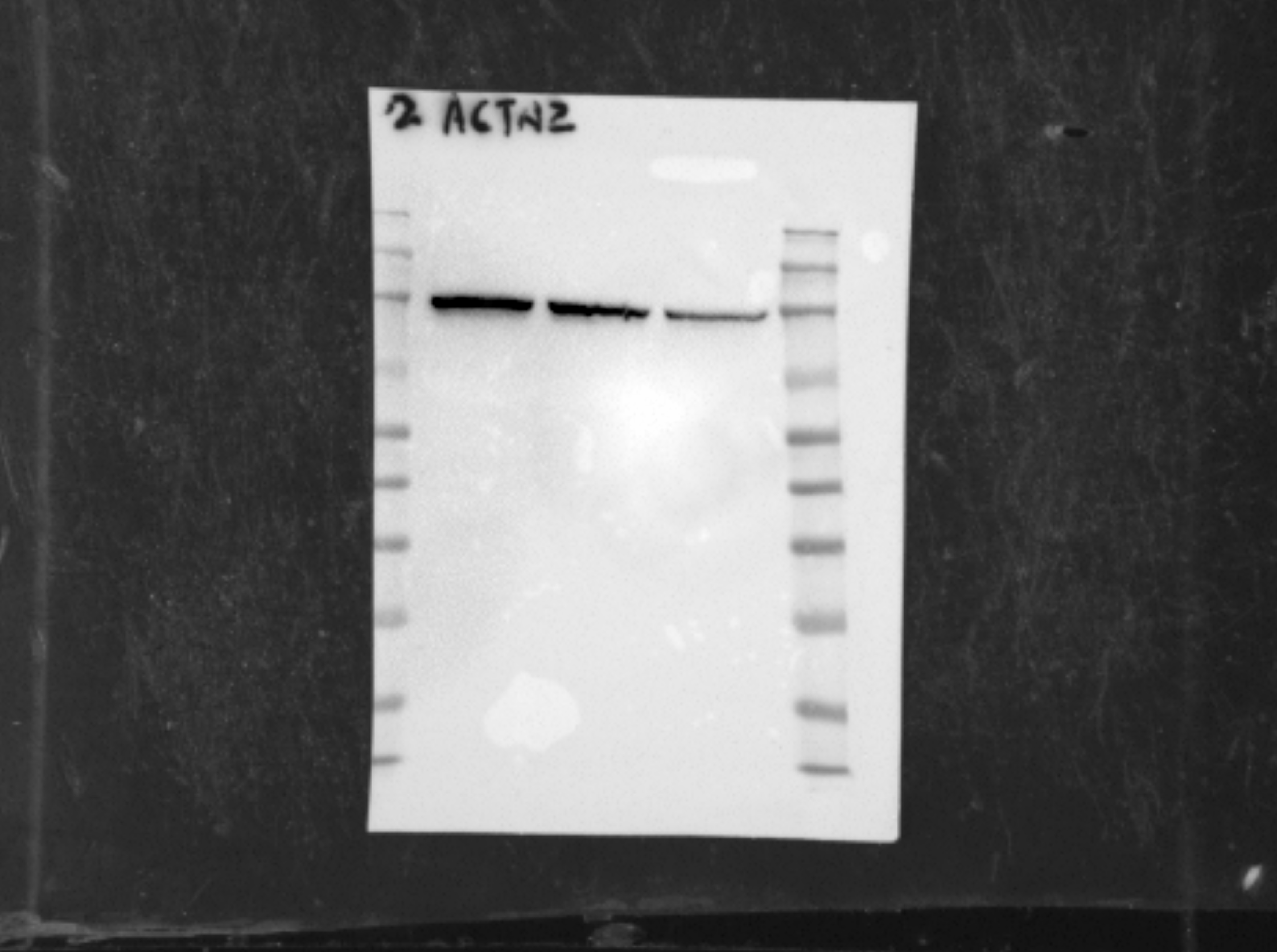

Supplement: Supplemental Information 31 [file peerj-14-21375-s031.zip › Figure 4H WB RAW oe-KLHL40 ACTN2/ACTN2-1 oe-KLHL40+mark.tif]

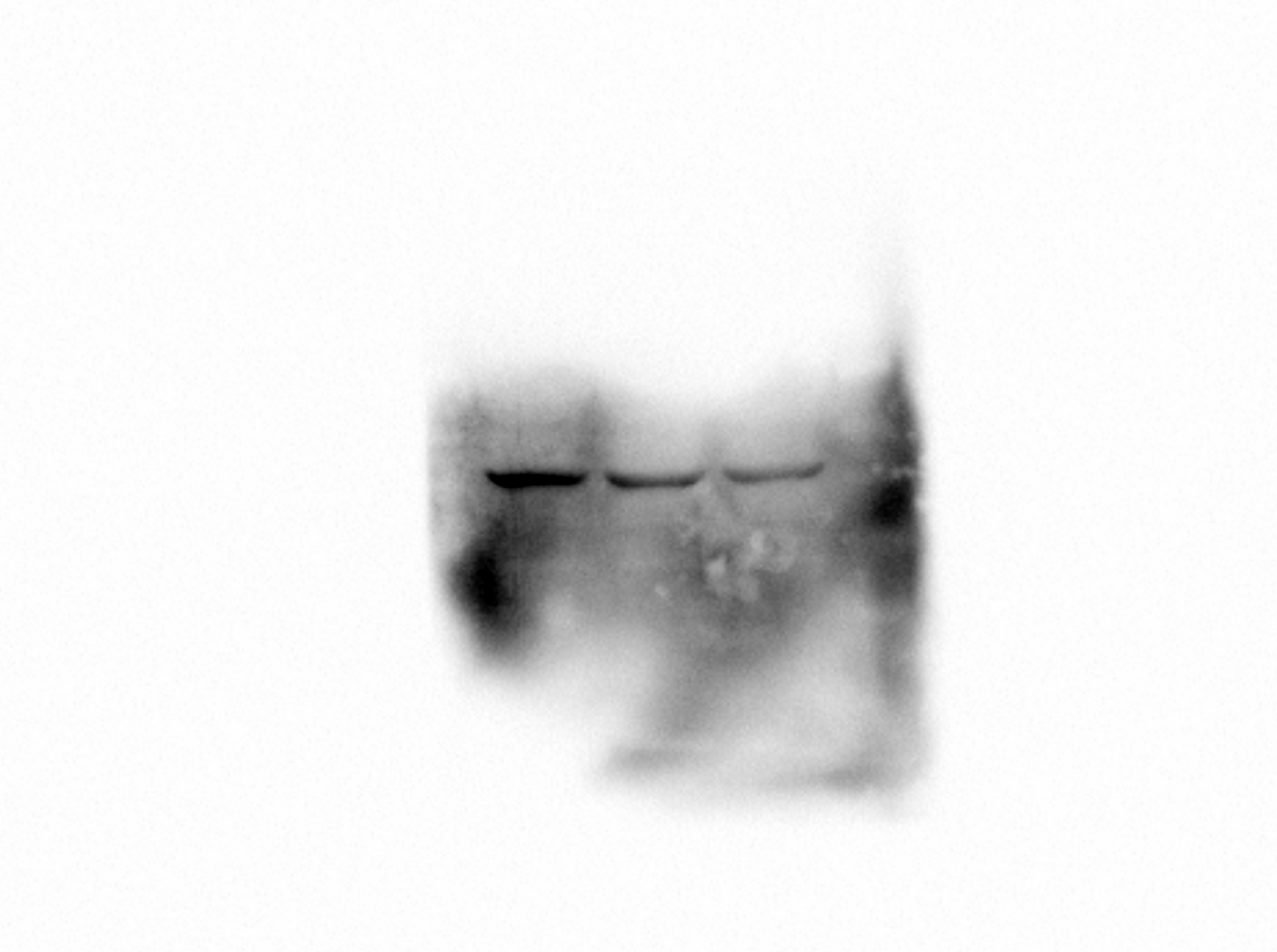

Supplement: Supplemental Information 31 [file peerj-14-21375-s031.zip › Figure 4H WB RAW oe-KLHL40 ACTN2/ACTN2-1 oe-KLHL40-ACTB.tif]

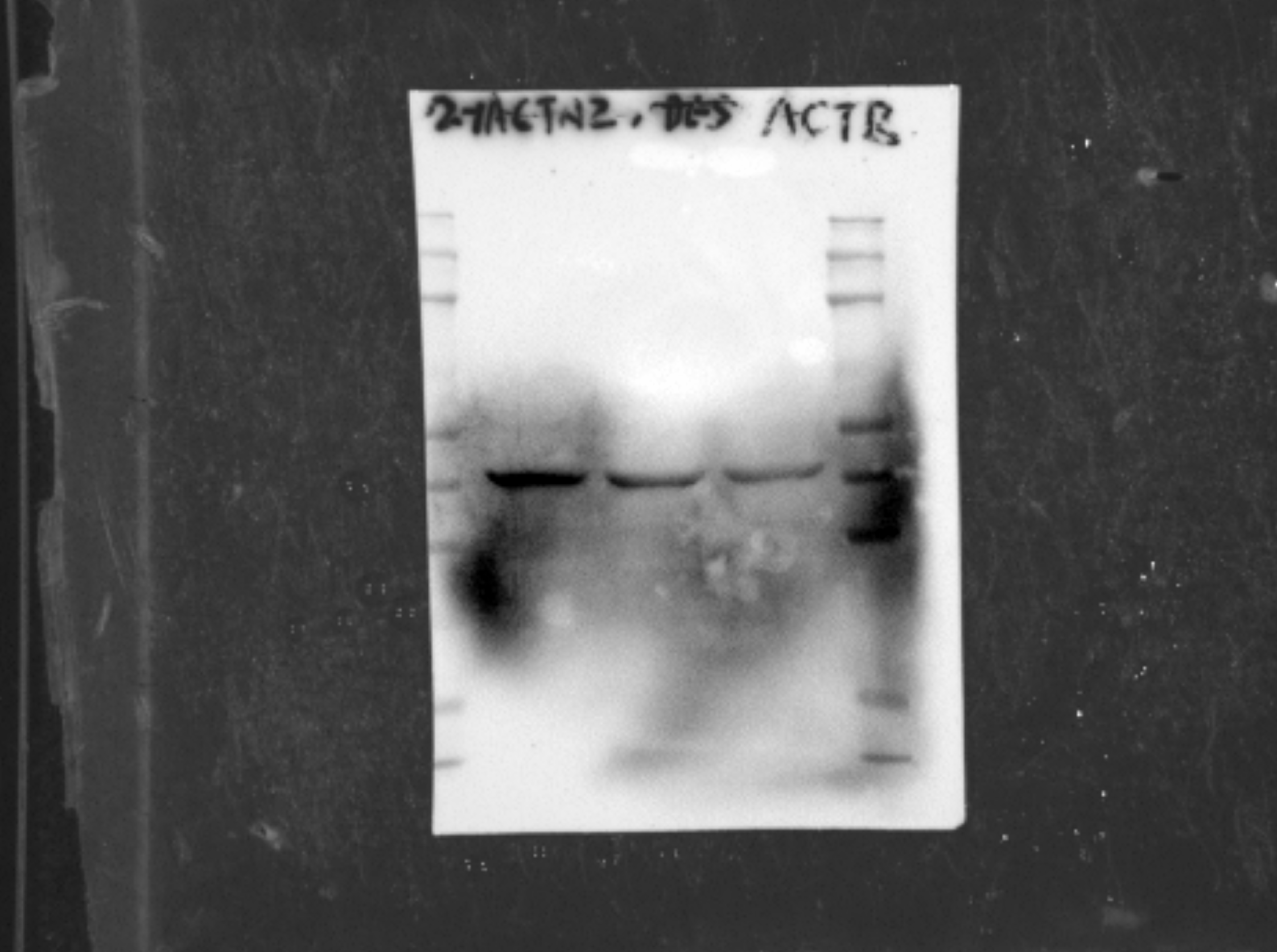

Supplement: Supplemental Information 31 [file peerj-14-21375-s031.zip › Figure 4H WB RAW oe-KLHL40 ACTN2/ACTN2-1 oe-KLHL40-ACTB+MARK.tif]

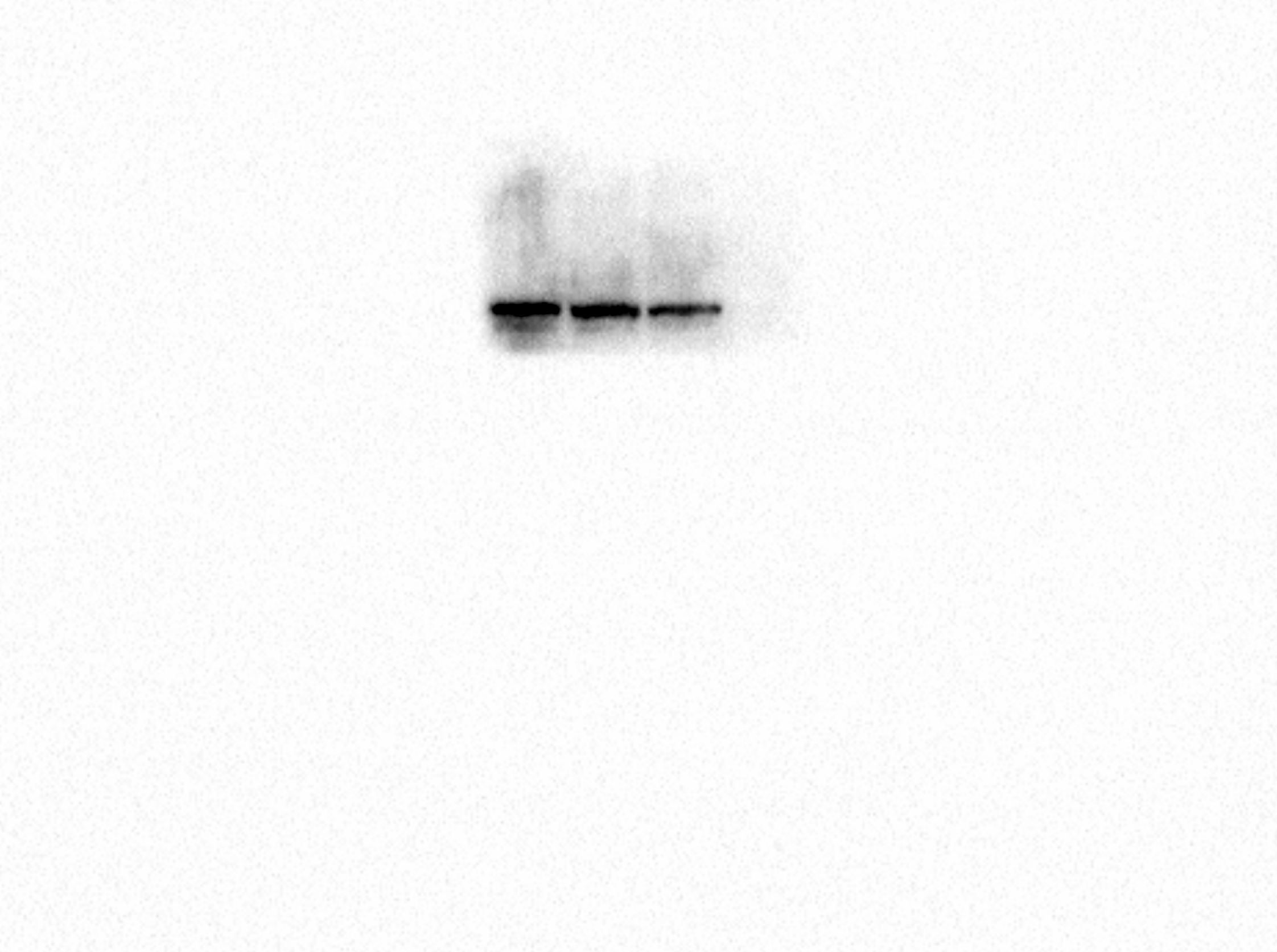

Supplement: Supplemental Information 31 [file peerj-14-21375-s031.zip › Figure 4H WB RAW oe-KLHL40 ACTN2/ACTN2-2 oe-KLHL40.tif]

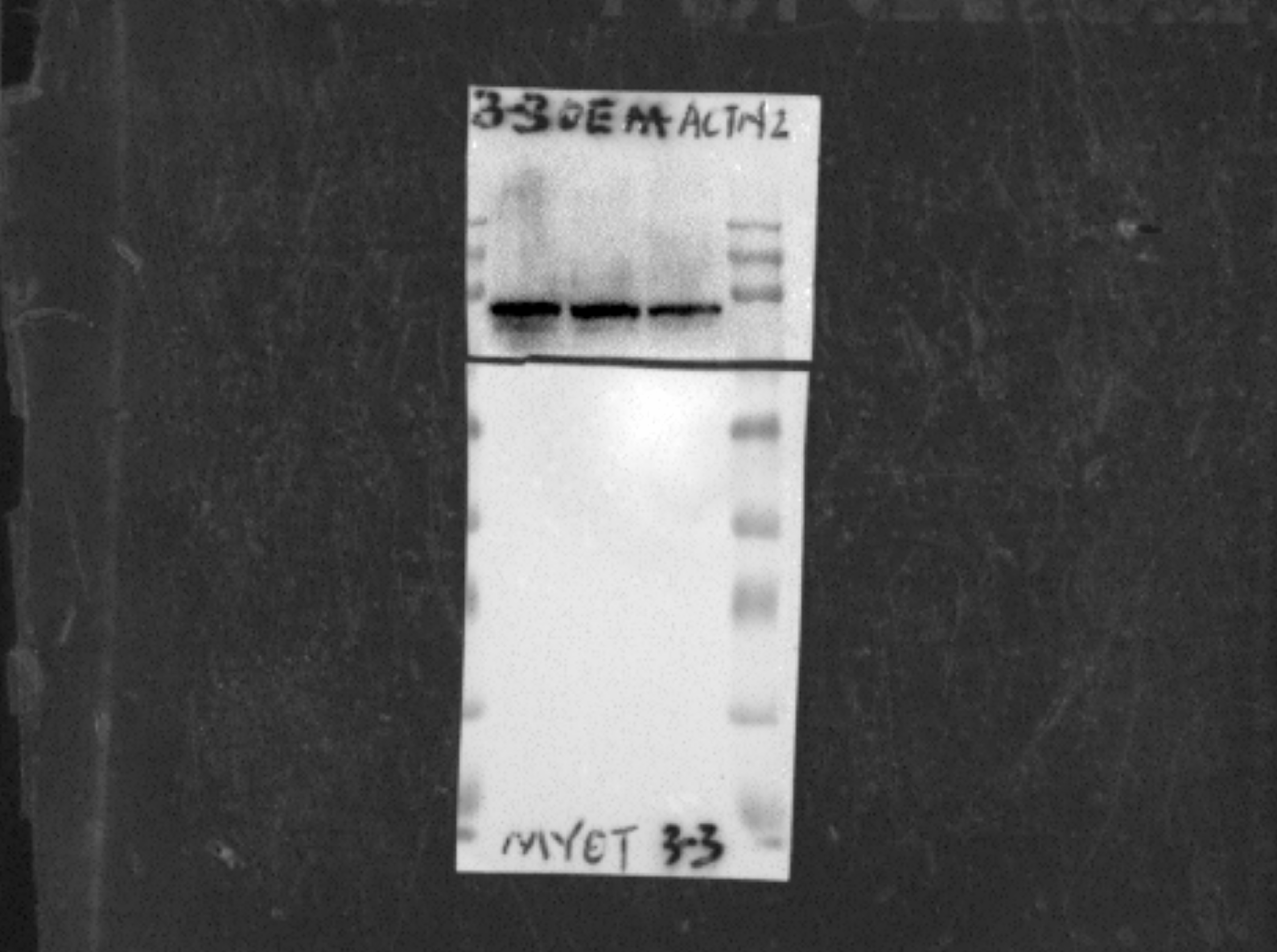

Supplement: Supplemental Information 31 [file peerj-14-21375-s031.zip › Figure 4H WB RAW oe-KLHL40 ACTN2/ACTN2-2 oe-KLHL40+mark.tif]

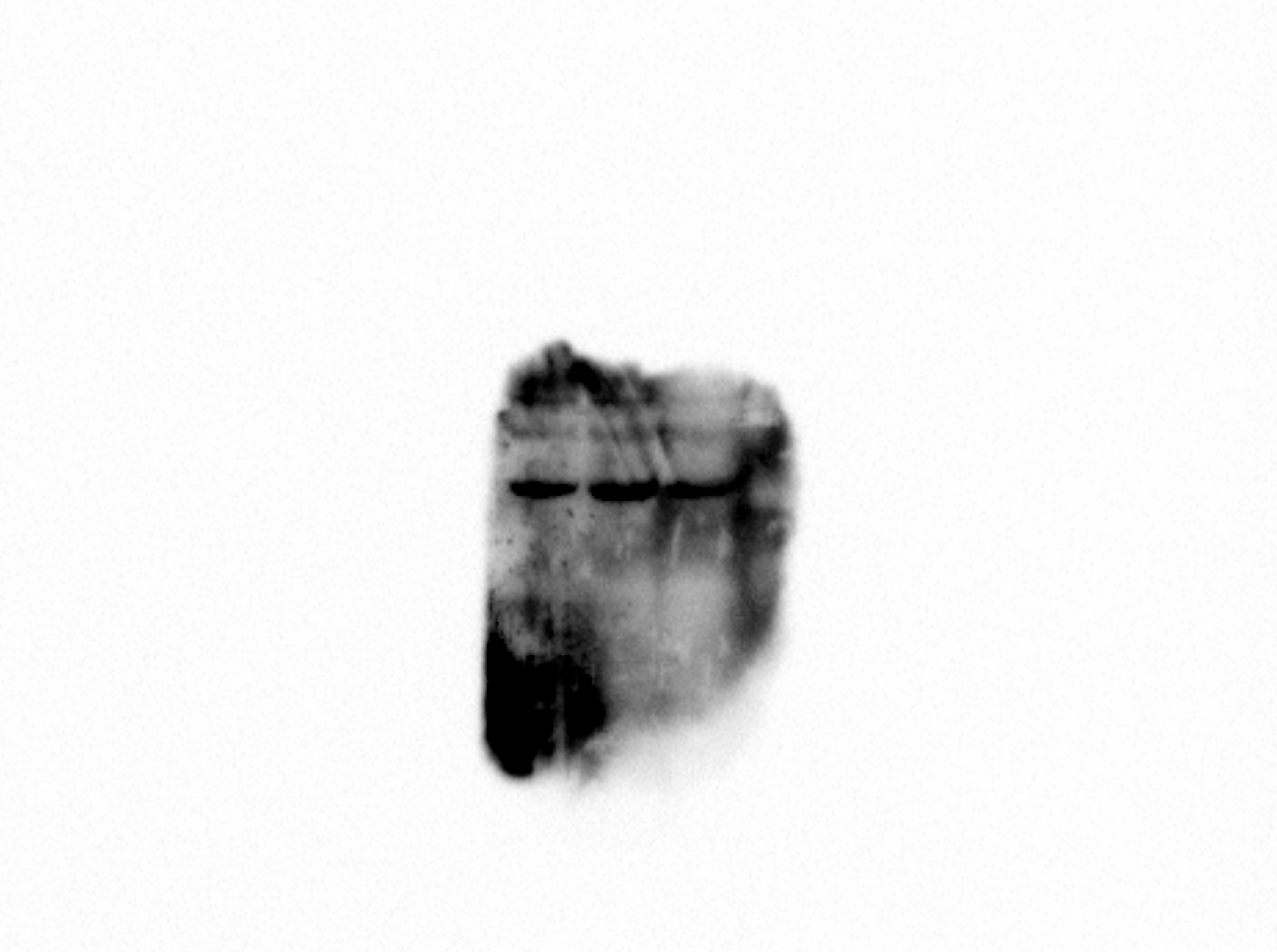

Supplement: Supplemental Information 31 [file peerj-14-21375-s031.zip › Figure 4H WB RAW oe-KLHL40 ACTN2/ACTN2-2 oe-KLHL40-ACTB.tif]

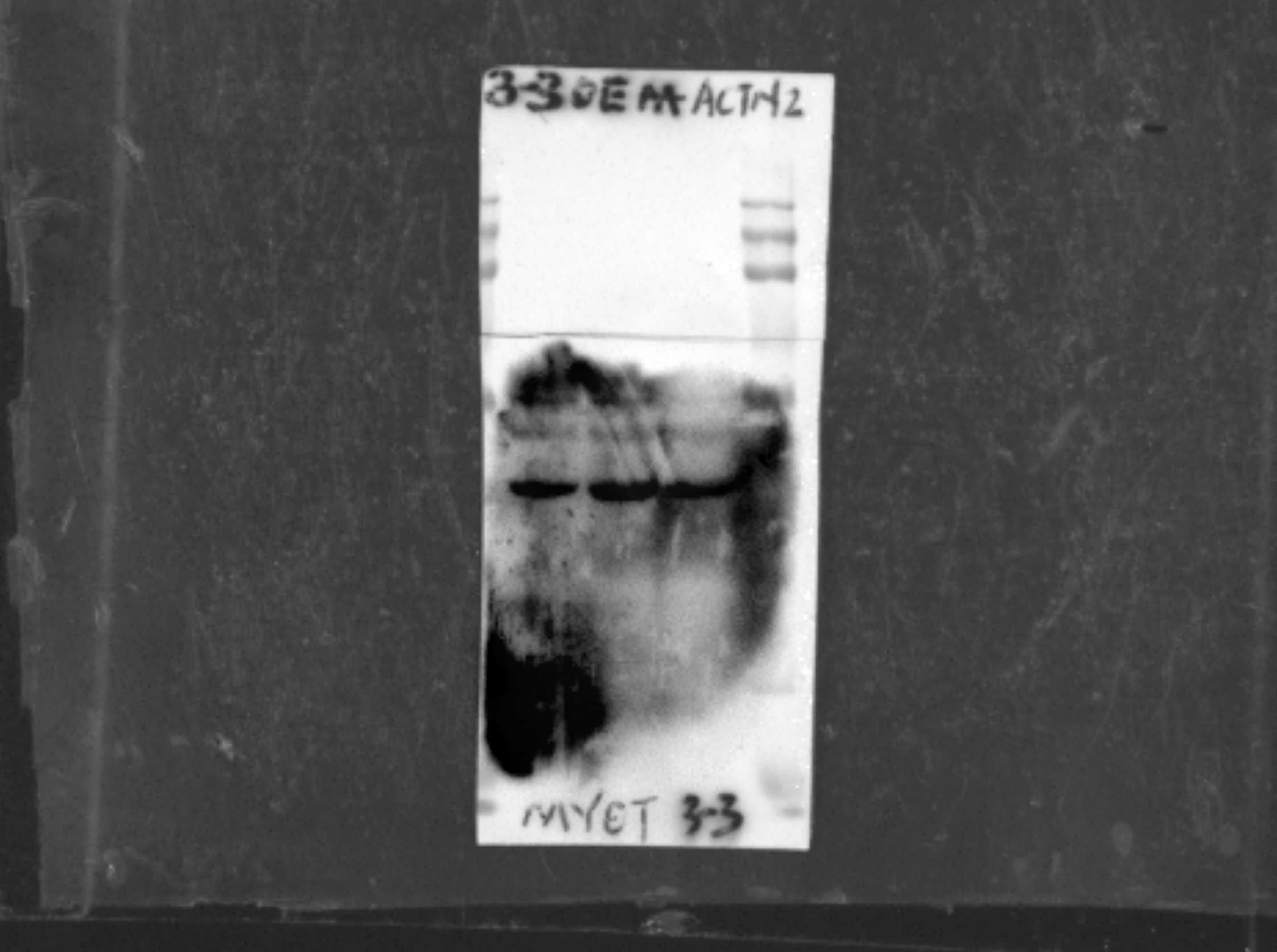

Supplement: Supplemental Information 31 [file peerj-14-21375-s031.zip › Figure 4H WB RAW oe-KLHL40 ACTN2/ACTN2-2 oe-KLHL40-ACTB+MARK.tif]

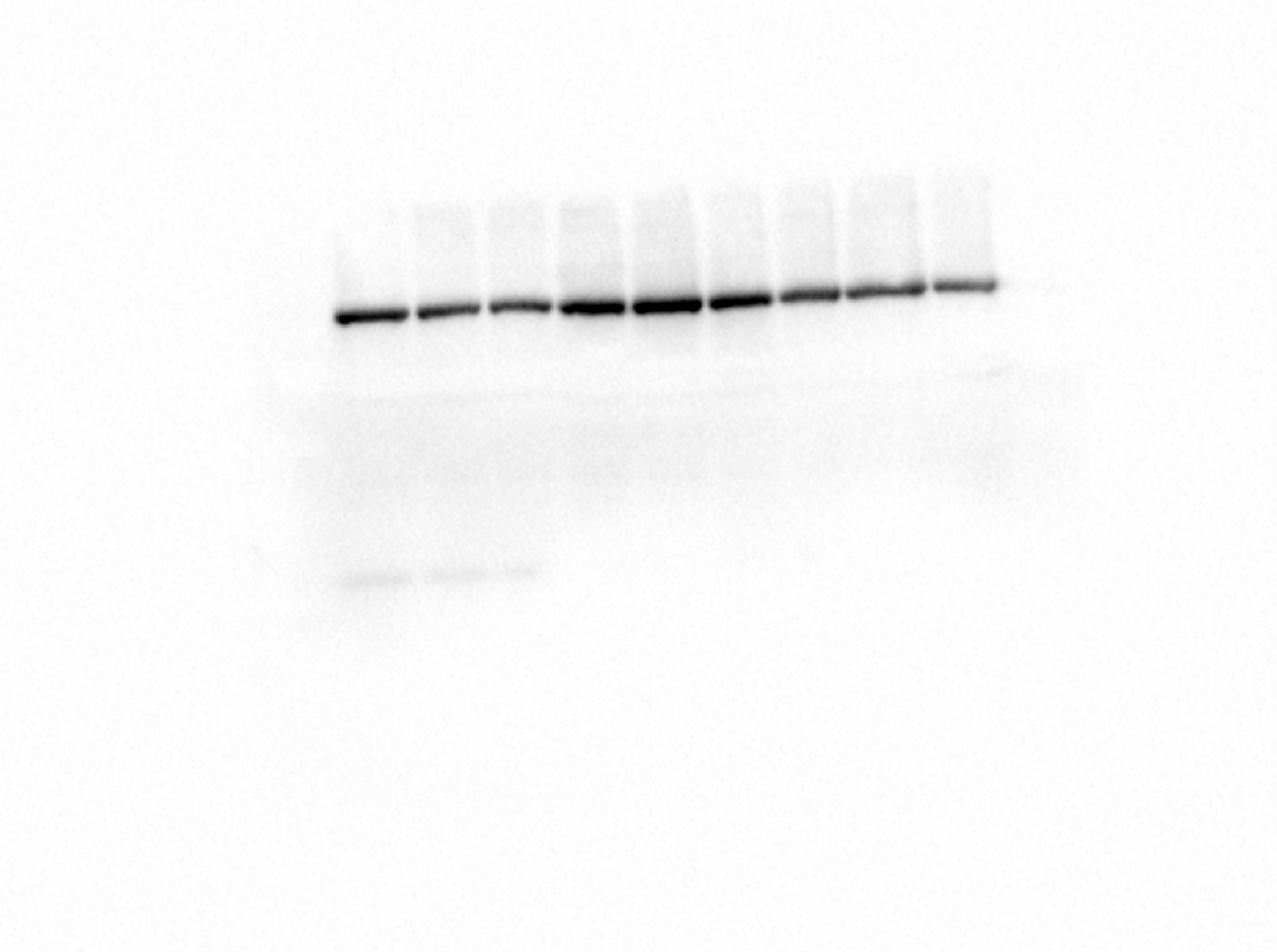

Supplement: Supplemental Information 31 [file peerj-14-21375-s031.zip › Figure 4H WB RAW oe-KLHL40 ACTN2/ACTN2-3 oe-KLHL40.tif]

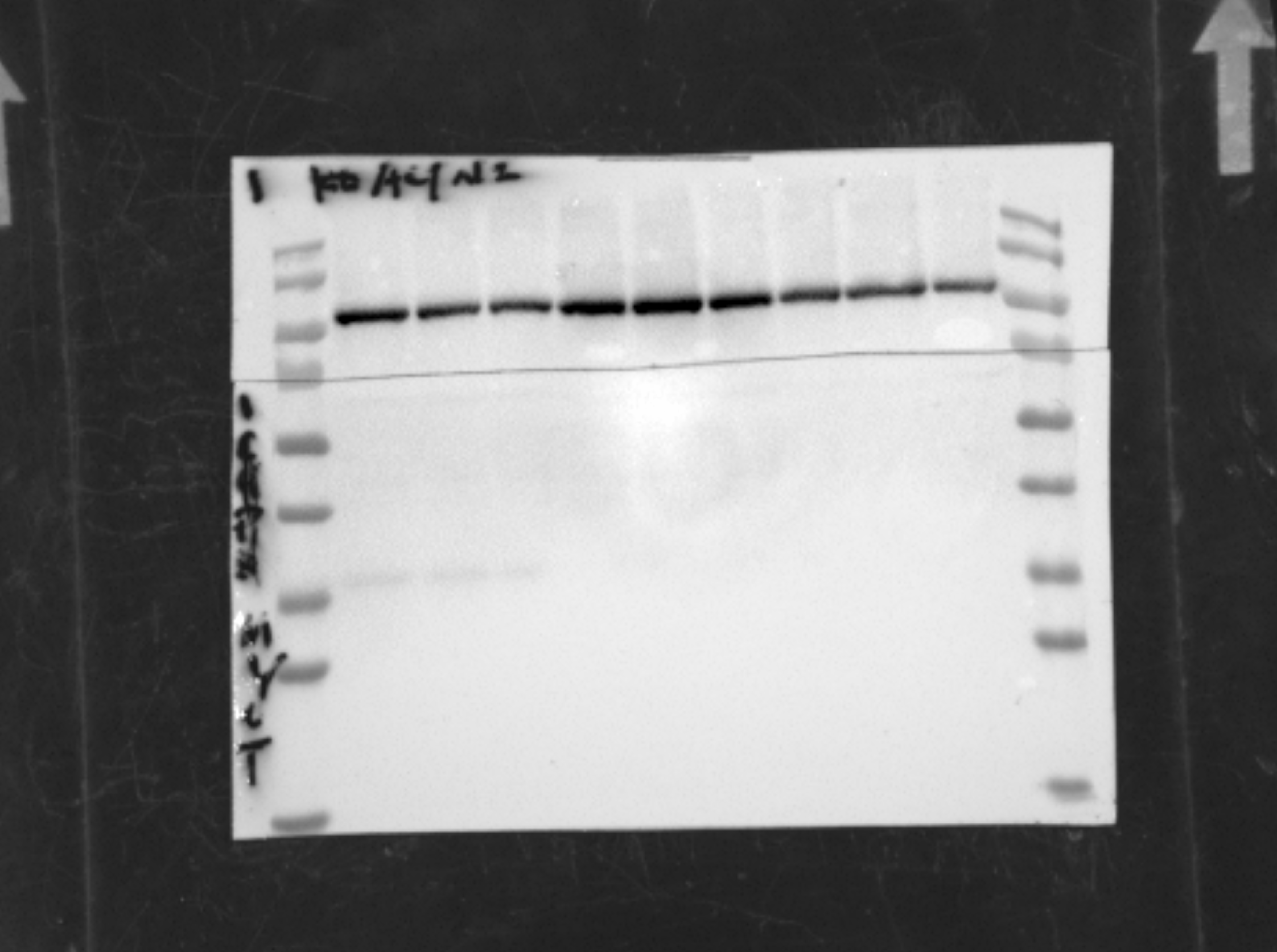

Supplement: Supplemental Information 31 [file peerj-14-21375-s031.zip › Figure 4H WB RAW oe-KLHL40 ACTN2/ACTN2-3 oe-KLHL40+mark.tif]

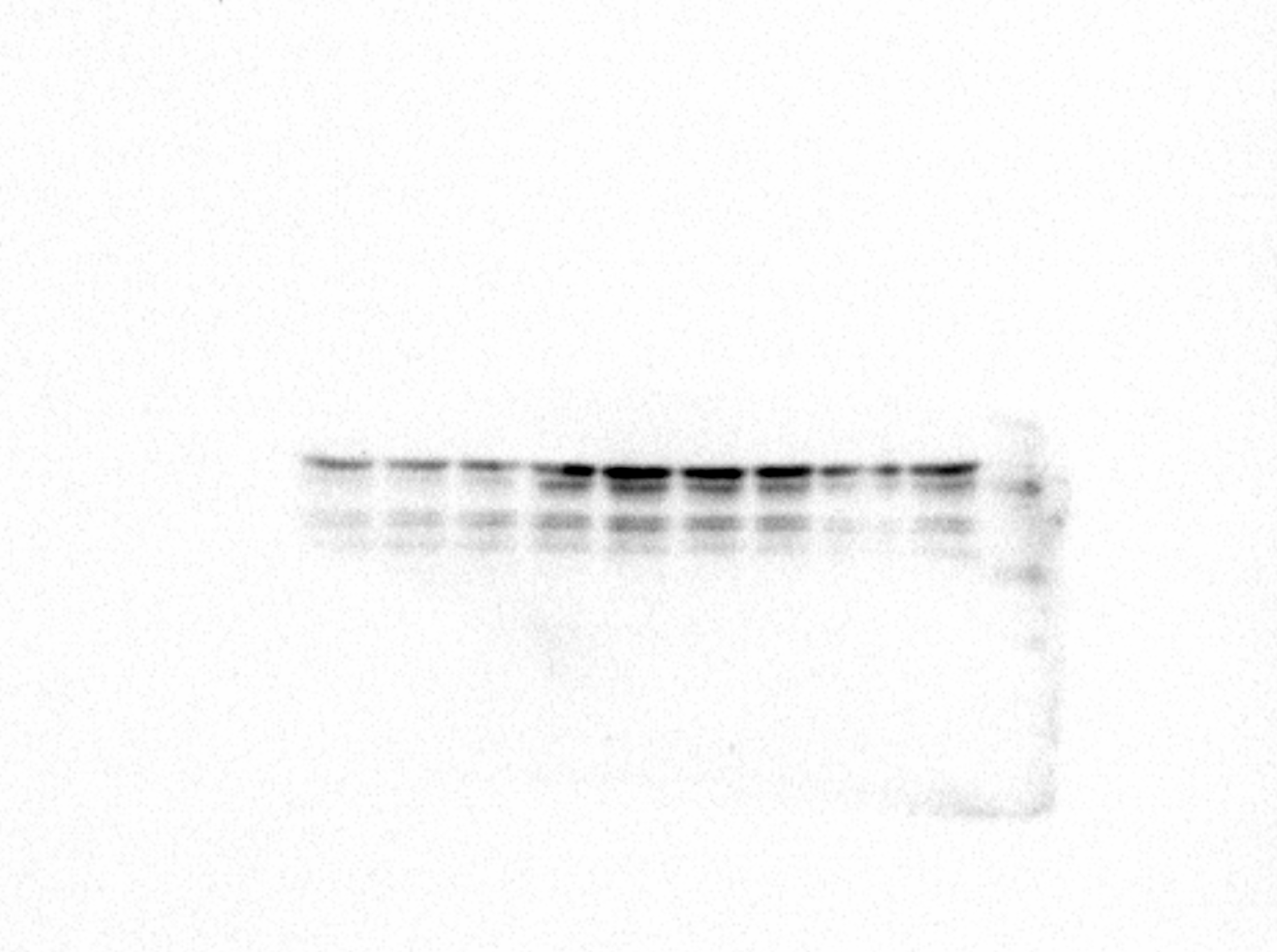

Supplement: Supplemental Information 31 [file peerj-14-21375-s031.zip › Figure 4H WB RAW oe-KLHL40 ACTN2/ACTN2-3 oe-KLHL40-ACTB.tif]

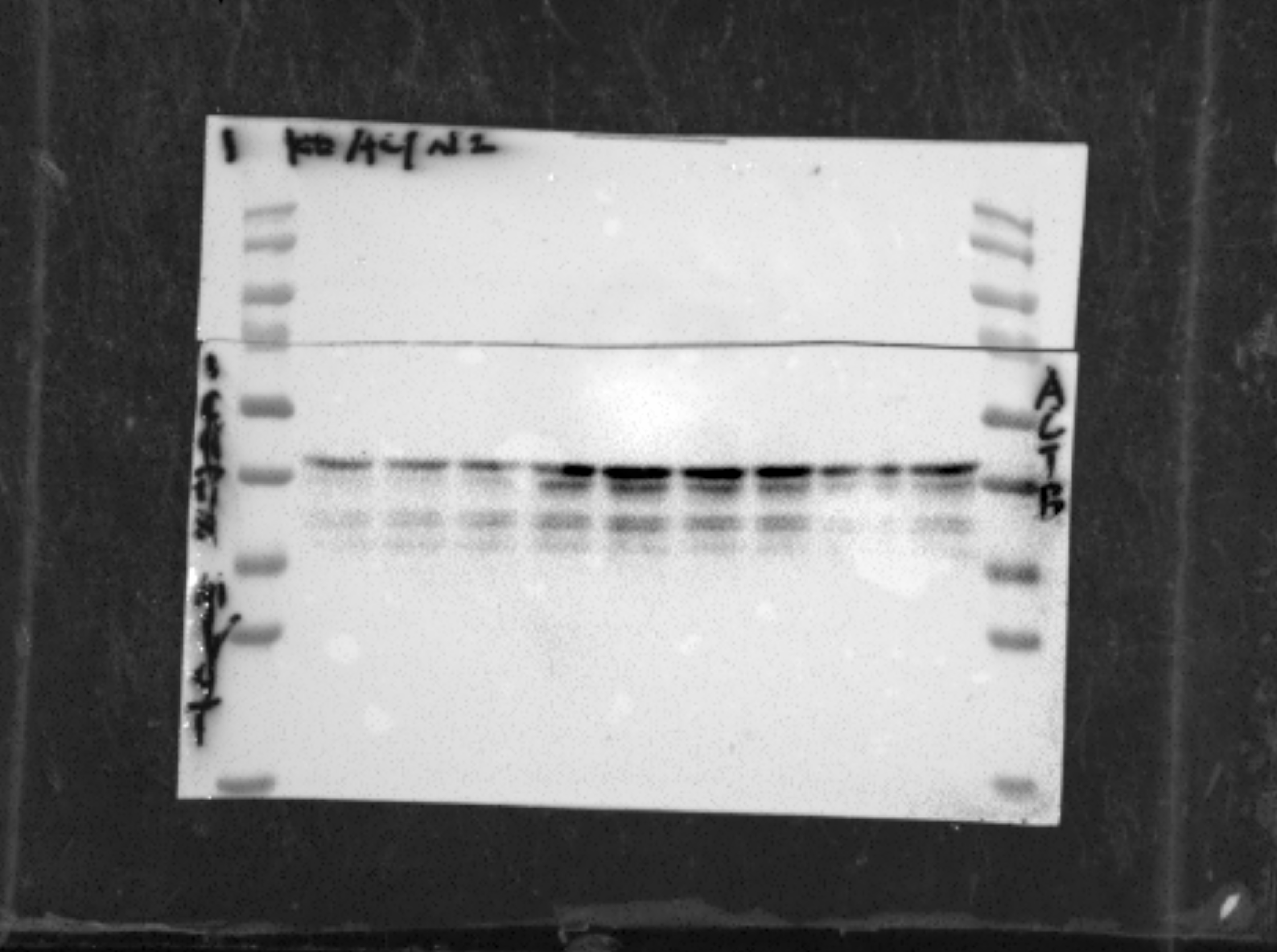

Supplement: Supplemental Information 31 [file peerj-14-21375-s031.zip › Figure 4H WB RAW oe-KLHL40 ACTN2/ACTN2-3 oe-KLHL40-ACTB+MARK.tif]

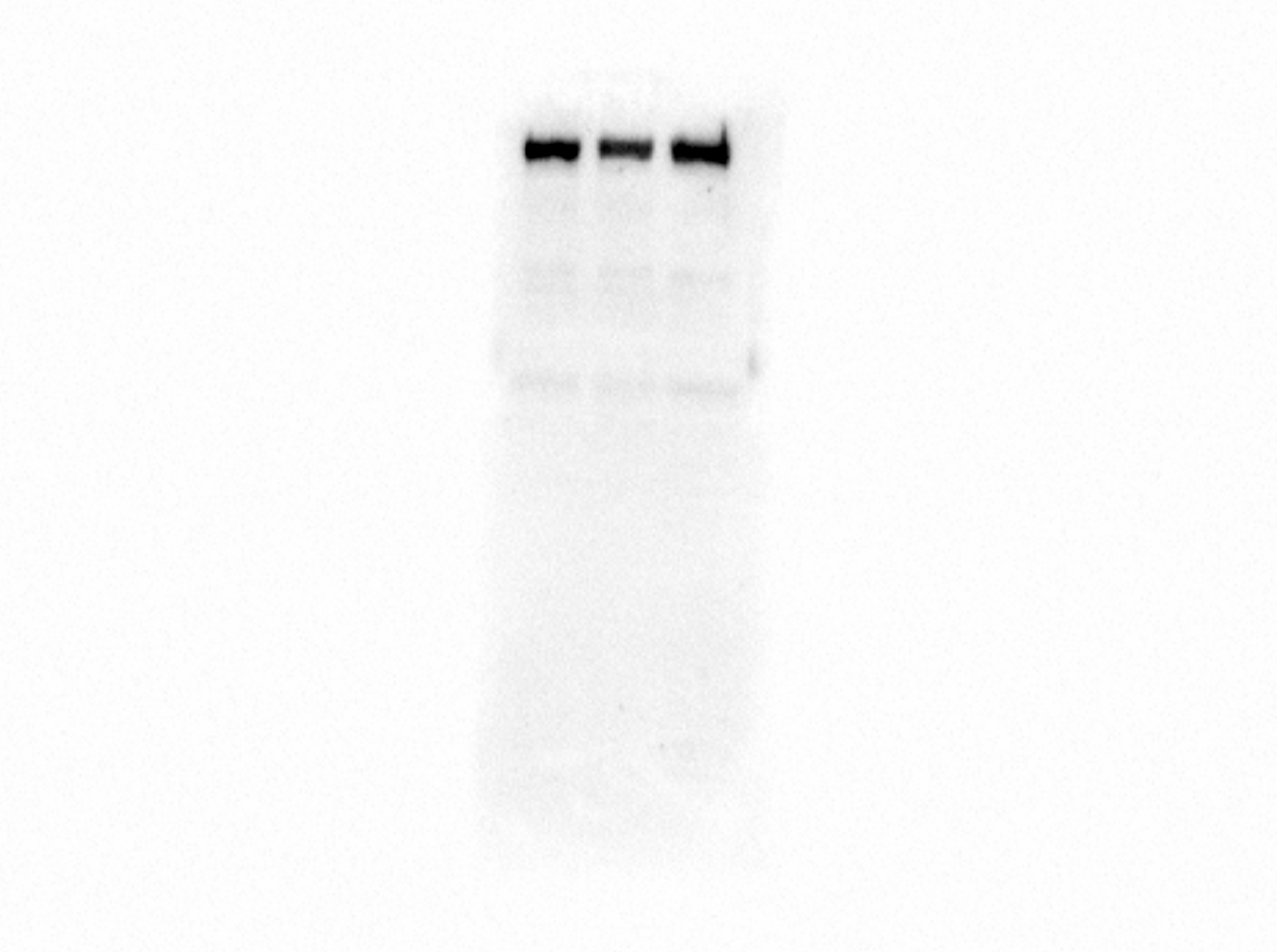

Supplement: Supplemental Information 32 [file peerj-14-21375-s032.zip › Figure 4I WB RAW sh-KLHL40 FLNC/FLNC-1 sh-KLHL40.tif]

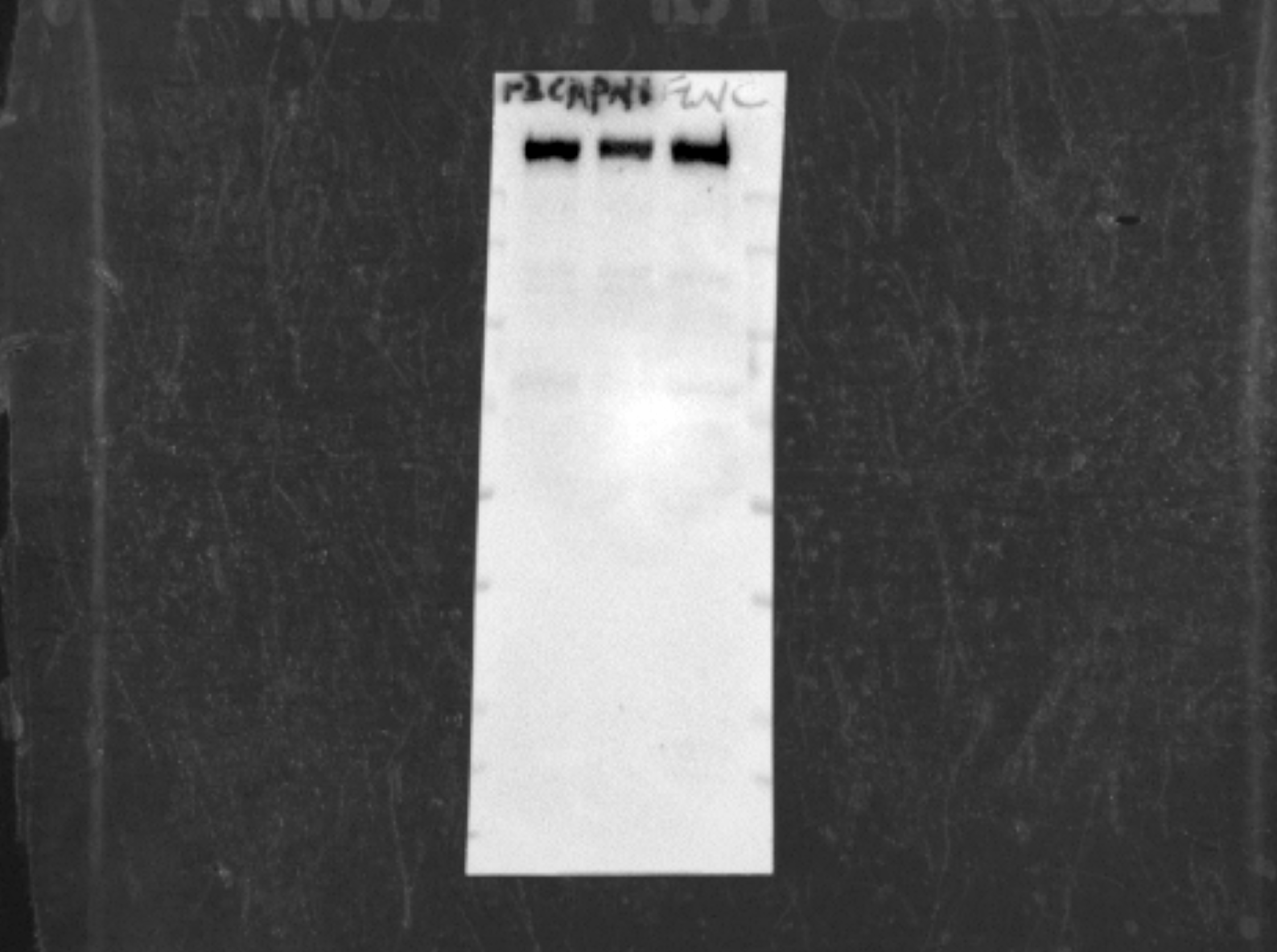

Supplement: Supplemental Information 32 [file peerj-14-21375-s032.zip › Figure 4I WB RAW sh-KLHL40 FLNC/FLNC-1 sh-KLHL40+MARK.tif]

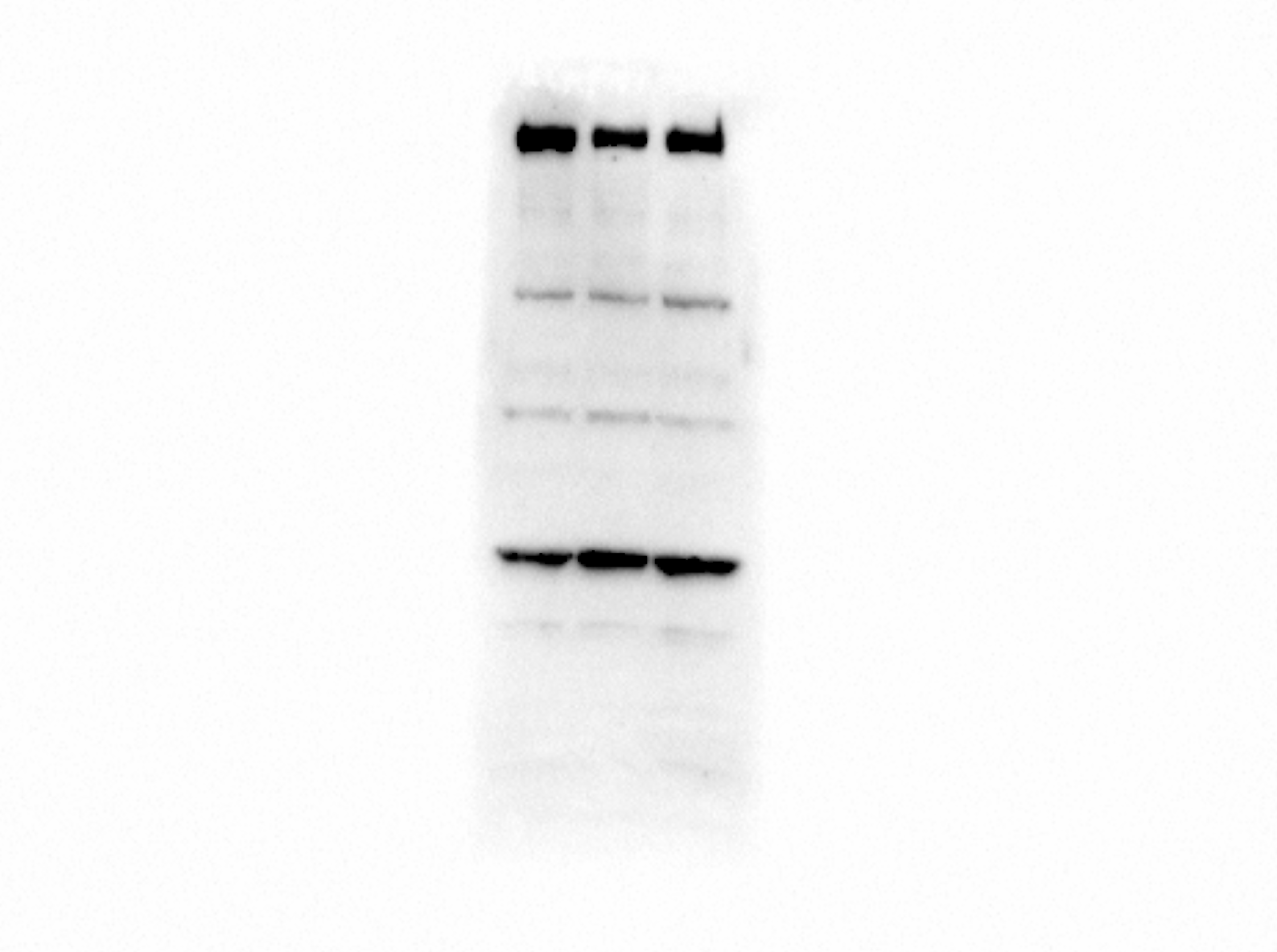

Supplement: Supplemental Information 32 [file peerj-14-21375-s032.zip › Figure 4I WB RAW sh-KLHL40 FLNC/FLNC-1 sh-KLHL40-ACTB.tif]

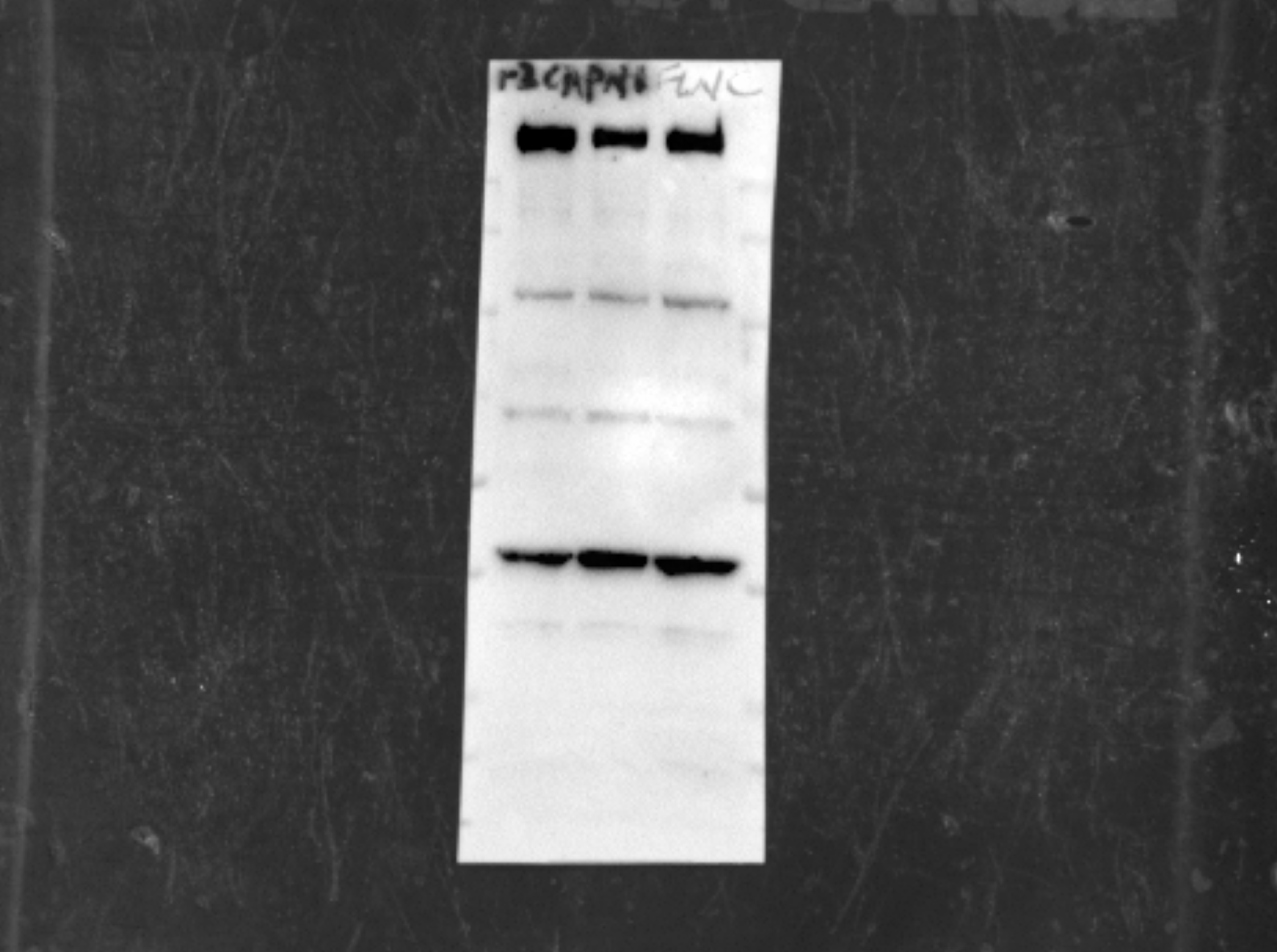

Supplement: Supplemental Information 32 [file peerj-14-21375-s032.zip › Figure 4I WB RAW sh-KLHL40 FLNC/FLNC-1 sh-KLHL40-ACTB+MARK.tif]

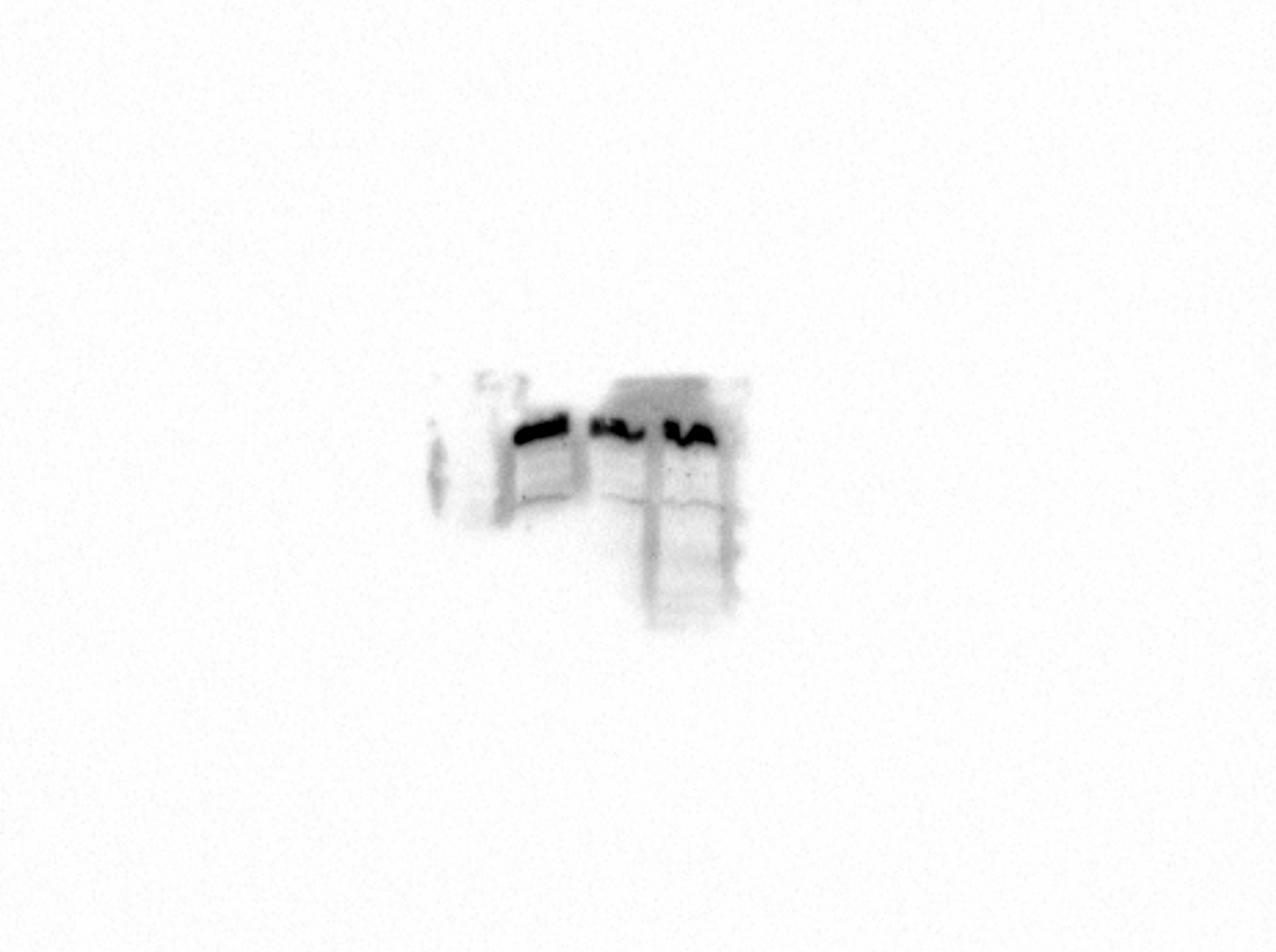

Supplement: Supplemental Information 32 [file peerj-14-21375-s032.zip › Figure 4I WB RAW sh-KLHL40 FLNC/FLNC-2 sh-KLHL40.tif]

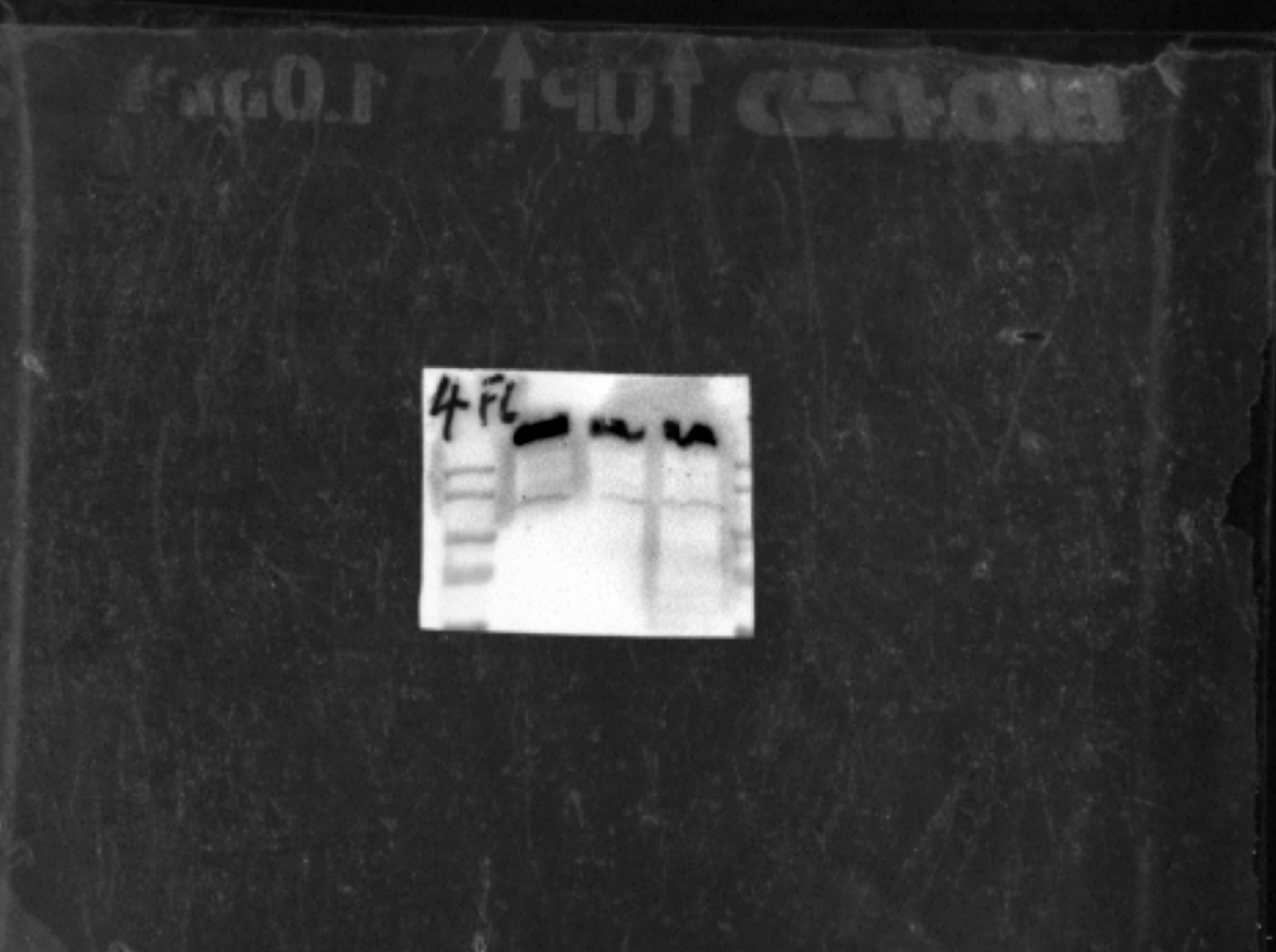

Supplement: Supplemental Information 32 [file peerj-14-21375-s032.zip › Figure 4I WB RAW sh-KLHL40 FLNC/FLNC-2 sh-KLHL40+MARK.tif]

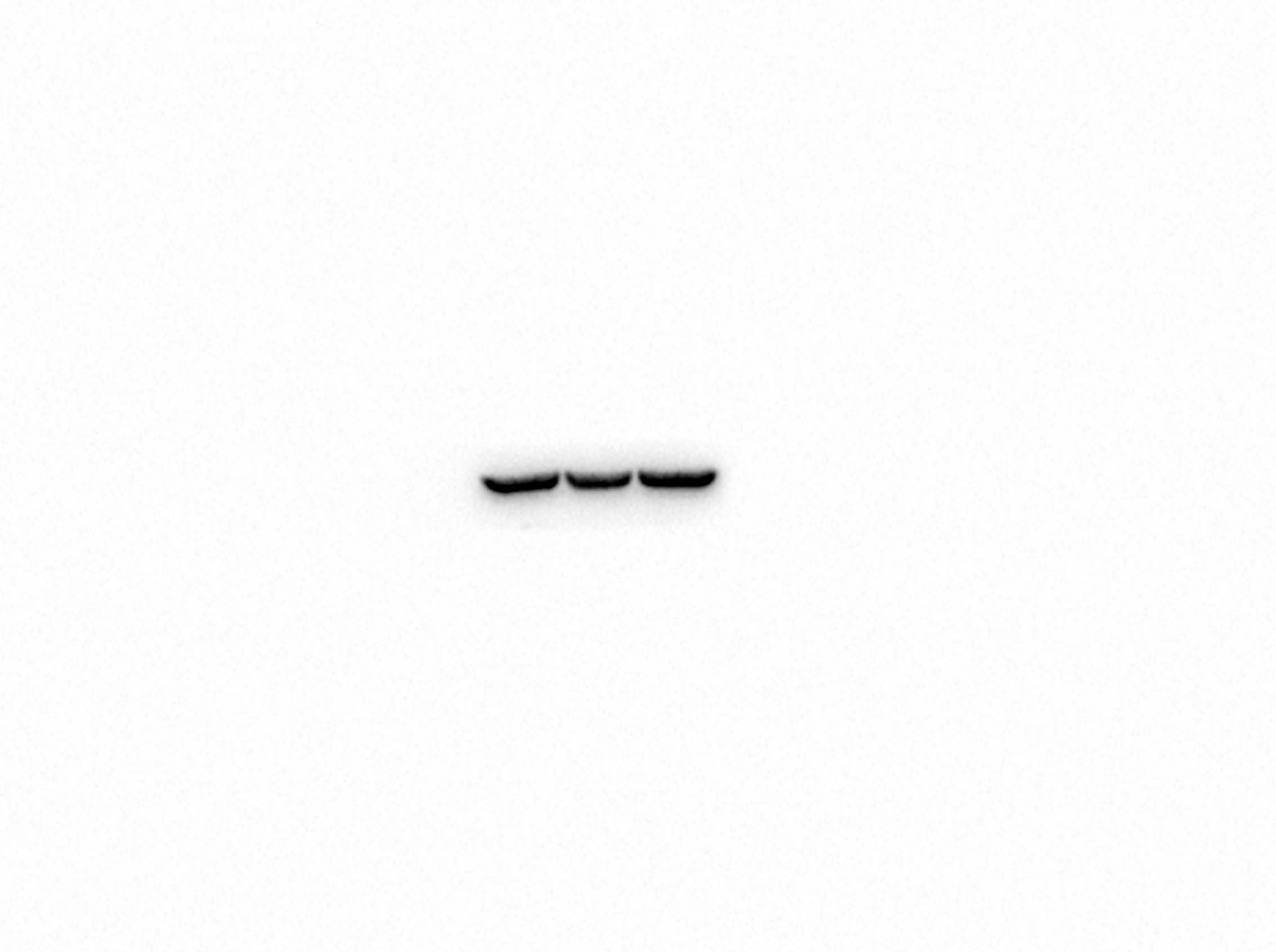

Supplement: Supplemental Information 32 [file peerj-14-21375-s032.zip › Figure 4I WB RAW sh-KLHL40 FLNC/FLNC-2 sh-KLHL40-ACTB.tif]

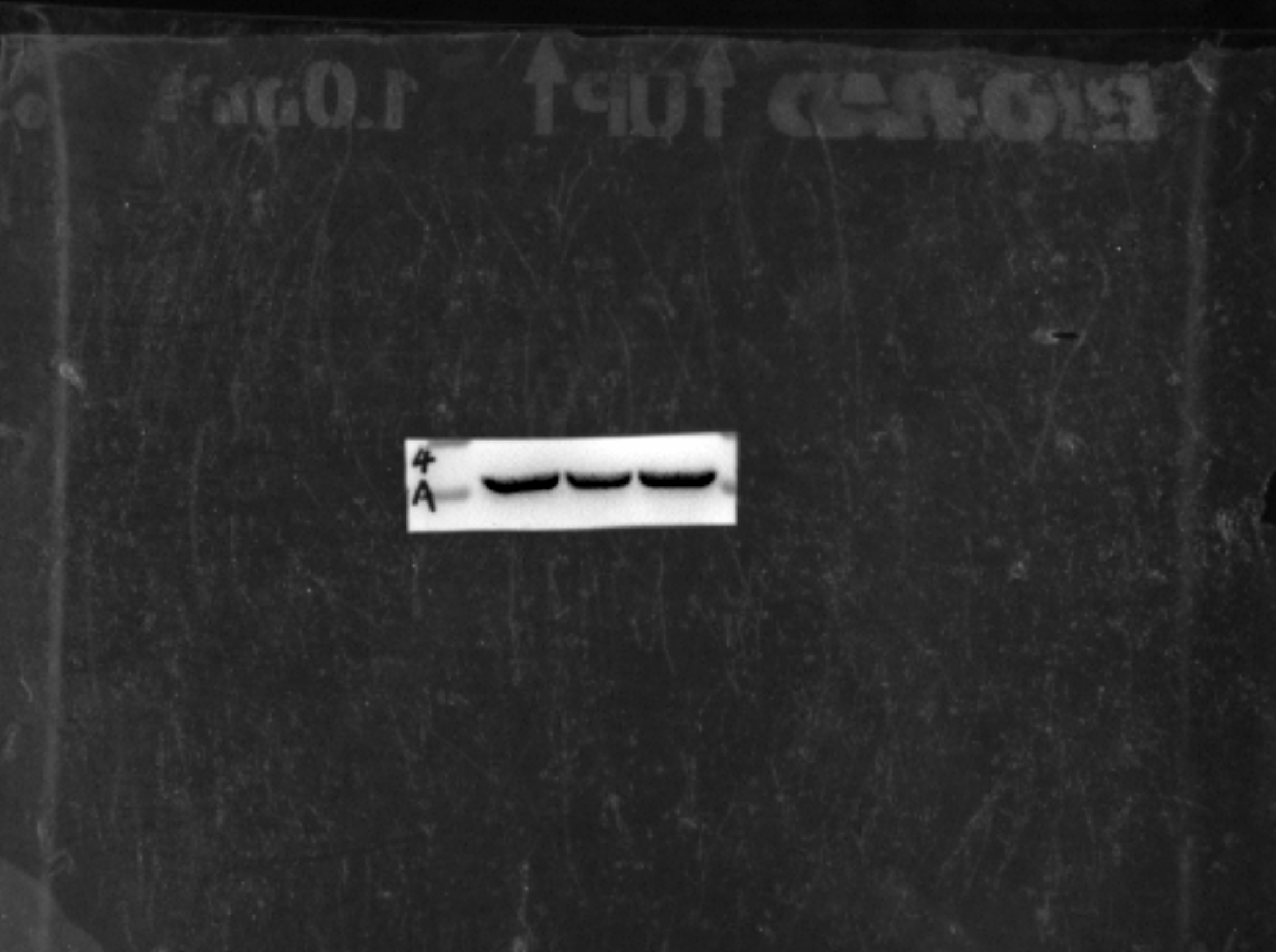

Supplement: Supplemental Information 32 [file peerj-14-21375-s032.zip › Figure 4I WB RAW sh-KLHL40 FLNC/FLNC-2 sh-KLHL40-ACTB+MARK.tif]

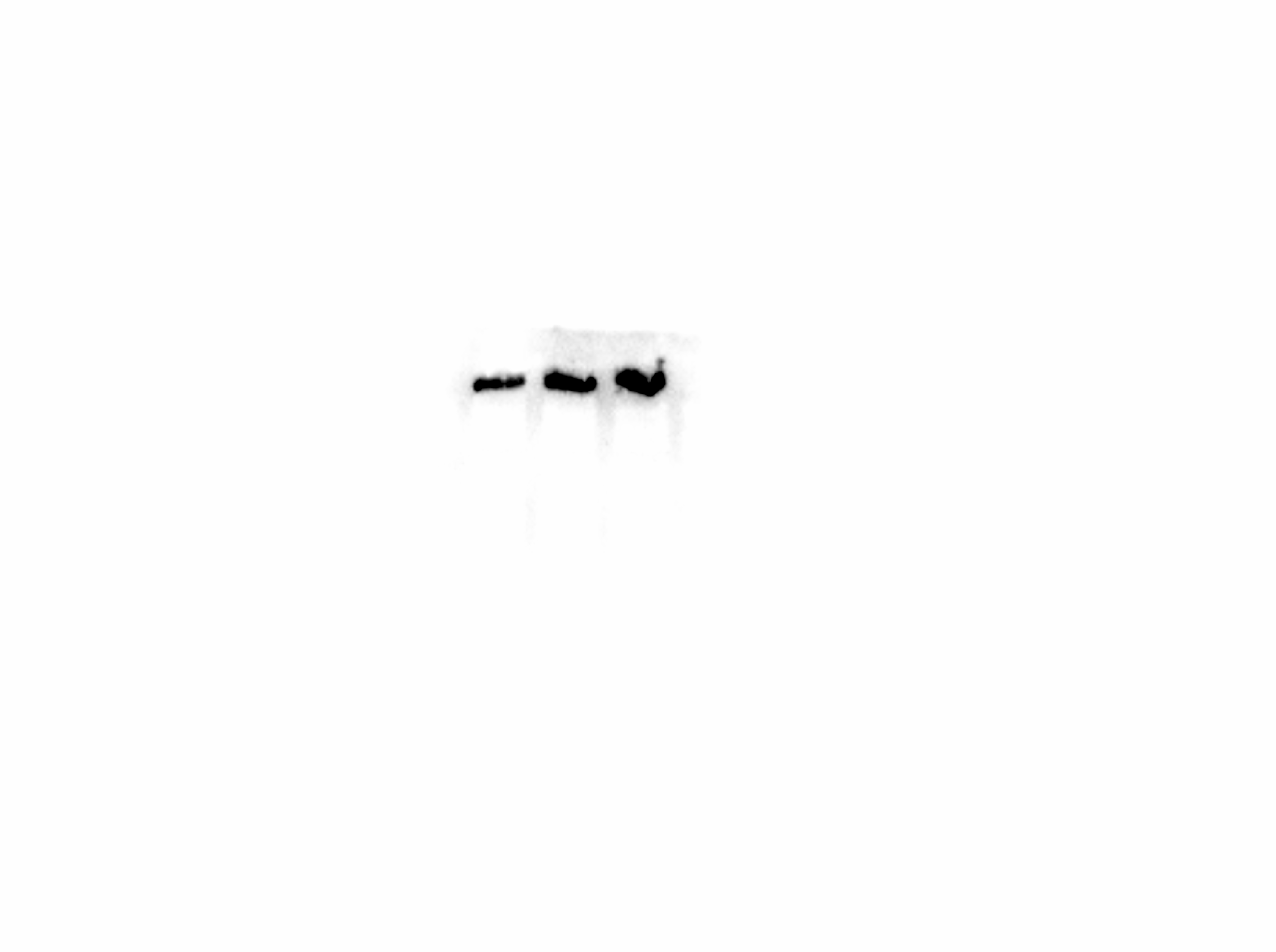

Supplement: Supplemental Information 32 [file peerj-14-21375-s032.zip › Figure 4I WB RAW sh-KLHL40 FLNC/FLNC-3 sh-KLHL40.tif]

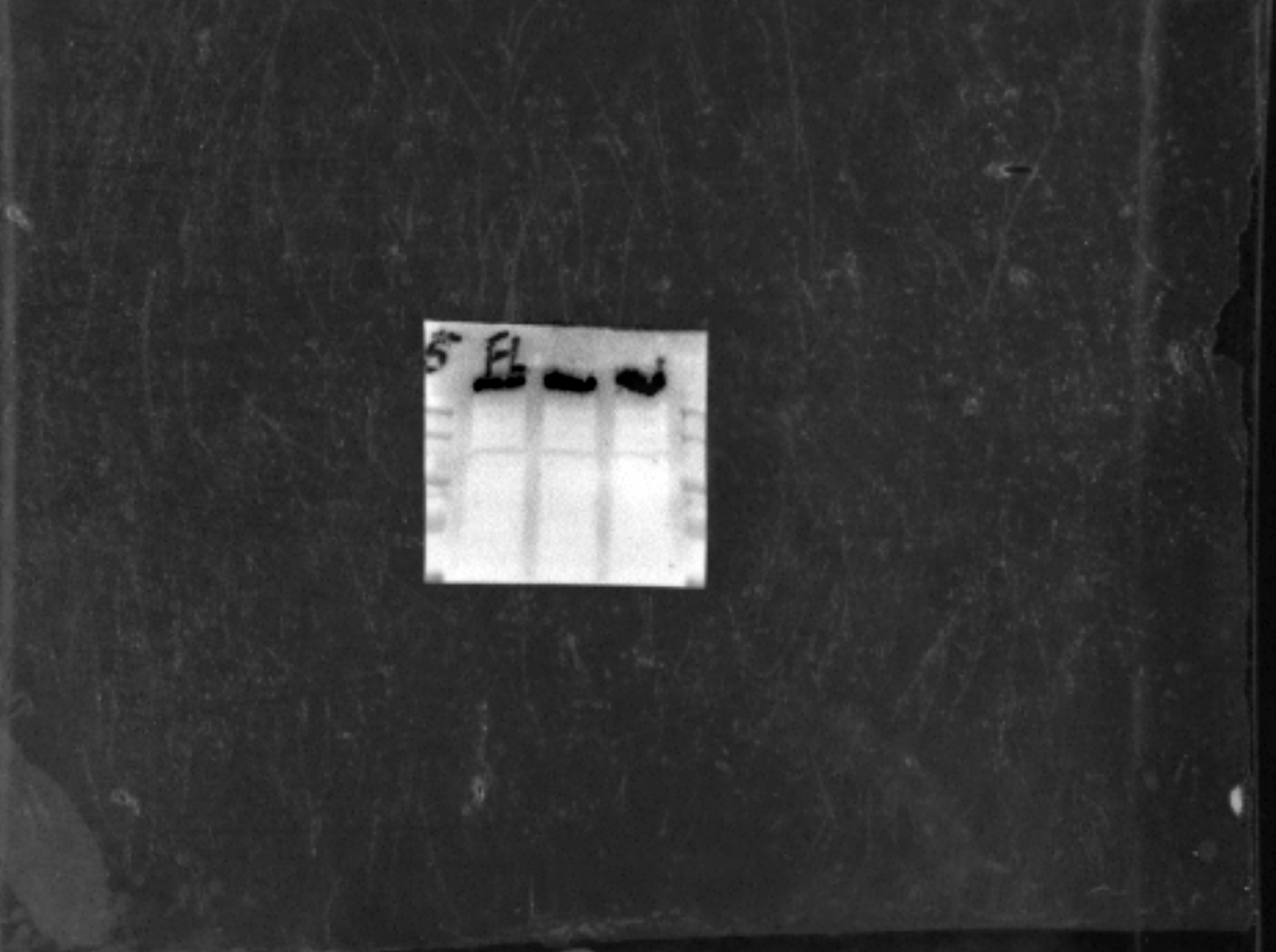

Supplement: Supplemental Information 32 [file peerj-14-21375-s032.zip › Figure 4I WB RAW sh-KLHL40 FLNC/FLNC-3 sh-KLHL40+MARK.tif]

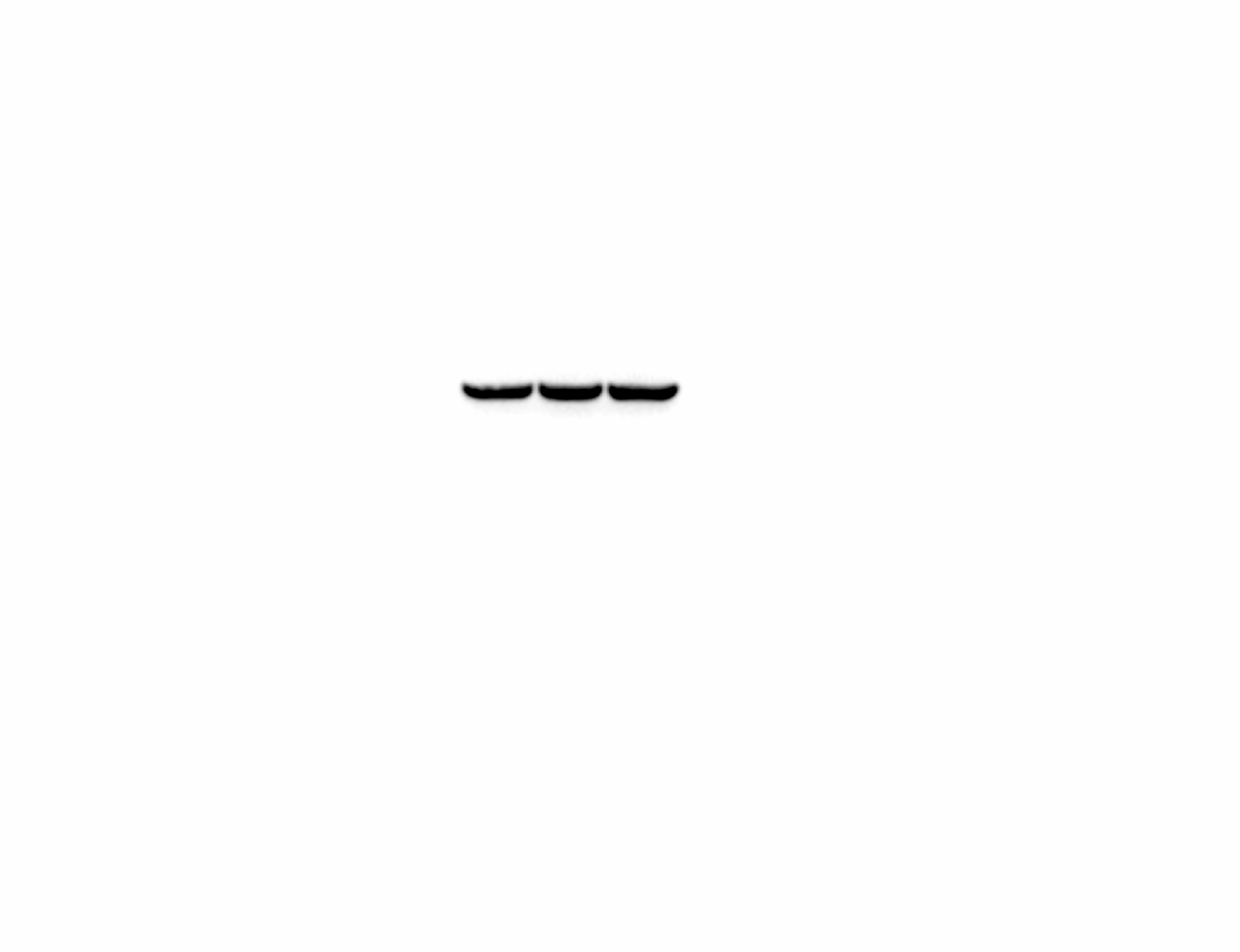

Supplement: Supplemental Information 32 [file peerj-14-21375-s032.zip › Figure 4I WB RAW sh-KLHL40 FLNC/FLNC-3 sh-KLHL40-ACTB.tif]

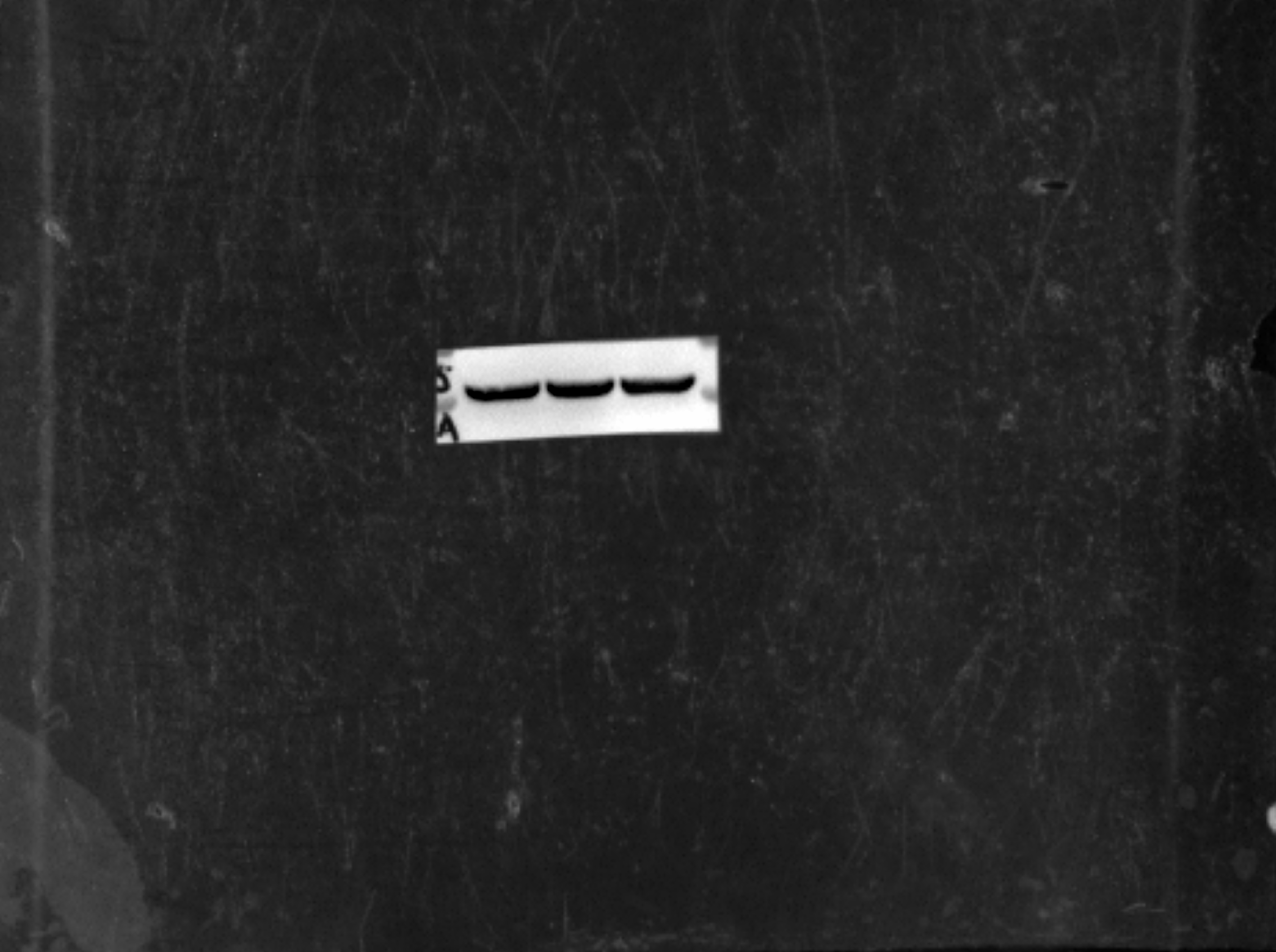

Supplement: Supplemental Information 32 [file peerj-14-21375-s032.zip › Figure 4I WB RAW sh-KLHL40 FLNC/FLNC-3 sh-KLHL40-ACTB+MARK.tif]

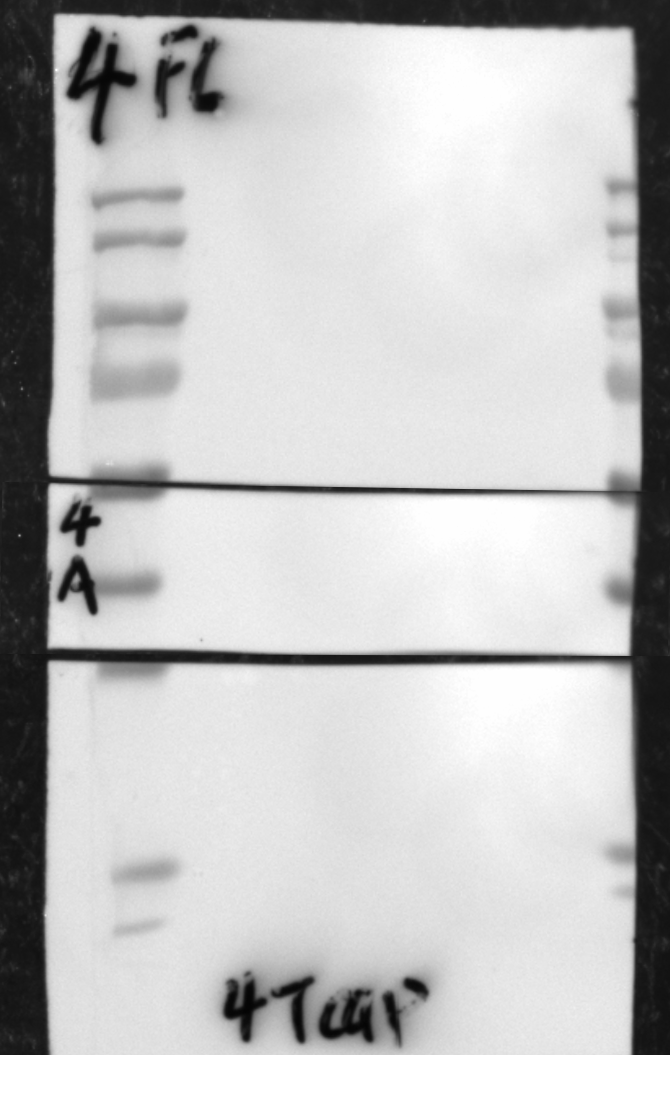

Supplement: Supplemental Information 32 [file peerj-14-21375-s032.zip › Figure 4I WB RAW sh-KLHL40 FLNC/TOTAL-2.tif]

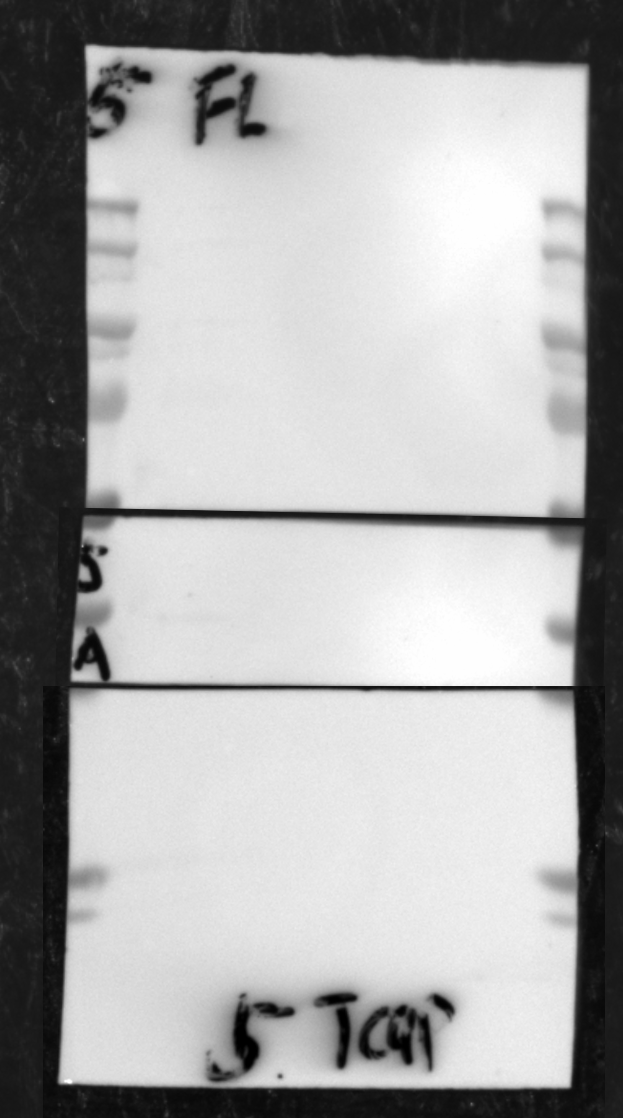

Supplement: Supplemental Information 32 [file peerj-14-21375-s032.zip › Figure 4I WB RAW sh-KLHL40 FLNC/TOTAL-3.tif]

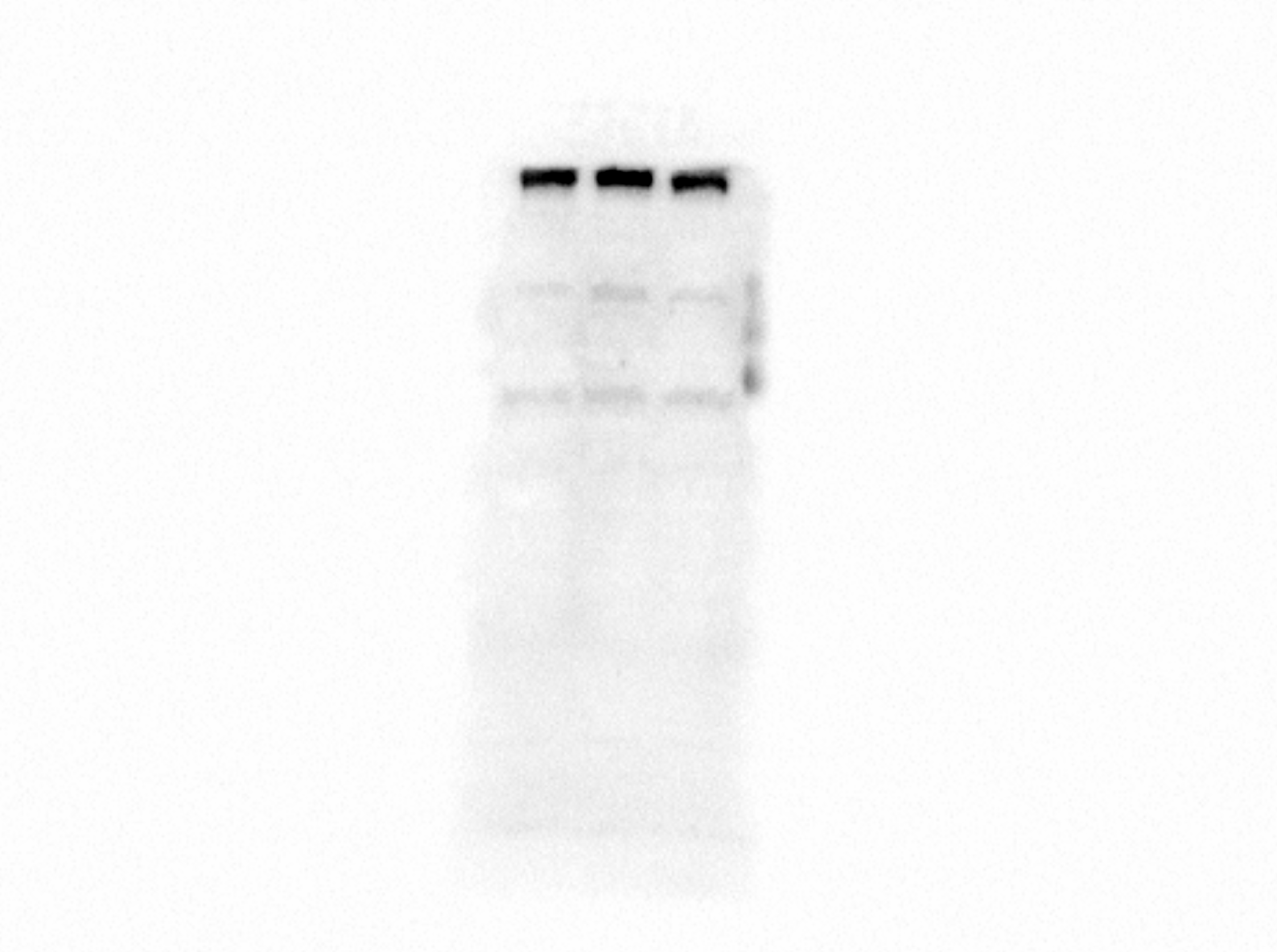

Supplement: Supplemental Information 33 [file peerj-14-21375-s033.zip › Figure 4J WB RAW oe-KLHL40 FLNC/FLNC-1 oe-KLHL40.tif]

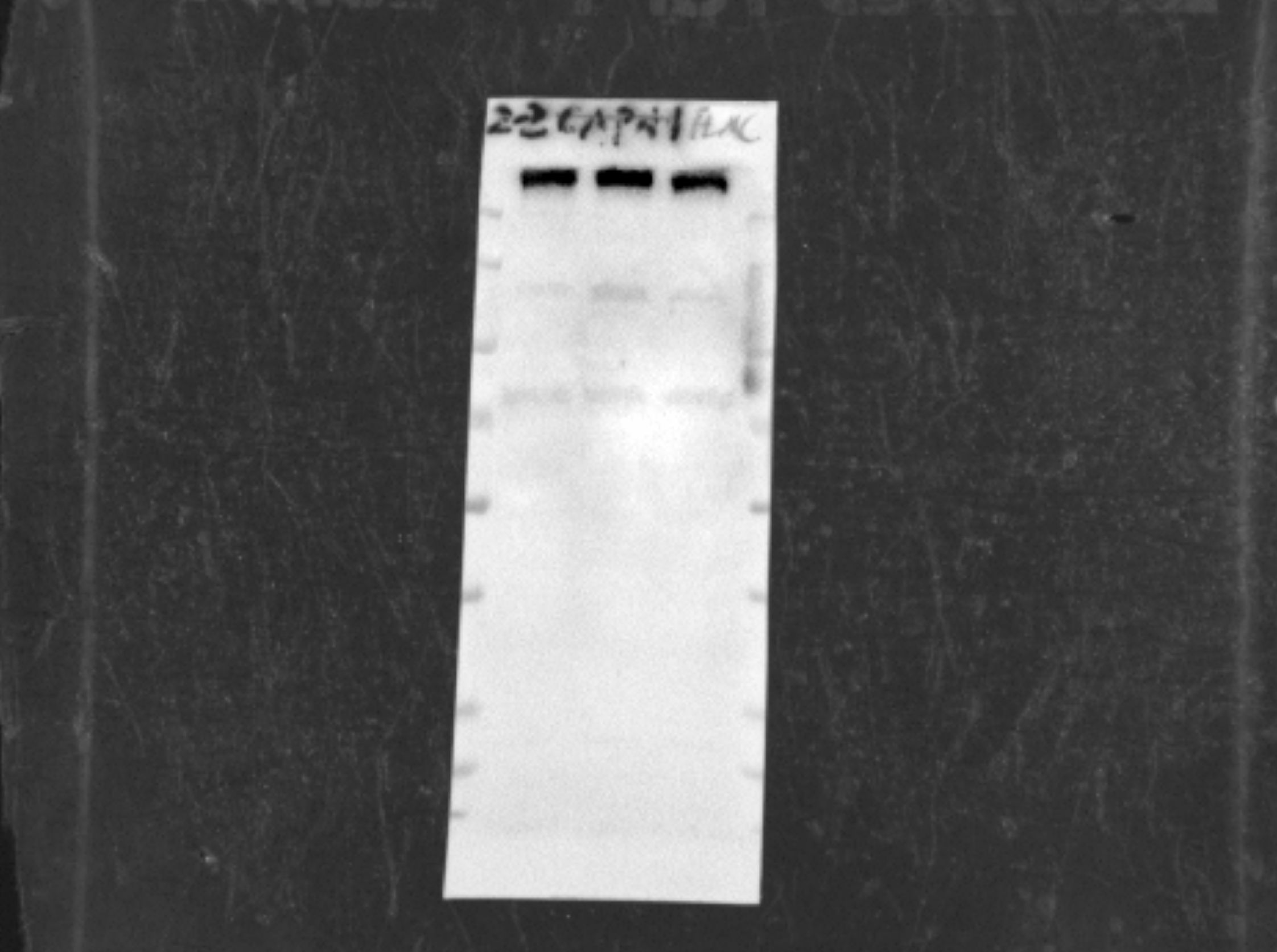

Supplement: Supplemental Information 33 [file peerj-14-21375-s033.zip › Figure 4J WB RAW oe-KLHL40 FLNC/FLNC-1 oe-KLHL40+MARK.tif]
