# Supplementary figures and images for: Post-infarction KLHL40-mediated regulation of cardiac sarcomeric integrity and function (part 3 of 5)
Source: PeerJ. 2026 Jun 5;14:e21375. doi: 10.7717/peerj.21375 (PMC13245431; doi:10.7717/peerj.21375)

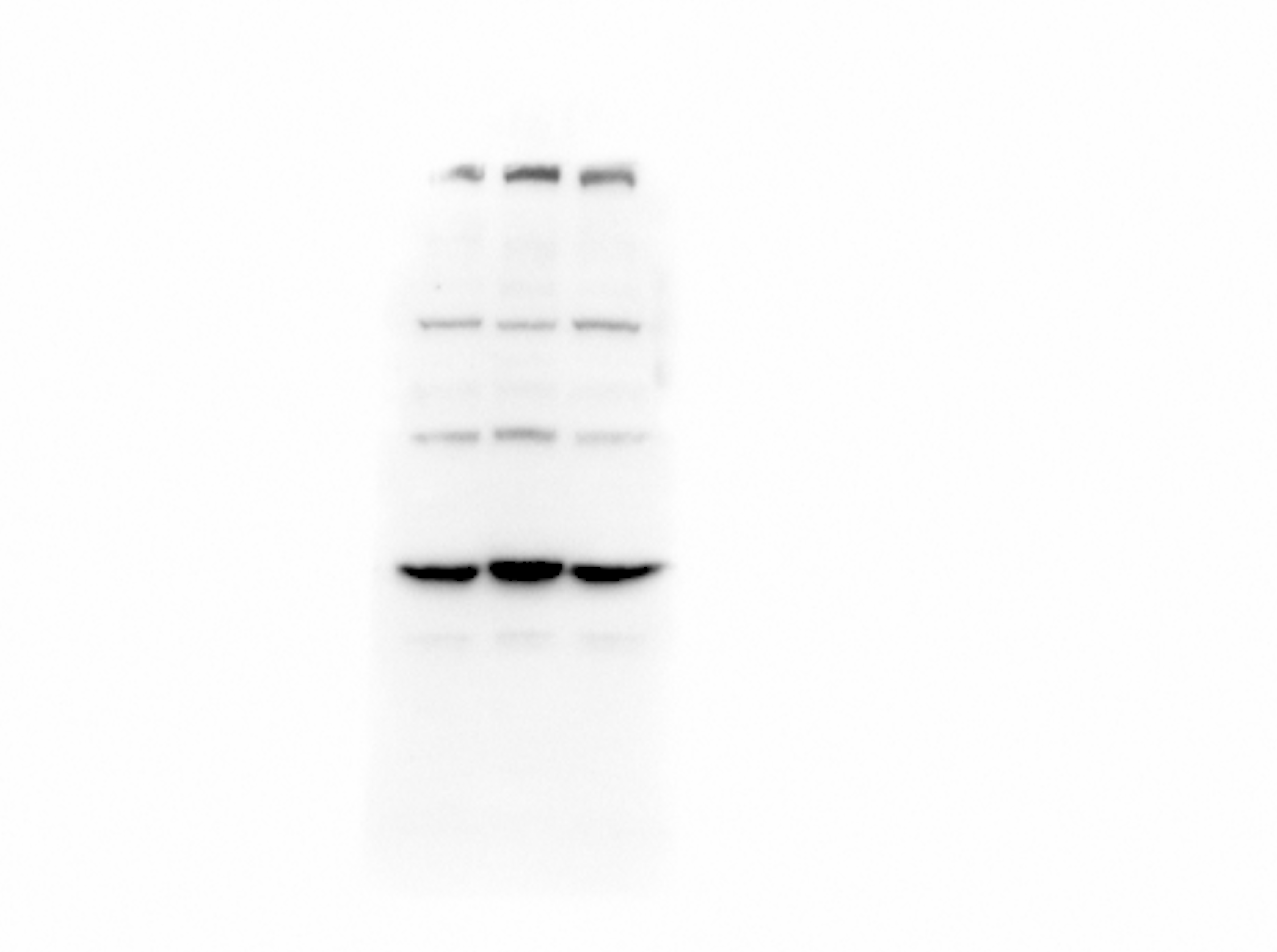

Supplement: Supplemental Information 33 [file peerj-14-21375-s033.zip › Figure 4J WB RAW oe-KLHL40 FLNC/FLNC-1 oe-KLHL40-ACTB.tif]

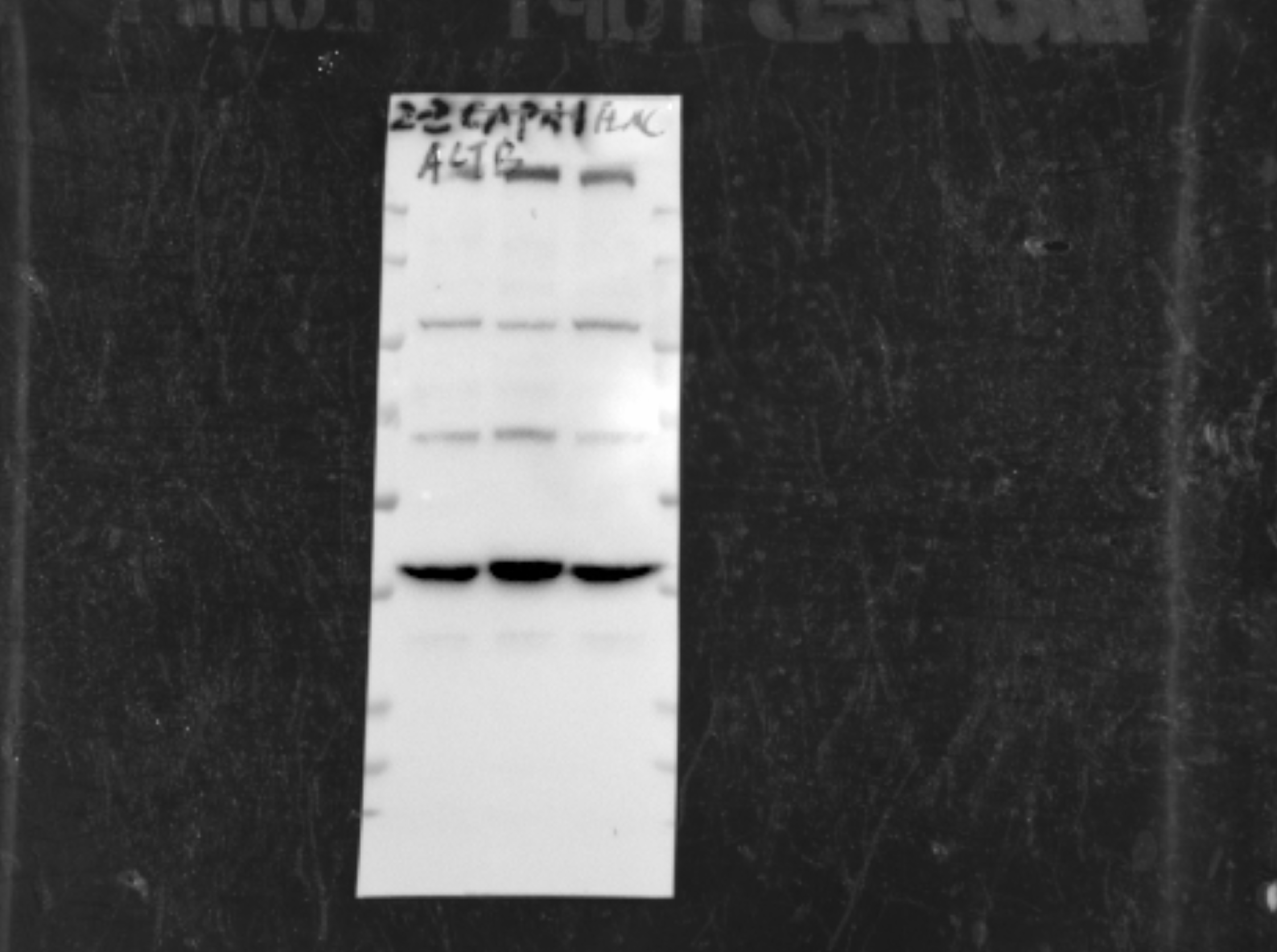

Supplement: Supplemental Information 33 [file peerj-14-21375-s033.zip › Figure 4J WB RAW oe-KLHL40 FLNC/FLNC-1 oe-KLHL40-ACTB+MARK.tif]

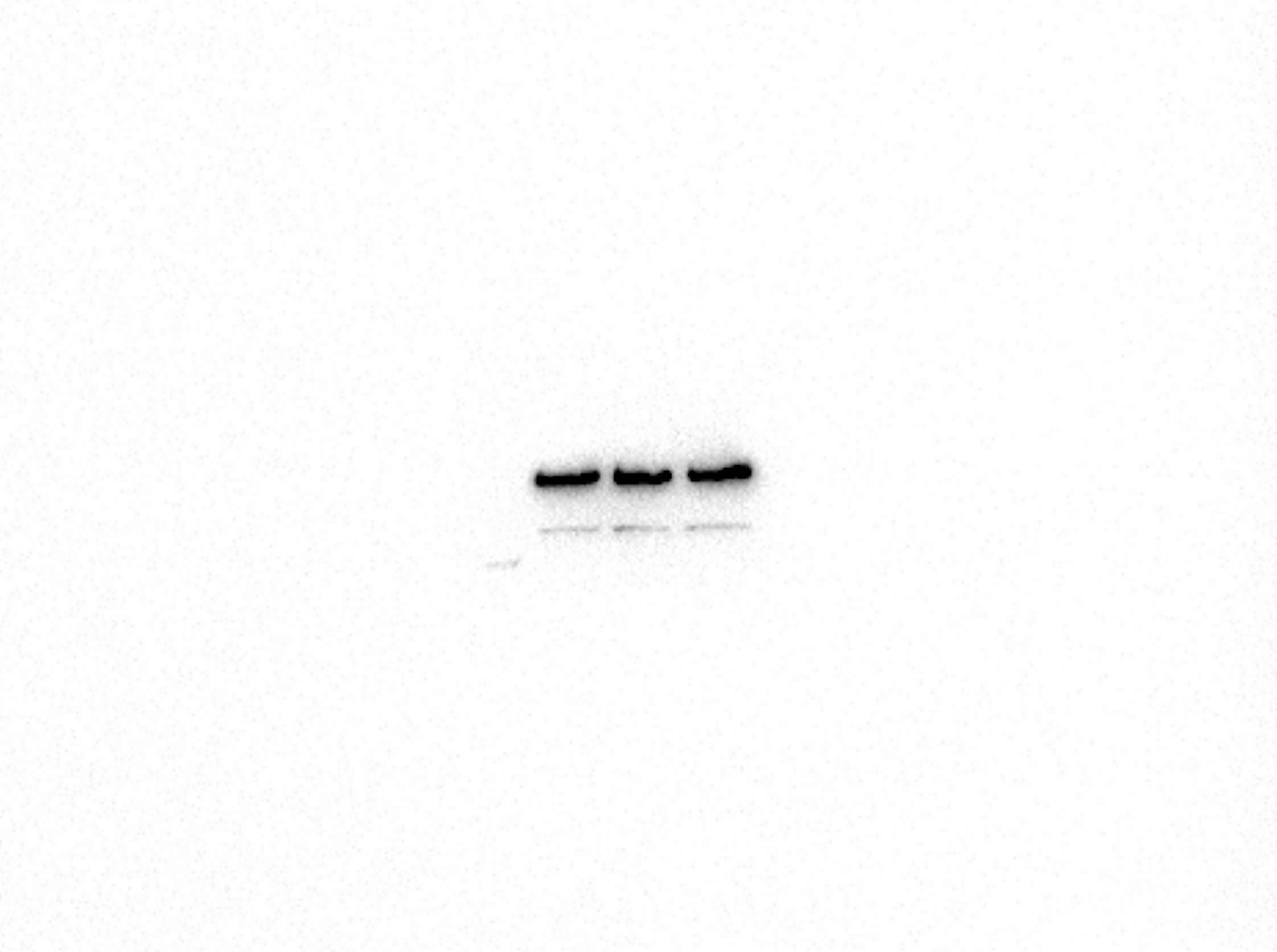

Supplement: Supplemental Information 33 [file peerj-14-21375-s033.zip › Figure 4J WB RAW oe-KLHL40 FLNC/FLNC-2 oe-KLHL40.tif]

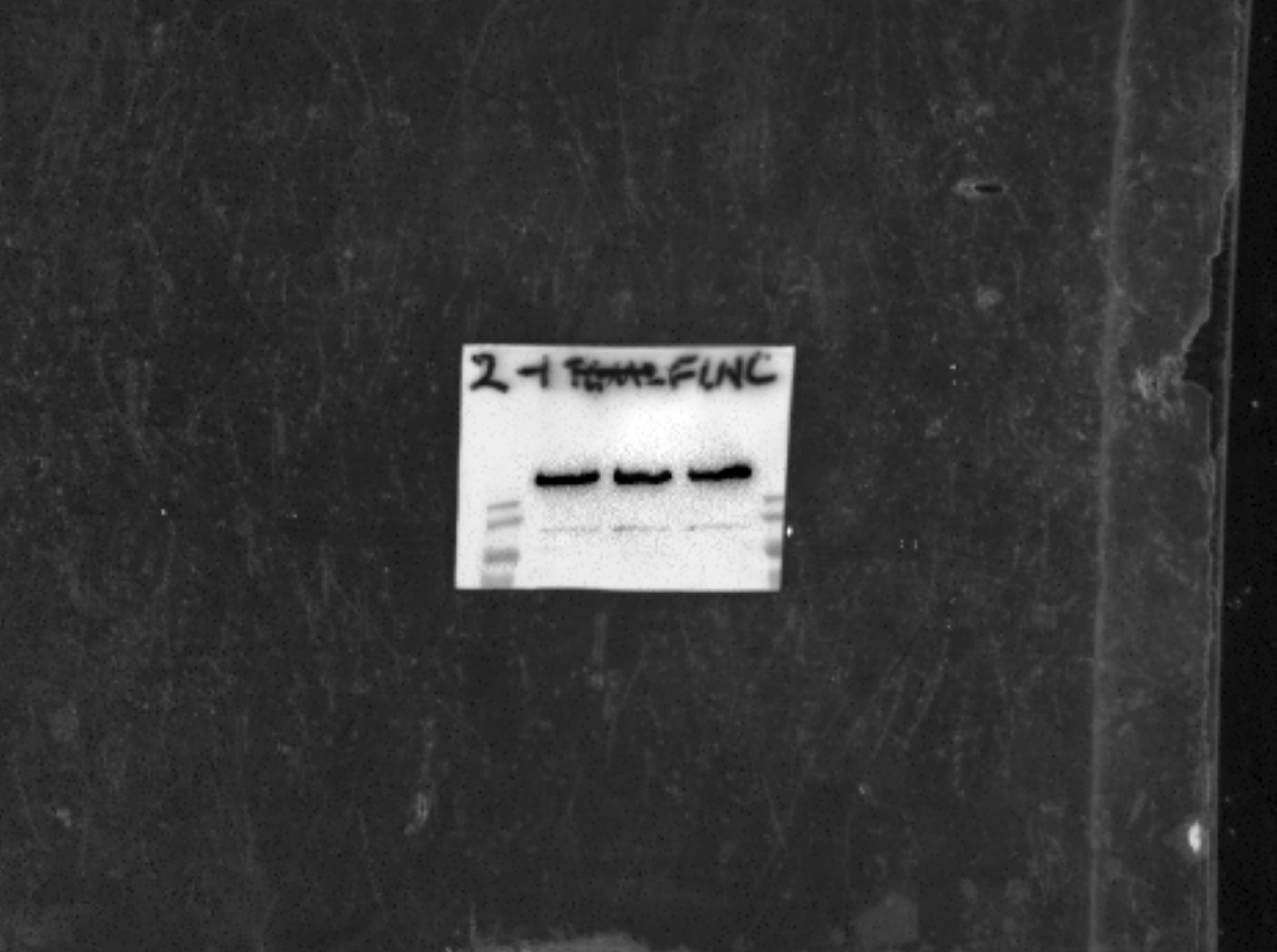

Supplement: Supplemental Information 33 [file peerj-14-21375-s033.zip › Figure 4J WB RAW oe-KLHL40 FLNC/FLNC-2 oe-KLHL40+MARK.tif]

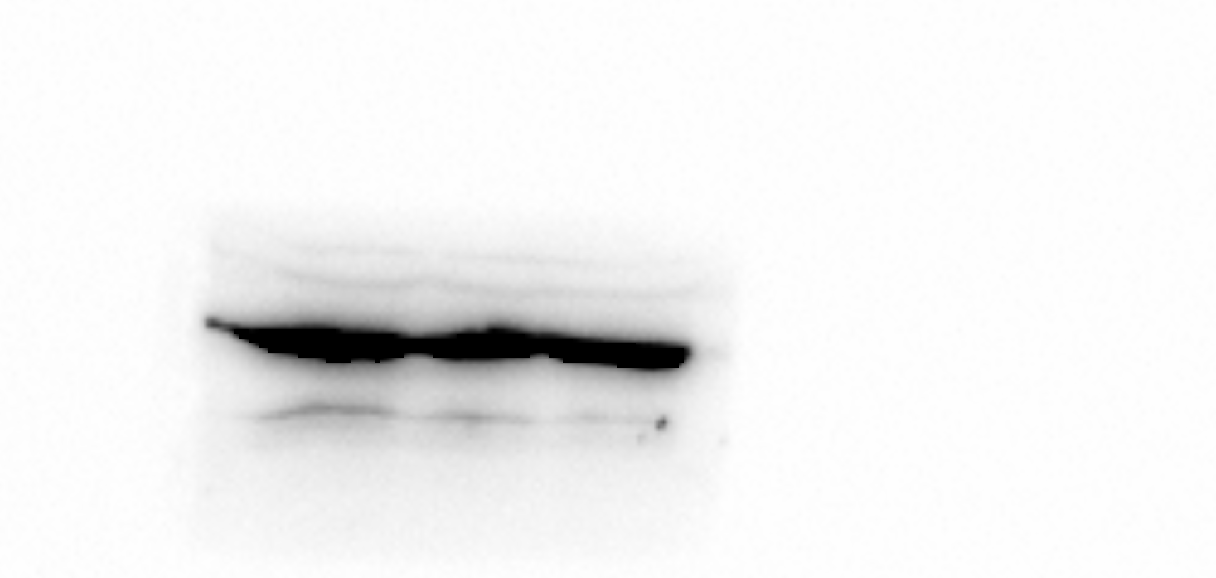

Supplement: Supplemental Information 33 [file peerj-14-21375-s033.zip › Figure 4J WB RAW oe-KLHL40 FLNC/FLNC-2 oe-KLHL40-ACTB.tif]

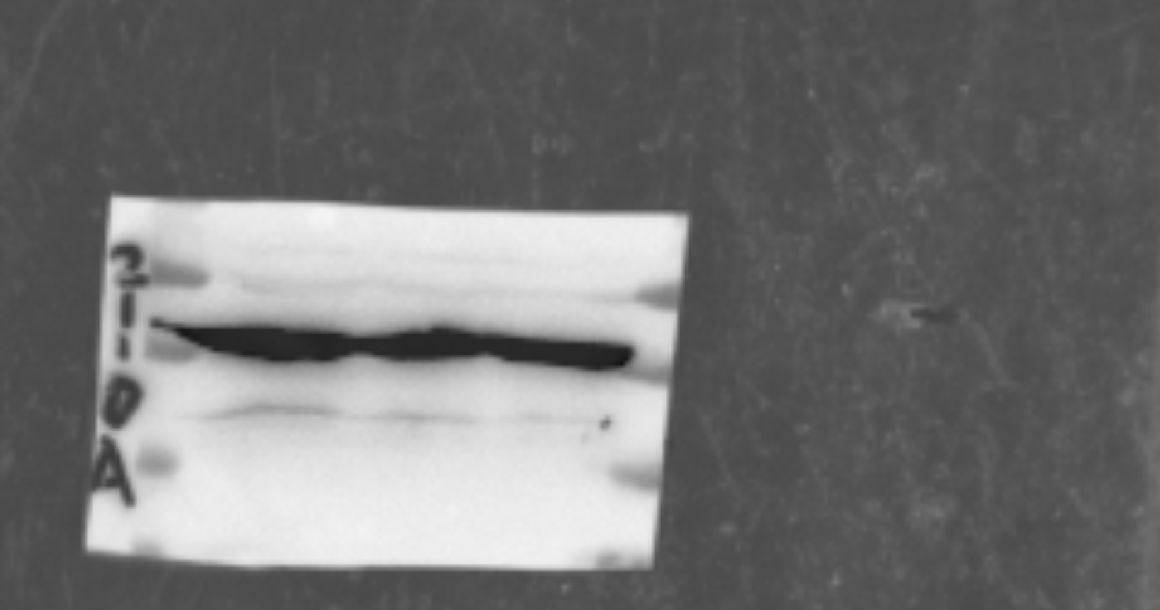

Supplement: Supplemental Information 33 [file peerj-14-21375-s033.zip › Figure 4J WB RAW oe-KLHL40 FLNC/FLNC-2 oe-KLHL40-ACTB+MARK.tif]

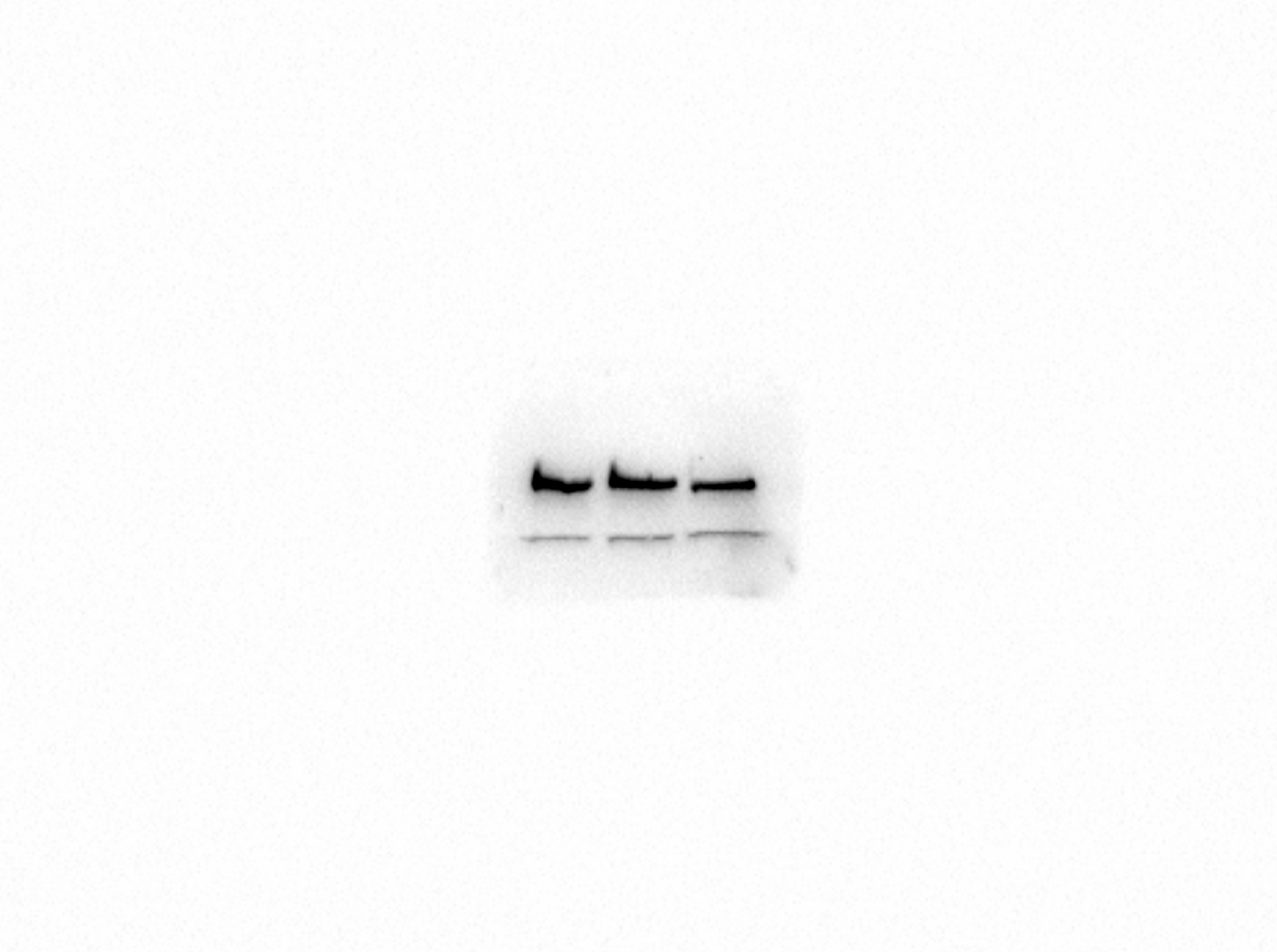

Supplement: Supplemental Information 33 [file peerj-14-21375-s033.zip › Figure 4J WB RAW oe-KLHL40 FLNC/FLNC-3 oe-KLHL40.tif]

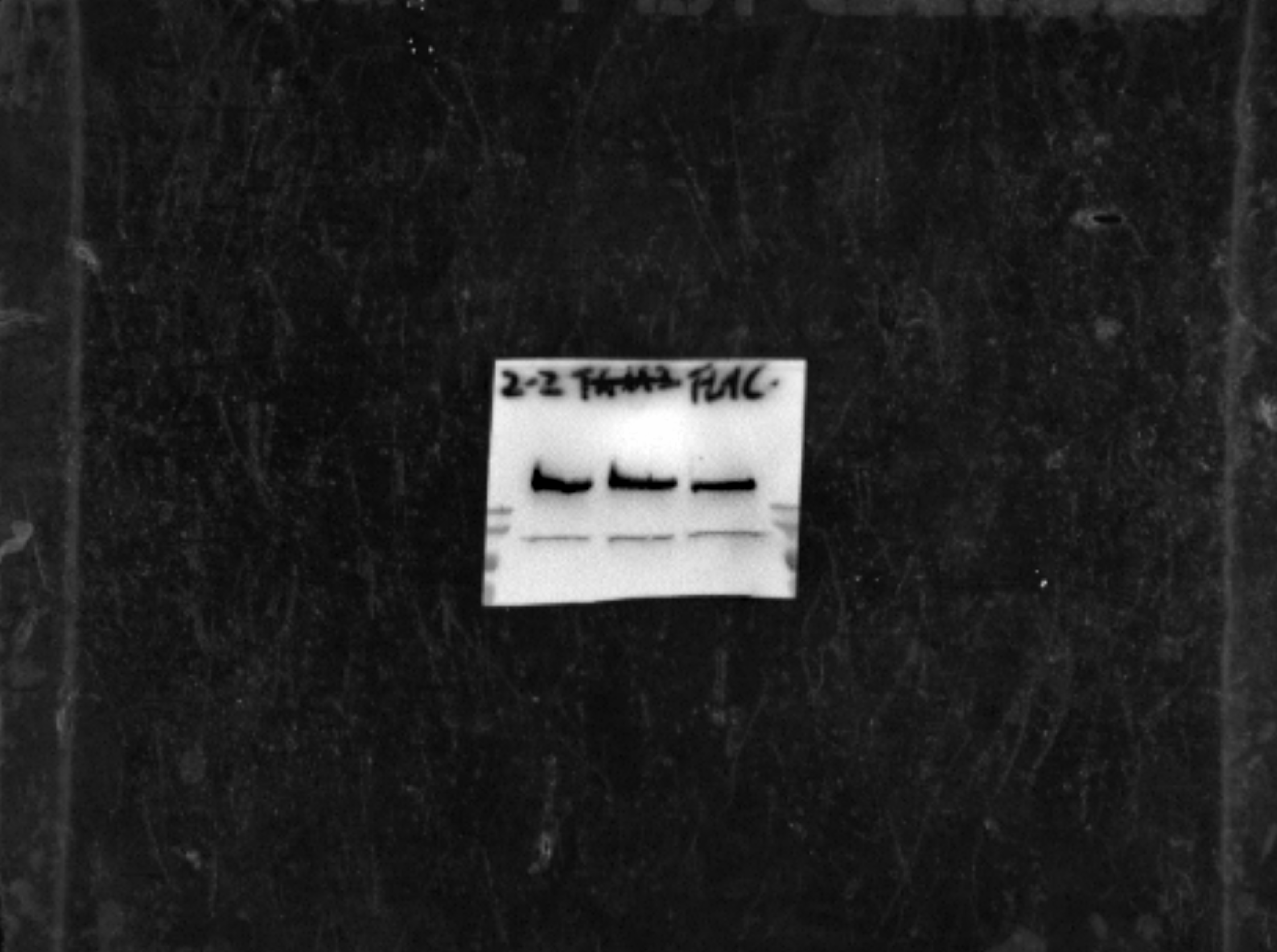

Supplement: Supplemental Information 33 [file peerj-14-21375-s033.zip › Figure 4J WB RAW oe-KLHL40 FLNC/FLNC-3 oe-KLHL40+MARK.tif]

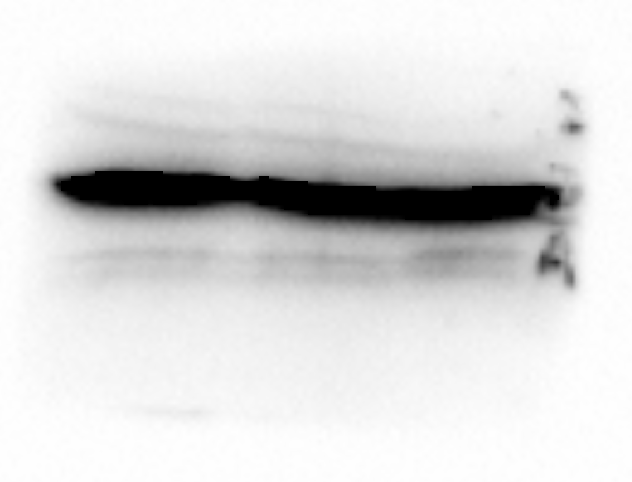

Supplement: Supplemental Information 33 [file peerj-14-21375-s033.zip › Figure 4J WB RAW oe-KLHL40 FLNC/FLNC-3 oe-KLHL40-ACTB.tif]

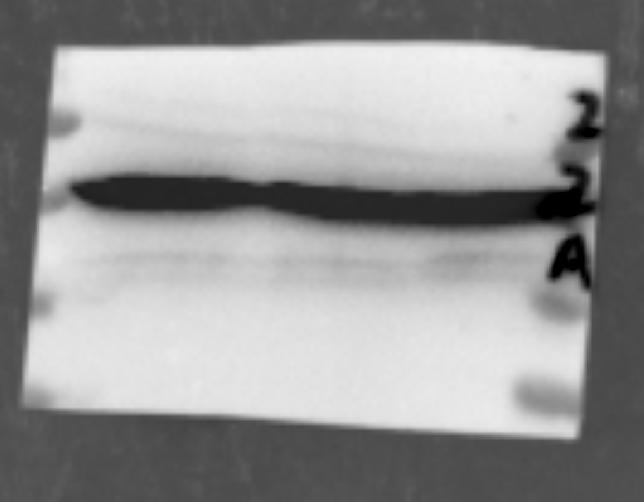

Supplement: Supplemental Information 33 [file peerj-14-21375-s033.zip › Figure 4J WB RAW oe-KLHL40 FLNC/FLNC-3 oe-KLHL40-ACTB+MARK.tif]

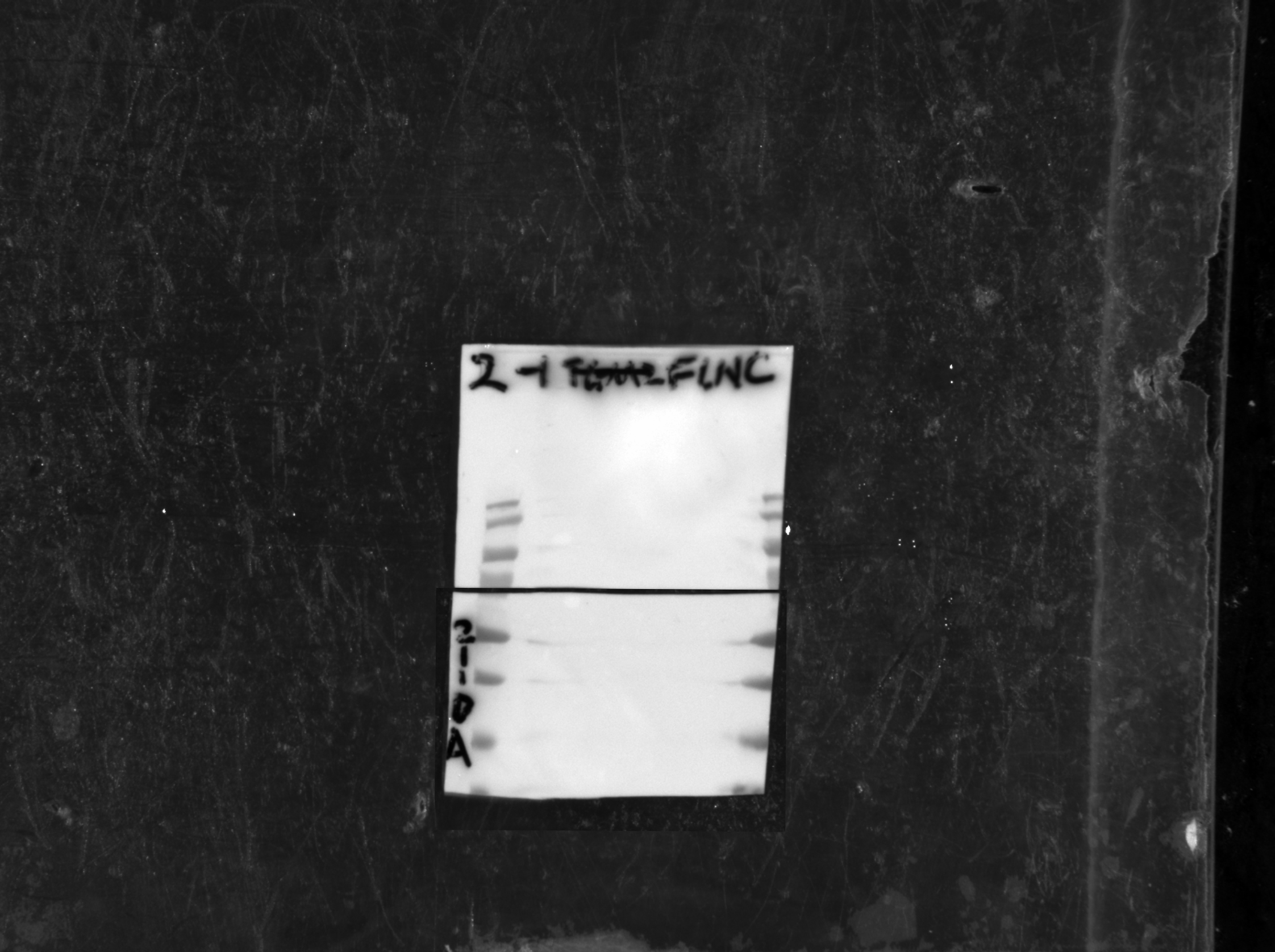

Supplement: Supplemental Information 33 [file peerj-14-21375-s033.zip › Figure 4J WB RAW oe-KLHL40 FLNC/TOTAL-2.tif]

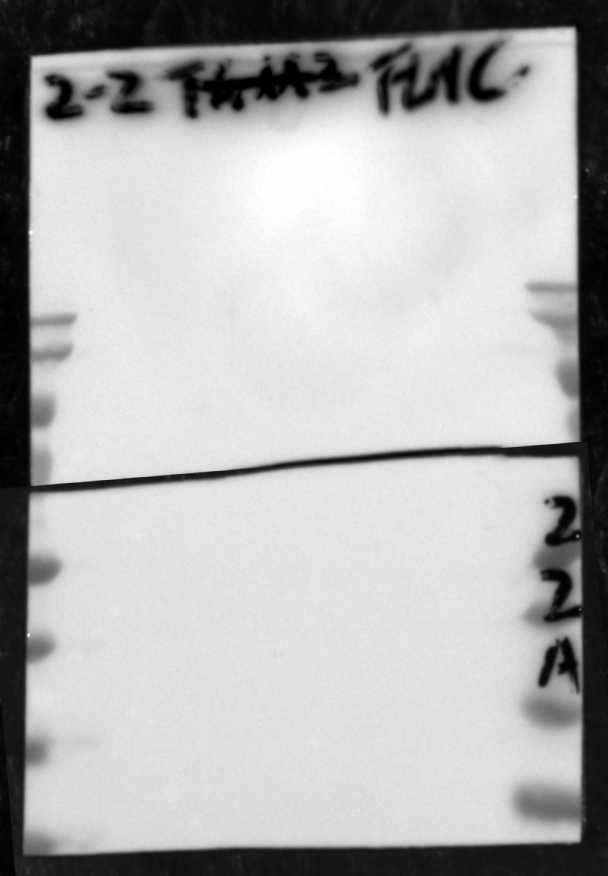

Supplement: Supplemental Information 33 [file peerj-14-21375-s033.zip › Figure 4J WB RAW oe-KLHL40 FLNC/TOTAL-3.tif]

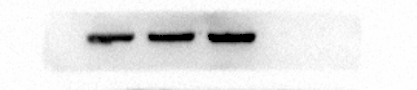

Supplement: Supplemental Information 35 [file peerj-14-21375-s035.zip › Figure 5A WB RAW SH-KLHL40 CAST/1-ACTIN.png]

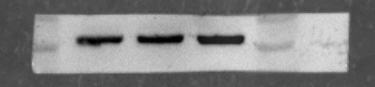

Supplement: Supplemental Information 35 [file peerj-14-21375-s035.zip › Figure 5A WB RAW SH-KLHL40 CAST/1-ACTIN-MARKER.png]

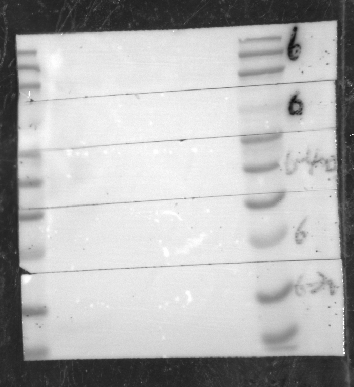

Supplement: Supplemental Information 35 [file peerj-14-21375-s035.zip › Figure 5A WB RAW SH-KLHL40 CAST/1ALL.png]

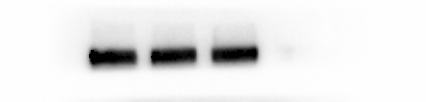

Supplement: Supplemental Information 35 [file peerj-14-21375-s035.zip › Figure 5A WB RAW SH-KLHL40 CAST/1-calpastatin.png]

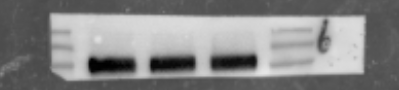

Supplement: Supplemental Information 35 [file peerj-14-21375-s035.zip › Figure 5A WB RAW SH-KLHL40 CAST/1-calpastatin-MARKER.png]

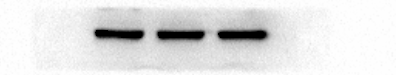

Supplement: Supplemental Information 35 [file peerj-14-21375-s035.zip › Figure 5A WB RAW SH-KLHL40 CAST/2-ACTIN.png]

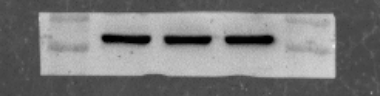

Supplement: Supplemental Information 35 [file peerj-14-21375-s035.zip › Figure 5A WB RAW SH-KLHL40 CAST/2-ACTIN-MARKER.png]

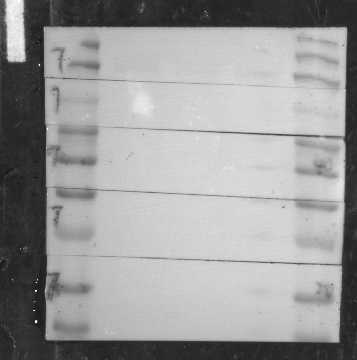

Supplement: Supplemental Information 35 [file peerj-14-21375-s035.zip › Figure 5A WB RAW SH-KLHL40 CAST/2ALL.png]

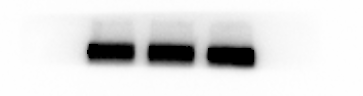

Supplement: Supplemental Information 35 [file peerj-14-21375-s035.zip › Figure 5A WB RAW SH-KLHL40 CAST/2-calpastatin.png]

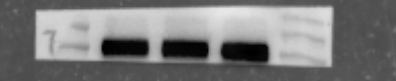

Supplement: Supplemental Information 35 [file peerj-14-21375-s035.zip › Figure 5A WB RAW SH-KLHL40 CAST/2-calpastatin-MARKER.png]

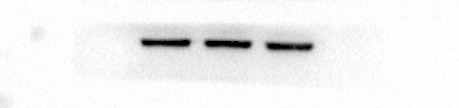

Supplement: Supplemental Information 35 [file peerj-14-21375-s035.zip › Figure 5A WB RAW SH-KLHL40 CAST/3-ACTIN.png]

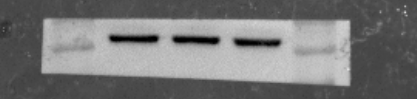

Supplement: Supplemental Information 35 [file peerj-14-21375-s035.zip › Figure 5A WB RAW SH-KLHL40 CAST/3-ACTIN-MARKER.png]

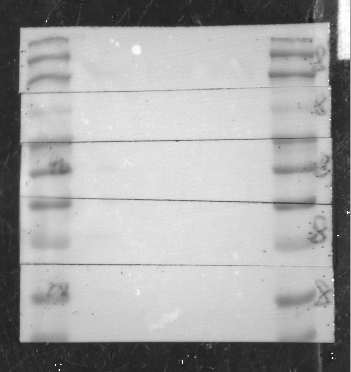

Supplement: Supplemental Information 35 [file peerj-14-21375-s035.zip › Figure 5A WB RAW SH-KLHL40 CAST/3ALL.png]

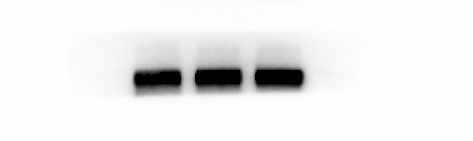

Supplement: Supplemental Information 35 [file peerj-14-21375-s035.zip › Figure 5A WB RAW SH-KLHL40 CAST/3-calpastatin.png]

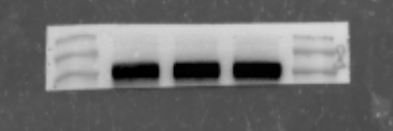

Supplement: Supplemental Information 35 [file peerj-14-21375-s035.zip › Figure 5A WB RAW SH-KLHL40 CAST/3-calpastatin-MARKER.png]

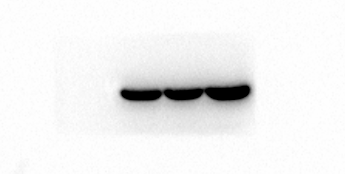

Supplement: Supplemental Information 36 [file peerj-14-21375-s036.zip › Figure 5B WB RAW OE-KLHL40 CAST/1ACTIN.png]

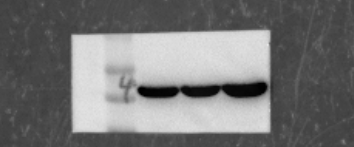

Supplement: Supplemental Information 36 [file peerj-14-21375-s036.zip › Figure 5B WB RAW OE-KLHL40 CAST/1ACTIN+MARKER.png]

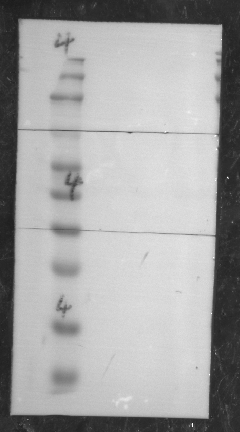

Supplement: Supplemental Information 36 [file peerj-14-21375-s036.zip › Figure 5B WB RAW OE-KLHL40 CAST/1ALL.png]

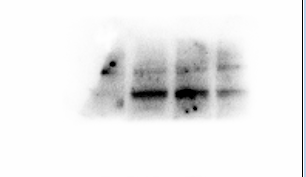

Supplement: Supplemental Information 36 [file peerj-14-21375-s036.zip › Figure 5B WB RAW OE-KLHL40 CAST/1calpastatin.png]

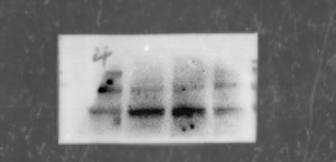

Supplement: Supplemental Information 36 [file peerj-14-21375-s036.zip › Figure 5B WB RAW OE-KLHL40 CAST/1calpastatin+MARKER.png]

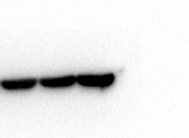

Supplement: Supplemental Information 36 [file peerj-14-21375-s036.zip › Figure 5B WB RAW OE-KLHL40 CAST/2ACTIN.png]

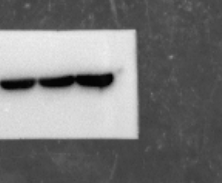

Supplement: Supplemental Information 36 [file peerj-14-21375-s036.zip › Figure 5B WB RAW OE-KLHL40 CAST/2ACTIN+MARKER.png]

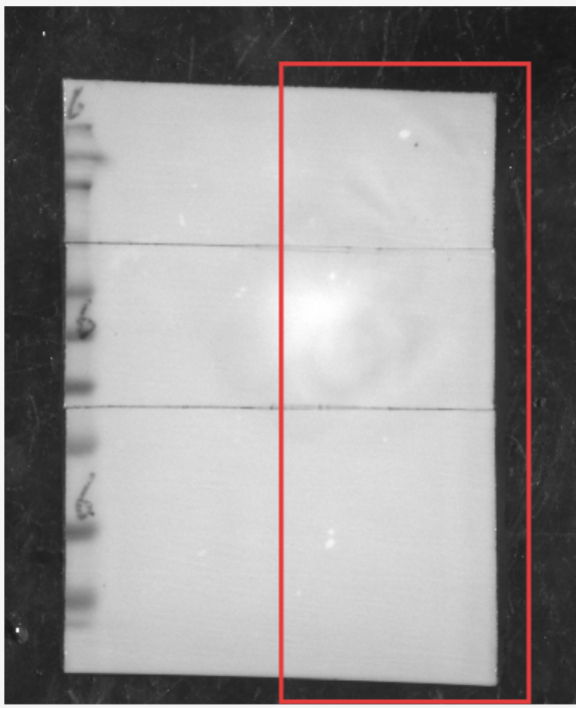

Supplement: Supplemental Information 36 [file peerj-14-21375-s036.zip › Figure 5B WB RAW OE-KLHL40 CAST/2ALL.png]

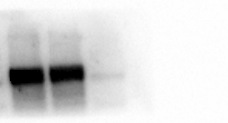

Supplement: Supplemental Information 36 [file peerj-14-21375-s036.zip › Figure 5B WB RAW OE-KLHL40 CAST/2calpastatin+.png]

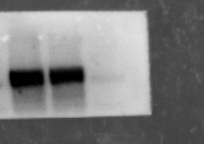

Supplement: Supplemental Information 36 [file peerj-14-21375-s036.zip › Figure 5B WB RAW OE-KLHL40 CAST/2calpastatin+MARKER.png]

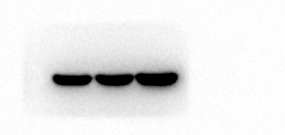

Supplement: Supplemental Information 36 [file peerj-14-21375-s036.zip › Figure 5B WB RAW OE-KLHL40 CAST/3ACTIN.png]

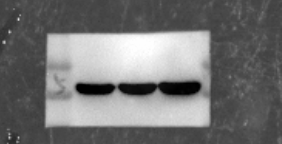

Supplement: Supplemental Information 36 [file peerj-14-21375-s036.zip › Figure 5B WB RAW OE-KLHL40 CAST/3ACTIN+MARKER.png]

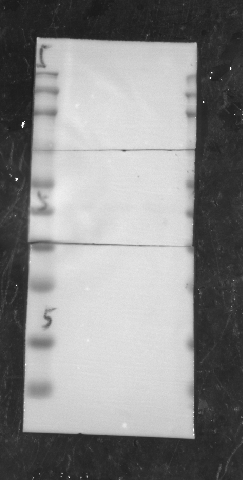

Supplement: Supplemental Information 36 [file peerj-14-21375-s036.zip › Figure 5B WB RAW OE-KLHL40 CAST/3ALL.png]

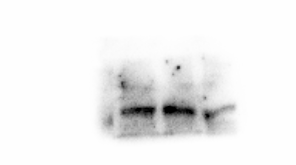

Supplement: Supplemental Information 36 [file peerj-14-21375-s036.zip › Figure 5B WB RAW OE-KLHL40 CAST/3calpastatin.png]

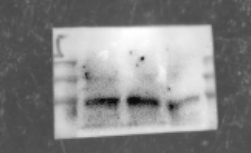

Supplement: Supplemental Information 36 [file peerj-14-21375-s036.zip › Figure 5B WB RAW OE-KLHL40 CAST/3calpastatin+MARKER.png]

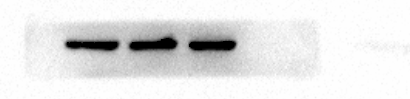

Supplement: Supplemental Information 37 [file peerj-14-21375-s037.zip › Figure 5C WB RAW SH-KLHL40 CAPN1 CAPN2/CAPN1/1-ACTIN.png]

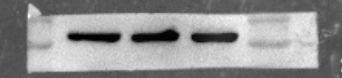

Supplement: Supplemental Information 37 [file peerj-14-21375-s037.zip › Figure 5C WB RAW SH-KLHL40 CAPN1 CAPN2/CAPN1/1-ACTIN-MARKER.png]

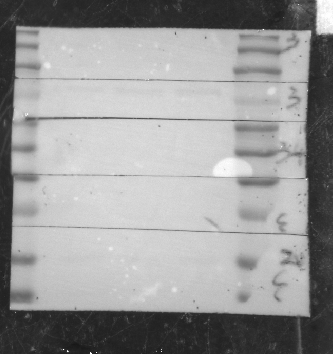

Supplement: Supplemental Information 37 [file peerj-14-21375-s037.zip › Figure 5C WB RAW SH-KLHL40 CAPN1 CAPN2/CAPN1/1ALL.png]

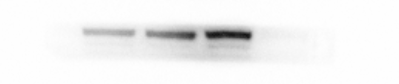

Supplement: Supplemental Information 37 [file peerj-14-21375-s037.zip › Figure 5C WB RAW SH-KLHL40 CAPN1 CAPN2/CAPN1/1-CAPN1.png]

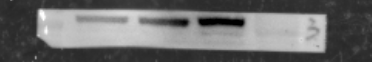

Supplement: Supplemental Information 37 [file peerj-14-21375-s037.zip › Figure 5C WB RAW SH-KLHL40 CAPN1 CAPN2/CAPN1/1-CAPN1-MARKER.png]

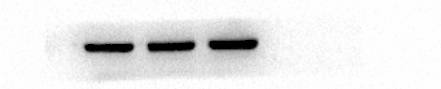

Supplement: Supplemental Information 37 [file peerj-14-21375-s037.zip › Figure 5C WB RAW SH-KLHL40 CAPN1 CAPN2/CAPN1/2-ACTIN.png]

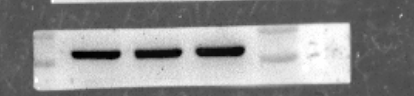

Supplement: Supplemental Information 37 [file peerj-14-21375-s037.zip › Figure 5C WB RAW SH-KLHL40 CAPN1 CAPN2/CAPN1/2-ACTIN-MARKER.png]

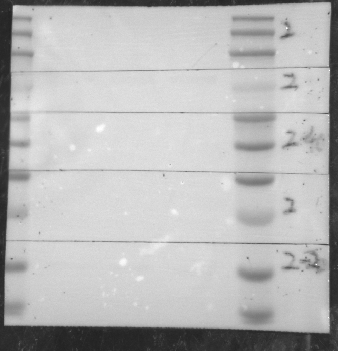

Supplement: Supplemental Information 37 [file peerj-14-21375-s037.zip › Figure 5C WB RAW SH-KLHL40 CAPN1 CAPN2/CAPN1/2ALL.png]

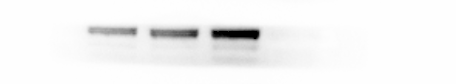

Supplement: Supplemental Information 37 [file peerj-14-21375-s037.zip › Figure 5C WB RAW SH-KLHL40 CAPN1 CAPN2/CAPN1/2-CAPN1.png]

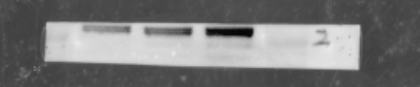

Supplement: Supplemental Information 37 [file peerj-14-21375-s037.zip › Figure 5C WB RAW SH-KLHL40 CAPN1 CAPN2/CAPN1/2-CAPN1-MARER.png]

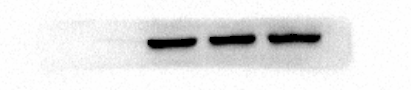

Supplement: Supplemental Information 37 [file peerj-14-21375-s037.zip › Figure 5C WB RAW SH-KLHL40 CAPN1 CAPN2/CAPN1/3-ACTIN.png]

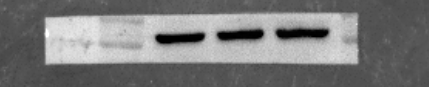

Supplement: Supplemental Information 37 [file peerj-14-21375-s037.zip › Figure 5C WB RAW SH-KLHL40 CAPN1 CAPN2/CAPN1/3-ACTIN-MARKER.png]

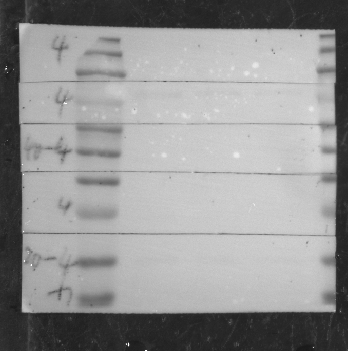

Supplement: Supplemental Information 37 [file peerj-14-21375-s037.zip › Figure 5C WB RAW SH-KLHL40 CAPN1 CAPN2/CAPN1/3ALL.png]

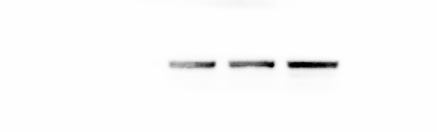

Supplement: Supplemental Information 37 [file peerj-14-21375-s037.zip › Figure 5C WB RAW SH-KLHL40 CAPN1 CAPN2/CAPN1/3-CAPN1.png]

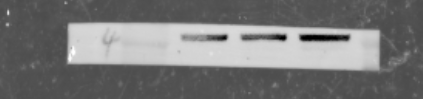

Supplement: Supplemental Information 37 [file peerj-14-21375-s037.zip › Figure 5C WB RAW SH-KLHL40 CAPN1 CAPN2/CAPN1/3-CAPN1-MARKER.png]

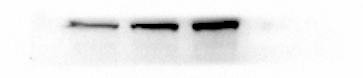

Supplement: Supplemental Information 37 [file peerj-14-21375-s037.zip › Figure 5C WB RAW SH-KLHL40 CAPN1 CAPN2/CAPN2/1CAPN2.png]

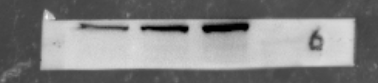

Supplement: Supplemental Information 37 [file peerj-14-21375-s037.zip › Figure 5C WB RAW SH-KLHL40 CAPN1 CAPN2/CAPN2/1CAPN2-MARKER.png]

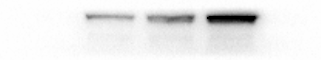

Supplement: Supplemental Information 37 [file peerj-14-21375-s037.zip › Figure 5C WB RAW SH-KLHL40 CAPN1 CAPN2/CAPN2/2CAPN2.png]

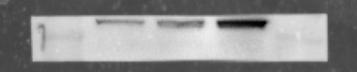

Supplement: Supplemental Information 37 [file peerj-14-21375-s037.zip › Figure 5C WB RAW SH-KLHL40 CAPN1 CAPN2/CAPN2/2CAPN2-MARKER.png]

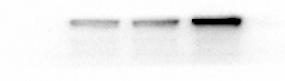

Supplement: Supplemental Information 37 [file peerj-14-21375-s037.zip › Figure 5C WB RAW SH-KLHL40 CAPN1 CAPN2/CAPN2/3CAPN2.png]

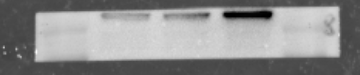

Supplement: Supplemental Information 37 [file peerj-14-21375-s037.zip › Figure 5C WB RAW SH-KLHL40 CAPN1 CAPN2/CAPN2/3CAPN2-MARKER.png]

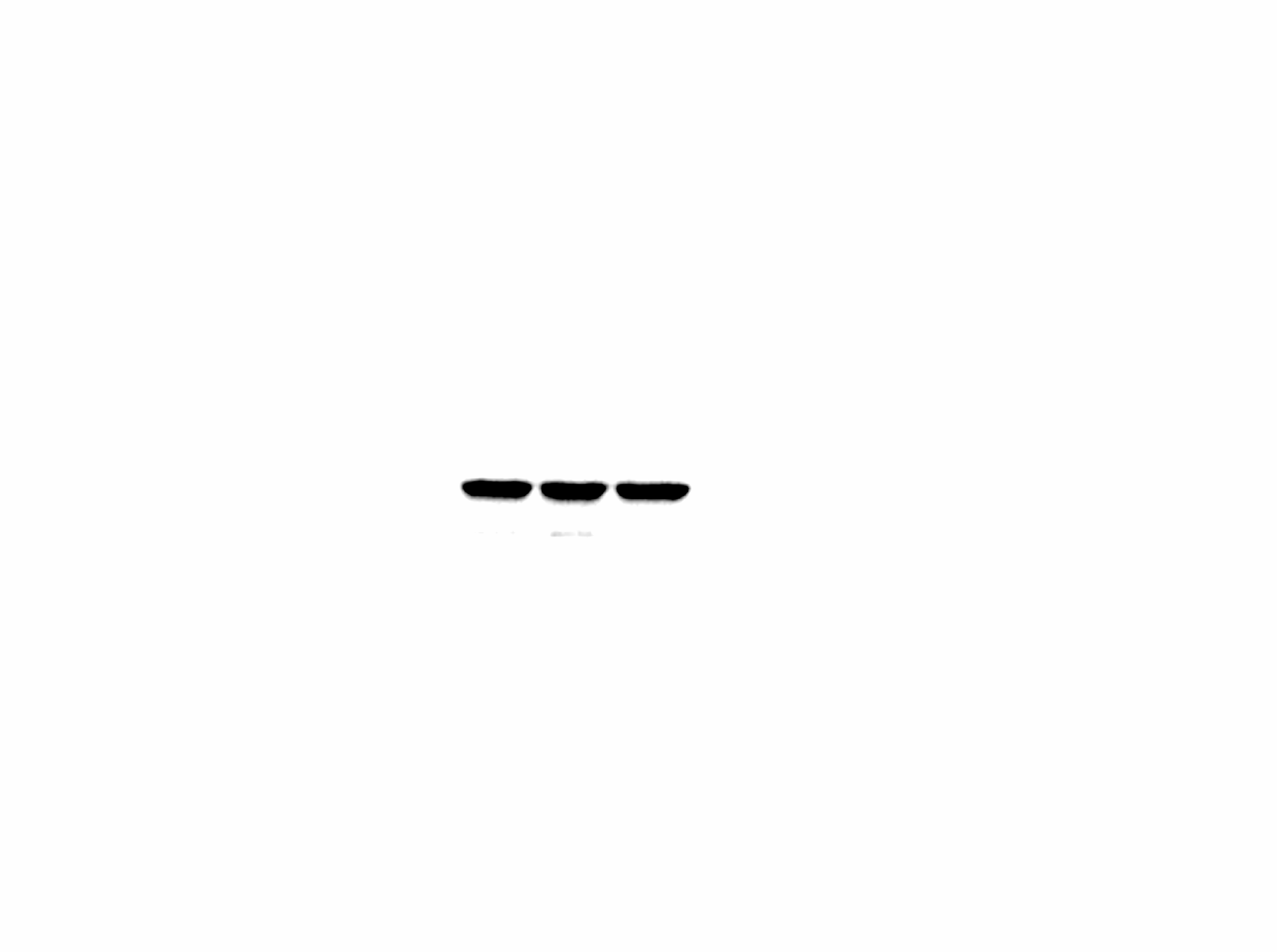

Supplement: Supplemental Information 38 — CAPN1 is involved in [brief function, e.g., cytoskeletal remodeling/signaling pathways]. [file peerj-14-21375-s038.zip › Figure 5D WB RAW OE-KLHL40 CAPN1/1ACTIN.tif]

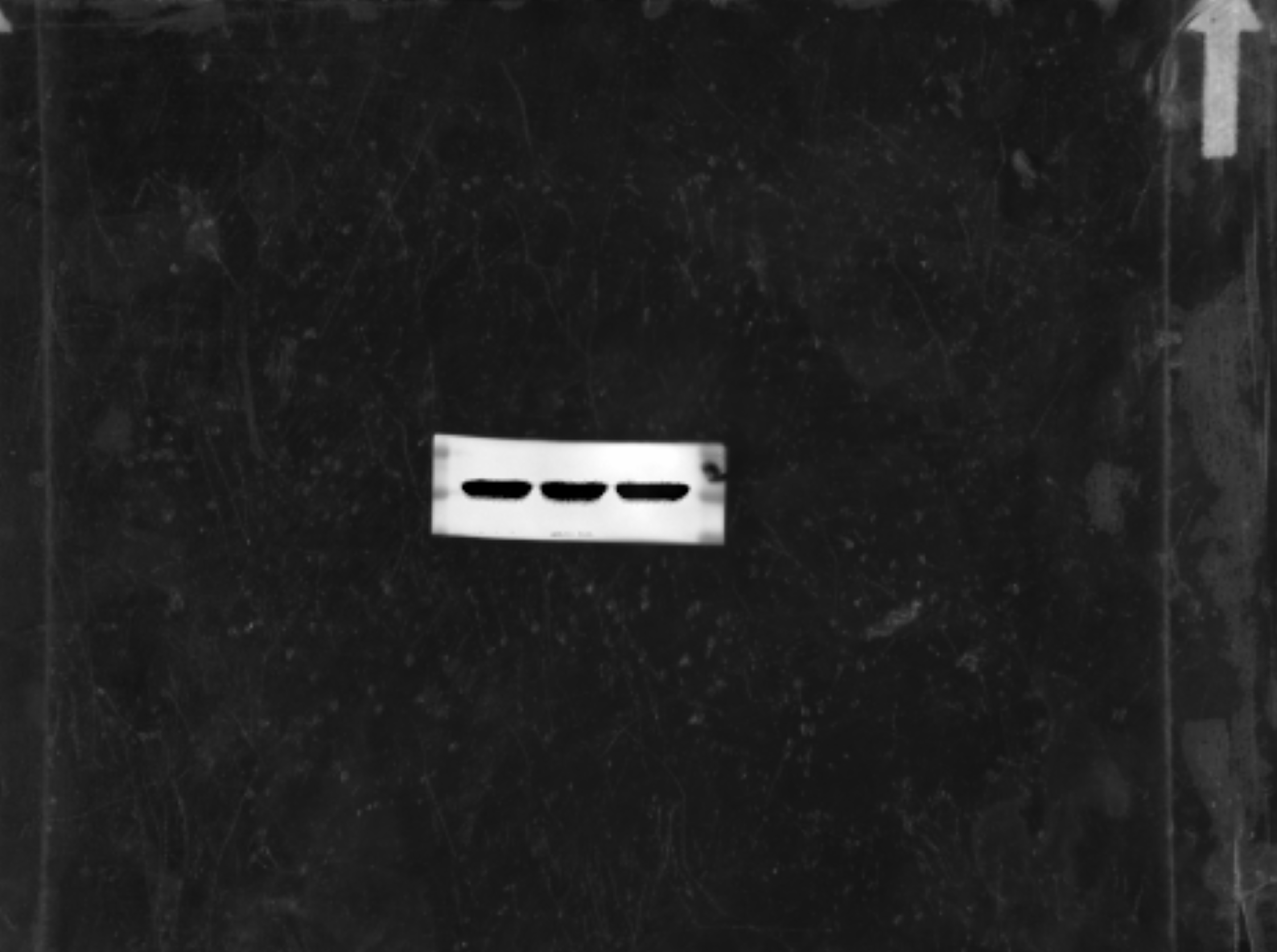

Supplement: Supplemental Information 38 — CAPN1 is involved in [brief function, e.g., cytoskeletal remodeling/signaling pathways]. [file peerj-14-21375-s038.zip › Figure 5D WB RAW OE-KLHL40 CAPN1/1ACTIN+MARKER.tif]

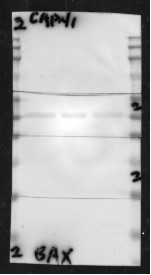

Supplement: Supplemental Information 38 — CAPN1 is involved in [brief function, e.g., cytoskeletal remodeling/signaling pathways]. [file peerj-14-21375-s038.zip › Figure 5D WB RAW OE-KLHL40 CAPN1/1ALL.jpg]

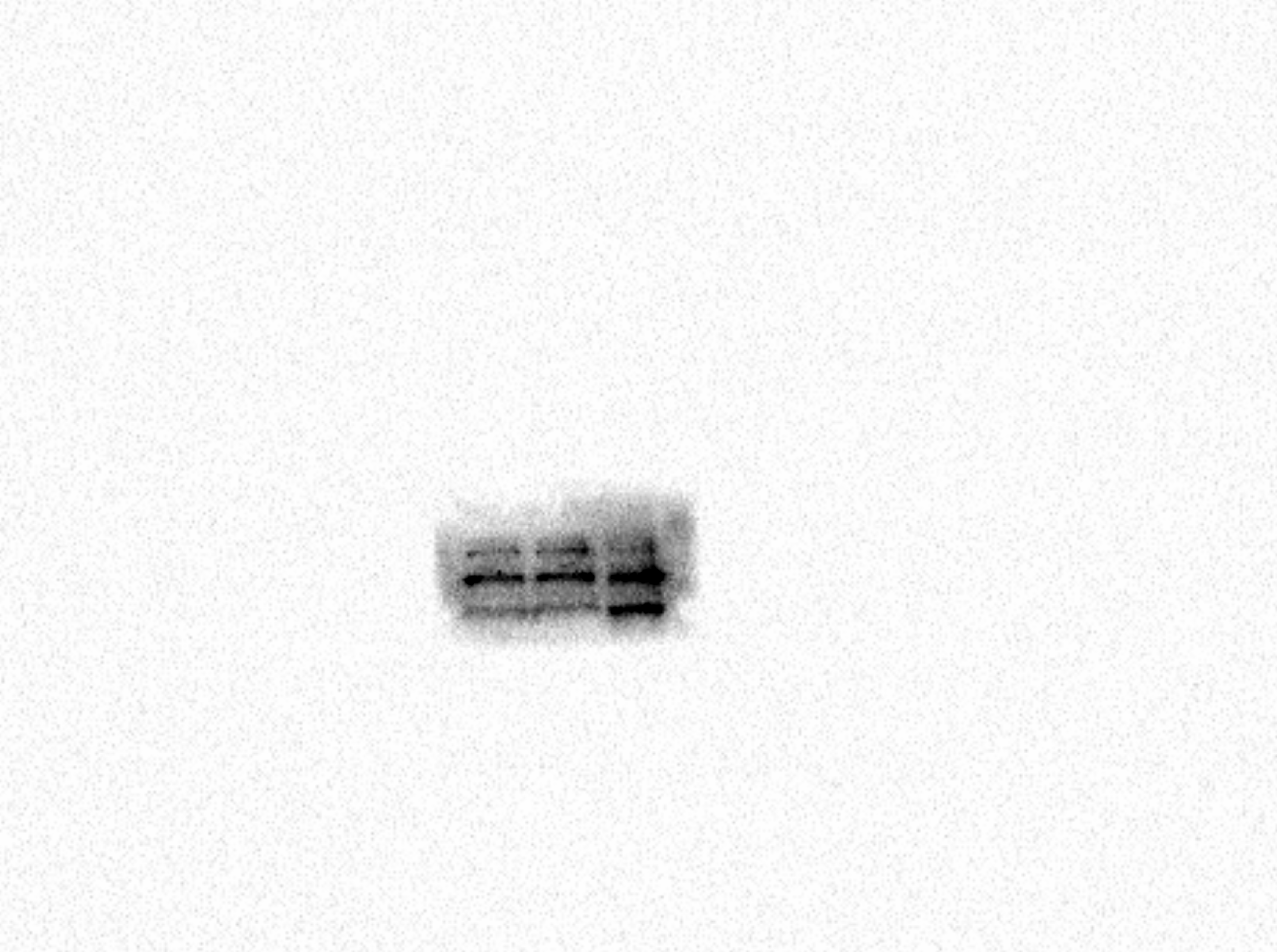

Supplement: Supplemental Information 38 — CAPN1 is involved in [brief function, e.g., cytoskeletal remodeling/signaling pathways]. [file peerj-14-21375-s038.zip › Figure 5D WB RAW OE-KLHL40 CAPN1/1CAPN1.tif]

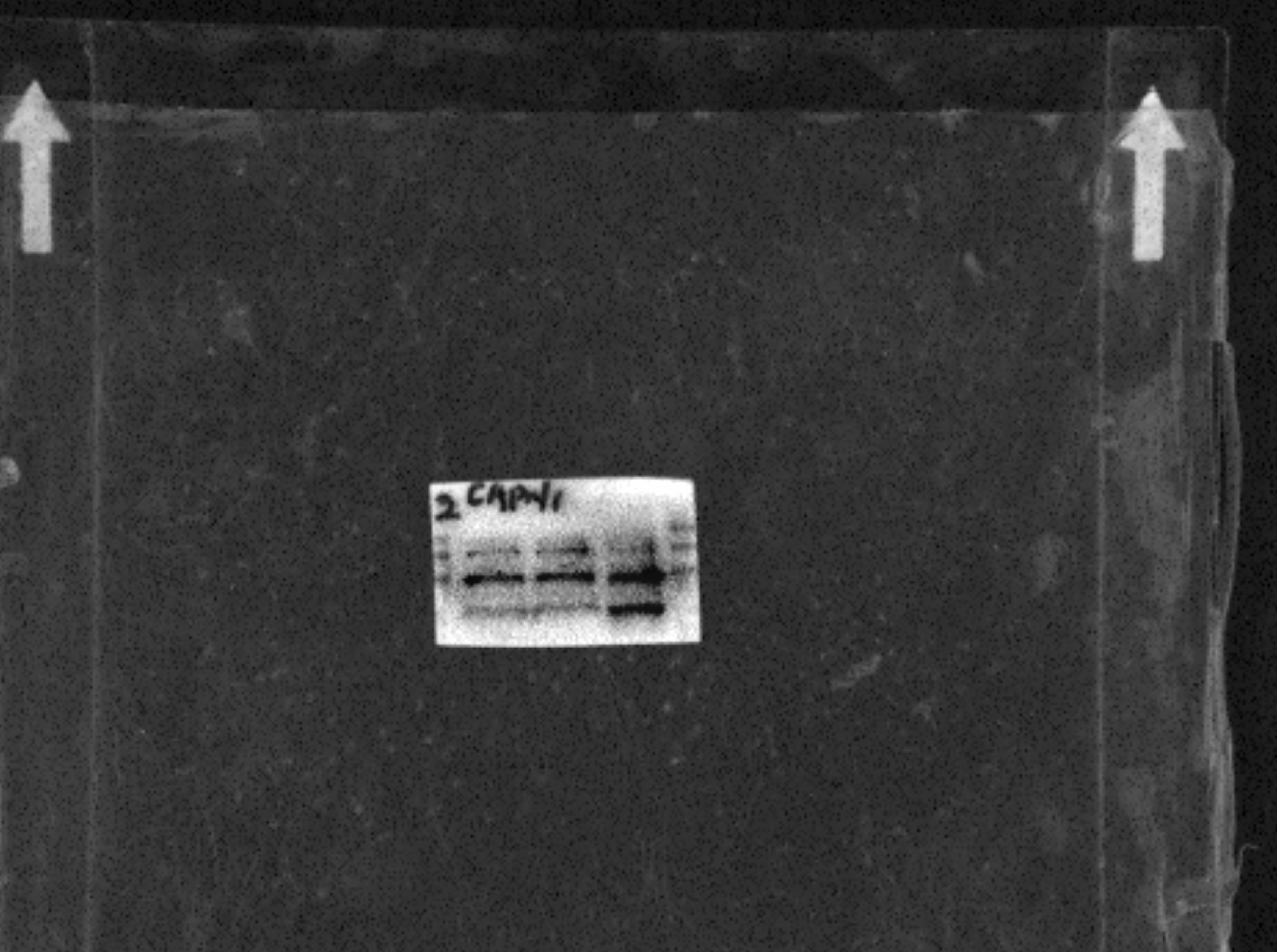

Supplement: Supplemental Information 38 — CAPN1 is involved in [brief function, e.g., cytoskeletal remodeling/signaling pathways]. [file peerj-14-21375-s038.zip › Figure 5D WB RAW OE-KLHL40 CAPN1/1CAPN1+MARKER.tif]

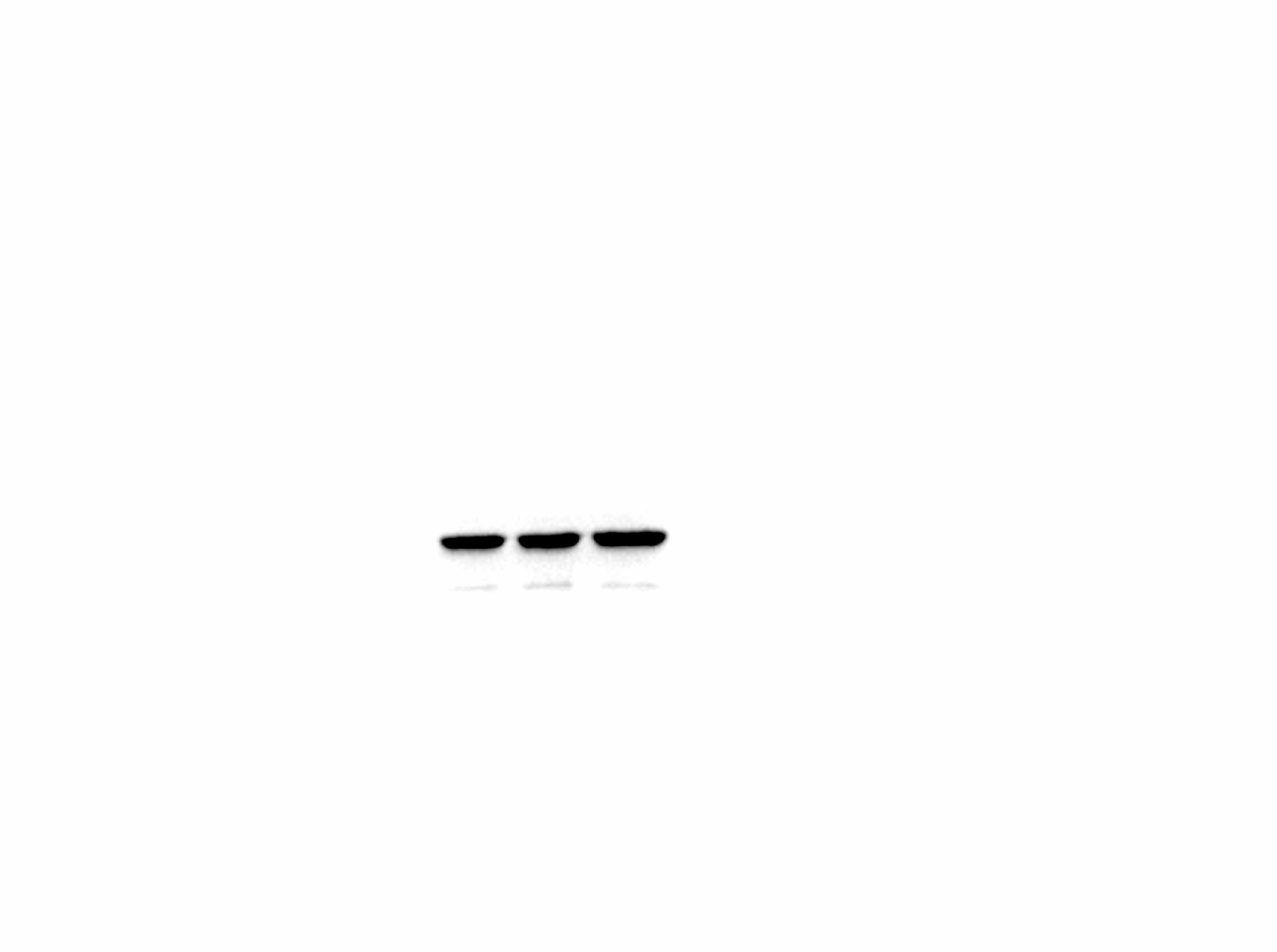

Supplement: Supplemental Information 38 — CAPN1 is involved in [brief function, e.g., cytoskeletal remodeling/signaling pathways]. [file peerj-14-21375-s038.zip › Figure 5D WB RAW OE-KLHL40 CAPN1/2ACTIN.tif]

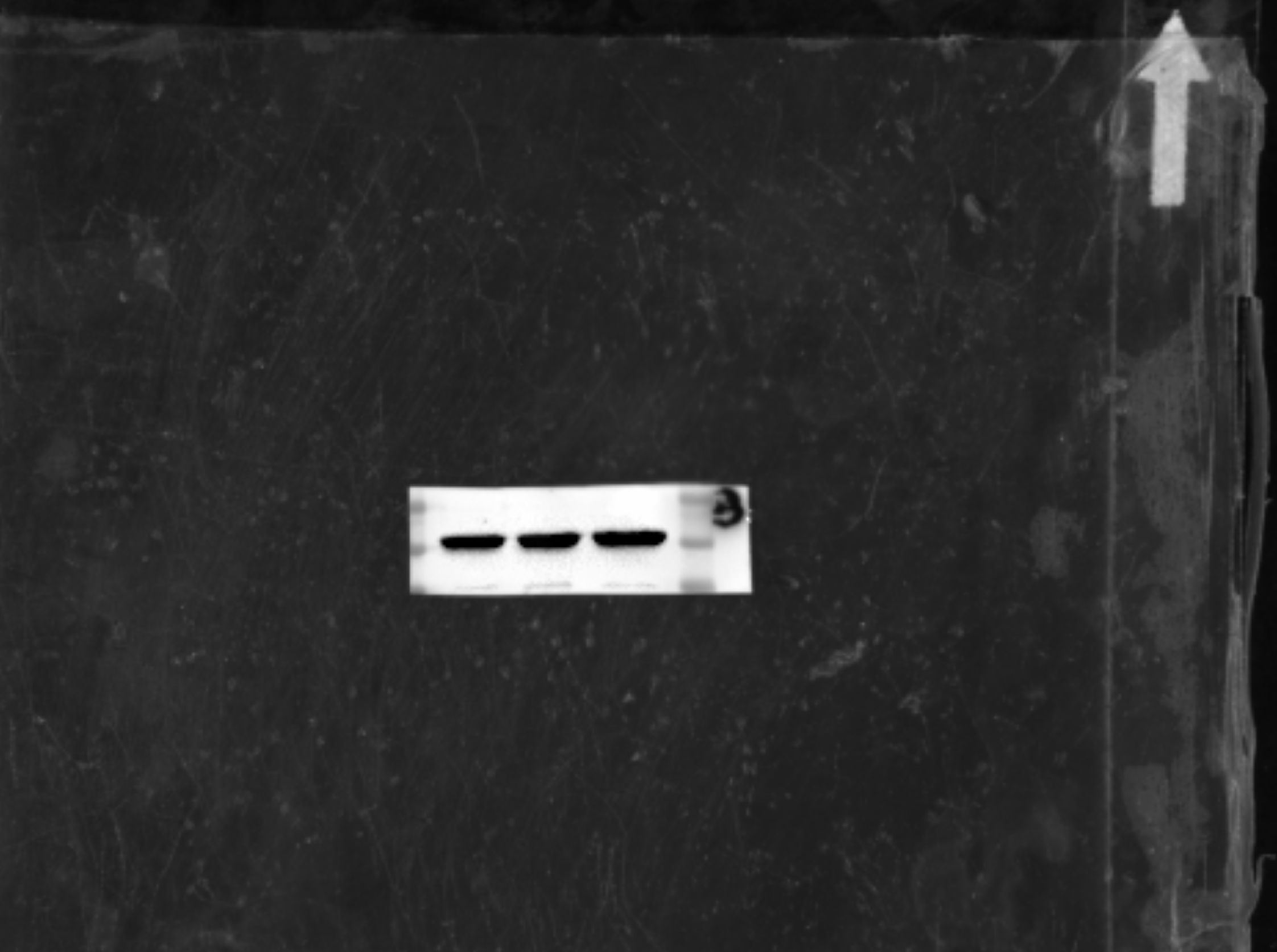

Supplement: Supplemental Information 38 — CAPN1 is involved in [brief function, e.g., cytoskeletal remodeling/signaling pathways]. [file peerj-14-21375-s038.zip › Figure 5D WB RAW OE-KLHL40 CAPN1/2ACTIN+MARKER.tif]

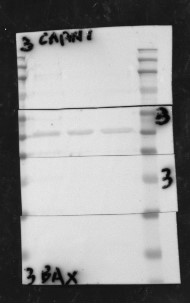

Supplement: Supplemental Information 38 — CAPN1 is involved in [brief function, e.g., cytoskeletal remodeling/signaling pathways]. [file peerj-14-21375-s038.zip › Figure 5D WB RAW OE-KLHL40 CAPN1/2ALL.jpg]

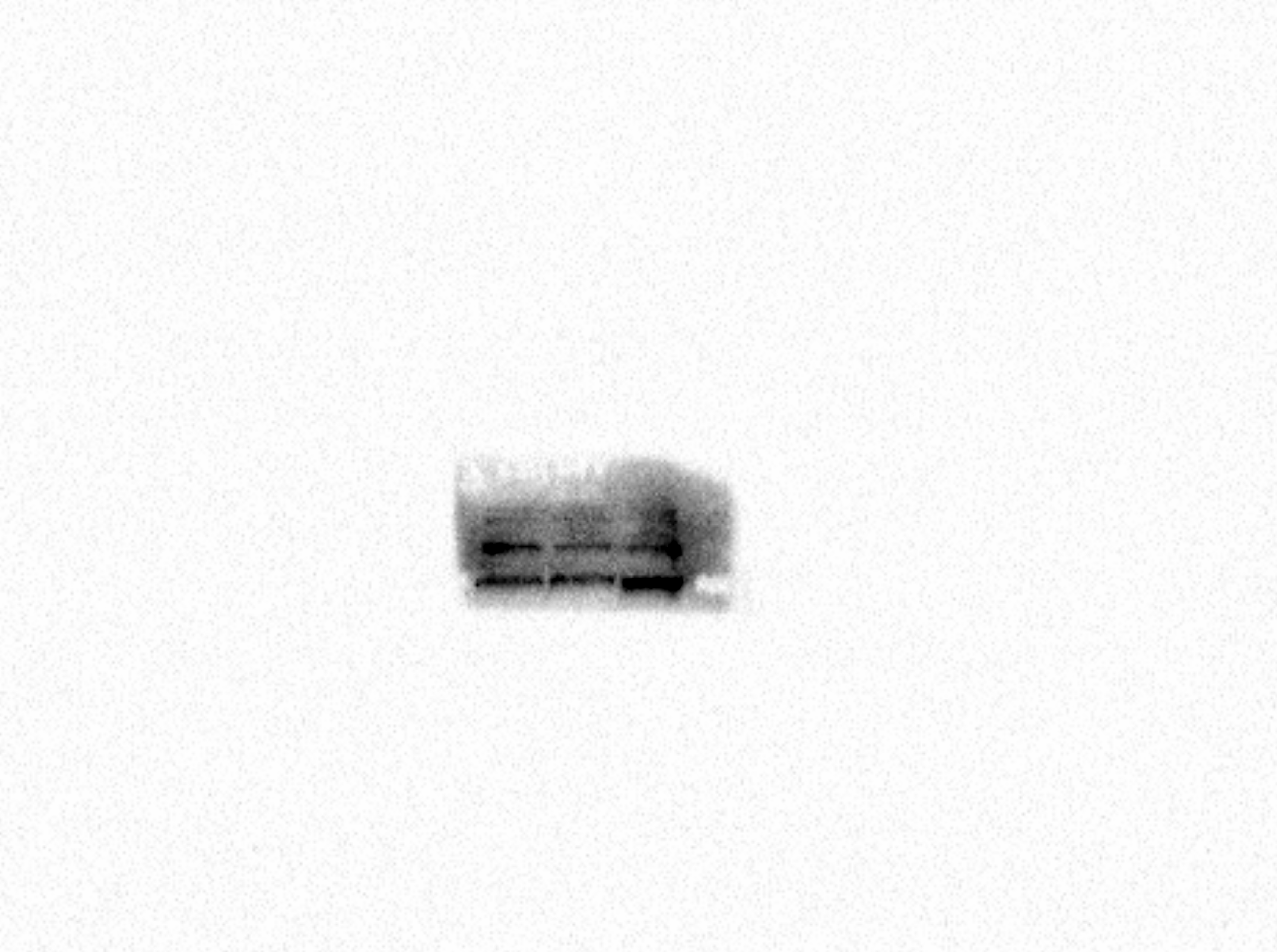

Supplement: Supplemental Information 38 — CAPN1 is involved in [brief function, e.g., cytoskeletal remodeling/signaling pathways]. [file peerj-14-21375-s038.zip › Figure 5D WB RAW OE-KLHL40 CAPN1/2CAPN1.tif]

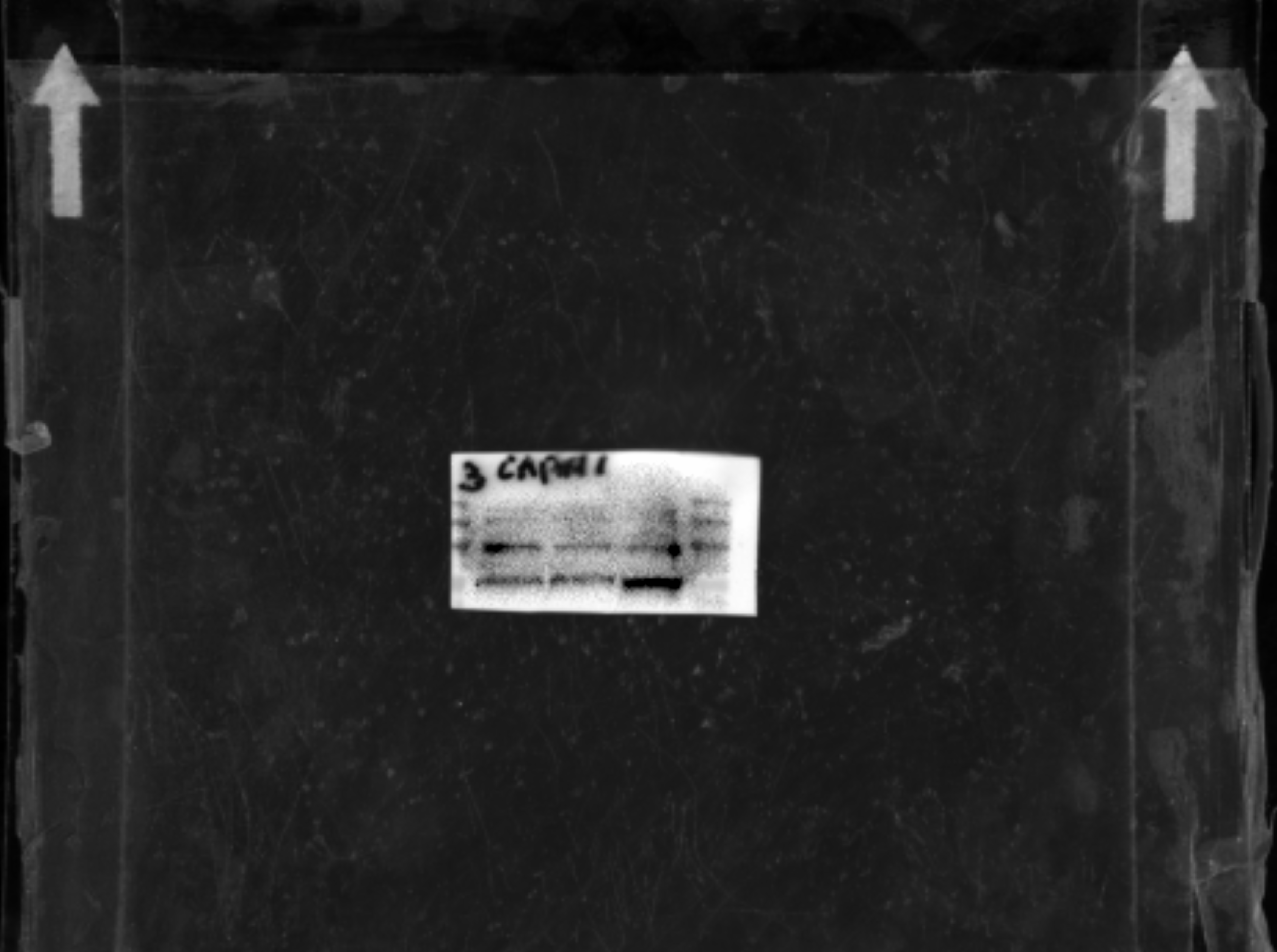

Supplement: Supplemental Information 38 — CAPN1 is involved in [brief function, e.g., cytoskeletal remodeling/signaling pathways]. [file peerj-14-21375-s038.zip › Figure 5D WB RAW OE-KLHL40 CAPN1/2CAPN1+MARKER.tif]

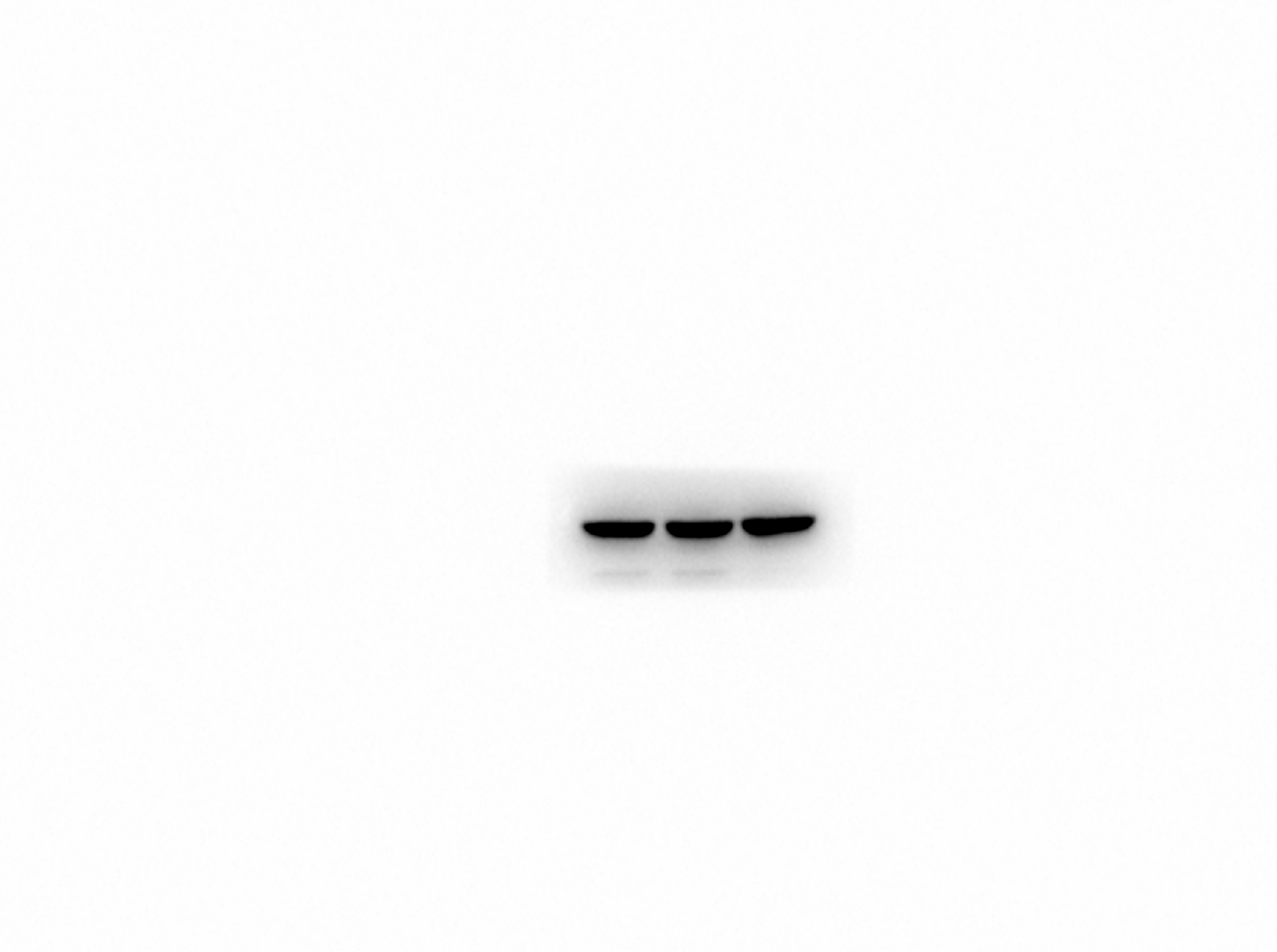

Supplement: Supplemental Information 38 — CAPN1 is involved in [brief function, e.g., cytoskeletal remodeling/signaling pathways]. [file peerj-14-21375-s038.zip › Figure 5D WB RAW OE-KLHL40 CAPN1/3ACTIN.tif]

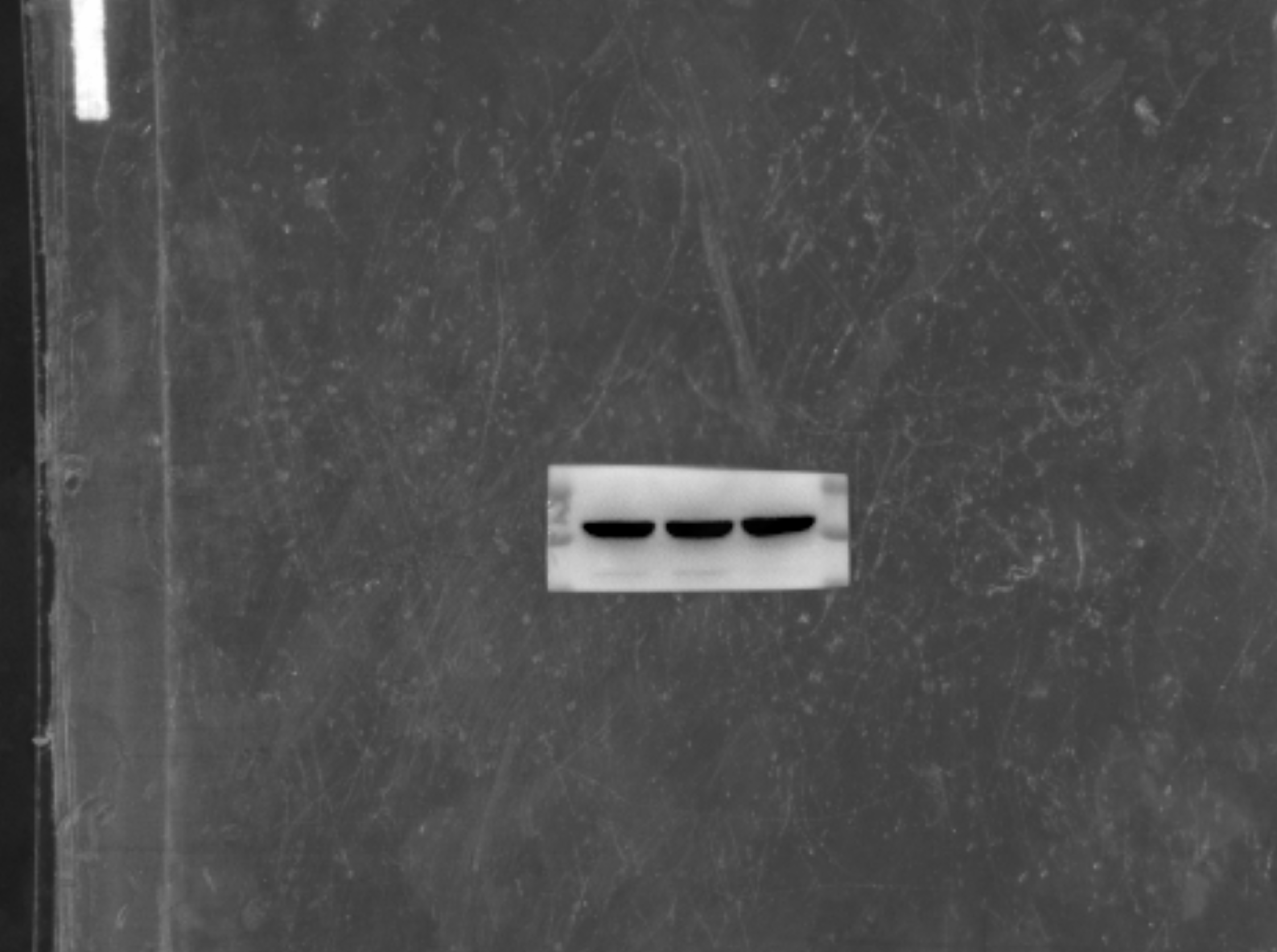

Supplement: Supplemental Information 38 — CAPN1 is involved in [brief function, e.g., cytoskeletal remodeling/signaling pathways]. [file peerj-14-21375-s038.zip › Figure 5D WB RAW OE-KLHL40 CAPN1/3ACTIN+MARKER.tif]

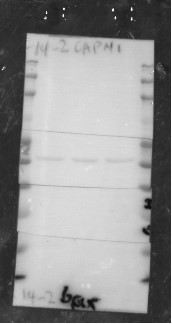

Supplement: Supplemental Information 38 — CAPN1 is involved in [brief function, e.g., cytoskeletal remodeling/signaling pathways]. [file peerj-14-21375-s038.zip › Figure 5D WB RAW OE-KLHL40 CAPN1/3ALL.jpg]

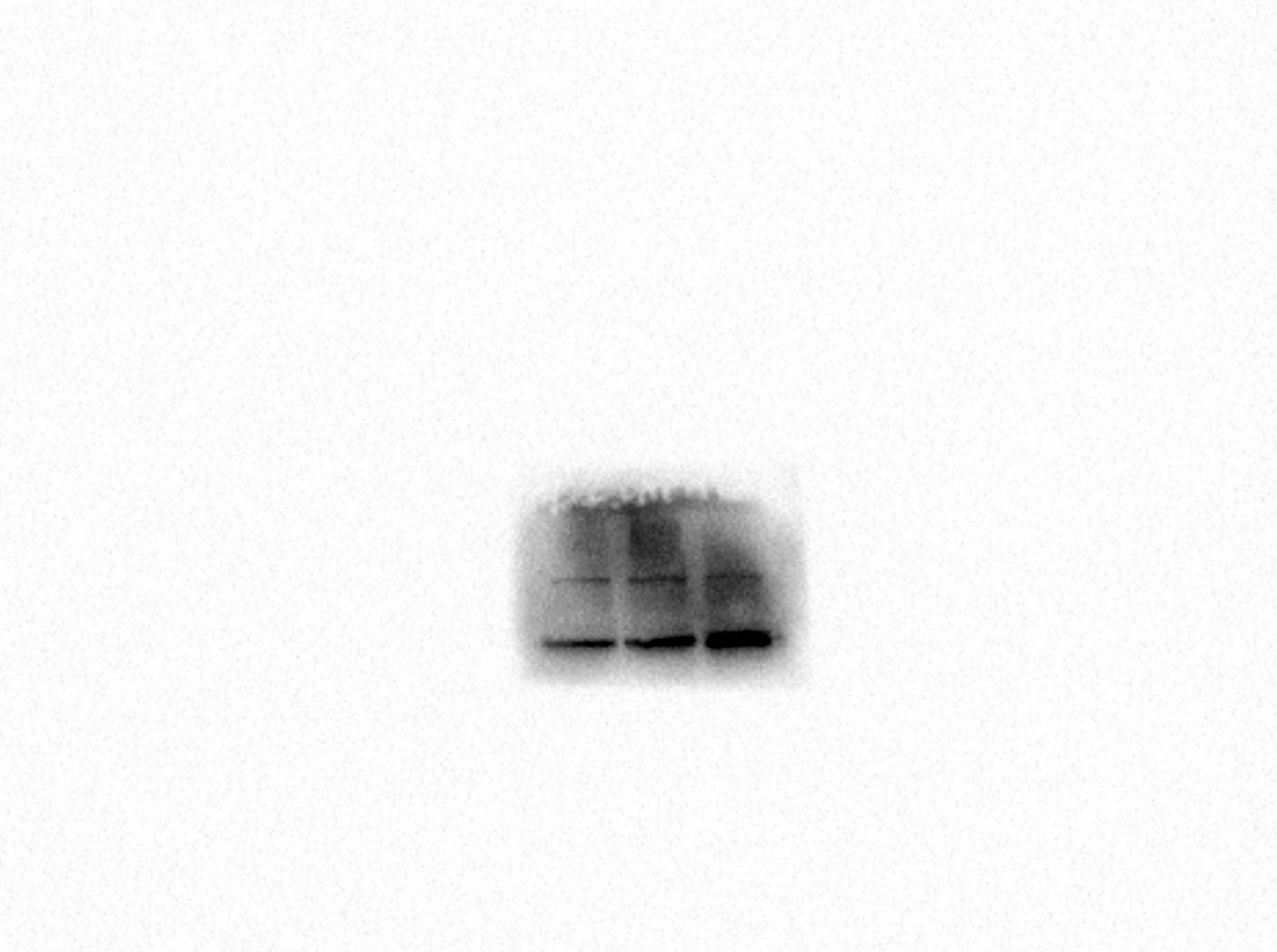

Supplement: Supplemental Information 38 — CAPN1 is involved in [brief function, e.g., cytoskeletal remodeling/signaling pathways]. [file peerj-14-21375-s038.zip › Figure 5D WB RAW OE-KLHL40 CAPN1/3CAPN1.tif]

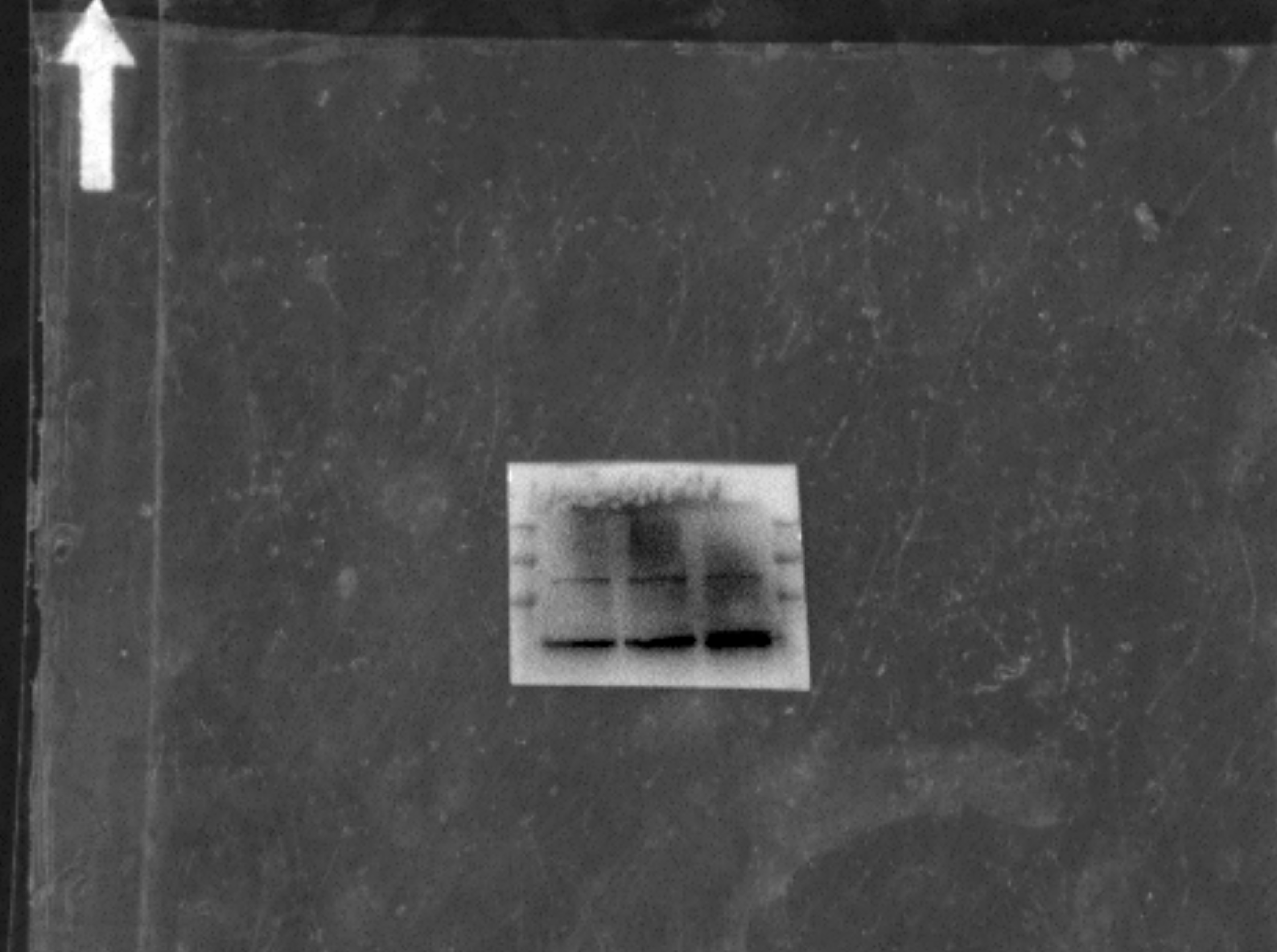

Supplement: Supplemental Information 38 — CAPN1 is involved in [brief function, e.g., cytoskeletal remodeling/signaling pathways]. [file peerj-14-21375-s038.zip › Figure 5D WB RAW OE-KLHL40 CAPN1/3CAPN1+MARKER.tif]

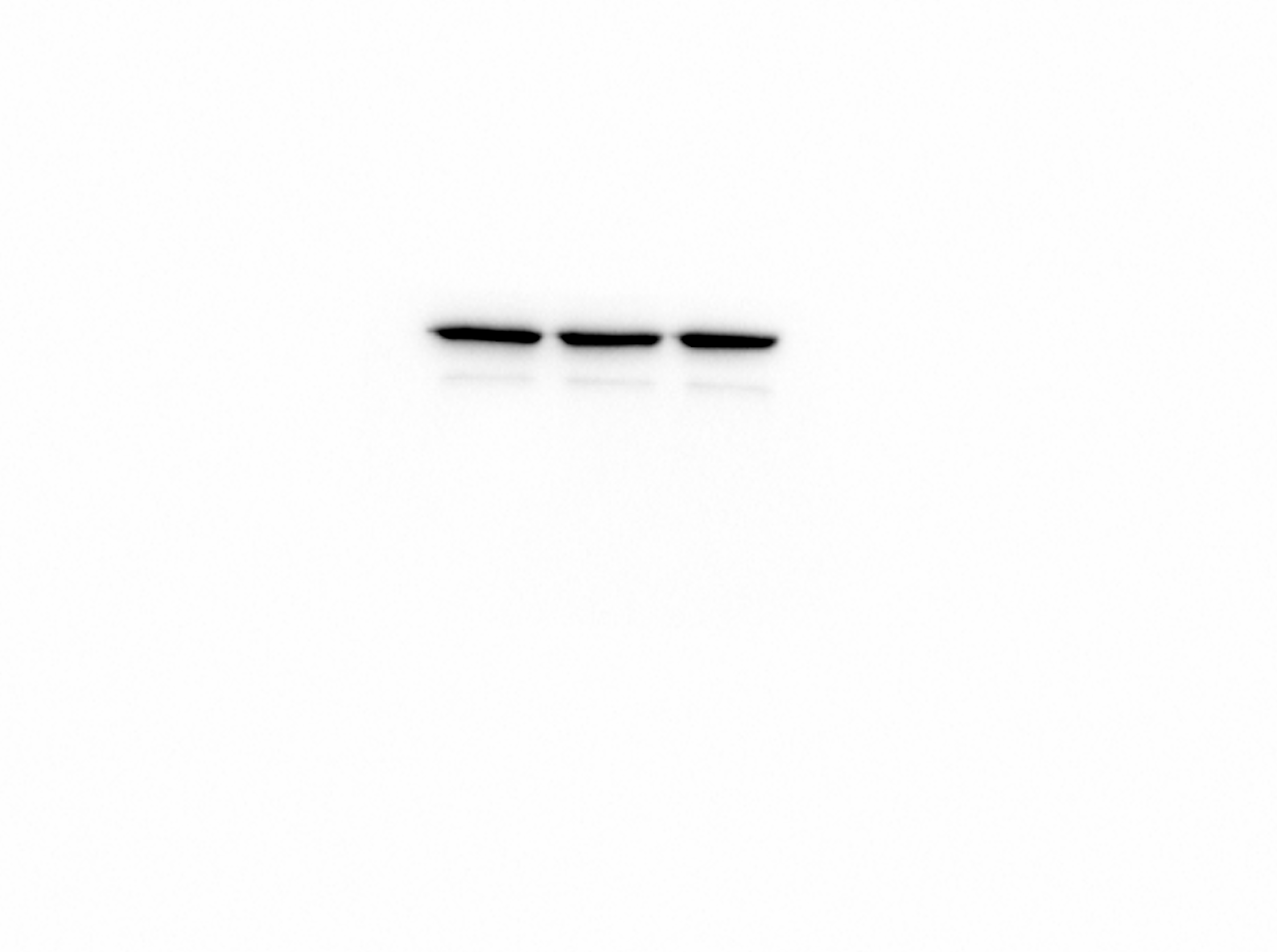

Supplement: Supplemental Information 39 — CAPN2 is involved in [brief function, e.g., cell migration/distinct proteolytic regulation compared to CAPN1] [file peerj-14-21375-s039.zip › Figure 5D WB RAW OE-KLHL40 CAPN2/1ACTB.tif]

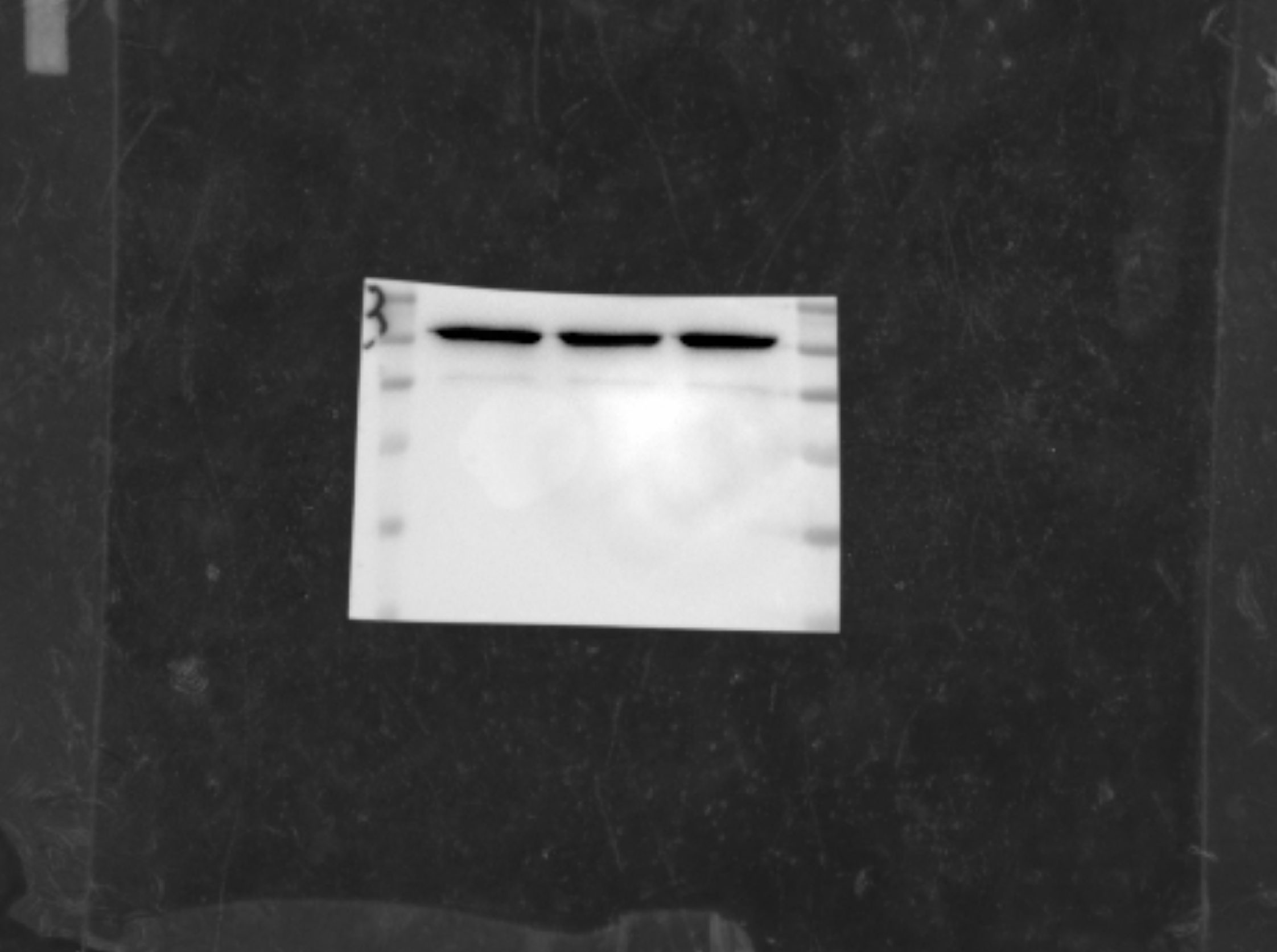

Supplement: Supplemental Information 39 — CAPN2 is involved in [brief function, e.g., cell migration/distinct proteolytic regulation compared to CAPN1] [file peerj-14-21375-s039.zip › Figure 5D WB RAW OE-KLHL40 CAPN2/1ACTIN+MARKER.tif]

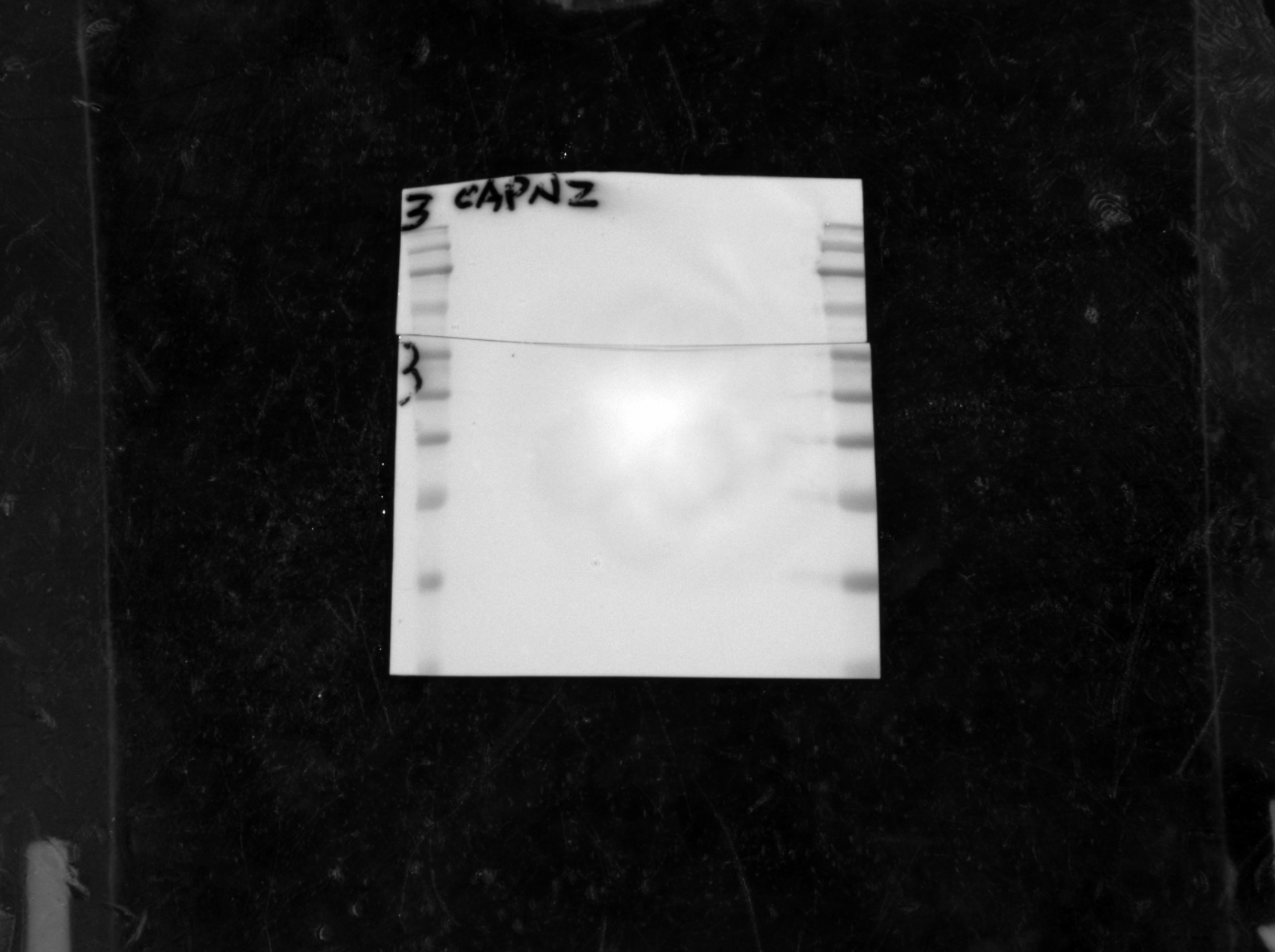

Supplement: Supplemental Information 39 — CAPN2 is involved in [brief function, e.g., cell migration/distinct proteolytic regulation compared to CAPN1] [file peerj-14-21375-s039.zip › Figure 5D WB RAW OE-KLHL40 CAPN2/1ALL.tif]

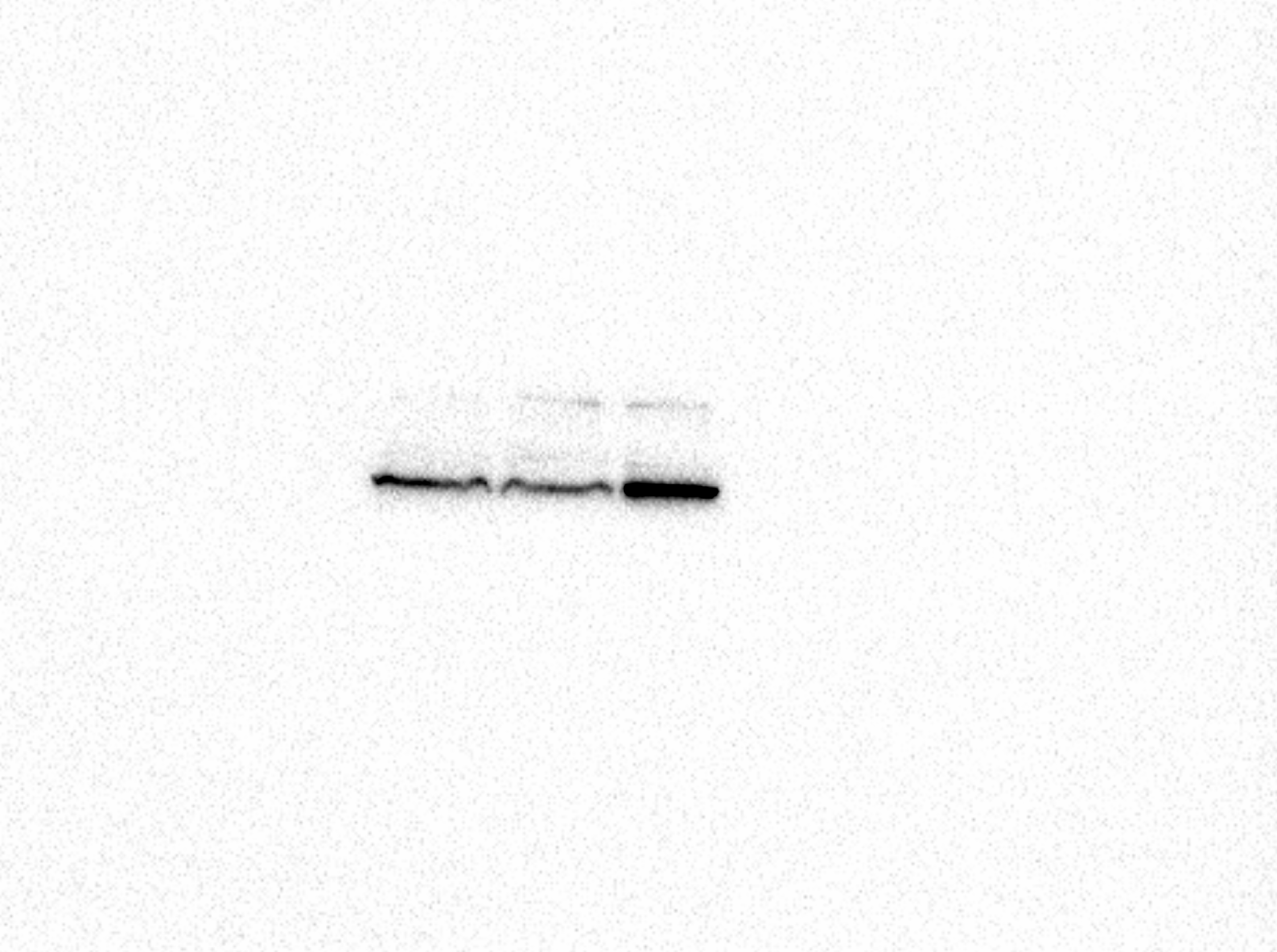

Supplement: Supplemental Information 39 — CAPN2 is involved in [brief function, e.g., cell migration/distinct proteolytic regulation compared to CAPN1] [file peerj-14-21375-s039.zip › Figure 5D WB RAW OE-KLHL40 CAPN2/1CAPN2.tif]

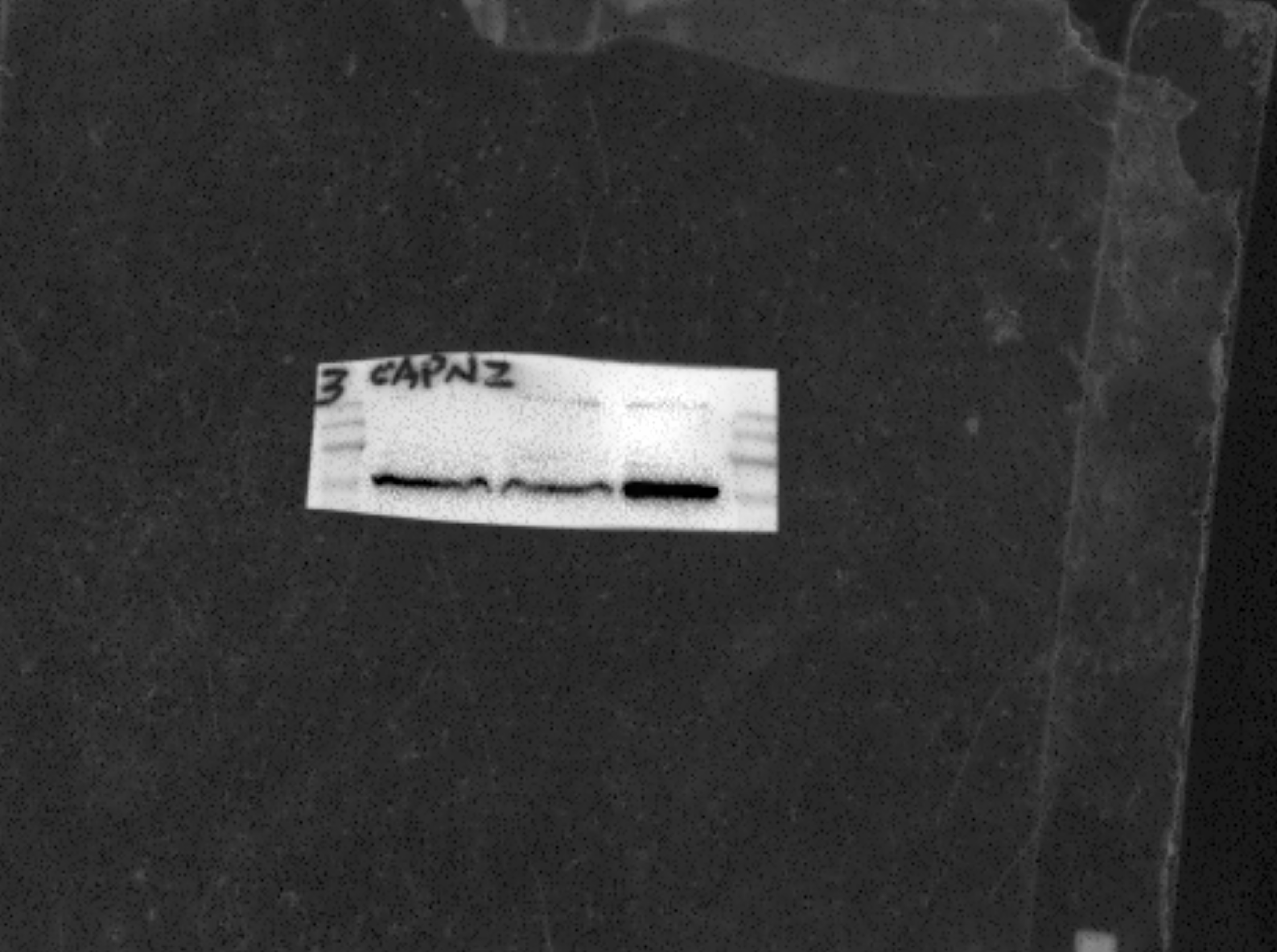

Supplement: Supplemental Information 39 — CAPN2 is involved in [brief function, e.g., cell migration/distinct proteolytic regulation compared to CAPN1] [file peerj-14-21375-s039.zip › Figure 5D WB RAW OE-KLHL40 CAPN2/1CAPN2+MARKER.tif]

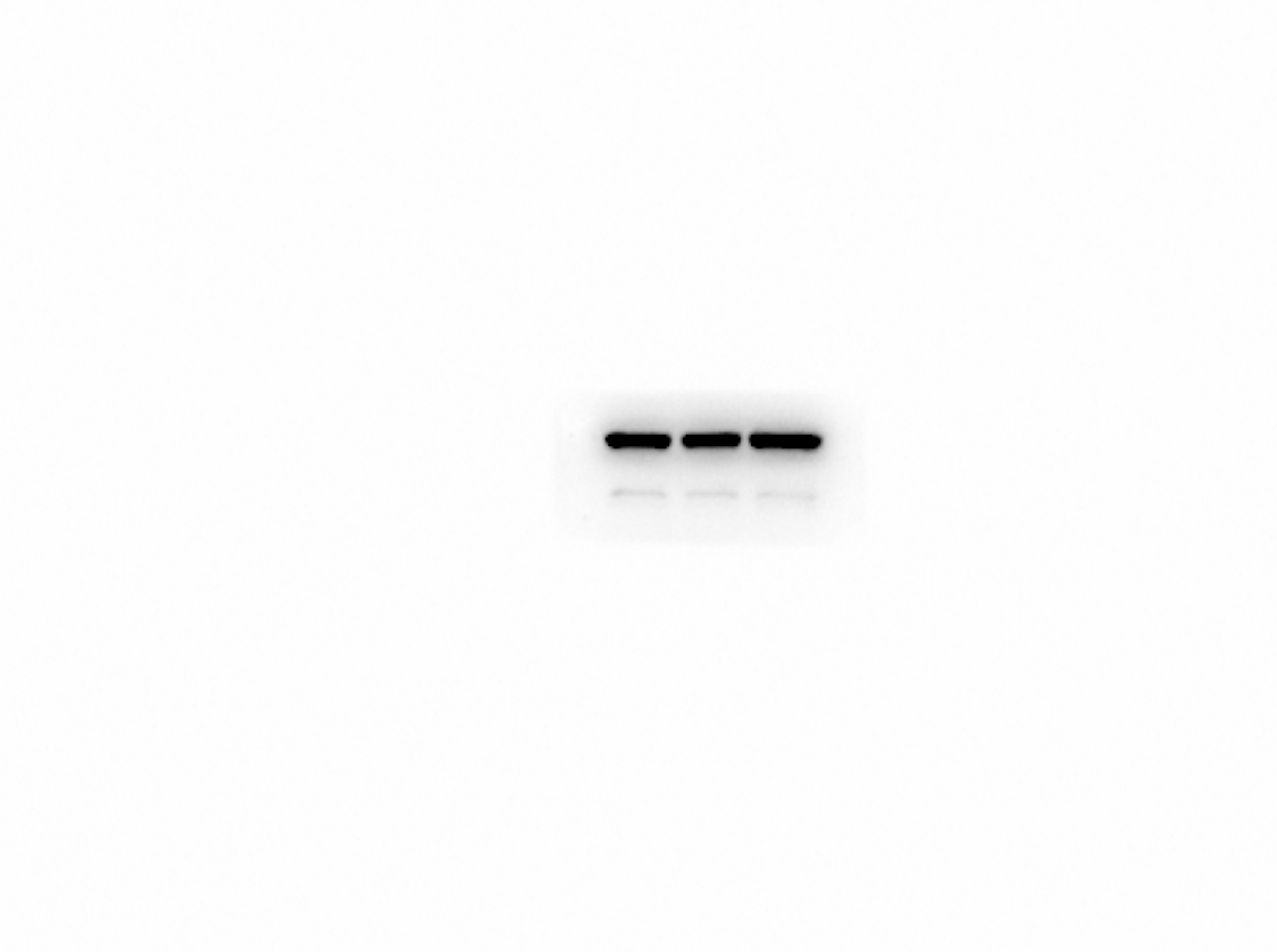

Supplement: Supplemental Information 39 — CAPN2 is involved in [brief function, e.g., cell migration/distinct proteolytic regulation compared to CAPN1] [file peerj-14-21375-s039.zip › Figure 5D WB RAW OE-KLHL40 CAPN2/2ACTIN.tif]

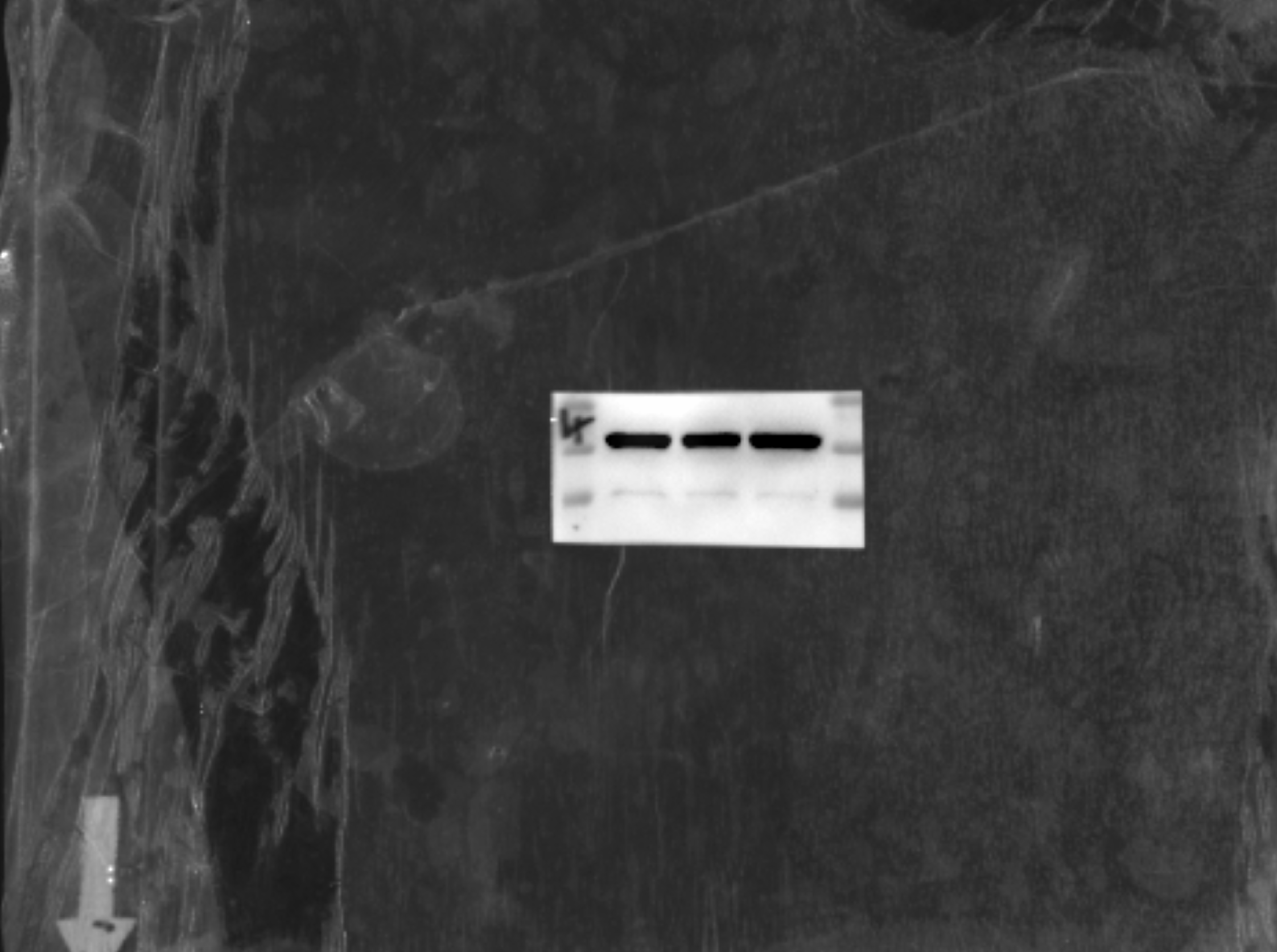

Supplement: Supplemental Information 39 — CAPN2 is involved in [brief function, e.g., cell migration/distinct proteolytic regulation compared to CAPN1] [file peerj-14-21375-s039.zip › Figure 5D WB RAW OE-KLHL40 CAPN2/2ACTIN+MARKER.tif]

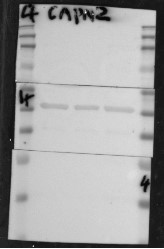

Supplement: Supplemental Information 39 — CAPN2 is involved in [brief function, e.g., cell migration/distinct proteolytic regulation compared to CAPN1] [file peerj-14-21375-s039.zip › Figure 5D WB RAW OE-KLHL40 CAPN2/2ALL.jpg]

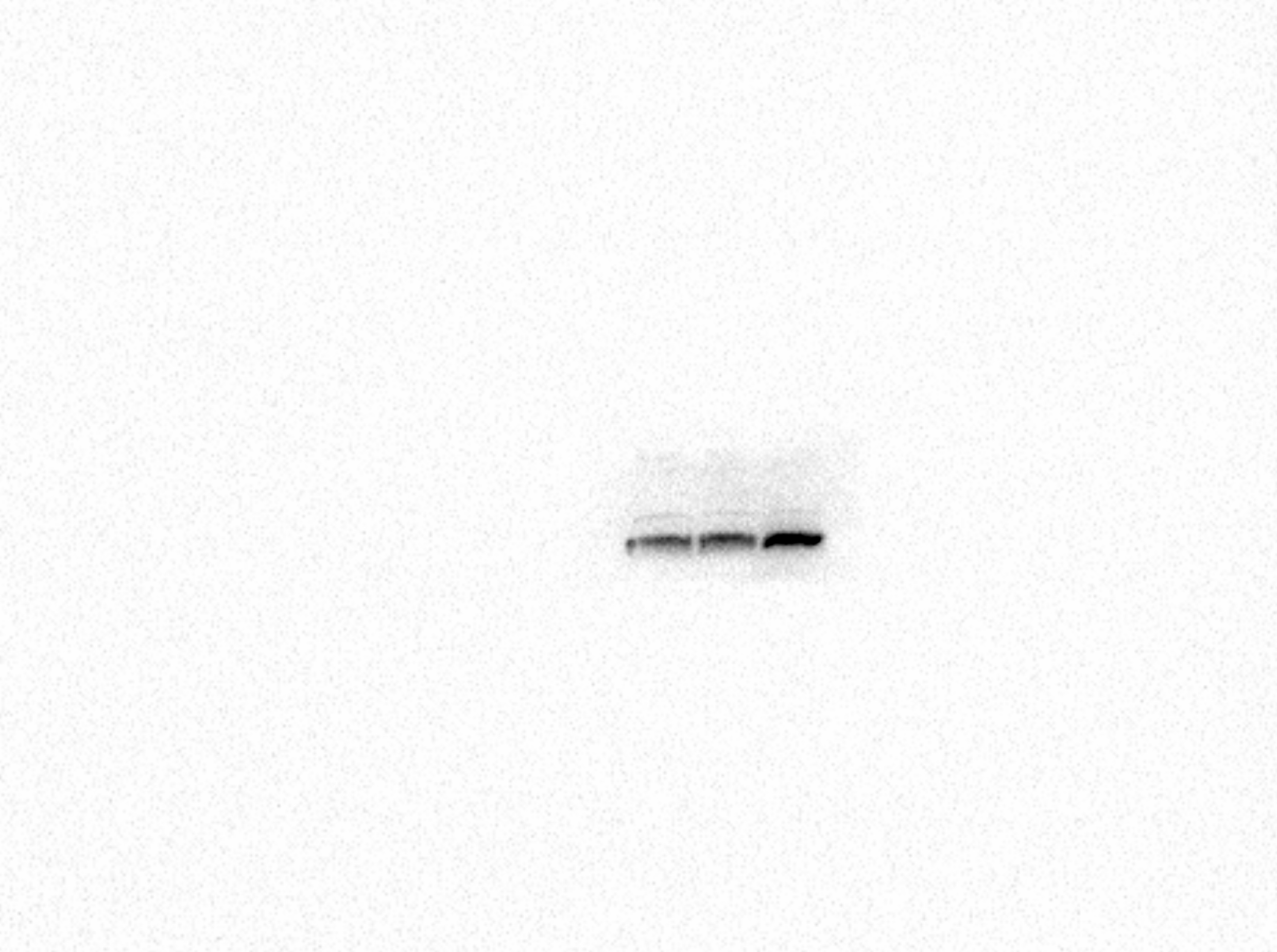

Supplement: Supplemental Information 39 — CAPN2 is involved in [brief function, e.g., cell migration/distinct proteolytic regulation compared to CAPN1] [file peerj-14-21375-s039.zip › Figure 5D WB RAW OE-KLHL40 CAPN2/2CAPN2.tif]

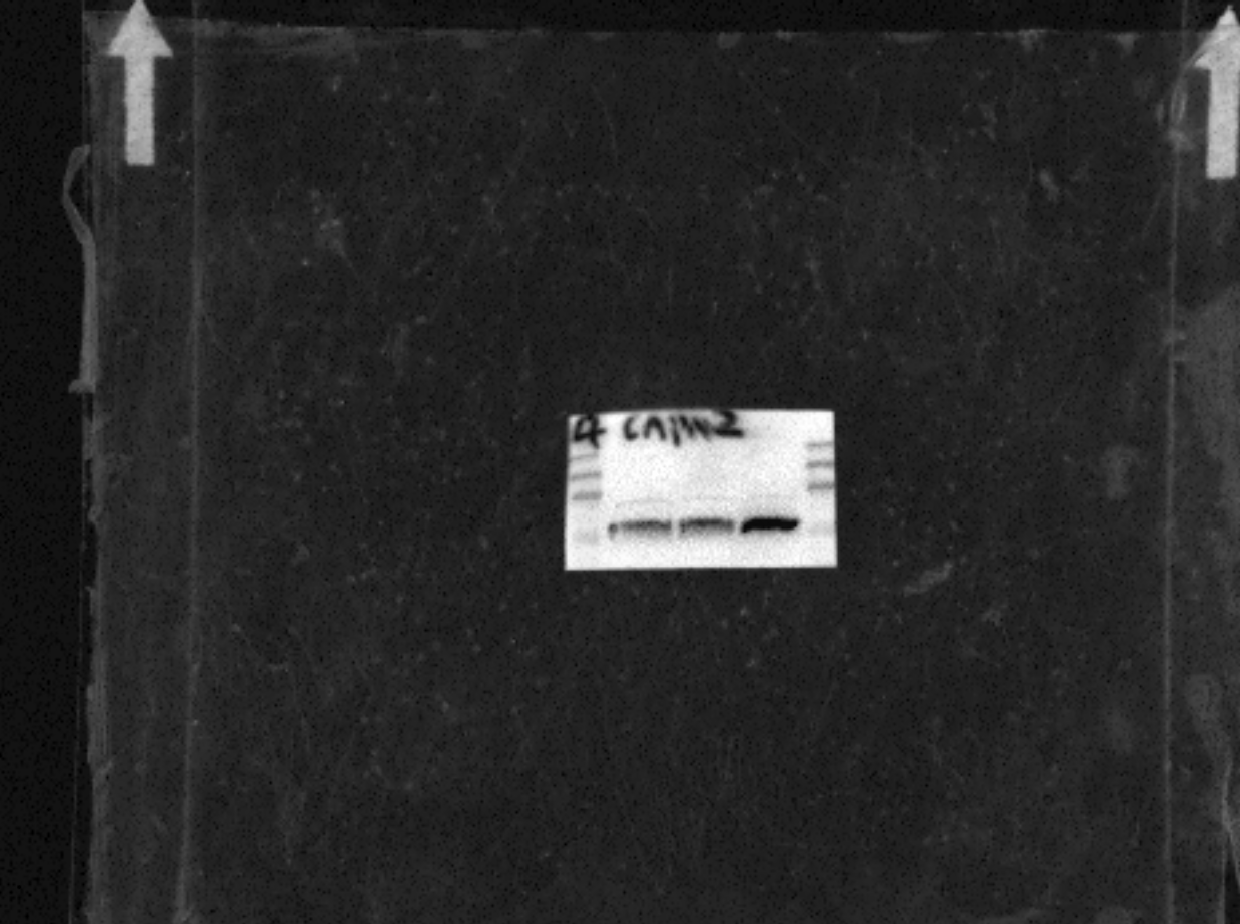

Supplement: Supplemental Information 39 — CAPN2 is involved in [brief function, e.g., cell migration/distinct proteolytic regulation compared to CAPN1] [file peerj-14-21375-s039.zip › Figure 5D WB RAW OE-KLHL40 CAPN2/2CAPN2+MARKER.tif]

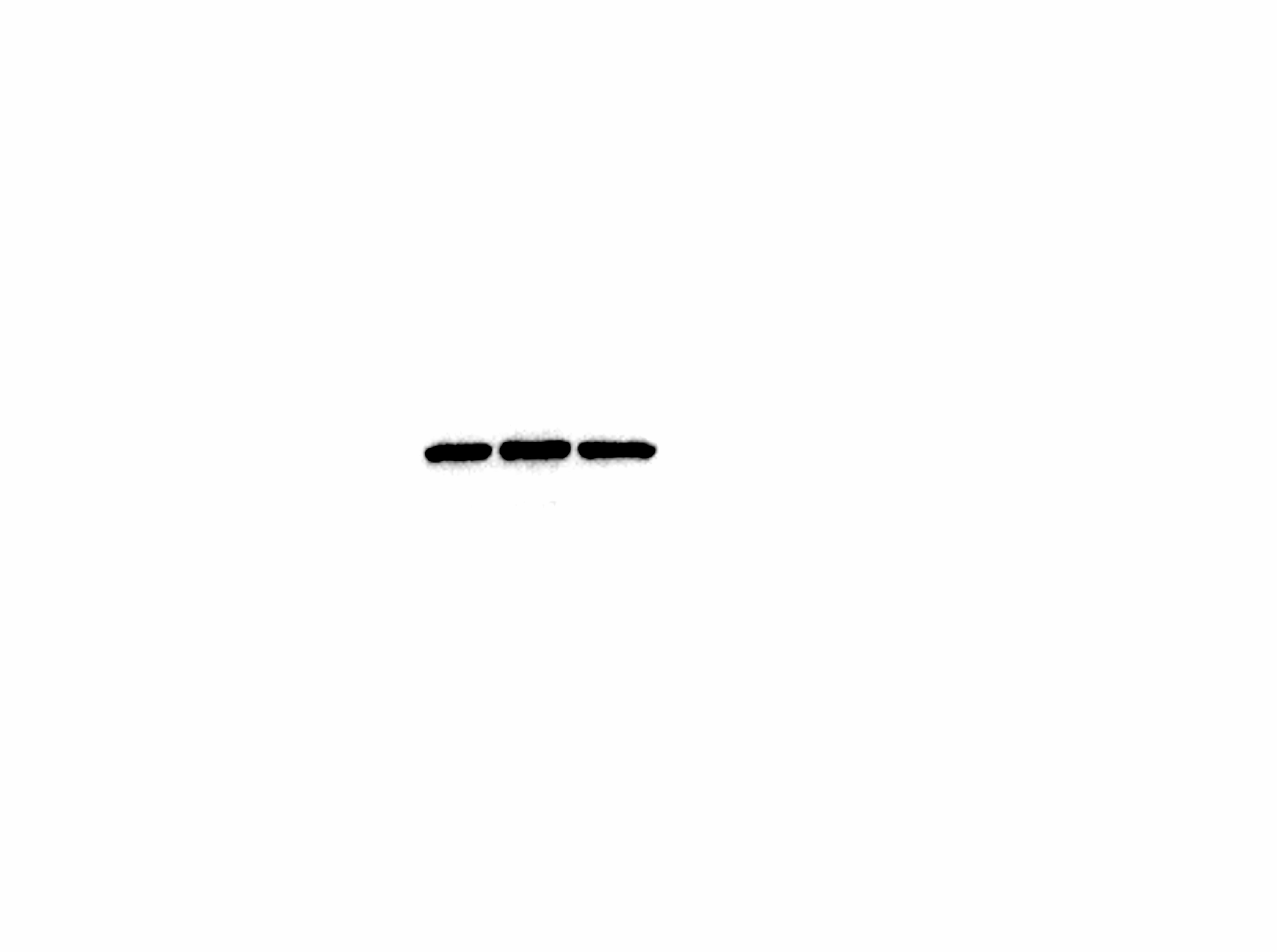

Supplement: Supplemental Information 39 — CAPN2 is involved in [brief function, e.g., cell migration/distinct proteolytic regulation compared to CAPN1] [file peerj-14-21375-s039.zip › Figure 5D WB RAW OE-KLHL40 CAPN2/3ACTIN.tif]

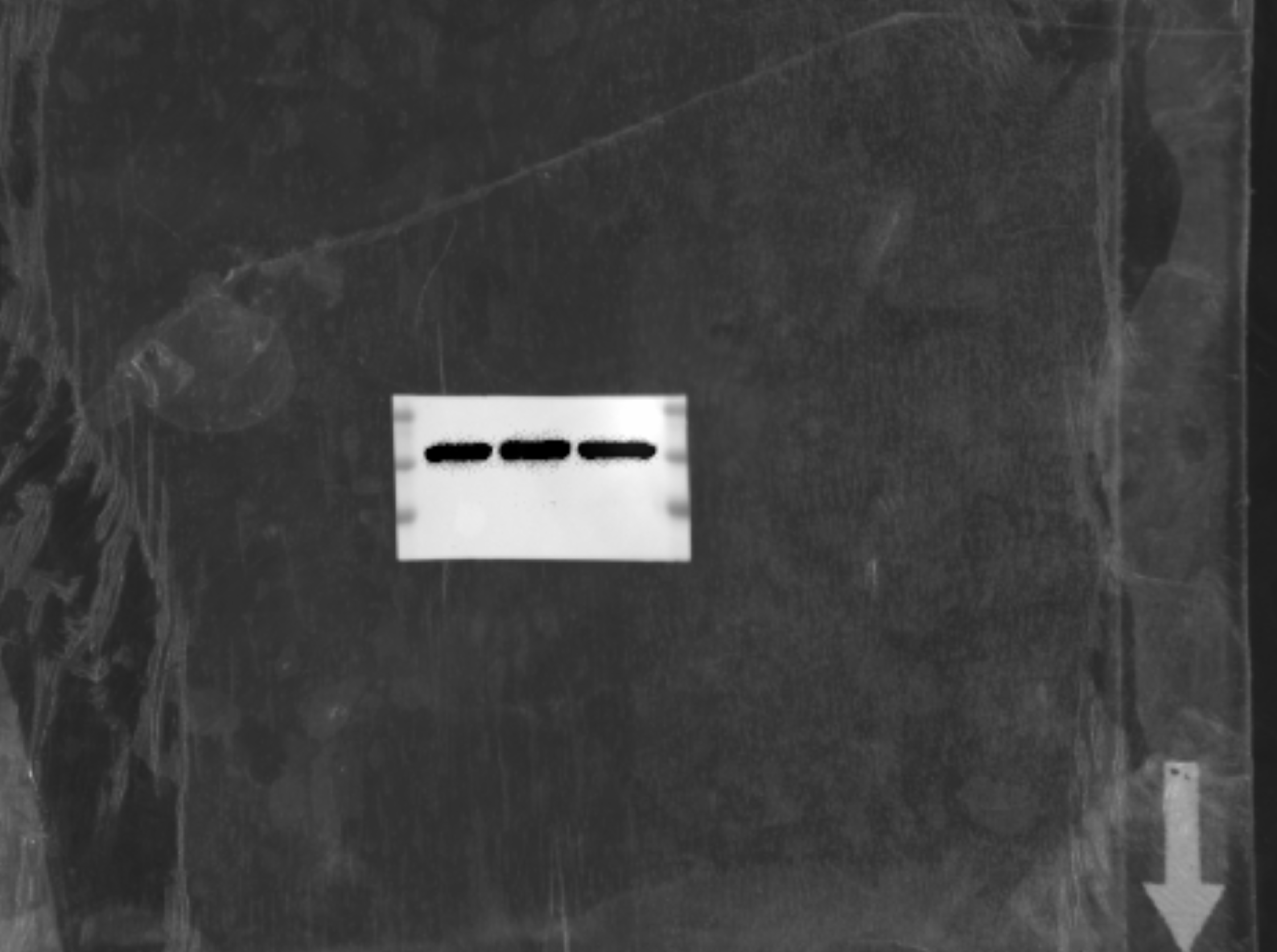

Supplement: Supplemental Information 39 — CAPN2 is involved in [brief function, e.g., cell migration/distinct proteolytic regulation compared to CAPN1] [file peerj-14-21375-s039.zip › Figure 5D WB RAW OE-KLHL40 CAPN2/3ACTIN+MARKER.tif]

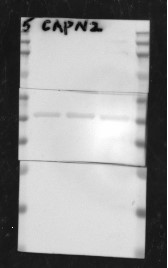

Supplement: Supplemental Information 39 — CAPN2 is involved in [brief function, e.g., cell migration/distinct proteolytic regulation compared to CAPN1] [file peerj-14-21375-s039.zip › Figure 5D WB RAW OE-KLHL40 CAPN2/3ALL.jpg]

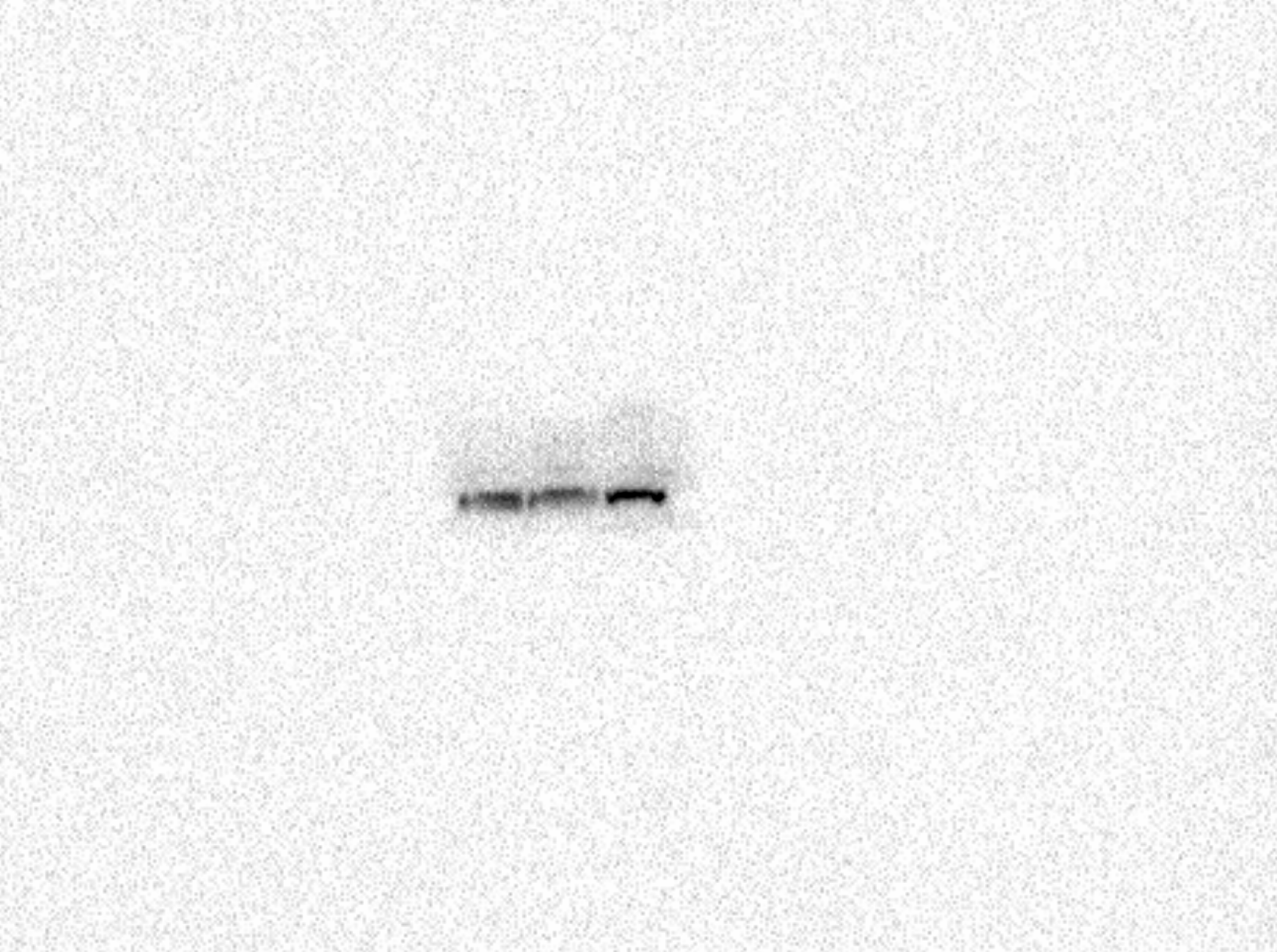

Supplement: Supplemental Information 39 — CAPN2 is involved in [brief function, e.g., cell migration/distinct proteolytic regulation compared to CAPN1] [file peerj-14-21375-s039.zip › Figure 5D WB RAW OE-KLHL40 CAPN2/3CAPN2.tif]

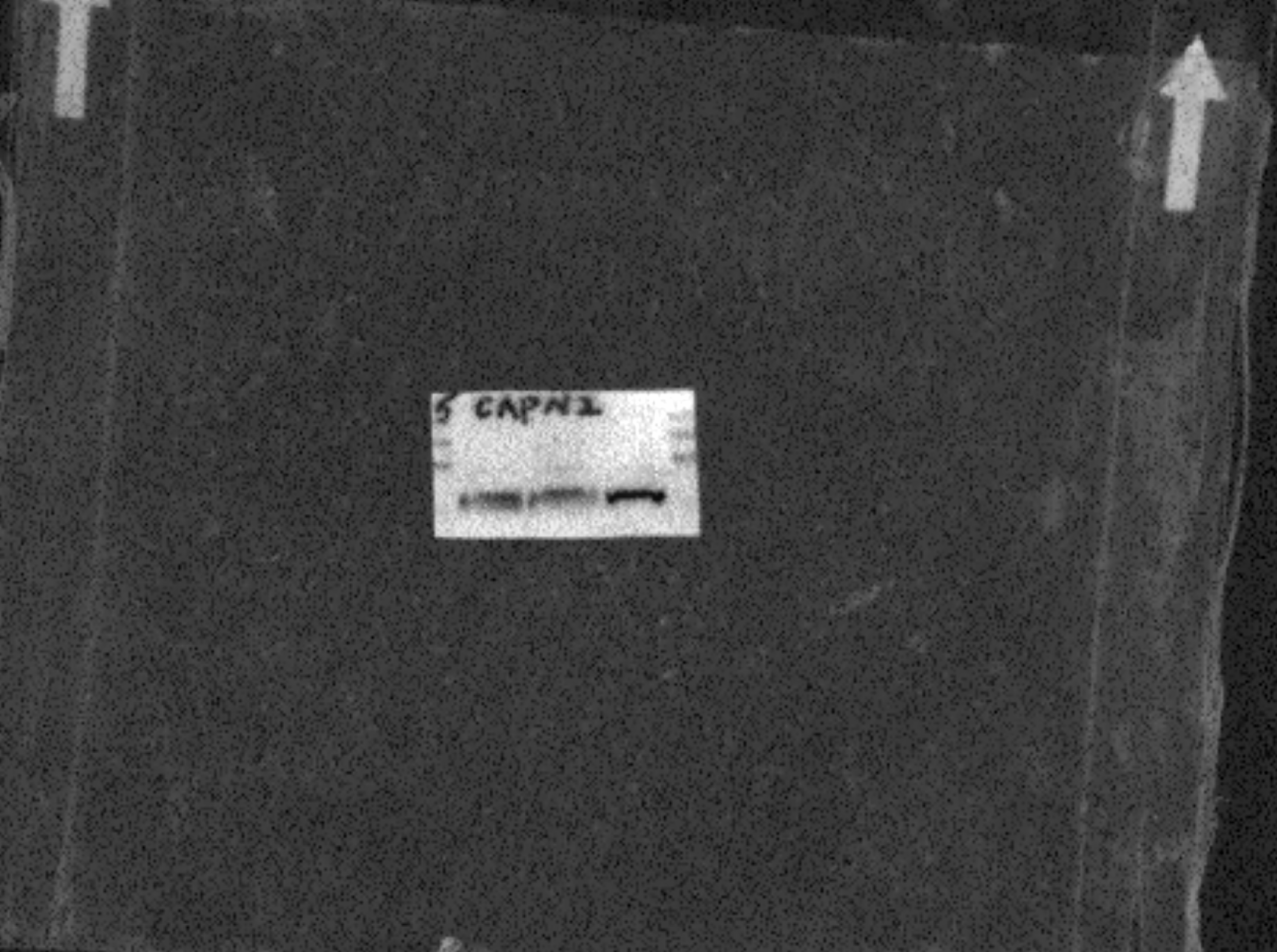

Supplement: Supplemental Information 39 — CAPN2 is involved in [brief function, e.g., cell migration/distinct proteolytic regulation compared to CAPN1] [file peerj-14-21375-s039.zip › Figure 5D WB RAW OE-KLHL40 CAPN2/3CAPN2+MARKER.tif]

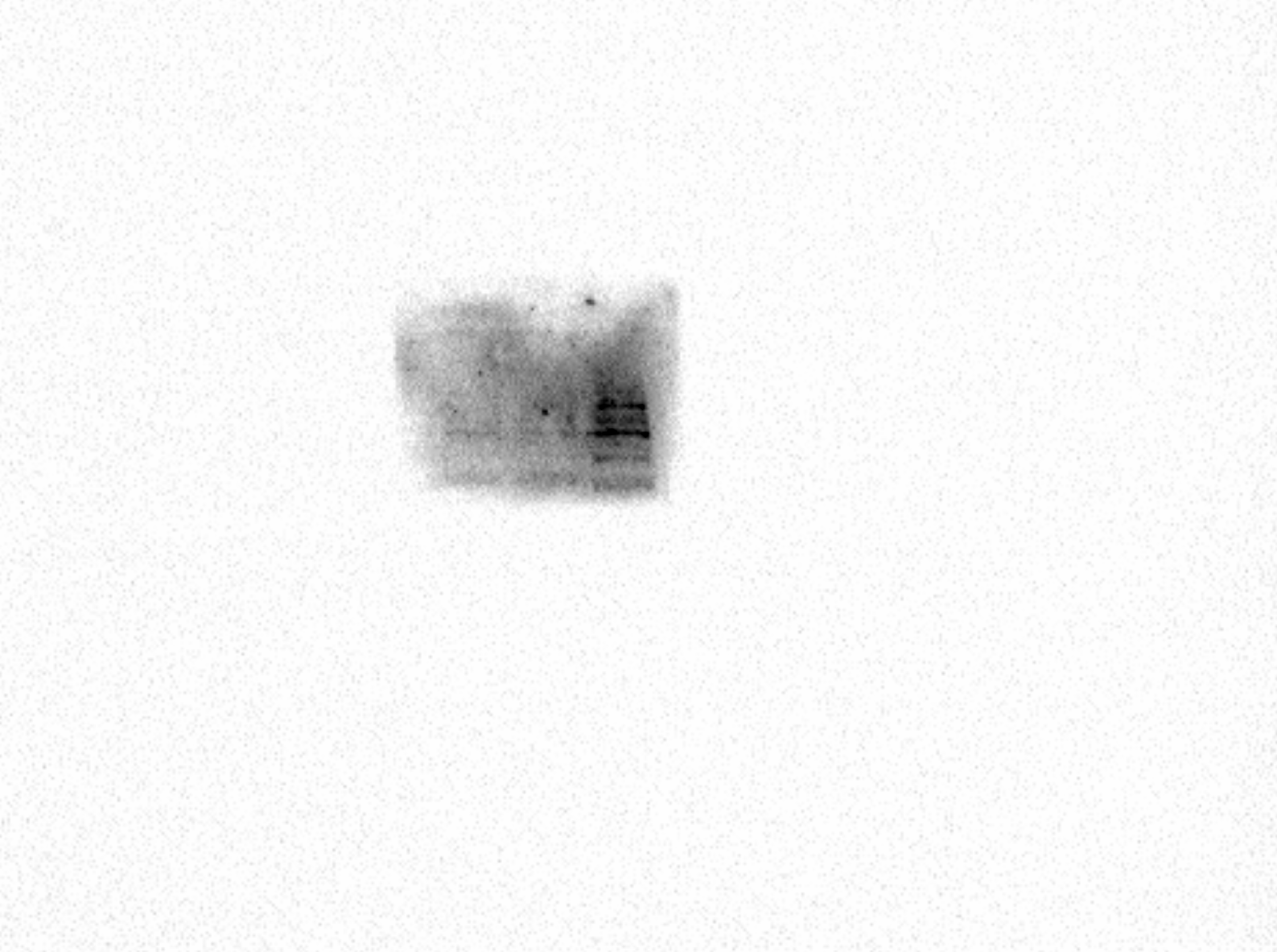

Supplement: Supplemental Information 40 [file peerj-14-21375-s040.zip › Figure 5E WB RAW SH-KLHL40 ATP2A2/ATP2A2-1 sh-KLHL40.tif]

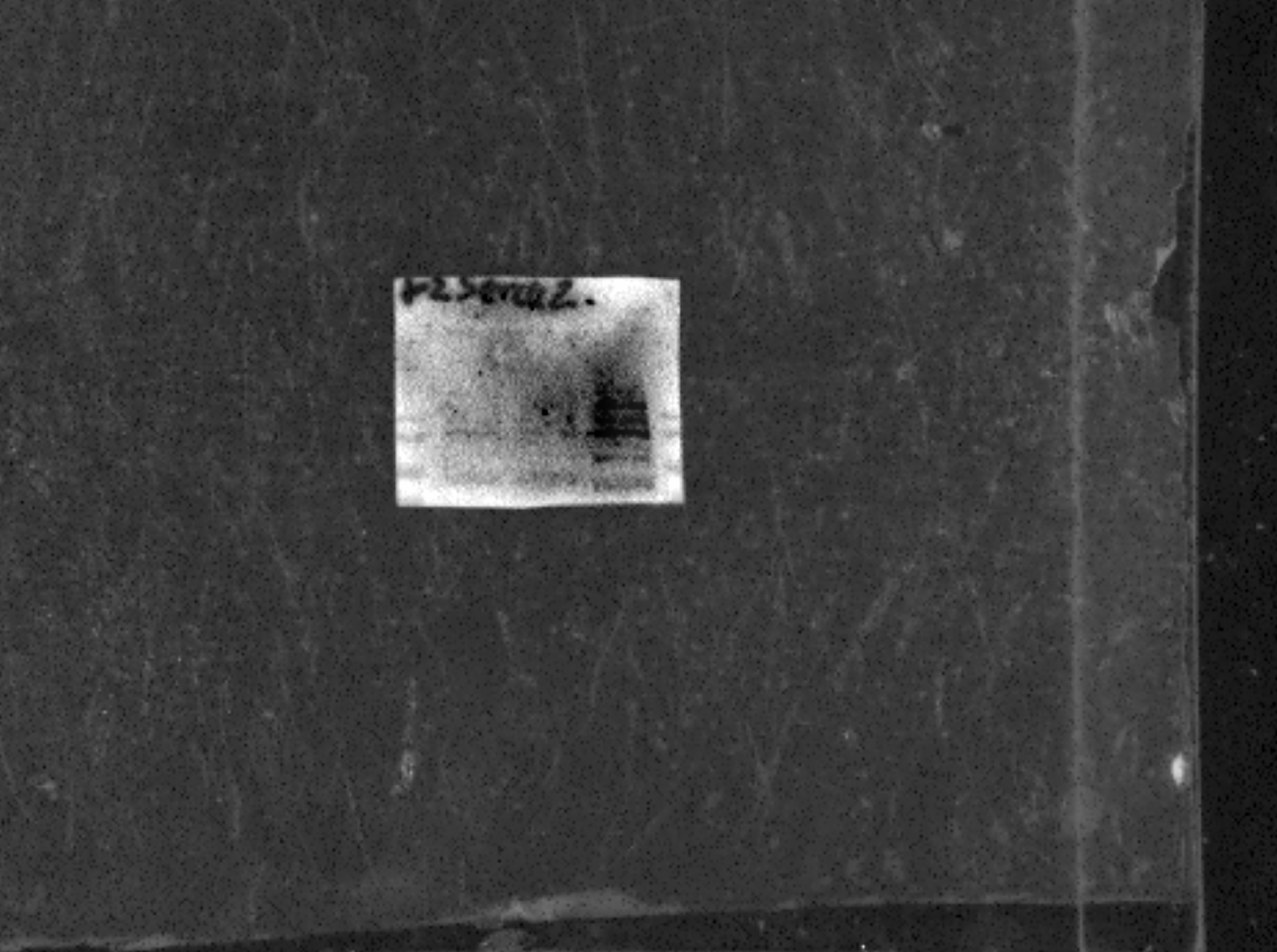

Supplement: Supplemental Information 40 [file peerj-14-21375-s040.zip › Figure 5E WB RAW SH-KLHL40 ATP2A2/ATP2A2-1 sh-KLHL40+MARK.tif]

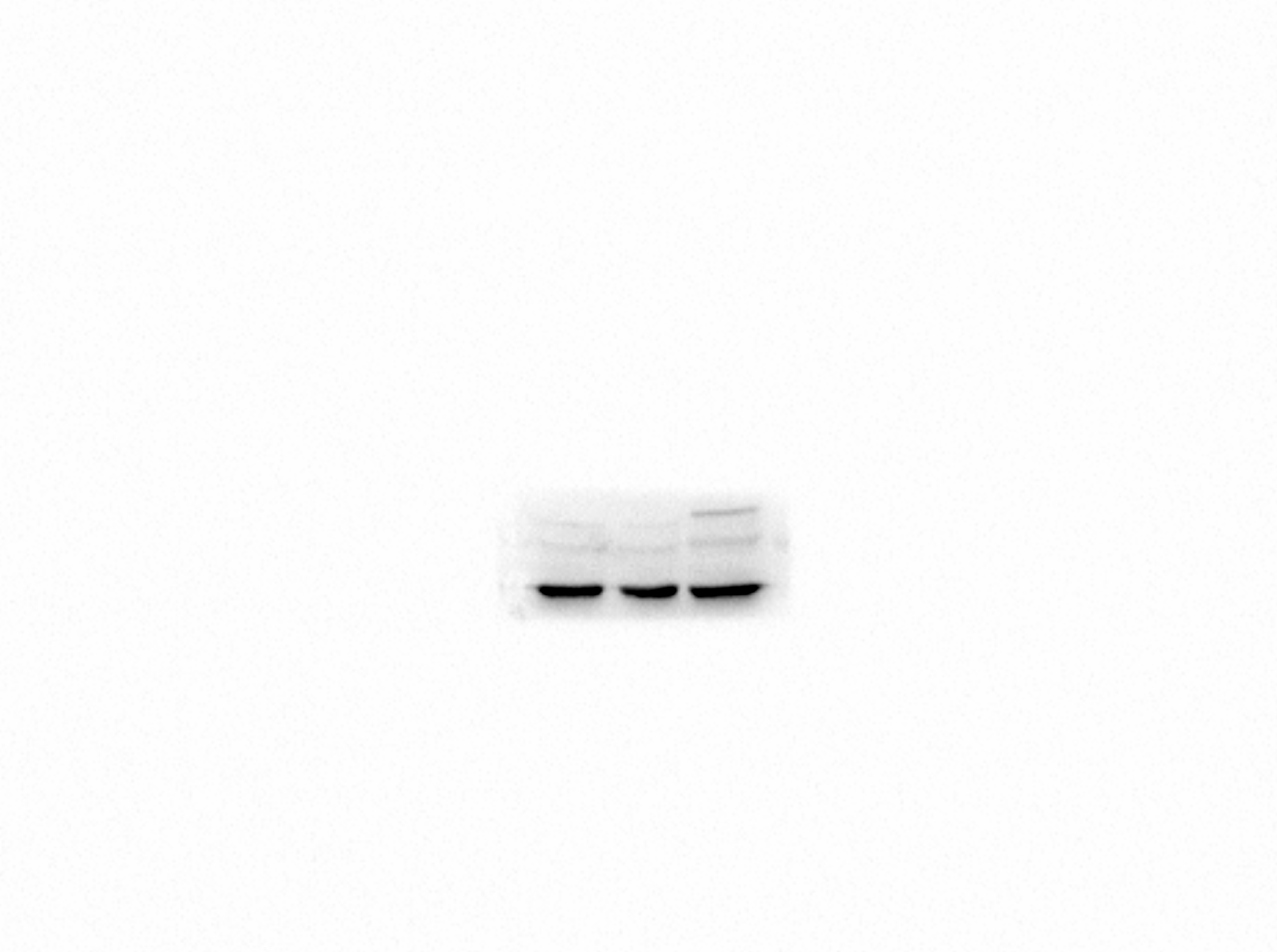

Supplement: Supplemental Information 40 [file peerj-14-21375-s040.zip › Figure 5E WB RAW SH-KLHL40 ATP2A2/ATP2A2-1 sh-KLHL40-ATCB.tif]

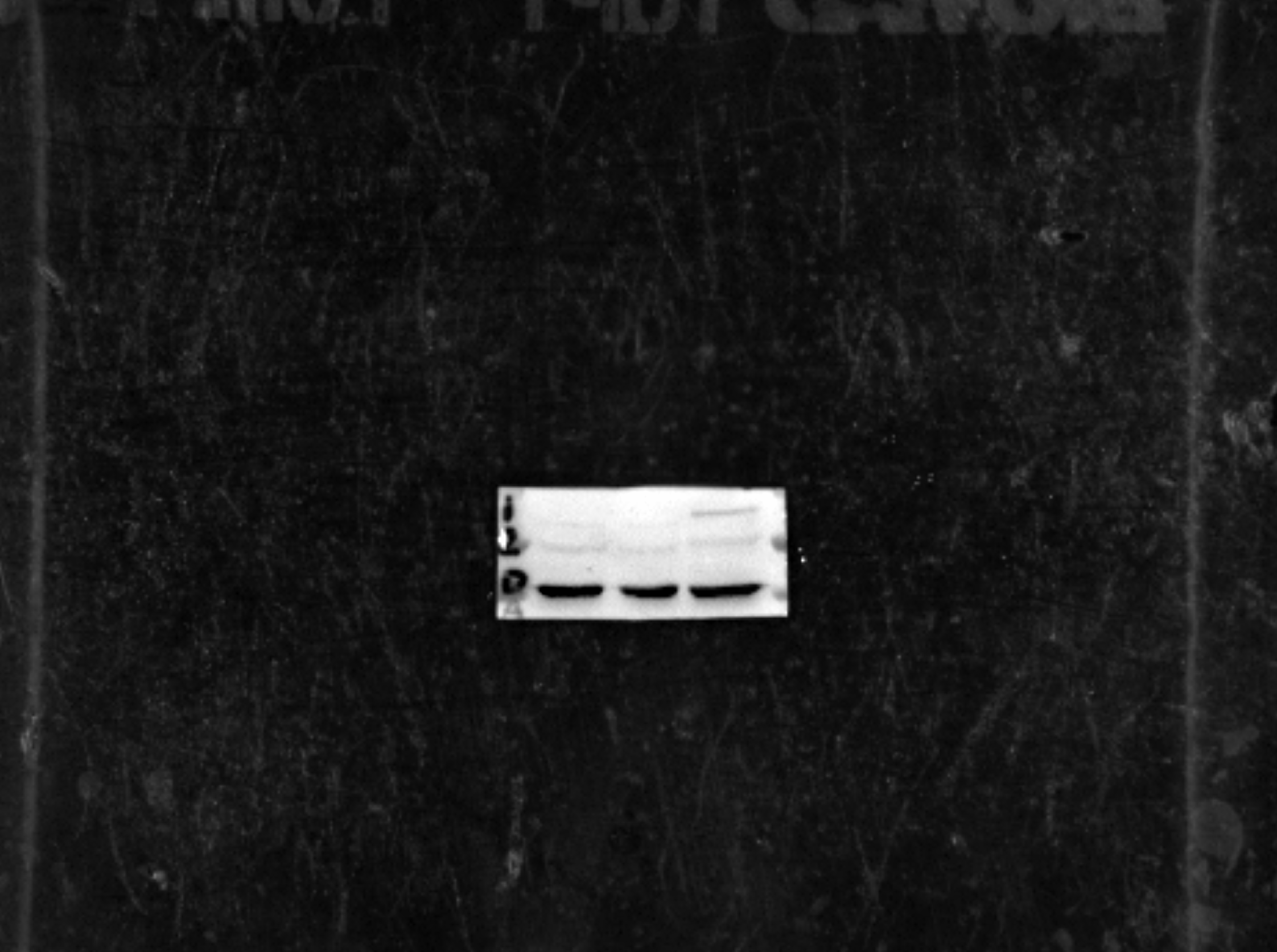

Supplement: Supplemental Information 40 [file peerj-14-21375-s040.zip › Figure 5E WB RAW SH-KLHL40 ATP2A2/ATP2A2-1 sh-KLHL40-ATCB+MARK.tif]

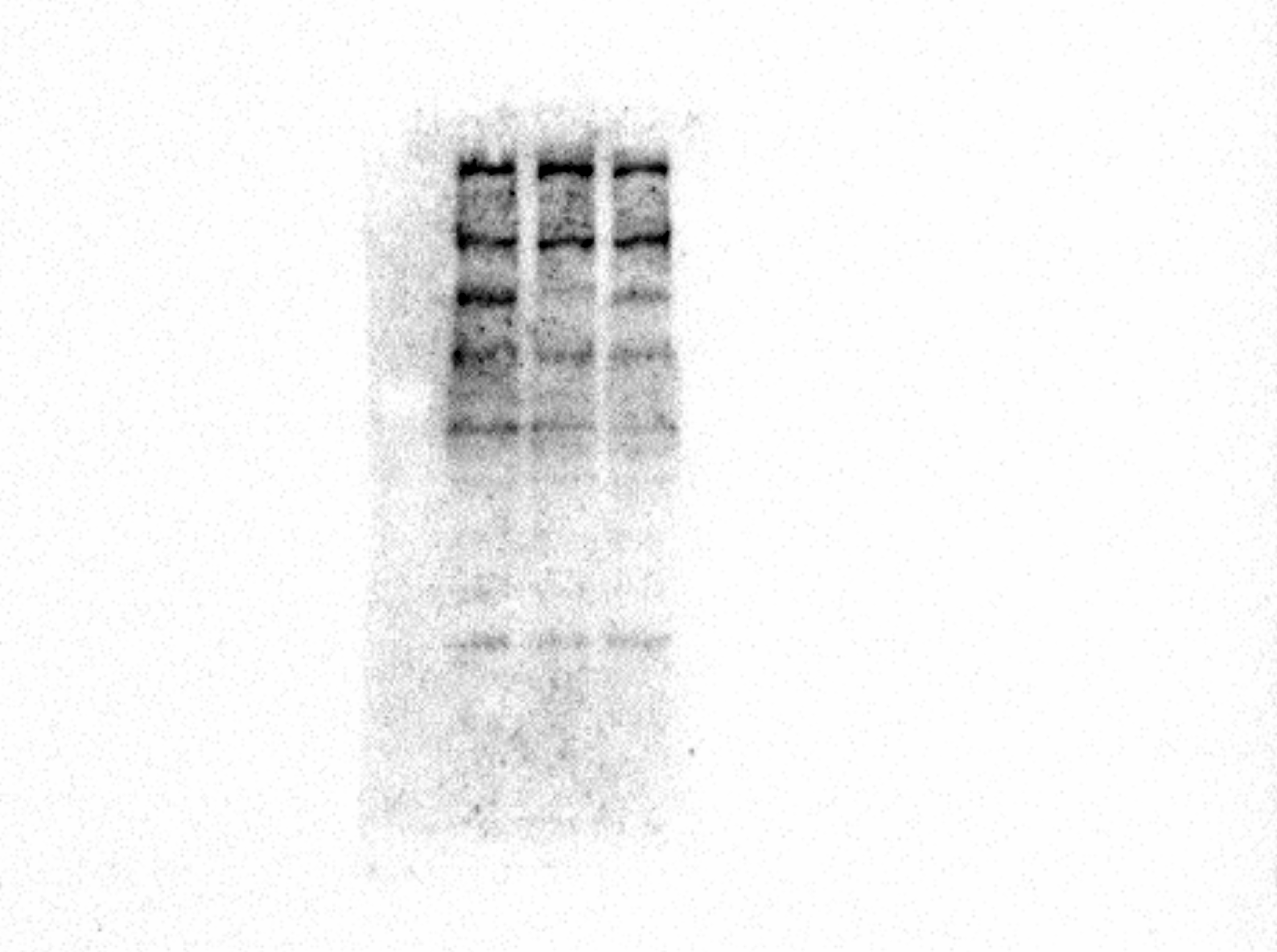

Supplement: Supplemental Information 40 [file peerj-14-21375-s040.zip › Figure 5E WB RAW SH-KLHL40 ATP2A2/ATP2A2-2 sh-KLHL40.tif]

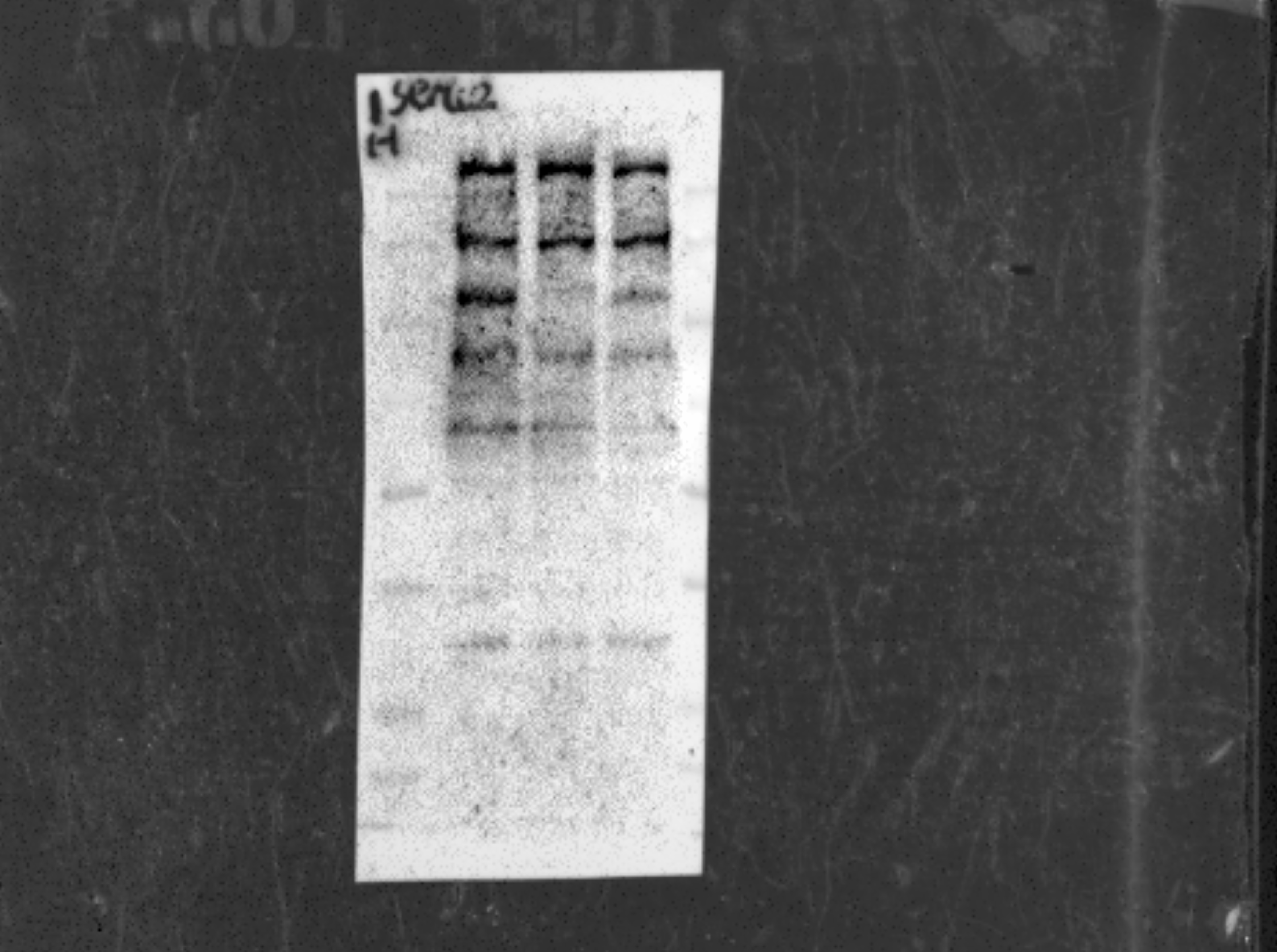

Supplement: Supplemental Information 40 [file peerj-14-21375-s040.zip › Figure 5E WB RAW SH-KLHL40 ATP2A2/ATP2A2-2 sh-KLHL40+MARK.tif]

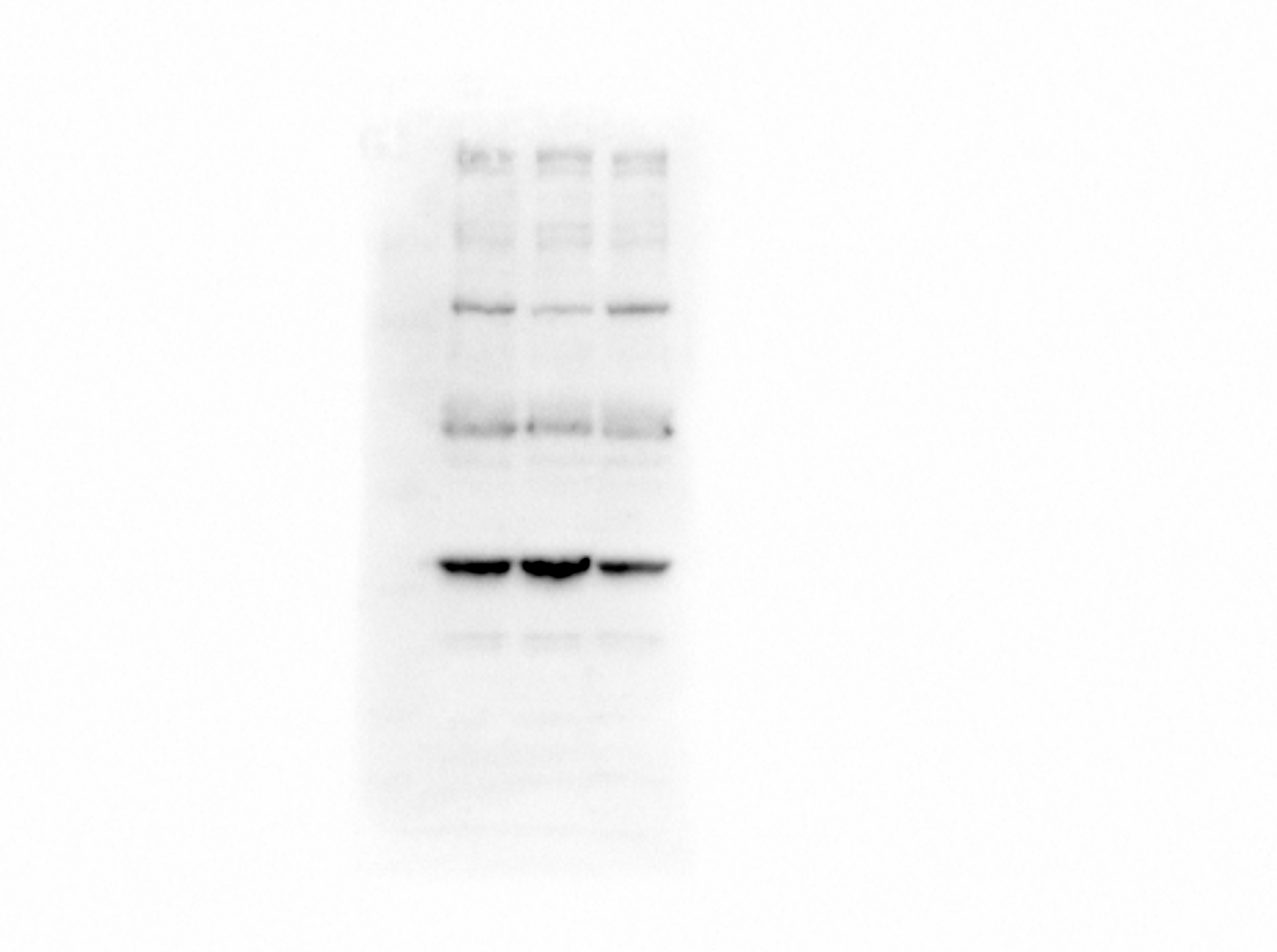

Supplement: Supplemental Information 40 [file peerj-14-21375-s040.zip › Figure 5E WB RAW SH-KLHL40 ATP2A2/ATP2A2-2 sh-KLHL40-ATCB.tif]
