# Supplementary material for: Post-infarction KLHL40-mediated regulation of cardiac sarcomeric integrity and function
Source: PeerJ. 2026 Jun 5;14:e21375. doi: 10.7717/peerj.21375 (PMC13245431; doi:10.7717/peerj.21375)
Supplement: Supplemental Information 34 [file peerj-14-21375-s034.zip › Figure 5 Labeled Western blot.docx]

Figure 5A SH-KLHL40 CAST

| C-Caspase1 | C-Caspase1 | β-ACTIN | β-ACTIN+marker | ALL |
| --- | --- | --- | --- | --- |
| 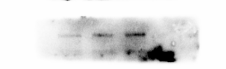 | 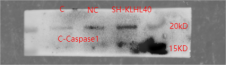 | 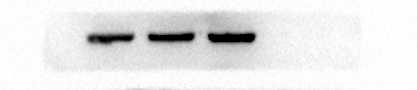 | 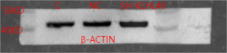 | 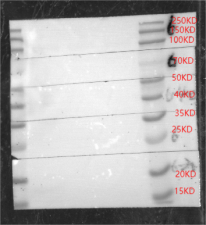 |
| 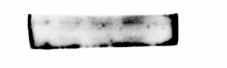 | 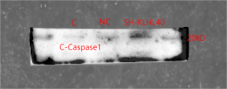 | 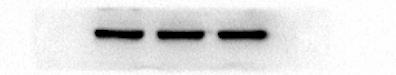 | 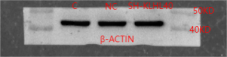 | 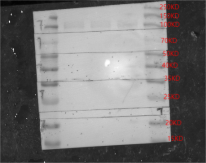 |
| 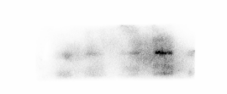 | 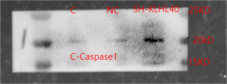 | 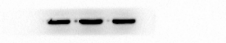 | 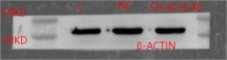 | 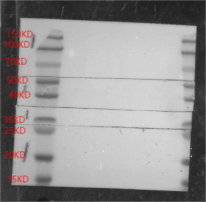 |

Figure 5B OE-KLHL40 CAST

| C-CASPASE1 | C-CASPASE1+MARKER | β-ACTIN | β-ACTIN+MARKER | ALL |
| --- | --- | --- | --- | --- |
| 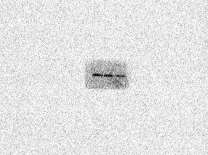 | 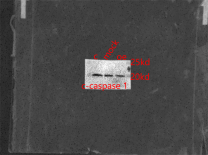 | 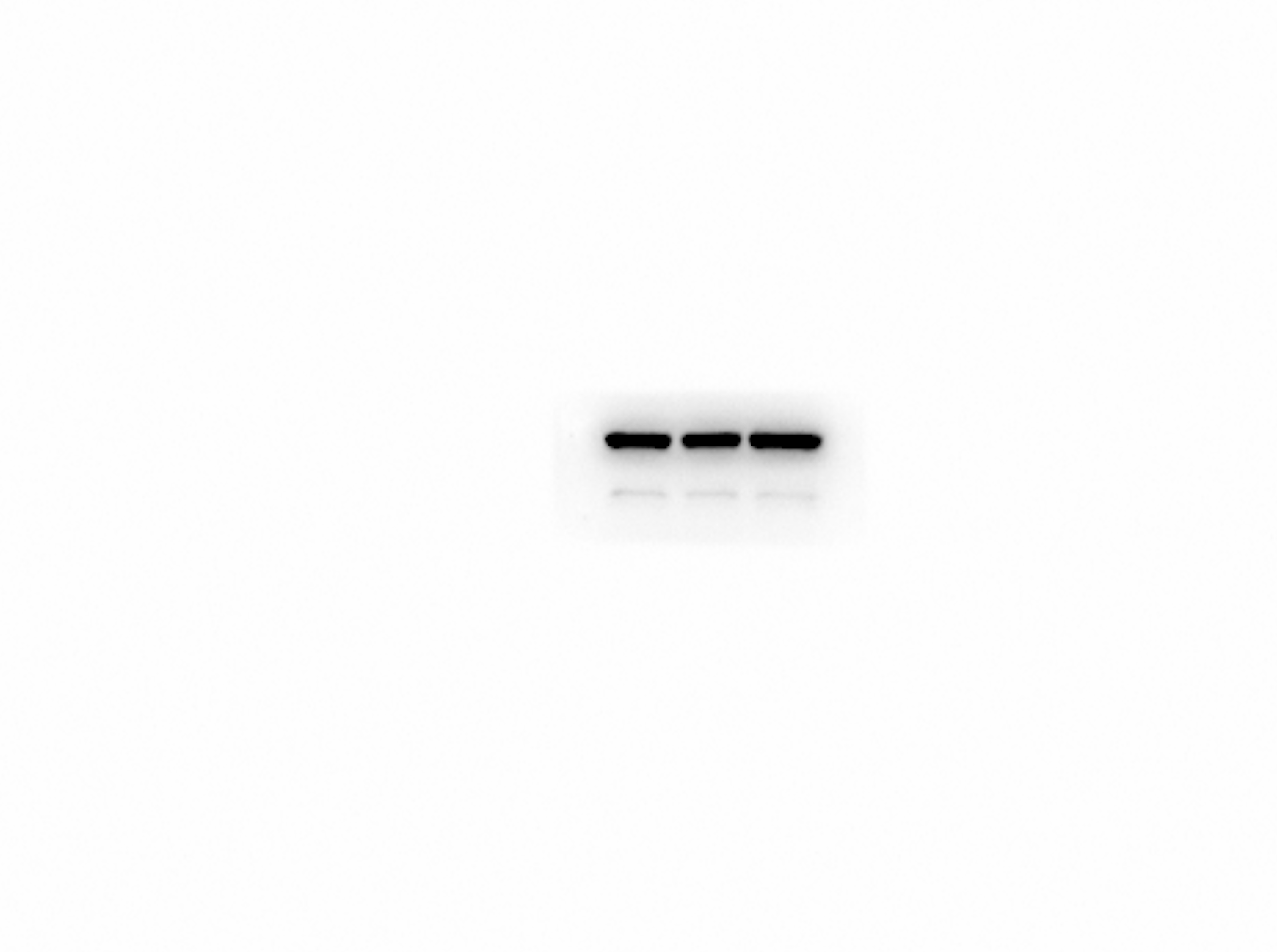 | 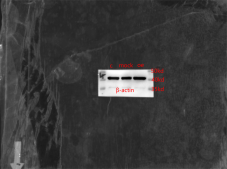 | 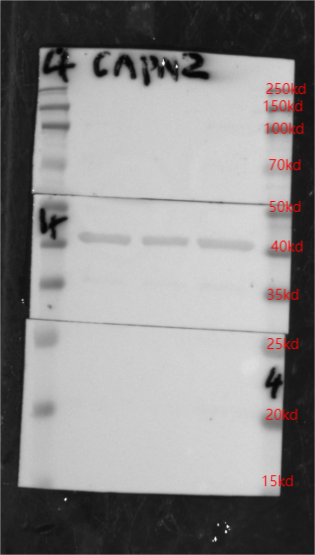 |
| 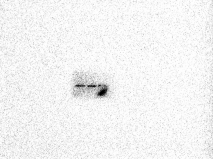 | 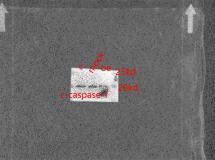 | 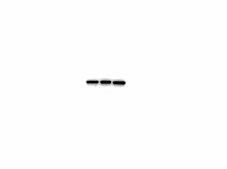 | 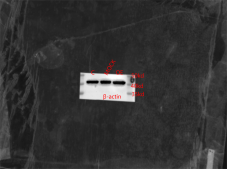 | 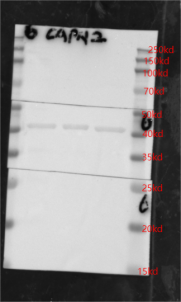 |
| 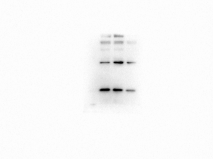 | 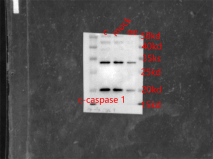 | 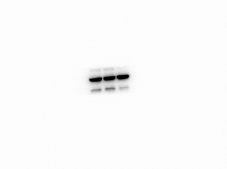 | 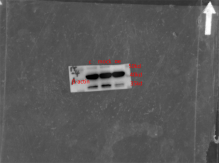 | 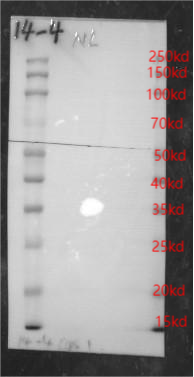 |

Figure 5C SH-KLHL40 CAPN1 CAPN2

| CAPN1 | CAPN1+MARKER | β-ACTIN | β-ACTIN+marker | ALL |
| --- | --- | --- | --- | --- |
| 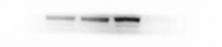 | 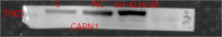 | 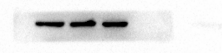 | 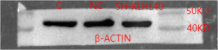 | 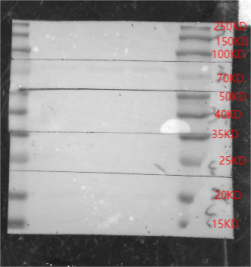 |
| 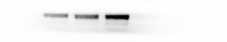 | 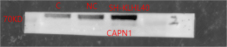 | 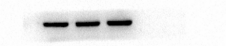 | 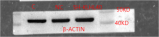 | 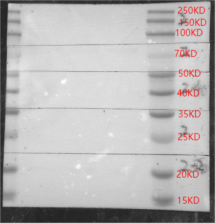 |
| 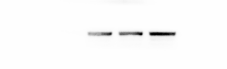 | 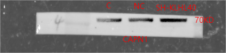 | 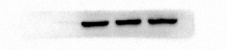 | 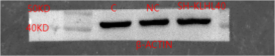 | 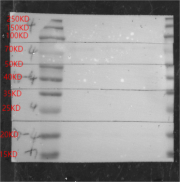 |
|  |  |  |  |  |
| CAPN2 | CAPN2+MARKER | β-ACTIN | β-ACTIN+marker | ALL |
| 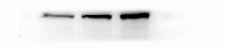 | 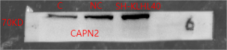 | 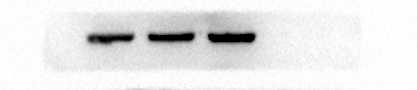 | 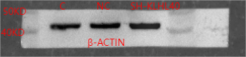 | 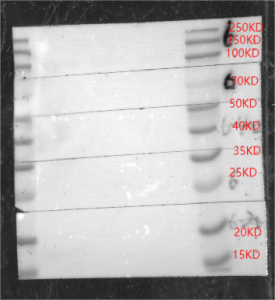 |
| 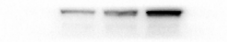 | 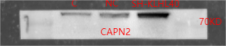 | 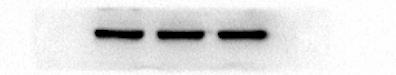 | 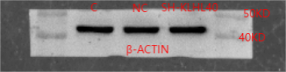 | 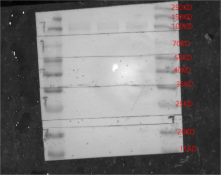 |
| 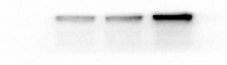 | 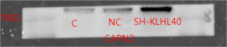 | 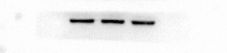 | 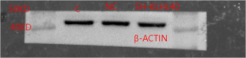 | 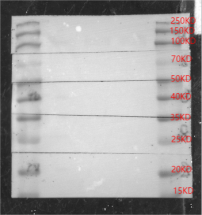 |

Figure 5D OE-KLHL40 CAPN1 CAPN2

| CAPN1 | CAPN1+MARKER | β-ACTIN | β-ACTIN+MARKER | ALL |
| --- | --- | --- | --- | --- |
| 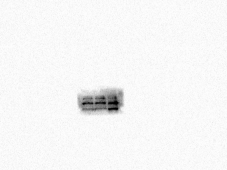 | 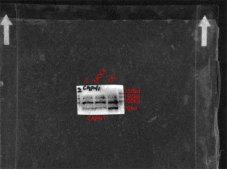 | 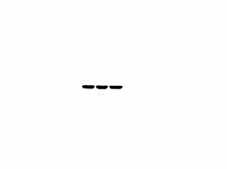 | 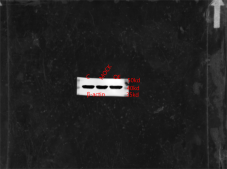 | 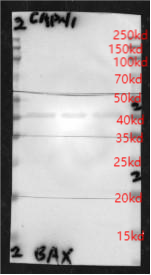 |
| 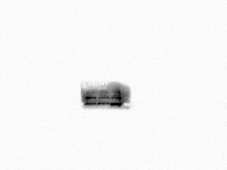 | 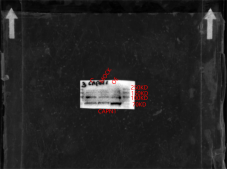 | 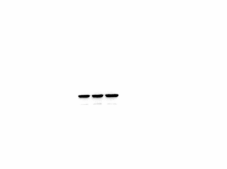 | 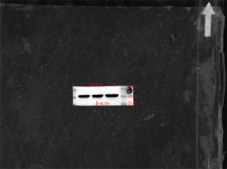 | 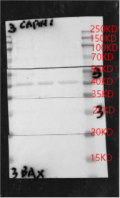 |
| 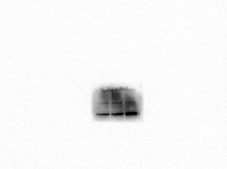 | 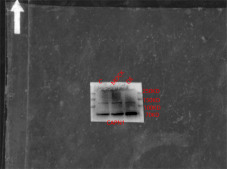 | 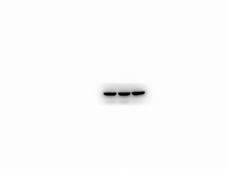 | 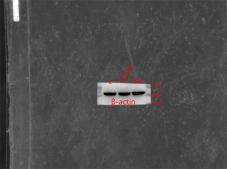 | 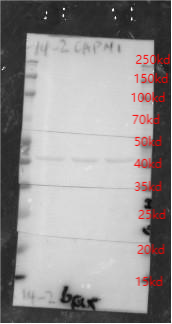 |

| CAPN2 | CAPN2+MARKER | β-ACTIN | β-ACTIN+MARKER | ALL |
| --- | --- | --- | --- | --- |
| 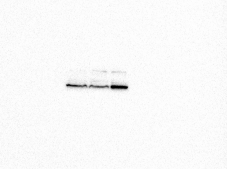 | 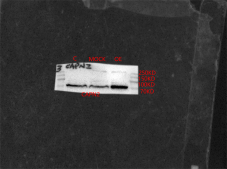 | 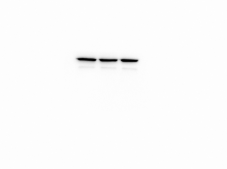 | 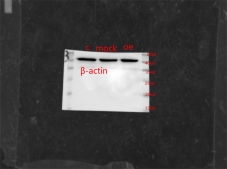 | 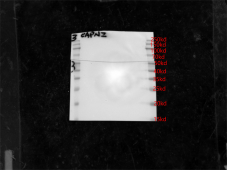 |
| 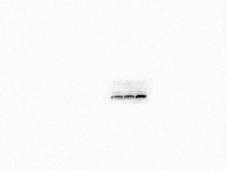 | 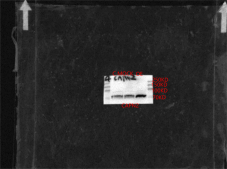 | 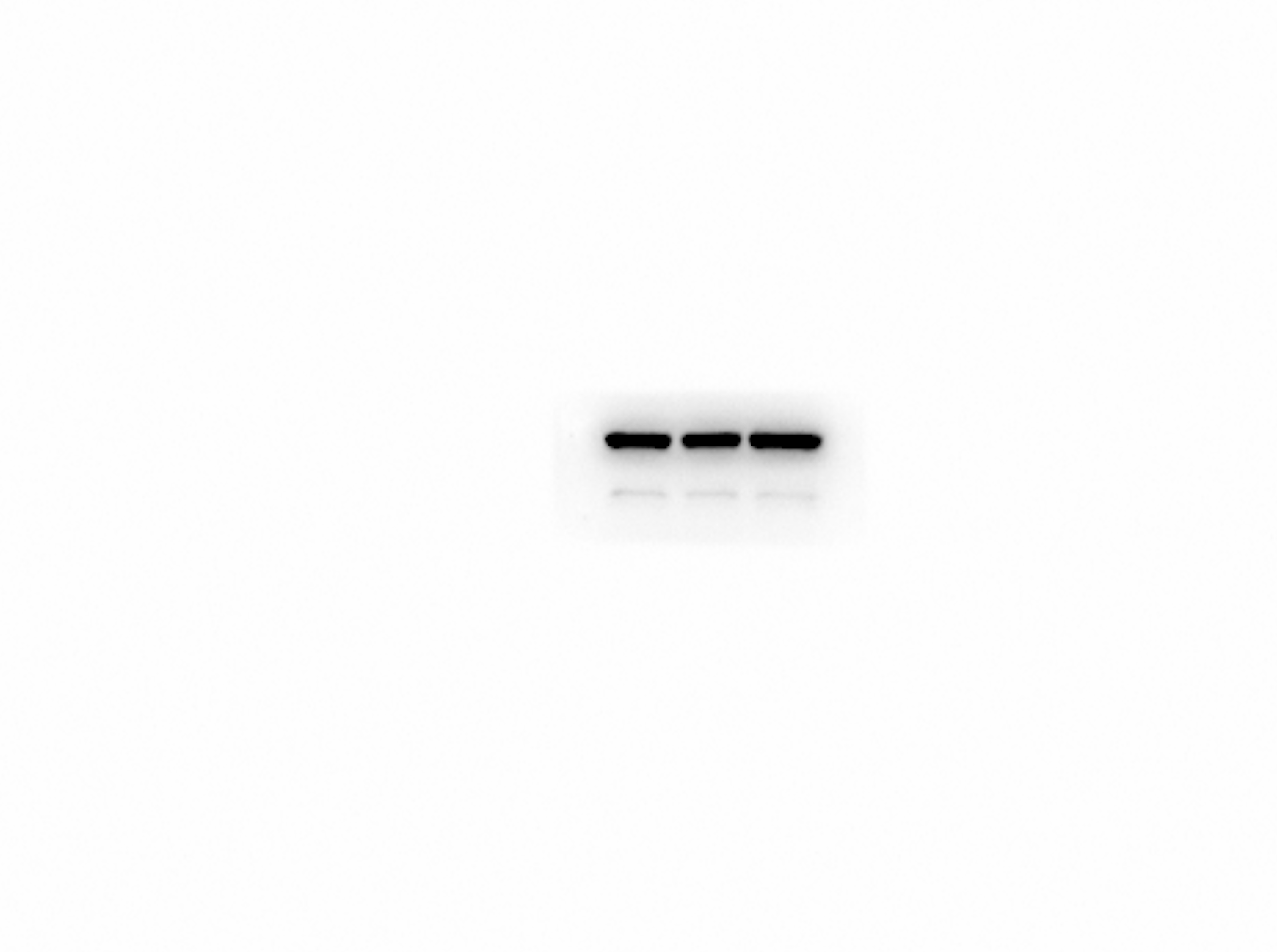 | 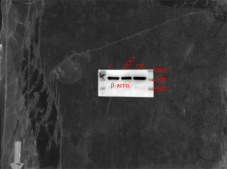 | 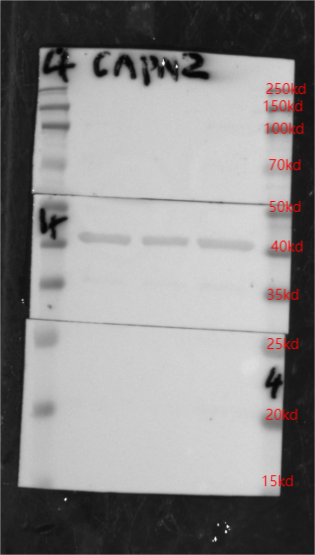 |
| 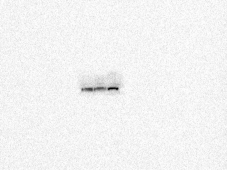 | 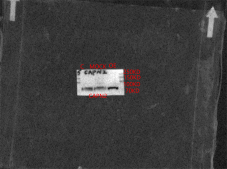 | 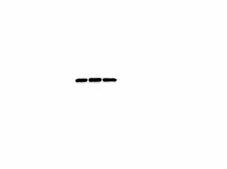 | 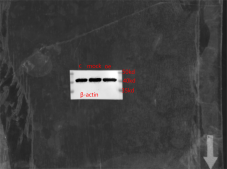 | 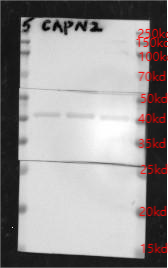 |

#

# Figure. 5E Wb ATP2A2 sh-KLHL40

| **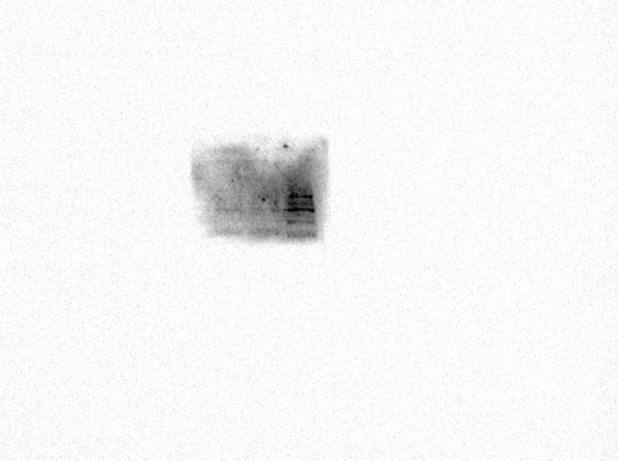** | **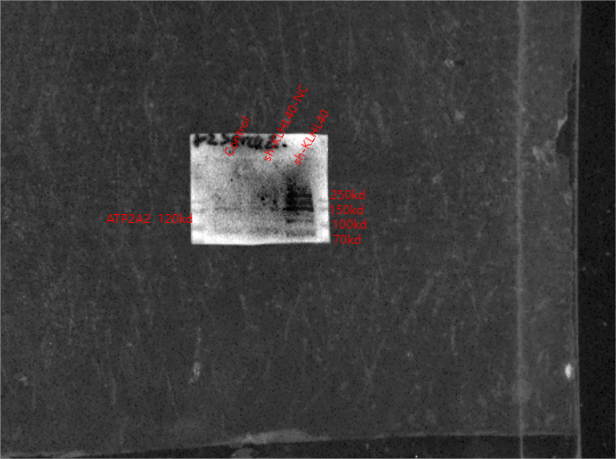** | 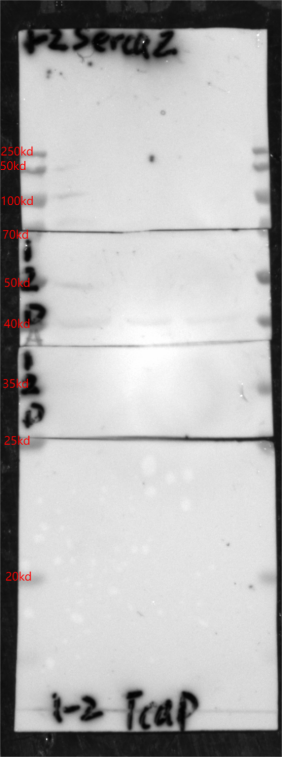 |
| --- | --- | --- |
| ATP2A2-1 sh-KLHL40 | ATP2A2-1 sh-KLHL40 MARK |  |
| 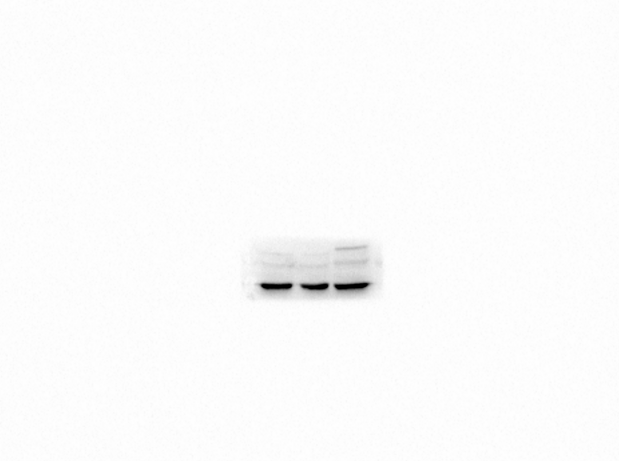 | 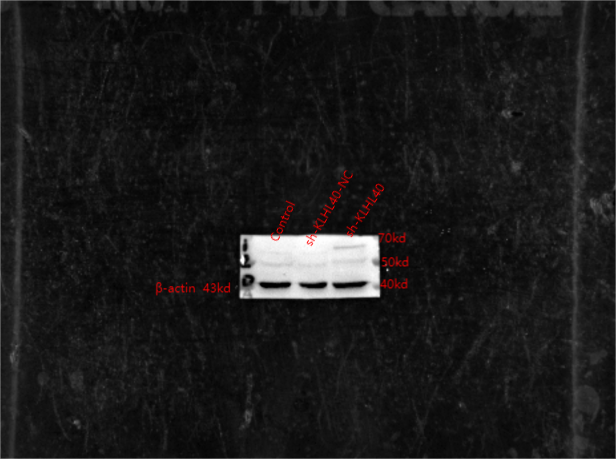 |  |
| ATP2A2-1 sh-KLHL40-ATCB | ATP2A2-1 sh-KLHL40-ATCB MARK | TOTAL |
| 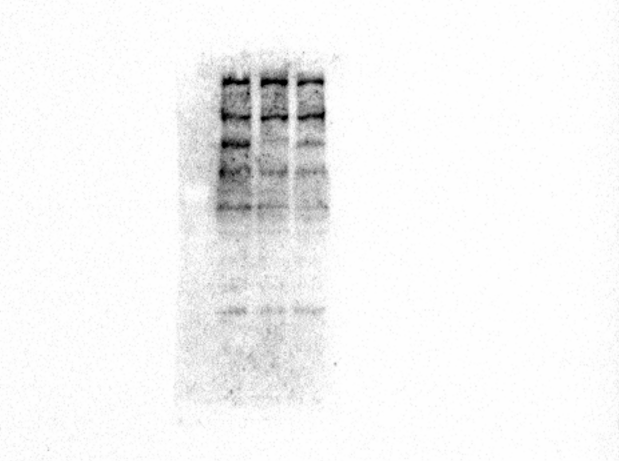 | 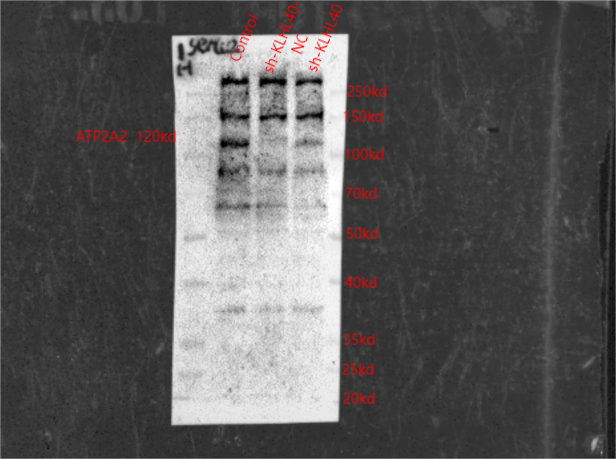 |  |
| ATP2A2-2 sh-KLHL40 | ATP2A2-2 sh-KLHL40 MARK |  |
| 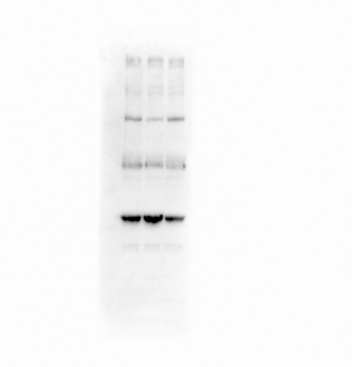 | 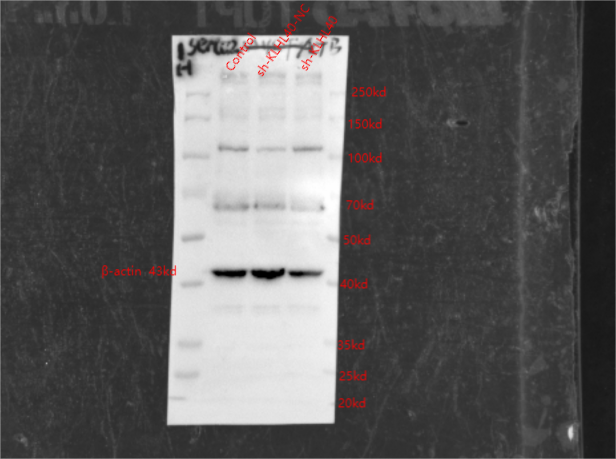 |  |
| ATP2A2-2 sh-KLHL40-ATCB | ATP2A2-2 sh-KLHL40-ATCB MARK |  |
| 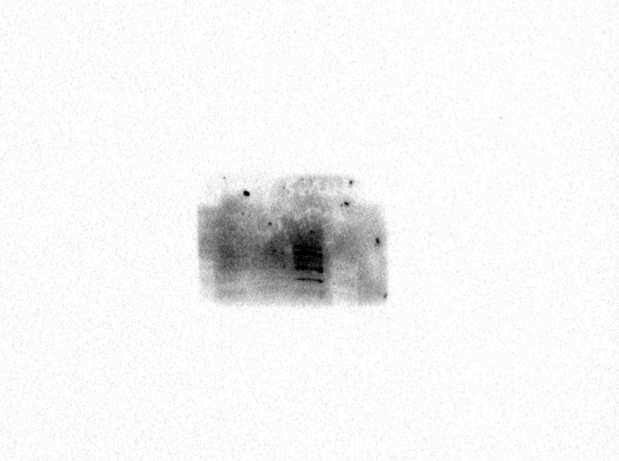 |  |  |
| ATP2A2-3 sh-KLHL40 | ATP2A2-3 sh-KLHL40 MARK |  |
|  |  |  |
| ATP2A2-3 sh-KLHL40-ATCB | ATP2A2-3 sh-KLHL40-ATCB MARK | TOTAL |

# Figure. 5F Wb ATP2A2 oe-KLHL40

|  |  |  |
| --- | --- | --- |
| ATP2A2-1 oe-KLHL40 | ATP2A2-1 oe-KLHL40 MARK |  |
|  |  |  |
| ATP2A2-1 oe-KLHL40-ACTB | ATP2A2-1 oe-KLHL40-ACTB MARK |  |
|  |  |  |
| ATP2A2-2 oe-KLHL40 | ATP2A2-2 oe-KLHL40 MARK |  |
|  |  |  |
| ATP2A2-2 oe-KLHL40-ACTB | ATP2A2-2 oe-KLHL40-ACTB MARK | TOTAL |
|  |  |  |
| ATP2A2-3 oe-KLHL40 | ATP2A2-3 oe-KLHL40 MARK |  |
|  |  |  |
| ATP2A2-3 oe-KLHL40-ACTB | ATP2A2-3 oe-KLHL40-ACTB MARK | TOTAL |

# Figure.5H Wb sh-KLHL40 DES

|  |  |
| --- | --- |
| DES-1 sh-KLHL40 | DES-1 sh-KLHL40 MARK |
|  |  |
| DES-1 sh-KLHL40-ACTB | DES-1 sh-KLHL40-ACTB MARK |
|  |  |
| DES-2 sh-KLHL40 | DES-2 sh-KLHL40 MARK |
|  |  |
| DES-2 sh-KLHL40-ACTB | DES-2 sh-KLHL40-ACTB MARK |
|  |  |
| DES-3 sh-KLHL40 | DES-3 sh-KLHL40 MARK |
|  |  |
| DES-3 sh-KLHL40-ACTB | DES-3 sh-KLHL40-ACTB MARK |

# Figure. 5I Wb oe-KLHL40 DES

|  |  |
| --- | --- |
| DES-1 oe-KLHL40 | DES-1 oe-KLHL40 MARK |
|  |  |
| DES-1 oe-KLHL40-ACTB | DES-1 oe-KLHL40-ACTB MARK |
|  |  |
| DES-2 oe-KLHL40 | DES-2 oe-KLHL40 MARK |
|  |  |
| DES-2 oe-KLHL40-ACTB | DES-2 oe-KLHL40-ACTB MARK |
|  |  |
| DES-3 oe-KLHL40 | DES-3 oe-KLHL40 MARK |
|  |  |
| DES-3 oe-KLHL40-ACTB | DES-3 oe-KLHL40-ACTB MARK |
