# Supplementary figures and images for: Post-infarction KLHL40-mediated regulation of cardiac sarcomeric integrity and function (part 4 of 5)
Source: PeerJ. 2026 Jun 5;14:e21375. doi: 10.7717/peerj.21375 (PMC13245431; doi:10.7717/peerj.21375)

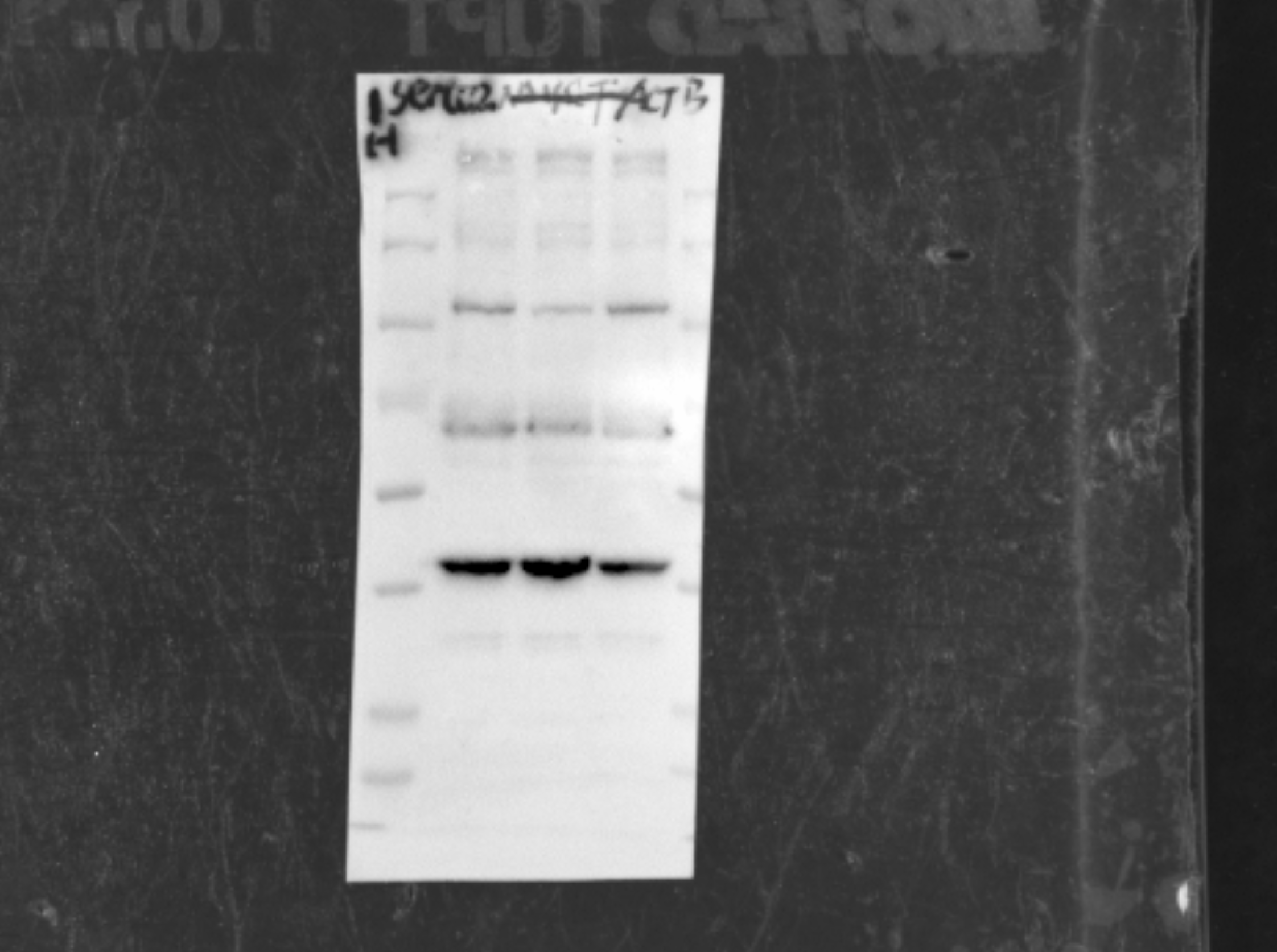

Supplement: Supplemental Information 40 [file peerj-14-21375-s040.zip › Figure 5E WB RAW SH-KLHL40 ATP2A2/ATP2A2-2 sh-KLHL40-ATCB+MARK.tif]

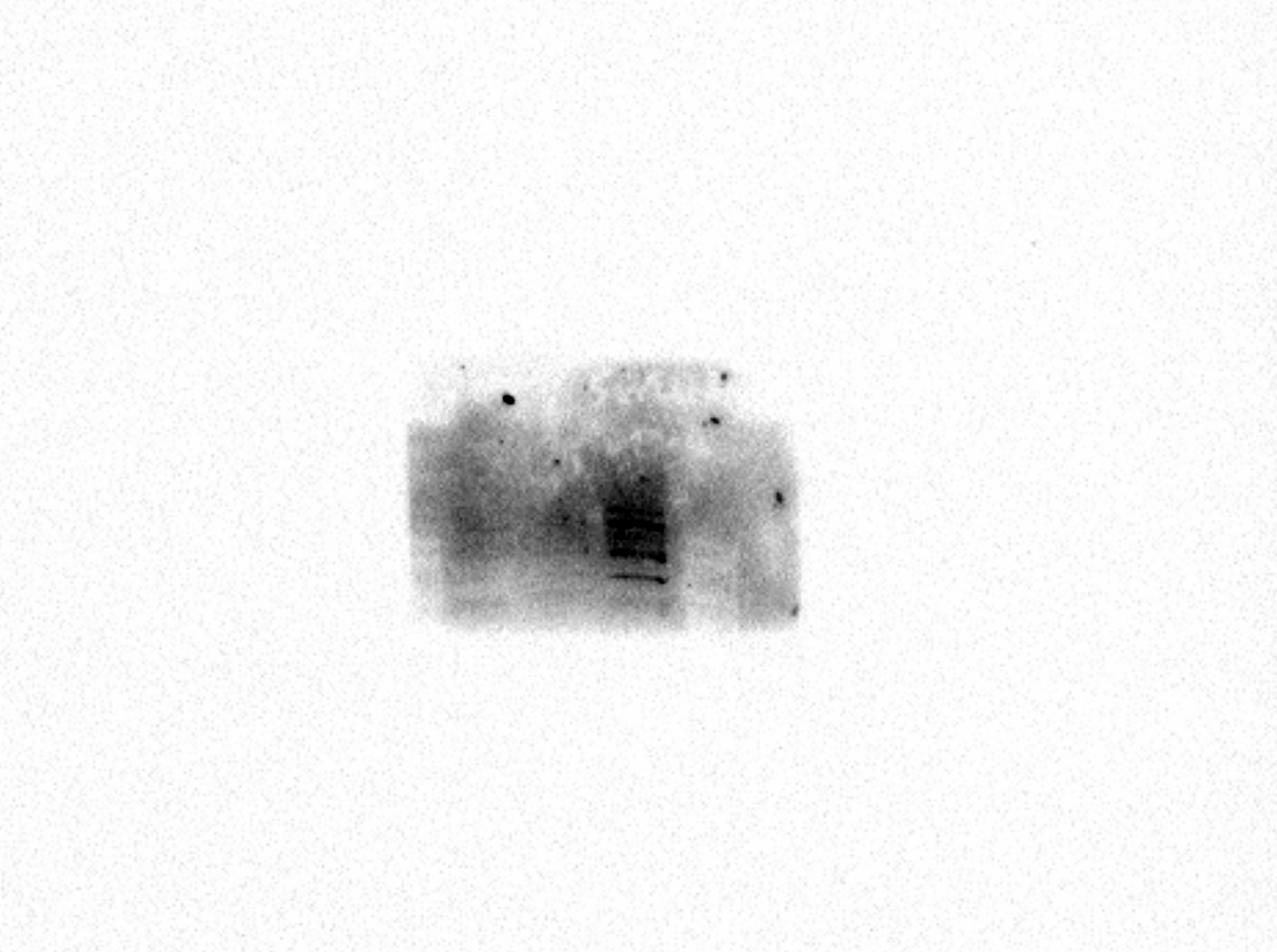

Supplement: Supplemental Information 40 [file peerj-14-21375-s040.zip › Figure 5E WB RAW SH-KLHL40 ATP2A2/ATP2A2-3 sh-KLHL40.tif]

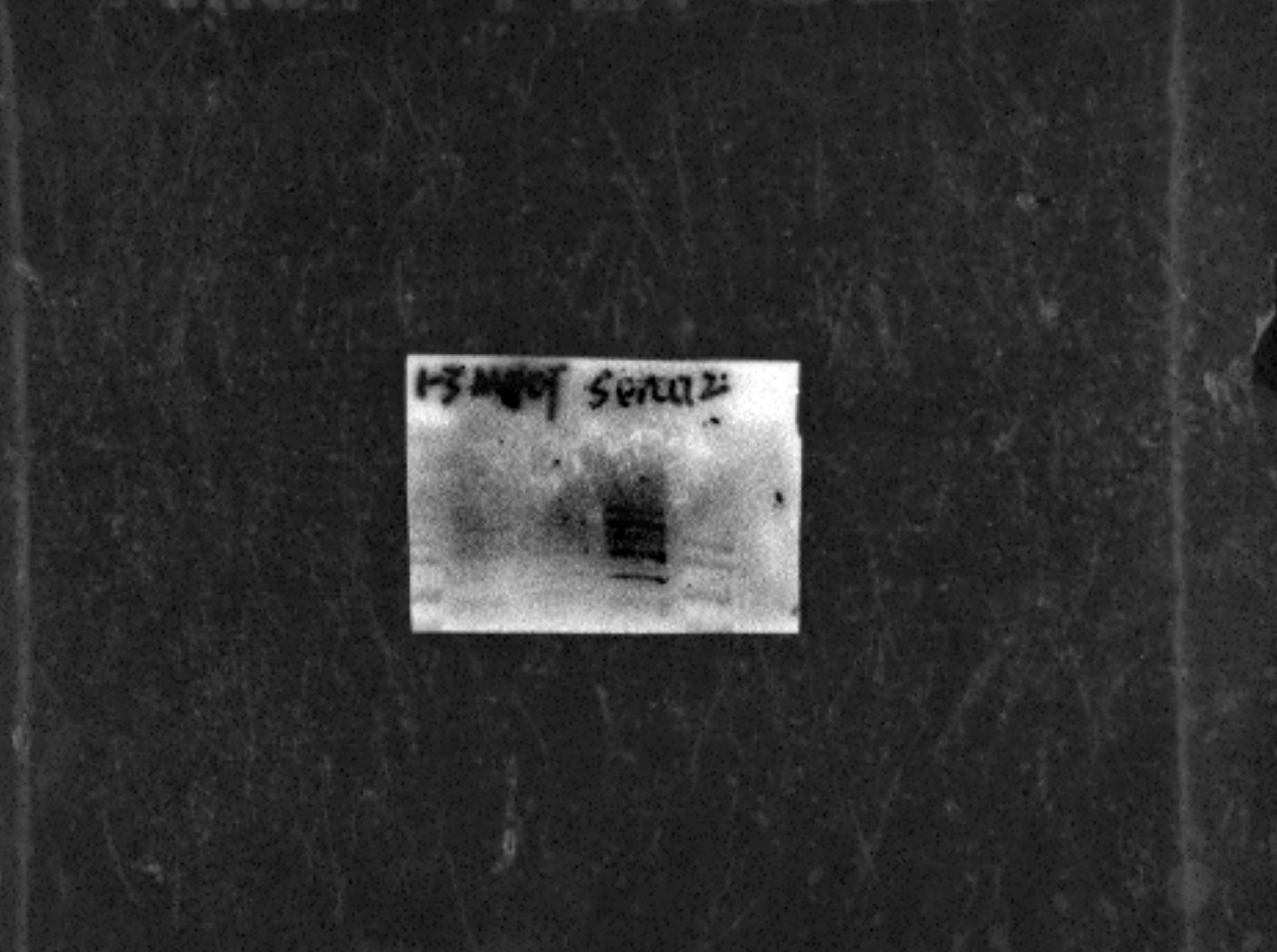

Supplement: Supplemental Information 40 [file peerj-14-21375-s040.zip › Figure 5E WB RAW SH-KLHL40 ATP2A2/ATP2A2-3 sh-KLHL40+MARK.tif]

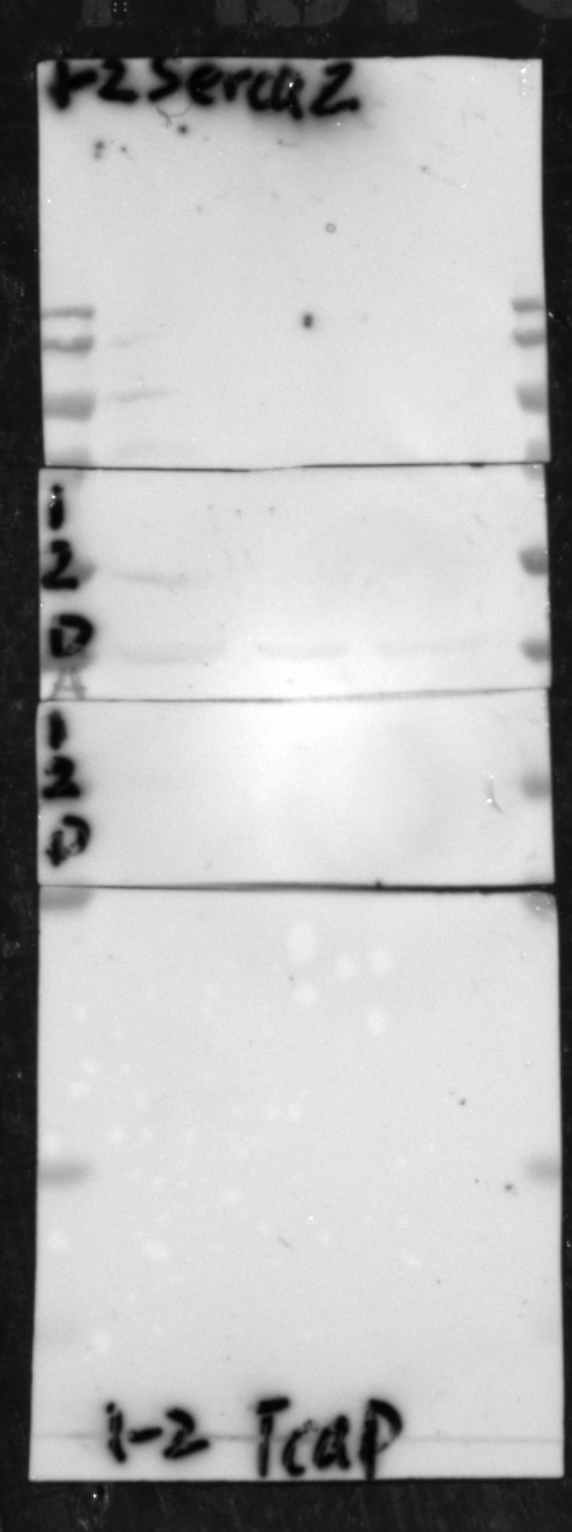

Supplement: Supplemental Information 40 [file peerj-14-21375-s040.zip › Figure 5E WB RAW SH-KLHL40 ATP2A2/TOTAL-1.tif]

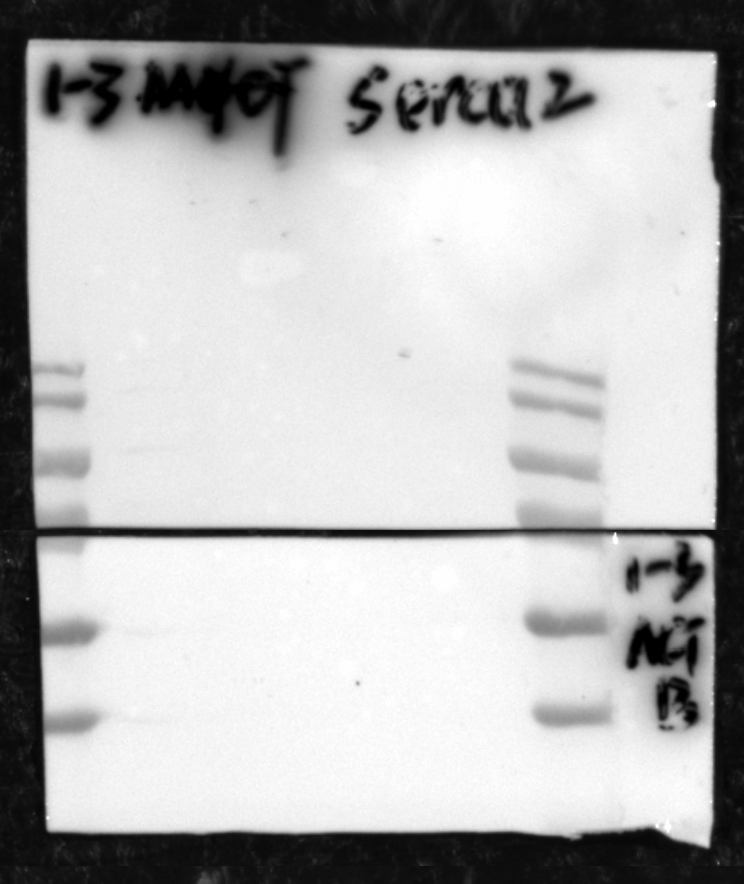

Supplement: Supplemental Information 40 [file peerj-14-21375-s040.zip › Figure 5E WB RAW SH-KLHL40 ATP2A2/TOTAL-3.tif]

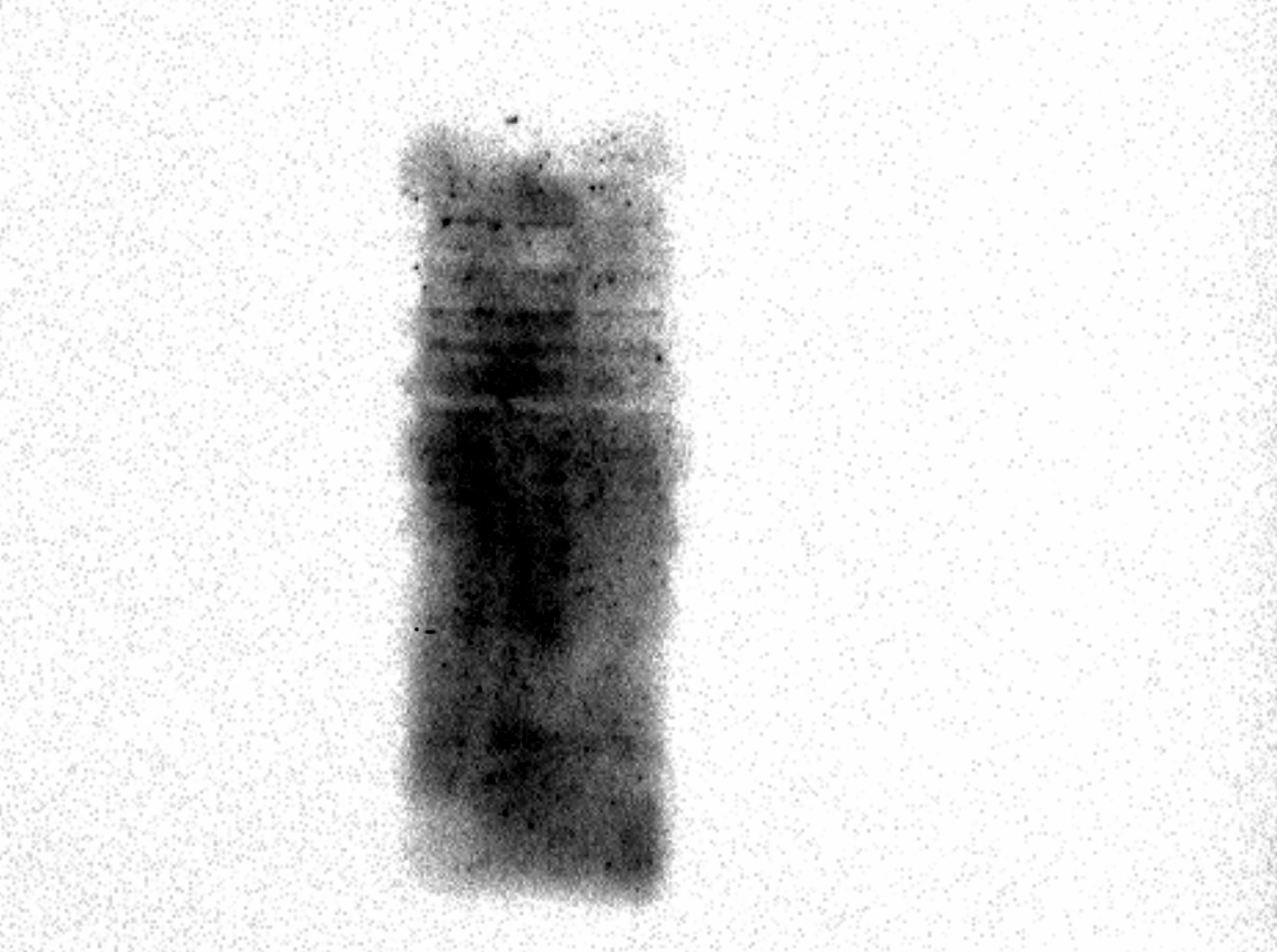

Supplement: Supplemental Information 41 [file peerj-14-21375-s041.zip › Figure 5F WB RAW OE-KLHL40 ATP2A2/ATP2A2-1 oe-KLHL40.tif]

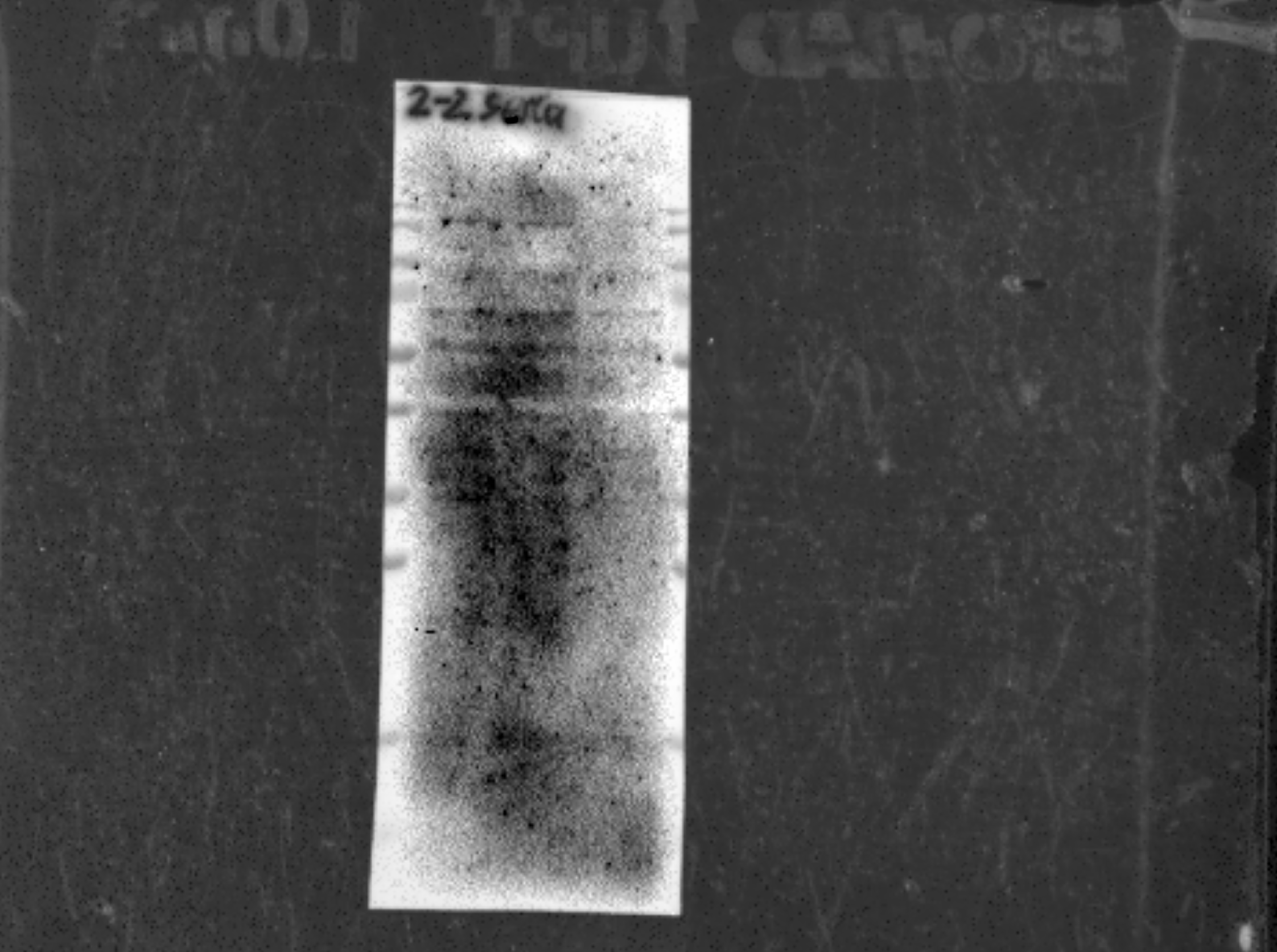

Supplement: Supplemental Information 41 [file peerj-14-21375-s041.zip › Figure 5F WB RAW OE-KLHL40 ATP2A2/ATP2A2-1 oe-KLHL40+MARK.tif]

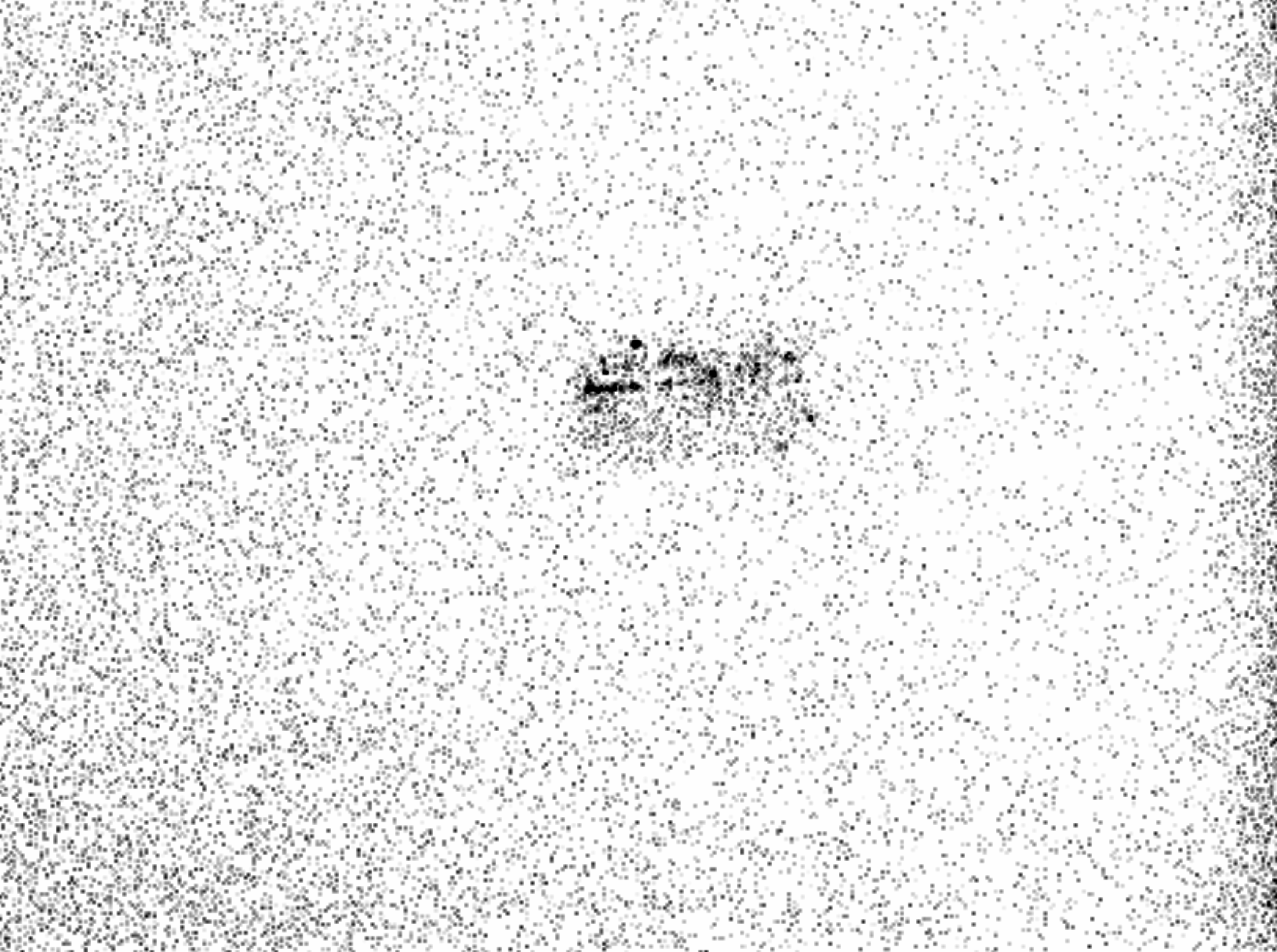

Supplement: Supplemental Information 41 [file peerj-14-21375-s041.zip › Figure 5F WB RAW OE-KLHL40 ATP2A2/ATP2A2-2 oe-KLHL40.tif]

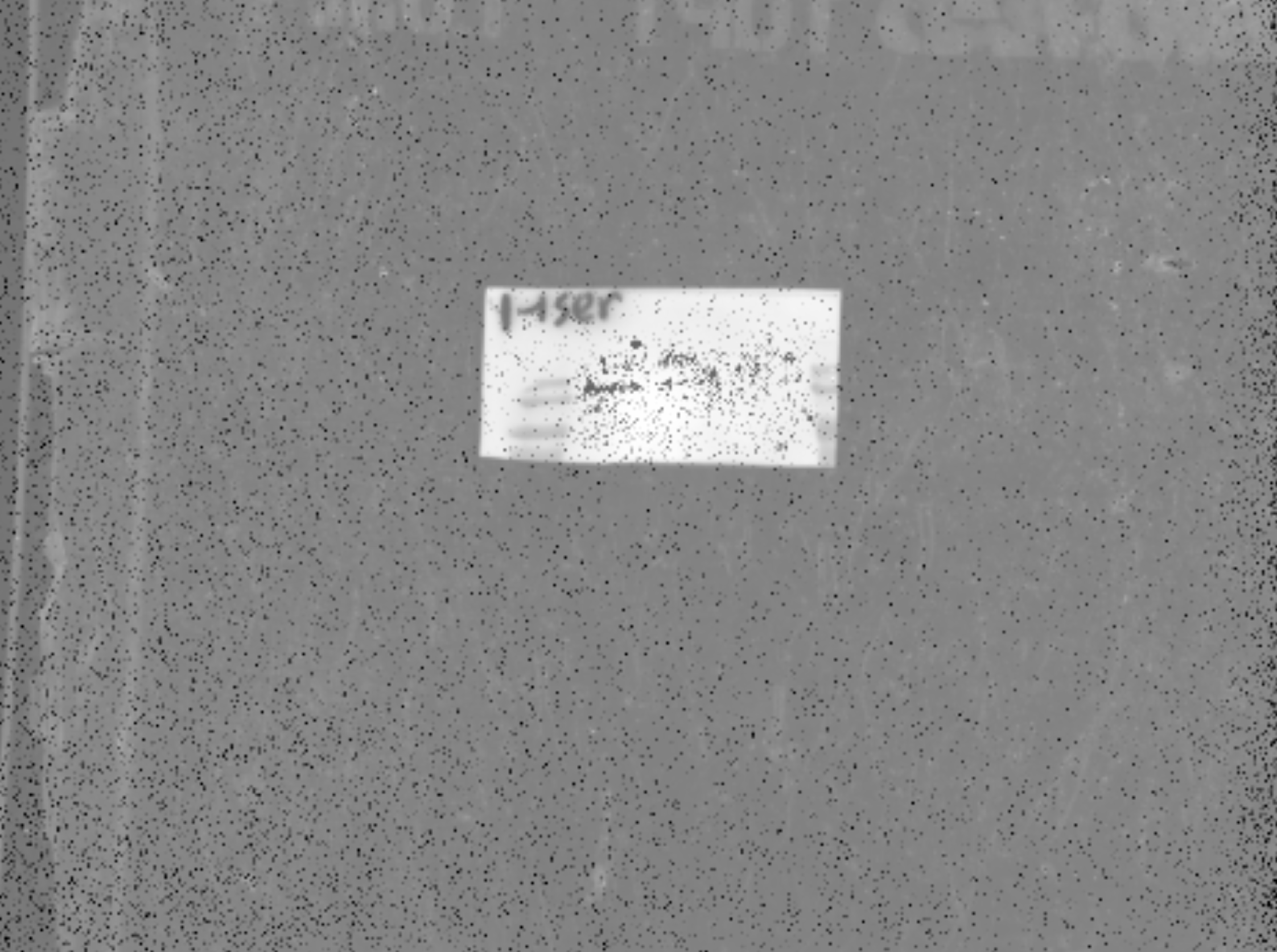

Supplement: Supplemental Information 41 [file peerj-14-21375-s041.zip › Figure 5F WB RAW OE-KLHL40 ATP2A2/ATP2A2-2 oe-KLHL40+MARK.tif]

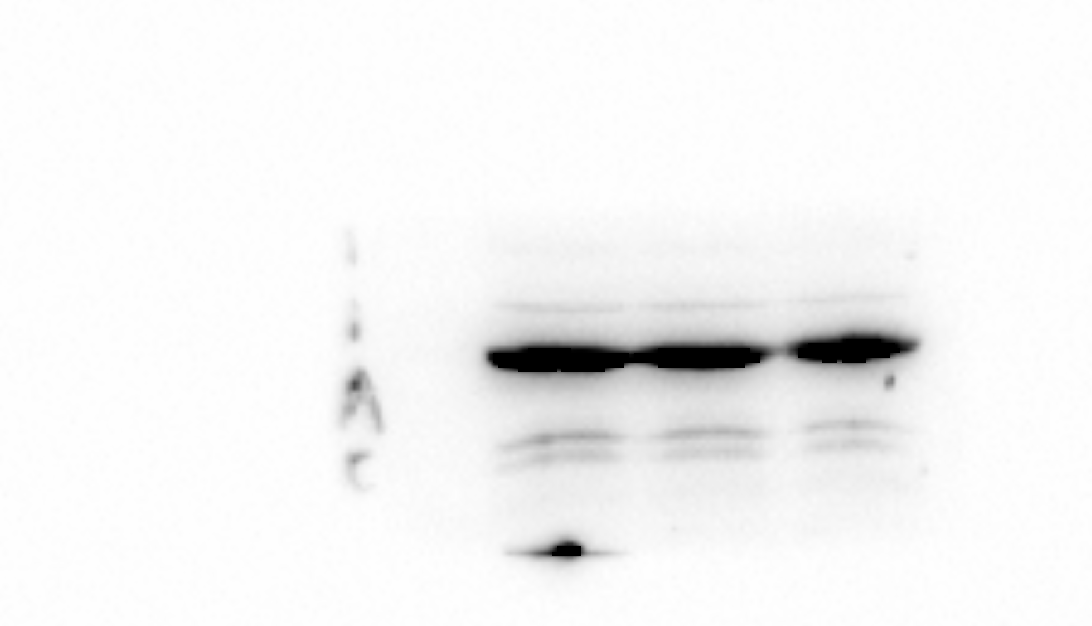

Supplement: Supplemental Information 41 [file peerj-14-21375-s041.zip › Figure 5F WB RAW OE-KLHL40 ATP2A2/ATP2A2-2 oe-KLHL40-ACTB.tif]

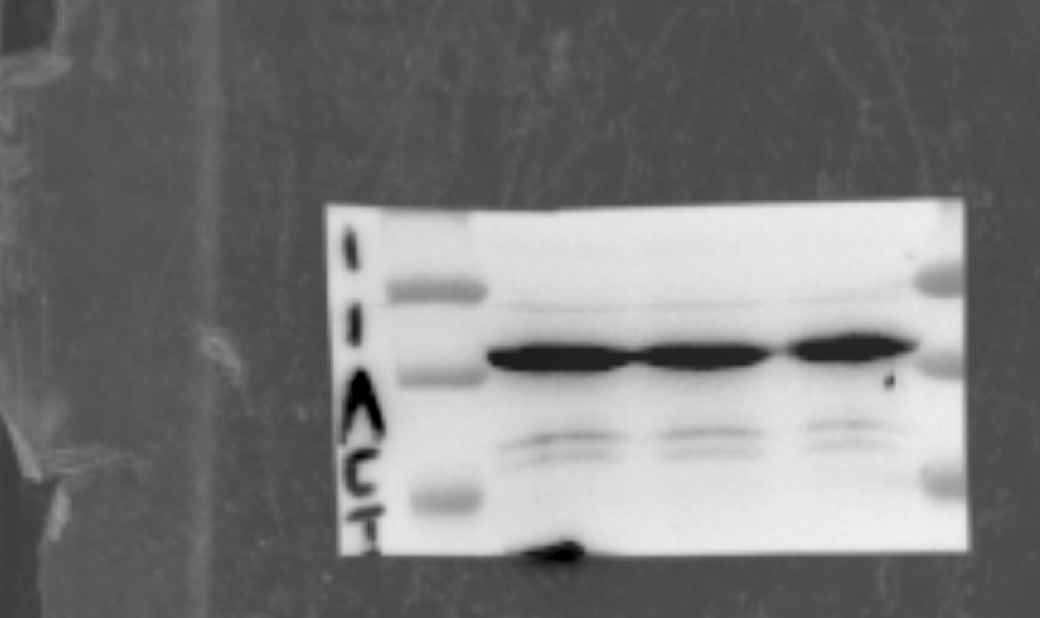

Supplement: Supplemental Information 41 [file peerj-14-21375-s041.zip › Figure 5F WB RAW OE-KLHL40 ATP2A2/ATP2A2-2 oe-KLHL40-ACTB+MARK.tif]

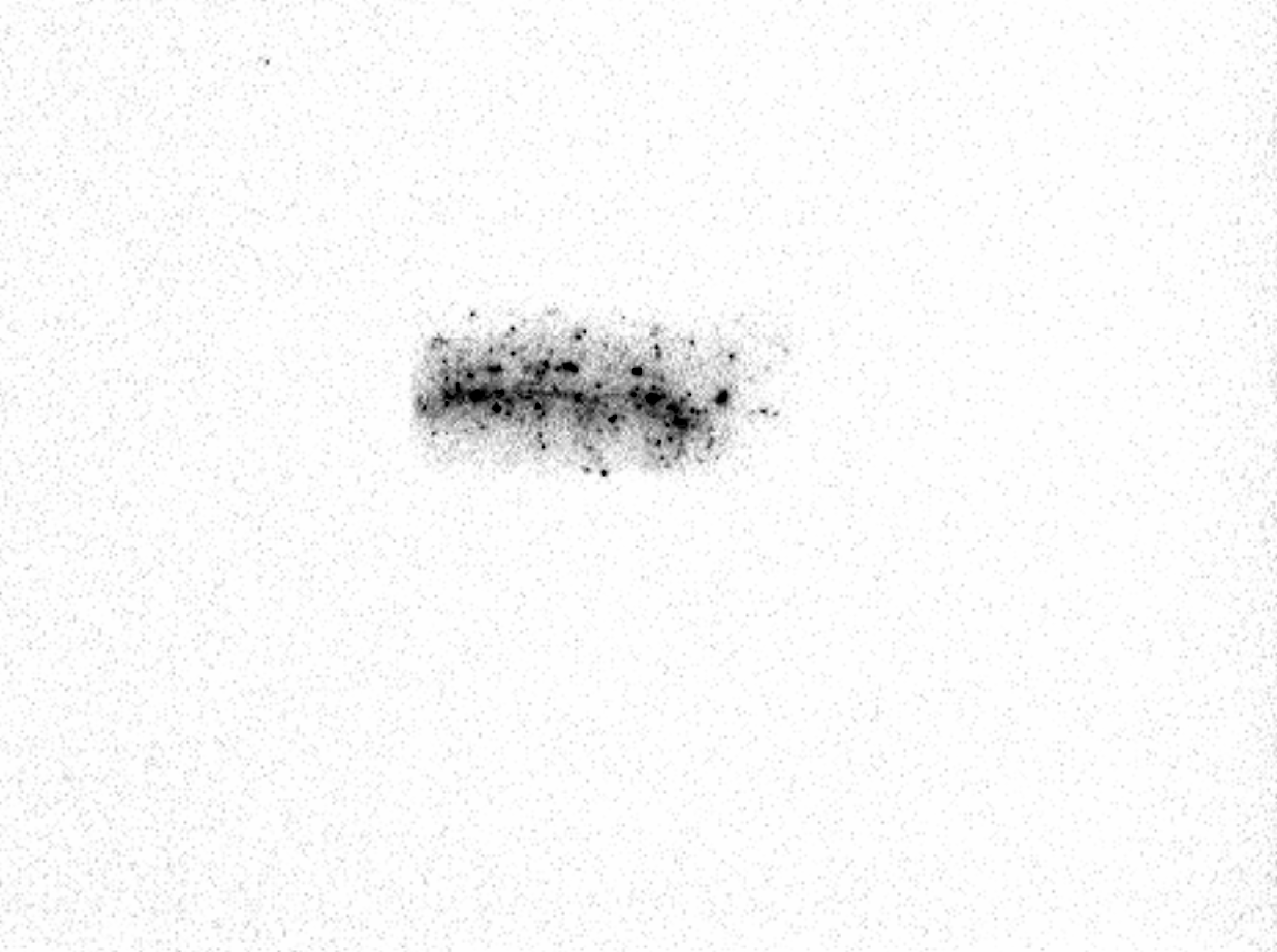

Supplement: Supplemental Information 41 [file peerj-14-21375-s041.zip › Figure 5F WB RAW OE-KLHL40 ATP2A2/ATP2A2-3 oe-KLHL40.tif]

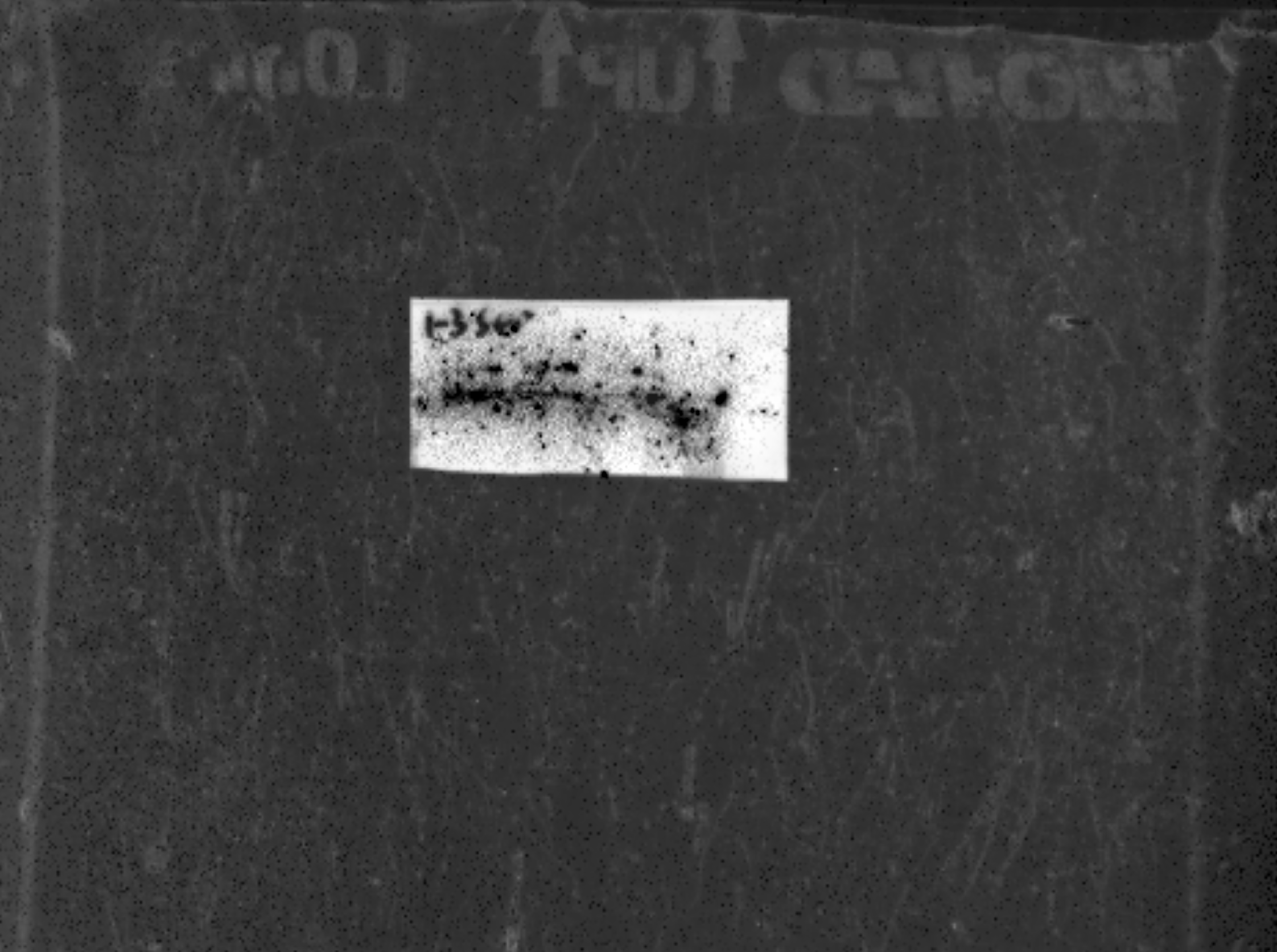

Supplement: Supplemental Information 41 [file peerj-14-21375-s041.zip › Figure 5F WB RAW OE-KLHL40 ATP2A2/ATP2A2-3 oe-KLHL40+MARK.tif]

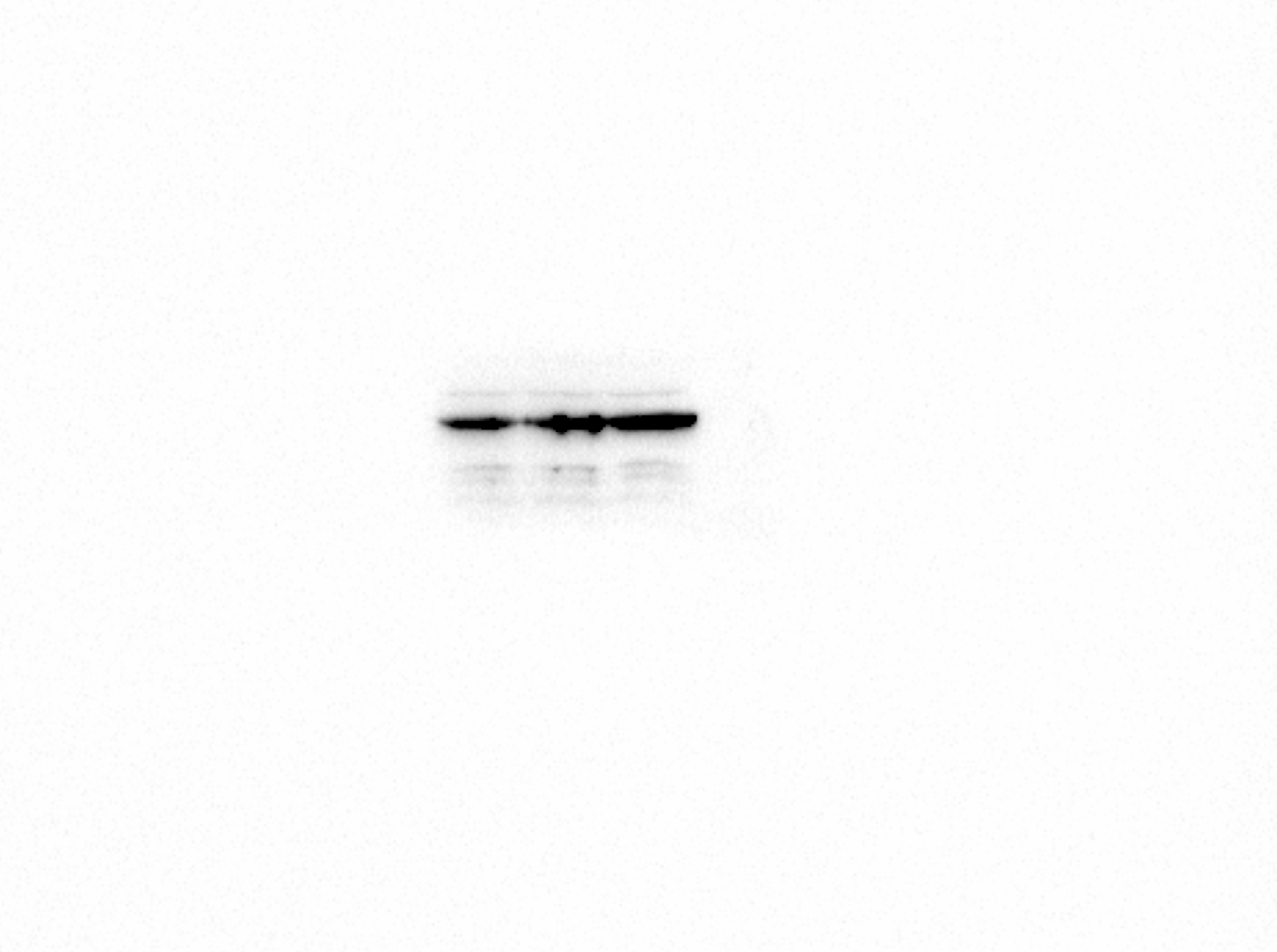

Supplement: Supplemental Information 41 [file peerj-14-21375-s041.zip › Figure 5F WB RAW OE-KLHL40 ATP2A2/ATP2A2-3 oe-KLHL40-ACTB.tif]

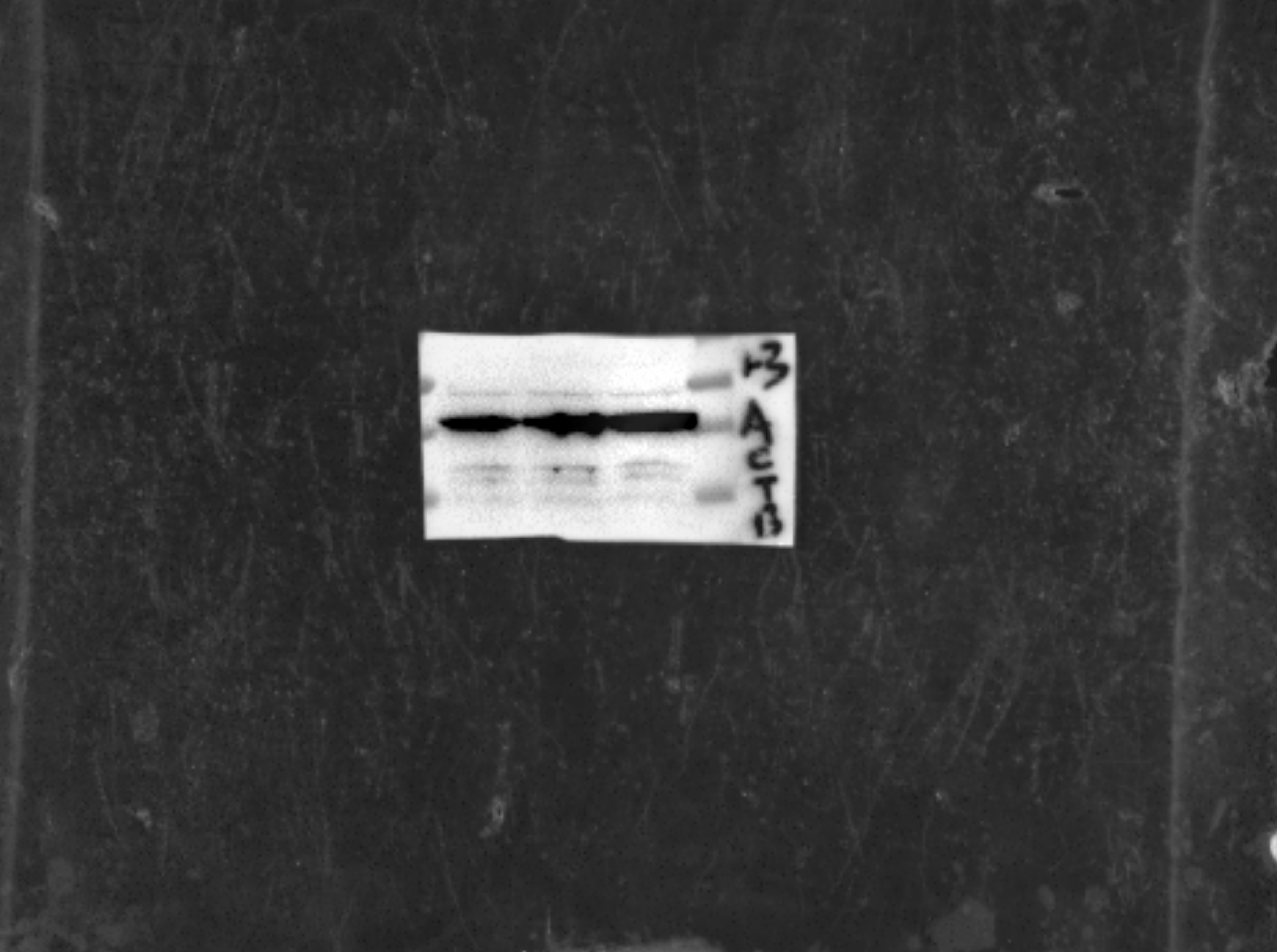

Supplement: Supplemental Information 41 [file peerj-14-21375-s041.zip › Figure 5F WB RAW OE-KLHL40 ATP2A2/ATP2A2-3 oe-KLHL40-ACTB+MARK.tif]

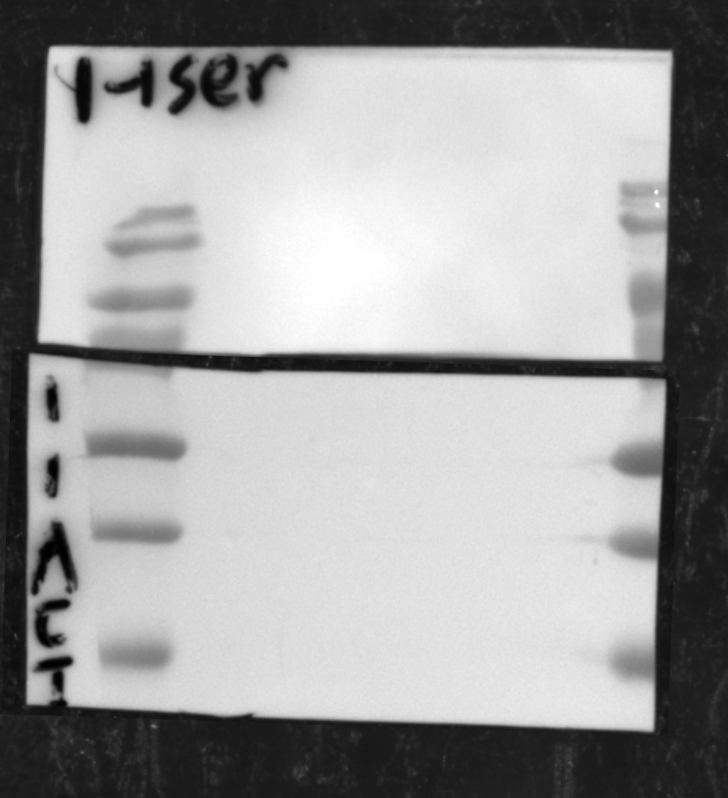

Supplement: Supplemental Information 41 [file peerj-14-21375-s041.zip › Figure 5F WB RAW OE-KLHL40 ATP2A2/TOTAL-2.tif]

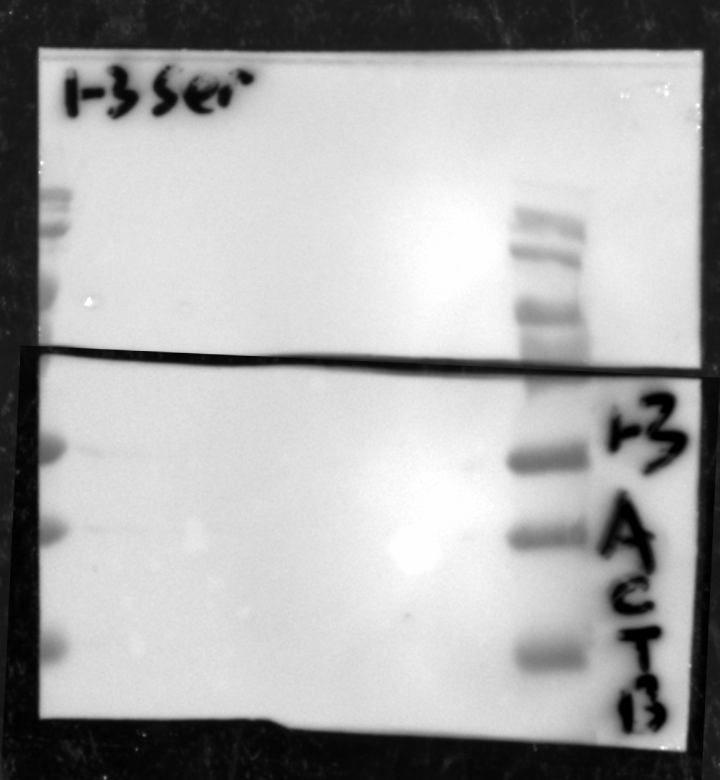

Supplement: Supplemental Information 41 [file peerj-14-21375-s041.zip › Figure 5F WB RAW OE-KLHL40 ATP2A2/TOTAL-3.tif]

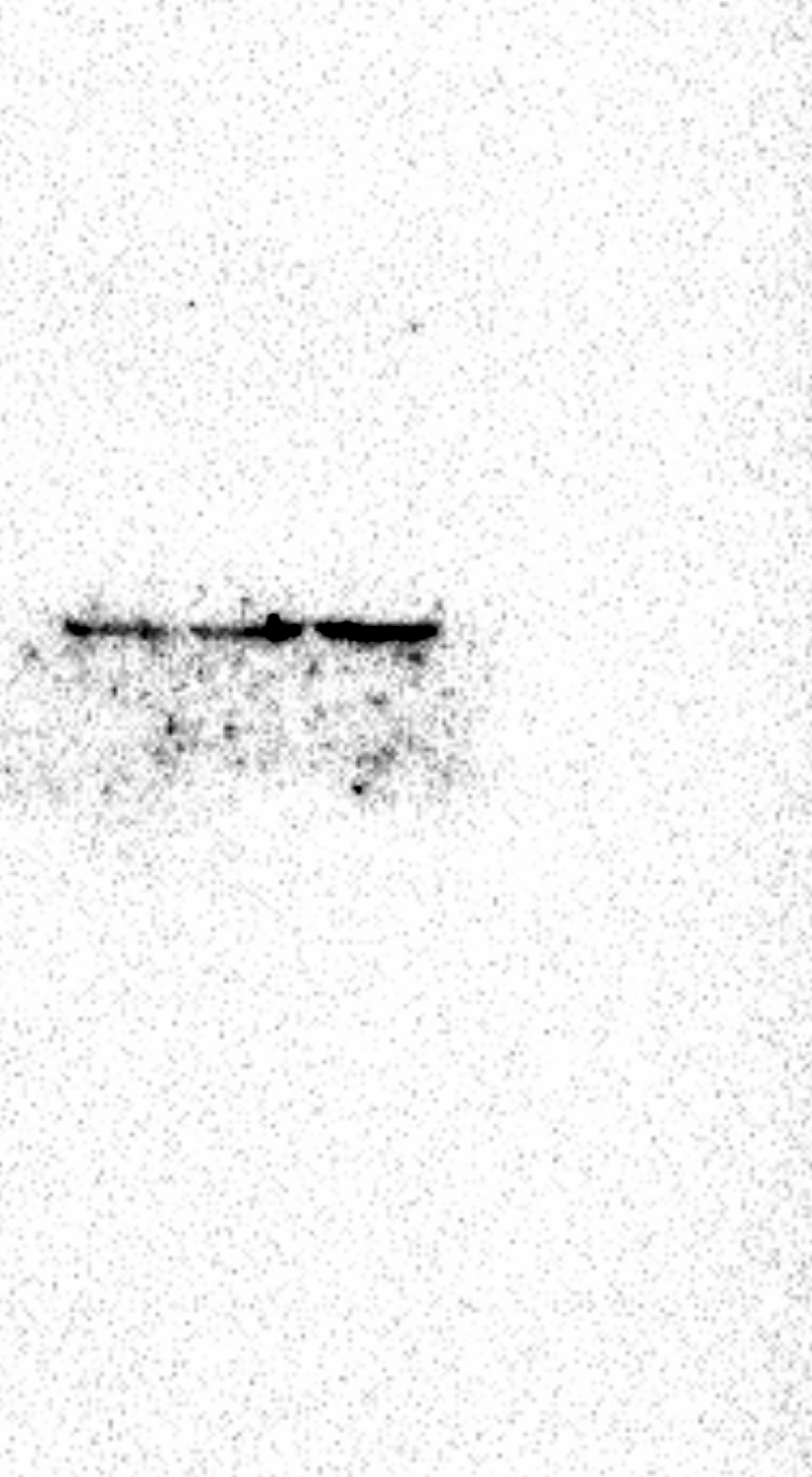

Supplement: Supplemental Information 42 [file peerj-14-21375-s042.zip › Figure 5H WB RAW SH-KLHL40 DES/DES-1 sh-KLHL40.tif]

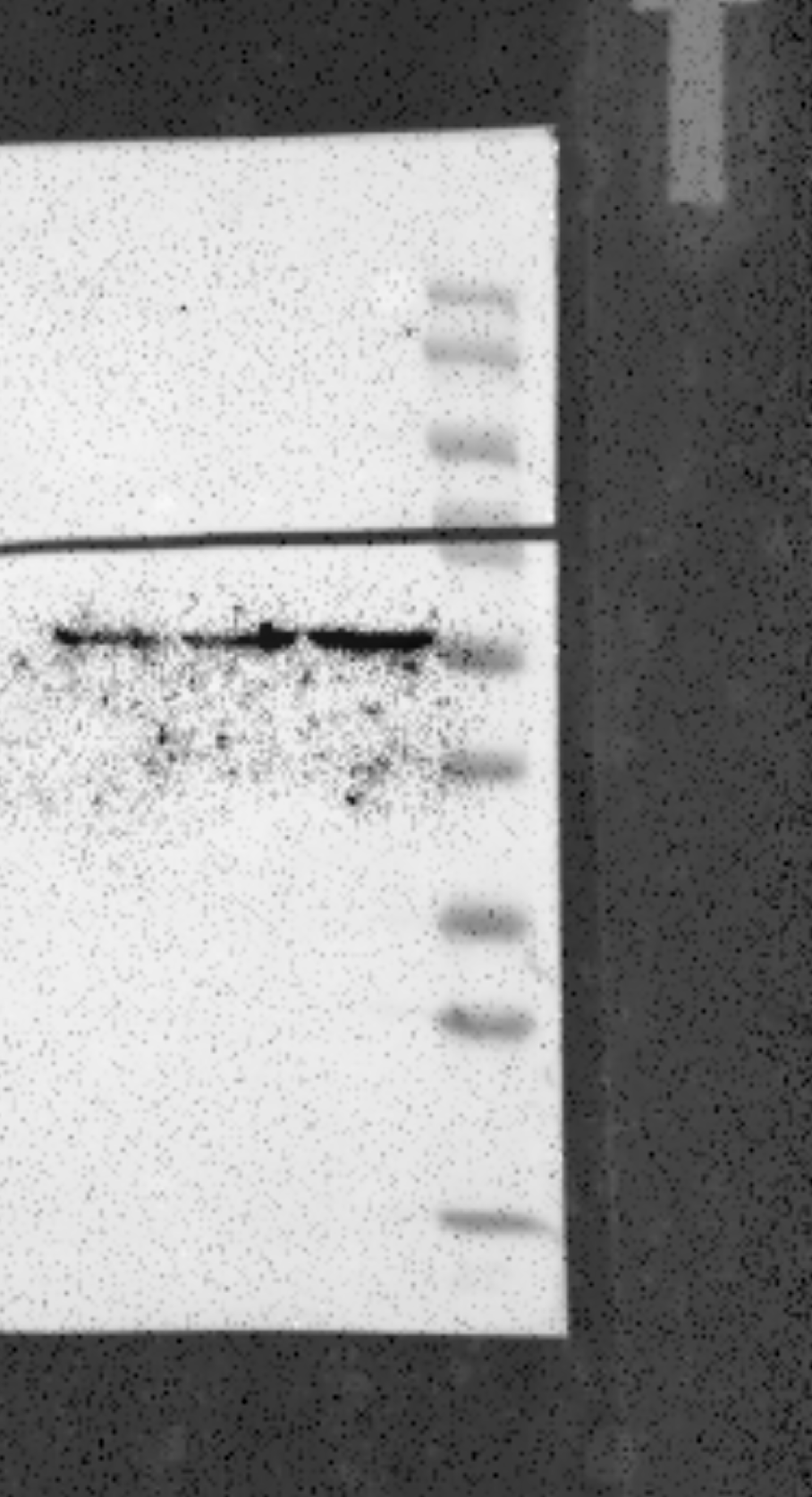

Supplement: Supplemental Information 42 [file peerj-14-21375-s042.zip › Figure 5H WB RAW SH-KLHL40 DES/DES-1 sh-KLHL40+MARK.tif]

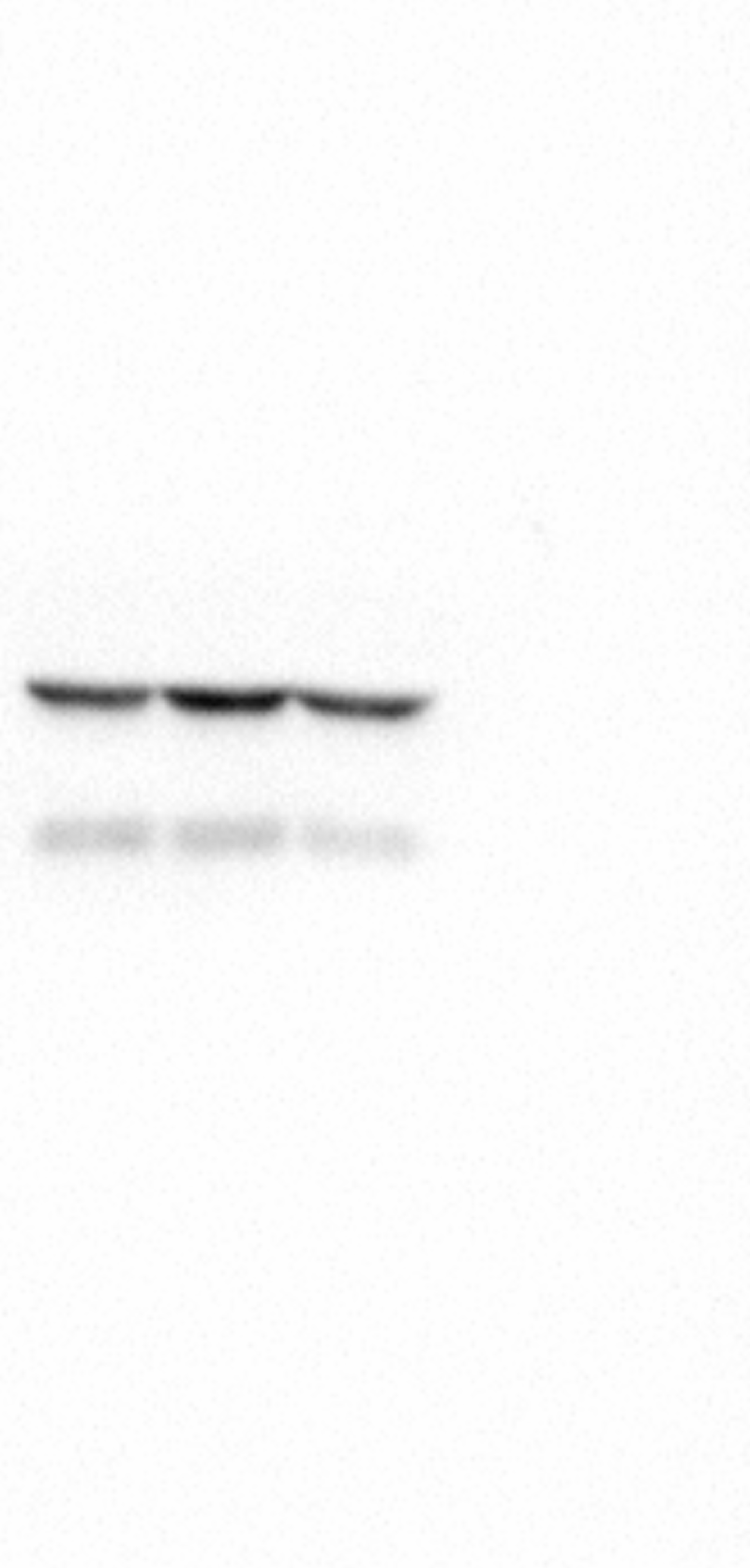

Supplement: Supplemental Information 42 [file peerj-14-21375-s042.zip › Figure 5H WB RAW SH-KLHL40 DES/DES-1 sh-KLHL40-ACTB.tif]

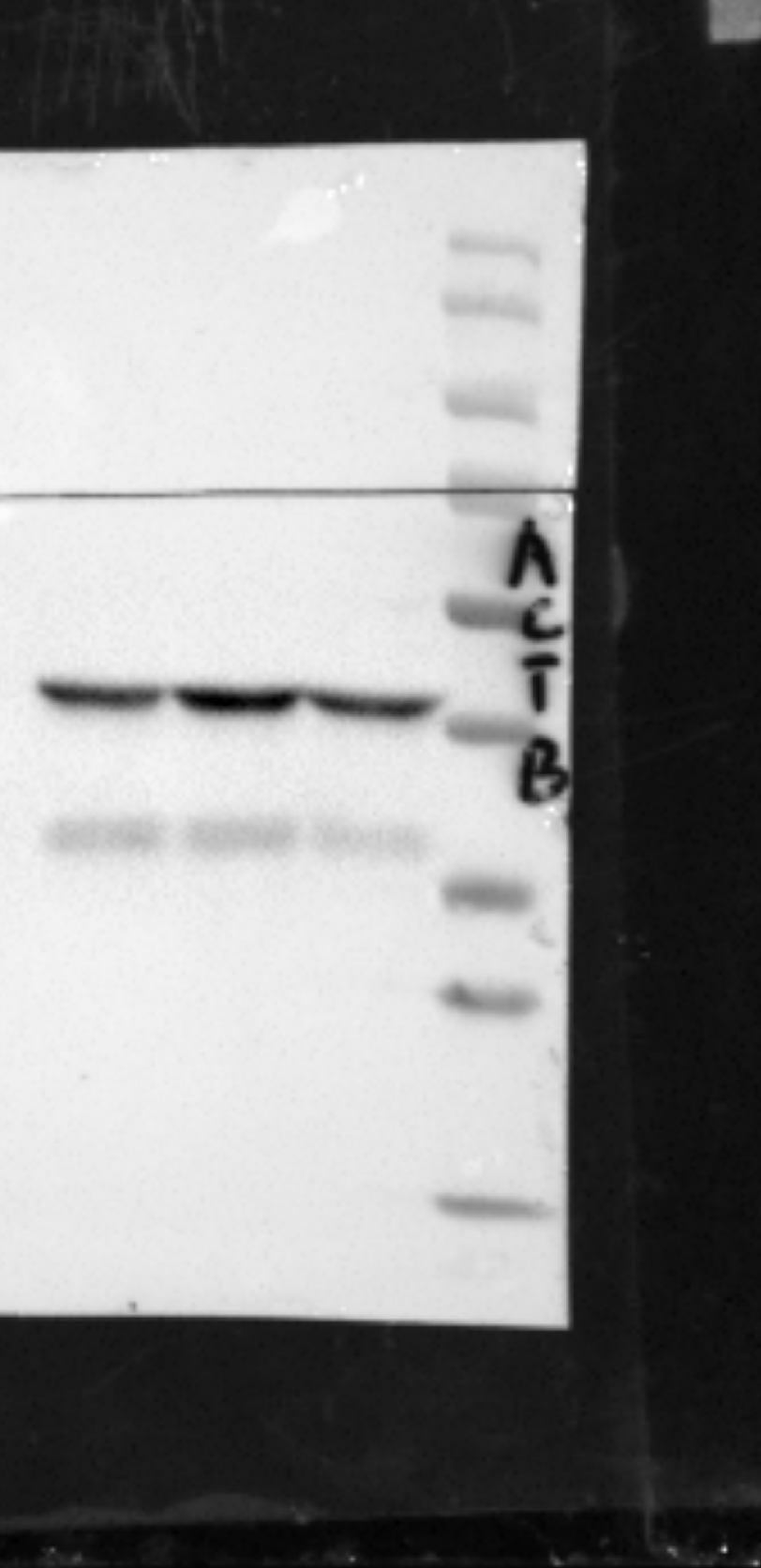

Supplement: Supplemental Information 42 [file peerj-14-21375-s042.zip › Figure 5H WB RAW SH-KLHL40 DES/DES-1 sh-KLHL40-ACTB+MARK.tif]

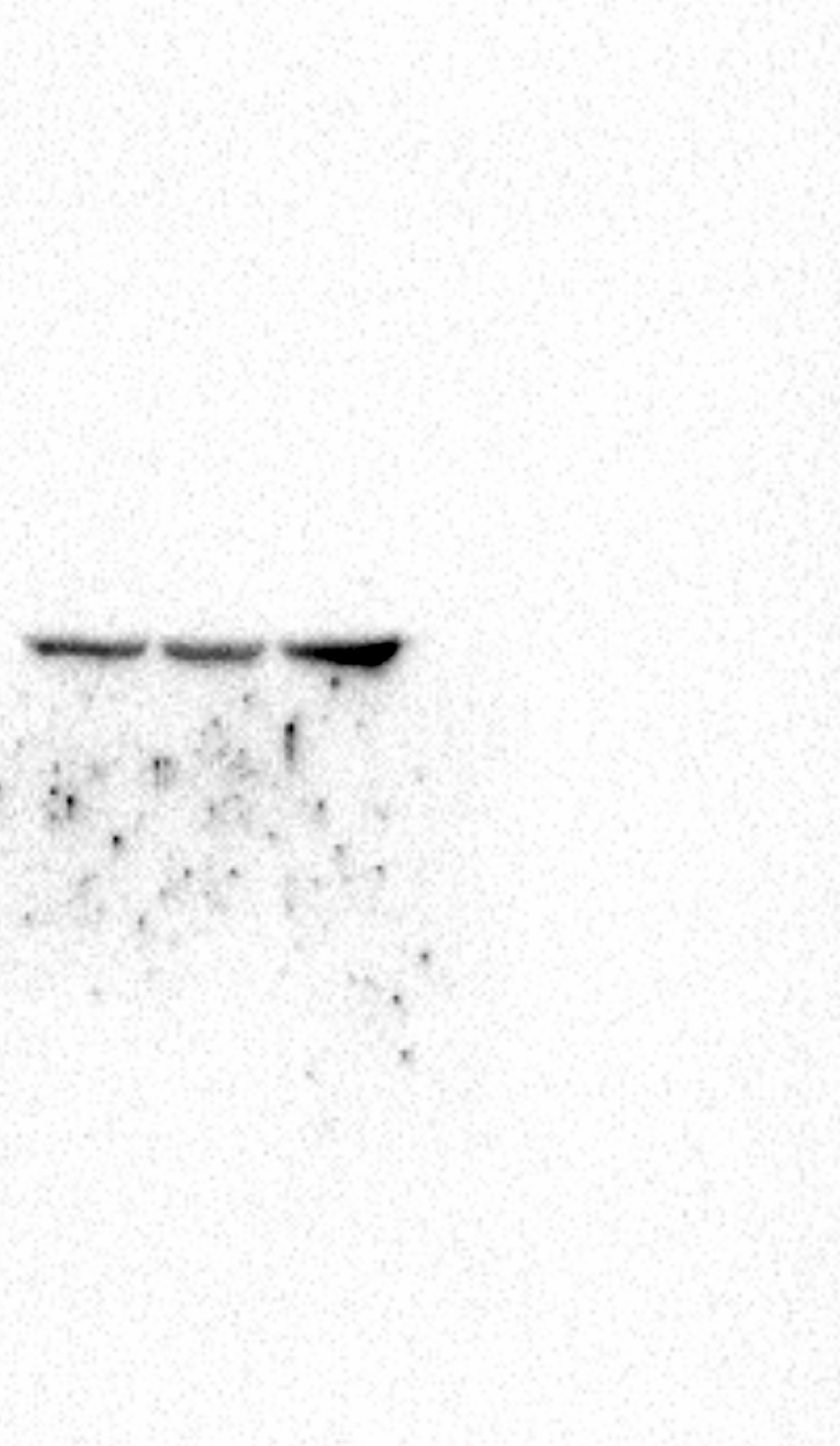

Supplement: Supplemental Information 42 [file peerj-14-21375-s042.zip › Figure 5H WB RAW SH-KLHL40 DES/DES-2 sh-KLHL40.tif]

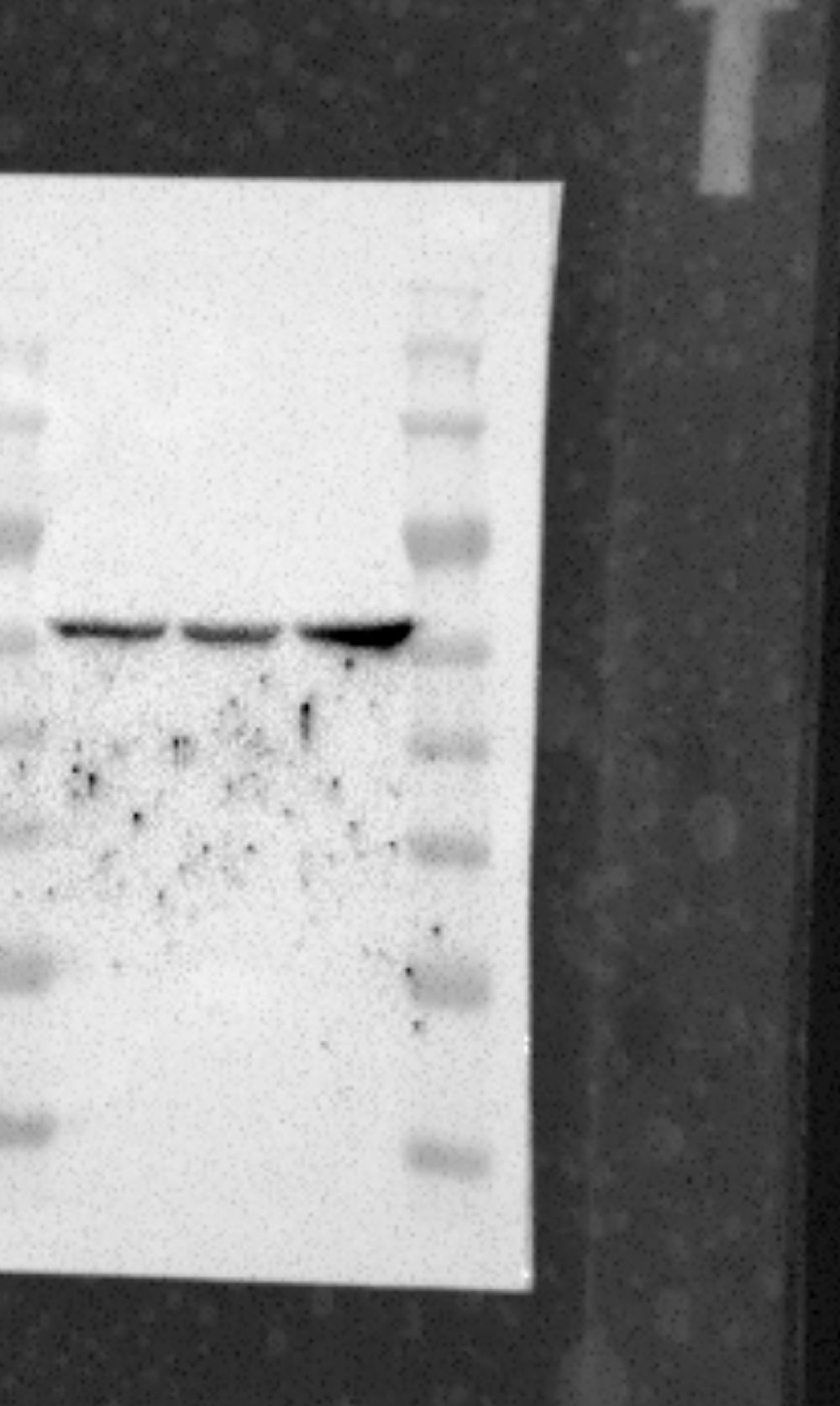

Supplement: Supplemental Information 42 [file peerj-14-21375-s042.zip › Figure 5H WB RAW SH-KLHL40 DES/DES-2 sh-KLHL40+MARK.tif]

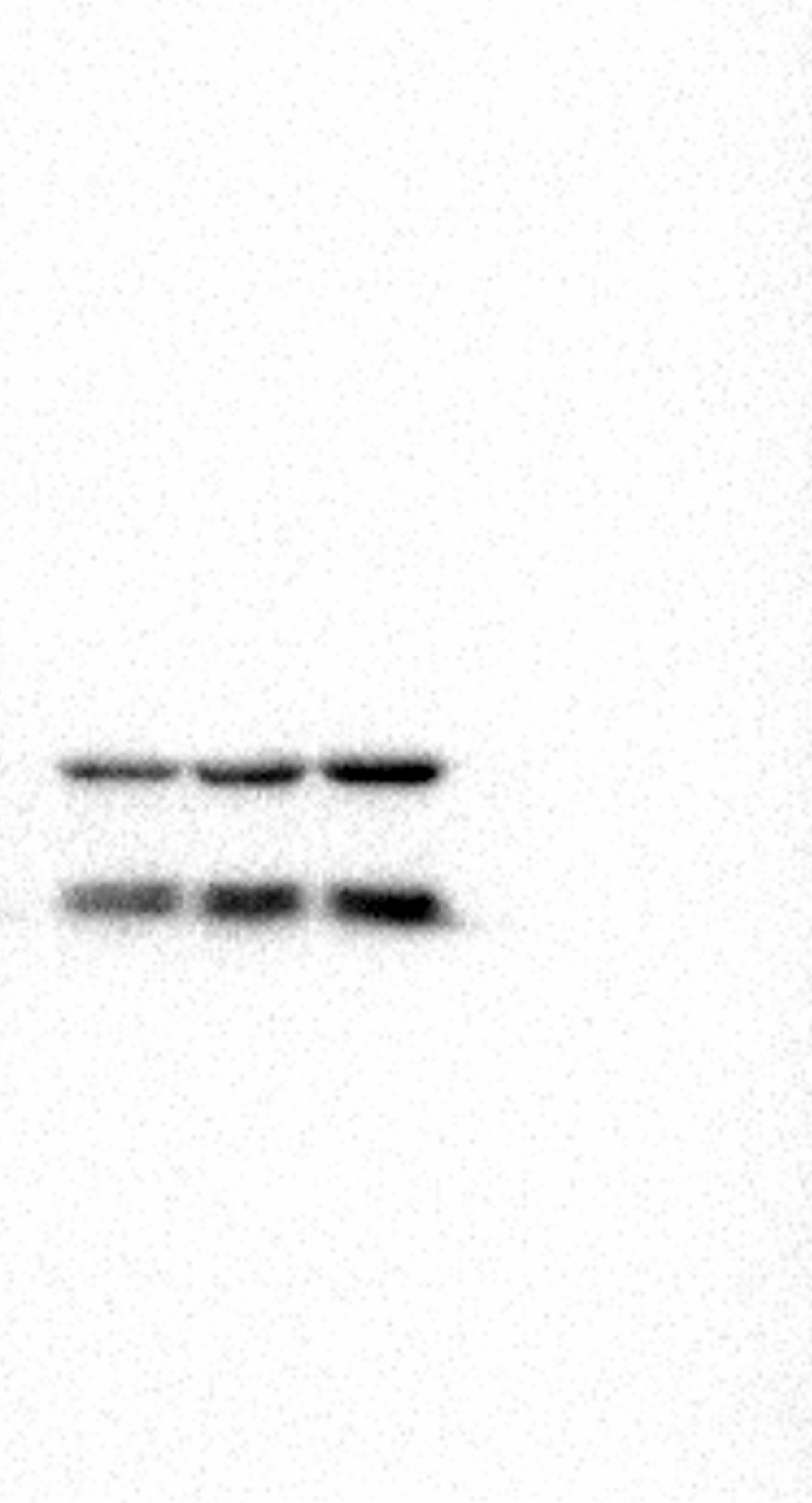

Supplement: Supplemental Information 42 [file peerj-14-21375-s042.zip › Figure 5H WB RAW SH-KLHL40 DES/DES-2 sh-KLHL40-ACTB.tif]

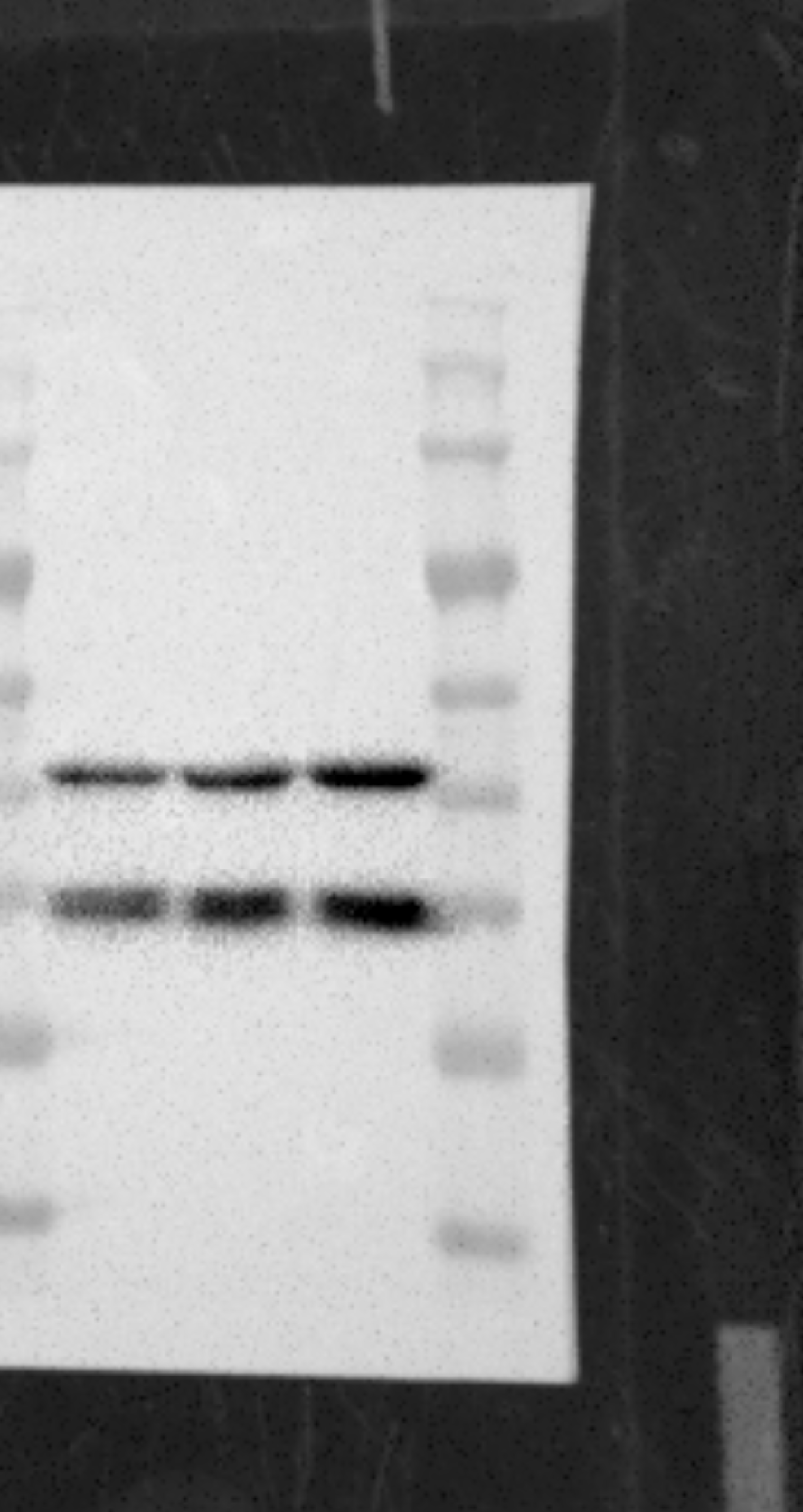

Supplement: Supplemental Information 42 [file peerj-14-21375-s042.zip › Figure 5H WB RAW SH-KLHL40 DES/DES-2 sh-KLHL40-ACTB+MARK.tif]

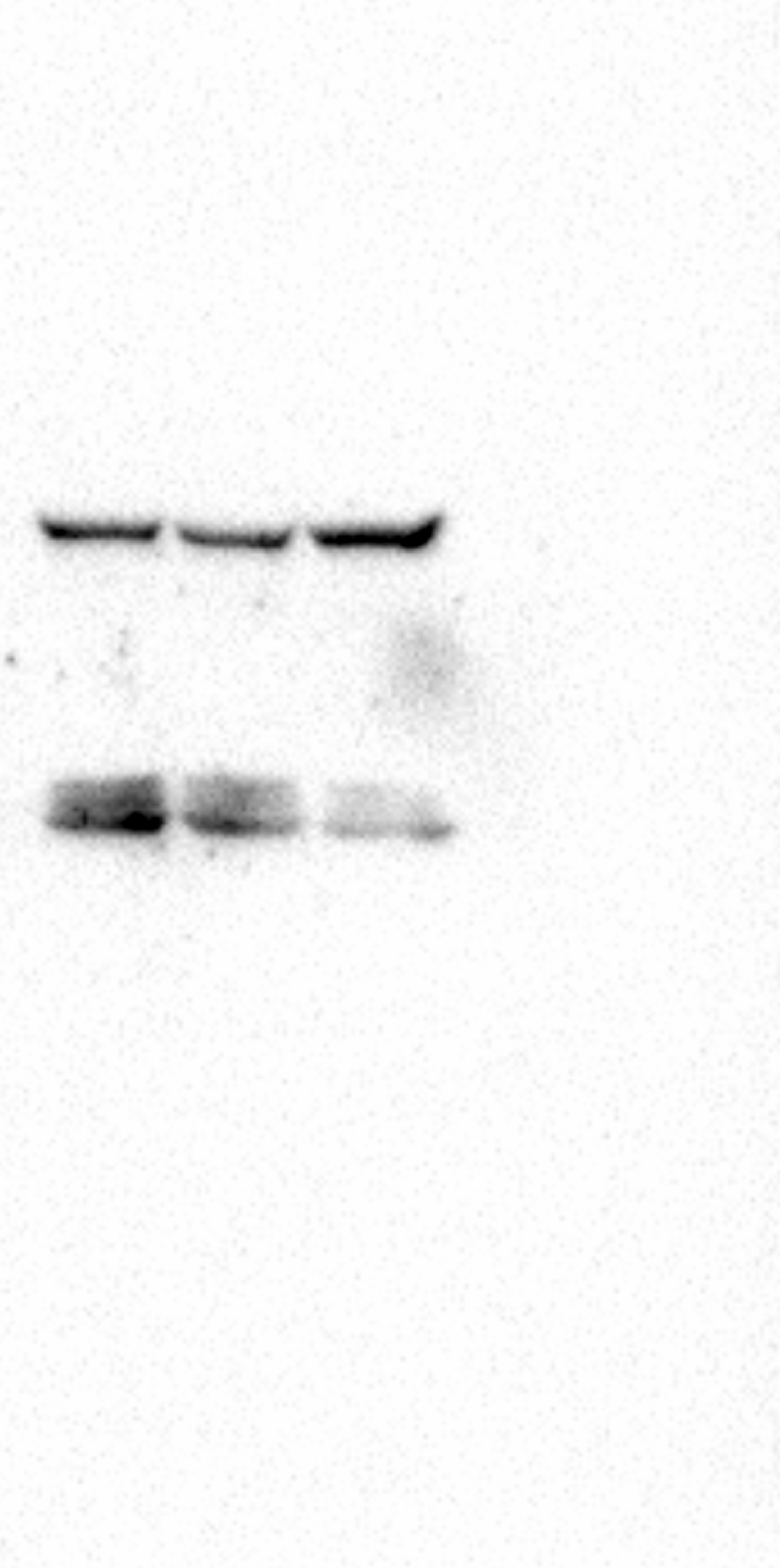

Supplement: Supplemental Information 42 [file peerj-14-21375-s042.zip › Figure 5H WB RAW SH-KLHL40 DES/DES-3 sh-KLHL40.tif]

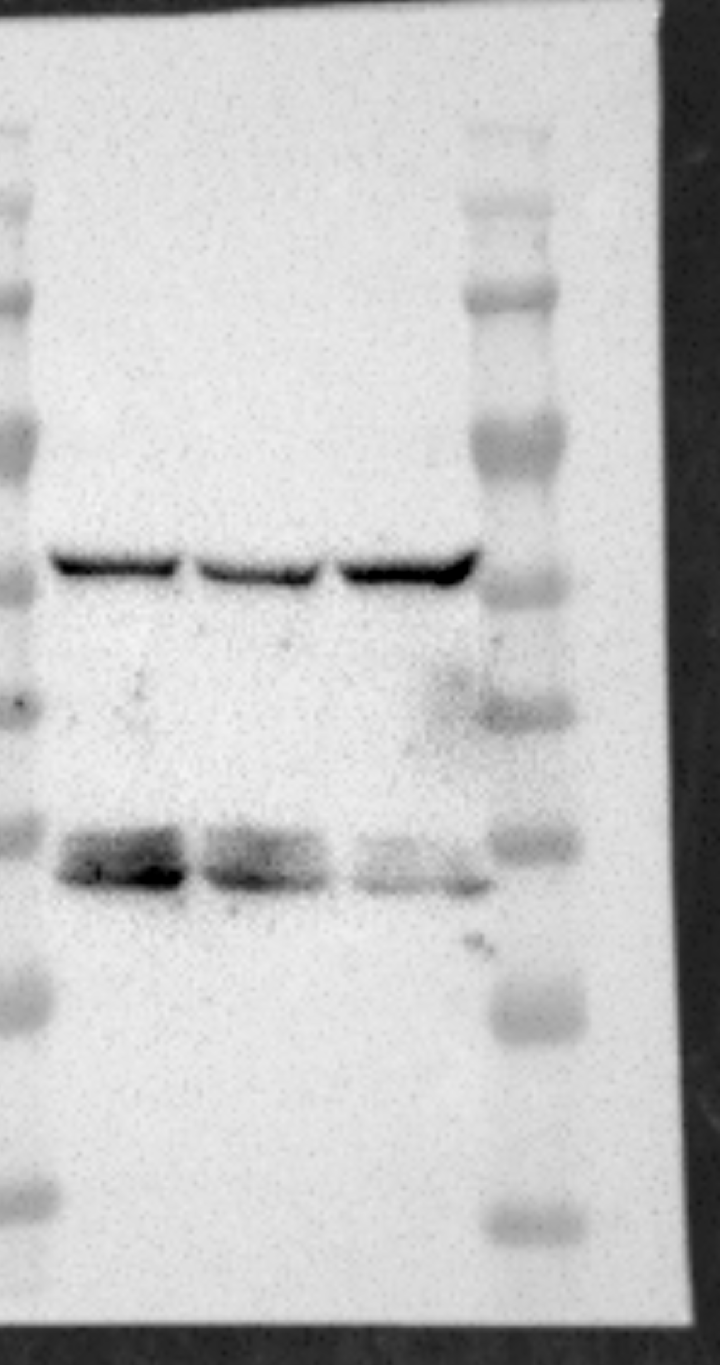

Supplement: Supplemental Information 42 [file peerj-14-21375-s042.zip › Figure 5H WB RAW SH-KLHL40 DES/DES-3 sh-KLHL40+MARK.tif]

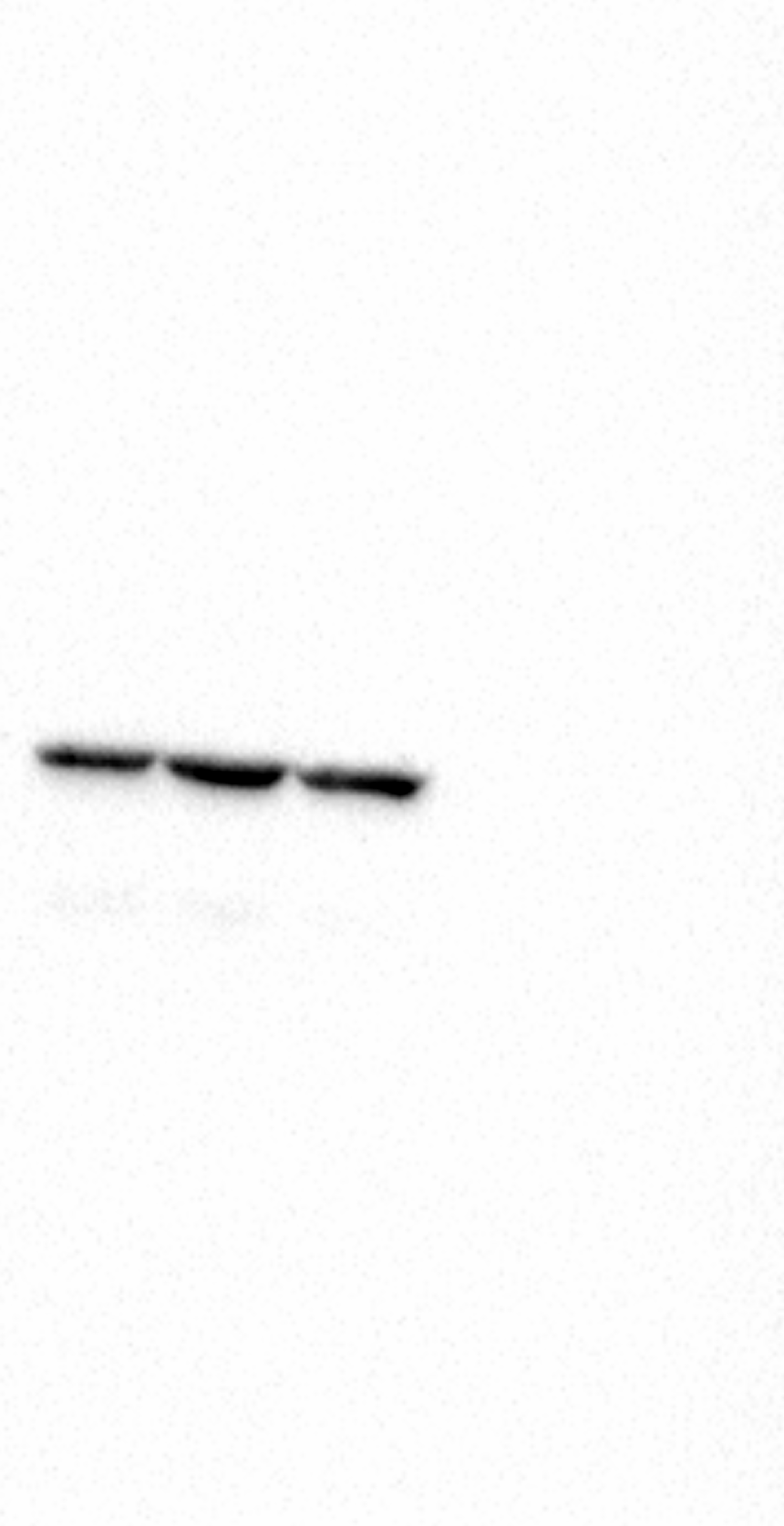

Supplement: Supplemental Information 42 [file peerj-14-21375-s042.zip › Figure 5H WB RAW SH-KLHL40 DES/DES-3 sh-KLHL40-ACTB.tif]

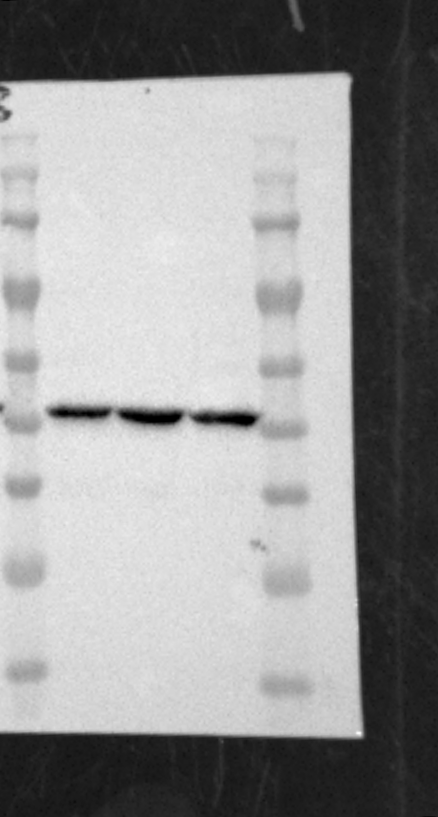

Supplement: Supplemental Information 42 [file peerj-14-21375-s042.zip › Figure 5H WB RAW SH-KLHL40 DES/DES-3 sh-KLHL40-ACTB+MARK.tif]

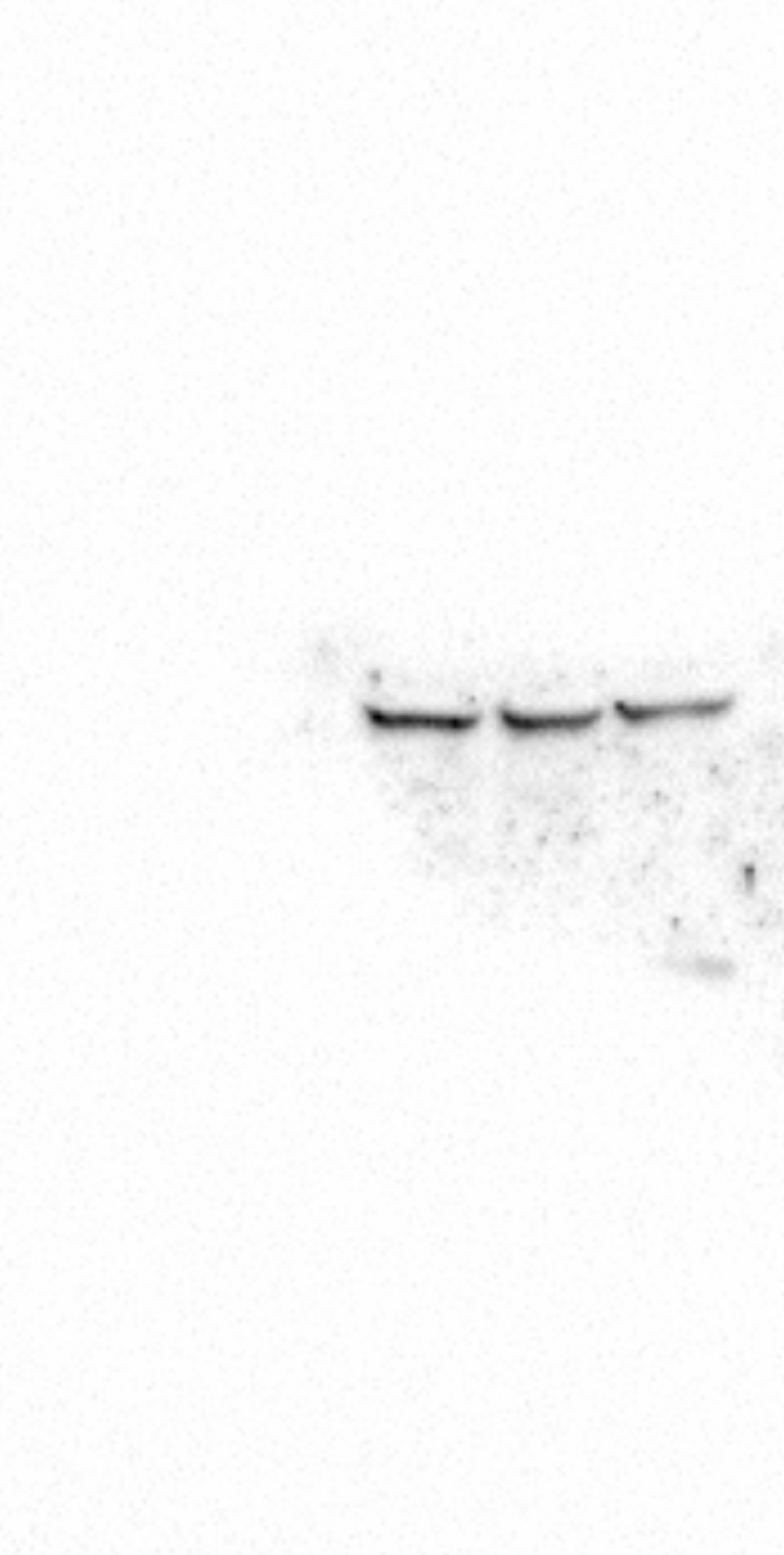

Supplement: Supplemental Information 43 [file peerj-14-21375-s043.zip › Figure 5I WB RAW OE-KLHL40 DES/DES-1 oe-KLHL40.tif]

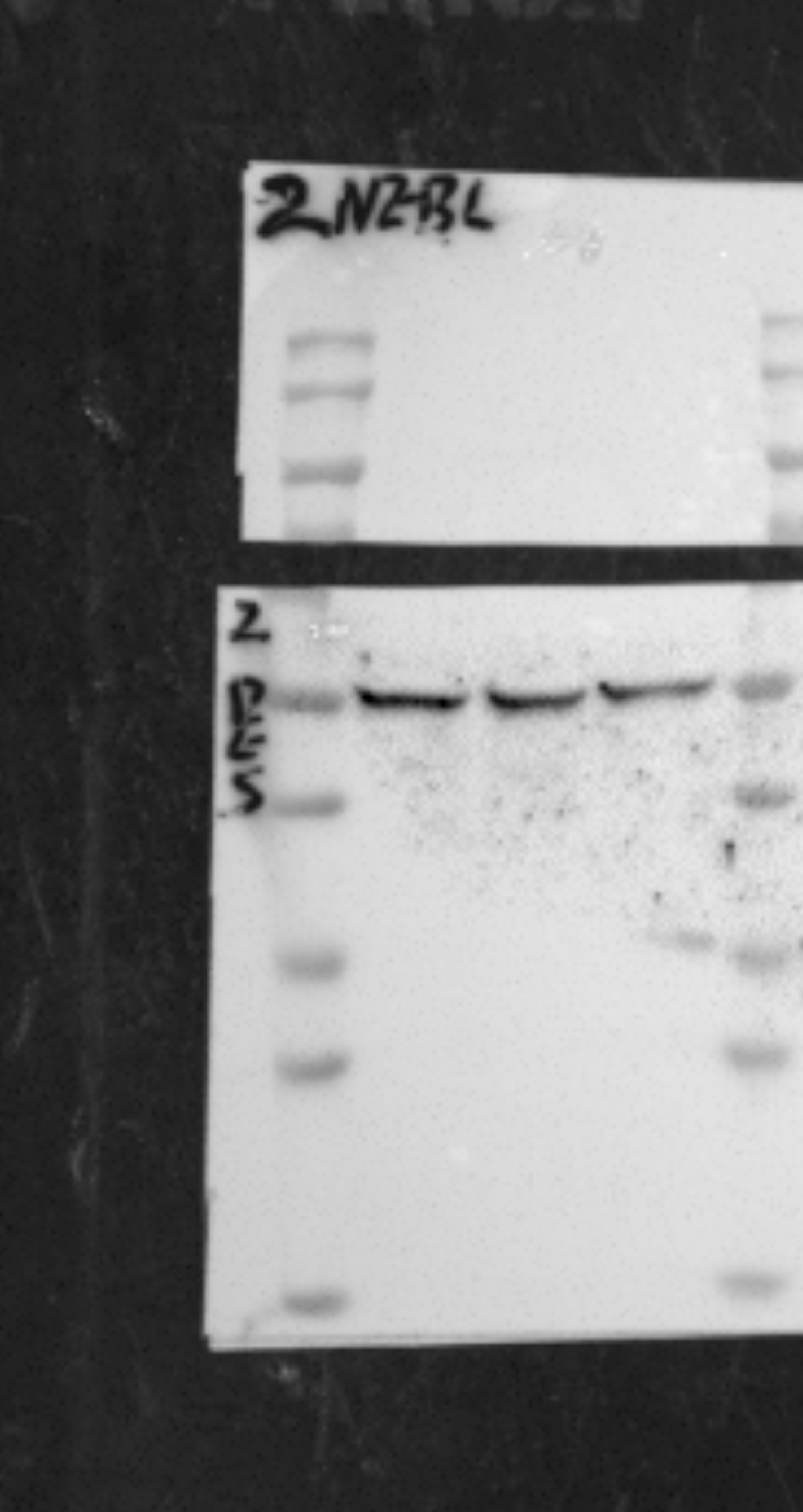

Supplement: Supplemental Information 43 [file peerj-14-21375-s043.zip › Figure 5I WB RAW OE-KLHL40 DES/DES-1 oe-KLHL40+MARK.tif]

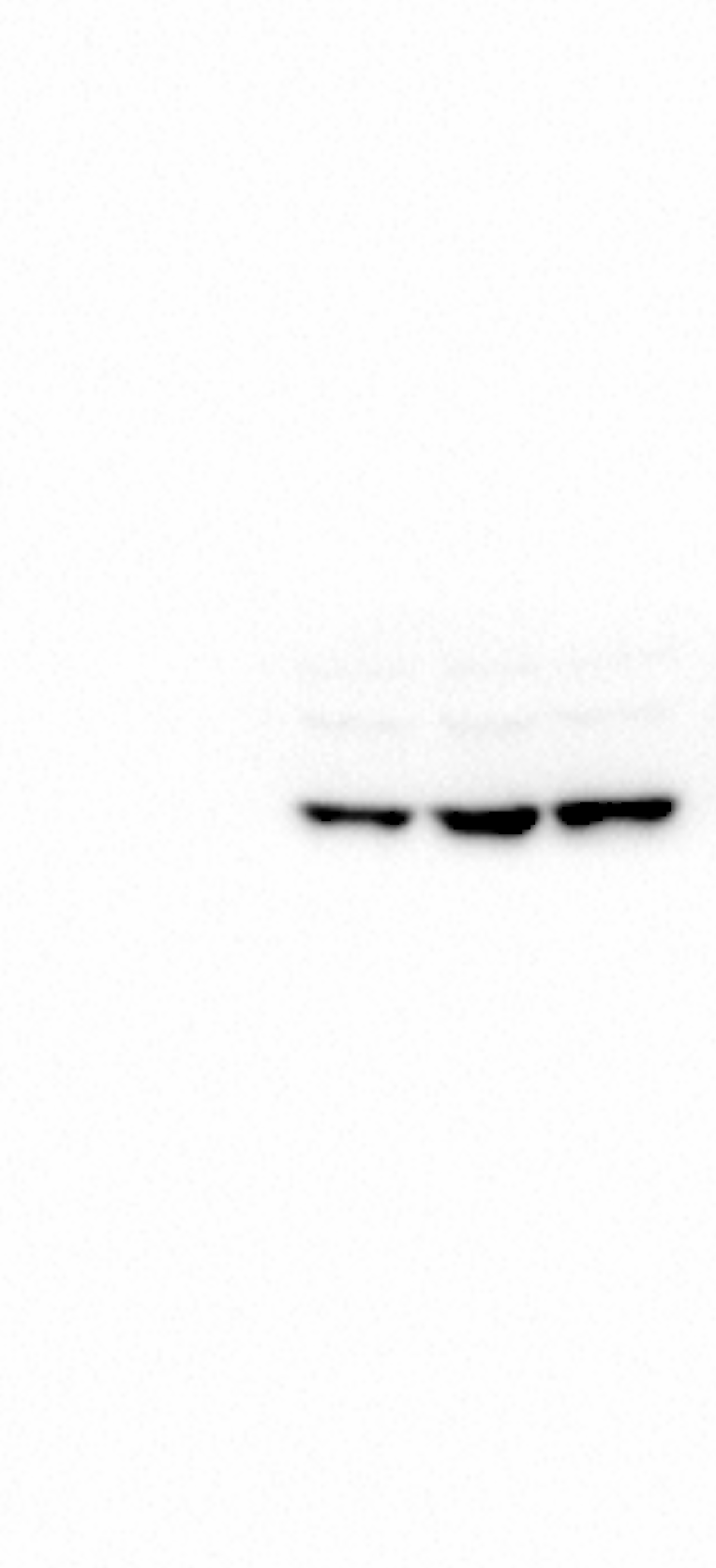

Supplement: Supplemental Information 43 [file peerj-14-21375-s043.zip › Figure 5I WB RAW OE-KLHL40 DES/DES-1 oe-KLHL40-ACTB.tif]

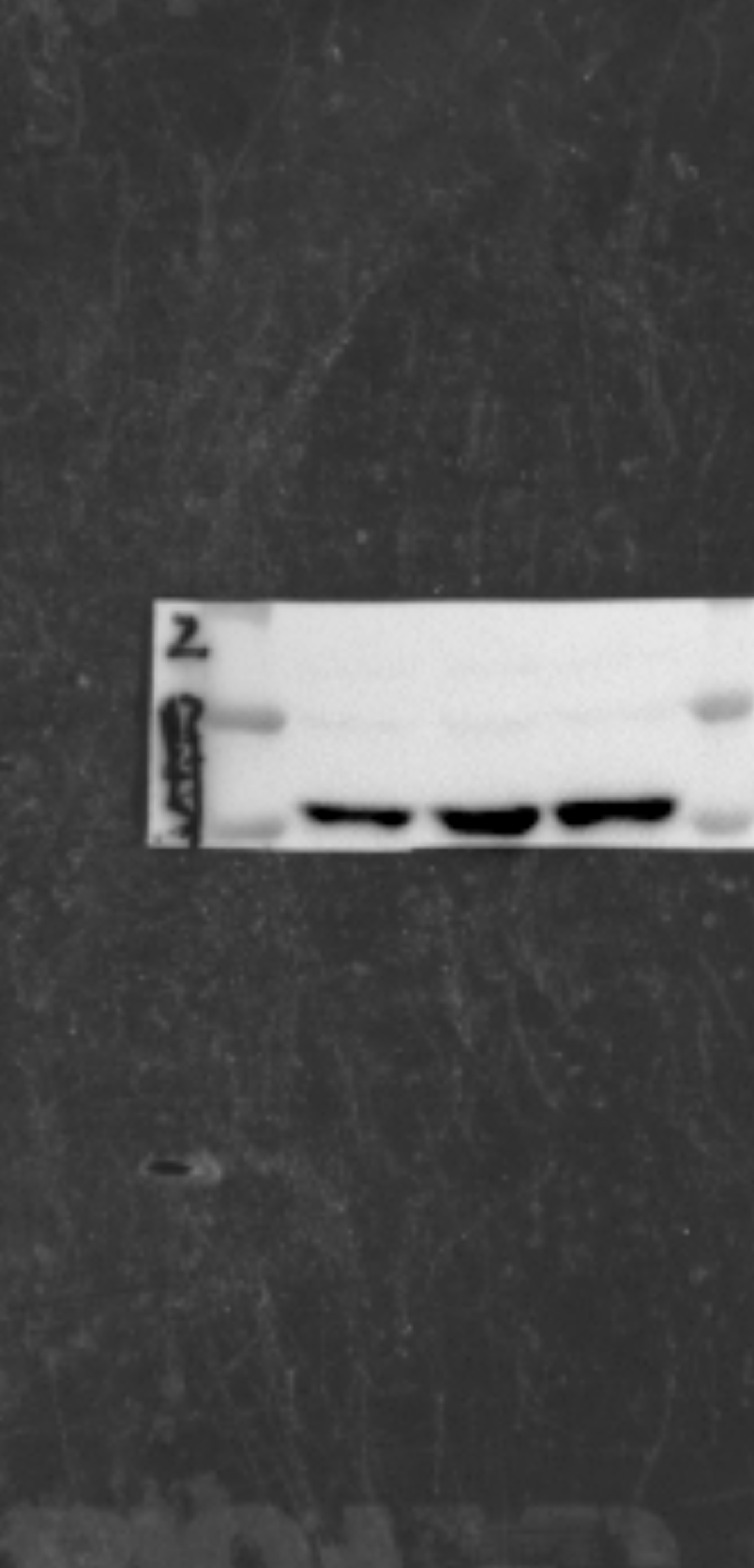

Supplement: Supplemental Information 43 [file peerj-14-21375-s043.zip › Figure 5I WB RAW OE-KLHL40 DES/DES-1 oe-KLHL40-ACTB+MARK.tif]

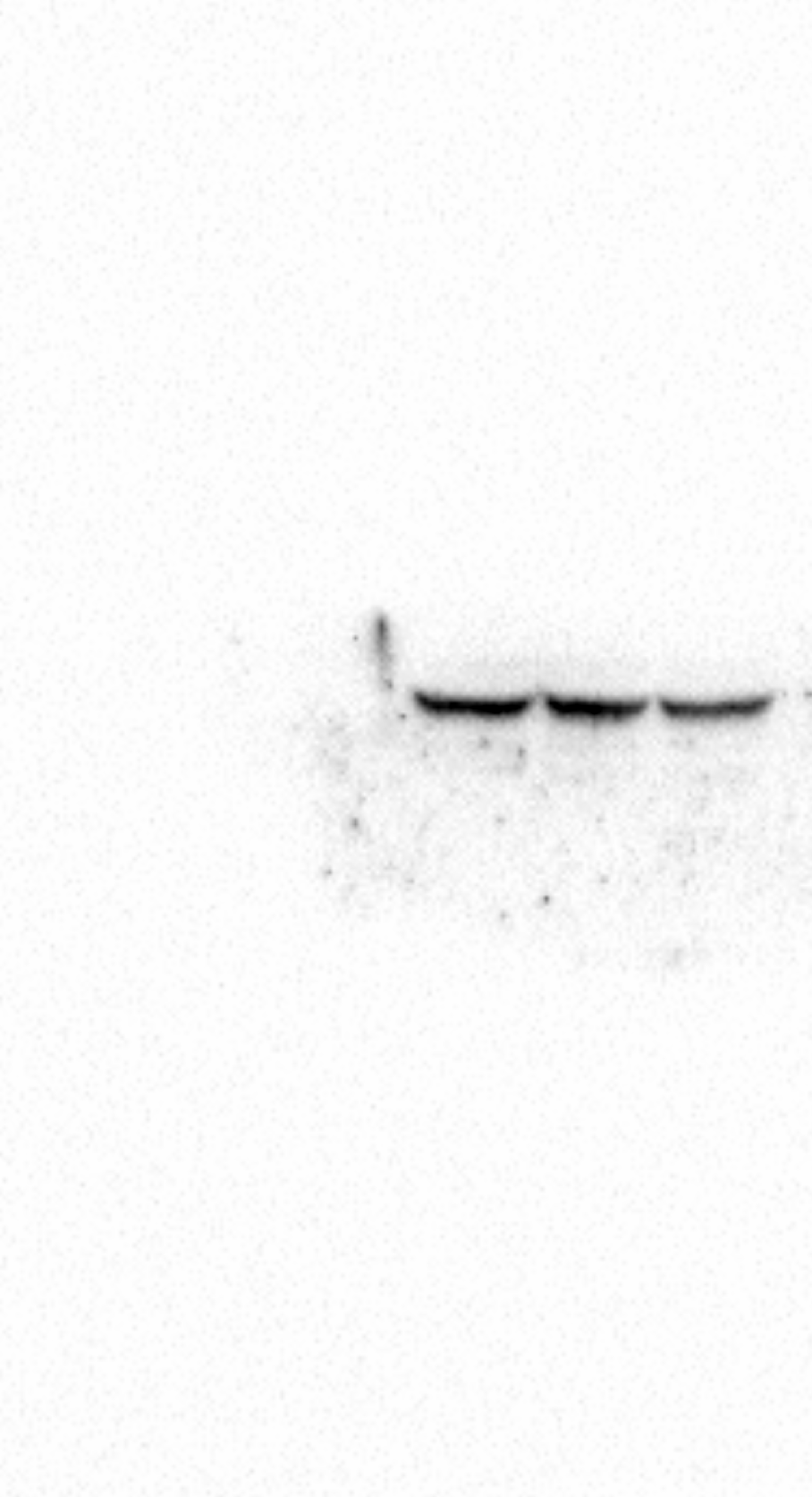

Supplement: Supplemental Information 43 [file peerj-14-21375-s043.zip › Figure 5I WB RAW OE-KLHL40 DES/DES-2 oe-KLHL40.tif]

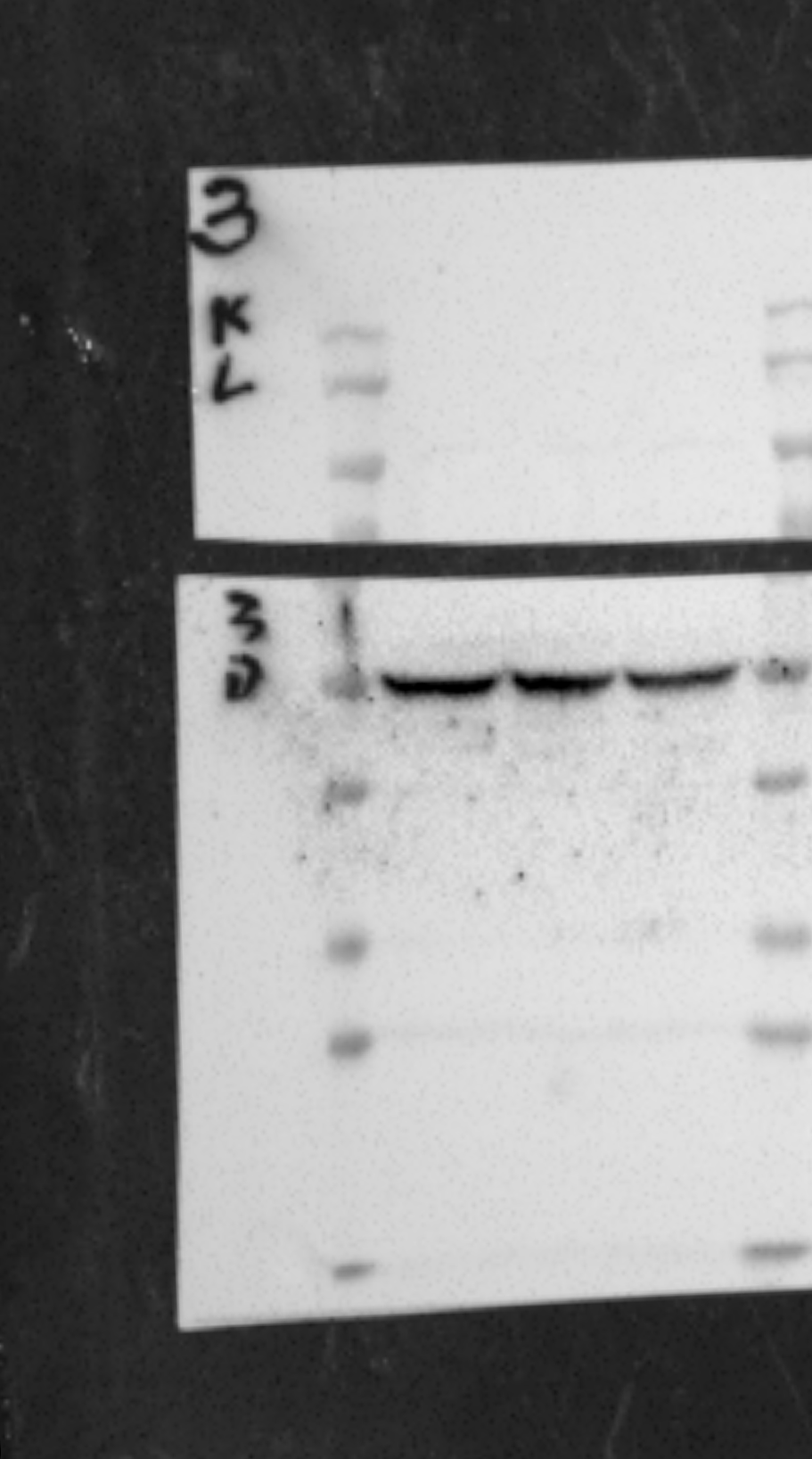

Supplement: Supplemental Information 43 [file peerj-14-21375-s043.zip › Figure 5I WB RAW OE-KLHL40 DES/DES-2 oe-KLHL40+MARK.tif]

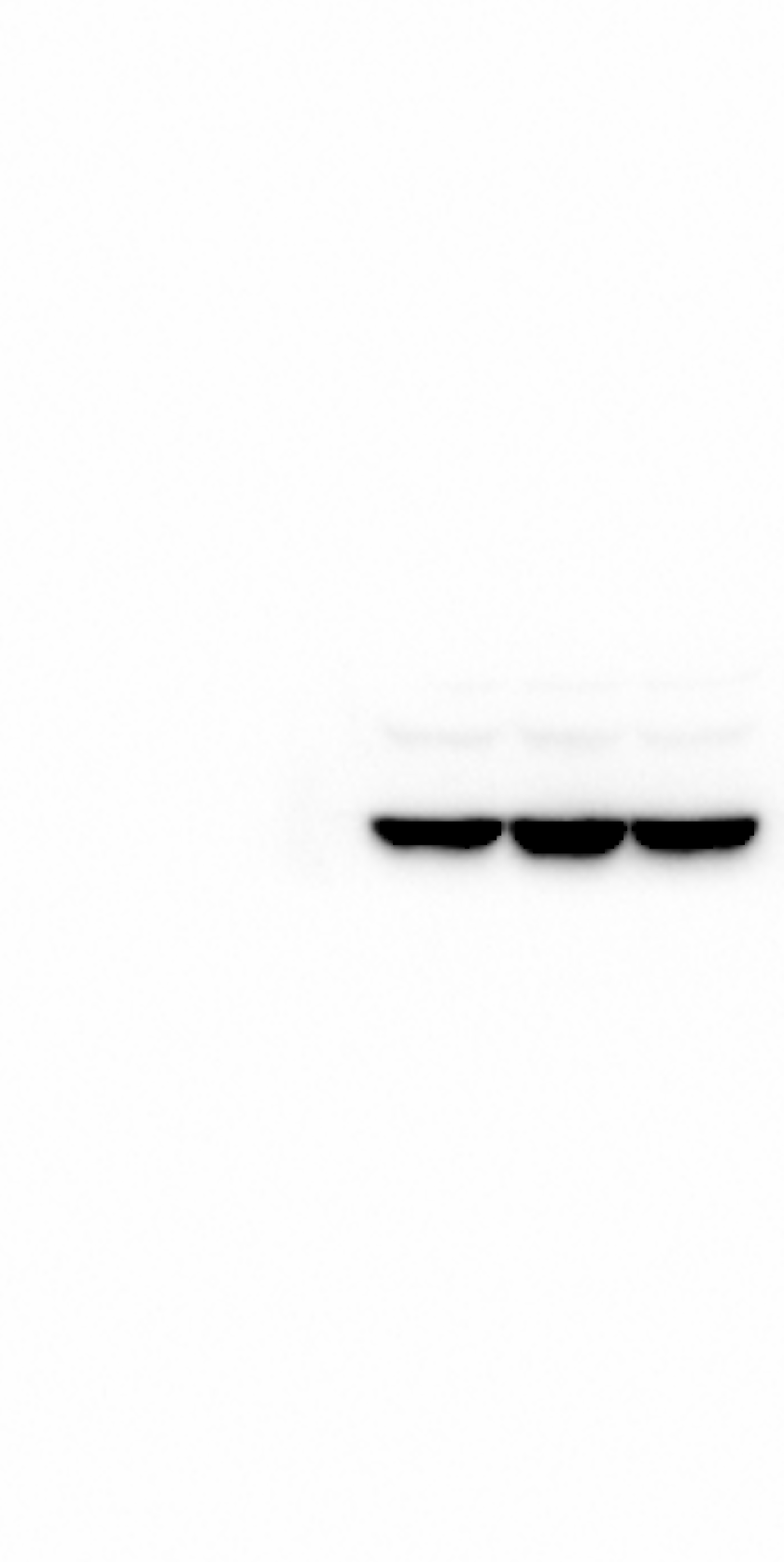

Supplement: Supplemental Information 43 [file peerj-14-21375-s043.zip › Figure 5I WB RAW OE-KLHL40 DES/DES-2 oe-KLHL40-ACTB.tif]

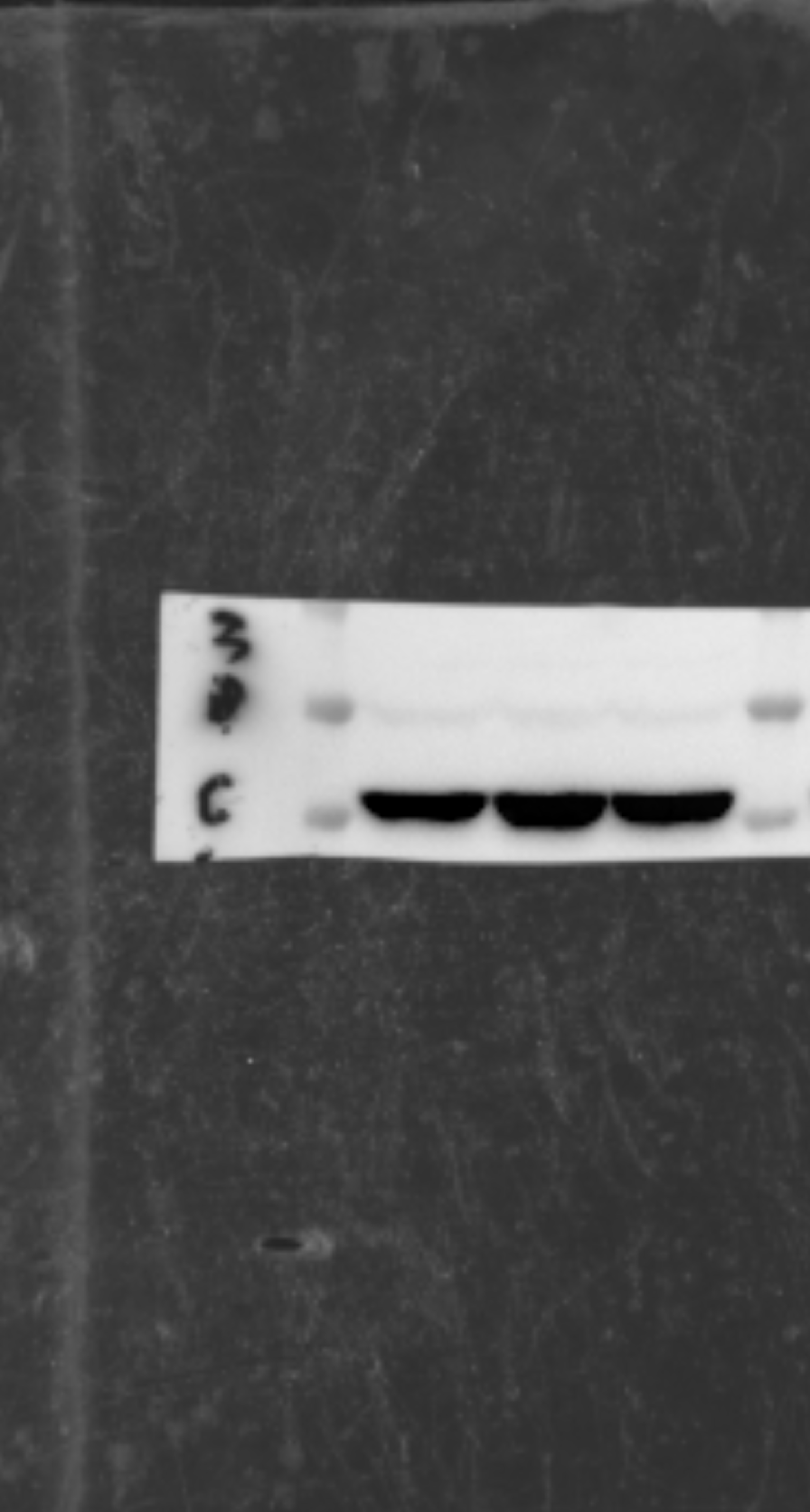

Supplement: Supplemental Information 43 [file peerj-14-21375-s043.zip › Figure 5I WB RAW OE-KLHL40 DES/DES-2 oe-KLHL40-ACTB+MARK.tif]

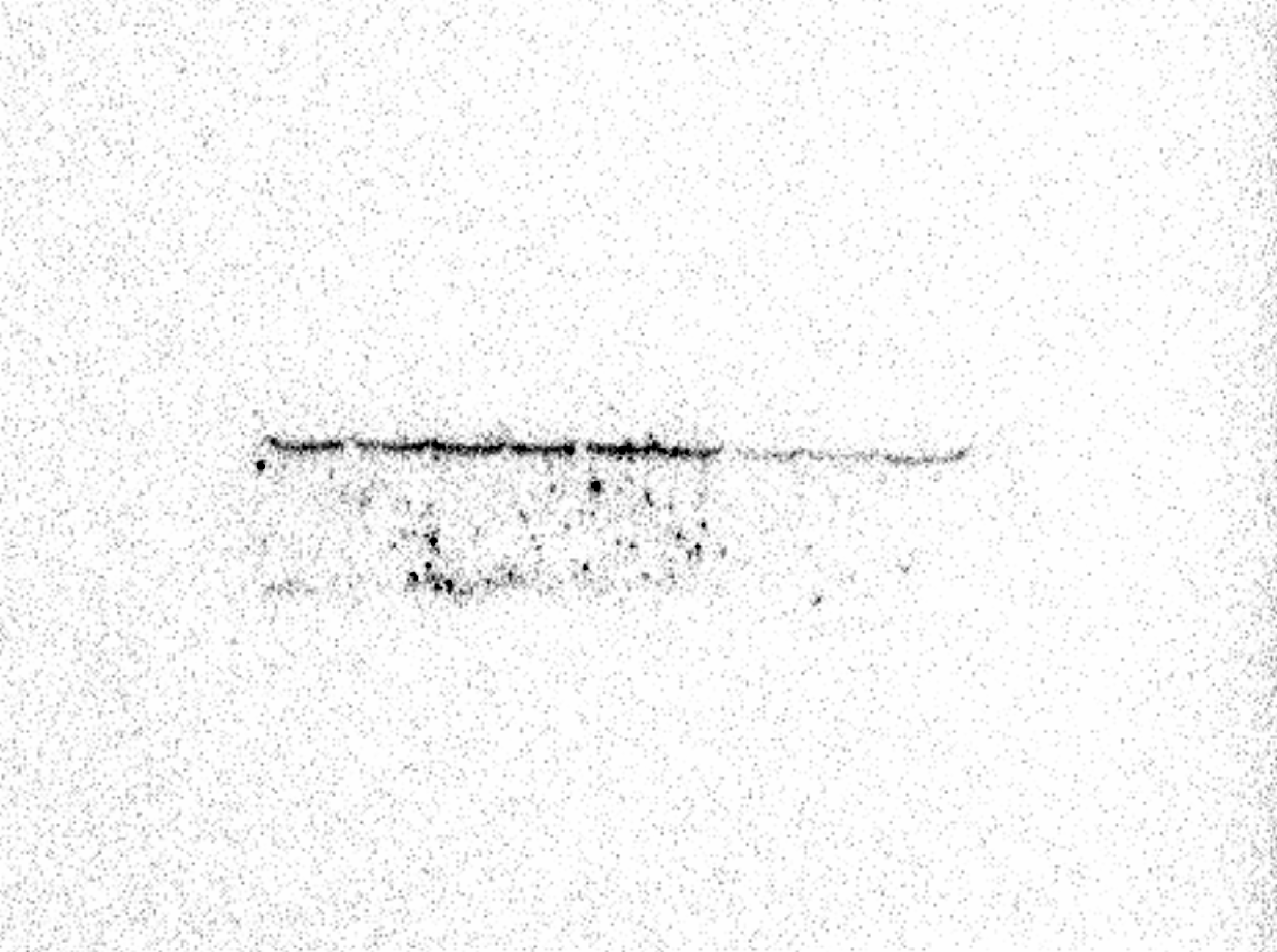

Supplement: Supplemental Information 43 [file peerj-14-21375-s043.zip › Figure 5I WB RAW OE-KLHL40 DES/DES-3 oe-KLHL40.tif]

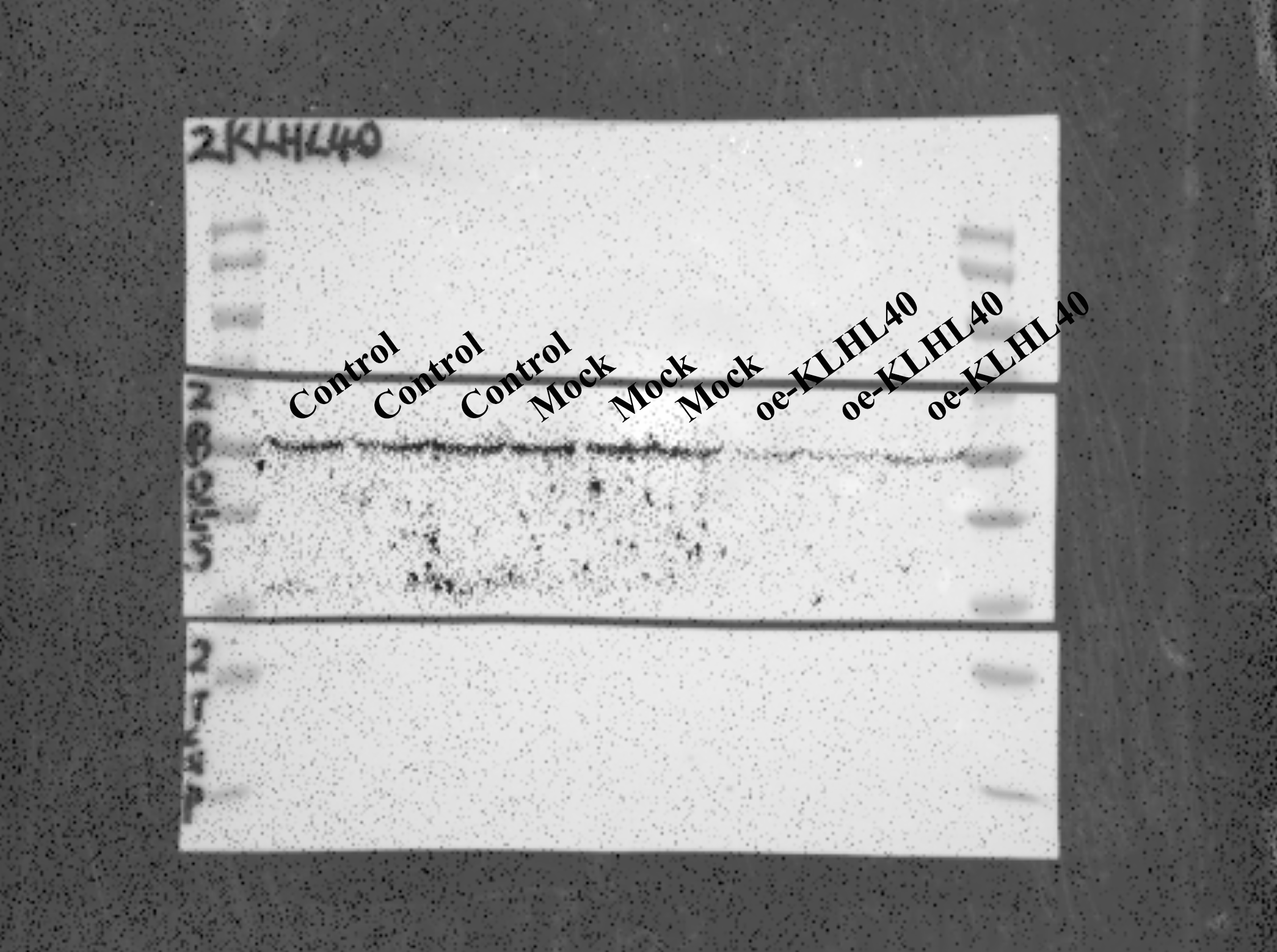

Supplement: Supplemental Information 43 [file peerj-14-21375-s043.zip › Figure 5I WB RAW OE-KLHL40 DES/DES-3 oe-KLHL40+MARK.tif]

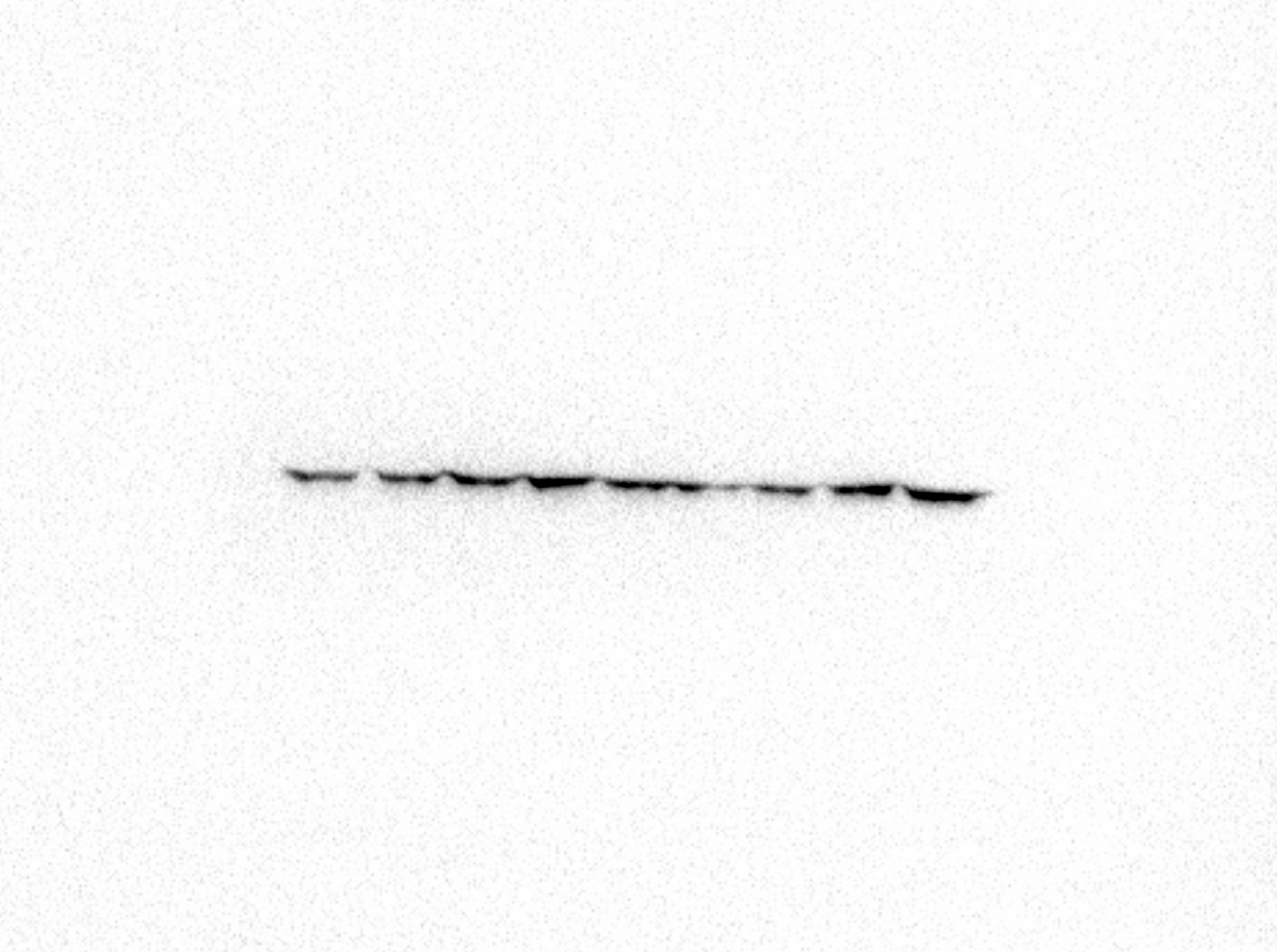

Supplement: Supplemental Information 43 [file peerj-14-21375-s043.zip › Figure 5I WB RAW OE-KLHL40 DES/DES-3 oe-KLHL40-ACTB.tif]

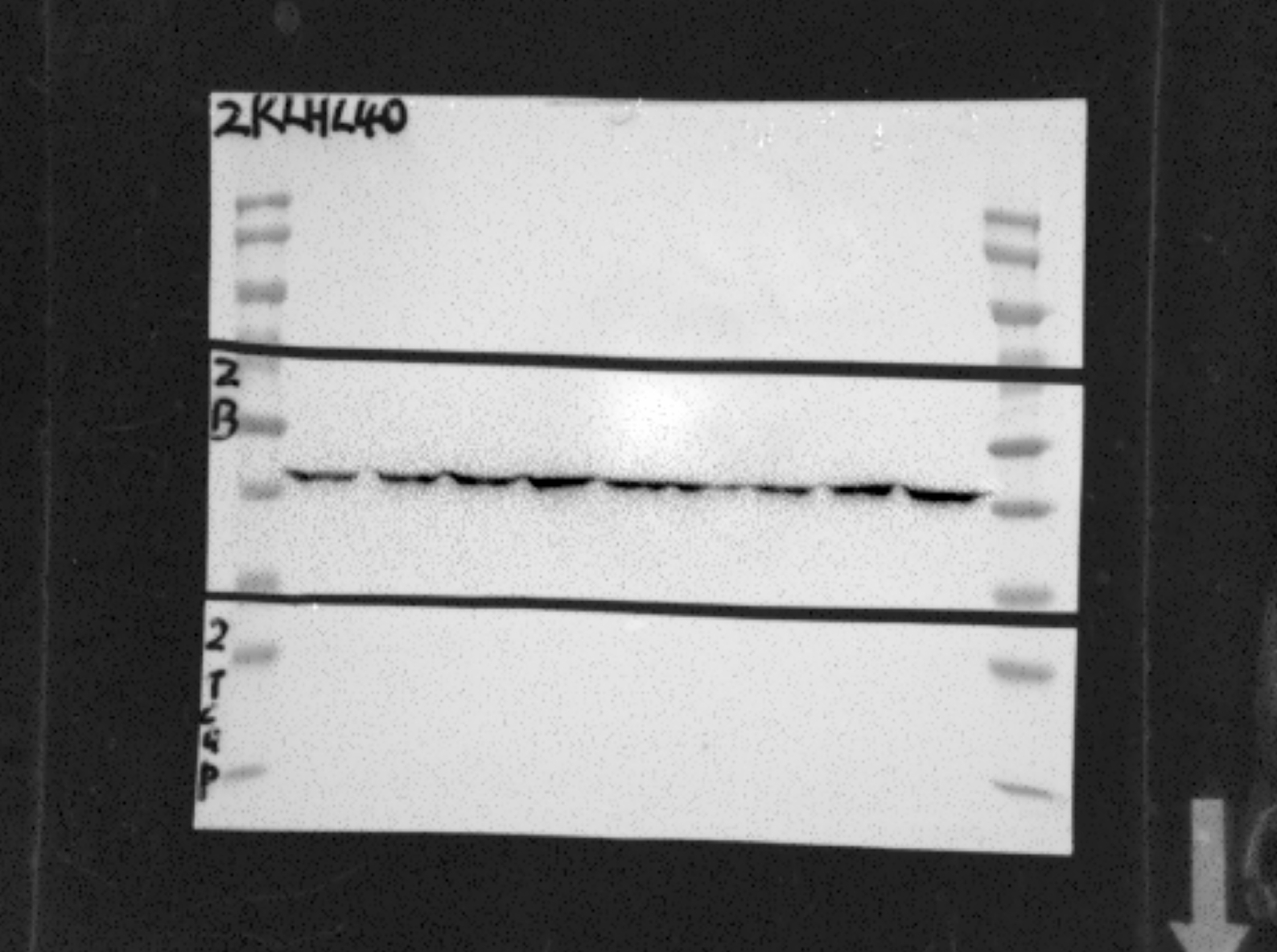

Supplement: Supplemental Information 43 [file peerj-14-21375-s043.zip › Figure 5I WB RAW OE-KLHL40 DES/DES-3 oe-KLHL40-ACTB+MARK.tif]

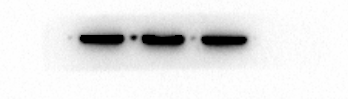

Supplement: Supplemental Information 45 [file peerj-14-21375-s045.zip › Figure 6A WB RAW SH-KLHL40 NLRP3/1ACTIN.png]

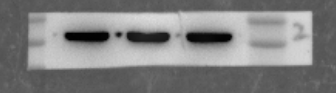

Supplement: Supplemental Information 45 [file peerj-14-21375-s045.zip › Figure 6A WB RAW SH-KLHL40 NLRP3/1ACTIN+MARKER.png]

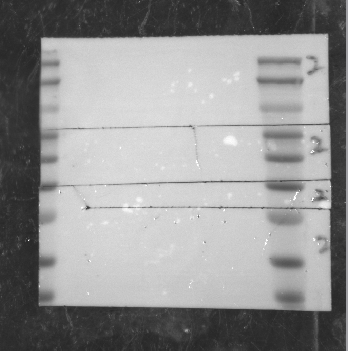

Supplement: Supplemental Information 45 [file peerj-14-21375-s045.zip › Figure 6A WB RAW SH-KLHL40 NLRP3/1ALL.png]

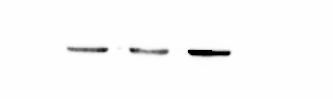

Supplement: Supplemental Information 45 [file peerj-14-21375-s045.zip › Figure 6A WB RAW SH-KLHL40 NLRP3/1NLRP3.png]

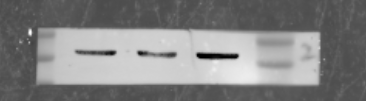

Supplement: Supplemental Information 45 [file peerj-14-21375-s045.zip › Figure 6A WB RAW SH-KLHL40 NLRP3/1NLRP3+MARKER.png]

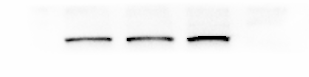

Supplement: Supplemental Information 45 [file peerj-14-21375-s045.zip › Figure 6A WB RAW SH-KLHL40 NLRP3/2NLRP3.png]

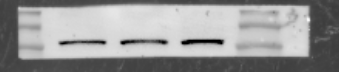

Supplement: Supplemental Information 45 [file peerj-14-21375-s045.zip › Figure 6A WB RAW SH-KLHL40 NLRP3/2NLRP3+MARKER.png]

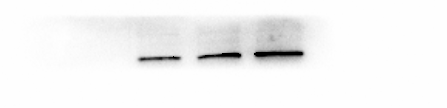

Supplement: Supplemental Information 45 [file peerj-14-21375-s045.zip › Figure 6A WB RAW SH-KLHL40 NLRP3/3-NLRP3.png]

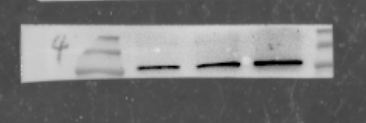

Supplement: Supplemental Information 45 [file peerj-14-21375-s045.zip › Figure 6A WB RAW SH-KLHL40 NLRP3/3-NLRP3-MARKER.png]

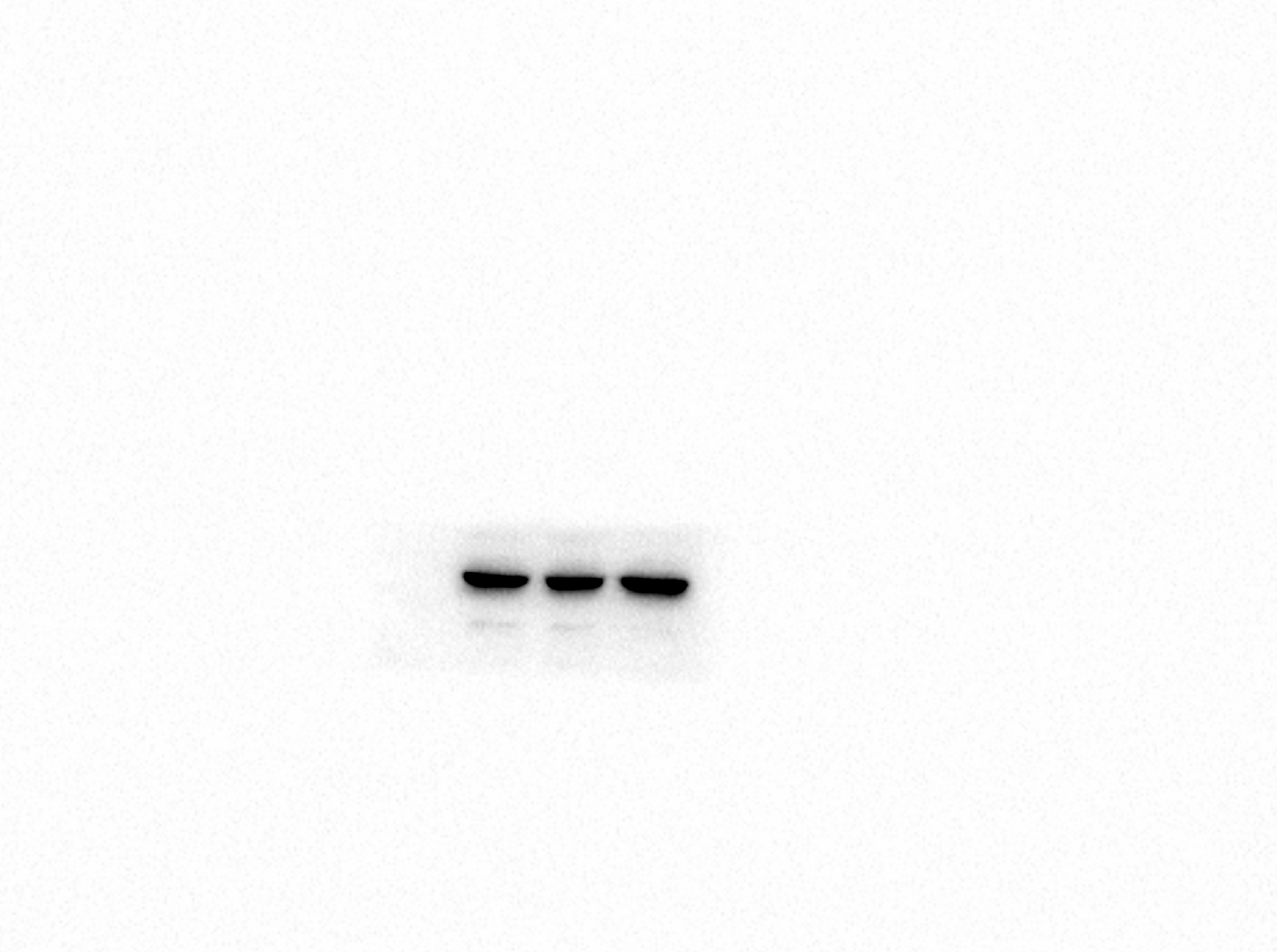

Supplement: Supplemental Information 46 [file peerj-14-21375-s046.zip › Figure 6B WB RAW OE-KLHL40 NLRP3/1ACTIN.tif]

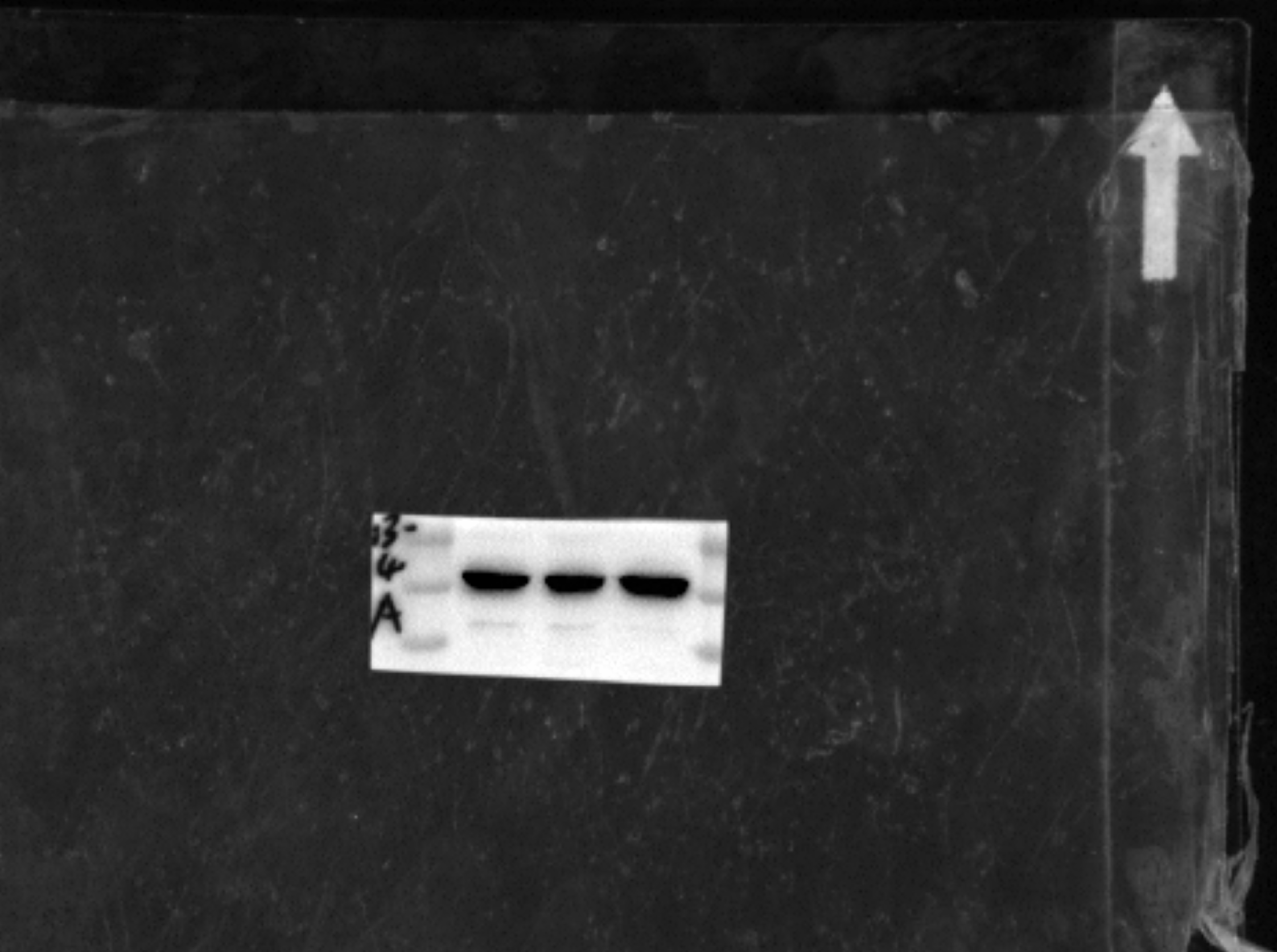

Supplement: Supplemental Information 46 [file peerj-14-21375-s046.zip › Figure 6B WB RAW OE-KLHL40 NLRP3/1ACTIN+MARKER.tif]

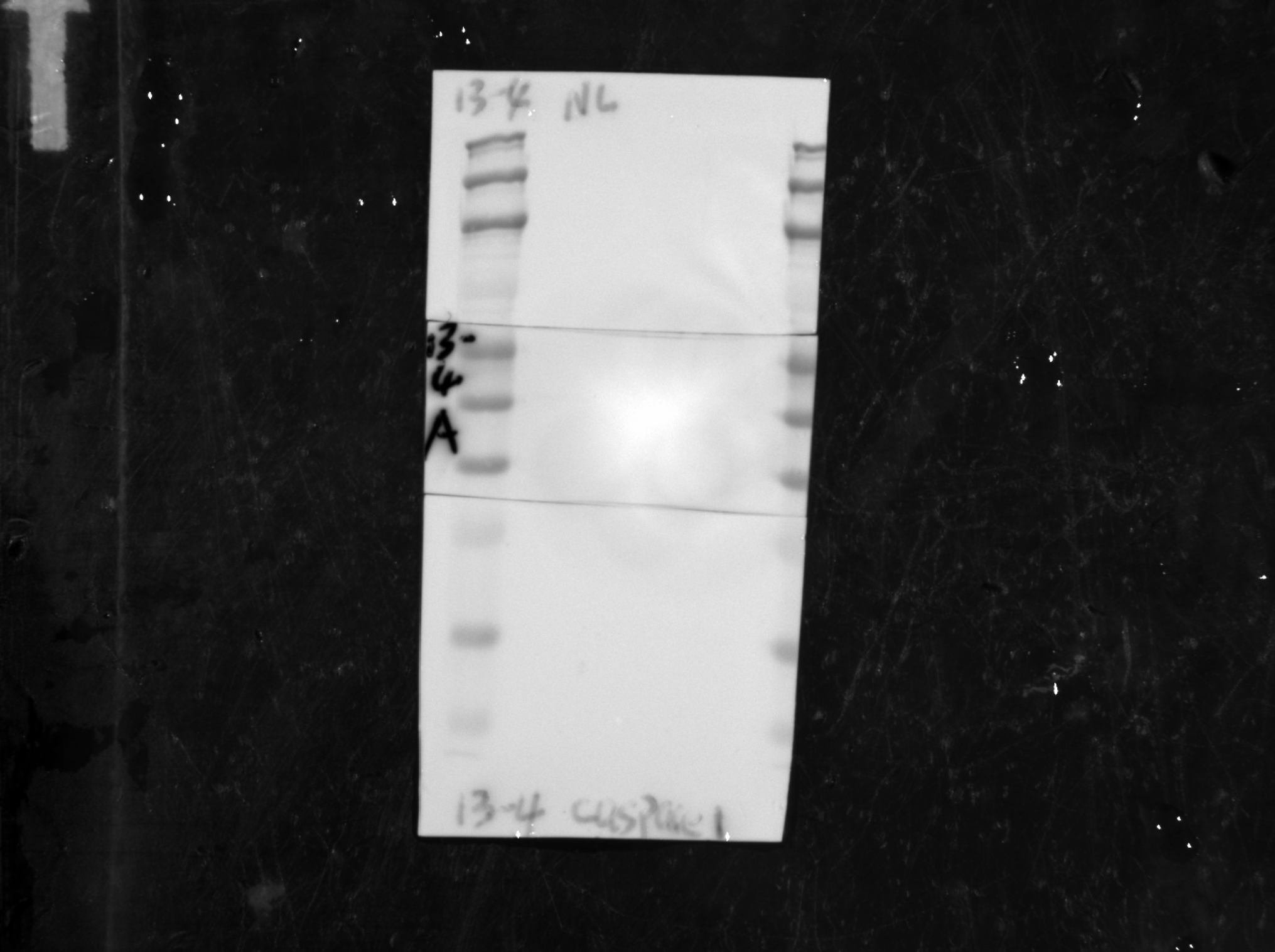

Supplement: Supplemental Information 46 [file peerj-14-21375-s046.zip › Figure 6B WB RAW OE-KLHL40 NLRP3/1ALL.tif]

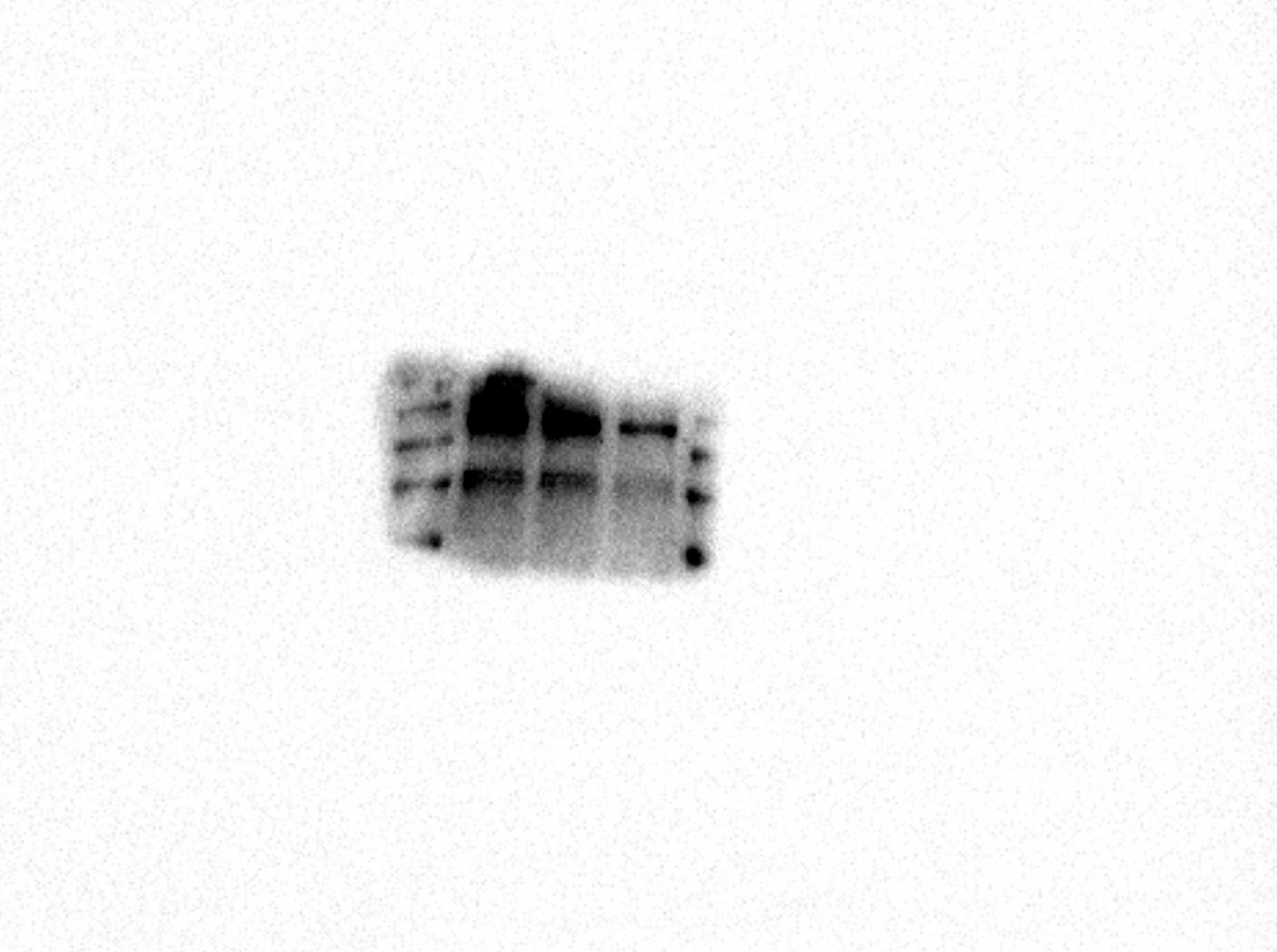

Supplement: Supplemental Information 46 [file peerj-14-21375-s046.zip › Figure 6B WB RAW OE-KLHL40 NLRP3/1NLRP3.tif]

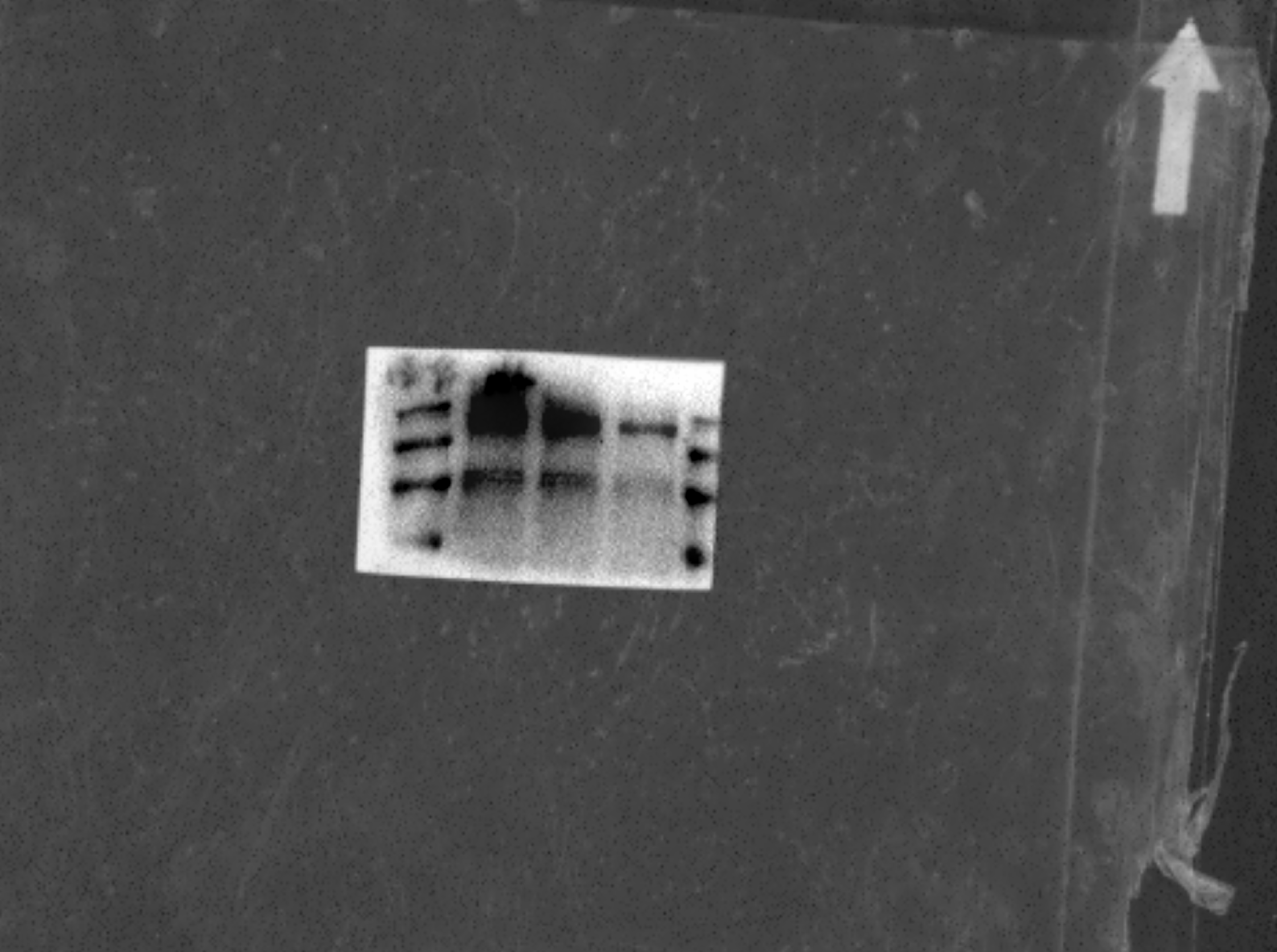

Supplement: Supplemental Information 46 [file peerj-14-21375-s046.zip › Figure 6B WB RAW OE-KLHL40 NLRP3/1NLRP3+MARKER.tif]

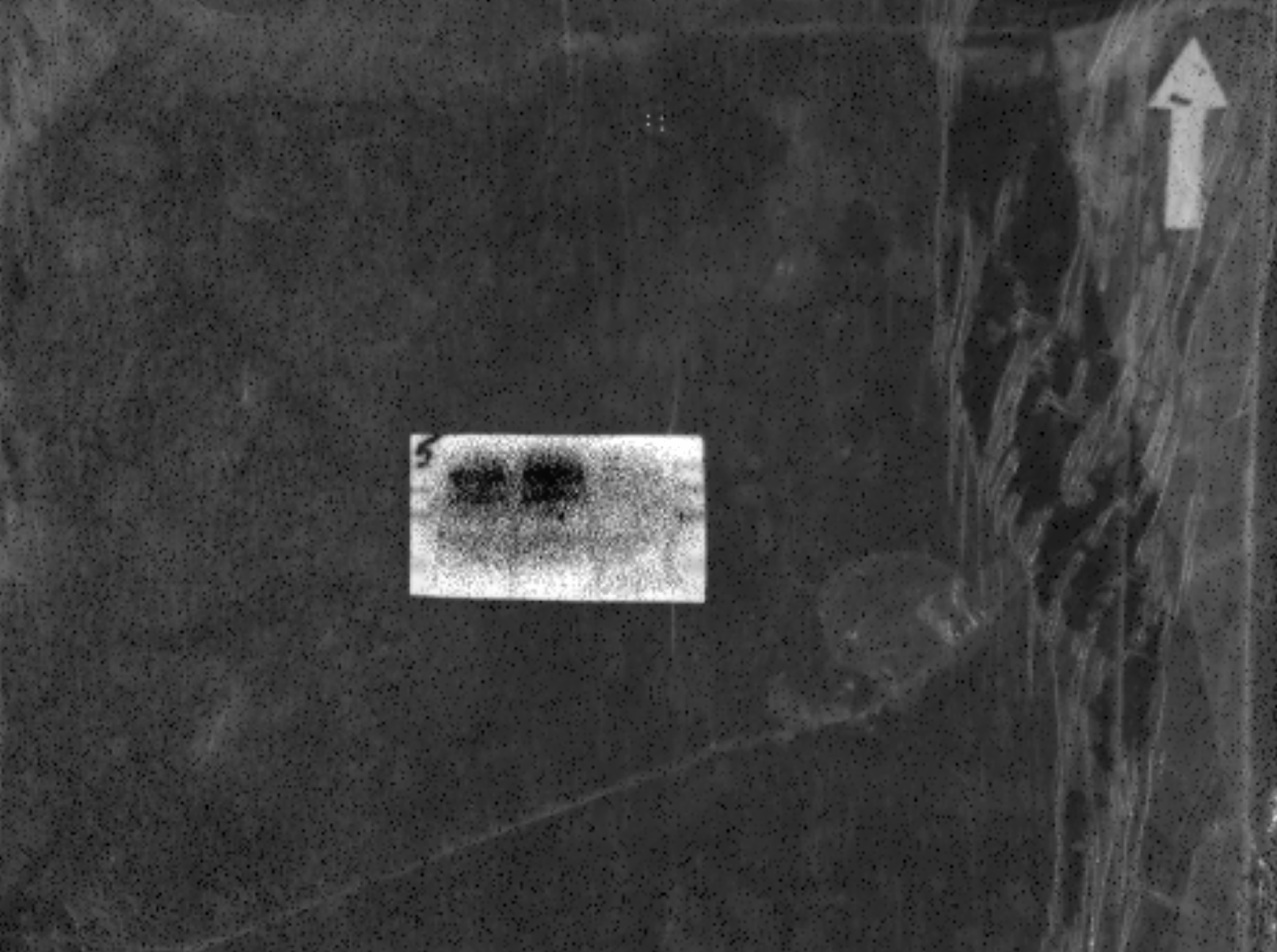

Supplement: Supplemental Information 46 [file peerj-14-21375-s046.zip › Figure 6B WB RAW OE-KLHL40 NLRP3/2 NPLR3+ MARKER.tif]

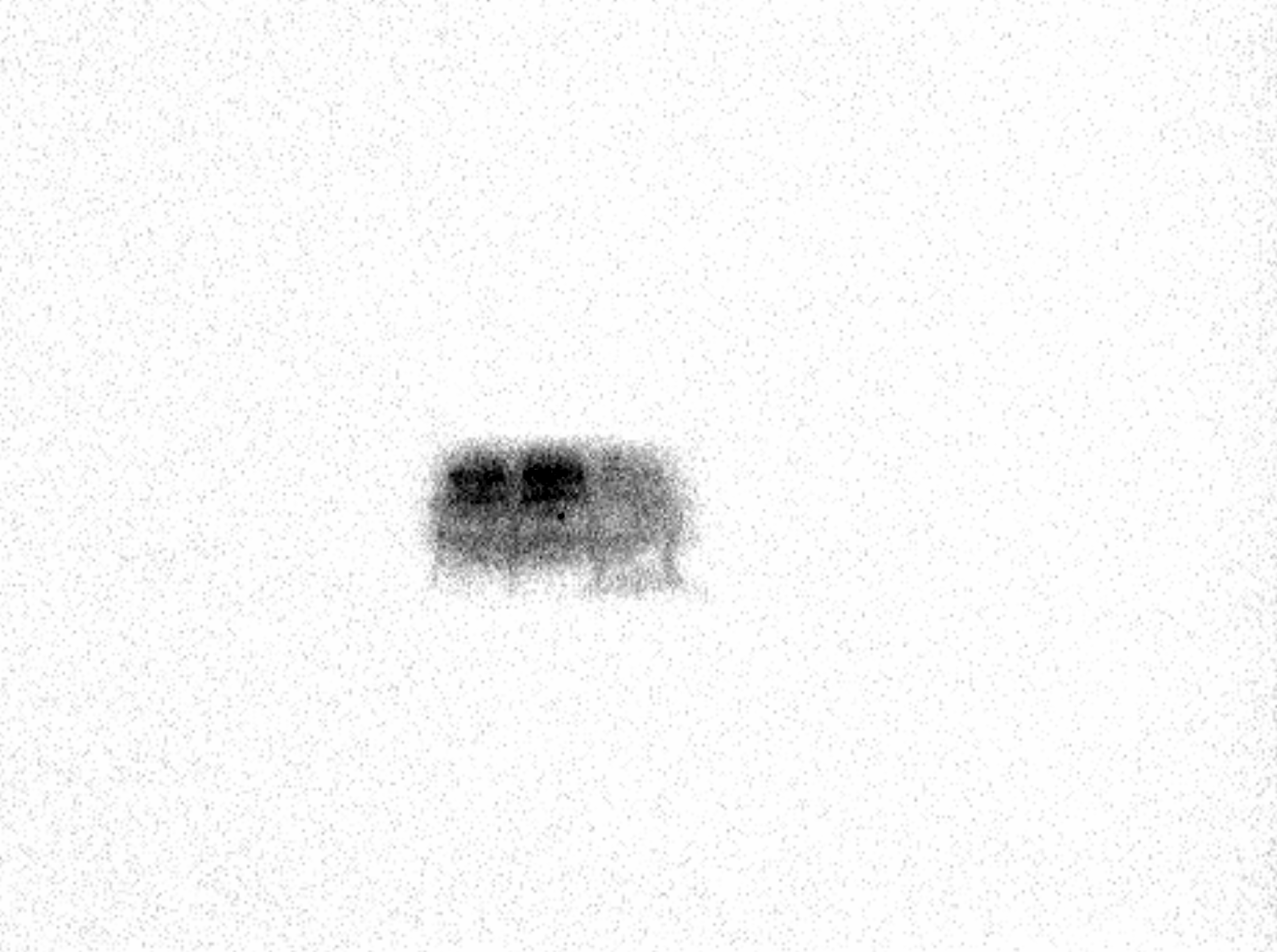

Supplement: Supplemental Information 46 [file peerj-14-21375-s046.zip › Figure 6B WB RAW OE-KLHL40 NLRP3/2NPLR3.tif]

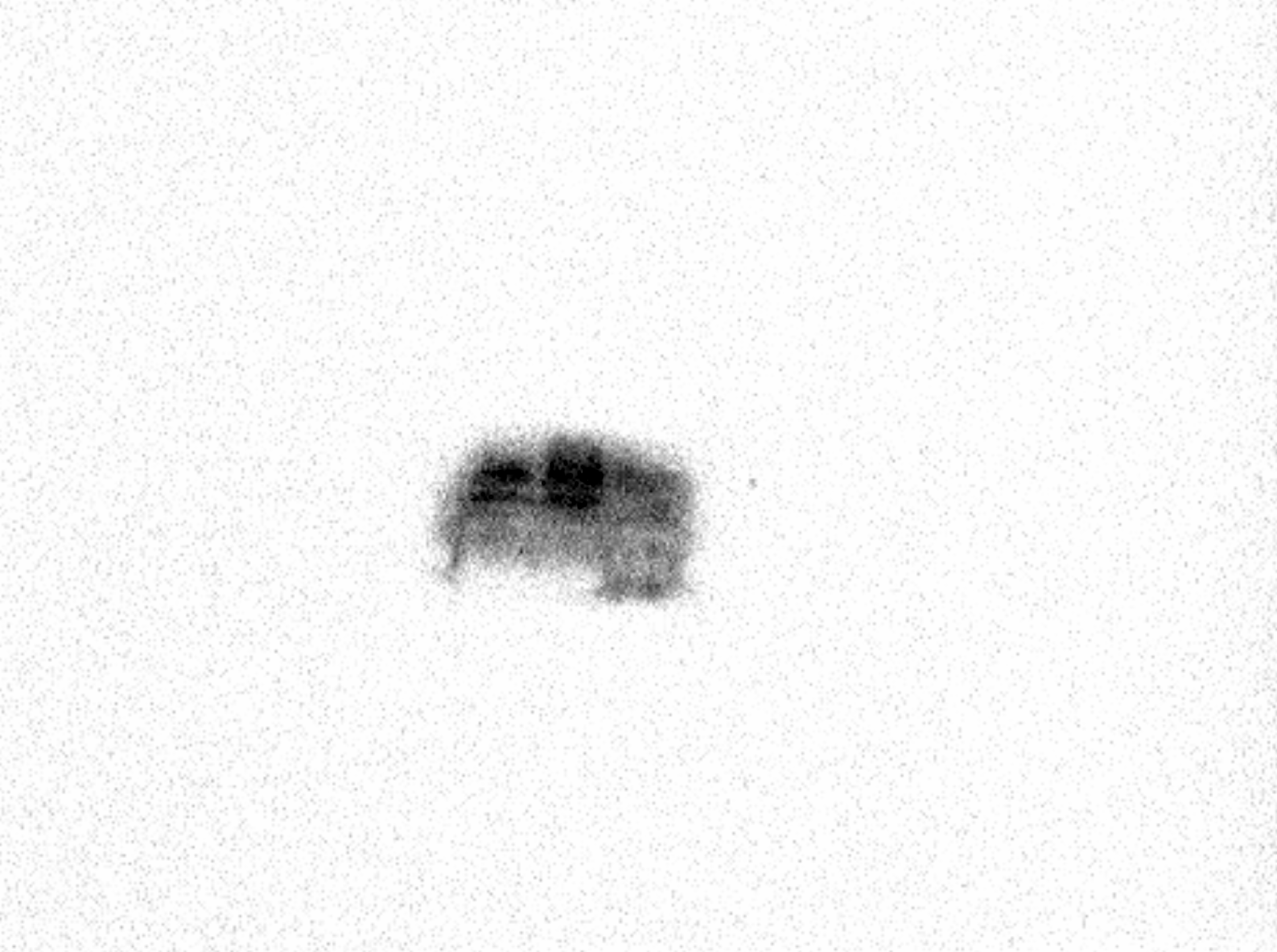

Supplement: Supplemental Information 46 [file peerj-14-21375-s046.zip › Figure 6B WB RAW OE-KLHL40 NLRP3/3 NLRP3.tif]

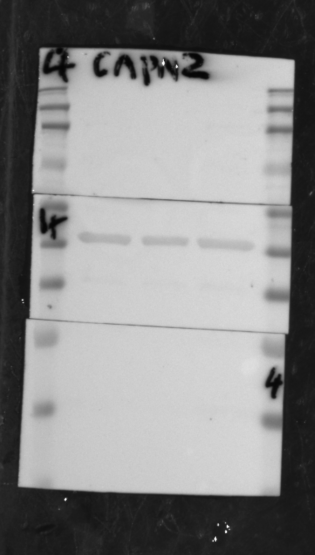

Supplement: Supplemental Information 46 [file peerj-14-21375-s046.zip › Figure 6B WB RAW OE-KLHL40 NLRP3/3ALL.png]

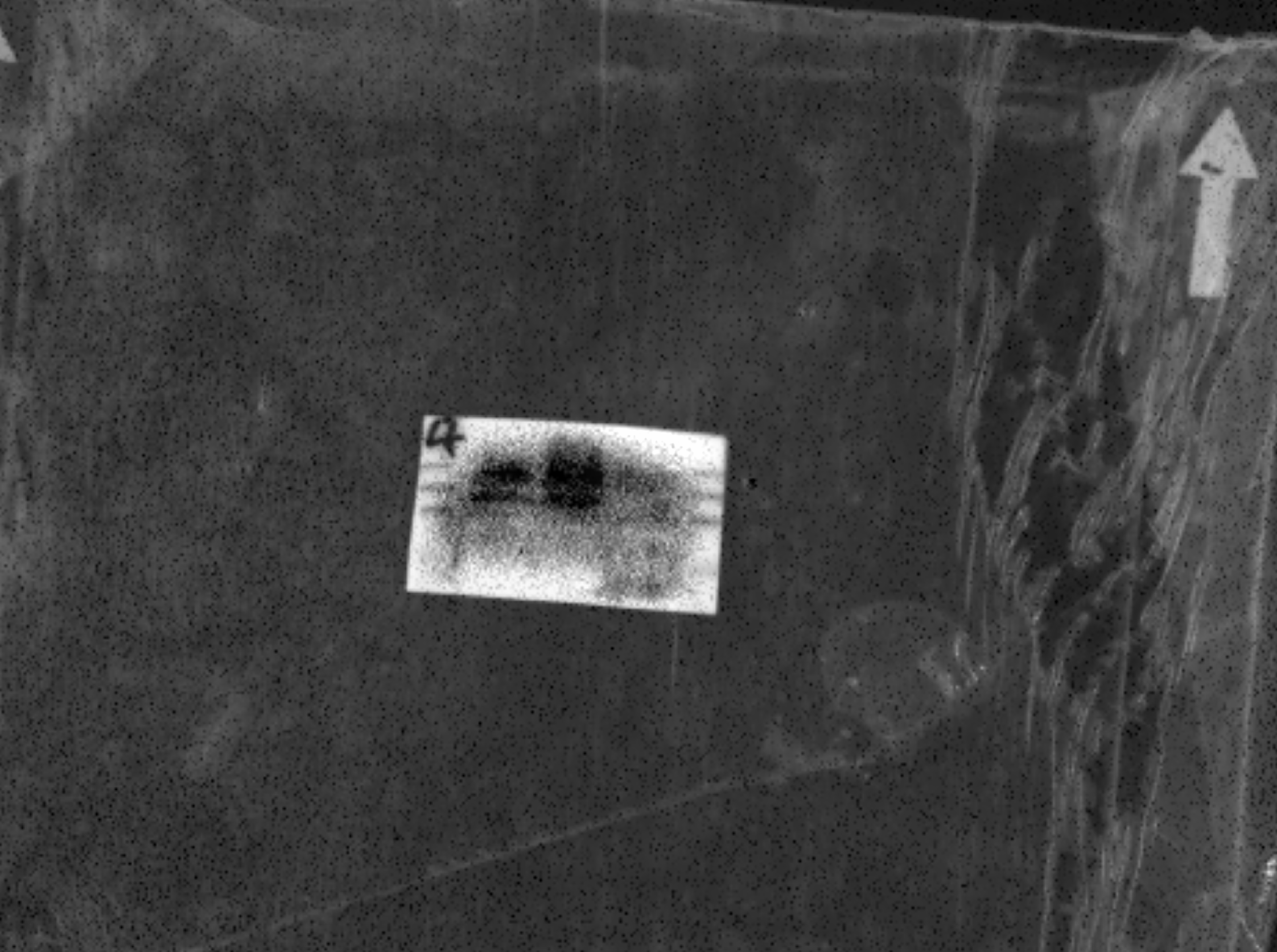

Supplement: Supplemental Information 46 [file peerj-14-21375-s046.zip › Figure 6B WB RAW OE-KLHL40 NLRP3/3NLRP3+MARKER.tif]

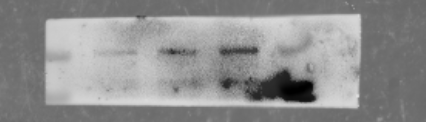

Supplement: Supplemental Information 47 [file peerj-14-21375-s047.zip › Figure 6C WB RAW SH-KLHL40 Cleaved CASPASE1/1-C caspe1-MARKER.png]

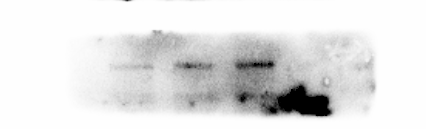

Supplement: Supplemental Information 47 [file peerj-14-21375-s047.zip › Figure 6C WB RAW SH-KLHL40 Cleaved CASPASE1/1-C-caspe1.png]

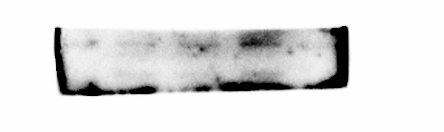

Supplement: Supplemental Information 47 [file peerj-14-21375-s047.zip › Figure 6C WB RAW SH-KLHL40 Cleaved CASPASE1/2- C CASPE1.png]

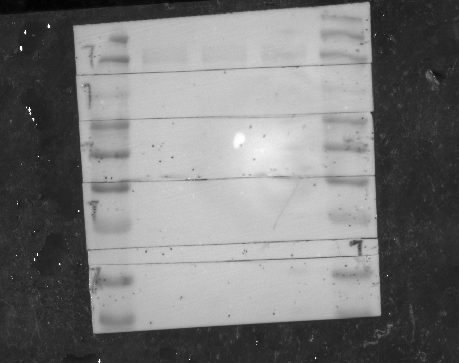

Supplement: Supplemental Information 47 [file peerj-14-21375-s047.zip › Figure 6C WB RAW SH-KLHL40 Cleaved CASPASE1/2ALL.png]

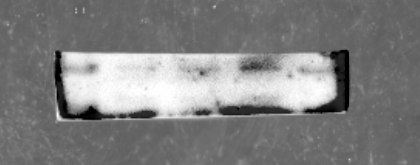

Supplement: Supplemental Information 47 [file peerj-14-21375-s047.zip › Figure 6C WB RAW SH-KLHL40 Cleaved CASPASE1/2-C CASPE1-MARKER.png]

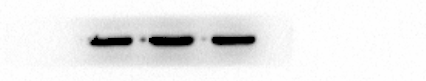

Supplement: Supplemental Information 47 [file peerj-14-21375-s047.zip › Figure 6C WB RAW SH-KLHL40 Cleaved CASPASE1/3-ACTIN.png]

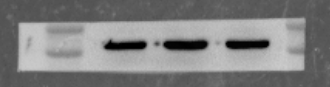

Supplement: Supplemental Information 47 [file peerj-14-21375-s047.zip › Figure 6C WB RAW SH-KLHL40 Cleaved CASPASE1/3-ACTIN-MARKER.png]

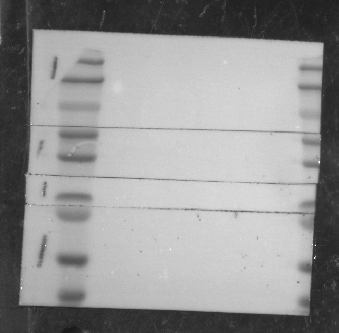

Supplement: Supplemental Information 47 [file peerj-14-21375-s047.zip › Figure 6C WB RAW SH-KLHL40 Cleaved CASPASE1/3ALL.png]

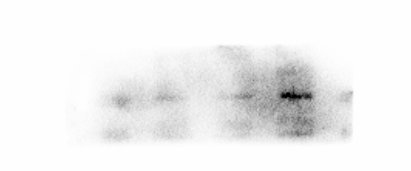

Supplement: Supplemental Information 47 [file peerj-14-21375-s047.zip › Figure 6C WB RAW SH-KLHL40 Cleaved CASPASE1/3-C-caspe1.png]

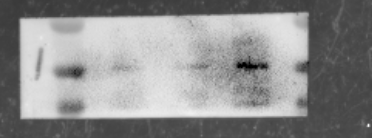

Supplement: Supplemental Information 47 [file peerj-14-21375-s047.zip › Figure 6C WB RAW SH-KLHL40 Cleaved CASPASE1/3-C-caspe1-MARKER.png]

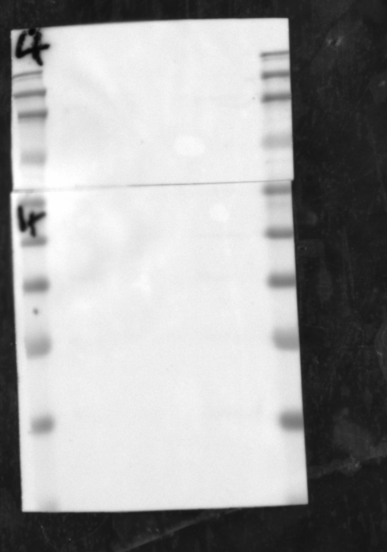

Supplement: Supplemental Information 48 [file peerj-14-21375-s048.zip › Figure 6D WB RAW OE-KLHL40 Cleaved CASPASE1/1ALL.png]

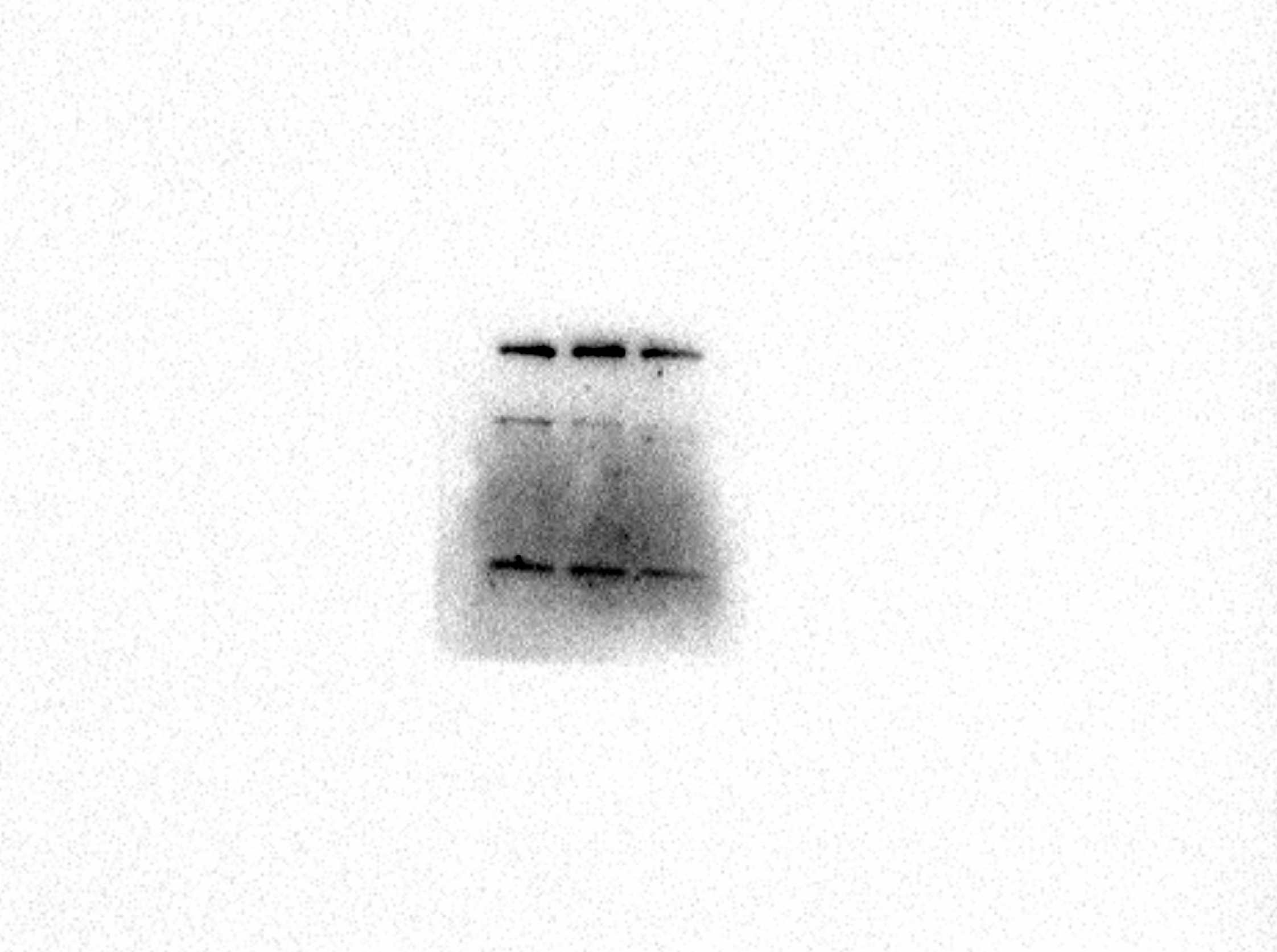

Supplement: Supplemental Information 48 [file peerj-14-21375-s048.zip › Figure 6D WB RAW OE-KLHL40 Cleaved CASPASE1/1C-Caspase1.tif]

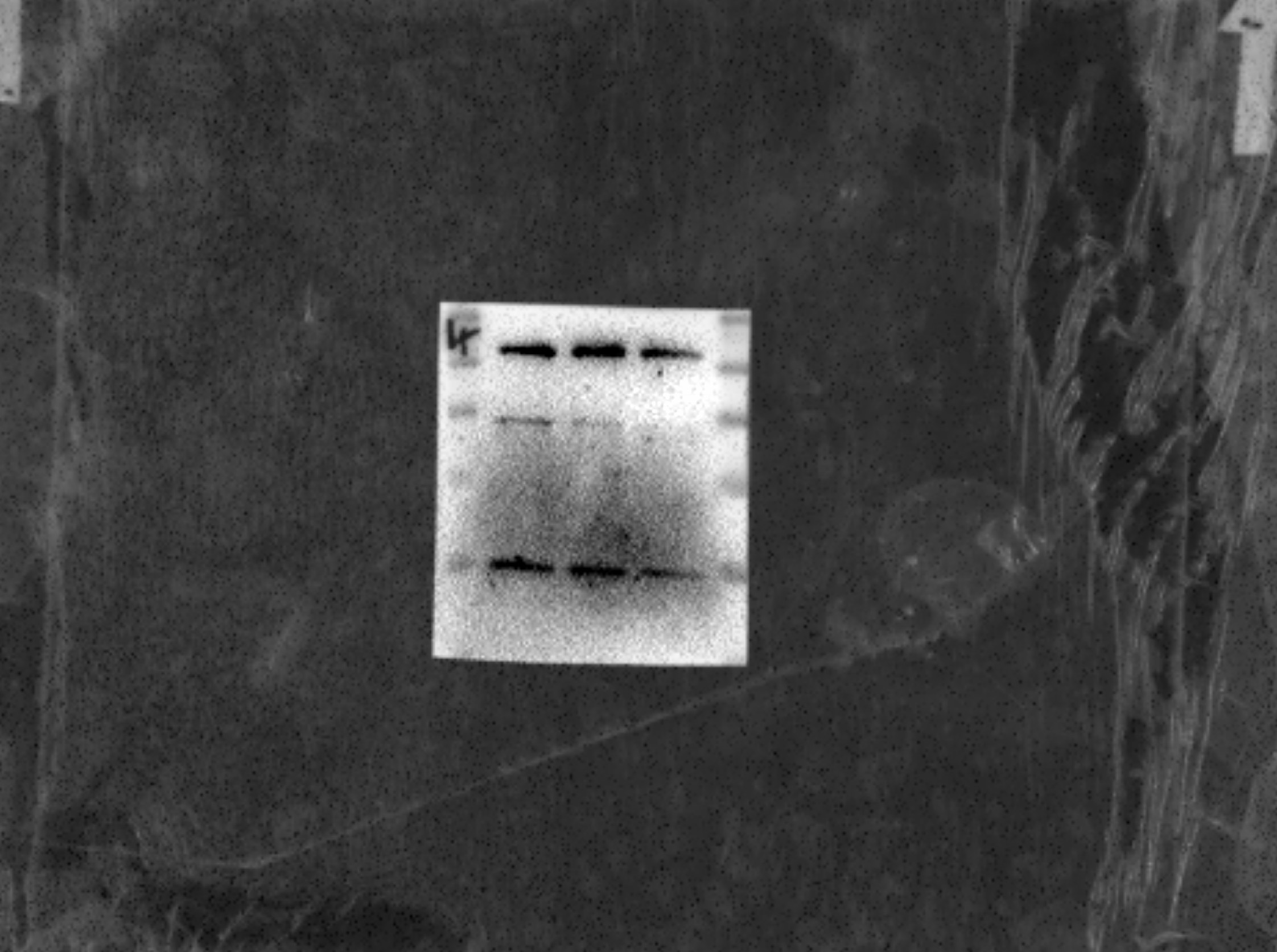

Supplement: Supplemental Information 48 [file peerj-14-21375-s048.zip › Figure 6D WB RAW OE-KLHL40 Cleaved CASPASE1/1C-CASPASE1+MARKER.tif]

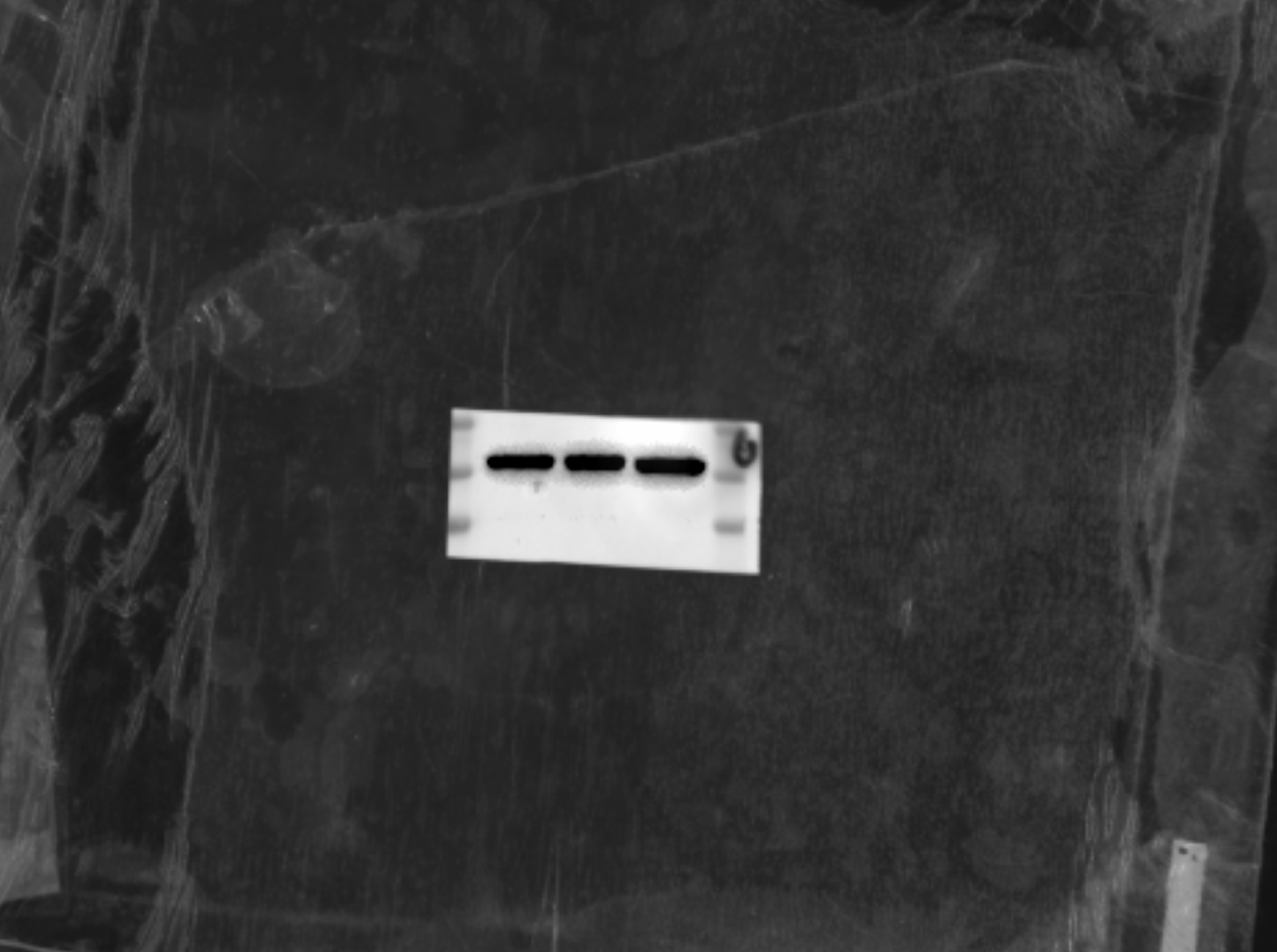

Supplement: Supplemental Information 48 [file peerj-14-21375-s048.zip › Figure 6D WB RAW OE-KLHL40 Cleaved CASPASE1/2 ACTIN+MARKER.tif]

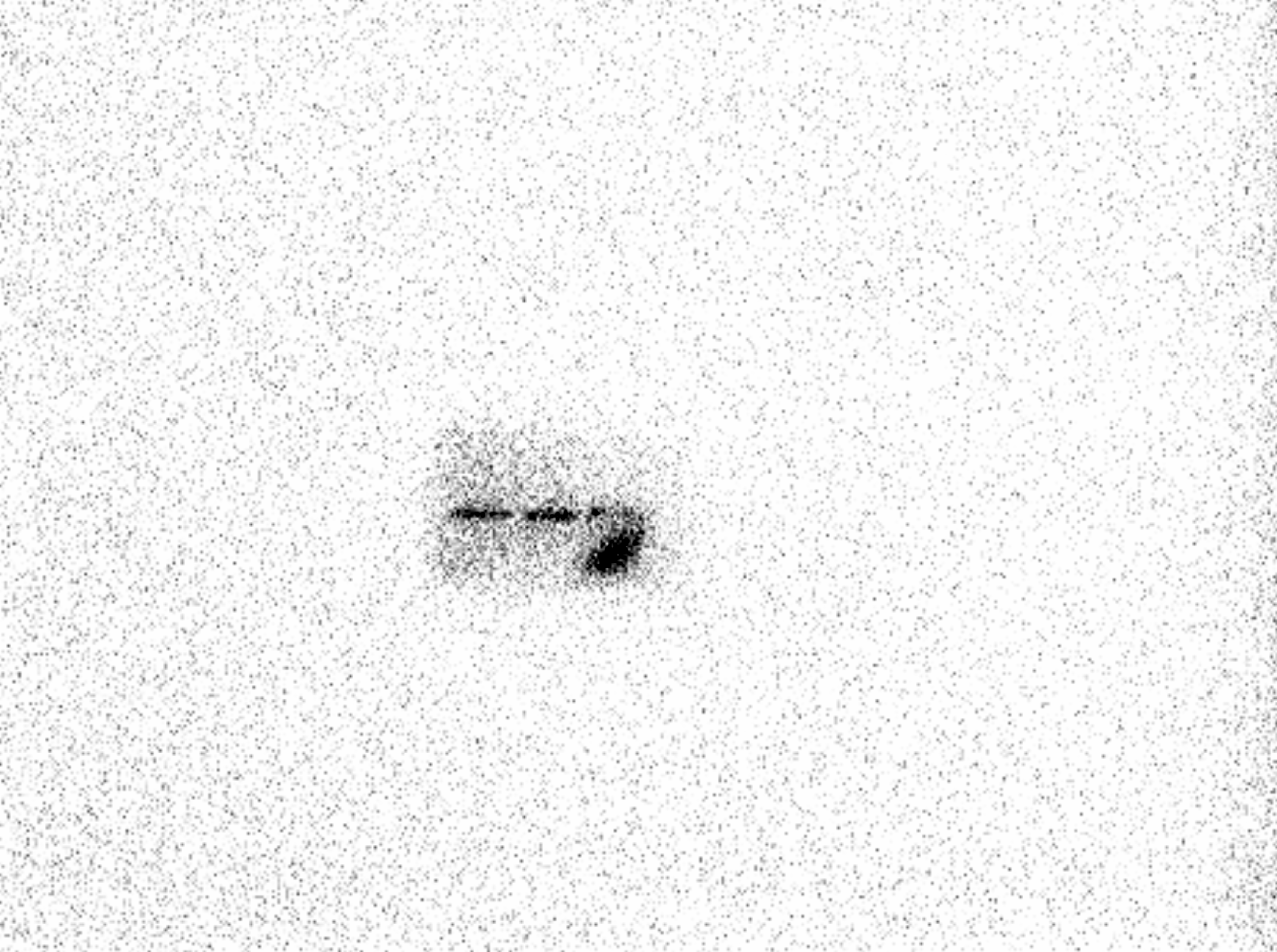

Supplement: Supplemental Information 48 [file peerj-14-21375-s048.zip › Figure 6D WB RAW OE-KLHL40 Cleaved CASPASE1/2 C-CASPASE1.tif]

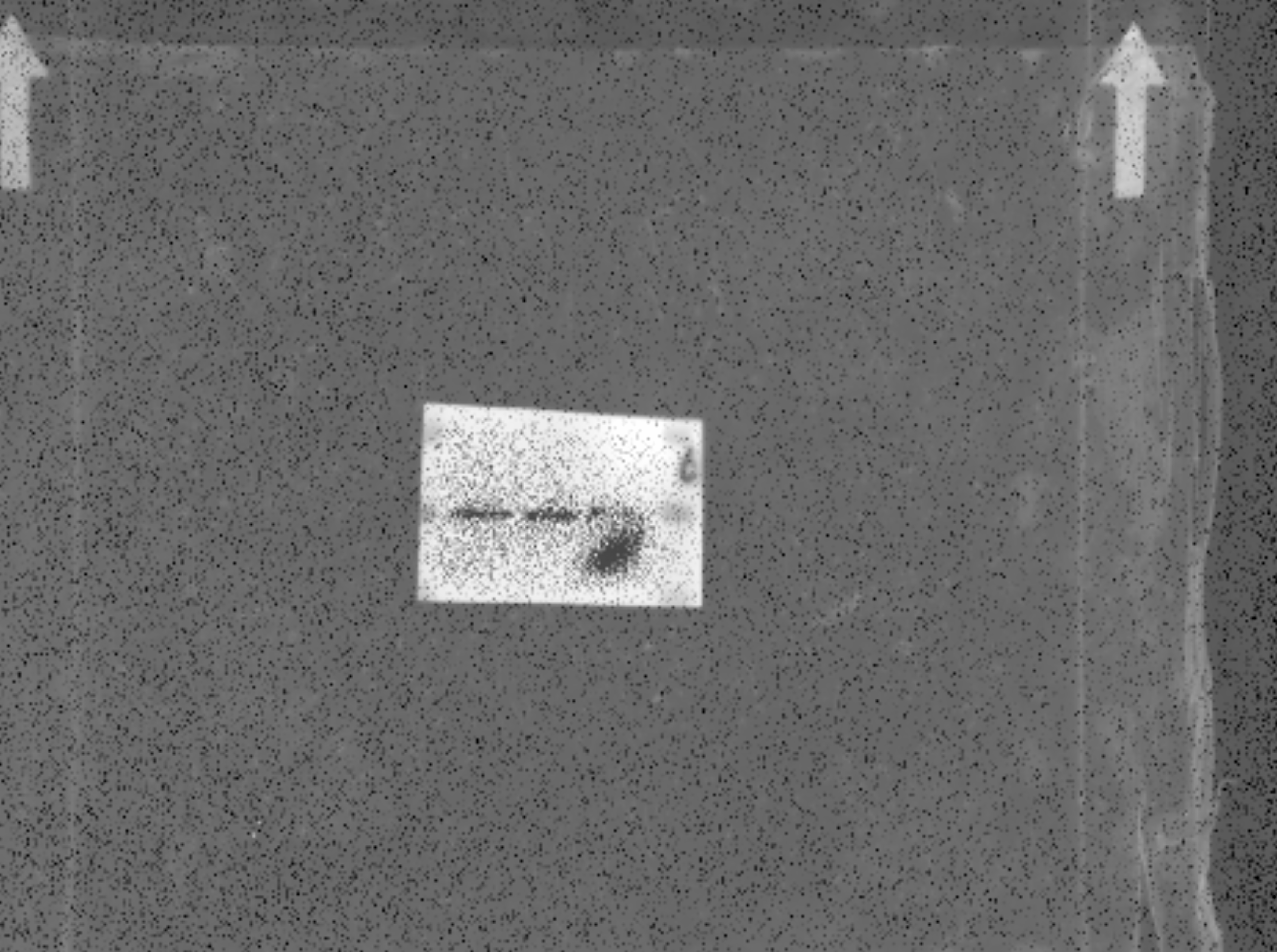

Supplement: Supplemental Information 48 [file peerj-14-21375-s048.zip › Figure 6D WB RAW OE-KLHL40 Cleaved CASPASE1/2 C-CASPASE1+MARKER.tif]

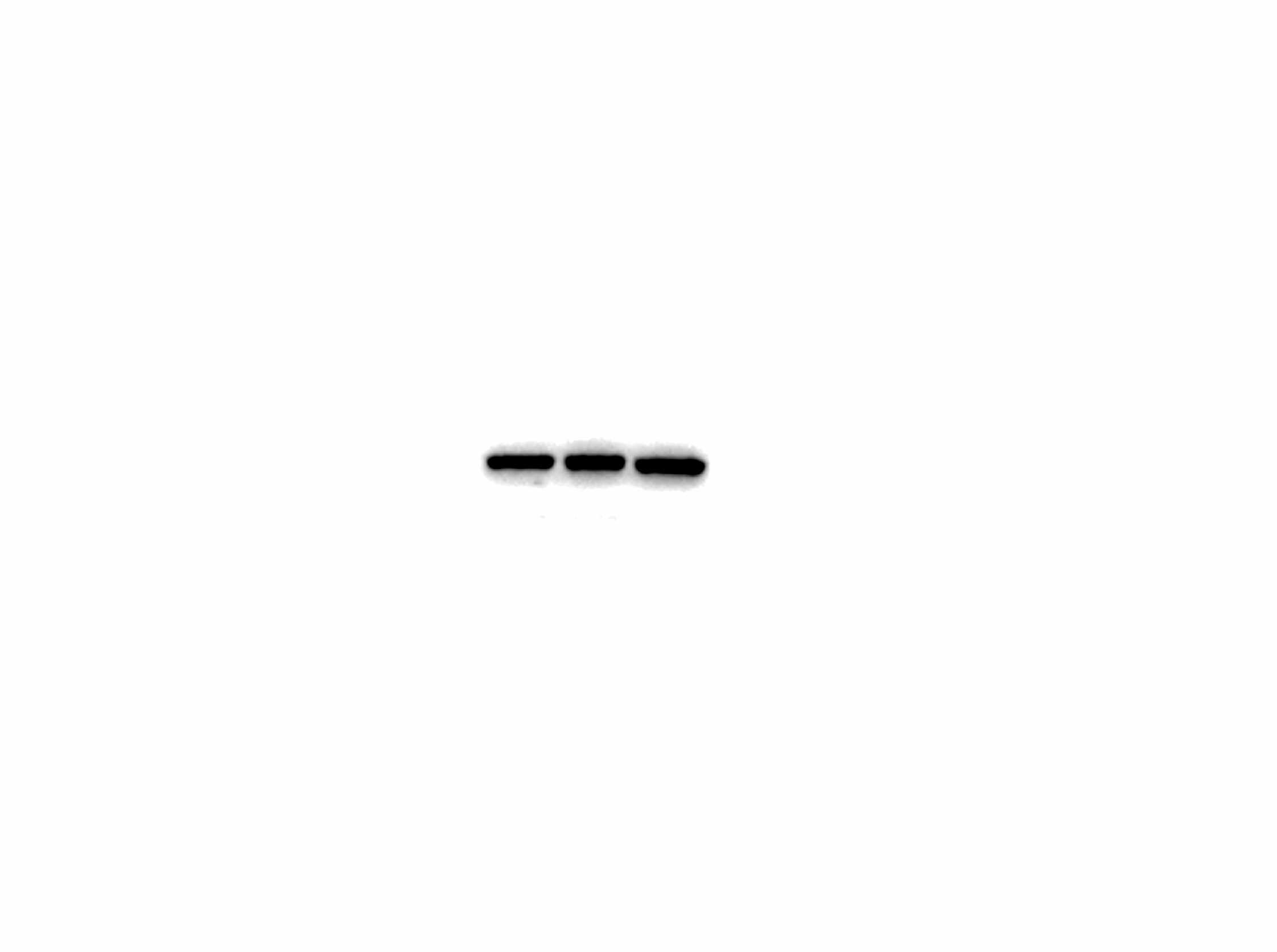

Supplement: Supplemental Information 48 [file peerj-14-21375-s048.zip › Figure 6D WB RAW OE-KLHL40 Cleaved CASPASE1/2ACTIN.tif]

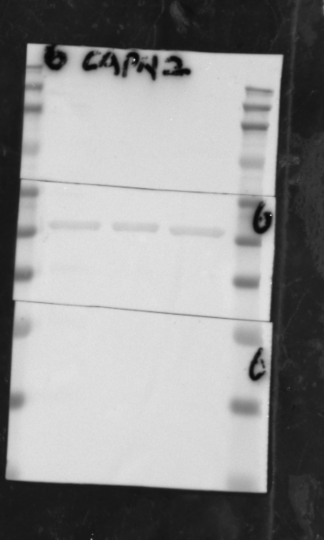

Supplement: Supplemental Information 48 [file peerj-14-21375-s048.zip › Figure 6D WB RAW OE-KLHL40 Cleaved CASPASE1/2ALL.png]

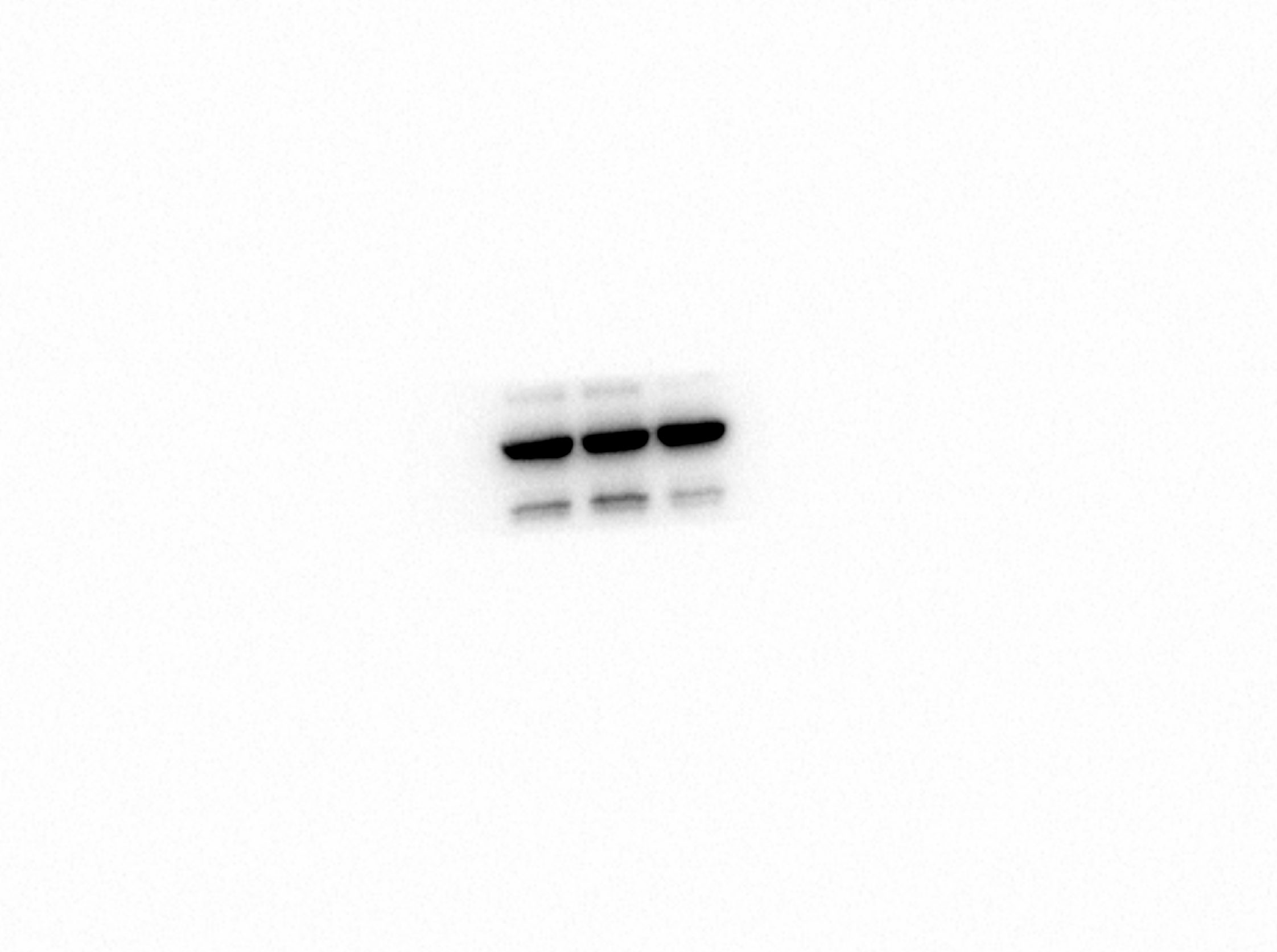

Supplement: Supplemental Information 48 [file peerj-14-21375-s048.zip › Figure 6D WB RAW OE-KLHL40 Cleaved CASPASE1/3ACTIN.tif]

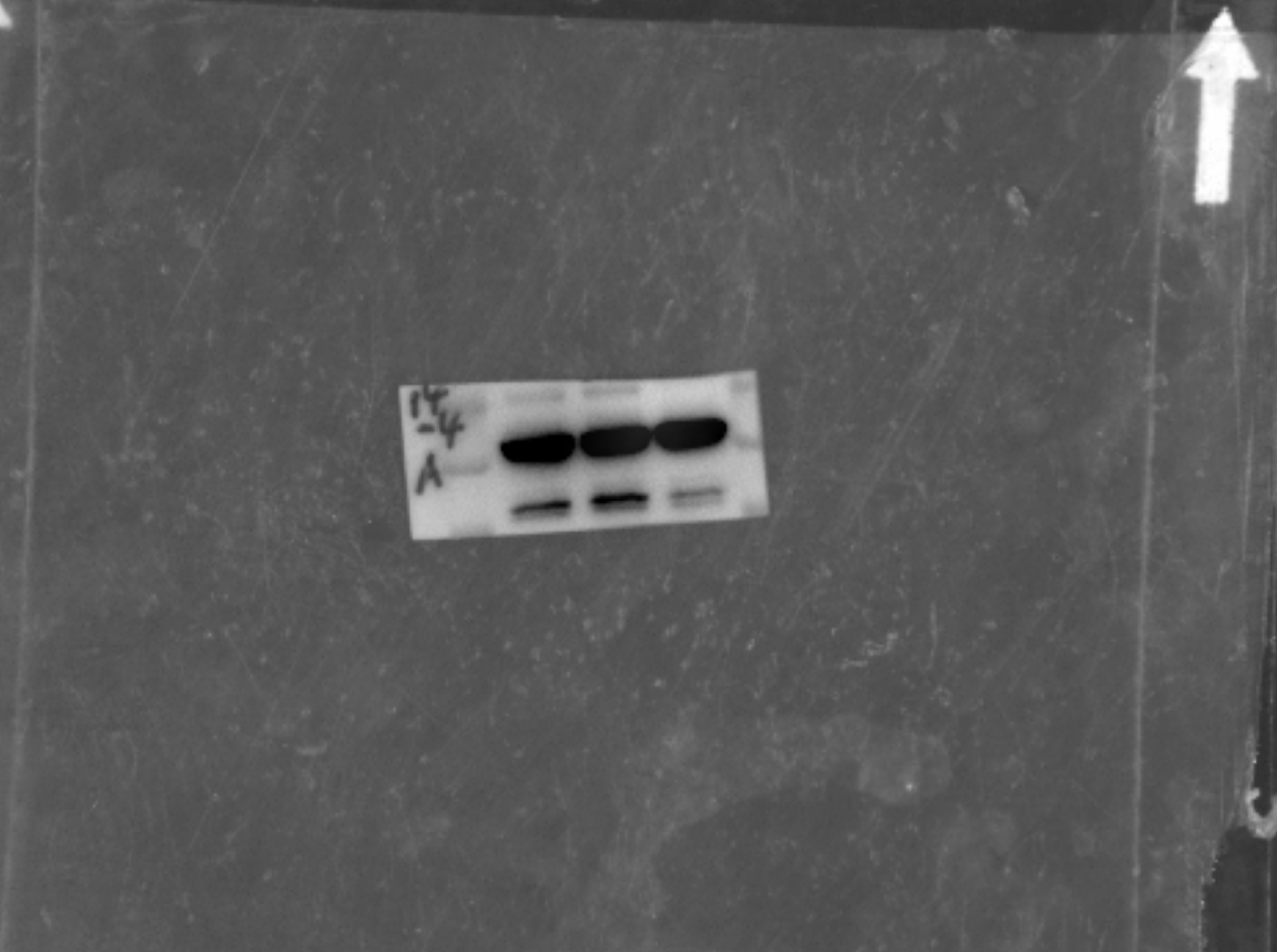

Supplement: Supplemental Information 48 [file peerj-14-21375-s048.zip › Figure 6D WB RAW OE-KLHL40 Cleaved CASPASE1/3ACTIN+MARKER.tif]

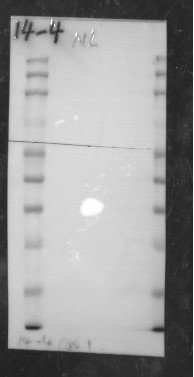

Supplement: Supplemental Information 48 [file peerj-14-21375-s048.zip › Figure 6D WB RAW OE-KLHL40 Cleaved CASPASE1/3ALL.jpg]

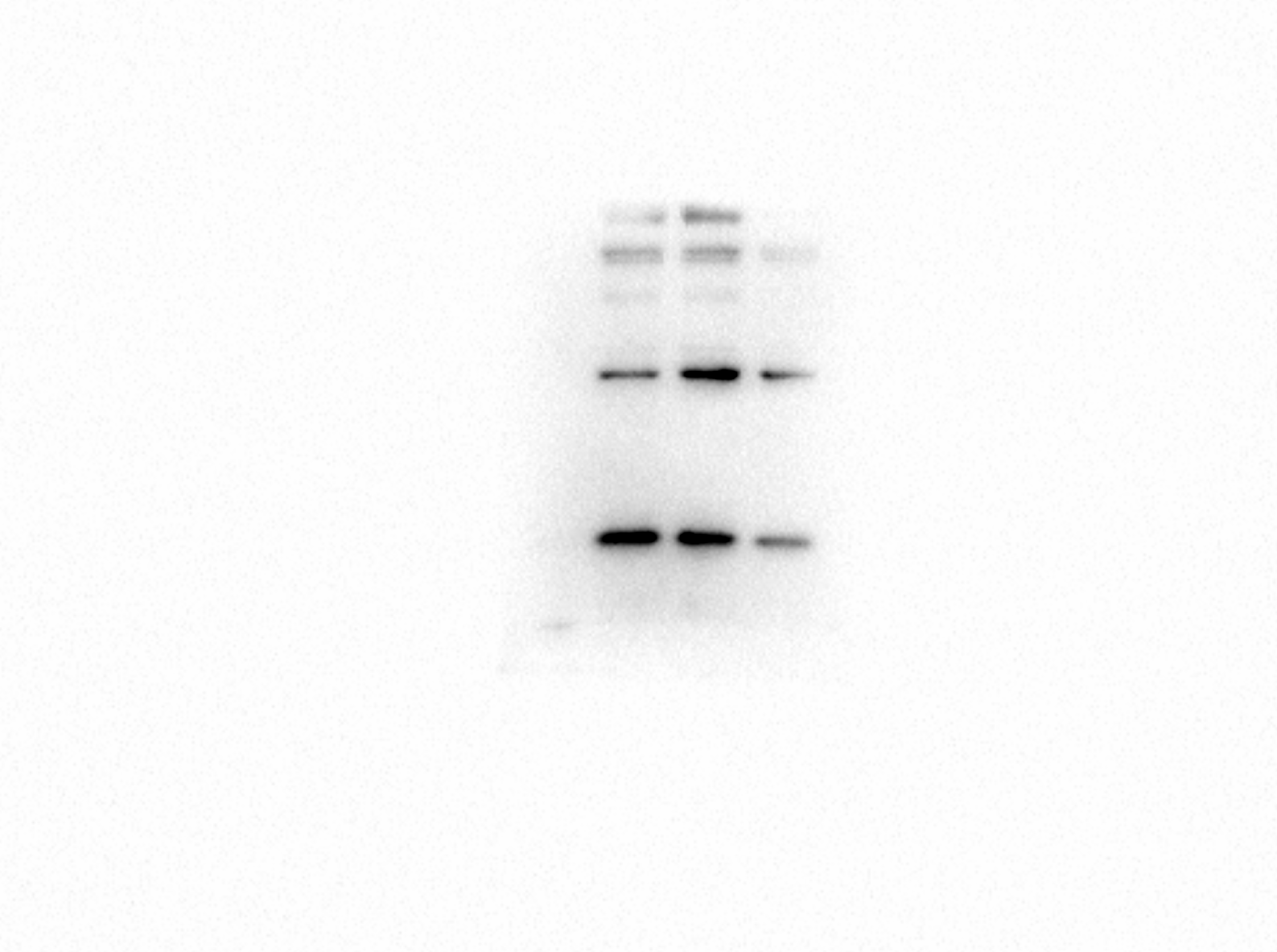

Supplement: Supplemental Information 48 [file peerj-14-21375-s048.zip › Figure 6D WB RAW OE-KLHL40 Cleaved CASPASE1/3C-cASPASE1.tif]

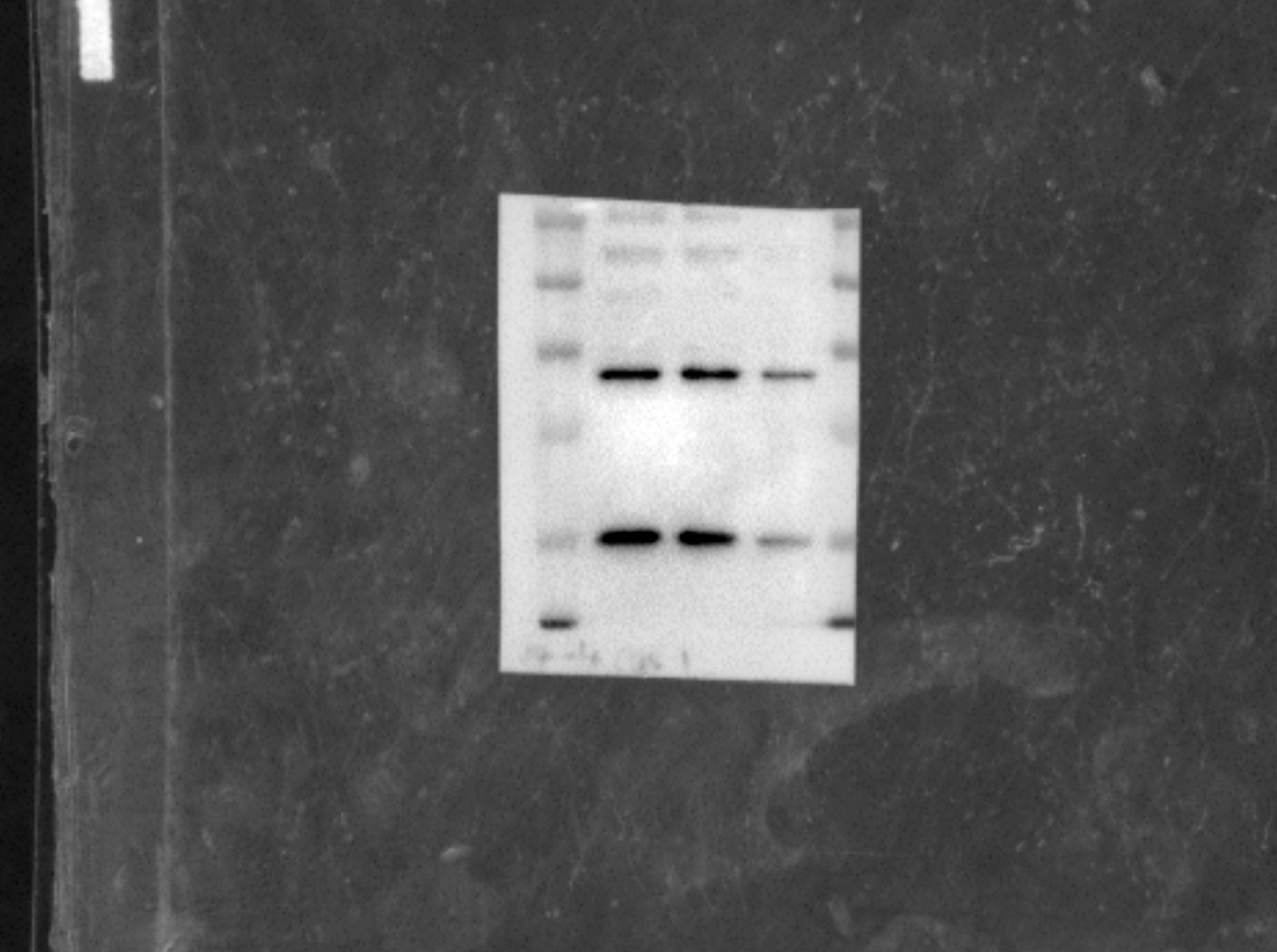

Supplement: Supplemental Information 48 [file peerj-14-21375-s048.zip › Figure 6D WB RAW OE-KLHL40 Cleaved CASPASE1/3C-caspase1+MARKER.tif]

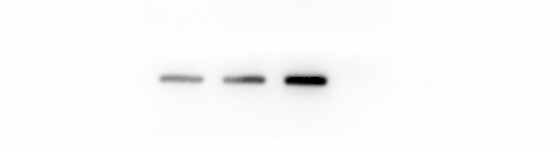

Supplement: Supplemental Information 49 [file peerj-14-21375-s049.zip › Figure 6E WB RAW SH-KLHL40 BAX BCL2/BAX/1-bax.png]

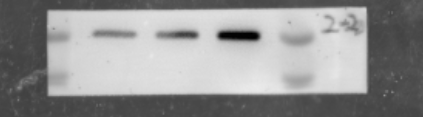

Supplement: Supplemental Information 49 [file peerj-14-21375-s049.zip › Figure 6E WB RAW SH-KLHL40 BAX BCL2/BAX/1-bax-marker.png]

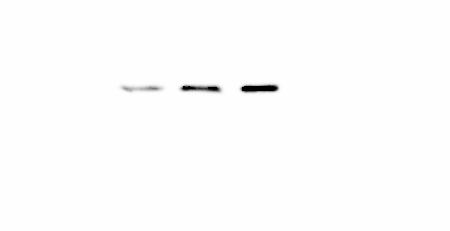

Supplement: Supplemental Information 49 [file peerj-14-21375-s049.zip › Figure 6E WB RAW SH-KLHL40 BAX BCL2/BAX/2BAX.png]

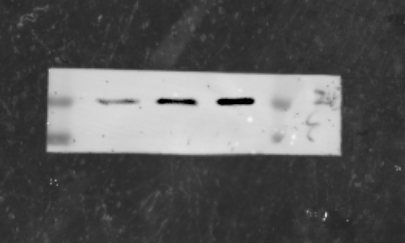

Supplement: Supplemental Information 49 [file peerj-14-21375-s049.zip › Figure 6E WB RAW SH-KLHL40 BAX BCL2/BAX/2BAX-MARKER.png]

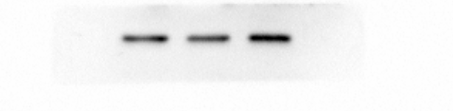

Supplement: Supplemental Information 49 [file peerj-14-21375-s049.zip › Figure 6E WB RAW SH-KLHL40 BAX BCL2/BAX/3-BAX.png]

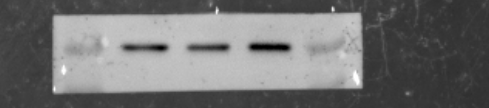

Supplement: Supplemental Information 49 [file peerj-14-21375-s049.zip › Figure 6E WB RAW SH-KLHL40 BAX BCL2/BAX/3-bax-MARKER.png]

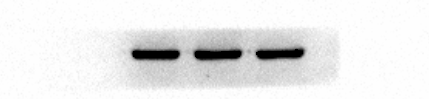

Supplement: Supplemental Information 49 [file peerj-14-21375-s049.zip › Figure 6E WB RAW SH-KLHL40 BAX BCL2/BCL2/1-ACTIN.png]

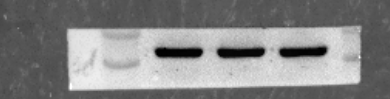

Supplement: Supplemental Information 49 [file peerj-14-21375-s049.zip › Figure 6E WB RAW SH-KLHL40 BAX BCL2/BCL2/1-ACTIN-MARKER.png]

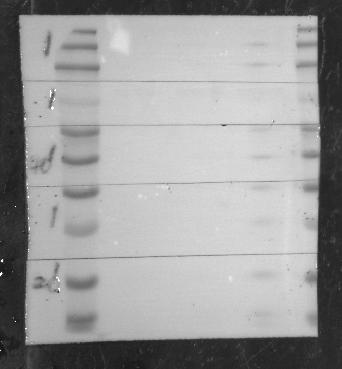

Supplement: Supplemental Information 49 [file peerj-14-21375-s049.zip › Figure 6E WB RAW SH-KLHL40 BAX BCL2/BCL2/1ALL.png]

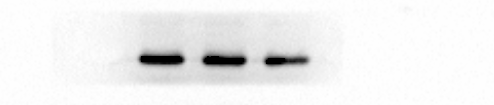

Supplement: Supplemental Information 49 [file peerj-14-21375-s049.zip › Figure 6E WB RAW SH-KLHL40 BAX BCL2/BCL2/1-bcl2.png]

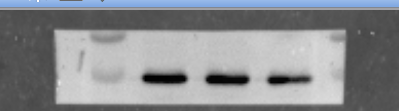

Supplement: Supplemental Information 49 [file peerj-14-21375-s049.zip › Figure 6E WB RAW SH-KLHL40 BAX BCL2/BCL2/1-bcl2-marker.png]

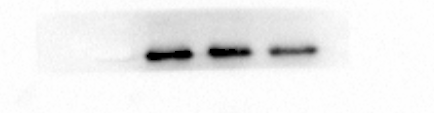

Supplement: Supplemental Information 49 [file peerj-14-21375-s049.zip › Figure 6E WB RAW SH-KLHL40 BAX BCL2/BCL2/2-bcl2.png]

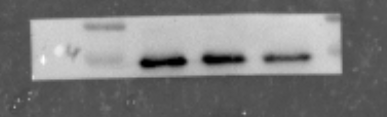

Supplement: Supplemental Information 49 [file peerj-14-21375-s049.zip › Figure 6E WB RAW SH-KLHL40 BAX BCL2/BCL2/2-bcl2-marker.png]

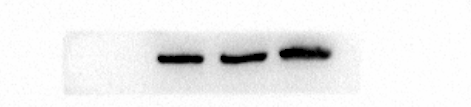

Supplement: Supplemental Information 49 [file peerj-14-21375-s049.zip › Figure 6E WB RAW SH-KLHL40 BAX BCL2/BCL2/3-ACTIN.png]

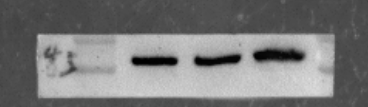

Supplement: Supplemental Information 49 [file peerj-14-21375-s049.zip › Figure 6E WB RAW SH-KLHL40 BAX BCL2/BCL2/3-ACTIN-MARKER.png]

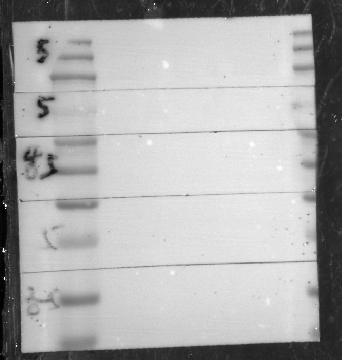

Supplement: Supplemental Information 49 [file peerj-14-21375-s049.zip › Figure 6E WB RAW SH-KLHL40 BAX BCL2/BCL2/3ALL.png]

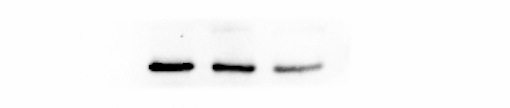

Supplement: Supplemental Information 49 [file peerj-14-21375-s049.zip › Figure 6E WB RAW SH-KLHL40 BAX BCL2/BCL2/3-bcl2.png]
