# Supplementary material for: Post-infarction KLHL40-mediated regulation of cardiac sarcomeric integrity and function
Source: PeerJ. 2026 Jun 5;14:e21375. doi: 10.7717/peerj.21375 (PMC13245431; doi:10.7717/peerj.21375)
Supplement: Supplemental Information 44 [file peerj-14-21375-s044.zip › Figure 6 Labeled Western blot.docx]

Figure 6A SH-KLHL40 NLRP3

| NLRP3 | NLRP3+MARKER | β-ACTIN | β-ACTIN+marker | ALL |
| --- | --- | --- | --- | --- |
| 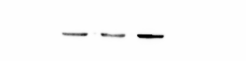 | 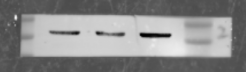 | 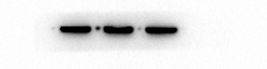 | 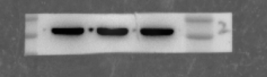 | **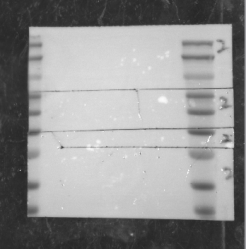** |
| 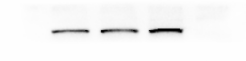 | 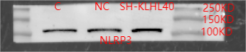 | 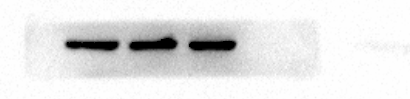 | 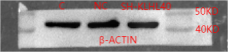 | 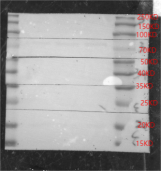 |
| 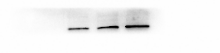 | 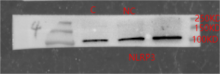 | 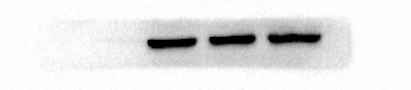 | 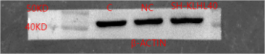 | 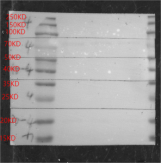 |

Figure 6B WB OE-KLHL40 NLRP3

| NLRP3 | NLRP3+MARKER | β-ACTIN | β-ACTIN+MARKER | ALL |
| --- | --- | --- | --- | --- |
| 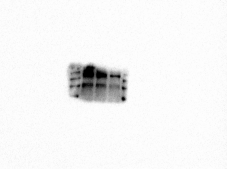 | 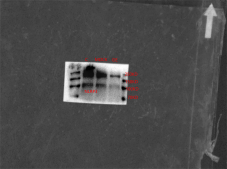 | 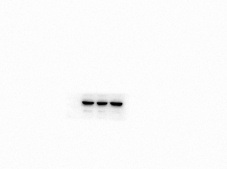 | 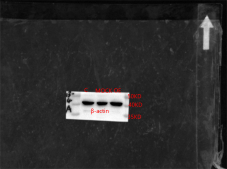 | 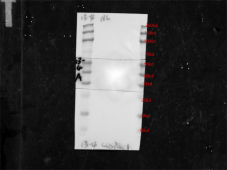 |
| 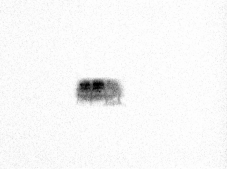 | 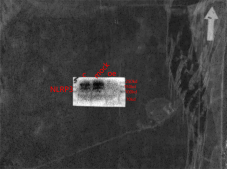 | 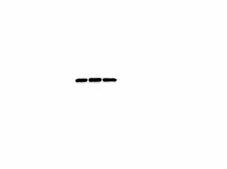 | 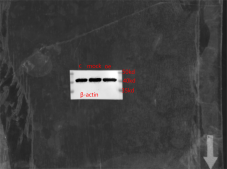 | 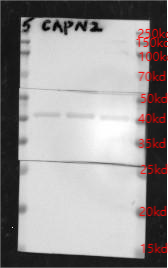 |
| 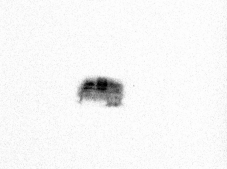 | 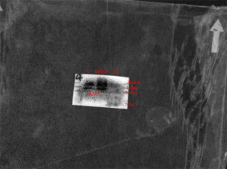 | 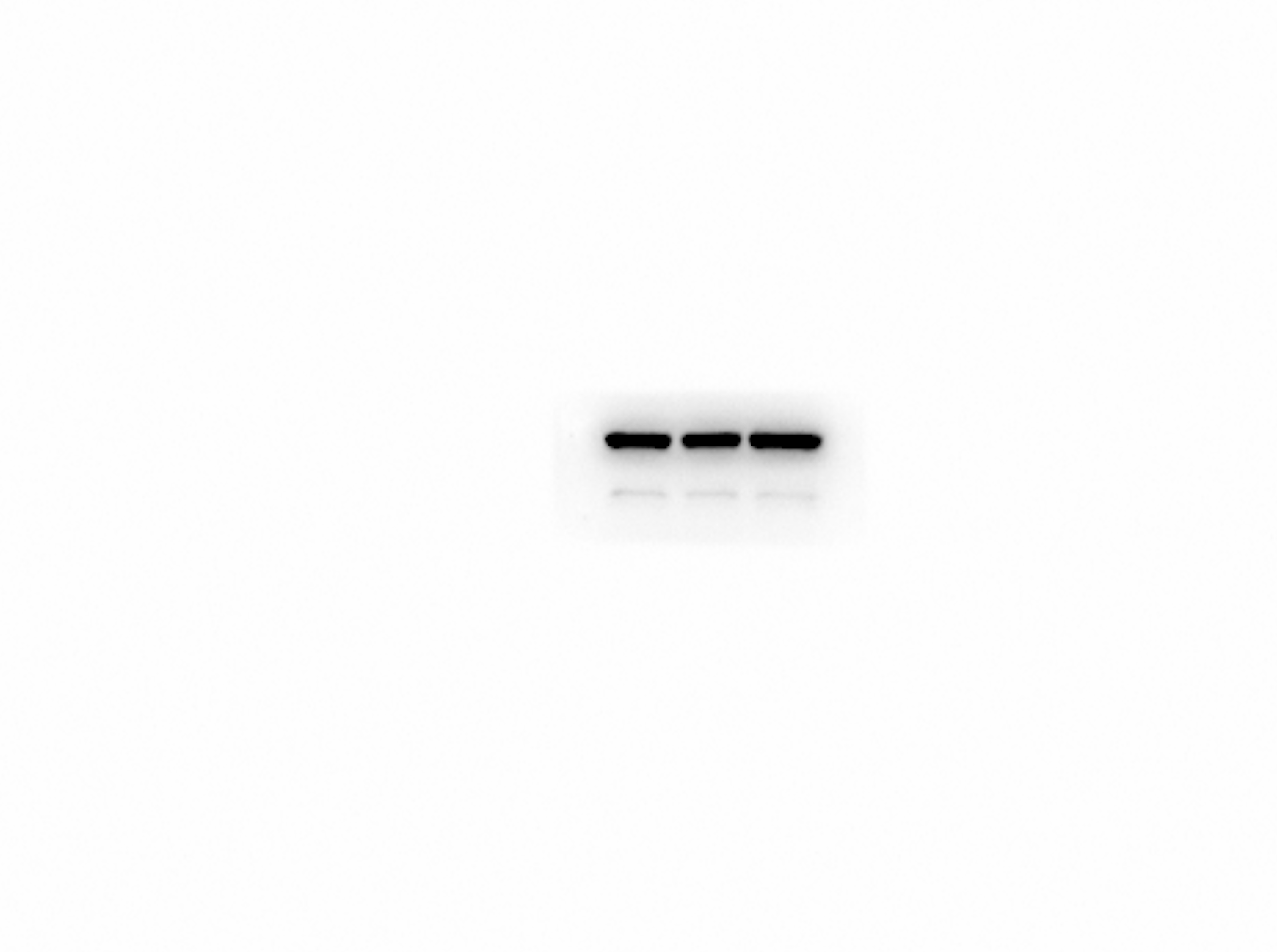 | 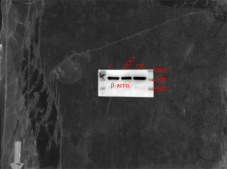 | 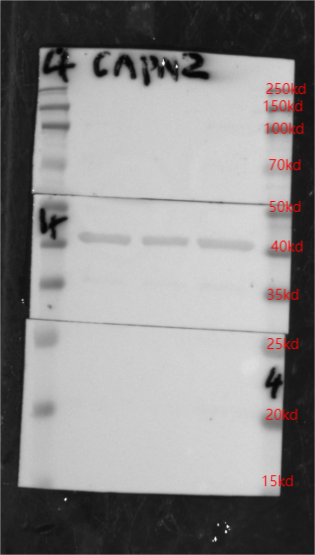 |

Figure 6C SH-KLHL40 C-caspase1

| C-Caspase1 | C-Caspase1 | β-ACTIN | β-ACTIN+marker | ALL |
| --- | --- | --- | --- | --- |
| 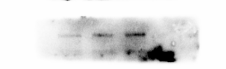 | 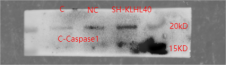 | 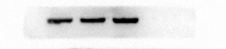 | 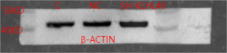 | 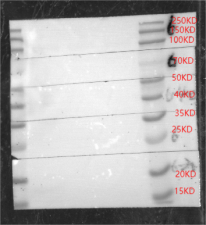 |
| 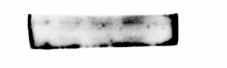 | 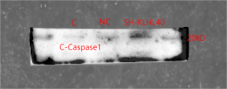 | 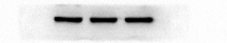 | 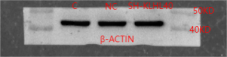 | 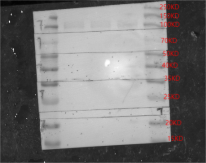 |
| 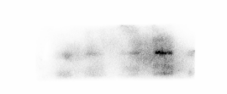 | 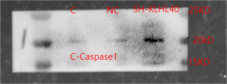 | 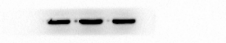 | 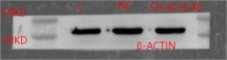 | 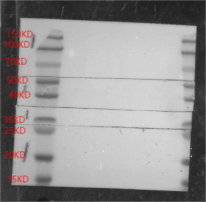 |

Figure 6D OE-KLHL40 C-caspase1

| C-CASPASE1 | C-CASPASE1+MARKER | β-ACTIN | β-ACTIN+MARKER | ALL |
| --- | --- | --- | --- | --- |
| 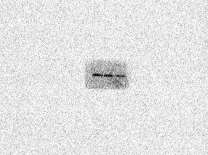 | 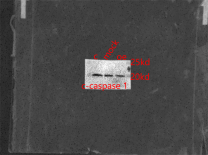 | 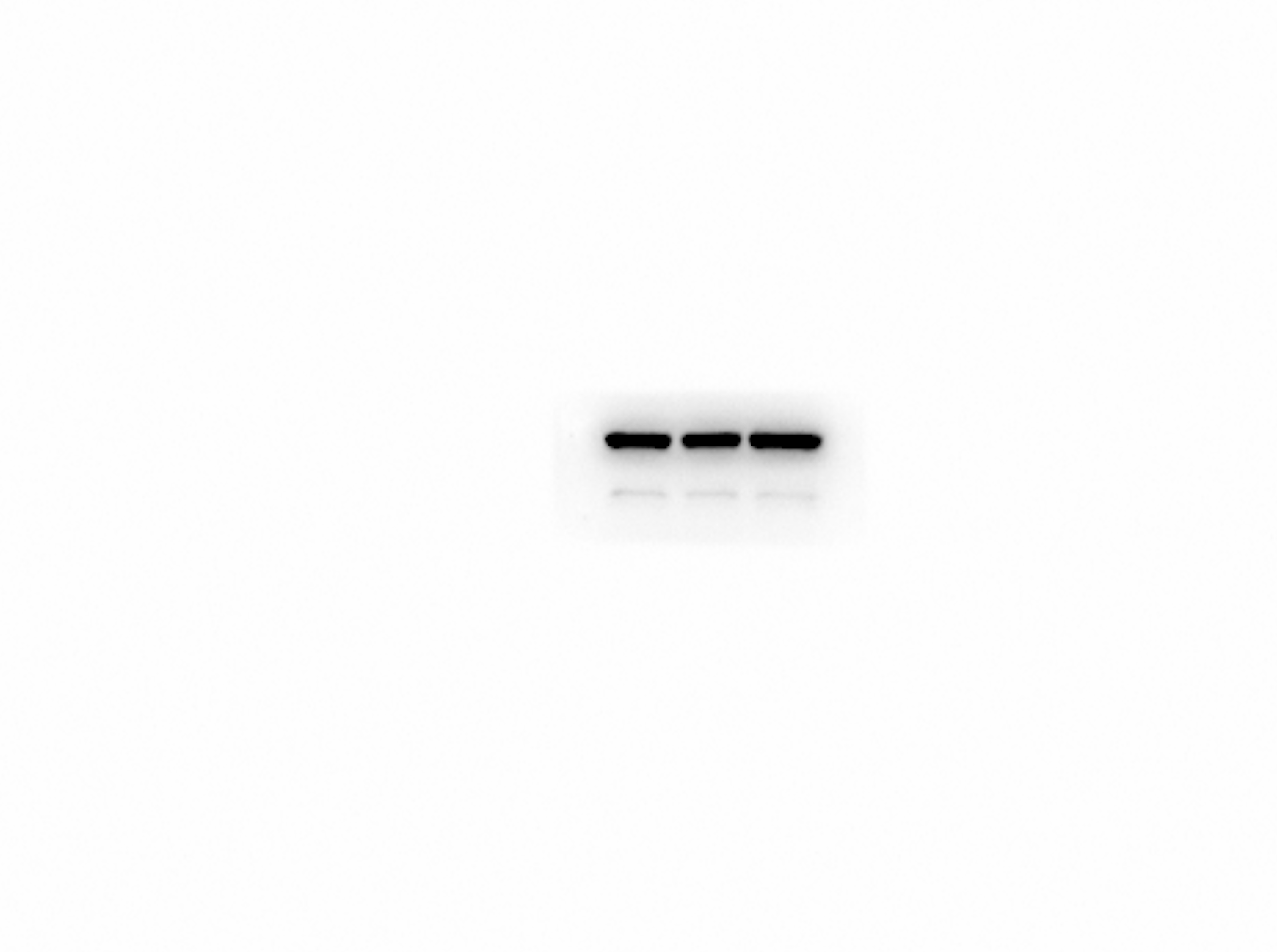 | 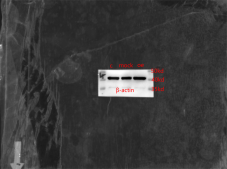 | 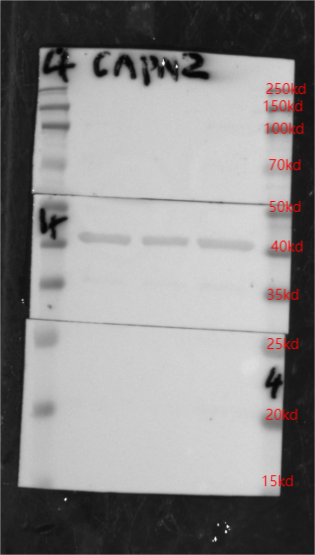 |
| 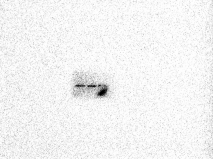 | 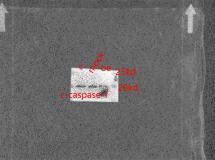 | 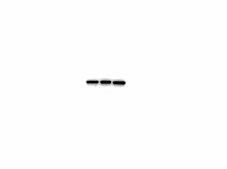 | 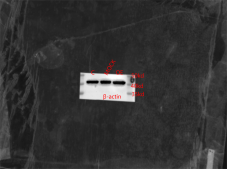 | 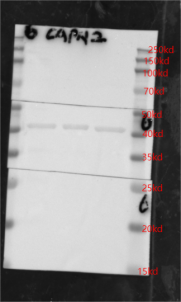 |
| 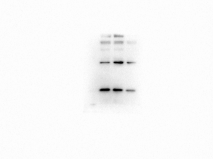 | 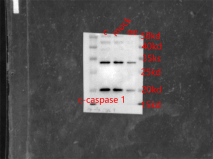 | 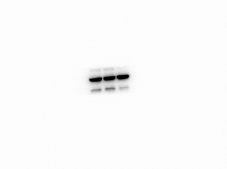 | 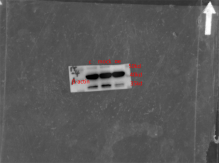 | 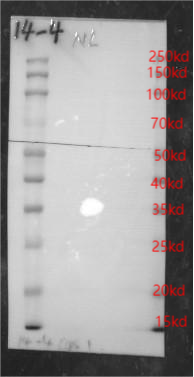 |

Figure 6E SH-KLHL40 BAX BCL2

| BAX | BAX+marker | β-ACTIN | β-ACTIN+marker | ALL |
| --- | --- | --- | --- | --- |
| 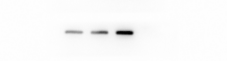 | 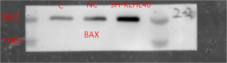 | 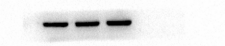 | 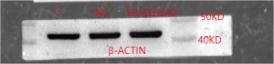 | 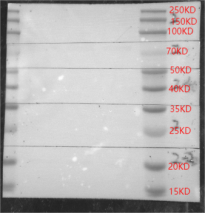 |
| 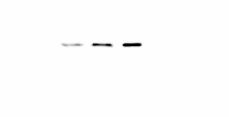 | 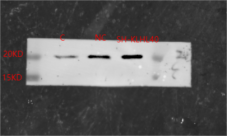 | 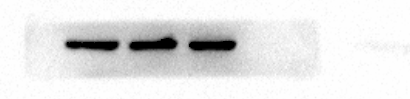 | 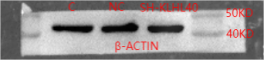 | 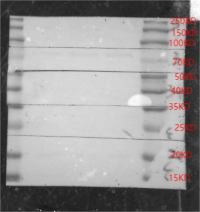 |
| 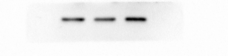 | 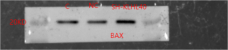 | 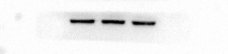 | 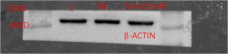 | 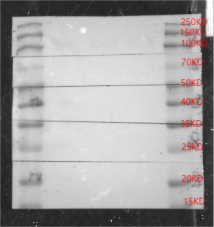 |

| BACL2 | BACL2+marker | β-ACTIN | β-ACTIN+marker | ALL |
| --- | --- | --- | --- | --- |
| 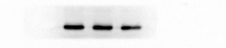 | 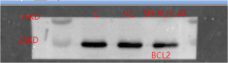 | 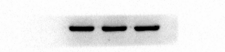 | 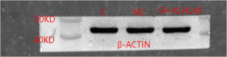 | 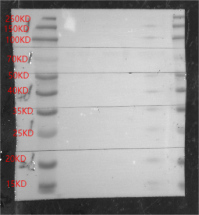 |
| 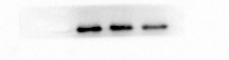 | 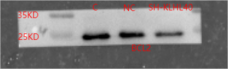 | 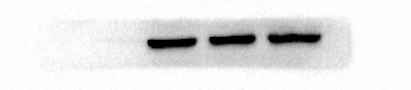 | 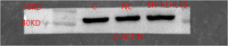 | 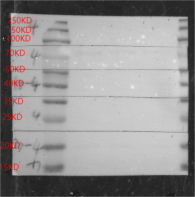 |
| 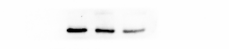 | 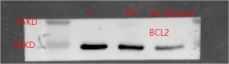 | 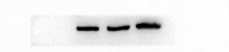 | 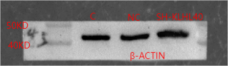 | 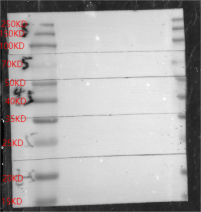 |

Figure 6E SH-KLHL40 BAX BCL2

| BAX | BAX+MARKER | β-ACTIN | β-ACTIN+MARKER | ALL |
| --- | --- | --- | --- | --- |
| 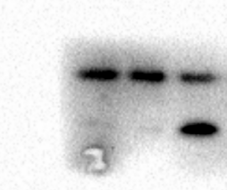 | 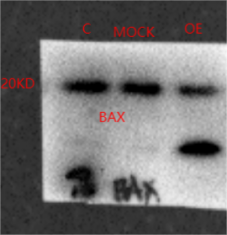 | 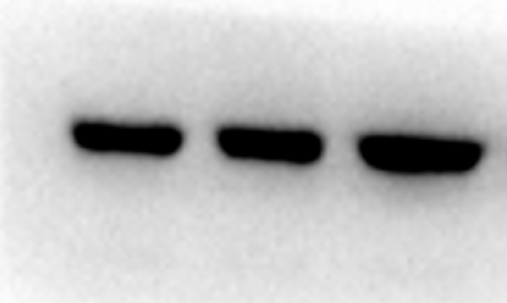 | 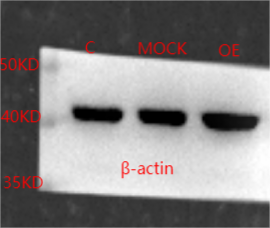 | 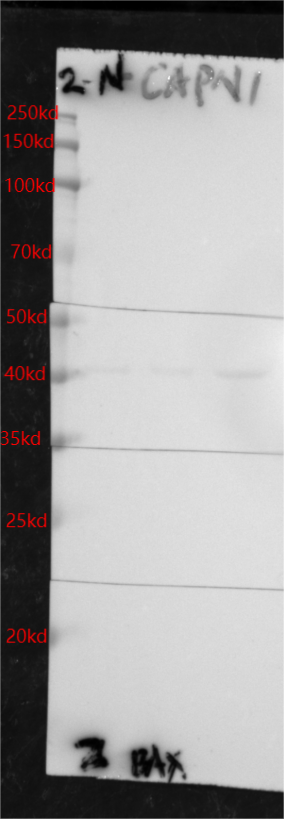 |
| 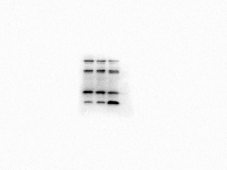 | 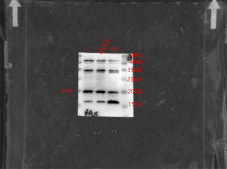 | 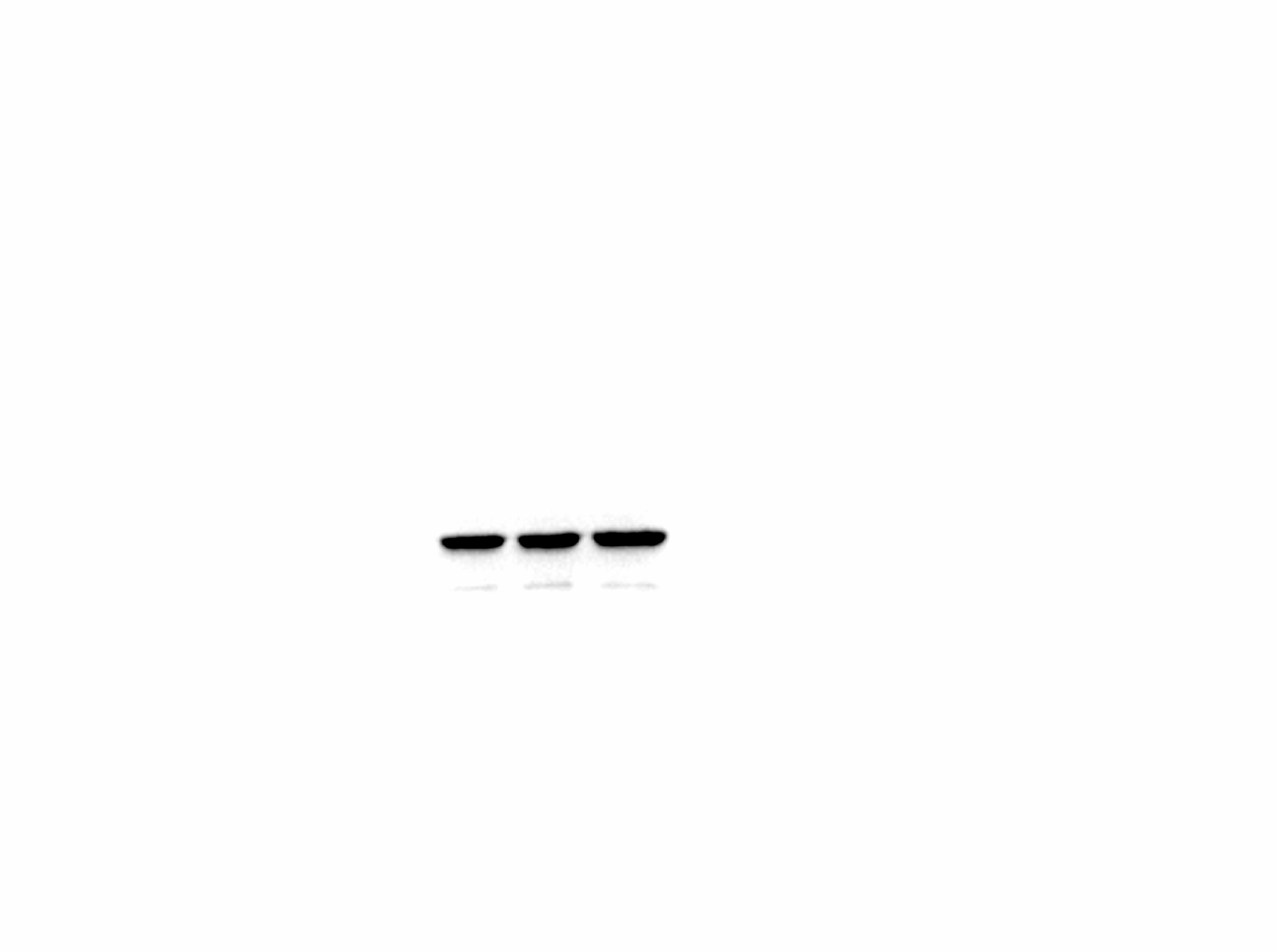 | 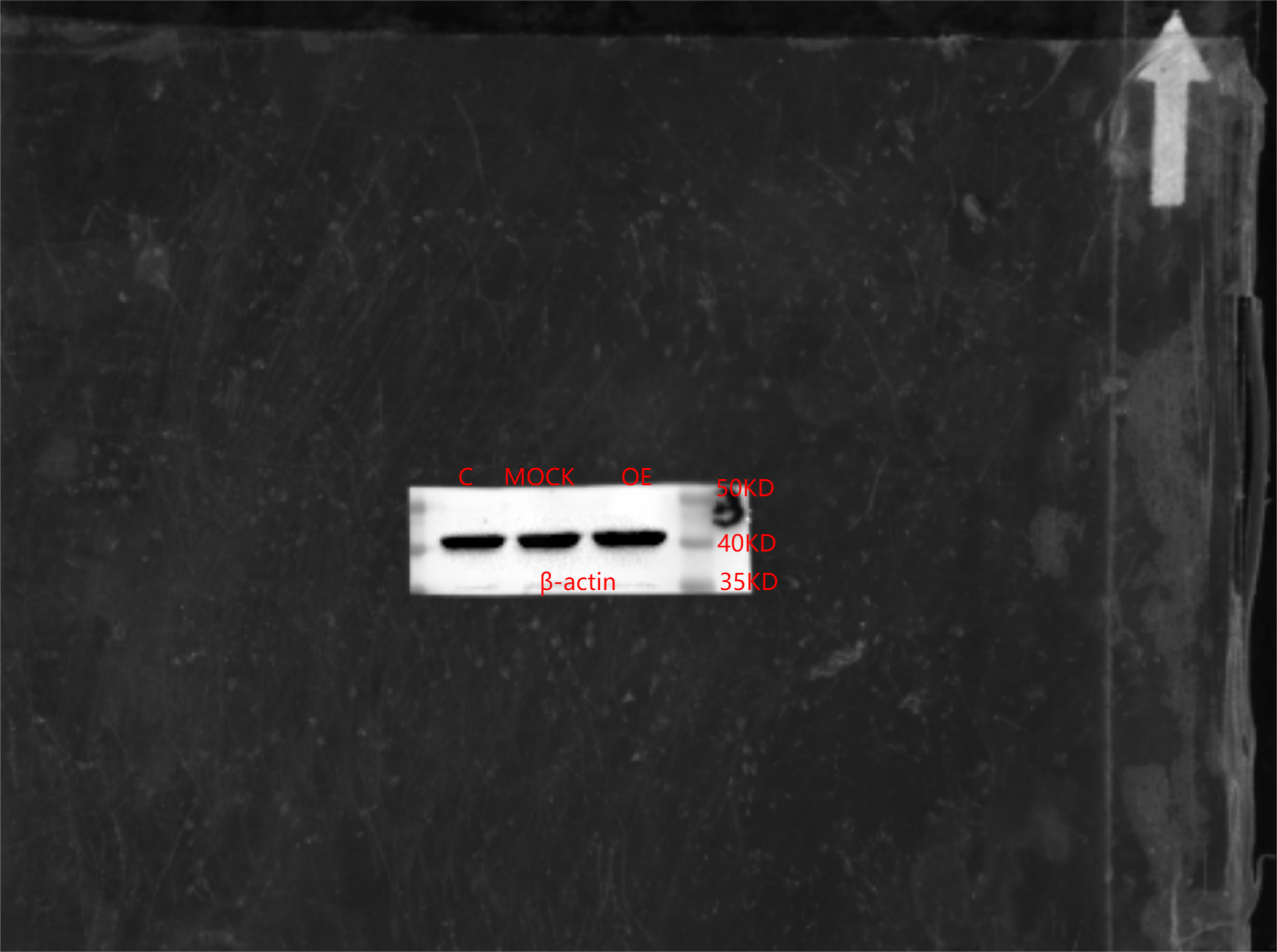 | 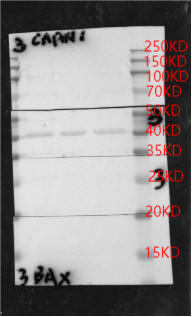 |
|  |  |  |  |  |

| BCL2 | BCL2+MARKER | β-ACTIN | β-ACTIN+MARKER | ALL |
| --- | --- | --- | --- | --- |
|  |  |  |  |  |
|  |  |  |  |  |
|  |  |  |  |  |
