# Supplementary figures and images for: Post-infarction KLHL40-mediated regulation of cardiac sarcomeric integrity and function (part 5 of 5)
Source: PeerJ. 2026 Jun 5;14:e21375. doi: 10.7717/peerj.21375 (PMC13245431; doi:10.7717/peerj.21375)

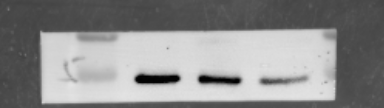

Supplement: Supplemental Information 49 [file peerj-14-21375-s049.zip › Figure 6E WB RAW SH-KLHL40 BAX BCL2/BCL2/3-bcl2-marker.png]

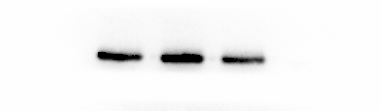

Supplement: Supplemental Information 49 [file peerj-14-21375-s049.zip › Figure 6E WB RAW SH-KLHL40 BAX BCL2/BCL2/4-bacl2.png]

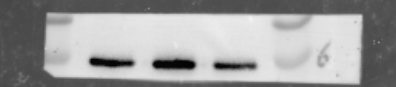

Supplement: Supplemental Information 49 [file peerj-14-21375-s049.zip › Figure 6E WB RAW SH-KLHL40 BAX BCL2/BCL2/4-bcl2-marker.png]

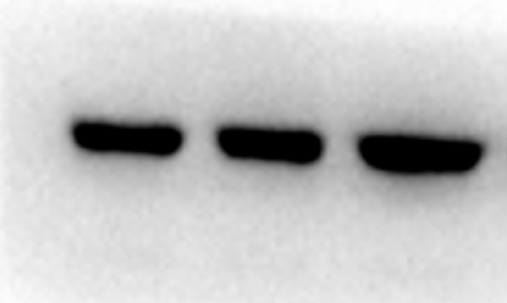

Supplement: Supplemental Information 50 [file peerj-14-21375-s050.zip › Figure 6F WB RAW OE-KLHL40 BAX BCL2/BAX/1ACTIN.png]

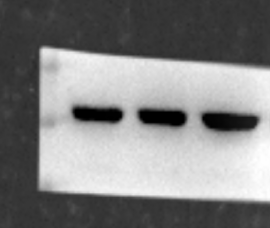

Supplement: Supplemental Information 50 [file peerj-14-21375-s050.zip › Figure 6F WB RAW OE-KLHL40 BAX BCL2/BAX/1ACTIN-MARKER.png]

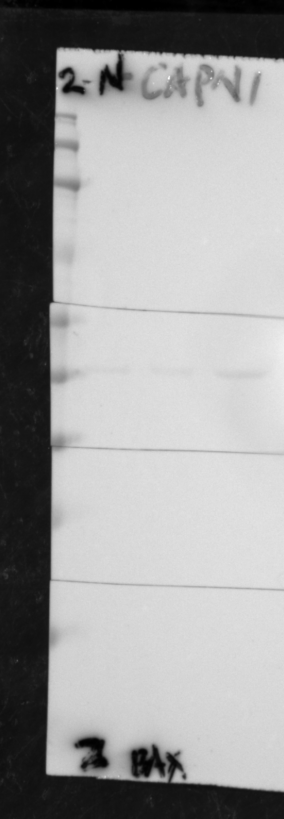

Supplement: Supplemental Information 50 [file peerj-14-21375-s050.zip › Figure 6F WB RAW OE-KLHL40 BAX BCL2/BAX/1ALL.png]

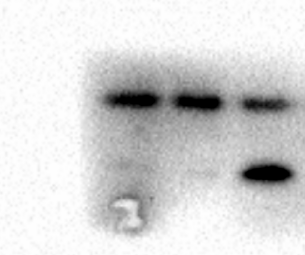

Supplement: Supplemental Information 50 [file peerj-14-21375-s050.zip › Figure 6F WB RAW OE-KLHL40 BAX BCL2/BAX/1BAX.png]

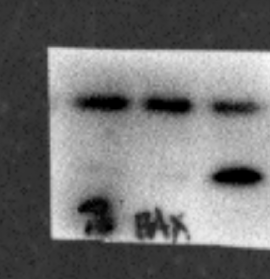

Supplement: Supplemental Information 50 [file peerj-14-21375-s050.zip › Figure 6F WB RAW OE-KLHL40 BAX BCL2/BAX/1BAX+MARKER.png]

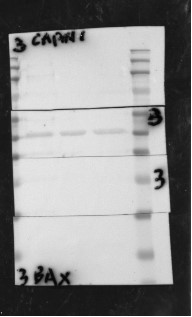

Supplement: Supplemental Information 50 [file peerj-14-21375-s050.zip › Figure 6F WB RAW OE-KLHL40 BAX BCL2/BAX/2ALL.jpg]

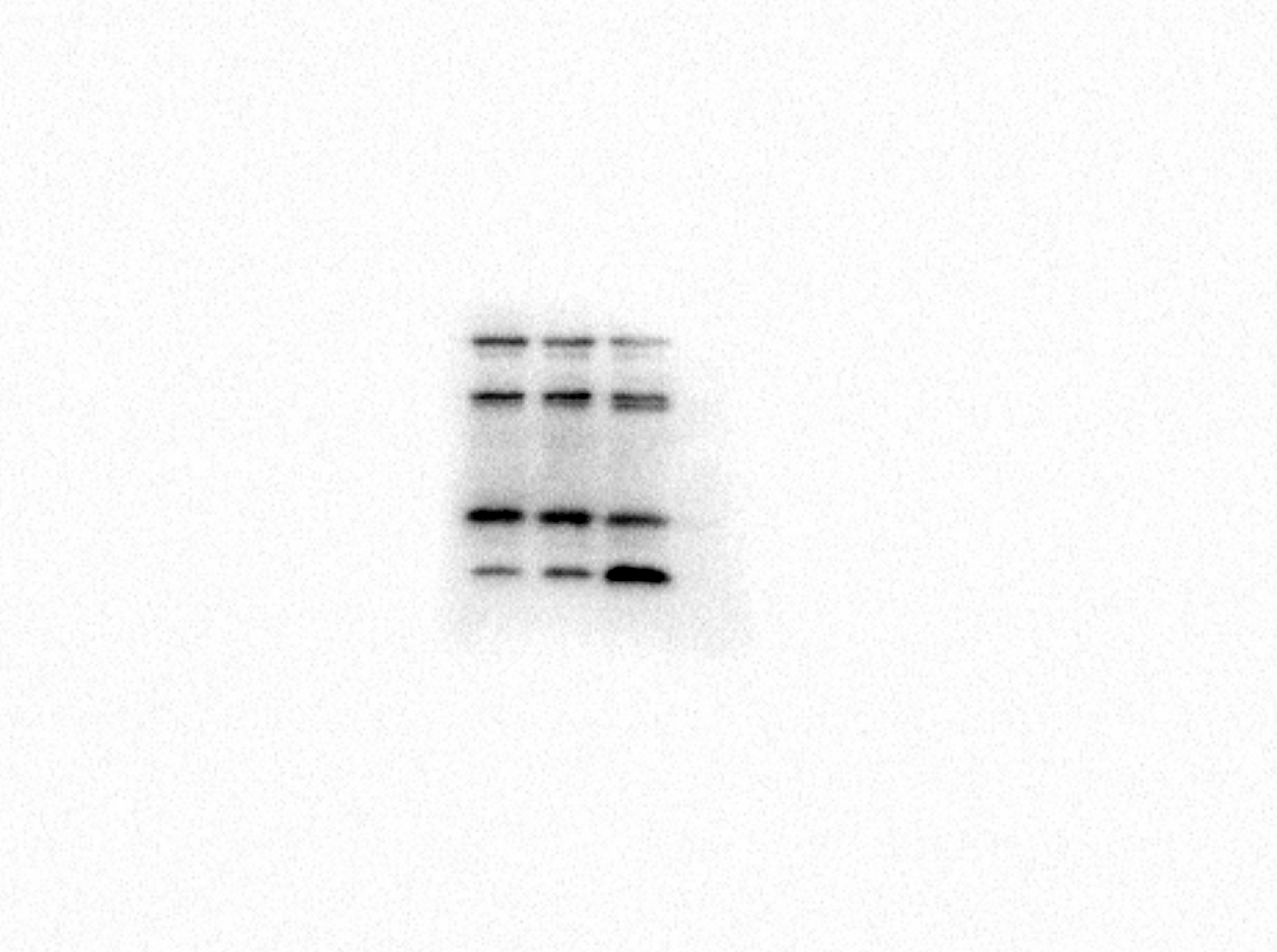

Supplement: Supplemental Information 50 [file peerj-14-21375-s050.zip › Figure 6F WB RAW OE-KLHL40 BAX BCL2/BAX/2BAX.tif]

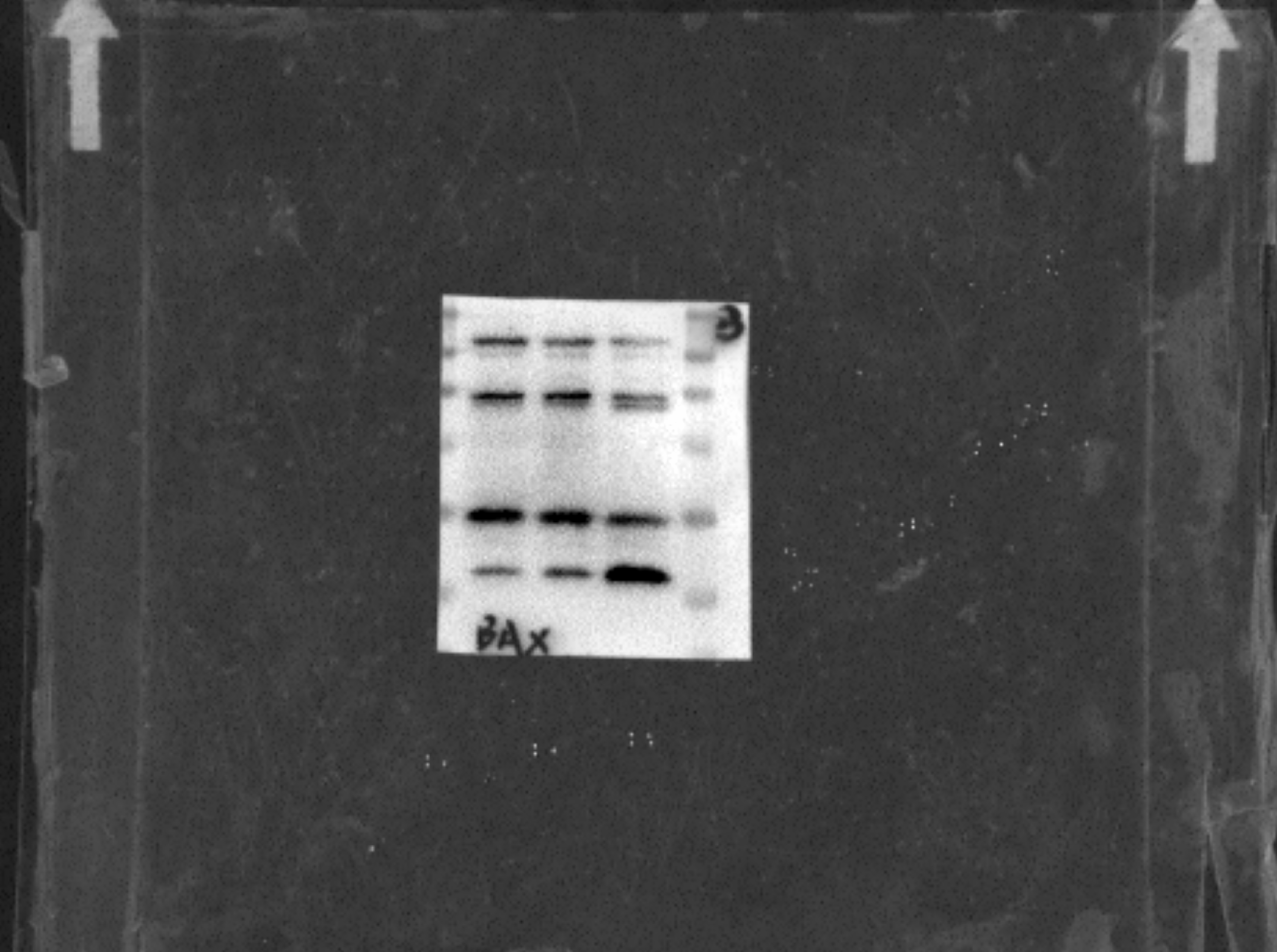

Supplement: Supplemental Information 50 [file peerj-14-21375-s050.zip › Figure 6F WB RAW OE-KLHL40 BAX BCL2/BAX/2BAX+MARKER.tif]

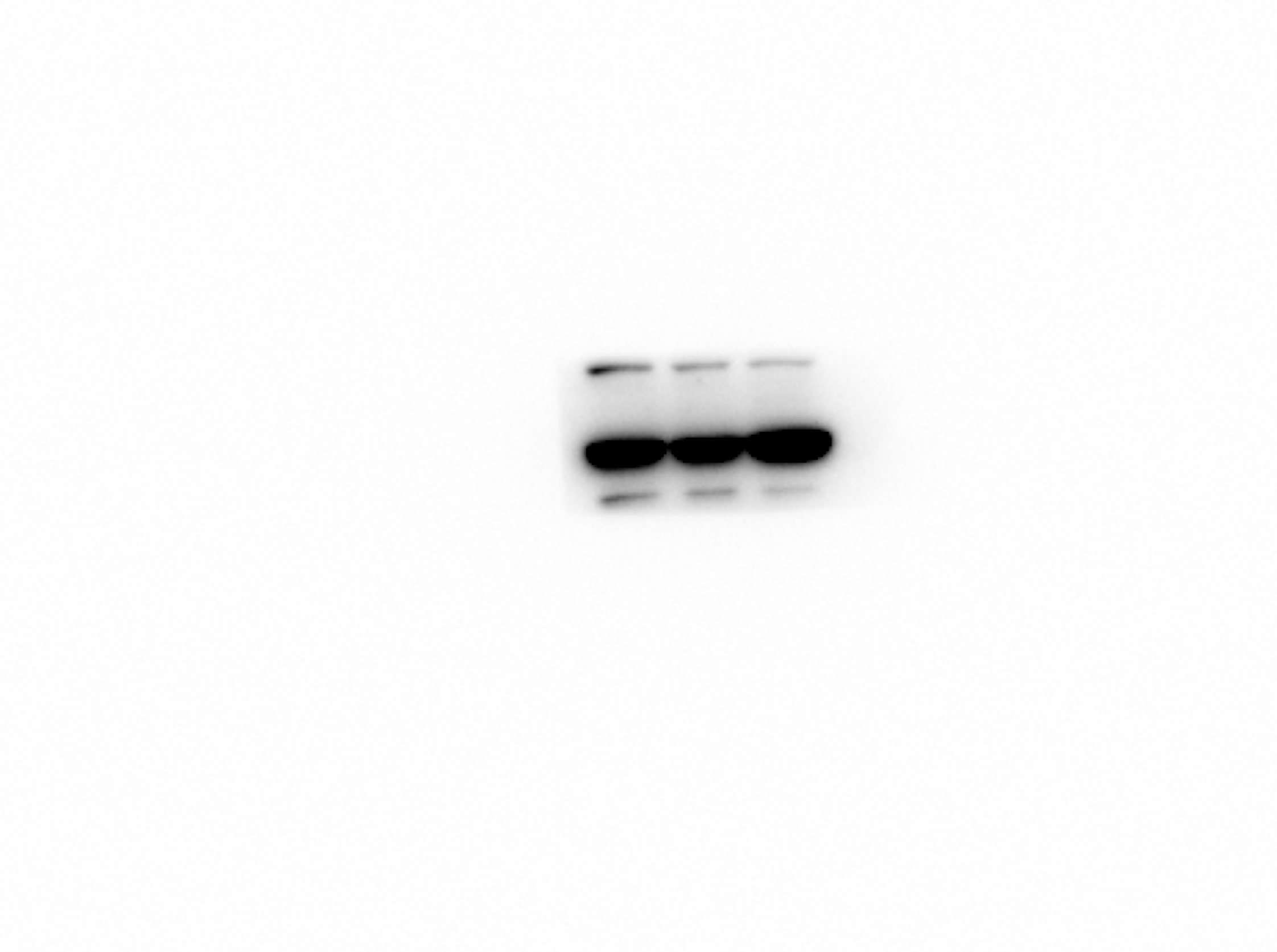

Supplement: Supplemental Information 50 [file peerj-14-21375-s050.zip › Figure 6F WB RAW OE-KLHL40 BAX BCL2/BAX/3ACTIN.tif]

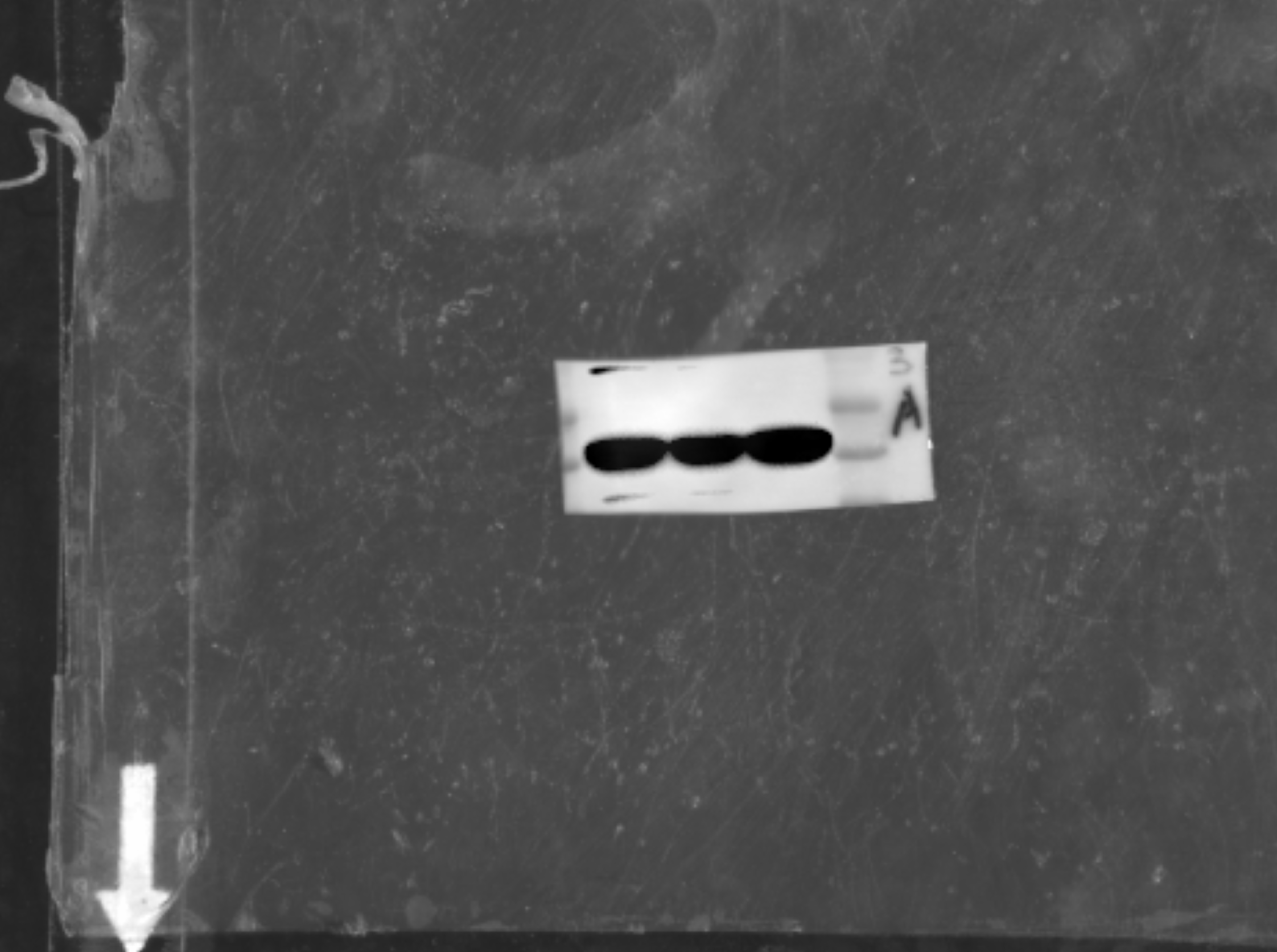

Supplement: Supplemental Information 50 [file peerj-14-21375-s050.zip › Figure 6F WB RAW OE-KLHL40 BAX BCL2/BAX/3ACTIN+MARKER.tif]

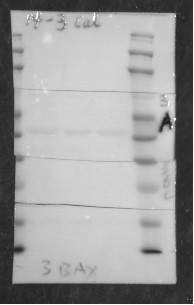

Supplement: Supplemental Information 50 [file peerj-14-21375-s050.zip › Figure 6F WB RAW OE-KLHL40 BAX BCL2/BAX/3ALL.jpg]

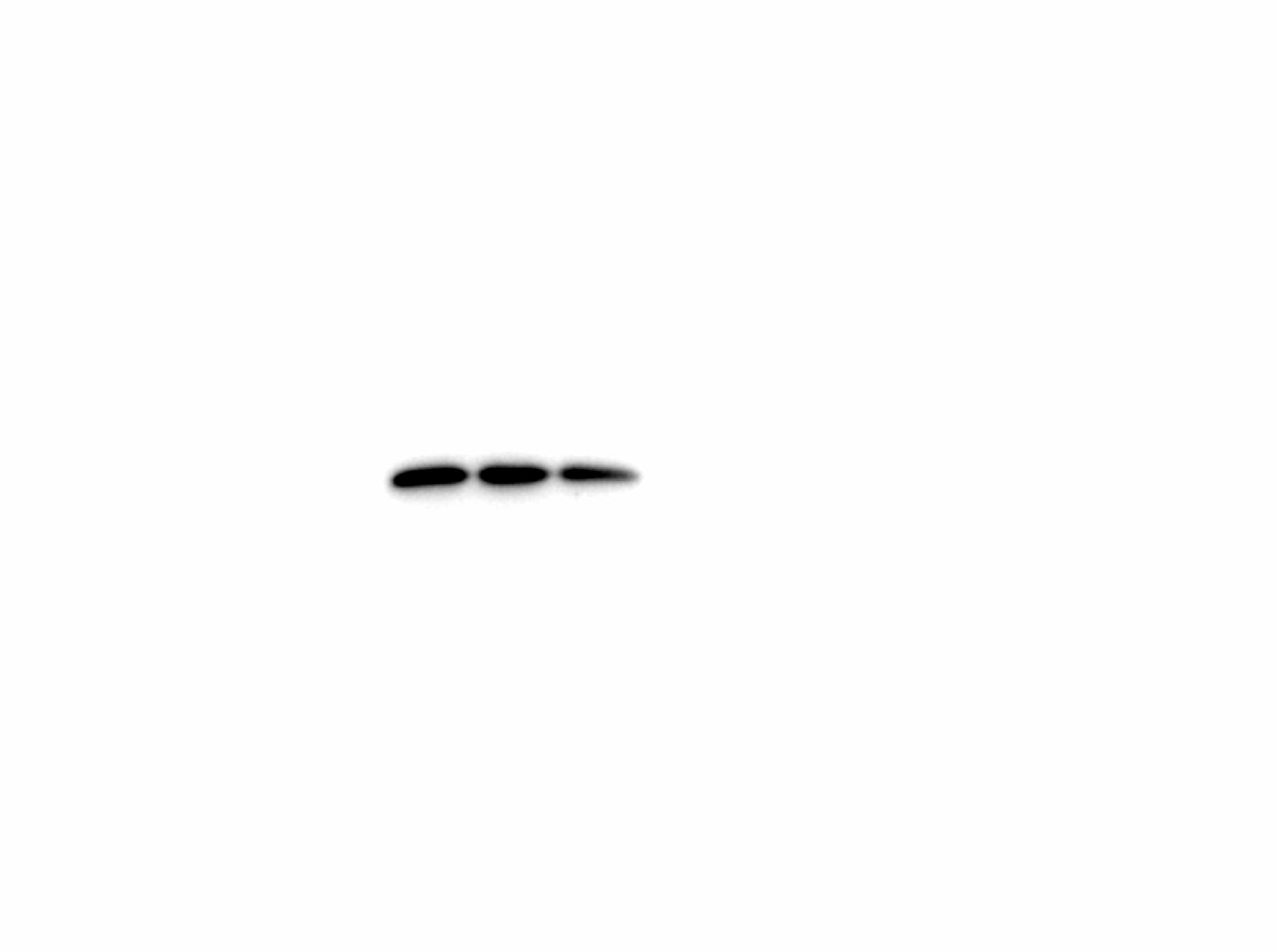

Supplement: Supplemental Information 50 [file peerj-14-21375-s050.zip › Figure 6F WB RAW OE-KLHL40 BAX BCL2/BAX/3BAX.tif]

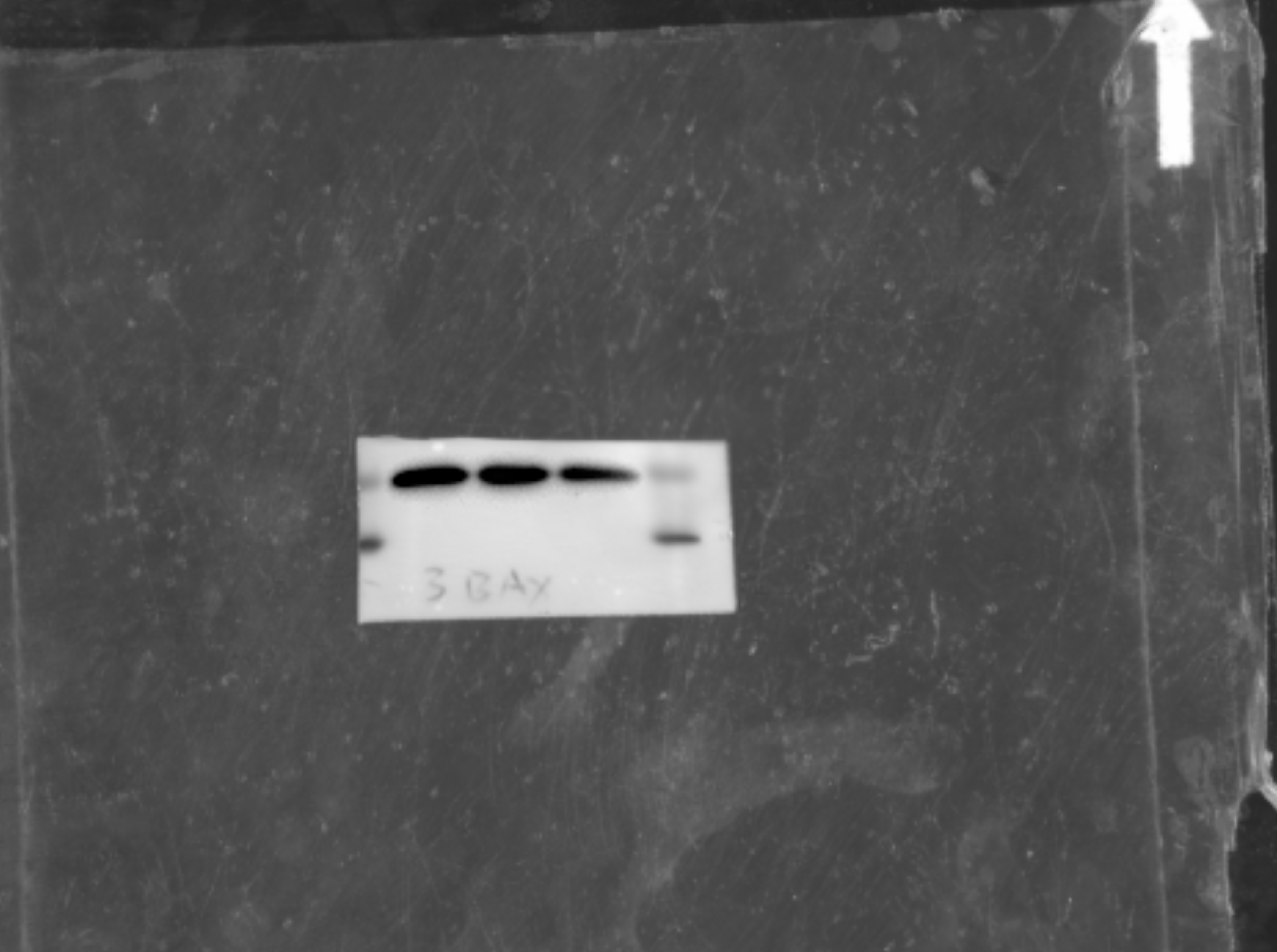

Supplement: Supplemental Information 50 [file peerj-14-21375-s050.zip › Figure 6F WB RAW OE-KLHL40 BAX BCL2/BAX/3BAX+MARKER.tif]

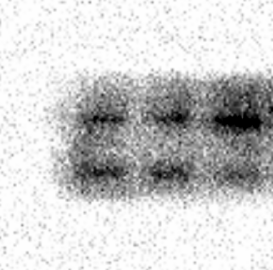

Supplement: Supplemental Information 50 [file peerj-14-21375-s050.zip › Figure 6F WB RAW OE-KLHL40 BAX BCL2/BCL2/1BCL2.png]

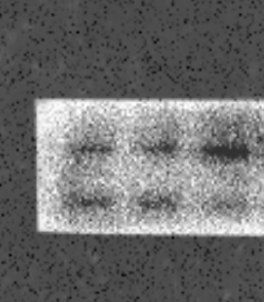

Supplement: Supplemental Information 50 [file peerj-14-21375-s050.zip › Figure 6F WB RAW OE-KLHL40 BAX BCL2/BCL2/1BCL2+MARKER.png]

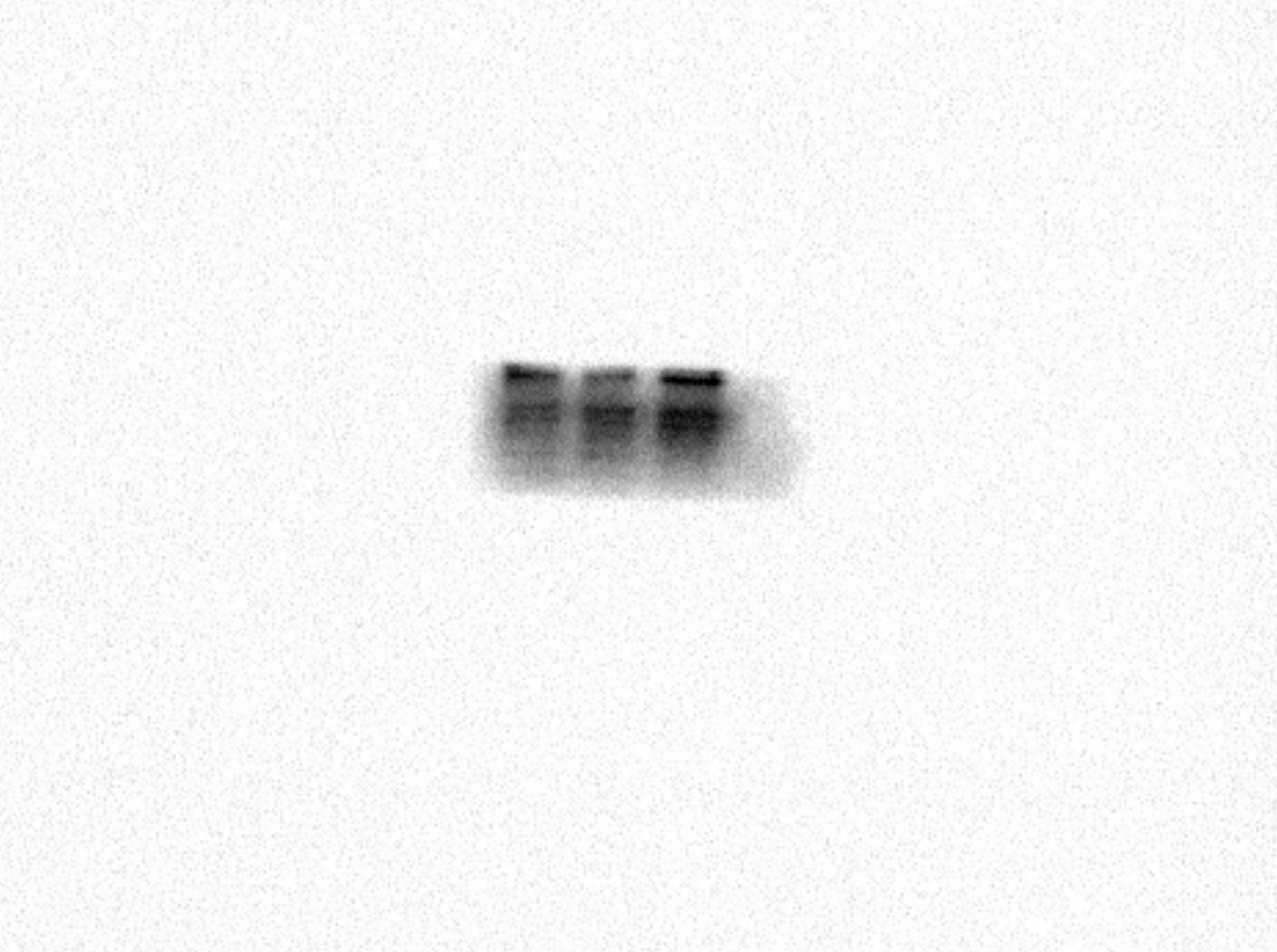

Supplement: Supplemental Information 50 [file peerj-14-21375-s050.zip › Figure 6F WB RAW OE-KLHL40 BAX BCL2/BCL2/2BCL2.tif]

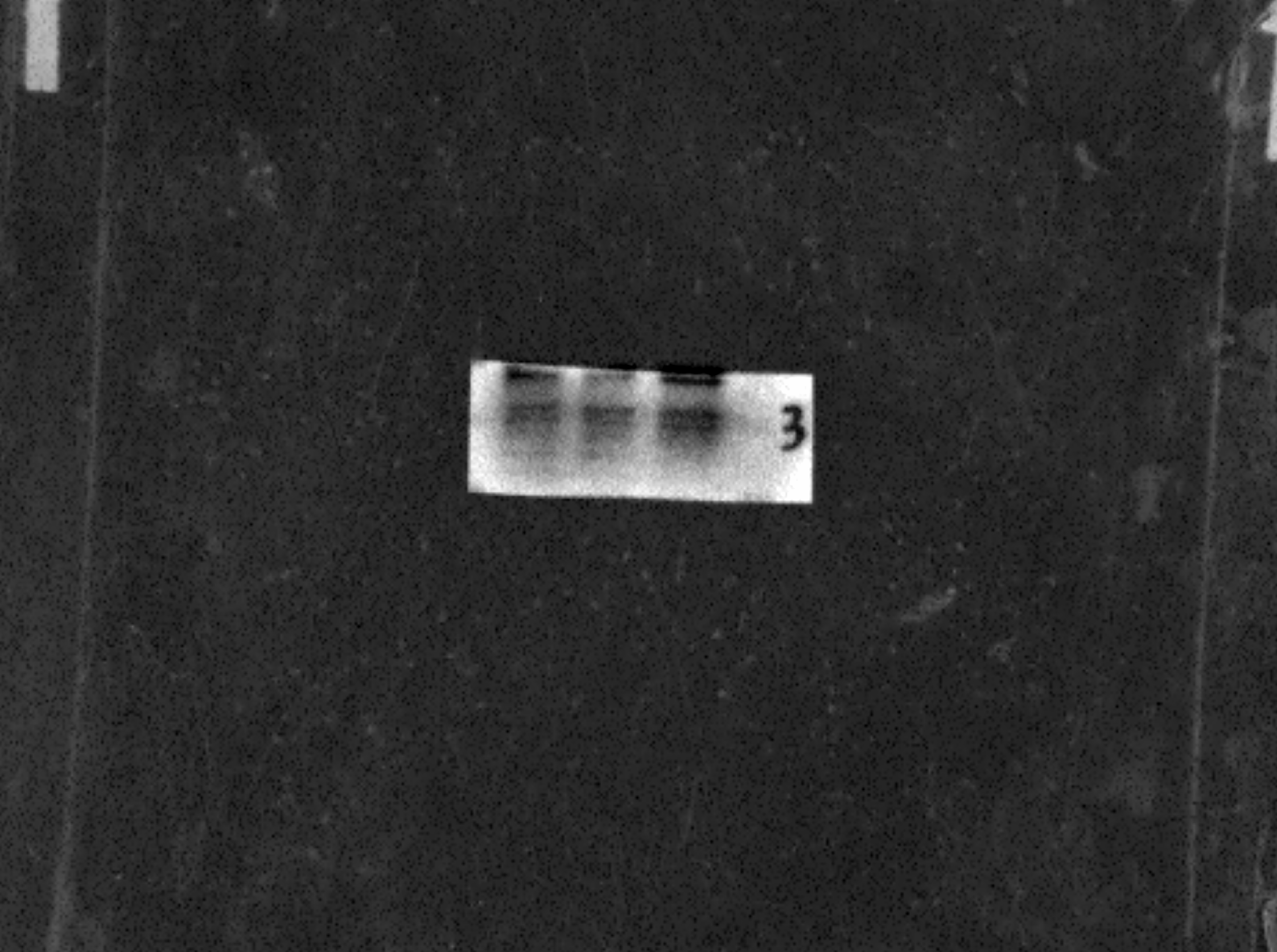

Supplement: Supplemental Information 50 [file peerj-14-21375-s050.zip › Figure 6F WB RAW OE-KLHL40 BAX BCL2/BCL2/2BCL2+MARKER.tif]

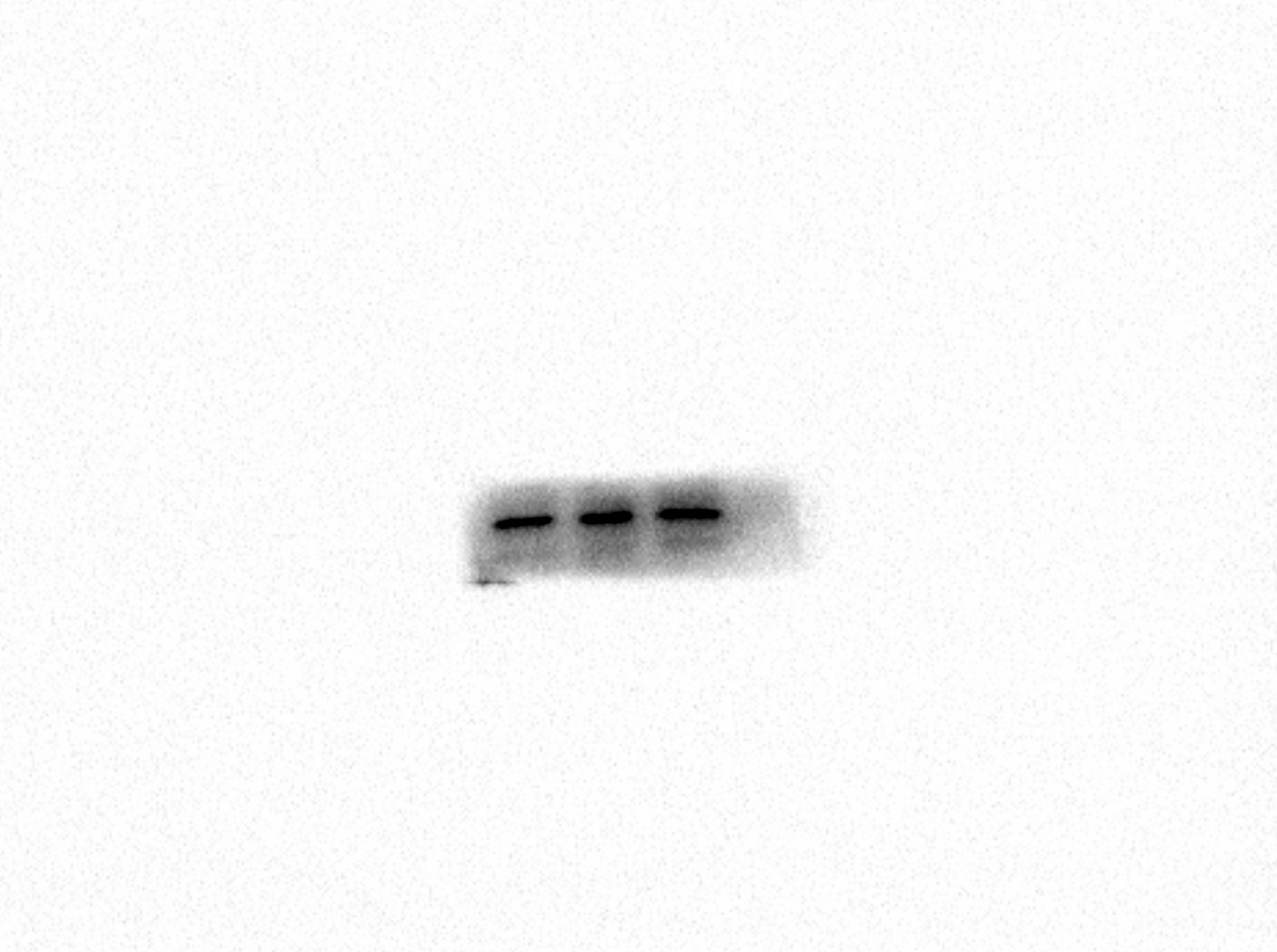

Supplement: Supplemental Information 50 [file peerj-14-21375-s050.zip › Figure 6F WB RAW OE-KLHL40 BAX BCL2/BCL2/3BCL2..tif]

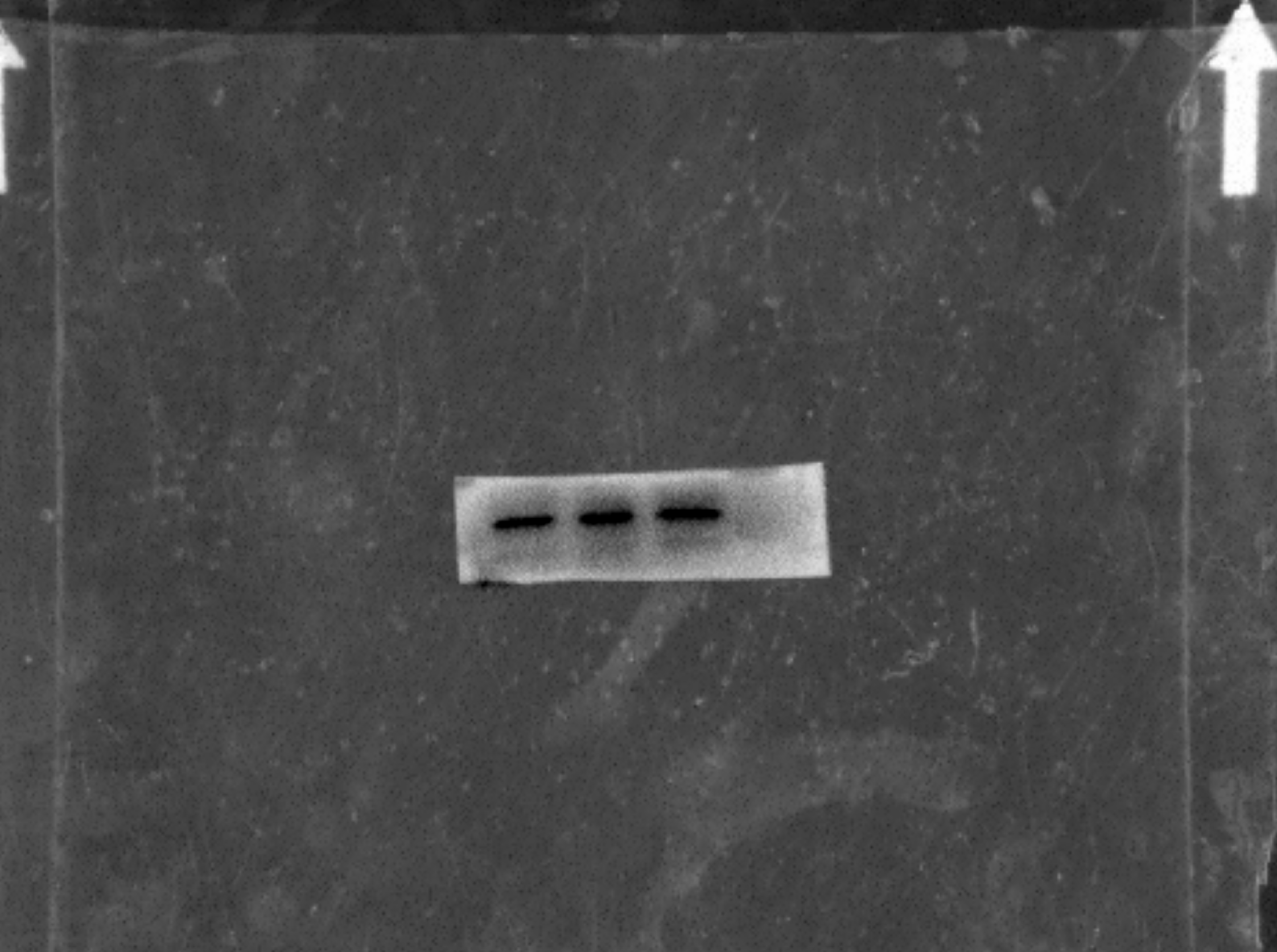

Supplement: Supplemental Information 50 [file peerj-14-21375-s050.zip › Figure 6F WB RAW OE-KLHL40 BAX BCL2/BCL2/3BCL2+MARKER.tif]

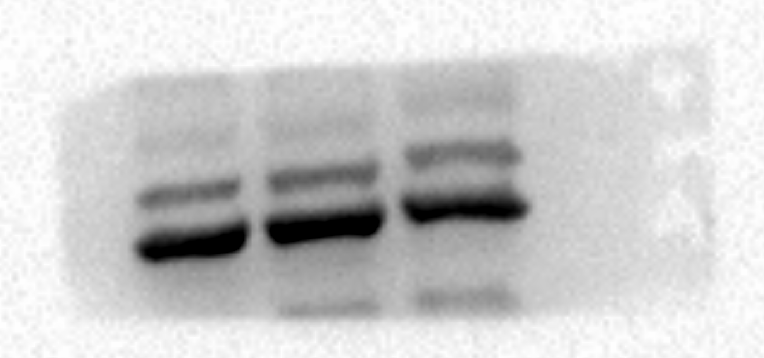

Supplement: Supplemental Information 52 [file peerj-14-21375-s052.zip › Figure 7E WB RAW MG132 KLHL40/1- ACTB.tif]

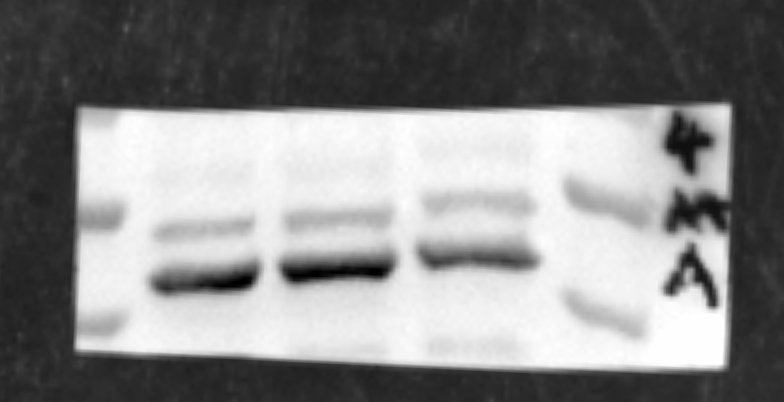

Supplement: Supplemental Information 52 [file peerj-14-21375-s052.zip › Figure 7E WB RAW MG132 KLHL40/1- ACTB+MARK.tif]

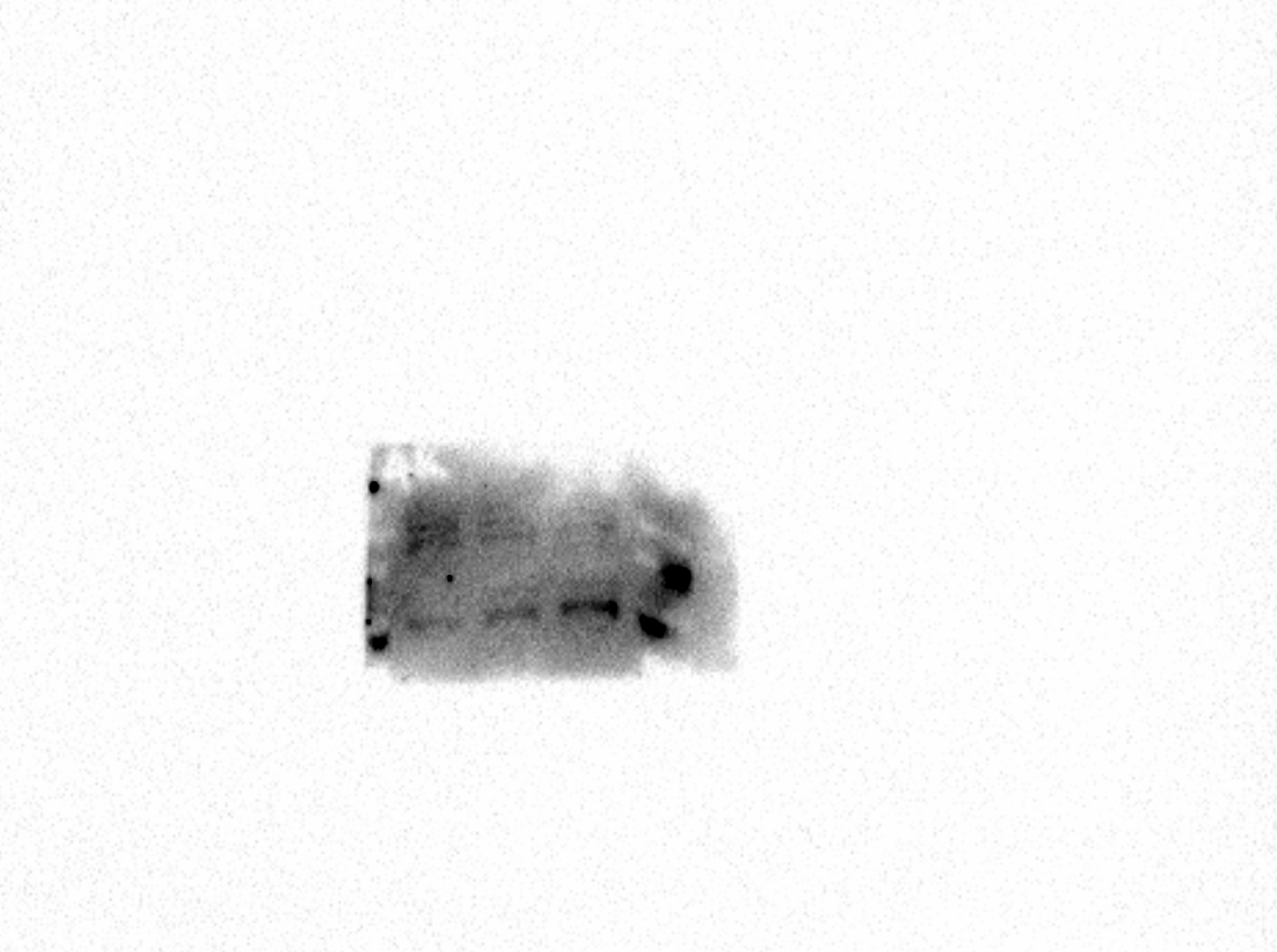

Supplement: Supplemental Information 52 [file peerj-14-21375-s052.zip › Figure 7E WB RAW MG132 KLHL40/1-1 KLHL40.tif]

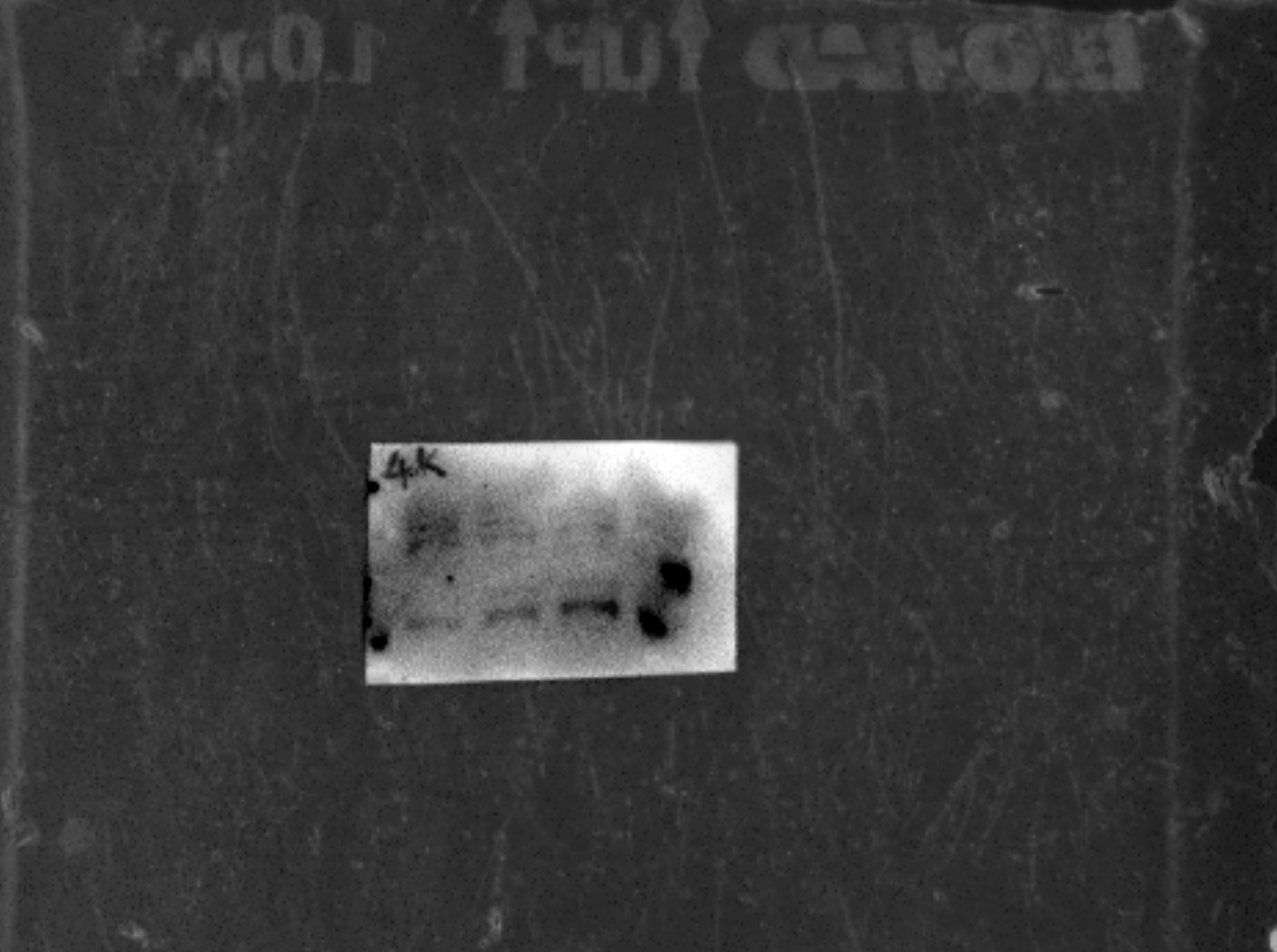

Supplement: Supplemental Information 52 [file peerj-14-21375-s052.zip › Figure 7E WB RAW MG132 KLHL40/1-2 KLHL40.tif]

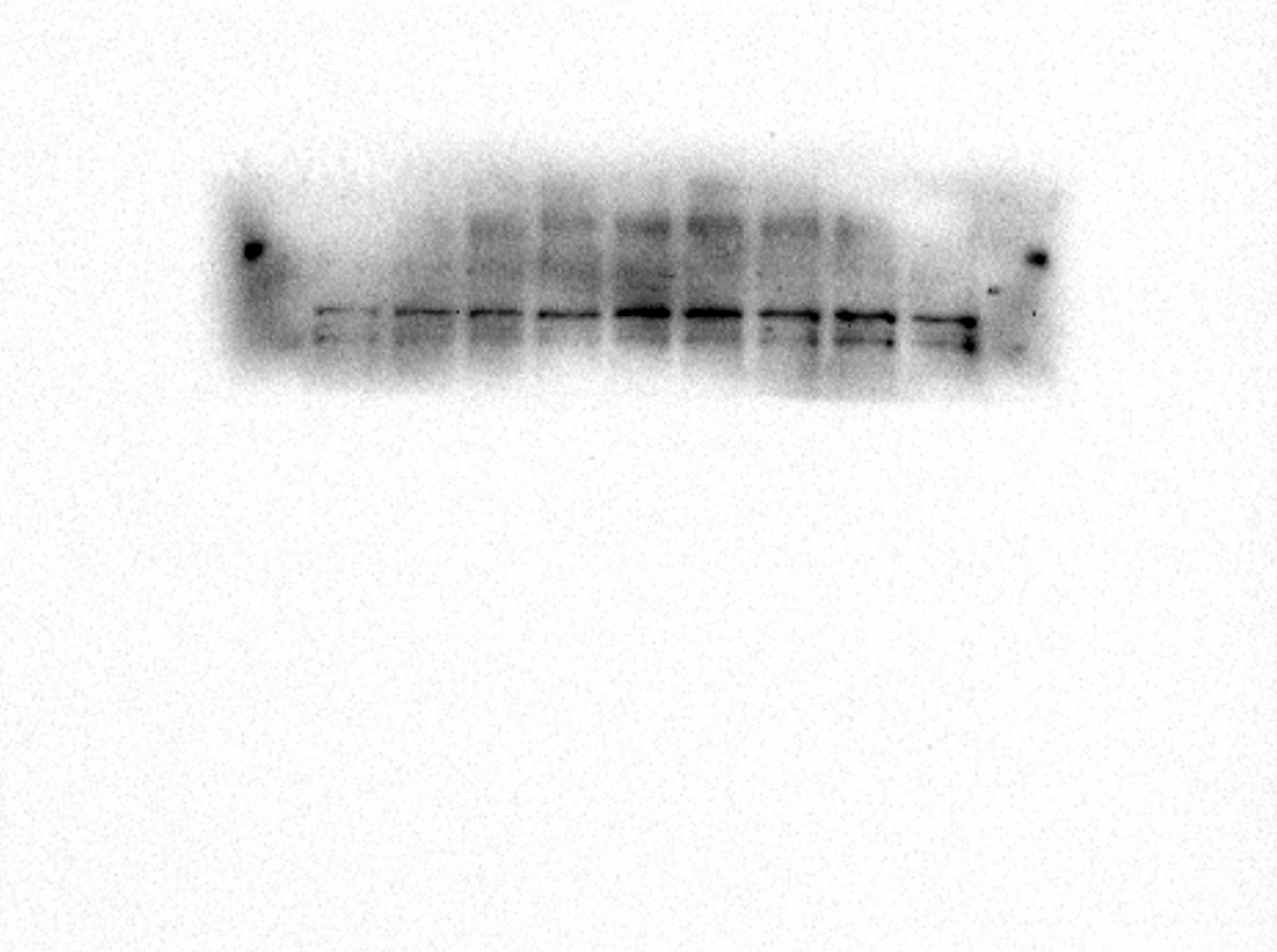

Supplement: Supplemental Information 52 [file peerj-14-21375-s052.zip › Figure 7E WB RAW MG132 KLHL40/2-1 KLHL40 0a╠M 0a╠M 0a╠M 5a╠M 5a╠M 5a╠M 10a╠M 10a╠M 10a╠M.tif]

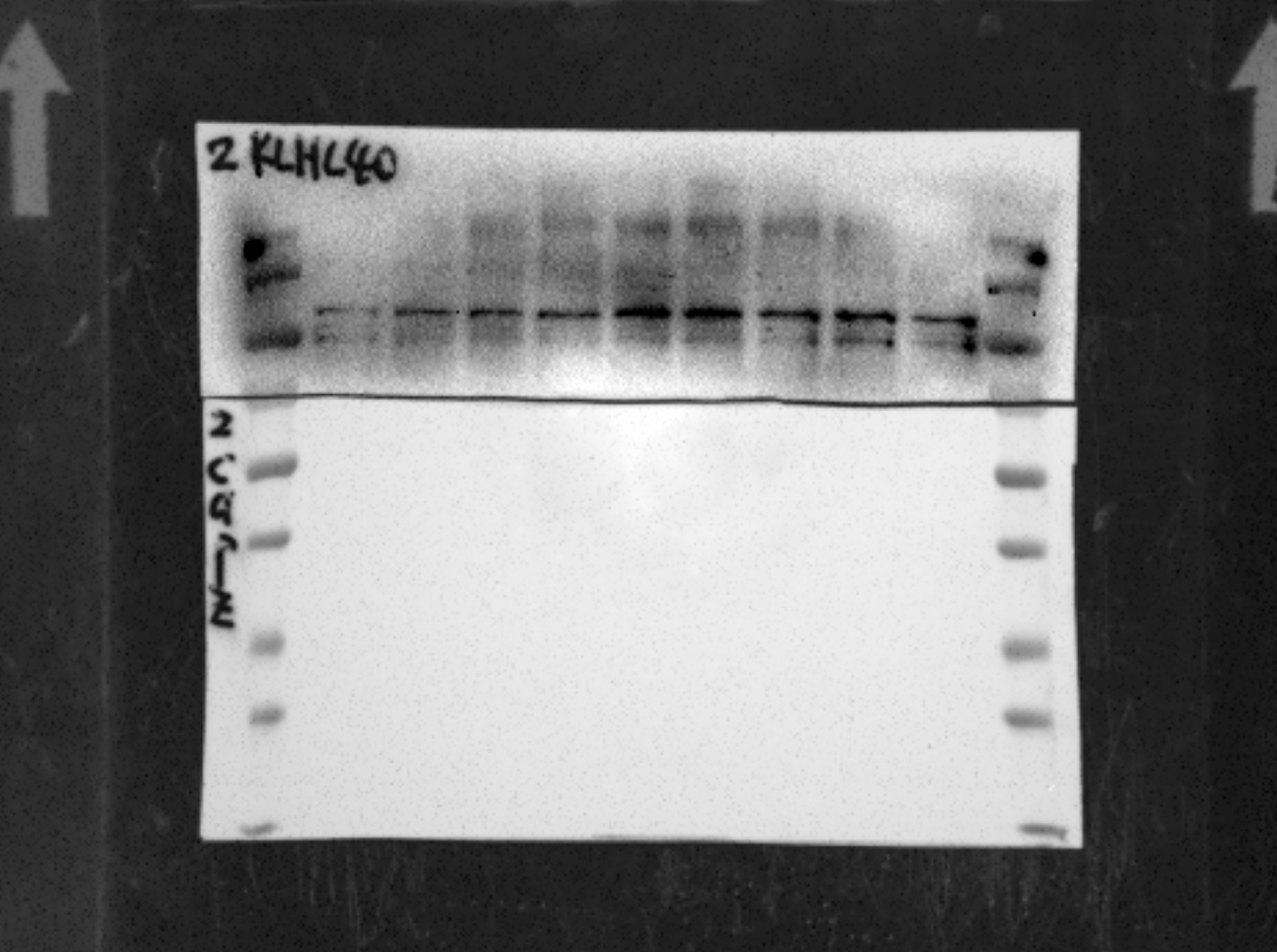

Supplement: Supplemental Information 52 [file peerj-14-21375-s052.zip › Figure 7E WB RAW MG132 KLHL40/2-2 KLHL40 0a╠M 0a╠M 0a╠M 5a╠M 5a╠M 5a╠M 10a╠M 10a╠M 10a╠M.tif]

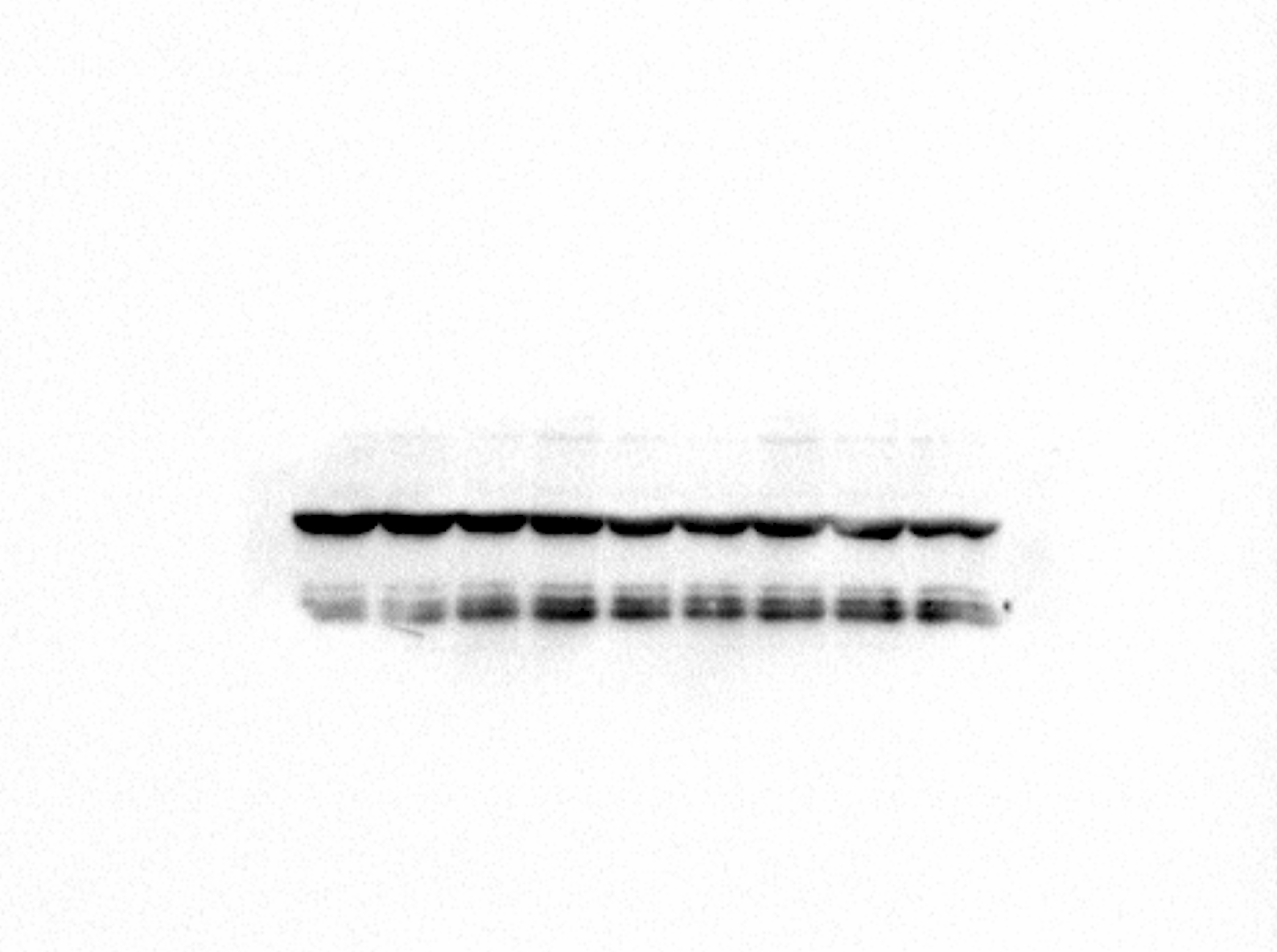

Supplement: Supplemental Information 52 [file peerj-14-21375-s052.zip › Figure 7E WB RAW MG132 KLHL40/2-3 ACTB 0a╠M 0a╠M 0a╠M 5a╠M 5a╠M 5a╠M 10a╠M 10a╠M 10a╠M.tif]

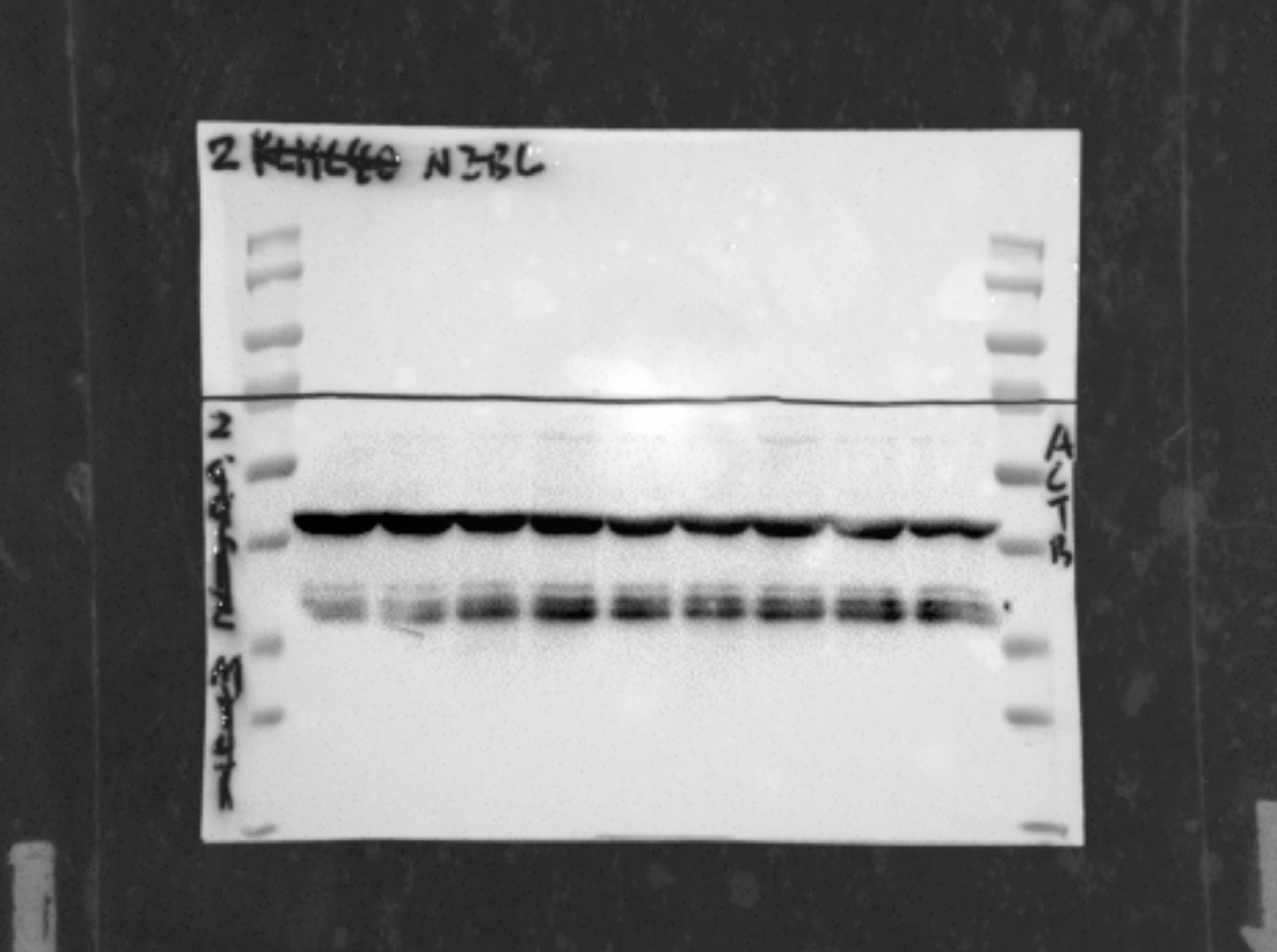

Supplement: Supplemental Information 52 [file peerj-14-21375-s052.zip › Figure 7E WB RAW MG132 KLHL40/2-4 ACTB 0a╠M 0a╠M 0a╠M 5a╠M 5a╠M 5a╠M 10a╠M 10a╠M 10a╠M.tif]

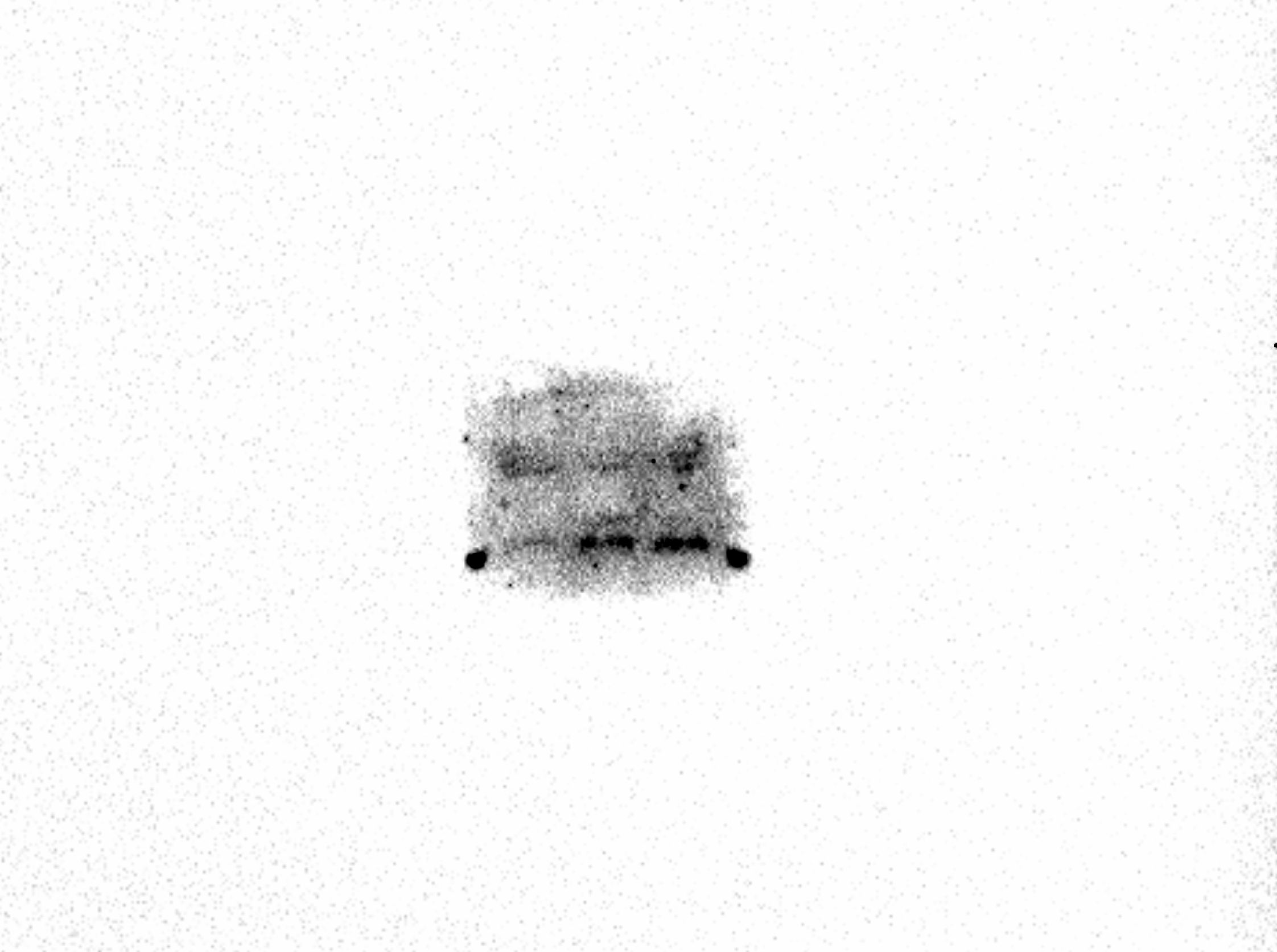

Supplement: Supplemental Information 52 [file peerj-14-21375-s052.zip › Figure 7E WB RAW MG132 KLHL40/3- KLHL40.tif]

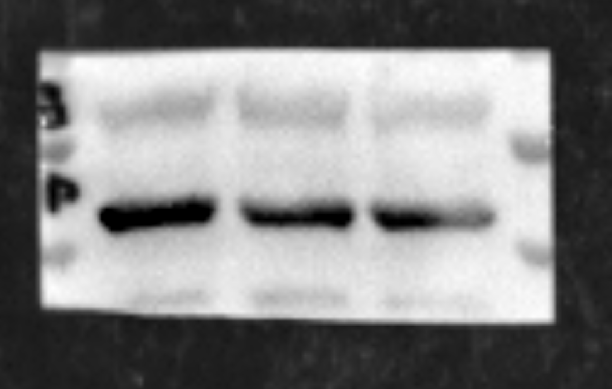

Supplement: Supplemental Information 52 [file peerj-14-21375-s052.zip › Figure 7E WB RAW MG132 KLHL40/3-ACTB.tif]

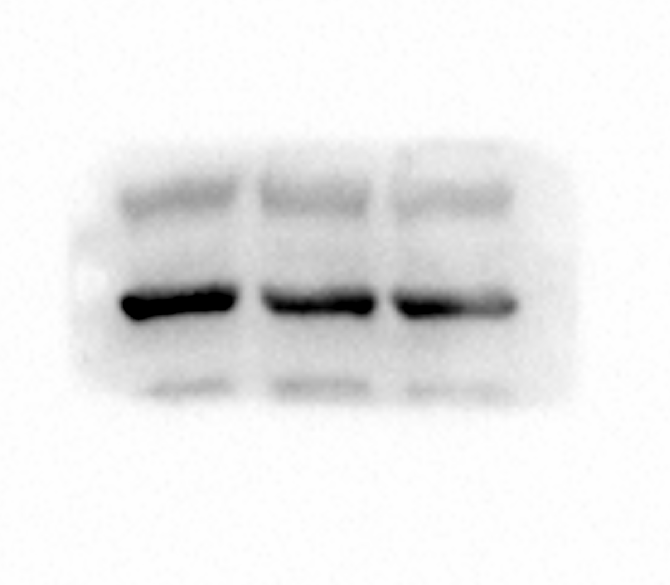

Supplement: Supplemental Information 52 [file peerj-14-21375-s052.zip › Figure 7E WB RAW MG132 KLHL40/3-ACTB+MARK.tif]

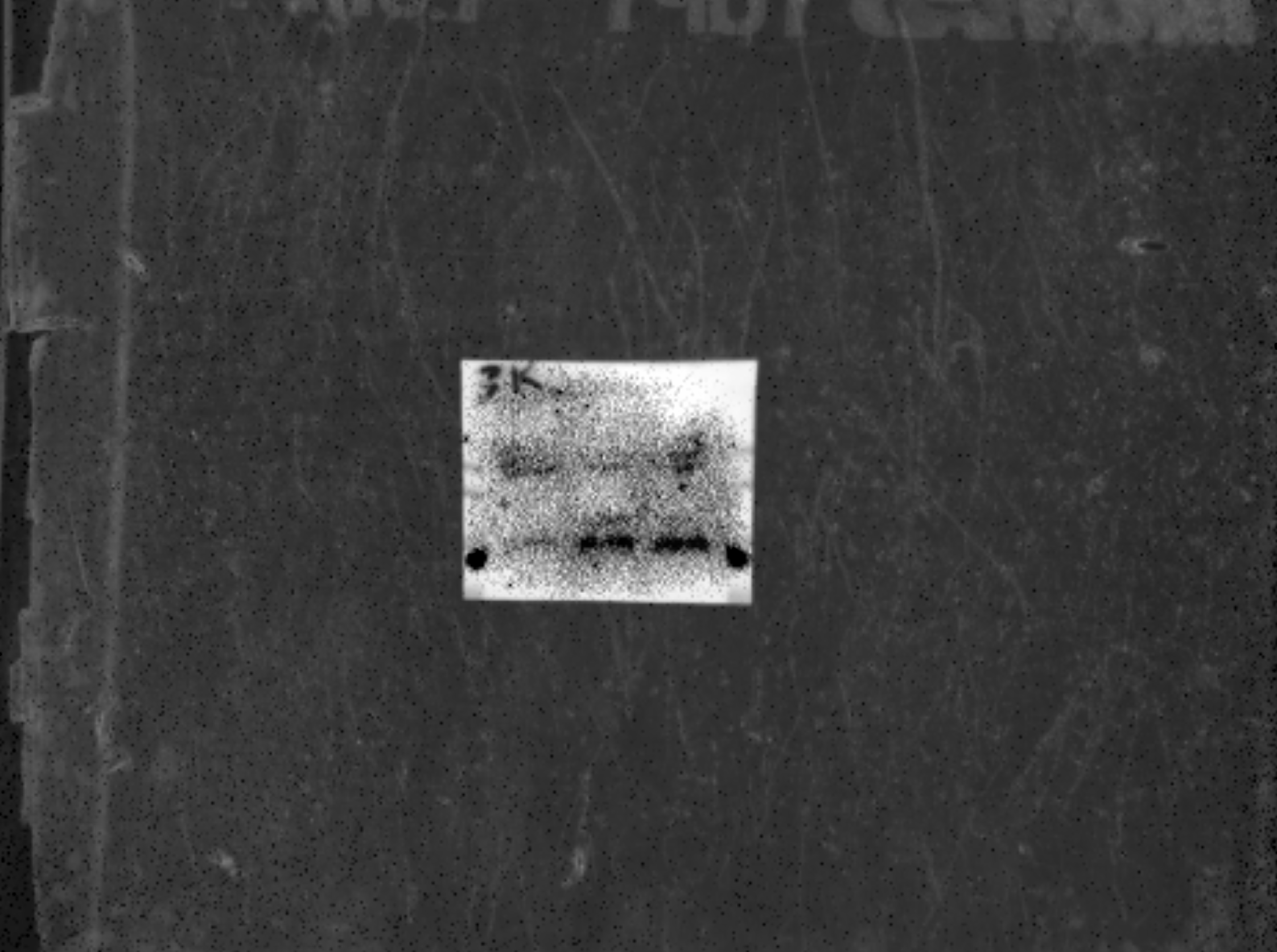

Supplement: Supplemental Information 52 [file peerj-14-21375-s052.zip › Figure 7E WB RAW MG132 KLHL40/3-KLHL40+MARK.tif]

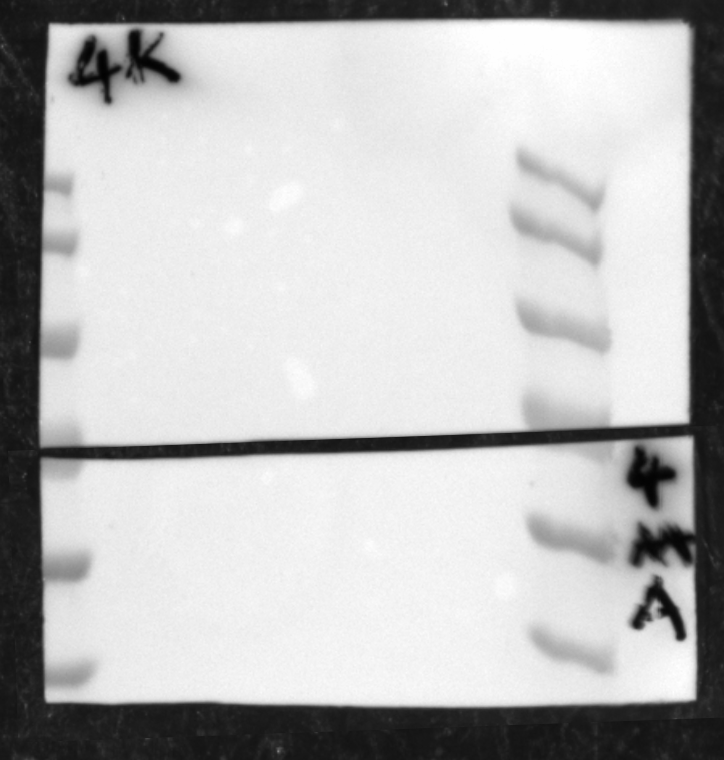

Supplement: Supplemental Information 52 [file peerj-14-21375-s052.zip › Figure 7E WB RAW MG132 KLHL40/TOTAL-1.tif]

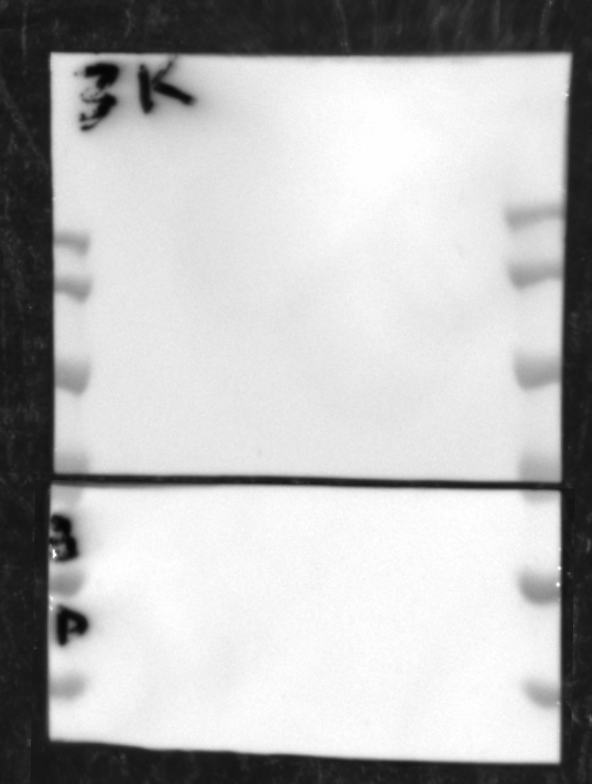

Supplement: Supplemental Information 52 [file peerj-14-21375-s052.zip › Figure 7E WB RAW MG132 KLHL40/TOTAL-3.tif]

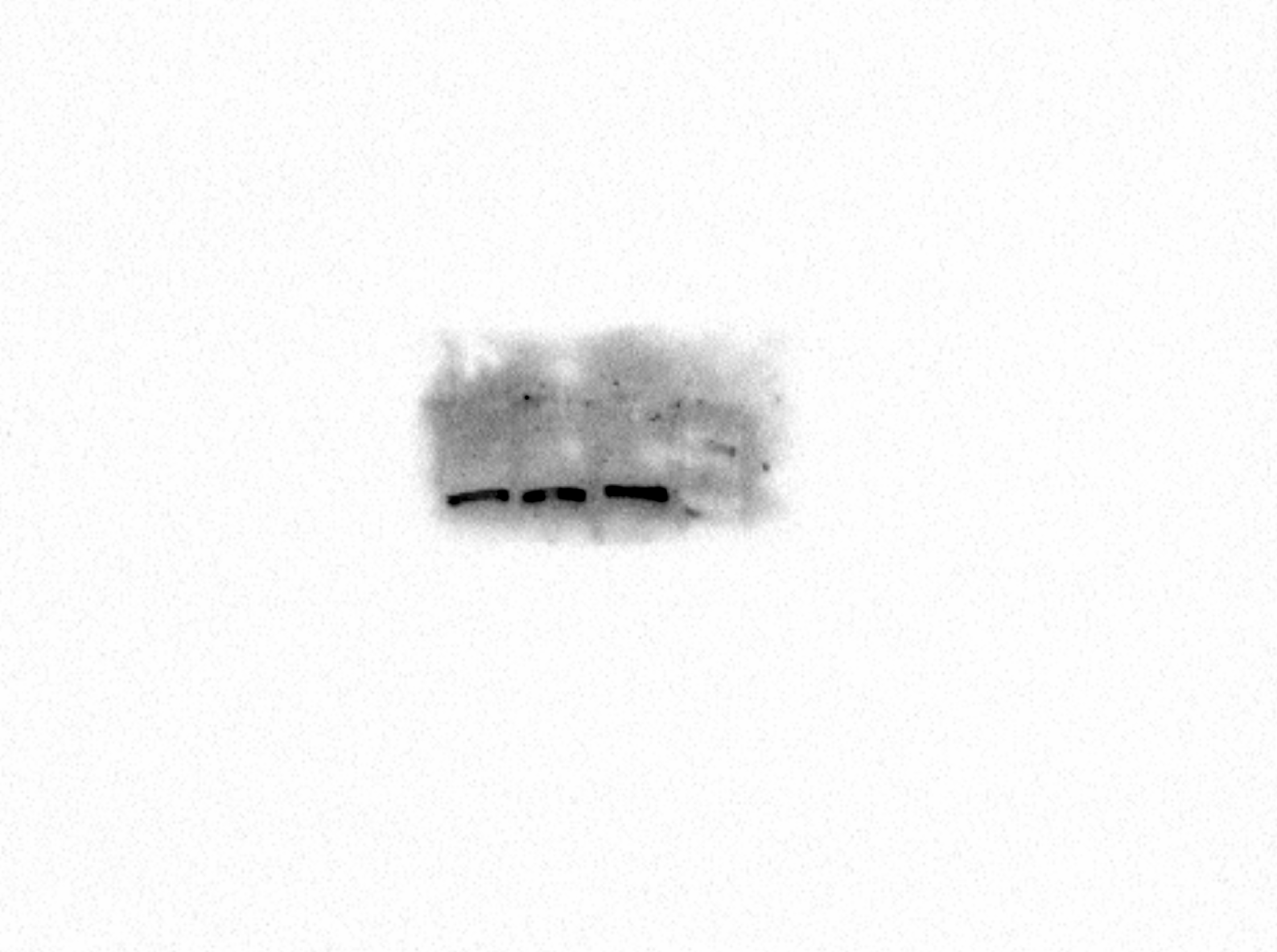

Supplement: Supplemental Information 53 [file peerj-14-21375-s053.zip › Figure 7J WB RAW Chloroquine KLHL40/1-1 KLHL40.tif]

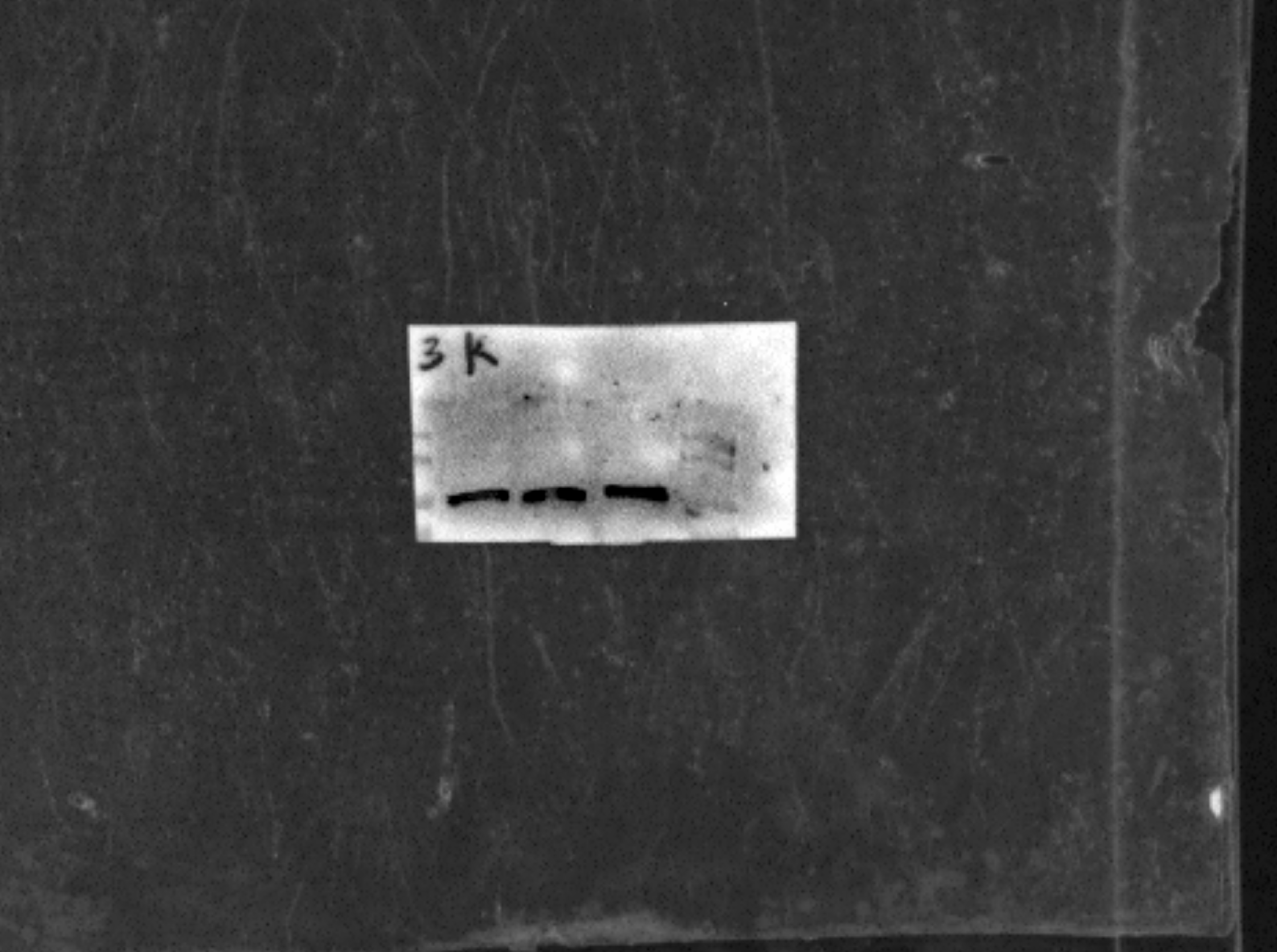

Supplement: Supplemental Information 53 [file peerj-14-21375-s053.zip › Figure 7J WB RAW Chloroquine KLHL40/1-2 KLHL40+MARK.tif]

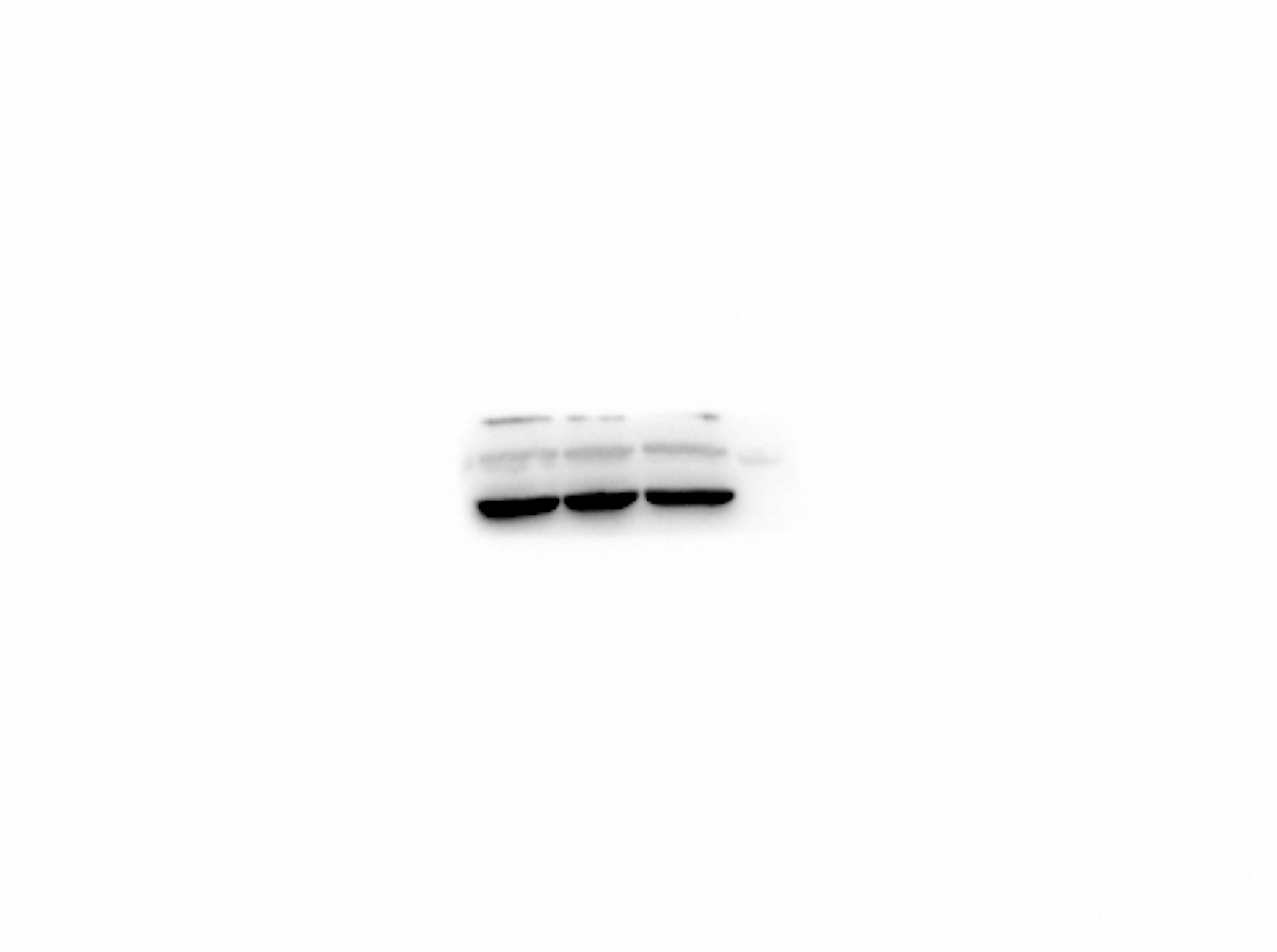

Supplement: Supplemental Information 53 [file peerj-14-21375-s053.zip › Figure 7J WB RAW Chloroquine KLHL40/1-3 ACTB.tif]

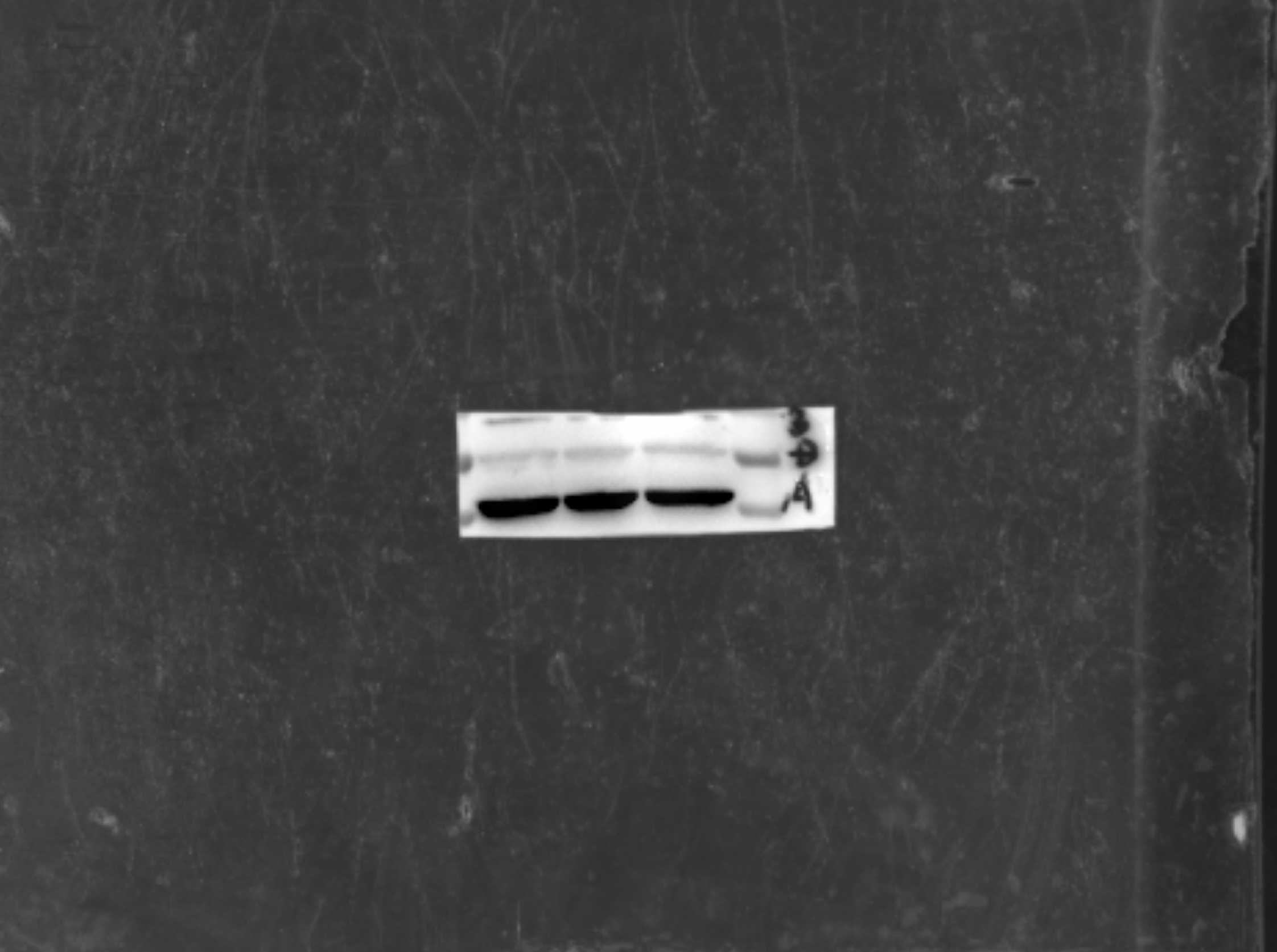

Supplement: Supplemental Information 53 [file peerj-14-21375-s053.zip › Figure 7J WB RAW Chloroquine KLHL40/1-4 ACTB+MARK.tif]

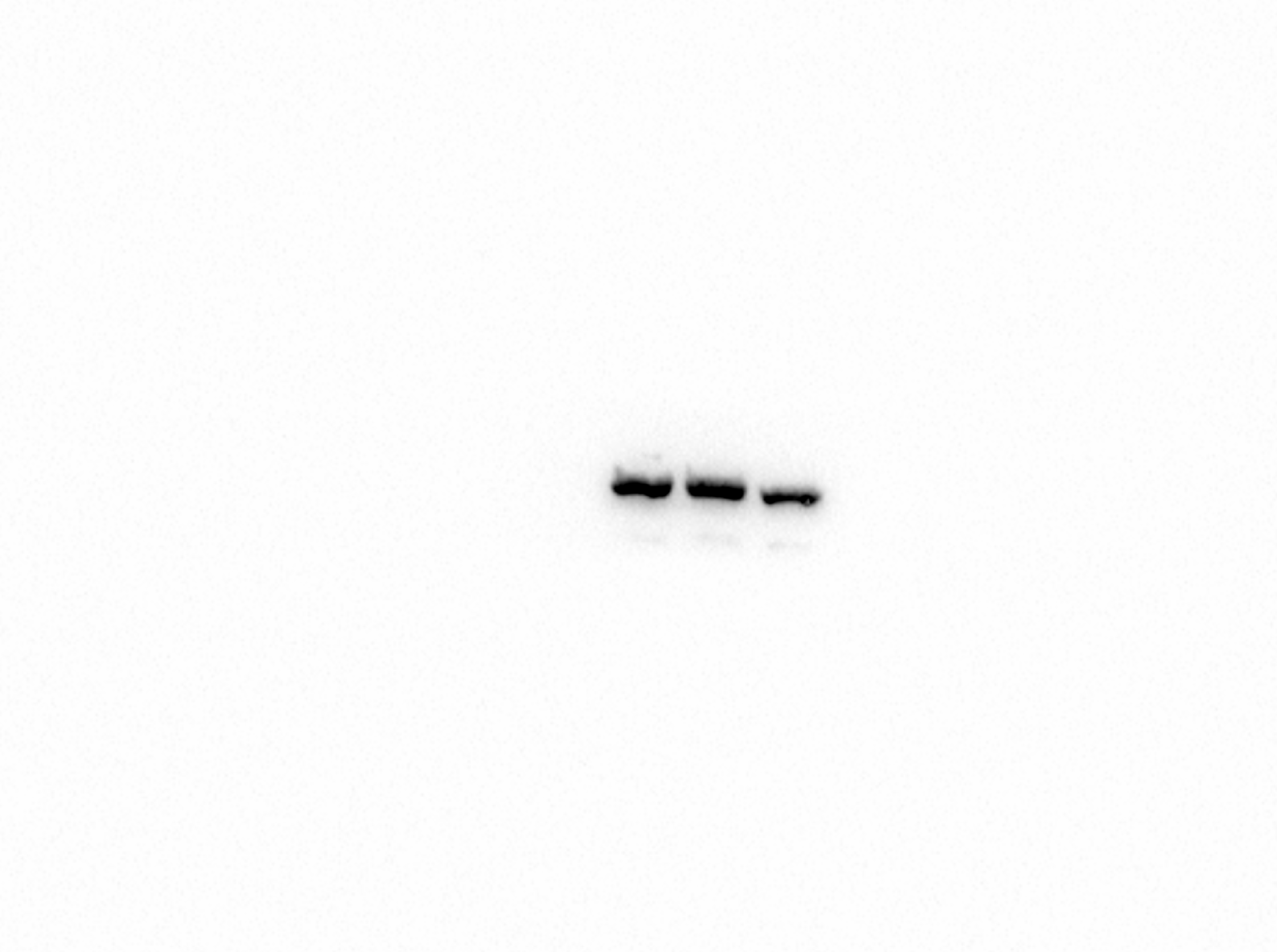

Supplement: Supplemental Information 53 [file peerj-14-21375-s053.zip › Figure 7J WB RAW Chloroquine KLHL40/2-ACTB.tif]

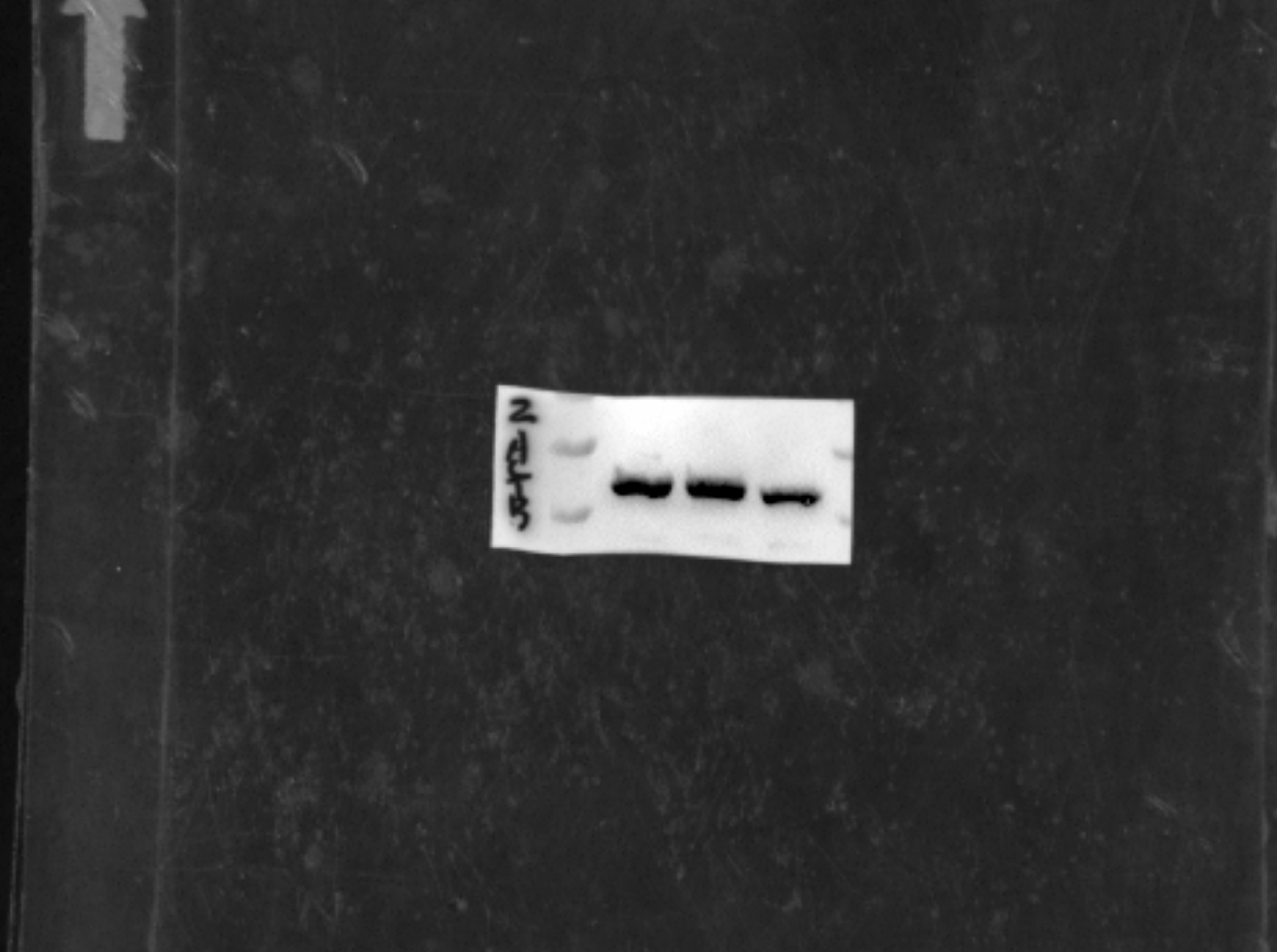

Supplement: Supplemental Information 53 [file peerj-14-21375-s053.zip › Figure 7J WB RAW Chloroquine KLHL40/2-ACTB+MARK.tif]

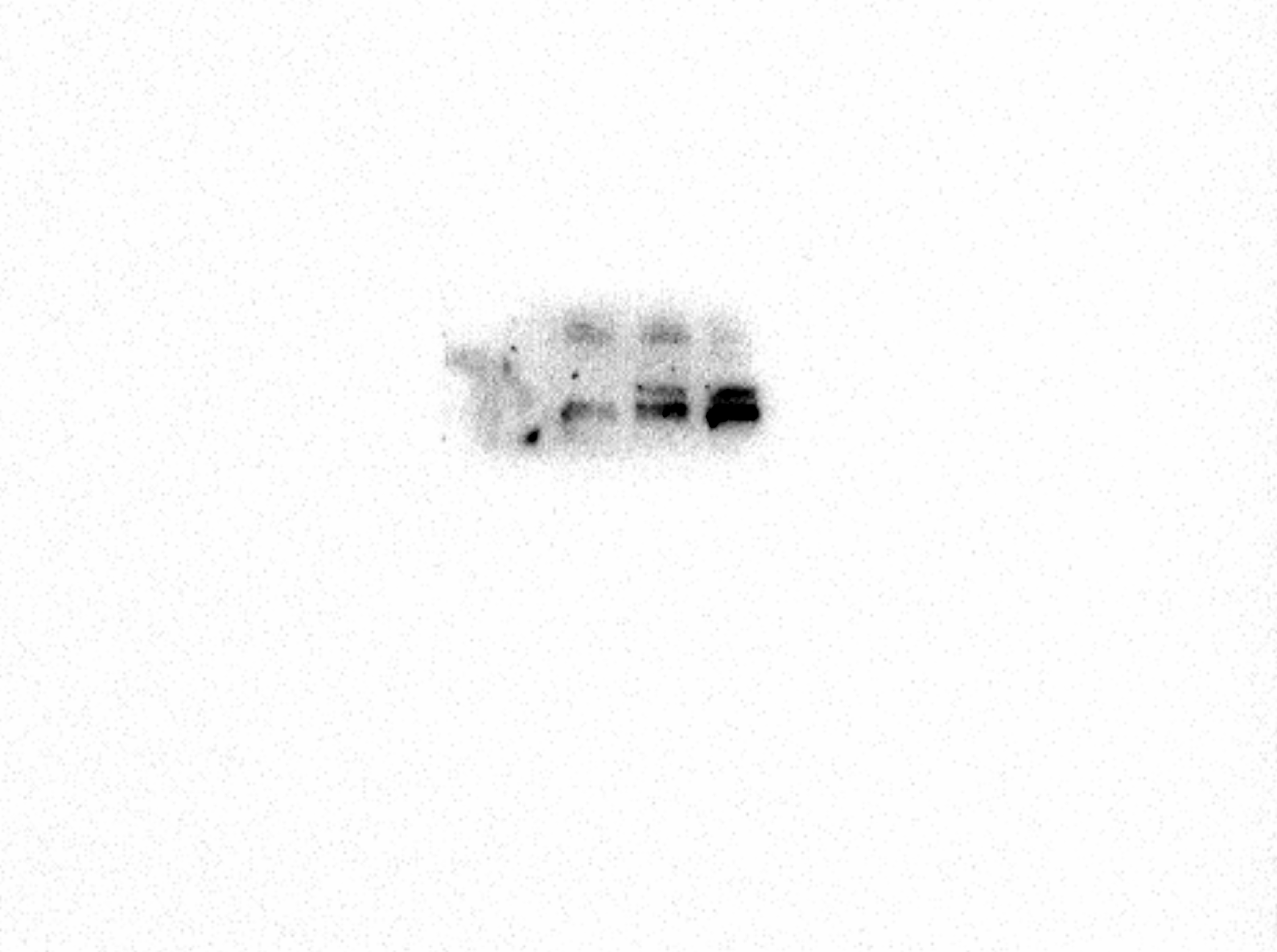

Supplement: Supplemental Information 53 [file peerj-14-21375-s053.zip › Figure 7J WB RAW Chloroquine KLHL40/2-KLHL40.tif]

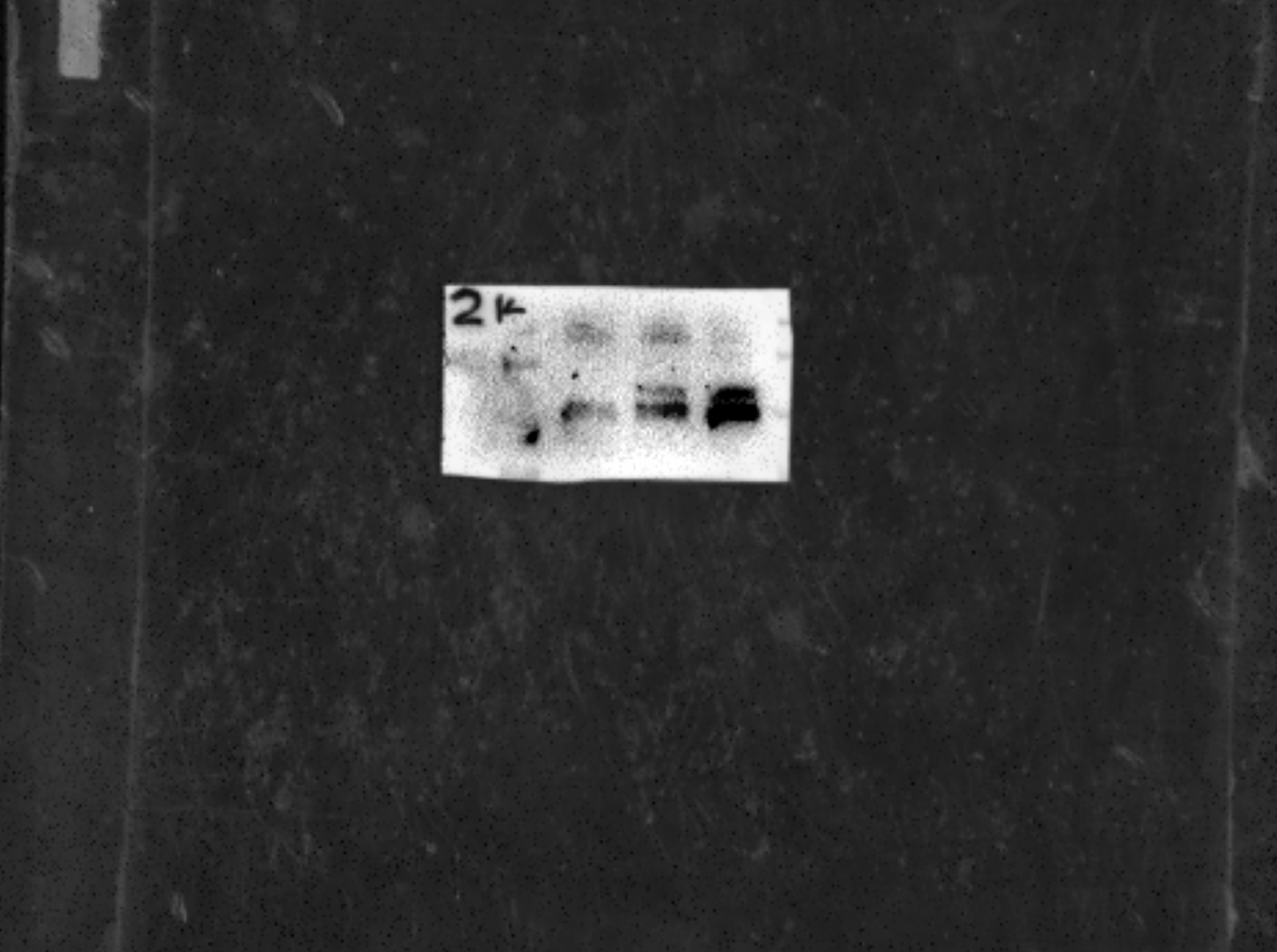

Supplement: Supplemental Information 53 [file peerj-14-21375-s053.zip › Figure 7J WB RAW Chloroquine KLHL40/2-KLHL40+MARK.tif]

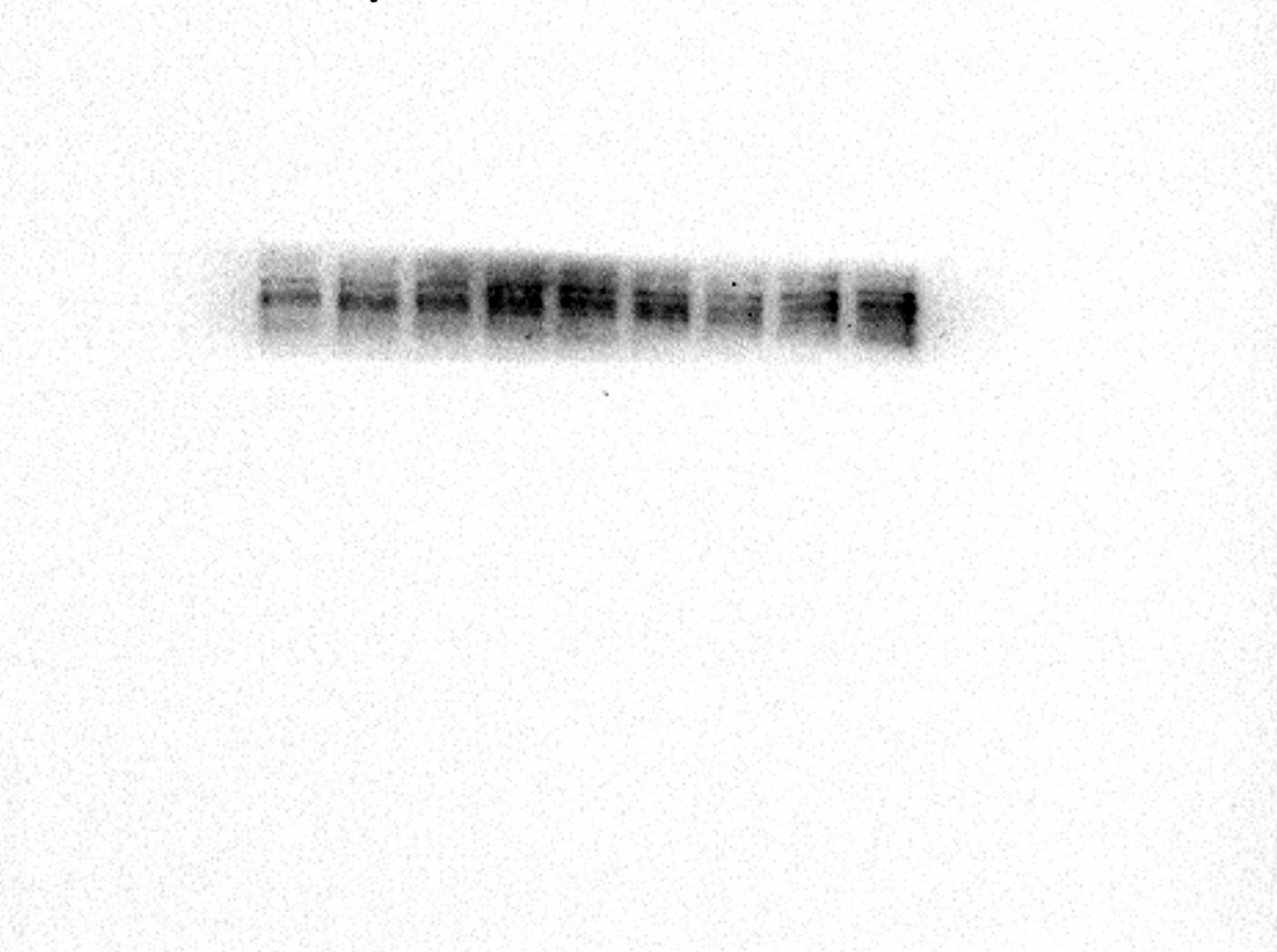

Supplement: Supplemental Information 53 [file peerj-14-21375-s053.zip › Figure 7J WB RAW Chloroquine KLHL40/3-1 KLHL40 0a╠M 0a╠M 0a╠M 50a╠M 50a╠M 50a╠M 100a╠M 100a╠M 100a╠M.tif]

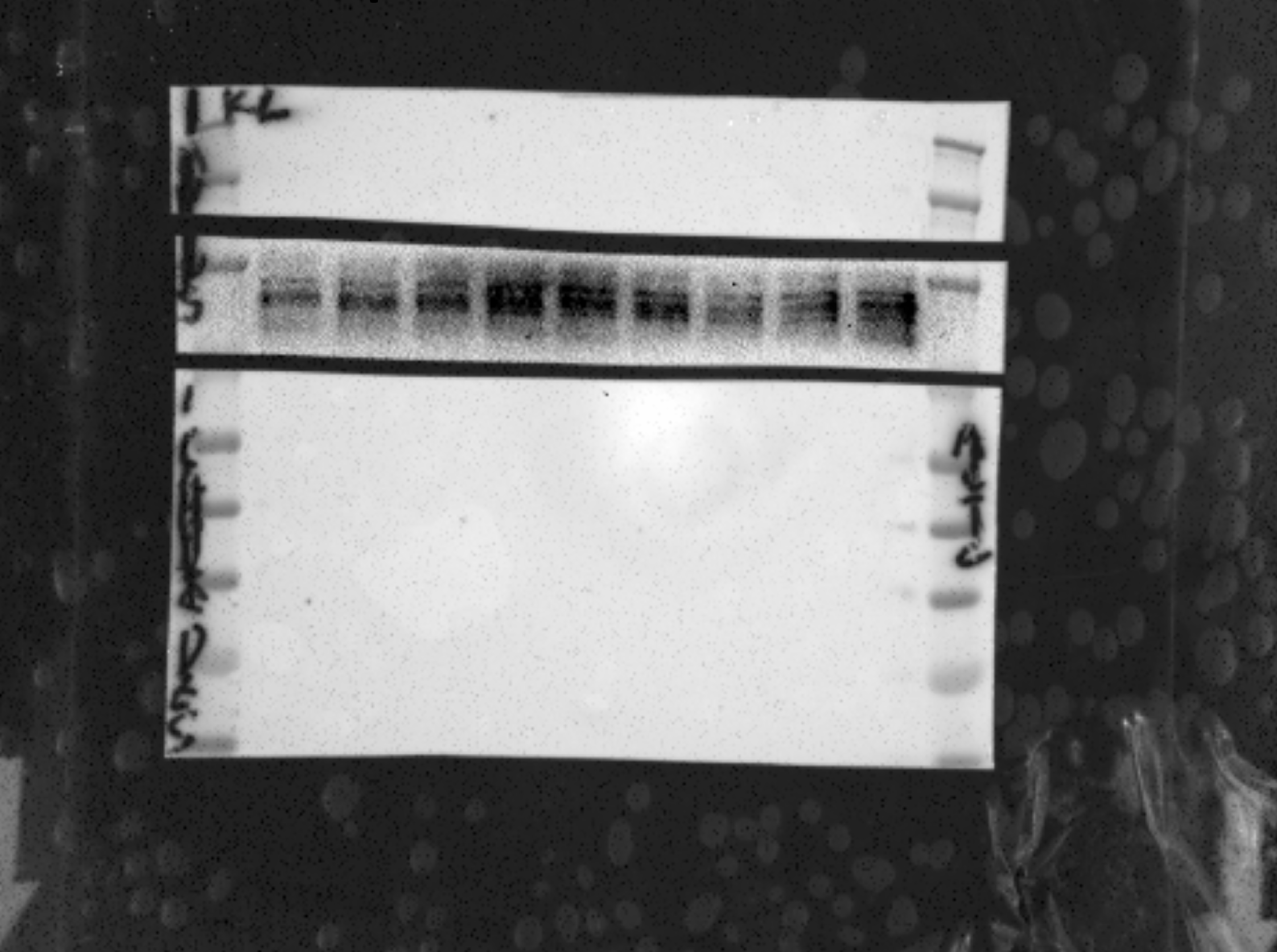

Supplement: Supplemental Information 53 [file peerj-14-21375-s053.zip › Figure 7J WB RAW Chloroquine KLHL40/3-2 KLHL40 0a╠M 0a╠M 0a╠M 50a╠M 50a╠M 50a╠M 100a╠M 100a╠M 100a╠M.tif]

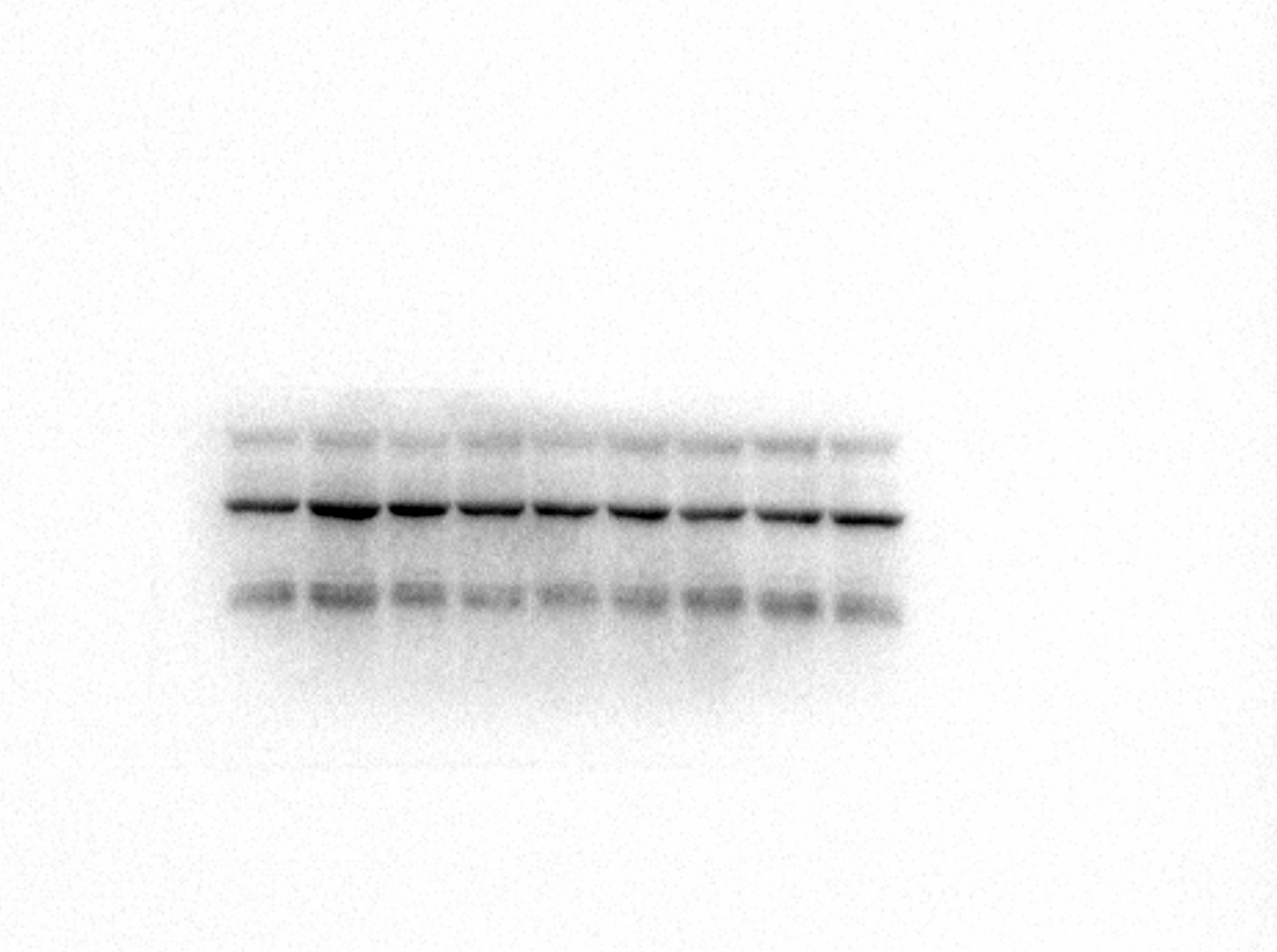

Supplement: Supplemental Information 53 [file peerj-14-21375-s053.zip › Figure 7J WB RAW Chloroquine KLHL40/3-3 ACTB 0a╠M 0a╠M 0a╠M 50a╠M 50a╠M 50a╠M 100a╠M 100a╠M 100a╠M.tif]

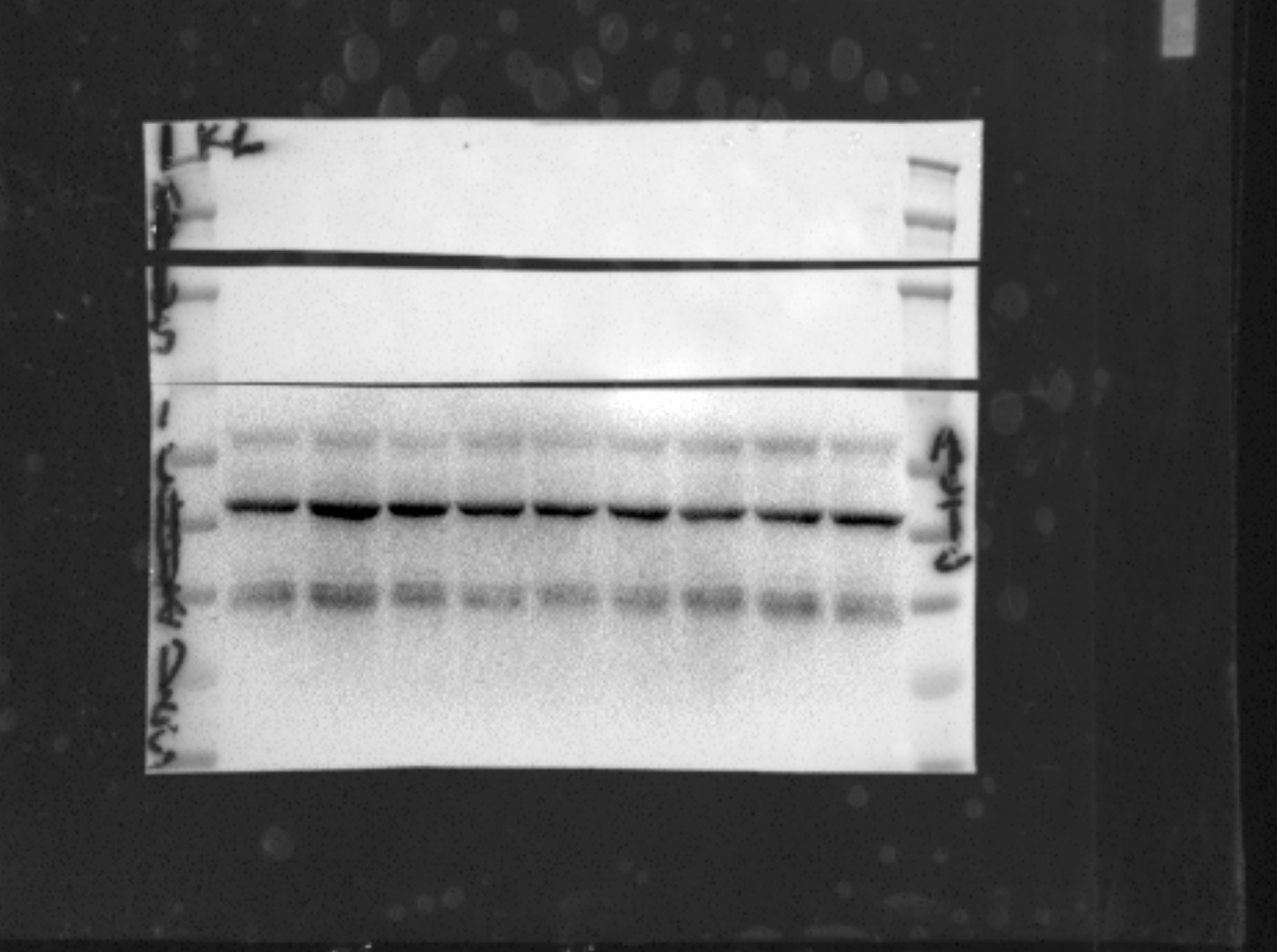

Supplement: Supplemental Information 53 [file peerj-14-21375-s053.zip › Figure 7J WB RAW Chloroquine KLHL40/3-4 ACTB 0a╠M 0a╠M 0a╠M 50a╠M 50a╠M 50a╠M 100a╠M 100a╠M 100a╠M.tif]

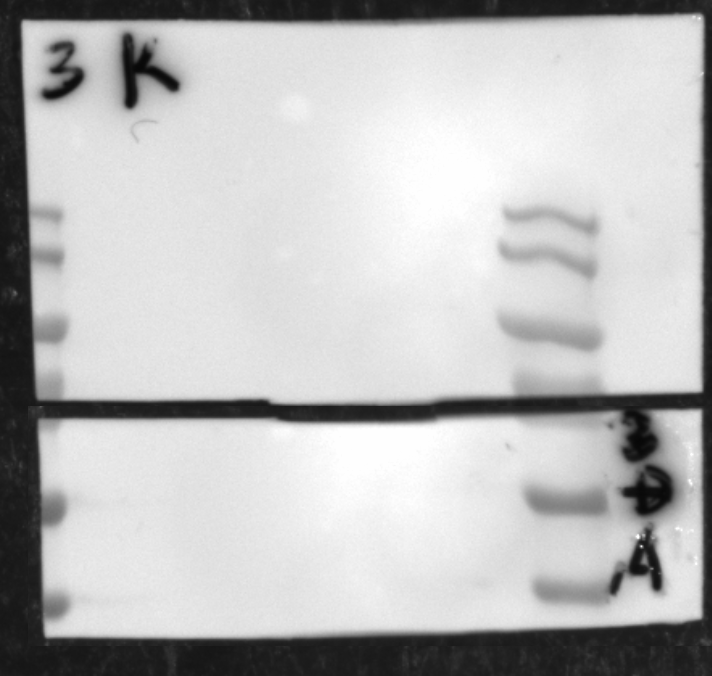

Supplement: Supplemental Information 53 [file peerj-14-21375-s053.zip › Figure 7J WB RAW Chloroquine KLHL40/TOTAL1.tif]

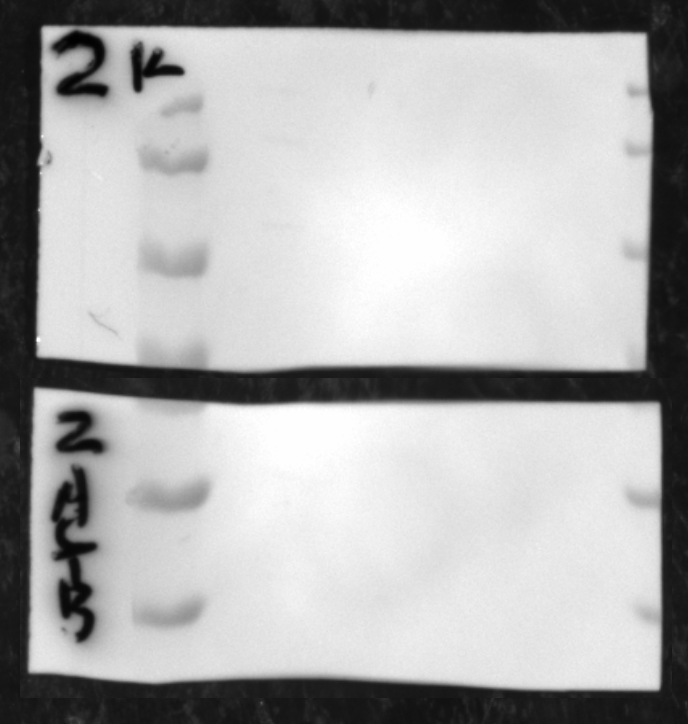

Supplement: Supplemental Information 53 [file peerj-14-21375-s053.zip › Figure 7J WB RAW Chloroquine KLHL40/TOTAL2.tif]
