# Supplementary material for: Post-infarction KLHL40-mediated regulation of cardiac sarcomeric integrity and function
Source: PeerJ. 2026 Jun 5;14:e21375. doi: 10.7717/peerj.21375 (PMC13245431; doi:10.7717/peerj.21375)
Supplement: Supplemental Information 51 [file peerj-14-21375-s051.zip › Figure 7 Labeled Western blot.docx]

# Figure. 7E Wb MG132 0,5,10μM

| **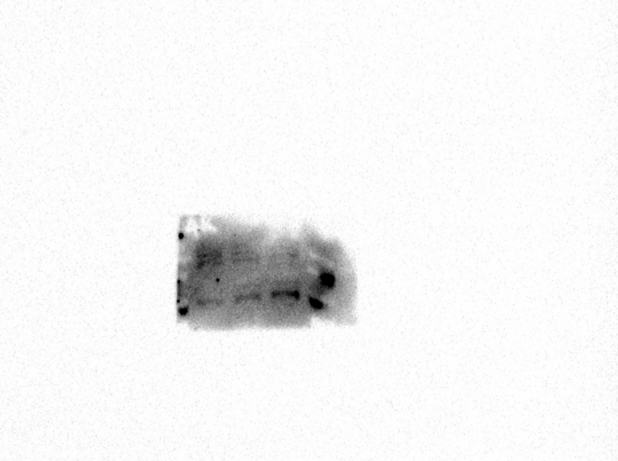** | **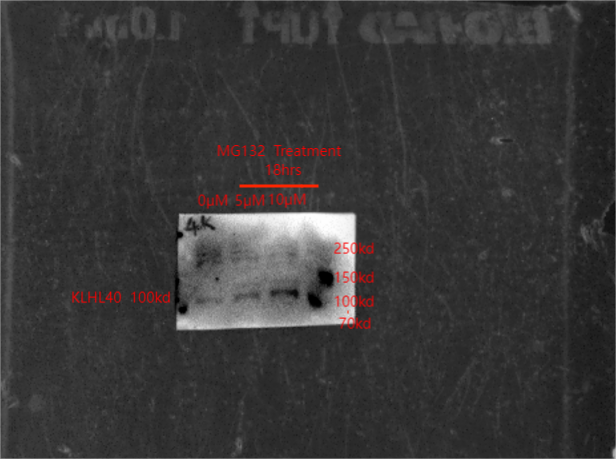** | 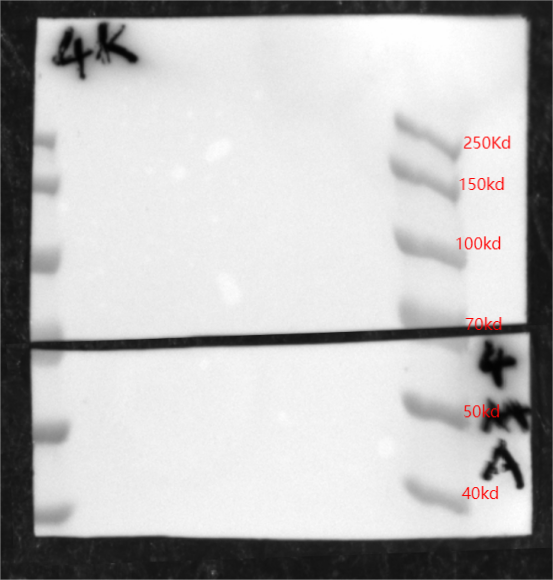 |
| --- | --- | --- |
| 1-1 KLHL40 | 1-1 KLHL40 MARK |  |
| 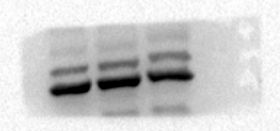 | 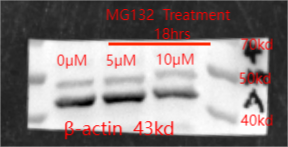 |  |
| 1- ACTB | 1- ACTB MARK | TOTAL |
| 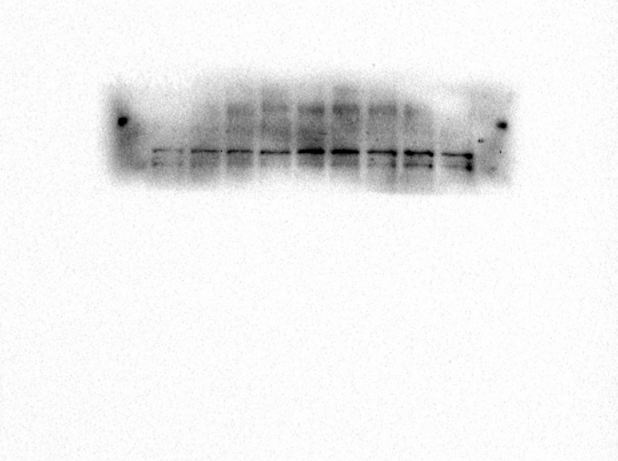 | 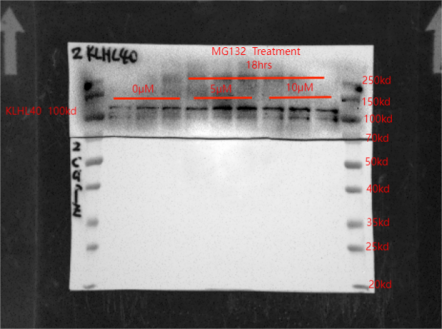 |  |
| 2-1 KLHL40 0μM 0μM 0μM 5μM 5μM 5μM 10μM 10μM 10μM | 2-1 KLHL40 0μM 0μM 0μM 5μM 5μM 5μM 10μM 10μM 10μM MARK |  |
| 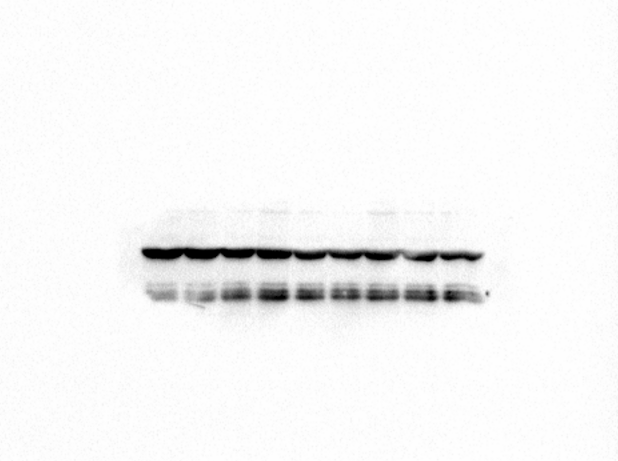 | 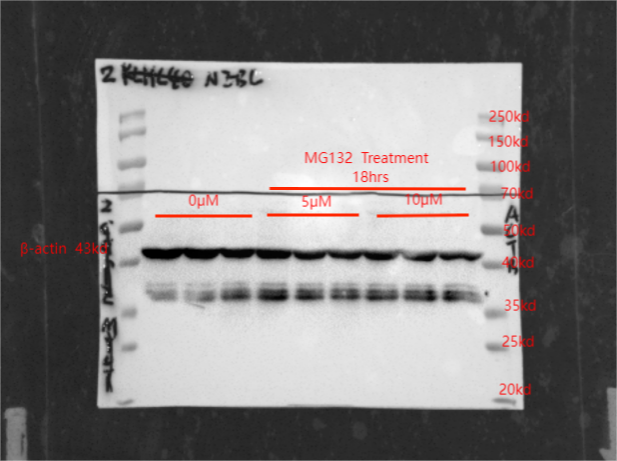 |  |
| 2-3 ACTB 0μM 0μM 0μM 5μM 5μM 5μM 10μM 10μM 10μM | 2-3 ACTB 0μM 0μM 0μM 5μM 5μM 5μM 10μM 10μM 10μM MARK |  |
| 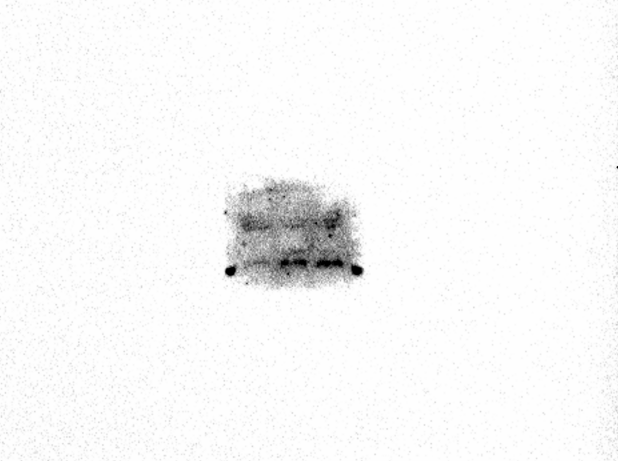 | 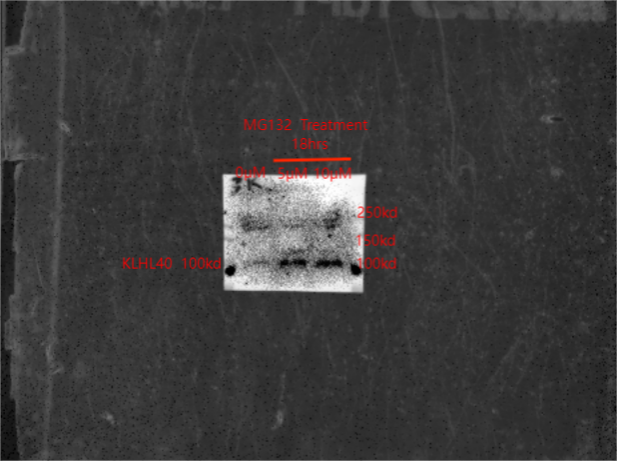 | 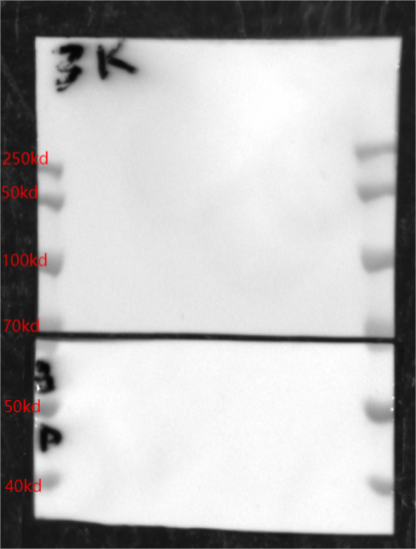 |
| 3-1 KLHL40 | 3-1 KLHL40 MARK |  |
| 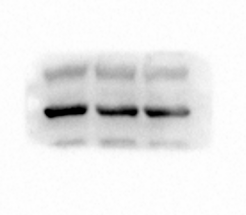 | 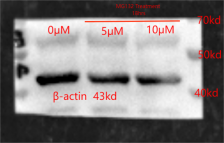 |  |
| 3-3 ACTB | 3-3 ACTB MARK | TOTAL |

# Figure. 7J Wb Chloroquine

| **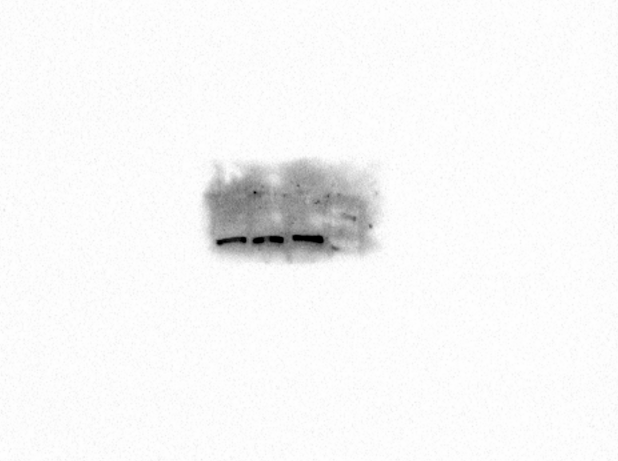** | **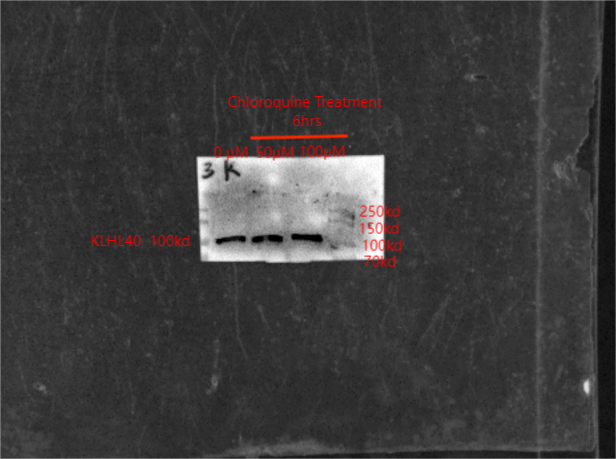** | 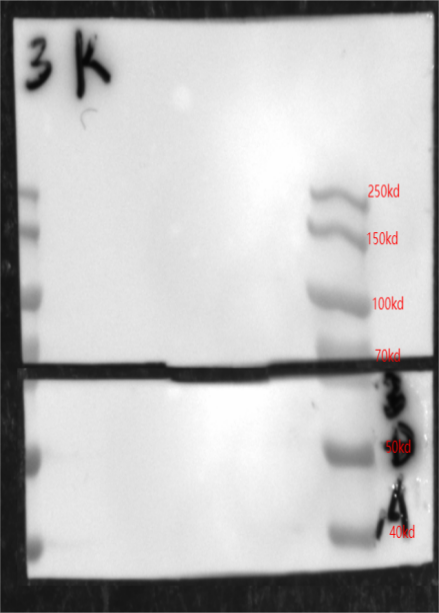 |  |
| --- | --- | --- | --- |
| 1-1 KLHL40 | 1-2 KLHL40 MARK |  |  |
| 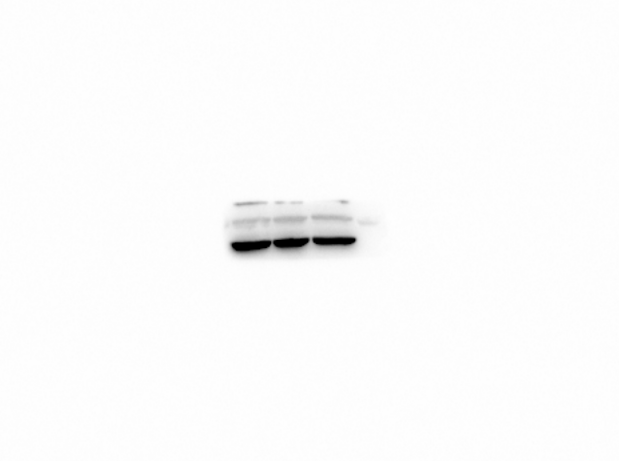 | 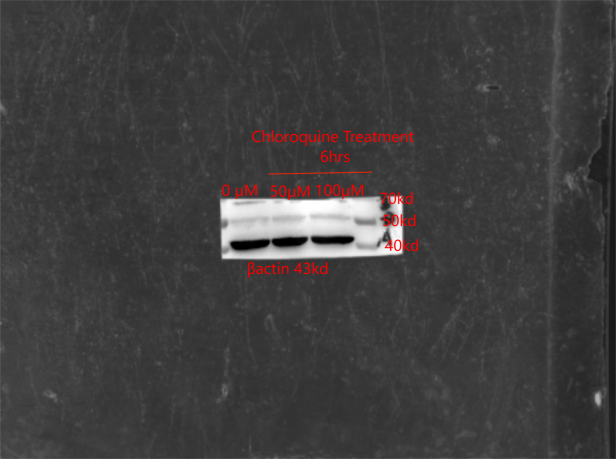 |  |  |
| 1-3 ACTB | 1-4 ACTB MARK | TOTAL |  |
| 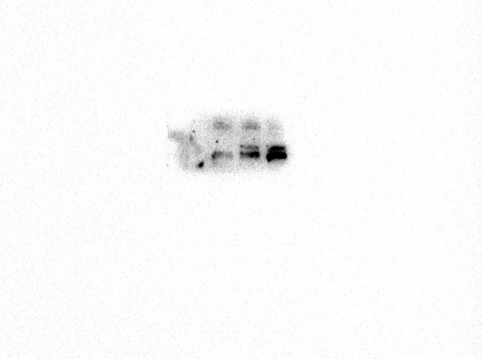 | 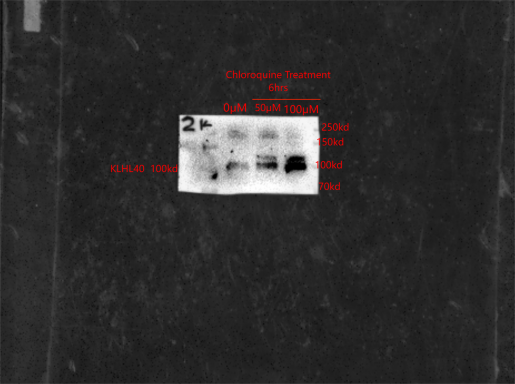 | 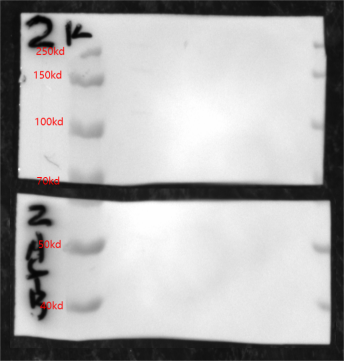 |  |
| 2-1 KLHL40 | 2-2 KLHL40 MARK |  |  |
| 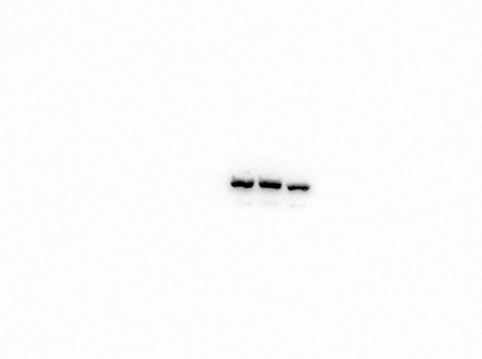 | 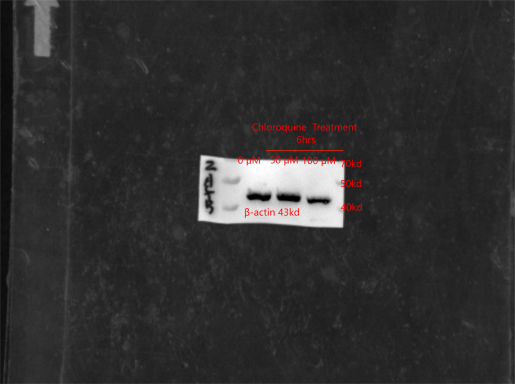 |  |  |
| 2-ACTB | 2-ACTB+MARK | TOTAL |  |
| 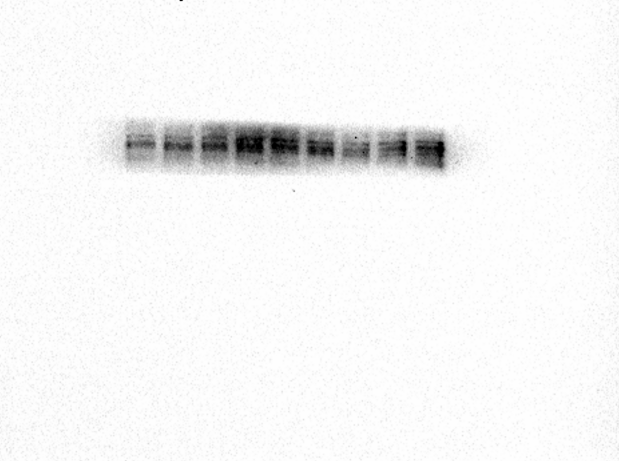 | 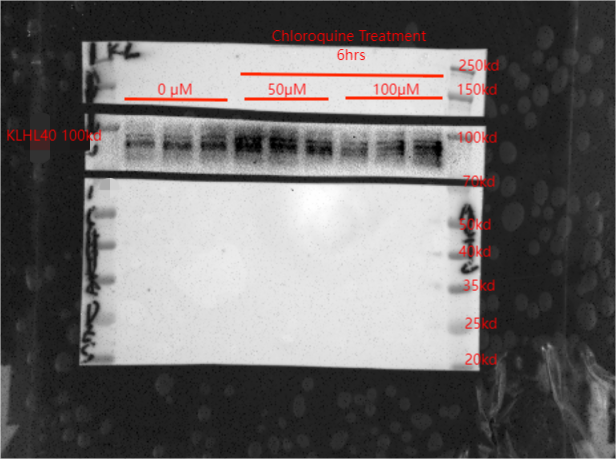 |  |  |
| 3-1 KLHL40 0μM 0μM 0μM 50μM 50μM 50μM 100μM 100μM 100μM | 3-2 KLHL40 0μM 0μM 0μM 50μM 50μM 50μM 100μM 100μM 100μM MARK |  |  |
| 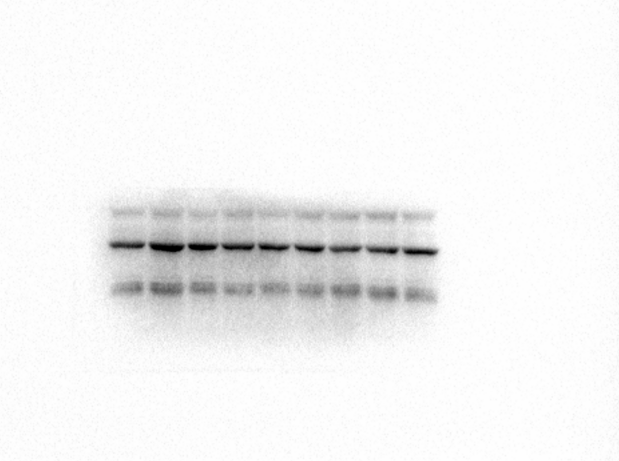 | 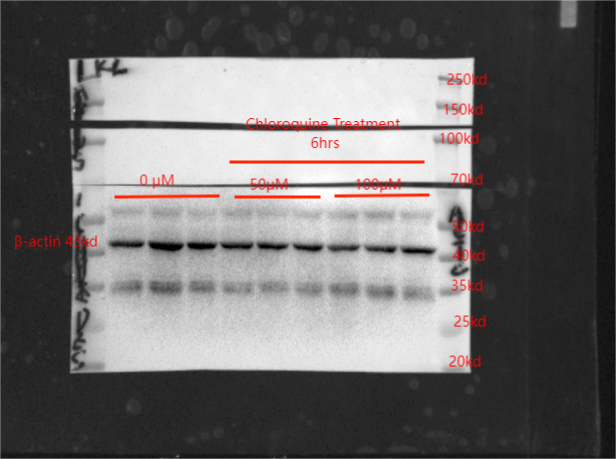 |  |  |
| 3-3 ACTB 0μM 0μM 0μM 50μM 50μM 50μM 100μM 100μM 100μM | 3-4 ACTB 0μM 0μM 0μM 50μM 50μM 50μM 100μM 100μM 100μM MARK |  |  |
